# Beyond the Big Five: Investigating Myostatin Structure, Polymorphism and Expression in *Camelus dromedarius*

**DOI:** 10.3389/fgene.2019.00502

**Published:** 2019-06-07

**Authors:** Maria Favia, Robert Fitak, Lorenzo Guerra, Ciro Leonardo Pierri, Bernard Faye, Ahmad Oulmouden, Pamela Anna Burger, Elena Ciani

**Affiliations:** ^1^Department of Biosciences, Biotechnologies and Biopharmaceutics, University of Bari “Aldo Moro”, Bari, Italy; ^2^Research Institute of Wildlife Ecology, Vetmeduni, Vienna, Austria; ^3^Department of Biology, Duke University, Durham, NC, United States; ^4^CIRAD, UMR SELMET, Montpellier, France; ^5^Département Sciences du Vivant, Université de Limoges, Limoges, France

**Keywords:** *Camelus dromedarius*, myostatin, skeletal muscle, Single Nucleotide Polymorphisms, Next Generation Sequencing, Digital Droplet PCR, Western Blot, 3D protein comparative modeling

## Abstract

Myostatin, a negative regulator of skeletal muscle mass in animals, has been shown to play a role in determining muscular hypertrophy in several livestock species, and a high degree of polymorphism has been previously reported for this gene in humans and cattle. In this study, we provide a characterization of the myostatin gene in the dromedary (*Camelus dromedarius*) at the genomic, transcript and protein level. The gene was found to share high structural and sequence similarity with other mammals, notably Old World camelids. 3D modeling highlighted several non-conservative SNP variants compared to the bovine, as well as putative functional variants involved in the stability of the myostatin dimer. NGS data for nine dromedaries from various countries revealed 66 novel SNPs, all of them falling either upstream or downstream the coding region. The analysis also confirmed the presence of three previously described SNPs in intron 1, predicted here to alter both splicing and transcription factor binding sites (TFBS), thus possibly impacting myostatin processing and/or regulation. Several putative TFBS were identified in the myostatin upstream region, some of them belonging to the myogenic regulatory factor family. Patterns of SNP distribution across countries, as suggested by Bayesian clustering of the nine dromedaries using the 69 SNPs, pointed to weak geographic differentiation, in line with known recurrent gene flow at ancient trading centers along caravan routes. Myostatin expression was investigated in a set of 8 skeletal muscles, both at transcript and protein level, via Digital Droplet PCR and Western Blotting, respectively. No significant differences were observed at the transcript level, while, at the protein level, the only significant differences concerned the promyostatin dimer (75 kDa), in four pair-wise comparisons, all involving the *tensor fasciae latae* muscle. Beside the mentioned band at 75 kDa, additional bands were observed at around 40 and 25 kDa, corresponding to the promyostatin monomer and the active C-terminal myostatin dimer, respectively. Their weaker intensity suggests that the unprocessed myostatin dimers could act as important reservoirs of slowly available myostatin forms. Under this assumption, the sequential cleavage steps may contribute additional layers of control within an already complex regulatory framework.

## Introduction

Myostatin (alias growth and differentiation factor-8, GDF8), a member of the transforming growth factor-β (TGF-β) super-family, is a negative regulator of skeletal muscle mass in animals during development and growth. It is exclusively expressed in skeletal muscle during embryogenesis, while in adults is also detected, at a much lower level, in other tissues (e.g., heart, adipose tissue, mammary gland) (McPherron et al., 1997; Ji et al., 1998; Sharma et al., 1999; Morissette et al., 2006; Shyu et al., 2006; Allen et al., 2008). Expression in these tissues can be upregulated under pathological conditions, such as myocardial infarction (Sharma et al., 1999), obesity (Allen et al., 2008) or experimentally induced skeletal muscle atrophy (Rodriguez et al., 2014), while it can be down regulated during chronic exercise (Carlson et al., 1999; Reardon et al., 2001; Kim et al., 2005; Matsakas et al., 2006; Allen et al., 2009; Gustafsson et al., 2010).

Like other TGF-β super-family members, myostatin is synthesized as a precursor protein (375 amino acids), referred to as pre-promyostatin. After translocation to the endoplasmic reticulum, it goes through a first cleavage to remove a 24-amino acid signal peptide and it forms a disulfide-linked homodimer (promyostatin dimer). Within the Golgi, the promyostatin dimer may be further cleaved by the furin family of protein convertases to generate two NH_2_-terminal (27.7 kDa, each) and two disulfide-linked COOH-terminal fragments (12.4 kDa, each) (Lee and McPherron, 2001; Thies et al., 2001). The two NH_2_-terminal fragments (also referred to as pro-domains) may complex with the COOH-terminal dimer (also referred to as active myostatin) via a non-covalent bound that maintains myostatin in a latent state by rendering it unable to engage its receptors (Wolfman et al., 2003; Jiang et al., 2004). The “latent myostatin complex” (Lee and McPherron, 2001; Thies et al., 2001) may be secreted in the extracellular space, though it has been shown that, in skeletal myocytes, myostatin is mainly secreted as uncleaved promyostatin (Anderson et al., 2008; Pirruccello-Straub et al., 2018). In the extracellular space, the action of furin protein convertase and metalloproteinases (like BMP-1, TLL-1, and TLL-2) may finally convert the uncleaved promyostatin and the latent complex, respectively, into the active form of myostatin (Lakshman et al., 2009). Notwithstanding, in serum, myostatin has been shown to exist mainly as a latent complex (Hill et al., 2002; Zimmers et al., 2002; Lee, 2010). The active myostatin present in plasma circulates bound to several proteins (Miura et al., 2006; Cash et al., 2009; Walker et al., 2015), including follistatin, FSTL3, GASP1, GASP2 and decorin, that prevent it binding to the receptor and activating signaling (Hill et al., 2002, 2003; Lee, 2008; Cotton et al., 2018). Composite pools of myostatin are hence available at the various compartments, suggesting that extracellular processing of the protein may be a key regulatory step for its signaling (Anderson et al., 2008). The presence, at various extents, of the myostatin active form in the extracellular space and in the serum is consistent with the postulated autocrine, paracrine (Gao et al., 2013), and/or endocrine manner of function (Zimmers et al., 2002).

Upon binding to the target cell, myostatin induces the formation of a heterotetrameric complex made of two activin responsive type II receptors (ActIIRA or, preferentially, ActIIRB) and two type I receptors, either activin (ALK4) or TGF-β (ALK5) (Lee and McPherron, 2001; Rebbapragada et al., 2003). Signaling is hence initiated by phosphorylation of SMAD2 or SMAD3, operated by the type I receptors, followed by translocation of SMADs to the nucleus for modulation of gene expression (Huang et al., 2011). In particular, in skeletal muscle, myostatin is known to block the transcription of genes responsible for the myogenesis, among which MyoD, a transcriptional factor that is involved in skeletal muscle development and repair (Megeney et al., 1996; Cornelison et al., 2000; Guttridge et al., 2000; Montarras et al., 2000). Beside the above mentioned canonical pathway, two other pathways have been highlighted, involving MAPK activation or inhibition of Akt signaling (Elkina et al., 2011).

Myostatin genomic organization was first provided for the murine species by McPherron et al. (1997) who also reported on the highly conserved nature of the myostatin transcript across several species. The myostatin gene (*MSTN*) comprises three exons and two introns. The nucleotide sequence coding for the active form of myostatin (109 a.a) is located in the 3′ terminal of the third exon (Gonzalez-Cadavid et al., 1998). Effects of abolishing myostatin function were first explored by McPherron et al. (1997) in mutant mice where the entire mature C-terminal region was deleted, showing a two- to three-fold increase in skeletal muscle mass in mutant compared to wild-type animals. Mutations at the myostatin gene, responsible for a significantly increased skeletal muscle mass, were also shown to naturally occur in several livestock species, like cattle, sheep, pigs, dogs, horses, rabbit, poultry (for a review, see Aiello et al., 2018), and human (Schuelke et al., 2004). In particular, the high level of polymorphism previously described for the myostatin gene in humans (Ferrell et al., 1999) was confirmed in cattle in a survey of 678 animals from 28 European breeds by using Single Strand Conformation Polymorphism (SSCP) analysis, followed by Sanger sequencing of the PCR re-amplified SSCP bands (Dunner et al., 2003). A total of 10 silent, 3 missense and 6 disruptive mutations were detected in the above study, giving origin to 20 distinct haplotypes whose sequence variation and breed distribution patterns supported the hypothesis that origin of muscular hypertrophy (also known in cattle as “double muscle” phenotype) was the result of both (i) European dispersal of the common variant nt821(del11) and (ii) arising and maintaining of various (mostly disruptive) mutations in single breeds.

Old World camels include both wild (*Camelus ferus*) and domestic (*Camelus dromedarius* and *Camelus bactrianus*) species. Despite differences in muscularity can be observed among distinct populations and/or individuals, these are not dramatic as those observed in other livestock species and no evident “double muscle” phenotype has been described so far (B. Faye, personal communication). The myostatin gene has been previously characterized in various dromedary populations from Pakistan and India, although only 256 bp of exon 1 and 375 bp of exon 2, respectively, were considered in the analyses (Shah et al., 2006; Agrawal et al., 2017). A more comprehensive sequence polymorphism analysis of the myostatin gene was performed in our laboratories, where more than 3.6 kb of genomic sequence, including the three exons, small part of the introns and part of the 3′ and 5′ ends, was sequenced in a total of 22 dromedaries from three different Northern African geographic regions (Muzzachi et al., 2015). In this study, to further expand the knowledge base about myostatin, we followed up by (i) characterizing the gene structure (transcriptional initiation/termination sites; exon/intron boundaries), (ii) analyzing polymorphism of the complete genomic sequence and of the partial cDNA in a set of animals from various sampling sites, (iii) investigating expression patterns at both the transcript and the protein level in different skeletal muscles.

## Materials and Methods

### Characterization of the Full-Length Myostatin cDNA

#### RNA Isolation From Skeletal Muscles

Skeletal muscles were sampled at slaughterhouses from seven different animals (Sudan, 3; Egypt, 2; Mauritania, 2). For each animal, a small sample of frozen muscle tissue (100 mg), previously stored in tubes containing RNAlater (QIAGEN), was finely chopped by using a sharp scalpel in 2 ml RLT buffer (RNeasy Midi Kit, QIAGEN) supplemented with β-mercaptoethanol following the manufacturer’s instructions. The sample was then homogenized using the T 10 basic Ultra-Turrax homogenizer (IKA). After homogenization, the sample was added with 4 ml of RNase-/DNase-free water plus 65 μl of Proteinase K (SIGMA, ≥0.6 Units/μl). After an incubation step at 55°C for 20 min, samples were processed following manufacturer’s instruction.

#### Myostatin Transcription Initiation and Termination Sites

Transcription initiation and termination sites were identified using the RACE (Rapid amplification of cDNA ends) PCR approach implemented in the SMARTer RACE cDNA Amplification Kit (CloneTech) following manufacturer’s instructions.

For the 5′RACE-PCR, the following primers were used:

• 1st reaction: 5′ATCCTCAGTAAACTTCGCCTGGAAACAGCT3′
• 2nd reaction: 5′GGCTGTGTAATGCATGTATGTGGAGACAAA3′

For the 3′RACE-PCR, the following primers were used:

• 1st reaction: 5′TGTGCACCAAGCAAACCCCAGAGGTTCGGC3′
• 2nd reaction: 5′CCTGCTGTACTCCCACAAAGATGTCTCCAA3′

After separation on a 2% agarose gel, PCR products were excised from the gel, purified using the QIAquick Gel Extraction Kit (QIAGEN) and sequenced using the following primers:

5′RACE: 5′TTTGTCTCCACATACATGCATTACACAGCC3′

3′RACE: 5′CCTGCTGTACTCCCACAAAGATGTCTCCAA3′

#### RNA Retro-Transcription

After quality control using a NanoDrop 2000C spectrophotometer (Thermo Fisher Scientific), 50 μl of total RNA was retro-transcribed into cDNA using High Capacity cDNA Reverse Transcription Kit (Thermo Fisher Scientific), which is based on a combination of oligo(dT) and random primers, following manufacturer’s instruction.

#### Amplification and Sequencing of the Myostatin cDNA

A nested PCR was developed, with external forward and reverse primers falling in the 5′UTR and 3′UTR, respectively. The primer sequences were:

• Forward 5′CCTTGGCATTACTCAAAAGCAA3′
• Reverse 5′CCTAAGTTTTCGAGCTAGGAGATC3′

The PCR conditions were: initial step at 95°C for 2 min; 35 cycles of a three-step thermal profile of 95°C for 30 s (denaturation), 57°C for 30 s (annealing), 72°C for 60 s (elongation); a final elongation step at 72°C for 5 min.

Internal primers were:

• Forward 5′CAGTACGATGTCCAGAGAGATGACAGCAGT3′
• Reverse 5′TGTGCACCAAGCAAACCCCAGAGGTTCGGC3′

The PCR conditions were: initial step at 95°C for 2 min; 35 cycles of a three-step thermal profile of 95°C for 30 s (denaturation), 61°C for 30 s (annealing), 72°C for 60 s (elongation); a final elongation step at 72°C for 5 min.

For both reactions, 3 μl of cDNA were added to a solution of 12.5 μl Master Mix (Multiplex PCR Kit, QIAGEN), 1 μl of each primer (Forward and Reverse), 7.5 μl water. After separation on a 2% agarose gel, PCR products were excised from the gel, purified using the QIAquick Gel Extraction Kit (QIAGEN) and sequenced on both directions with internal primers, using the Sanger method.

#### Sequences Alignment

Sequences obtained via Sanger method (RACE PCR and cDNA amplicons, see above) were aligned using ClustalOmega (Sievers et al., 2011).

#### Precursor Prediction

The SignalP 4.1^1^, the Combined Signal Peptide Predictor (CoSiDe)^2^ and the Signal-3L 2.0^3^ online tools were interrogated for predicting the most probable location of the signal peptide cleavage site.

### Comparative Protein Modeling

Myostatin orthologs were searched through and sampled from *Mammalia*. The crystallized structure of the myostatin was available under the PDB_ID 5ntu (Cotton et al., 2018). The retrieved sequences, including the proposed crystallized structure (5ntu.pdb), were thus aligned by using ClustalW (see Pierri et al., 2010, and references therein) for investigating chemical-physical properties of the amino acid regions showing variants between the human and the dromedary sequences. Accession numbers of the sequences considered in this study, together with additional details concerning the databases and the online tools used in this study, are summarized in Supplementary Table S1.

Then, SPDBV was used for generating a 3D model of the dromedary myostatin protein according to protocols described in Pierri et al. (2010). The obtained 3D comparative model was energetically minimized. A total of 100 steps of energy minimization were performed for relaxing the obtained 3D model by using the energy minimization tools implemented in Chimera. WhatIF and Chimera biochemical tools were used for checking the correct 3D model packing. PyMOL was used for manual inspection of the investigated 3D models and for generating figures (see Pierri et al., 2010, and references therein).

### Phylogenetic Analysis

The analysis of the evolutionary relationships among orthologous myostatin sequences was conducted using MEGA5 (Tamura et al., 2011). Orthologous sequences of myostatin/growth differentiation factor 8 with *E*-value lower than 10ˆ-55, query coverage higher than 70% and % of identical amino acids ranging between 40 and 100% were aligned by using ClustalW implemented in Jalview. For Arthropoda, Aves, and Mammalia, due to the existence of more than one hundred of sequences complying with the above criteria, we imposed a filter on the first 30 sequences for each taxonomic group. Redundant sequences with 100% identical amino acids were removed from the multiple sequence alignment. A final set of 83 protein sequences (see Supplementary Data Sheet S1) were retained for tree building. In detail, the tree was built from the ungapped multiple sequence alignment applying the maximum likelihood method with the JTT model for the amino acid substitutions and a gamma distribution (five discrete gamma categories) for the rates among sites. A total of 100 bootstrap samplings were applied to test the robustness of the tree.

### Polymorphism Analysis

#### Sample Collection and Whole-Genome Sequencing

Whole blood from 25 Old World camels was collected during routine veterinary procedures or as part of a monitoring program of the wild camel population in Mongolia. These samples included nine dromedaries (*C. dromedarius*), seven domestic Bactrian camels (*C. bactrianus*), and nine wild camels (*C. ferus*). Dromedary camels were selected to represent a variety of geographic locations: Pakistan (1), Kenya (1), Kingdom of Saudi Arabia (3), Sudan (1), United Arab Emirates (1), Qatar (1), Canary Islands – Spain (1). Domestic Bactrian camel originated from Mongolia or Kazakhstan, while all the wild camels originated from Mongolia. DNA was extracted using the Master PureTM DNA purification kit for blood (Epicentre version III) and generated a 500 bp paired-end library for each sample. Each library was sequenced with a single lane of an Illumina HiSeq (Illumina, United States) according to standard protocols.

#### Whole-Genome Read Processing and Alignments

The 3′ end of sequence reads were trimmed to a minimum phred-scaled base quality score of 20 (probability of error < 1.0%) and trimmed reads < 50 bp in length were excluded using POPOOLATION v1.2.2 (Kofler et al., 2011). All processed reads were aligned to the *C. ferus* CB1 reference genome (Genbank accession: AGVR01040332.1) using BWA v0.6.2 (Li and Durbin, 2009) with parameters ‘-n 0.01 -o 1 -e 12 -d 12 -l 32.’ Duplicate reads were removed and alignments were filtered to only include reads that were properly paired and unambiguously mapped with a mapping quality score > 20. Reads around insertions/deletions were realigned and a base quality score recalibration was performed using the Genome Analysis Toolkit (GATK) v3.1-1 following guidelines presented by Van der Auwera et al. (2013). As input into the base quality score recalibration step, a stringently filtered set of single nucleotide variants (SNVs) was generated using the overlap of three different variant-calling algorithms [SAMTOOLS v1.1] (Li et al., 2009); [GATK HAPLOTYPECALLER v3.1-1] (Van der Auwera et al., 2013); [ANGSD v0.563] (Korneliussen et al., 2014). The overlapping SNVs were filtered to exclude those with a quality score (Q) < 20, depth of coverage (DP) > 750 (∼30X/individual), quality by depth (QD) < 2.0, strand bias (FS) > 60.0, mapping quality (MQ) < 40.0, inbreeding coefficient < -0.8, mapping quality rank sum test (MQRankSum) < -12.5, and read position bias (ReadPosRankSum) < -8.0. Furthermore, SNVs were excluded if three or more were found within a 20 bp window, were within 10 bp of an insertion/deletion, or were found in an annotated repetitive region.

#### Whole-Genome Variant Identification

Another set of SNVs from the realigned and recalibrated alignment files was generated using the GATK HAPLOTYPECALLER and filtering criteria as described above. SNVs on scaffolds putatively assigned to the X and Y chromosome, with a minimum allele count < 2, missing a genotype in more than five individuals, with 4 > DP > 30 per genotype, and deviating from Hardy–Weinberg equilibrium (*p* < 0.0001) in VCFTOOLS v0.1.12b (Danecek et al., 2011) were further excluded. This set of SNVs was used as a training set to perform variant quality score recalibration in GATK, assigning a probability of error to the training set of 0.1. This recalibration develops a Gaussian mixture model across the various annotations in the high-quality training dataset then applies the model to all variants in the initial dataset. The process has been shown to outperform the ‘hard’ filtering of variants (e.g., Pirooznia et al., 2014). After variant recalibration, all SNVs with LOD score < -5.0 and 4 > DP > 30 per genotype were excluded.

#### Identification and Characterization of Variants at the Myostatin Locus

The publicly available *Camelus ferus* myostatin sequence (GenBank Accession No AGVR01040332) was BLASTed against our Old World camel genomes and the contig-8645394 (*Camelus dromedarius*), contig-8938518 (*Camelus bactrianus*) and contig-7907533 (*Camelus ferus*) were retrieved and used in the comparative analysis of the myostatin locus at the nucleotide level. Moreover, from the final set of SNVs described in the sub-section above, the *Camelus dromedarius* Single Nucleotide Polymorphisms (SNPs) falling in the contig-8645394 (Supplementary Data Sheet S2) were selected for further inspection.

#### Bayesian Clustering

The identified SNPs were used for clustering the nine dromedary samples by adopting the Bayesian algorithm implemented in the STRUCTURE software v. 2.2 (Falush et al., 2007). The analysis was performed without providing *a priori* information on population membership, adopting the “admixture model” option and a burn-in period of 10,000 generations, followed by 100,000 iterations. Five independent runs were performed for each *K* value (number of clusters to be tested), and the results were visually inspected for reproducibility. *K* values ranging from 1 to 9 were tested, and the *K* value showing the highest probability was discussed.

#### *In silico* Functional Prediction

The web-based analysis tool by the Human Splicing Finder Version 3.0.2 (Desmet et al., 2009), available at http://www.umd.be/HSF3/index.html, was used to predict putative functional effect of SNP variants in terms of potential alteration of splicing patterns. The *in silico* tool TFBIND (Tsunoda and Takagi, 1999), available at http://tfbind.hgc.jp/, was used to identify transcription factor binding sites (TFBS) and their possible disruption due to the presence of Single Nucleotide Polymorphisms.

### Absolute Quantification of Myostatin Transcripts

#### RNA Isolation and cDNA Synthesis

Skeletal muscles were sampled at slaughterhouses (Kingdom of Saudi Arabia) from two adult animals. For each animal, eight muscles, representative of the different anatomical regions of the body were taken: *brachiocephalicus* (head/neck), *deltoid, extensor carpi radialis*, and *tensor fasciae latae* (forelimbs), *semitendinosus* and *coccygeus* (trunk), *biceps femoris*, and *peroneus longus* (hindlimbs). For all muscles, sampling occurred within 30 min *post-mortem*. Immediately after collection, samples were stored in tubes containing RNAlater (QIAGEN). For RNA isolation and cDNA synthesis, the procedures described above were adopted.

#### Digital Droplet PCR Assay Design

The Digital Droplet PCR method is based on end-point fluorescence signal detection, and the intensity of signal observed for positive droplets, varying with primer/template combination, is not considered for target quantification. However, in this system, droplets are interpreted as either “positive” or “negative,” depending whether target amplification occurred or not, based on a settled fluorescence cut-off. Two different assays, with probes targeting the two possible exon junctions in the myostatin gene (between exon 1 and 2, and between exon 2 and 3), were used. FAM- (6-carboxy-fluorescein) and HEX- (hexa-chloro-fluorescein) labeled probes were used, respectively, in Assay 1 and Assay 2. Probes and primers sequences were as follows:

• Assay 1:
  Probe 1 5′-/56-FAM/CTACAGAGT/ZEN/CTGATCTTCTAATGC/3IABkFQ/-3′
  Primer 1 5′-GACGGAAACAATCATTACC-3′
  Primer 2 5′-GAGCTAAACTTAAAGAAGCAA-3′
• Assay 2:
  Probe 1 5′-/5HEX/AAGGGATTC/ZEN/AAACCATCTTCTC/3IABkFQ/-3′
  Primer 1 5′-GGTCATGATCTTGCTGTA-3′
  Primer 2 5′-GTCTGTTACCTTGACTTCTA-3′

By partitioning the reaction volume into thousands of droplets, this technique allows absolute quantification of nucleic acids without the need of a standard curve, with improved precision over classical quantitative PCR (qPCR).

#### Digital Droplet PCR Conditions

A 20-μl reaction mixture was prepared comprising of 10 μl ddPCR Supermix^TM^ for probes (no dUTP) (Bio-Rad), 1 μl primers and probe mix for Assay 1, 1 μl primers and probe mix for Assay 2, 2 μl cDNA, 6 μl RNase-/DNase-free water. The final concentration of primers and probe was 900 and 250 nM, respectively. The amplification conditions were 10 min DNA polymerase activation at 95°C, followed by 40 cycles of a two-step thermal profile of 30 s at 94°C for denaturation, and 60 s at 60°C for annealing and extension, followed by a final hold of 10 min at 98°C for droplet stabilization, and cooling to 4°C. A thermal cycler (T100^TM^; Bio-Rad) was used, and the temperature ramp rate was set to 2°C/s, with the lid heated to 105°C, according to the Bio-Rad recommendations. A negative (no template) and a positive control were included. The latter consisted, for both assays, of a synthetic oligonucleotide (gBlocks Gene Fragment, by IDT), with a size of 467 bp, including junctions between exons 1 and 2 and between exons 2 and 3, designed based on the predicted sequence for the myostatin transcript in *Camelus dromedarius* (XM_010991955). In the reaction preparation, for the positive control, 2 μl of the above synthetic oligonucleotide were added, at a final concentration of 1 ng/ml. For all the considered muscles, two biological and two technical replicates were included in the experiment.

#### Data Analysis

After the thermal cycling, the plates were transferred to a droplet reader (QX200^TM^; Bio-Rad). The software package provided with the ddPCR system was used for data acquisition (QuantaSoft^TM^ 1.6.6.0320; Bio-Rad). The rejection criterium for the exclusion of a reaction from subsequent analysis was a low number of droplets measured (<10,000 per 20 μl PCR). The data from the ddPCR are given in target copies/μl reaction. The significance of differences among muscles was tested using ANOVA (Analysis of Variance).

### Protein Extraction and Western Blotting

A small sample of frozen muscle tissue (100 mg), previously stored in tubes containing RNAlater (QIAGEN), was finely chopped and homogenized by using a sharp scalpel in 300 μl Ripa buffer [10 mM Tris-HCl pH 7.4, 140 mM NaCl, 1% (v/v) Triton X-100, 1% (w/v) Na-deoxycholate, 0.1% (v/v) SDS, 1 mM NaF, 1 mM EDTA, 1 mM Na_3_VO_4_] supplemented with 1x protease inhibitor cocktail (Sigma), and then by using the T 10 basic Ultra-Turrax homogenizer (IKA). After homogenization, the sample was kept on ice for 30 min, and then vortexed for 5 min. At the end, sample was centrifuged for 20 min at 4°C at 13,000 × g to remove unbroken cells, nuclei and cell debris. The supernatant, containing solubilized proteins, was recovered and protein concentration was measured by the method of Bradford (Bradford, 1976). An aliquot of 20 μg of protein for each sample was diluted in Laemmli buffer not containing DTT or β-mercaptoethanol, heated at 95°C for 5 min, and separated by 12 % (v/v) Tris/HCl SDS/PAGE. The separated proteins were transferred to Immobilon P (Millipore) in Trans-Blot semidry electrophoretic transfer cell (Amersham Biosciences) for immunoblotting. The used primary antibody was a rabbit polyclonal anti-MSTN antibody against the C-terminal region (300–349 aa) of mouse myostatin (TA343358, OriGene; dilution 1:1000) that presented broad species reactivity, including artiodactyls. The densitometric quantification and image processing of the considered bands were carried out using Adobe Photoshop and the Image software package (version 1.61, National Institutes of Health, Bethesda, MD, United States). The total lane density of transferred proteins on the membrane stained with Coomassie Blue dye was used for the normalization of the proteins of our interest. The significance of differences among muscles, for each considered band, was tested using ANOVA (Analysis of Variance). *Post hoc t*-tests were performed to determine where the groups differed. All *p*-levels for *post hoc t*-tests were adjusted using Bonferroni correction.

## Results

### Myostatin Gene Organization

**FIGURE 1:**
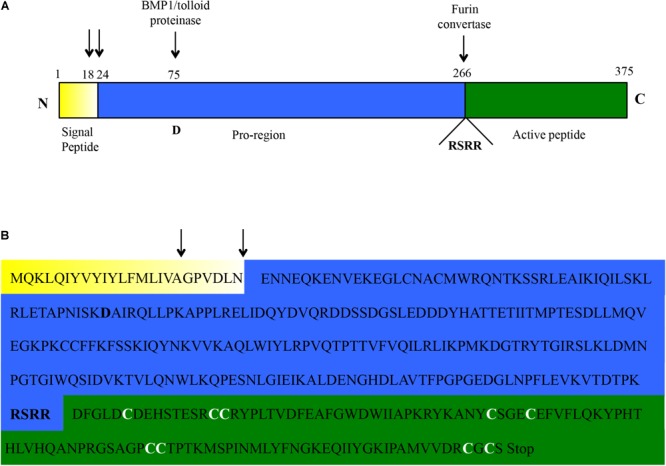
Amino acidic sequence of the *C. dromedarius* myostatin as inferred from the cDNA sequence. **(A)** Schematic outline. The three protein domains (signal peptide, pro-region, and active peptide) are highlighted in different colors (yellow, blue, and green, respectively). The two most likely residues involved in the signal peptide cleavage (see main text) are indicated by black arrows. Similarly, the residue (D, for aspartic acid) shown to be essential for BMP/tolloid protease cleavage, and the motif (RSRR) needed for recognition by furin convertase, are highlighted. **(B)** Amino acidic sequence of the *C. dromedarius* myostatin, with the three protein domains highlighted in different colors, as in **(A)**. The above mentioned hallmarks are also depicted here (signal peptide cleavage, black arrows; BMP/tolloid protease cleavage residue and furin convertase recognition motif, bold). In addition, the nine conserved cysteine residues in the active peptide are indicated (bold and white).

The RACE-PCR approach allowed to map the start transcription site (Supplementary Figure S1A) at 109 bp upstream the start-codon, in a position that is 24 and 25 bp downstream compared to the usual human and mouse transcription initiation sites, respectively^4^. The transcriptional termination site (Supplementary Figure S1B) was mapped 215 bp downstream the stop-codon, much earlier than in human and mouse where a 1561 and 1448 bp 3′UTR is usually reported^4^. No evidence was found, by combined RT-PCR and RACE approaches, for alternative splicing events or alternative 5′ or 3′ ends (Supplementary Figure S2). Based on the above, a 5292 bp genomic locus was identified for myostatin in *C. dromedarius*. The locus was highly conserved among the three Old World camelids species (Supplementary Figure S3). Comparative analysis of the *C. dromedarius* genomic sequence (contig-8645394) with the obtained cDNA sequences confirmed, as in other species, the presence of three exons and two introns, with a predicted *C. dromedarius* myostatin full length cDNA of 1452 bp (Supplementary Figure S4A) and a protein of 375 amino acids (Figure 1). The latter is consistent with the predicted protein size for most of the species^5^. The dromedary myostatin protein also showed all the hallmarks present in other TGF-ß family members, including an N-terminal signal sequence for secretion, a pro-region followed by the proteolytic processing RSRR site, and a C-terminal domain containing nine cysteine residues. In particular, the signal peptide was consistently predicted to have an 18 amino acid length by SignalP 4.1 and Signal-3L 2.0, while a length of 23 amino acids was predicted by CoSiDe.

### Comparative 3D Protein Modeling

**FIGURE 2:**
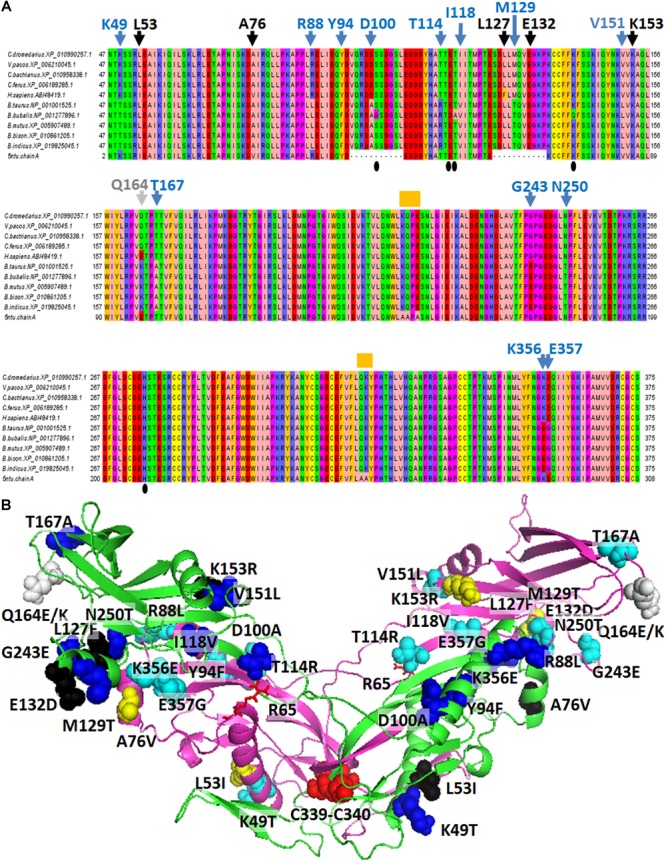
Comparative analysis of myostatin protein sequences. **(A)** The alignment of myostatin orthologous sequences sampled from various mammalian species is presented. Blue arrowheads indicate the variants detected between the four considered *Camelidae* sequences and the five Bovidae sequences at 13 specific sites. Black arrowheads indicate the variants detected between the four considered *Camelidae* sequences and the *Homo sapiens* sequence at 5 specific sites, different from the sites previously cited. The gray arrowhead indicates the position of two different variants detected in *H. sapiens* and in Bovidae in correspondence of Q164 from *C. dromedarius*. Orange “boxes” indicate variations between the *H. sapiens* sequence retrieved from refseq_database and the sequence of the human crystallized myostatin. Amino acid codes and numbering refers to the *C. dromedarius* myostatin. **(B)** Lateral view of the 3D comparative model of the *C. dromedarius* myostatin dimer. The protein is reported in green/magenta cartoon representation. Variants observed between *Camelidae* myostatin sequences and human/Bovidae myostatin are reported in black (5)/blue (13) spheres in chain A, and dark-yellow (5)/cyan (13) spheres in chain B, respectively. The only site of *C. dromedarius* myostatin showing a variation both in *H. sapiens* and in Bovidae locates at site 164 (Q164 for *C. dromedarius*, E164 in *H. sapiens*, K164 in *B. taurus*) and is indicated by gray spheres. Notably, variants observed between *C. dromedarius* and Bovidae occur at different sites with respect to those detected between *C. dromedarius* and *H. sapiens*, with the exclusion of residues at site 164. Residues C339/C340 of chain A and chain B, forming inter-monomer disulphide bridges, are reported in red spheres. R65 of chain A and chain B, involved in interactions with T114, is indicated by red sticks.

Sequences from nine *Artiodactyla* species, including the three phylogenetically close Old World camelids (*C. dromedarius, C. bactrianus*, and *C. ferus*), one New World camelid (*Vicugna pacos*), the two *Bos taurus* subspecies, i.e., *B. taurus taurus* (a non “double muscle” Hereford subject) and *B. taurus indicus*, the wild yak (*Bos mutus*), the buffalo (*Bubalus bubalis*) and the bison (*Bison bison*) were aligned with the human myostatin sequence (Accession no. ABI48419.1), and the human myostatin C-terminal domain solved by X-ray diffraction (residues 46-375 out of 375) (Figure 2A). *C. dromedarius, C. ferus, C. bactrianus*, and *V. pacos* share 100% of identical amino acids. Six, or fourteen, variants are observed among the above cited *Camelidae* sequences and the human myostatin sequence (Accession No. ABI48419.1), or the corresponding taurine myostatin sequence, respectively (Figure 2A). Out of them, 5 in the contrast with the human sequence and 13 in the contrast with the taurine sequence are variants occurring at different sites, while one, at position 164, presented different variants when contrasted with the human and the cattle sequence, respectively. In addition, it is possible to observe that the 6 variants detected in the contrast with the human sequence are conservative (Figure 2A), while 9, out of the 14 variants detected in the contrast with the cattle sequence are not conservative (Figure 2A). No variants were observed when contrasting among them the sequences from the five species belonging to the *Bovidae* family. A notable exception was *B. bubalis*, for which variants where observed at positions 101, 117 and 141. In all the above cases, the nucleotides observed in *B. bubalis* were different from those observed in all the other eight sequences. Figure 2B presents the 3D comparative model of the *C. dromedarius* myostatin dimer, and highlights the variants observed between the *Camelidae* myostatin sequences and the human/bovine myostatin.

### Evolutionary Relationships Among Myostatin Proteins

**FIGURE 3:**
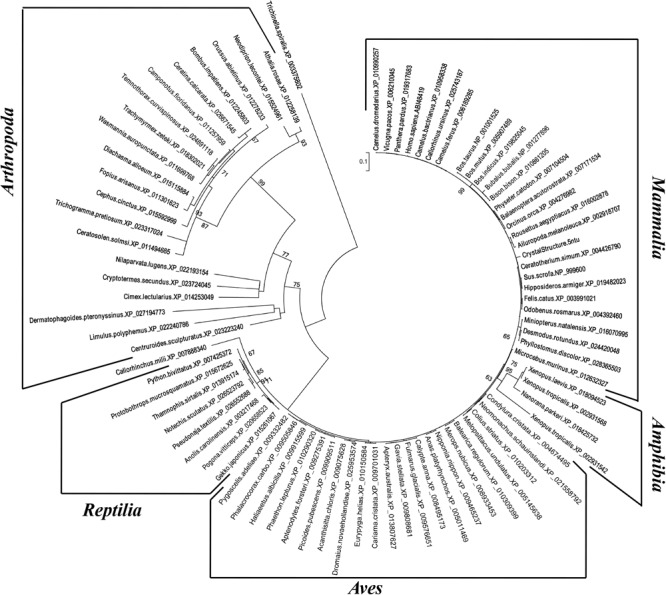
Phylogenetic analysis of myostatin protein sequences. Numbers indicate bootstrap values higher than 60/100.

The inferred maximum likelihood tree of myostatin protein sequences (Figure 3) highlighted the presence of three supported clusters (bootstrap value higher than 60%), corresponding to Arthropoda, Reptilia and Amphibia, that may reflect a different attitude in the regulation of skeletal muscle growth in the different taxonomic groups. Interestingly, within Mammalian sequences, the highest bootstrap value (99%) was observed for the cluster grouping myostatin sequences belonging to the Bovidae family.

### Polymorphism Analysis and *in silico* Functional Prediction

The results of the sequence polymorphism analysis for the myostatin locus are presented in Table 1. As can be observed, only three polymorphisms, two transitions (A66460G and T66461C) and one transversion (G66148C), were identified inside the myostatin gene, all located deep in the intron 1. On the other side, a total of 45 and 21 variant sites were identified in our study in the upstream and downstream regions, respectively, with an overall average density of one SNP every about 1.5 kb. In order to interpret the observed patterns of SNP distribution across samples from different countries, we performed a Bayesian clustering analysis of the nine dromedary samples using the 69 identified SNPs. At *K* = 7 (Supplementary Figure S5), the analysis highlighted that the sample from Pakistan was well differentiated, and the same occurred for a pair of samples, one from Kingdom of Saudi Arabia and one from United Arab Emirates, respectively. The rest of the samples were clearly “admixed,” suggesting that most of the considered SNPs do not follow a geographic pattern. Putative variants in the myostatin coding region, inferred from aligning previously published myostatin sequences with sequences generated in this study, are presented, for completeness, in Supplementary Figures S4A,B.

**Table 1:** Single Nucleotide Polymorphisms identified in the considered population sample (nine *C. dromedarius* animals from seven countries).

Target region	Size (bp)	Polymorphism
Upstream	64736	G13066T; T13901A°; C14568A°; T14745G; C16352T; G16640A°; G17867A; T25076C°; T29288A; G31182A; G31464A; C32869T^∘∗^; C33308G; G33597C; G34295T; T34901C^∘∗^; G35625A^∘∗^; C35782T°; G38778A; T40104C^∘∗^; T41149C^∗^; T42365A; C43439A°; C47473T; C48225T; G48931T^∘∗^; C49083T^∗^; G49630C^∘∗^; T50414C; T50514G^∗^; A50637C°; T50897C^∗^; T52618C°; G52669T; T53222C^∘∗^; G53398A^∘∗^; C55949T^∘∗^; A56391G; G57208A^∘∗^; T58686C^∘∗^; G59091T^∘∗^; A59187T^∗^; A60587G; T63437C; A64026G^∘∗^
5′ UTR	109	None
Exon 1	373	None
Intron 1	1801	G66148C°; A66460G^∗^; T66461C^∗^
Exon 2	374	None
Intron 2	2039	None^#^
Exon 3	381	None
3′ UTR	215	None
Downstream	21956	A72141T; A72526G; A73136C; G75845A^∘∗^; T75862C; G76270A^∘∗^; T77354C^∘∗^; T78572A; G78774A°; G78993A^∗^; T80531G; C81364T°; C81499T°; G83533C; T86423C°; T87060A°; G89595A°; A89886T; C90627A°; A90686T; T90834C°

*Variant sites are presented based on the region where they are located (either in the exons or introns of the myostatin locus, in the 5′ or in the 3′ UTR, or in the upstream/downstream regions). Site positions refer to the *C. dromedarius* contig-8645394. Circles (°) indicates those SNPs that could also be observed when aligning the contig-8645394 with the publicly available contig4726 (Accession No. JDVD01004726.1). Asterisks (^∗^) indicates those SNPs that could also be observed when aligning the contig-8645394 with the publicly available contig_13989_126 (Accession No. LSZX01094446.1). The ^#^ (hash) symbol indicates the only SNP, located in the myostatin gene (intron 2), that was not observed in our study but was evident when aligning the contig-8645394 with both contig4726 and contig_13989_126.*

Analysis by Human Splicing Finder highlighted, for the intronic polymorphisms, a potential role in alteration of splicing for G66148C, predicted to break an ESE (exonic splicing enhancer) site (Supplementary Figure S6A), A66460G, predicted to generate a new donor site and a new ESS (exonic splicing silencer) site (Supplementary Figure S6B), while no significant splicing motif alteration was detected for T66461C (Supplementary Figure S6C). TFBS analysis, performed for each SNP using the two input sequences harboring the alternative alleles, highlighted the presence of disrupted TFBSs for all the three loci (Table 2). Moreover, we analyzed the potential transcriptional factor binding sites in the DNA sequence of 8 kb of the *C. dromedarius* myostatin gene upstream region (included in the contig-8645394). A total of 10677 putative binding sites were identified (Supplementary Data Sheet S3). A graphical outline of the most significant predicted regulatory motifs in the 1.5 kb proximal to the transcription initiation site of the *C. dromedarius* myostatin gene is presented in Supplementary Figure S7. In addition, in this region two SNPs were present (T63437C and A64026G) (Table 1), out of which the latter was also observed as being polymorphic by aligning the contig-8645394 (Supplementary Data Sheet S2) with the publicly available contig4726 (Accession No. JDVD01004726.1) and contig_13989_126 (Accession No. LSZX01094446.1). TFBS analysis, repeated for each SNP using the two input sequences harboring the alternative alleles, suggested the disruption of one (NFKB_Q6) and three (COUP_01, MYB_Q6, and T3R_01) transcription factors binding sites for loci T63437C and A64026G, respectively (data not shown).

**Table 2:** Results of the TFBS analysis for the three intronic SNPs detected in this study.

SNP	AC	ID	Score	Strand	Consensus	Signal
**A66460G**	**Allele A**					
	M00103	V$CLOX_01	0.778624	(+)	NNTATCGATTANYNW	GGTATTAATTAGCTG
	M00104	V$CDPCR1_01	0.790948	(-)	NATCGATCGS	GGTATTAATT
	M00134	V$HNF4_01	0.769283	(-)	NNNRGGNCAAAGKTCANNN	ATTTAAATTTTGGTATTAA
	**Allele G**					
	M00211	V$PADS_C	0.825857	(+)	NGTGGTCTC	TTTGGTGTT
	M00212	V$POLY_C	0.787056	(+)	CAATAAAACCYYYYKCTN	CATTTAAATTTTGGTGTT
	M00279	V$MIF1_01	0.741931	(-)	NNGTTGCWWGGYAACNGS	GGTGTTAATTAGCTGCTA
	M00280	V$RFX1_01	0.776836	(-)	NNGTNRCNWRGYAACNN	GTGTTAATTAGCTGCTA
**G66148C**	**Allele G**					
	M00143	V$PAX5_01	0.787091	(+)	NCNNNRNKCANNGNWGNRKRGCSRSNNN	GAGACAGGCACCTTAACAGAGAAGGCAT
	**Allele C**					
	M00262	V$STAF_01	0.767919	(+)	NTTWCCCANMATGCAYYRCGNY	TTAACACAGAAGGCATGACAAG
**T66461C**	**Allele T**					
	M00103	V$CLOX_01	0.778624	(+)	NNTATCGATTANYNW	GGTATTAATTAGCTG
	M00104	V$CDPCR1_01	0.790948	(-)	NATCGATCGS	GGTATTAATT
	M00252	V$TATA_01	0.829231	(+)	STATAAAWRNNNNNN	GTATTAATTAGCTGC
	**Allele C**					
	M00185	V$NFY_Q6	0.779548	(-)	TRRCCAATSRN	CTAATTAGCTG

*AC, transcription factor matrix code. ID, transcription factor label, with V meaning “vertebrate.” SCORE, similarity (0.0–1.0) between a registered sequence for the transcription factor binding sites and the input sequence. STRAND, strandness. + and - means forward and reverse strands that the transcription factor binds, respectively. CONSENSUS, consensus sequence (fixed) of the transcription factor binding sites. S = C or G, W = A or T, R = A or G, Y = C or T, K = G or T, M = A or C, N = any base pair. SIGNAL, sub-sequence from the input sequence at the position corresponding to the consensus sequence. Default cut-offs by Tsunoda and Takagi (1999) were adopted. The analysis was performed, for each SNP, using the two input sequences harboring the alternative alleles.*

### Quantitative Myostatin Transcript Analysis in Dromedary Skeletal Muscles

**FIGURE 4:**
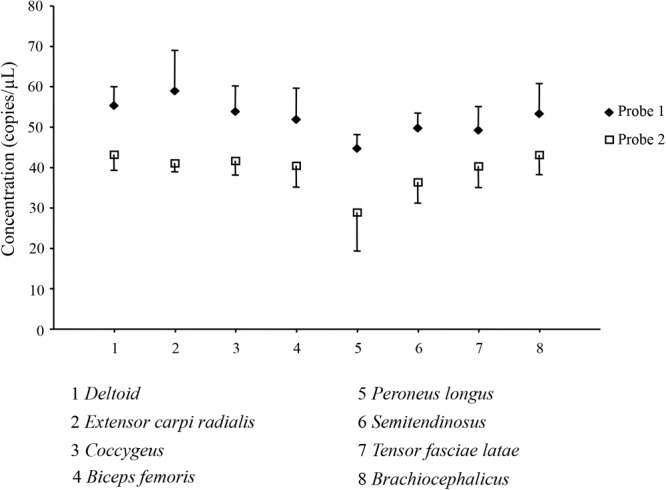
Absolute quantification of the myostatin transcript in skeletal muscles. Results of the Digital Droplet PCR analysis for the eight considered dromedary muscles (1, *deltoid*; 2, *extensor carpi radialis*; 3, *coccygeus*; 4, *biceps femoris*; 5, *peroneus longus*; 6, *semitendinosus*; 7, *tensor fasciae latae*; 8, *braciocephalicus*) and for the two used probes (u, Probe 1; ◆, □ Probe 2) are presented as mean ± SD of the four replicates.

We investigated the quantitative expression of myostatin transcripts in eight dromedary skeletal muscles (*deltoid, extensor carpi radialis, coccygeus, biceps femoris, peroneus longus, semitendinosus, tensor fasciae latae, brachiocephalicus*) by Digital Droplet PCR. Two different assays, with probes targeting the two possible exon junctions in the myostatin gene, were used. Number of droplets generated in each experiment replicate, together with plot of raw data, are shown in Supplementary Figure S8. All the replicates passed the cut-off value (>10,000 droplets). Figure 4 presents the results of the absolute quantification experiments for the eight muscles, and for the two probes, expressed as target copies/μl. The FAM-labeled probe (probe 1, targeting the junction between exon 1 and 2) produced systematically higher values compared to the HEX-labeled probe (probe 2, targeting the junction between exon 2 and 3). However, trends among different muscles were similar for both probes, as also supported by a correlation coefficient higher than 0.78 (data not shown). Analysis of variance among means of different muscles did not find significant difference for any of the two assays.

### Myostatin Protein Expression in Dromedary Skeletal Muscles

**FIGURE 5:**
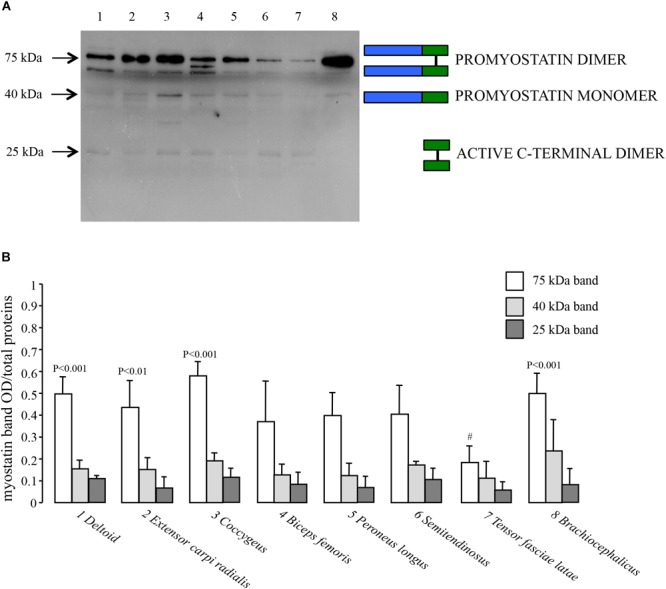
Myostatin protein expression. **(A)** Representative Western Blot of the eight considered dromedary muscles (1, *deltoid*; 2, *extensor carpi radialis*; 3, *coccygeus*; 4, *biceps femoris*; 5, *peroneus longus*; 6, *semitendinosus*; 7, *tensor fasciae latae*; 8, *brachiocephalicus*) performed using a rabbit polyclonal antibody (1:1000 dilution) that specifically binds the carboxy-terminal domain (Origene, TA343358). **(B)** Western Blot densitometric analysis of the promyostatin dimer (75 kDa), the promyostatin monomer (40 kDa) and the active C-terminal dimer (25 kDa), respectively. For each lane, optical density (OD) of the considered band is presented as a ratio over the total density of all the proteins transferred on the membrane and stained with Coomassie blue.

We investigated the expression of myostatin at the protein level on the same set of dromedary skeletal muscles previously described for quantitative transcript analysis. In order to detect the active C-terminal myostatin dimer, we performed Western Blot analyses using a polyclonal antibody raised against the C-terminus. Moreover, protein electrophoretic separation was run under non-reducing conditions in order to preserve the integrity of the disulphide bonds in the C-terminal domain. As shown in Figure 5A, a major band is present at an apparent molecular mass of 75 kDa, corresponding to the expected mass for the promyostatin dimer. Additional, weaker bands were observed at around 40 and 25 kDa, corresponding to the promyostatin monomer and the active C-terminal myostatin dimer, respectively. Densitometric analysis of Western Blots, performed on five different protein extracts for each muscle, highlighted significant differences (ANOVA, *p* = 0.0001) among muscle types for the promyostatin dimer (Figure 5B), while no significant difference was observed for both the promyostatin monomer and the active C-terminal myostatin dimer, respectively (Figure 5B). *Post hoc* tests highlighted four significant (*p* < 0.0017) pair-wise comparisons, all of them involving the *tensor fasciae latae* muscle (*vs. deltoid, extensor carpi radialis, coccygeus, and brachiocephalicus*).

## Discussion

### Myostatin Gene Organization and Protein Comparative Modeling

The myostatin gene has been largely studied in several livestock and model species. Very recently, the gene has been mapped to chromosome 5 in *Camelus dromedarius* (Elbers et al., 2019). By combining experimental and *in silico* approaches, we here describe, for the first time, the gene organization and the protein structure in the one-humped Old World camelid species. We confirmed the major features observed in other species for the myostatin gene. As expected, a high sequence similarity was observed among our experimentally obtained transcript sequence for *C. dromedarius* myostatin and the publicly available predicted sequences for the other two species within the *Camelus* genus, in line with the relatively recent divergence times between them, estimated in 5–8 mya between one-humped and two-humped domestic camels (Wu et al., 2014), and about 0.7 million years ago between *C. bactrianus* and *C. ferus* (Ji et al., 2009). Also, myostatin orthologs showed a high percentage of identical amino acids through *Mammalia*, which may explain the low bootstrap values observed in the maximum likelihood tree. A notable exception was represented by the Bovidae sequences, that clustered with high bootstrap values. Peculiar selection constraints may have played a role in shaping the evolutionary history of myostatin in Bovidae. Indeed, besides the well documented human-mediated positive selection experienced in recent times by the *Bos taurus* myostatin gene, particularly in specialized beef breeds, evidence for a more remote action of positive selection on this gene, operating during the time of divergence of Bovinae and Antilopinae, has been produced by Tellgren et al. (2004) in a systematic analysis of myostatin sequence evolution in ruminants. These periods of positive selective pressure on myostatin may correlate with changes in skeletal muscle mass. In fact, the early bovid fossil record, dating back to around 17 million years ago, had a body mass estimate of only around 20 kg. Hence, the hypothesis is that selective pressures on myostatin drove this increase in body mass coupled to an increase in skeletal muscle mass, in turn driven by ecological changes in the environments of the various species. In a recent paper, the phylogenetic relationships between camelids and other mammalian species were investigated using whole-genome sequence data (Wu et al., 2014). The authors report estimated divergence time between camelids and cattle lower than those between other mammals. This seeming discrepancy with our results may arise from the fact that single gene phylogenies could not reflect the complex evolutionary history of a whole genome and they could be less reliable in inferring genome-scale events.

Myostatin protein consists of a non-covalently held complex of the N-terminal propeptide and a disulfide-linked dimer of C-terminal fragments (Lee and McPherron, 2001). Variants detected respectively in *B. taurus* and *H. sapiens*, in correspondence of the *C. dromedarius* K49 and L53 may be involved in the stability of the myostatin dimer due to their location close to the disulphide bonds occurring among C339 and C340 residues at the myostatin dimer interface (Jiang et al., 2004). Notably, the myostatin N-terminal domain contains a region (residues 49–67) highly similar to the key latency-determining regions of the TGF-β superfamily (Walton et al., 2010). Thus, it is expected that variations observed in our comparative analysis at this region, above all in correspondence of the *C. dromedarius* K49 (Thr in *B. taurus* myostatin, Lys in TGF-β1) and L53 (Ile in *H. sapiens* myostatin, Leu in TGF-β1) may contribute toward its latency, according to Walton et al. (2010). More in general, all the cited variants locate at the monomer/monomer interface. Notably, we observed, in *Camelidae* myostatin sequences, not conservative substitutions compared to the *B. taurus taurus* myostatin sequence at position 49 (Lys in *C. dromedarius*; Thr in *B. taurus*), 88 (Arg in *C. dromedarius*; Leu in *B. taurus*), 100 (Asp in *C. dromedarius*; Ala in *B. taurus*),114 (Thr in *C. dromedarius*; Arg in *B. taurus*), 129 (Met in *C. dromedarius*, Thr in *B. taurus*), 167 (Thr in *C. dromedarius*, Ala in *B. taurus*), 243 (Gly in *C. dromedarius*; Glu in *B. taurus*), 356 (Lys in *C. dromedarius*; Glu in *B. taurus*), 357 (Glu in *C. dromedarius*, Gly in *B. taurus*) that may produce a different charge network in myostatin dimer, favoring different monomer/monomer interactions. In particular, among the described variants, it is worth noting that the residue at site 114 forms intra-chain binding interactions with Y111 and H112 and inter-chain binding interactions with R65. R65, Y111 and H112 together with K153 (R153 in *H. sapiens*) were already described as “fastener” residues associated with muscle- and obesity-related phenotypes (Gonzalez-Freire et al., 2010; Santiago et al., 2011; Bhatt et al., 2012; Garatachea et al., 2013; Szlama et al., 2015). Furthermore, variations of the residue at site 100 may influence the release of the active form from the myostatin propeptide complex due to its location close to the myostatin propeptide TLD cleavage target site (consisting of the dipeptide 98-RD-99) (Szlama et al., 2013).

### Genetic Sequence Polymorphism and Functional Prediction

Unexpectedly, the Next Generation Sequencing (NGS) of the whole myostatin locus in nine dromedaries from a variety of geographic locations in Asia, Africa and Europe did not allow the identification of novel intra-genic variants and only confirmed the presence of the three SNPs in intron 1 previously identified by Muzzachi et al. (2015) via Sanger-sequencing of a reduced portion of the gene on a different set of Northern African dromedaries. This result seems to support the hypothesis, formulated by Muzzachi et al. (2015) that the low diversity observed at the myostatin locus in *Camelus dromedarius* may reflect the peculiar evolutionary history of this species, which likely developed as domesticates from a low variable wild ancestor population.

Evidence about the existence of functional variants located in introns is growingly accumulating (Lee et al., 2015; Mou et al., 2015; Hong et al., 2018; Ostrovsky et al., 2018), not only restricted to exon–intron boundaries but also in deep intronic regions (Mendes de Almeida et al., 2017; Vaz-Drago et al., 2017). The three intronic SNPs detected in this study were *in silico* predicted to have the potential of altering both splicing and TFBS, thus suggesting they may play a role in myostatin processing and/or regulation. TFBS analysis in the myostatin upstream region allowed to predict several potential transcriptional factor binding sites, mainly belonging to the large family of dimerizing transcription factors harboring a basic helix-loop-helix (bHLH) structural motif, such as MYOD (myogenic differentiation), MYOG (myogenin), MYC (myelocytomatosis viral oncogene), MAX (MYC Associated Factor X), TAL1 (T-Cell Acute Lymphocytic Leukemia), SREBP (Sterol Regulatory Element-Binding Protein), AHR (Aryl Hydrocarbon Receptor), ARNT (Aryl hydrocarbon receptor nuclear translocator), HEN (Nescient Helix-Loop-Helix 1), HLF (Hepatic Leukemia Factor), USF (Upstream Transcription Factor) (Jones, 2004; Sailsbery and Dean, 2012). Out of them, MYOD and MYOG belong to the myogenic regulatory factor (MRF) family known to play key roles in the determination and differentiation of skeletal muscle (Botzenhart et al., 2018). Besides them, a role in regulating myostatin expression has been largely demonstrated for CREB (Xie et al., 2018), MEF (Bo Li et al., 2012; Estrella et al., 2015) and C/EBP (Allen et al., 2010; Deng et al., 2012), for which potential binding sites were also detected in our *in silico* analysis of the dromedary myostatin gene upstream sequence. Moreover, in the about 400 bp region upstream to the transcriptional start site, three TATA boxes and one CCAAT box were observed, consistently with previous reports by Spiller et al. (2002) in the bovine species and Du et al. (2011) in the ovine species. On the contrary, in the above region, we detected four E-boxes, unlike (Spiller et al., 2002) and (Du et al., 2005), who detected three and five E-boxes, respectively.

Most of the variants detected in our population sample, representative of seven different countries across the African and the Euro/Asiatic continent, could be also detected by aligning our *Camelus dromedarius* contig-8645394 with contigs available in public databases, representative of animals of African and Asiatic descent (Accession No. LSZX01094446.1, from a Targui animal sampled in Algeria, and Accession No. JDVD01004726, from an animal sampled in the Kingdom of Saudi Arabia, respectively). This result suggests that (i) our population sample, despite being limited in size, could be considered as providing a good representation of the species genetic diversity, and (ii) a limited differentiation may exist also among geographically distant samples, as pointed out by the preliminary results of the first world-wide *Camelus dromedarius* genetic diversity survey performed using genome-wide RAD-sequencing (Ciani et al., 2017)^6^, and in line with the known recurrent gene flow at ancient trading centers along the caravan routes (Almathen et al., 2016).

### Myostatin Expression Profiling in Skeletal Muscles

In order to quantify the level of myostatin transcript in dromedary skeletal muscle, we adopted a Digital Droplet PCR approach. In our experiments, we used two different probes designed on the two exon-junctions of the myostatin gene and differentially dye-labeled. The FAM-labeled probe gave systematically higher values compared to the HEX-labeled one. This result agrees with the known evidence that FAM has a stronger signal compared to other dyes. Indeed, this feature may determine a larger number of droplets, where target amplification occurred for both assays, to be designed as “positive” for the FAM-labeled probe compared to the HEX-labeled probe at a given fluorescence threshold. Alternatively, a different absolute quantification in a two-probe system targeting the same gene may arise from the presence of alternative transcripts that may reduce the amplification/detection efficiency of one of the two assays, but not necessarily both. However, in our study, given the systematic and consistent differences between the two assays across eight different skeletal muscles, a major role for the alternative transcript phenomenon appears rather unlike. Finally, we cannot exclude a minor role of stochastic factors in affecting the observed results.

In our experimental conditions, no significant differences were observed for the eight considered muscles by any of the two assays. These results are in line with those previously published by Morrison et al. (2014) who did not find significant variation in myostatin expression levels, assessed via Quantitative Real-time PCR, among four skeletal muscles (*rectus abdominis, longus colli, adductor, pectoralis transversus*) in the horse species. A similar scenario was observed in this study also at the protein level where no significant difference was observed among muscle types for the three myostatin forms (25, 40, and 75 kDa), with the exception of four pair-wise comparisons, all involving the *tensor fasciae latae* muscle, where a significantly lower expression was observed only for the promyostatin dimer. For the other two myostatin forms, *tensor fasciae latae* displayed weaker bands although they did not reach significance when contrasted to other muscles. The generally low expression of the three myostatin forms in the *tensor fasciae latae* muscle tempted us to speculate about a possible relationship with the fiber type composition of this muscle, which has been reported, in various species, to be mainly of the fast glycolytic type (Ariano et al., 1973; Abe et al., 1987; Manabe et al., 2000; Sazili et al., 2005; Bakou et al., 2015). In fact, some authors previously reported about a negative correlation between myostatin and the fast phenotype of skeletal muscles (Bouley et al., 2005; Hennebry et al., 2009; Baan et al., 2013). However, muscle phenotypes may be affected by many endogenous and exogenous factors, such as stage of maturity (Kugelberg, 1976), level of activity (Goldspink, 1983), different sampling regions of the same muscle (Torrella et al., 2000), histological method (Karlsson et al., 1999). Hence, the known large variability of muscle fiber phenotypes, coupled to the lack of specific data for the dromedary camels, makes it hazardous to extend the mentioned correlation to the species under study.

In general, densitometric analysis highlighted that the promyostatin dimer is the most expressed form in all the considered muscles while the active myostatin has the lowest level of expression. The above results fit well with multiple evidences that, in muscle, myostatin resides primarily as unprocessed promyostatin (Anderson et al., 2008; Pirruccello-Straub et al., 2018) and that the active mature growth factor is significantly less abundant in this compartment (Hill et al., 2002, 2003; Zimmers et al., 2002; Anderson et al., 2008; Lakshman et al., 2009). Moreover, it must be pointed out that the observed 25 kDa bands, suggestive of the active myostatin form, could, in our study, reflect the amount of myostatin dimers, deriving from the latent complex generated by furin cleavage, and artificially “activated” by the experimental SDS environment (Wehling et al., 2000), rather than reflecting a physiologically activated form. Based on the above, the unprocessed or partially processed myostatin dimers could act as important reservoirs of slowly available myostatin forms, and the sequential cleavage steps contribute an additional layer of control, within an already complex regulatory framework.

## Ethics Statement

Tissue sampling was done on slaughterhouses from dead animals. Blood sampling from 25 Old World camels was collected during routine veterinary procedures or as part of a monitoring program of the wild camel population in Mongolia.

## Author Contributions

MF performed the experiments, analyzed the data, prepared the figures and contributed toward writing the manuscript. RF performed the experiments and analyzed the data. LG and CP performed the experiments, analyzed the data, and contributed to the discussion and toward writing the manuscript. BF helped in sample collection and contributed to the discussion. AO performed the RACE-PCR experiments and contributed to the discussion. PB performed the experiments, analyzed the data, and contributed to the discussion. EC designed the research, performed the experiments, analyzed the data, and wrote the manuscript. All authors have read and approved the final manuscript.

## Conflict of Interest Statement

The authors declare that the research was conducted in the absence of any commercial or financial relationships that could be construed as a potential conflict of interest. The reviewer RM declared a shared affiliation, with no collaboration, with several of the authors MF, LG, CP, and EC to the handling Editor at the time of review.

## Footnotes

1 http://www.cbs.dtu.dk/services/SignalP/

2 http://sigpep.services.came.sbg.ac.at/coside.html

3 http://www.csbio.sjtu.edu.cn/bioinf/Signal-3L/

4 http://genome-euro.ucsc.edu/index.html

5 https://www.uniprot.org/uniprot/

6 https://pag.confex.com/pag/xxv/meetingapp.cgi/Paper/23709

## Supplementary Material

FIGURE S1 Electropherograms of 5′ and 3′ RACE PCR products. **(A)** Partial sequence of the 5′ untranslated region (UTR) of the *C. dromedarius* myostatin transcript, together with a partial sequence of the universal oligonucleotide provided in the SMARTer RACE cDNA amplification kit (Clonetech). **(B)** Partial sequence of the 3′ untranslated region (UTR) of the *C. dromedarius* myostatin transcript, together with part of the poly(A) tail.

FIGURE S2 RACE PCR products separated via 2% agarose gel electrophoresis. The upper arrow corresponds to 366 base pairs, while the lower arrow corresponds to 316 base pairs.

FIGURE S3 Comparative analysis of the myostatin genomic locus in the three Old World camelid species. Dromedary, *C. dromedarius* (contig-8645394). Wild camel, *C. ferus* (contig-7907533). Bactrian camel, *C. bactrianus* (contig-8938518). Highlighted, in the *C. dromedarius* sequence, the 5′ and 3′ UTR regions (light blue) and the three exons (yellow).

FIGURE S4 Putative SNPs in the myostatin coding region, as inferred from alignment among previously published myostatin sequences and sequences generated in this study. **(A)** Consensus sequence of the myostatin coding region. Highlighted, in yellow, the exonic region, and, in red, the variant sites. Numbering of polymorphism positions refers to the contig AGVR01040332. **(B)** Summary table of the inferred polymorphisms showing the nature of the putative variants, together with the reference literature, and the prediction of the variant effects (in case of a missense mutation, the alternative amino acids are reported, otherwise “none” is entered).

FIGURE S5 Plot of the Bayesian clustering analysis perfoemed on the nine considered dromedaries using the 69 identified SNPs. The plot shows the results obtained for K = 7, that was identified as the most likely output by visual inspection of the probability values associated to each tested K value (from 1 to 9). Numbers indicate different samples (1, United Arab Emirates; 2, Qatar; 3-4-5, Kingdom of Saudi Arabia; 6, Austria; 7, Kenya; 8, Sudan; 9, Pakistan). Colors indicate the seven different clusters. The proportion of each individual sample in each inferred cluster is shown in the y-axis.

FIGURE S6 Results of the Human Splicing Finder analysis for the three intronic SNPs detected in this study. **(A)** G66148C. **(B)** A66460G. **(C)** T66461C.

FIGURE S7 Graphical outline of the major predicted regulatory motifs in the 1.5 kb proximal to the transcription initiation site of the *C. dromedarius* myostatin gene. TATA boxes (highlighted in yellow), E-boxes (highlighted in gray), CREBP1 (light green), CREB_01 (orange), MYOGNF1 (blue), CEBP_01 (purple), MEF (pink), MYOD (dark green), and the CCAAT box (highlighted in purple) are presented.

FIGURE S8 Graphical evaluation of the Digital Droplet PCR performance. **(A)** 2D-dot plot of fluorescent signals detected in the Digital Droplet PCR experiments. Fluorescence results are plotted as two-dimensional dot plots. Gray dots correspond to empty droplets. Blue dots correspond to droplets containing at least one copy of the sequence complementary to Probe 1. Green dots correspond to droplets containing at least one copy of the sequence complementary to Probe 2. Orange dots correspond to droplets containing at least one copy of the sequence complementary to Probe 1 and at least one copy of the sequence complementary to Probe 2 (double-positive droplets). The observed pattern is relatively well balanced and dot clouds are well separated, suggesting (i) the absence of significant bias in the amplification of the two regions targeted by the two considered probes (exon1/exon2, and exon2/exon3) and (ii) the applicability of the BIORAD proprietary auto-analysis tool for target quantification. **(B)** Number of droplets generated in the Digital Droplet PCR experiments. Results for eight dromedary skeletal muscles, each from two different animals (biological replicate), analyzed in duplicate (technical replicate) are shown.

TABLE S1 Sequence accession numbers (left panel) and links to the webpages of databases and softwares (right panel) considered in this study.

DATA SHEET S1 Amino acidic sequences of the 83 non redundant myostatin proteins used for building the maximum likelihood tree presented in Figure 3. The data are in the “multiple sequence alignment” format.

DATA SHEET S2 Sequence of the *Camelus dromedarius* contig-8645394. Exons (highlighted in yellow), 5′ and 3′ UTR regions (underlined), SNPs (highlighted in red), the GAT codon for the aspartic acid essential for BMP/tolloid protease cleavage (highlighted in green), and the 12 nucleotides coding for the RSRR motif (highlighted in gray), needed for recognition by furin convertase, are shown.

DATA SHEET S3 Results of the TFBIND analysis for the SNPs present in the *C. dromedarius* myostatin gene upstream region (8 kb). AC, transcription factor matrix code. ID, transcription factor label, with V meaning “vertebrate”. SCORE, similarity (0.0-1.0) between a registered sequence for the transcription factor binding sites and the input sequence. STRAND, strandness. + and - means forward and reverse strands that the transcription factor binds, respectively. CONSENSUS, consensus sequence (fixed) of the transcription factor binding sites. S = C or G, W = A or T, R = A or G, Y = C or T, K = G or T, M = A or C, N = any base pair. SIGNAL, sub-sequence from the input sequence at the position corresponding to the consensus sequence. Default cut-offs by Tsunoda and Takagi, 1999 were adopted. The analysis was performed, for each SNP, using the two input sequences harboring the alternative alleles.
