# Supplementary figures and images for: Beyond the Big Five: Investigating Myostatin Structure, Polymorphism and Expression in Camelus dromedarius
Source: Front Genet. 2019 Jun 7;10:502. doi: 10.3389/fgene.2019.00502 (PMC6566074; doi:10.3389/fgene.2019.00502)

## A

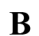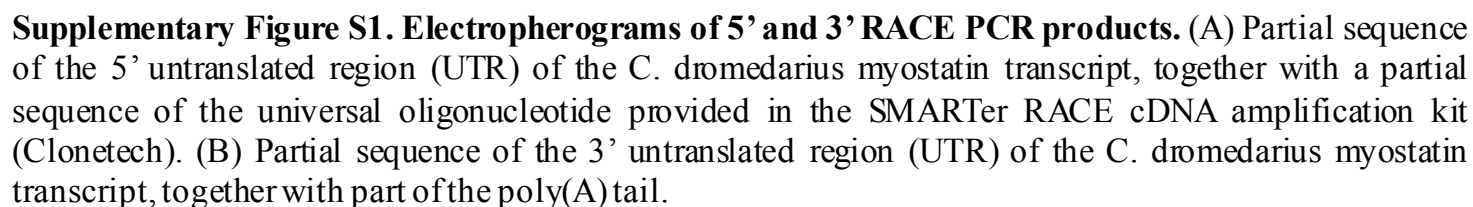

Supplement: FIGURE S1 — Electropherograms of 5′ and 3′ RACE PCR products. (A) Partial sequence of the 5′ untranslated region (UTR) of the C. dromedarius myostatin transcript, together with a partial sequence of the universal oligonucleotide provided in the SMARTer RACE cDNA amplification kit (Clonetech). (B) Partial sequence of the 3′ untranslated region (UTR) of the C. dromedarius myostatin transcript, together with part of the poly(A) tail. [file Image_1.pdf]
