# Supplementary material for: Beyond the Big Five: Investigating Myostatin Structure, Polymorphism and Expression in Camelus dromedarius
Source: Front Genet. 2019 Jun 7;10:502. doi: 10.3389/fgene.2019.00502 (PMC6566074; doi:10.3389/fgene.2019.00502)
Supplement: FIGURE S2 — RACE PCR products separated via 2% agarose gel electrophoresis. The upper arrow corresponds to 366 base pairs, while the lower arrow corresponds to 316 base pairs. [file Image_2.pdf]

Supplementary Figure S2

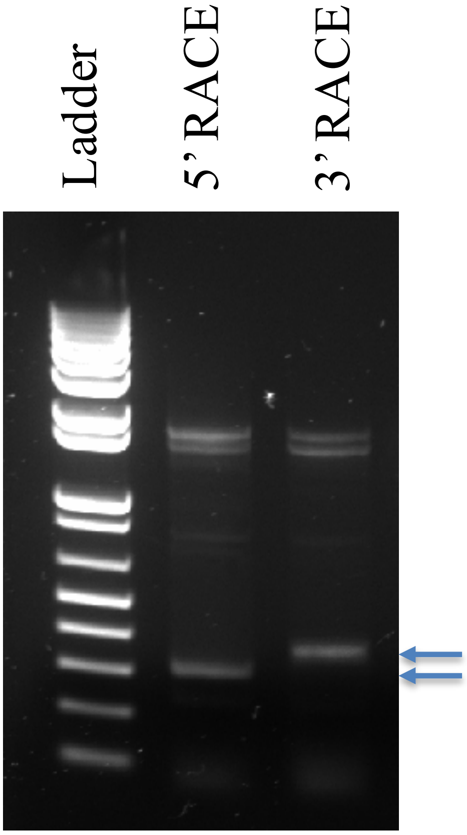

Supplementary Figure S2. RACE PCR products separated via 2% agarose gel electrophoresis. The upper arrow corresponds to 366 base pairs, while the lower arrow corresponds to 316 base pairs.
