# Supplementary material for: Beyond the Big Five: Investigating Myostatin Structure, Polymorphism and Expression in Camelus dromedarius
Source: Front Genet. 2019 Jun 7;10:502. doi: 10.3389/fgene.2019.00502 (PMC6566074; doi:10.3389/fgene.2019.00502)
Supplement: FIGURE S3 — Comparative analysis of the myostatin genomic locus in the three Old World camelid species. Dromedary, C. dromedarius (contig-8645394). Wild camel, C. ferus (contig-7907533). Bactrian camel, C. bactrianus (contig-8938518). Highlighted, in the C. dromedarius sequence, the 5′ and 3′ UTR regions (light blue) and the three exons (yellow). [file Image_3.pdf]

Supplementary Figure S3

Comparative analysis of the myostatin genomic locus in the three Old World camelid species. Dromedary, *C. dromedarius* (contig-8645394). Wild camel, *C. ferus* (contig-7907533). Bactrian camel, *C. bactrianus* (contig-8938518). Highlighted, in the *C. dromedarius* sequence, the 5' and 3' UTR regions (light blue) and the three exons (yellow).

```
Dromedary      GTACCTTCTCTTGTTAGTATTAATTTTTTAAGTTCTAGAGACAATGGGAAATAATCTTGA 184
Wild           -----------------------------------------------------------A 1
Domestic       GTACCTTCTCTTGTTAGTATTAATTTTTTAAATTCTAGAGACAATGGGAAATAATCTTGA 420
                                                                          *

Dromedary      GATGGTCGTACAGAATTATCATTGTTATGTCCCCATTAACTTCGATTTTTTTCCCACAAT 244
Wild           GATGGTCGTACAGAATTATCATTGTTATGTCCCCATTAACTTCGATTTTTTTCCCACAAT 61
Domestic       GATGGTCGTACAGAATTATCATTGTTATGTCCCCATTAACTTCGATTTTTTTCCCACAAT 480
               ************************************************************

Dromedary      GGATATAGCATACCCTTCCCTTAATCATGCATACTCATTTAAAAAAATTATGCAACTAAA 304
Wild           GGATATAGCATACCCTTCTCTTAATCATGCATACTCATTTAAAAAAATTATGCAACTAAA 121
Domestic       GGATATAGCATACCCTTCTCTTAATCATGCATACTCATTTAAAAAAATTATGCAACTAAA 540
               ****************** *****************************************

Dromedary      AATTAAAAACCAAAGTAAAAATAAAAATCACATAGAATACACTCTTCCCCTCAGATAATC 364
Wild           AATTAAAAACCAAAGTAAAAATAAAAATCACATAGAATACACTCTTCCCCTCAGATAATC 181
Domestic       AATTAAAAACCAAAGTAAAAATAAAAATCACATAGAATACACTCTTCCCCTCAGATAATC 600
               ************************************************************

Dromedary      ATTCTTTCATAAACAGCTGTATGAGATTATAGCTACATAATTTTTTTTACCATGAATAGT 424
Wild           ATTCTTTCATAAACAGCTGTATGAGATTATAGCTACATAATTTTTTTTACCATGAATAGT 241
Domestic       ATTCTTTCATAAACAGCTGTATGAGATTATAGCTACATAATTTTTTTTACCATGAATAGT 660
               ************************************************************

Dromedary      ATATTTACTTCTTCCATAAGGAATAATGTTACCTCACGTGTAGCCCTCCATACCTTTCTG 484
Wild           ATATTTACTTCTTCCATAAGGAATAATGTTACCTCACGTGTAGCCCTCCATACCTTTCTG 301
Domestic       ATATTTACTTCTTCCATAAGGAATAATGTTACCTCACGTGTAGCCCTCCATACCTTTCTG 720
               ************************************************************

Dromedary      GTATTGAAACATATTCTAGATAAACAGCATGCTATCAGGGTGTGTGTTTGTGTGTACAAA 544
Wild           GTATTGAAACATATTCTAGATAAACAGCATGCTATCAGGGTGTGTGTTTGTGTGTACAAA 361
Domestic       GTATTGAAACATATTCTAGATAAACAGCATGCTATCAGGGTGTGTGTTTGTGTGTACAAA 780
               ************************************************************

Dromedary      GTTTAGGGGGGTCATTTGTTTTGATAATAATGTGATCTTATTATATAAAACTCTATACAT 604
Wild           GTTTAGGGGGGTCATTTGTTTTGATAATAATGTGATCTTATTATATAAAACTCTATACAT 421
Domestic       GTTTAGGGGGGTCATTTGTTTTGATAATAATGTGATCTTATTATATAAAACTCTATACAT 840
               ************************************************************

Dromedary      TAAGATTTTCATACTGAAGAGTATTATGAGACTCTGCAAGTCAGCAGCATAAGGAGAAAA 664
Wild           TAAGATTTTCATACTGAAGAGTATTATGAGACTCTGCAAGTCAGCAGCATAAGGAGAAAA 481
Domestic       TAAGATTTTCATACTGAAGAGTATTATGAGACTCTGCAAGTCAGCAGCATAAGGAGAAAA 900
               ************************************************************

Dromedary      TACAGTGATTTTAAATACAGTTCTGTGAGGCCTTGGGTAACTTTTTAAAAACTCTATGTT 724
Wild           TACAGTGATTTTAAATACAGTTCTGTGAGGCCTTGGGTAACTTTTTAAAAACTCTATGTT 541
Domestic       TACAGTGATTTTAAATACAGTTCTGTGAGGCCTTGGGTAACTTTTTAAAAACTCTGTGTT 960
               ******************************************************* ****

Dromedary      TTAGTTCCCTTTCCTATCACGTTGTTACCACCTACTTCACCAGGCTGTGAGGATCCAGAG 784
Wild           TTAGTTCCCTTTCCTATCACGTTGTTACCACCTACTTCACCAGGCTGTGAGGATCCAGAG 601
Domestic       TTAGTTCCCTTTCCTATCACGTTGTTACCACCTACTTCACCAGGCTGTGAGGATCCAGAG 1020
               ************************************************************

Dromedary      TGCTTCATAAGAACCTAATTTATTCCTTCCAGTGGCTGTGTAGTACCTGACATGGGTGTG 844
Wild           TGCTTCATAAGAACCTAATTTATTCCTTCCAGTGGCTGTGTAGTACCTGACATGGGTGTG 661
Domestic       TGCTTCATAAGAACCTAATTTATTCCTTCCAGTGGCTGTGTAGTACCTGACATGGGTGTG 1080
               ************************************************************
```

```
Dromedary      CCATACTGAGTCACCTACTGAGGGGCATTCACTTTGTTTCCAGTCTTTGACTAGCACAAT  904
Wild           CCATACTGAGTCACCTACTGAGGGGCATTCACTTTGTTTCCAGTCTTTGACTAGCACAAT  721
Domestic       CCATACTGAGTCACCTACTGAGGGGCATTCACTTTGTTTCCAGTCTTTGACTAGCACAAT  1140
               ************************************************************

Dromedary      GTTCAATAAACATAACTGTGTGTACACCTTACCTAGTAGTGATTTTATTCCCATGGGAGA  964
Wild           GTTCAATAAACATAACTGTGTGTACACCTTACCTAGTAGTGATTTTATTCCCATGGGAGA  781
Domestic       GTTCAATAAACATAACTGTGTGTACACCTTACCTAGTAGTGATTTTATTCCCATGGGAGA  1200
               ************************************************************

Dromedary      GATTCCTAAGCGTGGGATAGGTAAATGTATCATTAATTTCAATGGTTAAAACCATATTTC  1024
Wild           GATTCCTAAGCGTGGGATAGGTAAATGTATCATTAATTTCAATGGTTAAAACCATATTTC  841
Domestic       GATTCCTAAGCGTGGGATAGGTAAATGTATCATTAATTTCAATGGTTAAAACCATATTTC  1260
               ************************************************************

Dromedary      TTTTCAAAAAGACTGAAACAATGACTATTTCCACATGCACAAGAGCACCCTTCTCCCCAA  1084
Wild           TTTTCAAAAAGACTGAAACAATGACTATTTCCACATGCACAAGAGCACCCTTCTCCCCAA  901
Domestic       TTTTCAAAAAGACTGAAACAATGACTATTTCCACATGCACAAGAGCACCCTTCTCCCCAA  1320
               ************************************************************

Dromedary      AATTCCTCAGCAATAGATATTTTCTCTCTCAGCTTTTGCTCATCTAATGGCTTTGAGAGG  1144
Wild           AATTCCTCAGCAATAGATATTTTCTCTCTCAGCTTTTGCTCATCTAATGGCTTTGAGAGG  961
Domestic       AATTCCTCAGCAATAGATATTTTCTCTCTCAGCTTTTGCTCATCTAATGGCTTTGAGAGG  1380
               ************************************************************

Dromedary      ATGCGTCATTGTTACTTTACTTCCCTGACTCCCGGCAAGGTGGAGCATGAGTTCACGTTT  1204
Wild           ATGCGTCATTGTTACTTTACTTCCCTGACTCCCGGCAAGGTGGAGCATGAGTTCACGTTT  1021
Domestic       ATGCGTCATTGTTACTTTACTTCCCTGACTCCCGGCAAGGTGGAGCATGAGTTCACGTTT  1440
               ************************************************************

Dromedary      ATTGGCAACTTGGATTTGCCTTCTCCGTATCTCATTTGCCCATTTTTCTTTTTGGTTATT  1264
Wild           ATTGGCAACTTGGATTTGCCTTCTCCGTATCTCATTTGCCCATTTTTCTTTTTGGTTATT  1081
Domestic       ATTGGCAACTTGGATTTGCCTTCTCCGTATCTCATTTGCCCATTTTTCTTTTTGGTTATT  1500
               ************************************************************

Dromedary      TATGTTTTTATCCATTAGTGAGCACTTTTGTATGGTTTACAGCTTCTCTATCATGTATTT  1324
Wild           TATGTTTTTATCCATTAGTGAGCACTTTTGTATGGTTTACAGCTTCTCTATCATGTATTT  1141
Domestic       TATGTTTTTATCCATTAGTGAGCACTTTTGTATGGTTTACAGCTTCTCTATCATGTATTT  1560
               ************************************************************

Dromedary      CAAATAATTTTCCCTGAGTTATAATTTCCTTTTCCAAAGTAAAATTTTTATTGAAGCACA  1384
Wild           CAAATAATTTTCCCTGAGTTATAATTTCCTTTTCCAAAGTAAAATTTTTATTGAAGCACA  1201
Domestic       CAAATAATTTTCCCTGAGTTATAATTTCCTTTTCCAAAGTAAAATTTTTATTGAAGCACA  1620
               ************************************************************

Dromedary      ACATTCATTTCCTTCTGGTTTACATTTGATCTCTTATAAACGAGTTAAAATTTAAAATGT  1444
Wild           ACATTCATTTCCTTCTGGTTTACATTTGATCTCTTATAAACGAGTTAAAATTTAAAATGT  1261
Domestic       ACATTCATTTCCTTCTGGTTTACATTTGATCTCTTATAAACGAGTTAAAATTTAAAATGT  1680
               ************************************************************

Dromedary      GTGTCTTCTCCTCCACGGCTTCTTGGTCCCGTCCCGATTAATCAACAACTTGAGGGATTT  1504
Wild           GTGTCTTCTCCTCCACAGCTTCTTGGTCCCGTCCCGATTAATCAACAACTTGAGGGATTT  1321
Domestic       GTGTCTTCTCCTCCACAGCTTCTTGGTCCCGTCCCGATTAATCAACAACTTGAGGGATTT  1740
               **************** *******************************************

Dromedary      CTGTGTGTATATGAGACCTATTCTTTCTTTCCTTTAGGCTATCTGGAAACTACCTGATAC  1564
Wild           CTGTGTGTATATGAGACCTATTCTTTCTTTCCTTTAGGCTATCTGGAAACTACCTGATAC  1381
Domestic       CTGTGTGTATATGAGACCTATTCTTTCTTTCCTTTAGGCTATCTGGAAACTACCTGATAC  1800
               ************************************************************

Dromedary      AGTTTTTTCCAGATGAATAGGTTGCTGGGTCAGCACCATTTTAAAAATAACCTGTCTTTT  1624
Wild           AGTTTTTTCCAGATGAATAGGTTGCTGGGTCAGCACCATTTTAAAAATAACCTGTCTTTT  1441
Domestic       AGTTTTTTCCAGATGAATAGGTTGCTGGGTCAGCACCATTTTAAAAATAACCTGTCTTTT  1860
               ************************************************************

Dromedary      CCCTTTCAACTGAAATACCACTTTTGTTATATATCAAATACCCGCATGTACTGGGATCTA  1684
Wild           CCCTTTCAACTGAAATACCACTTTTGTTATATATCAAATACCCGCATGTACTGGGATCTA  1501
Domestic       CCCTTTCAACTGAAATACCACTTTTGTTATATATCAAATACCCGCATGTACTGGGATCTA  1920
               ************************************************************
```

```
Dromedary      TTTCTGAGCTCTCTAGTCTCTTCTCTGACTTGTCTCTTTCTAAGCCAAAACCAAAGCCAA 1744
Wild           TTTCTGAGCTCTCTAGTCTCTTCTCTGACTTGTCTCTTTCTAAGCCAAAACCAAAGCCAA 1561
Domestic       TTTCTGAGCTCTCTAGTCTCTTCTCTGACTTGTCTCTTTCTAAGCCAAAACCAAAGCCAA 1980
               ************************************************************

Dromedary      TAAGGAGGTCATGATACGCTTCACTCATGCACATGTCACTAGGCTTTACTAGTCTTCTTA 1804
Wild           TAAGGAGGTCATGATACGCTTCAGTCATGCACATGTCACTAGGCTTTACTAGTCTTCTTA 1621
Domestic       TAAGGAGGTCATGATACGCTTCAGTCATGCACATGTCACTAGGCTTTACTAGTCTTCTTA 2040
               *********************** ************************************

Dromedary      TTTACATTTCTAATTCCATAGAATCTTTAAGATCATATTATCCAACCCCCCATCTTATTC 1864
Wild           TTTACATCTCTAATTCCATAGAATCTTTAAGATCATATTATCCAACCCCCCATCTTATTC 1681
Domestic       TTTACATCTCTAATTCCATAGAATCTTTAAGATCATATTATCCAACCCCCCATCTTATTC 2100
               ******* ****************************************************

Dromedary      AAATTTAAATTAGAATTTATTACATTTGCTTAATTTTGGAAGAAGTCATGTTTTTATGAT 1924
Wild           AAATTTAAATTAGAATTTATTACATTTGCTTAATTTTGGAAGAAGTCATGTTTTTATGAT 1741
Domestic       AAATTTAAATTAGAATTTATTACATTTGCTTAATTTTGGAAGAAGTCATGTTTTTATGAT 2160
               ************************************************************

Dromedary      ATTAAATCTTTCTATCTAAAAACAACAGATGTTGTTTAATATCTTTGTATAATGTTTTTT 1984
Wild           ATTAAATCTTTCTATCTAAAAACAACAGATGTTGTTTAATATCTTTGTATAATGTTTTTT 1801
Domestic       ATTAAATCTTTCTATCTAAAAACAACAGATGTTGTTTAATATCTTTGTATAATGTTTTTT 2220
               ************************************************************

Dromedary      AGATAGTCCATTGTTATTTTTTTCTCTTTTATAACTTGTTGCTAGTACAGAAAAAAGTAAT 2044
Wild           AGATAGTCCATTGTTATTTTTTTCTCTTTTATAACTTGTTGCTAGTACAGAAAAAAGTAAT 1861
Domestic       AGATAGTCCATTGTTATTTTTTTCTCTTTTATAACTTGTTGCTAGTACAGAAAAAAGTAAT 2280
               ************************************************************

Dromedary      TTATATTTATCTCTCTACCTTAGGTCAAATTCAACTTTAGTATTTCAATGAACATTCTAA 2104
Wild           TTATATTTATCTCTCTACCTTAGGTCAAATTCAACTTTAGTATTTCAATGAACATTCTAA 1921
Domestic       TTATATTTATCTCTCTACCTTAGGTCAAATTCAACTTTAGTATTTCAATGAACATTCTAA 2340
               ************************************************************

Dromedary      ACTATCTTCACTGTTAAAGCAGTCCTTGTTCTCAGCAGACATCCTCATTTCCTACTTTGT 2164
Wild           ACTATCTTCACTGTTAAAGCAGTCCTTGTTCTCAGCAGACATCCTCATTTCCTACTTTGT 1981
Domestic       ACTATCTTCACTGTTAAAGCAGTCCTTGTTCTCAGCAGACATCCTCATTTCCTACTTTGT 2400
               ************************************************************

Dromedary      TTAAAAAGTTGAGGTTATCAGCTCTCCAGACTTCTCTGTGCACAATCACCTCTTTCAAAC 2224
Wild           TTAAAAAATTGAGGTTATCAGCTCTCCAGACTTCTCTGTGCACAATCACCTCTTTCAAAC 2041
Domestic       TTAAAAAATTGAGGTTATCAGCTCTCCAGACTTCTCTGTGCACAATCACCTCTTTCAAAC 2460
               ******* ****************************************************

Dromedary      CCCTTCCTAACATCTTTTCCTCCATTCTCAAAGGGTGAGATTTGTTTCCTCTTCTTCAAG 2284
Wild           CCCTTCCTAACATCTTTTCCTCCATTCTCAAAGGGTGAGATTTGTTTCCTCTTCTTCAAG 2101
Domestic       CCCTTCCTAACATCTTTTCCTCCATTCTCAAAGGGTGAGATTTGTTTCCTCTTCTTCAAG 2520
               ************************************************************

Dromedary      CCCAGGCTCTATTGACTCCAATCCTTTCAAGTTCCTCAAGGACCTTGCTCCATTAGTTAT 2344
Wild           CCCAGGCTCTATTGACTCCAATCCTTTCAAGTTCCTCAAGGACCTTGCTCCATTAGTTAT 2161
Domestic       CCCAGGCTCTATTGACTCCAATCCTTTCAAGTTCCTCAAGGACCTTGCTCCATTAGTTAT 2580
               ************************************************************

Dromedary      GCCCCCTTCTCTCCTGCATCTTCCAACTCTGCTACTTATTTCTTTTTTTCAGTCTCTACT 2404
Wild           GCCCCCTTCTCTCCTGCATCTTCCAACTCTGCTACTTATTTCTTTTTTTCAGTCTCTACT 2221
Domestic       GCCCCCTTCTCTCCTGCATCTTCCAACTCTGCTACTTATTTCTTTTTTTCAGTCTCTACT 2640
               ************************************************************

Dromedary      CTGTGCTATGTTAAATTCCCCTTTTCTGTTGTGGAGAGGCTCCTTTAAATCTCTTGGCCA 2464
Wild           CTGTGCTATGTTAAATTCCCCTTTTCTGTTGTGGAGAGGCTCCTTTAAATCTCTTGGCCA 2281
Domestic       CTGTGCTATGTTAAATTCCCCTTTTCTGTTGTGGAGAGGCTCCTTTAAATCTCTTGGCCA 2700
               ************************************************************

Dromedary      GATTCTGCAGTCTTTCAGCAATTTCCAAGCATCATAACTACATCATTCTGATTGTTAGTT 2524
Wild           GATTCTGCAGTCTTTCAGCAATTTCCAAGCATCATAACTACATCATTGTGATTGTTAGTT 2341
Domestic       GATTCTGCAGTCTTTCAGCAATTTCCAAGCATCATAACTACATCATTCTGATTGTTAGTT 2760
               *********************************************** ************
```

```
Dromedary      TTTTTCCTACTCTTTCTTAACTTAGGCTAAAGCTACGGCTCTATCAACTTTATCAGCTTA 2584
Wild           TTTTTCCTACTCTTTCTTAACTTAGGCTAAAGCTACGGCTCTATCAACTTTATCAGCTTA 2401
Domestic       TTTTTCCTACTCTTTCTTAACTTAGGCTAAAGCTACGGCTCTATCAACTTTATCAGCTTA 2820
               ************************************************************

Dromedary      GGGTGAGAAGAAAGGCCAGAAAAATGTCTCCTTCCTTGACTGAGGAAAGTCACAATCCTC 2644
Wild           GGGTGAGAAGAAAGGCCAGAAAAATGTCTCCTTCCTTGACTGAGGAAAGTCACAATCCTC 2461
Domestic       GGGTGAGAAGAAAGGCCAGAAAAATGTCTCCTTCCTTGACTGAGGAAAGTCACAATCCTC 2880
               ************************************************************

Dromedary      TATGTTGGGTATTGTTGCTTCTGTGGTTAACATTGAATAGCCATCAATATCCTCTGGGGA 2704
Wild           TATGTTGGGTATTGTTGCTTCTGTGGTTAACGTTGAATAGCCATCAATATCCTCTGGGGA 2521
Domestic       TATGTTGGGTATTGTTGCTTCTGTGGTTAACGTTGAATAGCCATCAATATCCTCTGGGGA 2940
               ******************************* ****************************

Dromedary      GCACACGTTCAAATGCTGGCTTTCTCTTCTAATACATGCATGTGCCAGCTAAAGAATGTT 2764
Wild           GCACACGTTCAAATGCTGGCTTTCTCTTCTAATACATGCATGTGCCAGCTAAAGAATGTT 2581
Domestic       GCACACGTTCAAATGCTGGCTTTCTCTTCTAATACATGCATGTGCCAGCTAAAGAATGTT 3000
               ************************************************************

Dromedary      GCATACCATCTGTTTCTTTAAGAATGAAGTCACTCTTCACTGCAGTCACTGACCTTTAAC 2824
Wild           GCATACCATCTGTTTCTTTAAGAATGAAGTCACTCTTCACTGCAGTCACTGACCTTTAAC 2641
Domestic       GCATACCATCTGTTTCTTTAAGAATGAAGTCACTCTTCACTGCAGTCACTGACCTTTAAC 3060
               ************************************************************

Dromedary      ACACCCTGAAAGGAGTTCAGGGTGGAGATCAGGAATGAAGTATTCTGTGCTCTGGGAAAA 2884
Wild           ACACCCTGAAAGGAGTTCAGGGTGGAGATCAGGAATGAAGTATTCTGTGCTCTGGGAAAA 2701
Domestic       ACACCCTGAAAGGAGTTCAGGGTGGAGATCAGTAATGAAGTATTCTGTGCTCTGGGAAAA 3120
               ******************************** ***************************

Dromedary      ACTGGCAGAACAGGCCTTCAGATAGATTACTTTCAAGGGAAAATTTTATGAACTCAATTT 2944
Wild           ACTGGCAGAACAGGCCTTCAGATAGATTACTTTCAAGGGAAAATTTTATGAACTCAATTT 2761
Domestic       ACTGGCAGAACAGGCCTTCAGATAGATTACTTTCAAGGGAAAATTTTATGAACTCAATTT 3180
               ************************************************************

Dromedary      CTTGCATCTTCACATGCCTAGAAAAGCACTAAAATTATTCATGGCTCCTCATCACTGGCA 3004
Wild           CTTGCATCTTCACATGCCTAGAAAAGCACTAAAATTATTCATGGCTCCTCATCACTGGCA 2821
Domestic       CTTGCATCTTCACATGCCTAGAAAAGCACTAAAATTATTCATGGCTCCTCATCACTGGCA 3240
               ************************************************************

Dromedary      GCAGCCTTCTACTGAGATGTGTGCTGGACTGCACGTACCCCTTACACTAAAATCATGTAT 3064
Wild           GCAGCCTTCTACTGAGATGTGTGCTGGACTGCACGTACCCCTTACACTAAAATCATGTAT 2881
Domestic       GCAGCCTTCTACTGAGATGTGTGCTGGACTGCACGTACCCCTTACACTAAAATCATGTAT 3300
               ************************************************************

Dromedary      ATACTGAACTCCCCCACTACCTCTTTGGAGGAGTTCCTCAGAGCTATCTGAAAGGCTGTC 3124
Wild           ACACTGAACTCCCCCACTACCTCTTTGGAGGAGTTCCTCAGAGCTATCTGAAAGGCTGTC 2941
Domestic       ACACTGAACTCCCCCACTACCTCTTTGGAGGAGTTCCTCAGAGCTATCTGAAAGGCTGTC 3360
               * **********************************************************

Dromedary      TCCCGGGCTATGGTCCTCAGTAAGCCCCAAACAAAACTGAACCTAGAGCTCTCACATTGT 3184
Wild           TCCCGGGCTATGGTCCTCAGTAAGCCCCAAACAAAACTGAACCTAGAGCTCTCACATTGT 3001
Domestic       TCCCGGGCTATGGTCCTCAGTAAGCCCCAAACAAAACTGAACCTAGAGCTCTCACATTGT 3420
               ************************************************************

Dromedary      GCTTTTTTTCTTTTTTTCAGCTGACACATGGATTCTTAAGTAAGCCTTGGAAAAGAGTCTC 3244
Wild           GCTTTTTTTCTTTTTTTCAGCTGACACATGGATTCTTAAGTAAGCCTTGGAAAAGAGTCTC 3061
Domestic       GCTTTTTTTCTTTTTTTCAGCTGACACATGGATTCTTAAGTAAGCCTTGGAAAAGAGTCTC 3480
               ************************************************************

Dromedary      TCTATTGTGTAATTTCCTTGAGGGGAGATCTGCCTACAAGCATCTATCTCTTCTCCACTC 3304
Wild           TCTATTGTGTAATTTCCTTGAGGGGAGATCTGCCTACAAGCATCTATCTCTTCTCCACTC 3121
Domestic       TCTATTGTGTAATTTCCTTGAGGGGAGATCTGCCTACAAGCATCTATCTCTTCTCCACTC 3540
               ************************************************************

Dromedary      CCCAGTTAGCTTTTACACAGGCAGTCCACCTCACTGCCTGGAGTGGATCCATTCTCTGAC 3364
Wild           CCCAGTTAGCTTTTACACAGGCAGTCCACCTCACTGCCTGGAGTGGATCCATTCTCTGAC 3181
Domestic       CCCAGTTAGCTTTTACACAGGCAGTCCACCTCACTGCCTGGAGTGGATCCATTCTCTGAC 3600
               ************************************************************
```

```
Dromedary      CAAATGGCCATGGTATTTTGCAACTTCTCCCTAAGATTAGAGTTAACTTCCCCACCCTTT 3424
Wild           CAAATGGCCATGGTATTTTGCAACTTCTCCCTAAGATTAGAGTTAACTTCCCCACCCTTT 3241
Domestic       CAAATGGCCATGGTATTTTGCAACTTCTCCCTAAGATTAGAGTTAACTTCCCCACCCTTT 3660
               ************************************************************

Dromedary      GAATCTGGACTCAGCCGTGAAACTTGCTTCGGCTAGCAGAACATAAGAAAATGTGACTTA 3484
Wild           GAATCTGGACTCAGCCGTGAAACTTGCTTCGGCTAGCAGAACATAAGAAAATGTGACTTA 3301
Domestic       GAATCTGGACTCAGCCGTGAAACTTGCTTCGGCTAGCAGAACATAAGAAAATGTGACTTA 3720
               ************************************************************

Dromedary      TGGATTTGTGTGCATGATACACTTTTATTGTAAAGAAAAACTGAGCCCTATGCTGTAGAC 3544
Wild           TGGATTTGTGTGCATGATACACTTTTATTGTAAAGAAAAACTGAGCCCTATGCTGTAGAC 3361
Domestic       TGGATTTGTGTGCATGATACACTTTTATTGTAAAGAAAAACTGAGCCCTATGCTGTAGAC 3780
               ************************************************************

Dromedary      AAGAGGCACTCCAAAGACAATTGAAATTGATAAATTAGGTTGTAGTAGCCTAAGCATAGT 3604
Wild           AAGAGGCACTCCAAAGACAATTGAAATTGATAAATTAGGTTGTAGTAGCCTAAGCATAGT 3421
Domestic       AAGAGGCACTCCAAAGACAATTGAAATTGATAAATTAGGCTGTAGTAGCCTAAGCATAGT 3840
               *************************************** ********************

Dromedary      TAAGCGTATCAAGGTTAAACACTGTAAGATAATACTAAAGTTGTGAATTGGAATTAAGAG 3664
Wild           TAAGCGTATCAAGGTTAAACACTGTAAGATAATACTAAAGTTGTGAATTGGAATTAAGAG 3481
Domestic       TAAGCGTATCAAGGTTAAACACTGTAAGATAATACTAAAGTTGTGAATTGGAATTAAGAG 3900
               ************************************************************

Dromedary      AAATCCTTAAAGAAGTGGTTACAAACTGGTTTAGCTGTTGCTTATATATGTTGCAAATAT 3724
Wild           AAATCCTTAAAGAAGTGGTTACAAACTGGTTTAGCTGTTGCTTATATATGTTGCAAATAT 3541
Domestic       AAATCCTTAAAGAAGTGGTTACAAACTGGTTTAGCTGTTGCTTATATATGTTGCAAATAT 3960
               ************************************************************

Dromedary      CTTCTCCA-CCCTCCAAAAAGTACCATTATCTAGCCTCACAGAGGAATCTTTGGAGAGCA 3783
Wild           CTTCTCCACCCCCCCAAAAAGTACCATTATCTAGCCTCACAGAGGAATCTTTGGAGAGCA 3601
Domestic       CTTCTCCACCCCCCCAAAAAGTACCATTATCTAGCCTCACAGAGGAATCTTTGGAGAGCA 4020
               ******** *** ***********************************************

Dromedary      GGTAATTCTAAAGCTATTTAAGCTGTCCCAGTGCCTAGAAAAAGTAAAACCTCCAAATTA 3843
Wild           GGTAATTCTAAAGCTATTTAAGCTGTCCCAGTGCCTAGAAAAAGTAAAACCTCCAAATTA 3661
Domestic       GGTAATTCTAAAGCTATTTAAGCTGTCCCAGTGCCTAGAAAAAGTAAAACCTCCAAATTA 4080
               ************************************************************

Dromedary      TTTTATAAATTACAACACTGATACTAAAACCTGATAGACTGCAAAAAGATGATCAGTTTT 3903
Wild           TTTTGTAAATTACAACACTGATACTAAAACCTGATAGACTGCAAAAAGATGATCAGTTTT 3721
Domestic       TTTTGTAAATTACAACACTGATACTAAAACCTGATAGACTGCAAAAAGATGATCAGTTTT 4140
               **** *******************************************************

Dromedary      ACTTATGAATTTCAATTCAAAAAGACAATAAAATATTAACAAACAGCACAGAGAATAATG 3963
Wild           ACTTATGAATTTCAATTCAAAAAGACAATAAAATATTAACAAACAGCACAGAGAATAATG 3781
Domestic       ACTTATGAATTTCAATTCAAAAAGACAATAAAATATTAACAAACAGCACAGAGAATAATG 4200
               ************************************************************

Dromedary      TGTCATAATCAAATGGGGTTCCTCCCCAACACCTATGCAAGAATATAAGAATAGTTCAAT 4023
Wild           TGTCATAATCAAATGGGGTTCCTCCCCAACACCTATGCAAGAATATAAGAATAGTTCAAT 3841
Domestic       TGTCATAATCAAATGGGGTTCCTCCCCAACACCTATGCAAGAATATAAGAATAGTTCAAT 4260
               ************************************************************

Dromedary      GTACAAAATTAATTCATGTATTTCAGCATATCAATAAATCCATGGAAAAAAACTATGTTT 4083
Wild           GTACAAAATTAATTCATGTATTTCAGCATATCAATAAATCCATGGAAAAAAACTATGTTT 3901
Domestic       GTACAAAATTAATTCATGTATTTCAGCATATCAATAAATCCATGGAAAAAAACTATGTTT 4320
               ************************************************************

Dromedary      ATACATATAGTCATTGAAAAGGAATTTGATAAGAATCAAATCTGCTCTTGATTTTT-AAA 4142
Wild           ATACATATAGTCATTGAAAAGGAATTTGATAAAAATCAAATCTGCTCTTGATTTTTAAAA 3961
Domestic       ATACATATAGTCATTGAAAAGGAATTTGATAAAAATCAAATCTGCTCTTGATTTTTAAAA 4380
               ******************************** *********************** ***

Dromedary      AAAATACACTCAAAATAAGAGTTGATAAAAACATCCTTTATGAGTATACATATTTTATCT 4202
Wild           AAAATACACTCAAAATAAGAGTTGATAAAAACGTCCTTTATGAGTATACATATTTTATCT 4021
Domestic       AAAATACACTCAAAATAAGAGTTGATAAAAACGTCCTTTATGAGTATACATATTTTATCT 4440
               ******************************** ***************************
```

```
Dromedary      TACCCTTAAGGCTAGCATATTTCCTAGTGGAGAAGTACTGAAGATATTCTTCTTAAATTT  4262
Wild           TACCCTTAAGGCTAGCGTATTTCCTAGTGGAGAAGTACTGAAGATATTCTTCTTAAATTT  4081
Domestic       TACCCTTAAGGCTAGCGTATTTCCTAGTGGAGAAGTACTGAAGATATTCTTCTTAAATTT  4500
               **************** *******************************************

Dromedary      AGGAACTGGACAAAGATGTCCAAAATGACCACTACTACTTCCCAGTGTCTAAATGATTGC  4322
Wild           AGGAACTGGACAAAGATGTCCAAAATGACCACTACTACTTCCCAGTGTCTAAATGATTGC  4141
Domestic       AGGAACTGGACAAAGATGTCCAAAATGACCACTACTACTTCCCAGTGTCTAAATGATTGC  4560
               ************************************************************

Dromedary      CCAGTATCTCCAAAACTATAGAAAATTAAAGGGATAAAAATTGAAAAAAAAATTGAATTA  4382
Wild           CCAGTATCTCCAAAACTATAGAAAATTAAAGGGATAAAAATTGAAAAAAAAATTGAATTA  4201
Domestic       CCAGTATCTCCAAAACTATAGAAAATTAAAGGGATAAAAATTGAAAAAAAAATTGAATTA  4620
               ************************************************************

Dromedary      TATCTGTATTTTCTGACGGTTTGACTGCATACCTGAAAAATCCAAGAGATAGCAAATGAA  4442
Wild           TATCTGTATTTTCTGACGGTTTGACTGCATACCTGAAAAATCCAAGAGATAGCAAATGAA  4261
Domestic       TATCTGTATTTTCTGACGGTTTGACTGCATACCTGAAAAATCCAAGAGATAGCAAATGAA  4680
               ************************************************************

Dromedary      ACTGATACTAATAAGAGAATTCATCAAGGTAAAAGGATATCAAATTATTAATATACAGAA  4502
Wild           ACTGATACTAATAAGAGAATTCATCAAGGTAAAAGGATATCAAATTATTAATATACAGAA  4321
Domestic       ACTGATACTAATAAGAGAATTCATCAAGGTAAAAGGATATCAAATTATTAATATACAGAA  4740
               ************************************************************

Dromedary      ATTAATCAATTAACATAAAAAACAATGACAAAGTAATGGGATAAACAGACCCCATTTCAA  4562
Wild           ATTAATCAATTAACATAAAAAACAATGACAAAGTAATGGGATAAACAGACCCCATTTCAA  4381
Domestic       ATTAATCAATTAACATAAAAAACAATGACAAAGTAATGGGATAAACAGACCCCATTTCAA  4800
               ************************************************************

Dromedary      TAACAACAAAAGACAAAATATTCAGGACTTAATAATAAATATCCAGAACCTAAAGGAGAA  4622
Wild           TAACAACAAAAGACAAAATATTCAGGACTTAATAATAAATATCCAGAACCTAAAGGAGAA  4441
Domestic       TAACAACAAAAGACAAAATATTCAGGACTTAATAATAAATATCCAGAACCTAAAGGAGAA  4860
               ************************************************************

Dromedary      TCTTTCTAATACAAAGAATATAAAAGTTAACTTGGGAGGATCTAAAGAAAAAAGAACGCT  4682
Wild           TCTTTCTAATACAAAGAATATAAAAGTTAACTTGGGAGGATGTAAAGAAAAAAGAACGCT  4501
Domestic       TCTTTCTAATACAAAGAATATAAAAGTTAACTTGGGAGGATGTAAAGAAAAAAGAACGCT  4920
               ***************************************** ******************

Dromedary      TATGCACTCCTGGTGGGAATGTAAACTGATGTAGCCACTATGGAAAACAGTATGGAGATT  4742
Wild           TATGCACTGCTGGTGGGAATGTAAACTGATGTAGCCACTATGGAAAACAGTATGGAGATT  4561
Domestic       TATGCACTGCTGGTGGGAATGTAAACTGATGTAGCCACTATGGAAAACAGTATGGAGATT  4980
               ******** ***************************************************

Dromedary      CCTCAAAAAATTAAAAATAGAACTACCTTCCCATCCAGCAATTCCACTTCTGGGTATTTT  4802
Wild           CCTCAAAAAATTAAAAATAGAACTACCTTCCCATCCAGCAATTCCACTTCTGGGTATTTT  4621
Domestic       CCTCAAAAAATTAAAAATAGAACTACCTTCCCATCCAGCAATTCCACTTCTGGGTATTTT  5040
               ************************************************************

Dromedary      TCCAAAGGAAACAAAAGCACTAACTCGAAAAGATATCCCCATGTTCATAGCAGCATTATT  4862
Wild           TCCAAAGGAAACAAAAGCACTAACTCGAAAAGATATCCCCATGTTCATAGCAGCATTATT  4681
Domestic       TCCAAAGGAAACAAAAGCACTAACTCGAAAAGATATCCCCATGTTCATAGCAGCATTATT  5100
               ************************************************************

Dromedary      CACAAGAGCCAAGATATGGAAACAACCAAAGTGTCCATCAGTGAATGAATGGATAAAGAA  4922
Wild           CACAAGAGCCAAGATACGGAAACAGCCAAAGTGTCCATCAGTGAATGAATGGATAAAGAA  4741
Domestic       CACAAGAGCCAAGATACGGAAACAGCCAAAGTGTCCATCAGTGAATGAATGGATAAAGAA  5160
               **************** ******* ***********************************

Dromedary      ATTGTGGCATATTTATATTTATATTCAAGTTCATATTTATATAATTCAGTGTAAAAGGAG  4982
Wild           ATTGTGGCATATTTATATTTATATTCAAGTTCATATTTATATAATTCAGTGTAAAAGGAG  4801
Domestic       ATTGTGGCATATTTATATTTATATTCAAGTTCATATTTATATAATTCAGTGTAAAAGGAG  5220
               ************************************************************

Dromedary      GAAATCTTGCCATTTGGGCCTTGAGGGCATTATGCTGTGTGAAATAACTCAGAGAAAGAC  5042
Wild           GAAATCTTGCCATTTGGGCCTTGAGGGCATTATGCTGTGTGAAATAACTCAGAGAAAGAC  4861
Domestic       GAAATCTTGCCATTTGGGCCTTGAGGGCATTATGCTGTGTGAAATAACTCAGAGAAAGAC  5280
               ************************************************************
```

```
Dromedary      AAATATTGTATGATCTTACTTACACGTGGAGCCTAAAAAAAAAAAAACTGATCTCATAGA  5102
Wild           AAATATTGTATGACCTTACTTACACGTGGAGCCT-------AAAAAACTGATCTCATAGA  4914
Domestic       AAATATTGTATGACCTTACTTACACGTGGAGCCT----AAAAAAAAACTGATCTCATAGA  5336
               ************* ********************       *******************

Dromedary      CACAGAGAACAGATTGGTGATTGCCAGAGGTAGGGAGTTGGGGTTAGGGGCAAATGGGTG  5162
Wild           CACAGAGAACAGATTGGTGATTGCCAGAGGTAGGGAGTTGGGGTTAGGGGCAAATGGGTG  4974
Domestic       CACAGAGAACAGATTGGTGATTGCCAGAGGTAGGGAGTTGGGGTTAGGGGCAAATGGGTG  5396
               ************************************************************

Dromedary      AAGGAGGTCAAAGATGCAAACTTCTAGTTACAAAATAAGCCTTGGGGATGTAATATACAG  5222
Wild           AAGGAGGTCAAAGATGCAAACTTCTAGTTACAAAATAAGCCTTGGGGATGTAATATACAG  5034
Domestic       AAGGAGGTCAAAGATGCAAACTTCTAGTTACAAAATAAGCCTTGGGGATGTAATATACAG  5456
               ************************************************************

Dromedary      CATGGCAACTATACTTAATACTGTTTTGTATATTTGAAAGTTGCTGAGAAAGTTCAGTGA  5282
Wild           CATGGCAACTATATTTAATACTGTTTTGTATATTTGAAAGTTGCTGAGAAAGTTCAGTGA  5094
Domestic       CATGGCAACTATATTTAATACTGTTTTGTATATTTGAAAGTTGCTGAGAAAGTTCAGTGA  5516
               ************* **********************************************

Dromedary      TAGATATTAACTAGATTTATTGTGATGATCATTTCACAGTATAGACATATACTGAATCAT  5342
Wild           TAGATATTAACTAGATTTATTGTGATGATCATTTCACGGTATAGACATATACTGAATCAT  5154
Domestic       TAGATATTAACTAGATTTATTGTGATGATCATTTCACAGTATAGACGTATACTGAATCAT  5576
               ************************************* ******** *************

Dromedary      TATGCTGTACACCTGAAACTGATATAATGTTACATGTCAATTATATCACAGTAAAATTTT  5402
Wild           TATGCTGTACACCTGAAACTGATATAATGTTACATGTCAATTATATCACAGTAAAATTTT  5214
Domestic       TATGCTGTACACCTGAAACTGATATAATGTTACATGTCAATTATATCACAGTAAAATTTT  5636
               ************************************************************

Dromedary      TTTAAGTTAACTTGGATAAATGTAAAGATATACTGTATTCTTTAATAGGAAGACTCAATA  5462
Wild           TTTAAGTTAACTTGGATAAATGTAAAGATATACTGTATTCTTTAATAGGAAGACTCAATA  5274
Domestic       TTTAAGTTAACTTGGATAAATGTAAAGATATACTGTATTCTTTAATAGGAAGACTCAATA  5696
               ************************************************************

Dromedary      TCATAAAGCAGTATGTTACATTTATAAATTATTAAGGAATAATCATAATAAAAATACAGG  5522
Wild           TCATAAAGCAGTATGTTACATTTATAAATTATTAAGGAATAATCATAATAAAAATACAGG  5334
Domestic       TCATAAAGCAGTATGTTACATTTATAAATTATTAAGGAATAATCATAATAAAAATACAGG  5756
               ************************************************************

Dromedary      TTATTTATTAGAATTAGATAAACAAATTTTAAAGTTCTCATGAGAAAAATAAGTGAGCAA  5582
Wild           TTATTTATTAGAATTAGATAAACAAATTTTAAAGTTCTCATGAGAAAAATAAGTGAGCAA  5394
Domestic       TTATTTATTAGAATTAGATAAACAAATTTTAAAGTTCTCATGAGAAAAATAAGTGAGCAA  5816
               ************************************************************

Dromedary      AGAAGAAACACCTGGAACATTTTGACAAAGAGCAGTATGGGTACTACTCCTACCAGATAT  5642
Wild           AGAAGAAACACCTGGAACATTTTGACAAAGAGCAGTATGGGTACTACTCCTACCAGATAT  5454
Domestic       AGAAGAAACACCTGGAACATTTTGACAAAGAGCAGTATGGGTACTACTCCTACCAGATAT  5876
               ************************************************************

Dromedary      GCAAACATGCTTTAATGCAGCAATAATTCAAAGTGTAGGATTGGCTCATGAAAAGAGAGA  5702
Wild           GCAAACATGCTTTAATGCAGCAATAATTCAAAGTGTAGGATTGGCTCATGAAAAGAGAGA  5514
Domestic       GCAAACATGCTTTAATGCAGCAATAATTCAAAGTGTAGGATTGGCTCATGAAAAGAGAGA  5936
               ************************************************************

Dromedary      CTCATCAAACAGAATAGAAAGTCCAGAGATAGGCTTGAATACATACGGGAATTCAGTGCA  5762
Wild           CTCATCAAACAGAATAGAAAGTCCAGAGATAGGCTTGAATACATATGGGAATTCAGTGCA  5574
Domestic       CTCATCAAACAGAATAGAAAGTCCAGAGATAGGCTTGAATACATATGGGAATTCAGTGCA  5996
               ********************************************* **************

Dromedary      CAATGAAGACTGTAGCCAAGTCAAACACTAAAGACAGACTTTTTGGTAAGTAGTACTGGG  5822
Wild           CAATGAAGACTGTAGCCAAGTCAAACACTAAAGACAGACTTTTTGGTAAGTAGTACTGGG  5634
Domestic       CAATGAAGACTGTAGCCAAGTCAAACACTAAAGACAGACTTTTTGGTAAGTAGTACTGGG  6056
               ************************************************************

Dromedary      GAAACTGGGTAGCAAGTTGGAAAAAAATAAAGCAAATTCCATATTGACGTTATTATCAGA  5882
Wild           GAAACTGGGTAGCAAGTTGGAAAAAAATAAAGCAAATTCCATATTGACGTT--------A  5686
Domestic       GAAACTGGGTAGCAAGTTGGAAAAAAATAAAGCAGATTCCATATTGACGTT--------A  6108
               ********************************** ****************        *
```

```
Dromedary      TTAAACAAATAGGTCCAAATGGATCAAAGATTTAAATGTAATAAAACAAAACTATAGAAG  5942
Wild           TTAAACAAATAGGTCCAAATGGATCAAAGATTTAAATGTAATAAAACAAAACTATAGAAG  5746
Domestic       TTAAACAAATAGGTCCAAATGGATCAAAGATTTAAATGTAATAAAACAAAACTATAGAAG  6168
               ************************************************************

Dromedary      TGTTAGAAAACAACATAAGTGGTTTTTATTGTAAACTTGGGAAGTTATTTTATTATAAAC  6002
Wild           TGTTAGAAAACAACATAAGTGGTTTTTATTGTAAACTTGGGAAGTTATTTTATTATAAAC  5806
Domestic       TGTTAGAAAACAACATAAGTGGTTTTTATTGTAAACTTGGGAAGTTATTTTATTATAAAC  6228
               ************************************************************

Dromedary      GTTCTAACTATGACTCAAATTTTGGAAAGGCTAATAGTTAAAAGTAAACACAAACACTTT  6062
Wild           GTTCTAACTATGACTCAAATTTTGGAAAGGCTAATAGTTAAAAGTAAACACAAACACTTC  5866
Domestic       GTTCTAACTATGACTCAAATTTTGGAAAGGCTAATAGTTAAAAGTAAACACAAACACTTC  6288
               ***********************************************************

Dromedary      GGCATGTCAGAAATACGCCATTAGCAGAGTGAAAACAAATAAGATAAAATAAGACATTTG  6122
Wild           GGCATGTCAGAAATATGCCATTAGCAGAGTGAAAACAAATAAGATAAAATAAGACATTTG  5926
Domestic       GGCATGTCAGAAATATGCCATTAGCAGAGTGAAAACAAATAAGATAAAATAAGACATTTG  6348
               *************** ********************************************

Dromedary      CAACTCATAGCACAGACTGCTTACATTCCCAAAATACATAAAAACTTCTAGAAATAGAGG  6182
Wild           CAACTCATAGCACAGACTGCTTACATTCCCAAAATACATAAAAACTTCTAGAAATAGAGG  5986
Domestic       CAACTCATAGCACAGACTGCTTACATTCCCAAAATACATAAAAACTTCTAGAAATAGAGG  6408
               ************************************************************

Dromedary      AGAAAAAGACTAACTGTATAGAAAAATAAGCCAAAGATAATGGGCAGGTTATAGGAAAAA  6242
Wild           AGAAAAAGACTAACTGTATAGAAAAATAAGCCAAAGATAATGGGCAGGTTATAGGAAAAA  6046
Domestic       AGAAAAAGACTAACTGTATAGAAAAATAAGCCAAAGATAATGGGCAGGTTATAGGAAAAA  6468
               ************************************************************

Dromedary      ATCCAATAACAAATAGCCACTAAACACATGAAATGGTGCTAACTTCACTAGTAAAAATGC  6302
Wild           ATCCAATAACAAATAGCCTCTAAACACATGAAATGGTGCTAACTTCACTAGTAAAAATGC  6106
Domestic       ATCCAATAACAAATAGCCACTAAACACATGAAATGGTGCTAACTTCACTAGTAAAAATGC  6528
               ****************** *****************************************

Dromedary      AAATTAATATTATACTGATATATAATTCTTCAACTATTGGATTGGCAAAATCCAAAAGTC  6362
Wild           AAATTAATATTATACTGAGATATAATTCTTCAACTATTGGATTGGCAAAATCCAAAAGTC  6166
Domestic       AAATTAATATTATACTGAGATATAATTCTTCAACTATTGGATTGGCAAAATCCAAAAGTC  6588
               ****************** *****************************************

Dromedary      CTATGACAAACTGTTGGAGAAGCTTTGGGGAAATGGACACTTTCAAACATTGCTGGTAAG  6422
Wild           CTATGACAAACTGTTGGAGAAGCTATGGGGAAATGGACACTTTCAAACATTGCTGGTAAG  6226
Domestic       CTATGACAAACTGTTGGAGAAGCTATGGGGAAATGGACACTTTCAAACATTGCTGGTAAG  6648
               ************************ ***********************************

Dromedary      AGTAGAAGATGGAATAACCATCAGGAGGGAATTTTTGGAAATAACTACCAAAATTTCAGA  6482
Wild           AGTAGAAGATGGAATAACCATCAGGAGGGAATTTTTGGAAATAACTACCAAAATTTCAGA  6286
Domestic       AGTAGAAGATGGAATAACCATCAGGAGGGAATTTTTGGAAATAACTACCAAAATTTCAGA  6708
               ************************************************************

Dromedary      TGCATTTACTCATGATGTAATAATAATCTAATTCCTAGGAATCTCATACATATAAGTGTA  6542
Wild           TGCATTTACTCATGATGTAATAATAATCTAATTCCTAGGAATCTCATACATATAAATGTA  6346
Domestic       TGCATTTACTCATGATGTAATAATAATCTAATTCCTAGGAATCTCATACATATAAATGTA  6768
               ******************************************************* ****

Dromedary      TATGTATGTATATACACTGAATTAGACACAGATATCATATATAACATACATATAATGTAT  6602
Wild           TATGTATGTATATACACTGAATTAGACACAGAT---ATATATAACATACATATAATGTAT  6403
Domestic       TATGTATGTATATACACTGAATTAGACACAGAT---ATATATAACATACATATAATGTAT  6825
               *********************************   ************************

Dromedary      GTGCCTATCTATATATCTGTGCTAACATGAAATGACAGTTGTTCAAGGTTATCCTTTGCA  6662
Wild           GTGGCTATCAATATATCTGTGCTAACATGAAATGACAGTTGTTCAAGGTTATCTTTTGCA  6463
Domestic       GTGGCTATCAATATATCTGTGCTAACATGAAATGACAGTTGTTCAAGGTTATCTTTTGCA  6885
               *** ***** ******************************************* ******

Dromedary      GGATTGTTTGTAAAAGAAAAGGACTGGAACACAGTATCCAGCATTAGGGGACTGGTTGAA  6722
Wild           GGATTGTTTGTAAAAGAAAAGGACTGGAACACAGTACCCAGCATTAGGGGACTGGTTGAA  6523
Domestic       GGATTGTTTGTAAAAGAAAAGGACTGGAACACAGTATCCAGCATTAGGGGACTGGTTGAA  6945
               *********************************** ************************
```

```
Dromedary     TAAATCACAGTAAAACCATACAATCAAAAGCTATGCACCTACAAGAAAGATTAAAGAAGA  6782
Wild          TAAATCACAGTAAAACCATACAATCAAAAGCTATGCACCTACAAGAAAGATTAAAGAAGA  6583
Domestic      TAAATCACAGTAAAACCATACAATCAAAAGCTATGCACCTACAAGAAAGATTAAAGAAGA  7005
              ************************************************************

Dromedary     TCTCTGTCAACTACTGACGTGGAGGCATCTCTAGGATAAATTTTCATGCTTAAAAAAAAG  6842
Wild          TCTCTGTCAACTACTGACGTGGAGGCATCTCTAGGATAAATTTTCATGCTTAAAAAAAAG  6643
Domestic      TCTCTGTCAACTACTGACGTGGAGGCATCTCTAGGATAAATTTTCATGCTTAAAAAAAAG  7065
              ************************************************************

Dromedary     CAAATAATAAATGTACATTTTAGGCCATCTTTTATGTAAGAATACATTGATGCTTACTTG  6902
Wild          CAAAGAATAAATGTACATTTTAGGCCATCTTTTATGTAAGAATACATTGATGCTTACTTG  6703
Domestic      CAAAGAATAAATGTACATTTTAGGCCATCTTTTATGTAAGAATACATTGATGCTTACTTG  7125
              **** *******************************************************

Dromedary     TATTTGCATAAAGAAACACTGAAAGGACAAAAAATAAACTAATAAAATGGTAATCAATAC  6962
Wild          TATTTGCATAAAGAAACACTGAAAGGACAAAAAATAAACTAATAAAATGGTAATCAATAC  6763
Domestic      TATTTGCATAAAGAAACACTGAAAGGACAAAAAATAAACTAATAAAATGGTAATCAATAC  7185
              ************************************************************

Dromedary     CAGCAAGAGGAAAAAGGGTGAAAAGGCACAGGTAGGAATGAGATTTCTATGTATTCCTTT  7022
Wild          CAGCAAGAGGAAAAAGGGTGAAAAGGCACAGGTAGGAATGAGATTTCTATGTATTCCTTT  6823
Domestic      CAGCAAGAGGAAAAAGGGTGAAAAGGCACAGGTAGGAATGAGATTTCTATGTATTCCTTT  7245
              ************************************************************

Dromedary     TACATATATATTGATACTTGAATCAAAAAATTAAAGTTAATAAAAATATCAAGTTTTCTT  7082
Wild          TACATATATATTGATATTTGAATCAAAAAATTAAAGTTAATAAAAATATCAAGTTTTCTT  6883
Domestic      TACATATATATTGATATTTGAATCAAAAAATTAAAGTTAATAAAAATATCAAGTTTTCTT  7305
              **************** *******************************************

Dromedary     AGAATCTGATGCCAGTTTTAGAGCAAAGCTATCTGGATAAACTTTAGCAAAGTAATGCAG  7142
Wild          AGAATCTGATGCCAGTTTTAGAGCAAAGCTATCTGGATAAACTTTAGCAAAGTAATGCAG  6943
Domestic      AGAATCTGATGCCAGTTTTAGAGCAAAGCTATCTGGATAAACTTTAGCAAAGTAATGCAG  7365
              ************************************************************

Dromedary     TTTTAGATGATTGTATAAGGCAATTGTCCATCTTTTACTTCCAATTAGGTATTTTACTTT  7202
Wild          TTTTAGATGATTGTATAAGGCAATTGTCCATCTCTTACTTCCAATTAGGTATTTTACTTT  7003
Domestic      TTTTAGATGATTGTATAAGGCAATTGTCCATCTCTTACTTCCAATTAGGTATTTTACTTT  7425
              ********************************* **************************

Dromedary     TCAGTGGAACTGAGAACTCAAAAAACTTGATGAAATCATGTGTTGATGGTATCTTTTATG  7262
Wild          TCAGTGGAACTGAGAACTCAAAAAACTTGATGAAATCATGTGTTGATGGTATCTTTTATG  7063
Domestic      TCAGTGGAACTGAGAACTCAAAAAACTTGATGAAATCATGTGTTGATGGTATCTTTTATG  7485
              ************************************************************

Dromedary     CCAGGTACTGAGGACCCAGTAAGAATACATGTTCTTGCCCTCAAGGAGTTCAGACTTATG  7322
Wild          CCAGGTACTGAGGACCCAGTAAGAATACGTGTTCTTGCCCTCAAGGAGTTCAGACTTATG  7123
Domestic      CCAGGTACTGAGGACCCAGTAAGAATACGTGTTCTTGCCCTCAAGGAGTTCAGACTTATG  7545
              **************************** *******************************

Dromedary     GGGAGACCAAAAATAAGCATGTGATTACCAGCATAAGAATACTAAGCACTACACACAATA  7382
Wild          GGGAGACCAAAAATAAGCATGTGATTACCAGCATAAGAATACTAAGCACTACACACAATA  7183
Domestic      GGGAGACCAAAAATAAGCATGTGATTACCAGCATAAGAATACTAAGCACTACACACAATA  7605
              ************************************************************

Dromedary     TGTAAAGTGATTAAGTGCCTTCTGCTGCCGAGGAAGACTTCCTAGAAGAGGTGAGAGCCA  7442
Wild          TGTAAAGTGATTAAGTGCCTTCTGCTGCCGAGGAAGACTTCCTAGAAGAGGTGAGAGCCA  7243
Domestic      TGTAAAGTGATTAAGTGCCTTCTGCTGCCGAGGAAGACTTCCTAGAAGAGGTGAGAGCCA  7665
              ************************************************************

Dromedary     AGCTAGGCTTAAAGGATGACTAGGAGCTTGAAGGTGACCCCGGGCAATGTGGGCATTTCA  7502
Wild          AGCTAGGCTTAAAGGATGACTAGGAGCTTGAAGGTGACCCCAGGCAATGTGGGCATTTCA  7303
Domestic      AGCTAGGCTTAAAGGATGACTAGGAGCTTGAAGGTGACCCCAGGCAATGTGGGCATTTCA  7725
              ***************************************** ******************

Dromedary     AGCACAGGCAACAGCAAGTGCCACGATCAGTGCTGCTTTCCTATTCTTACAGGATAAACT  7562
Wild          AGCACAGGCAACAGCAAGTGCCACGATCAGTGCTGCTTTCCTATTCTTACAGGATAAACT  7363
Domestic      AGCACAGGCAACAGCAAGTGCCACGATCAGTGCTGCTTTCCTATTCTTACAGGATAAACT  7785
              ************************************************************
```

```
Dromedary      TCAAGTTCCTCAGGCTCAAAGCTGCCCTCCGCAGCCTGGCTCCTGCCTCTCTCTCCAGCT  7622
Wild           TCAAGTTCCTCAGGCTCAAAGCTGCCCTCCGCAGCCTGGCTCCTGCCTCTCTCTCCAGCT  7423
Domestic       TCAAGTTCCTCAGGCTCAAAGCTGCCCTCCGCAGCCTGGCTCCTGCCTCTCTCTCCAGCT  7845
               ************************************************************

Dromedary      GCGTCTTCTGCTCTGGAGGCCTAGACATGTGTAGGTGTGGTAGGAACTCGAAAGATAGAA  7682
Wild           GCGTCTTCTGCTCTGGAGGCCTAGACATGTGTAGGTGTGGTAGGAACTCGAAAGATAGAA  7483
Domestic       GCGTCTTCTGCTCTGGAGGCCTAGACATGTGTAGGTGTGGTAGGAACTCGAAAGATAGAA  7905
               ************************************************************

Dromedary      GCTGTGGTCAGAGAAGTTTACTACCATAGAAGAGTCCTCCCACAGGGCGGCCCCTGGTCT  7742
Wild           GCTGTGGTCAGAGAAGTTTACTACCATAGAAGAGTCCTCCCACAGGGTGGCCCCTGGTCT  7543
Domestic       GCTGTGGTCAGAGAAGTTTACTACCATAGAAGAGTCCTCCCACAGGGTGGCCCCTGGTCT  7965
               *********************************************** ************

Dromedary      AGAGGAGCTGCCCAGTGTCCCCTGGAGTTGCCCATGTCATGTCAGGAGCTCTGATGTAGA  7802
Wild           AGAGGAGCTGCCCAGTGTCCCCTGGAGTTGCCCATGTCATGTCAGGAGCTCTGATGTAGA  7603
Domestic       AGAGGAGCTGCCCAGTGTCCCCTGGAGTTGCCCATGTCATGTCAGGAGCTCTGATGTAGA  8025
               ************************************************************

Dromedary      TGTGGTGGCTGAGCTAGTCAAAGGGAGGTAGCAGGAGTTTGGGATAGAGAGGCAAATTAT  7862
Wild           TGTGGTGGCTGAGCTAGTCAAAGGGAGGTAGCAGGAGTTTGGGATAGAGAGGCAAACTAT  7663
Domestic       TGTGGTGGCTGAGCTAGTCAAAGGGAGGTAGCAGGAGTTTGGGATAGAGAGGCAAACTAT  8085
               ******************************************************** ***

Dromedary      GACCTGGTGGGGACAACACTCAATAAATACTGGAGAGCAAGGCCTACGTGAATATAACTT  7922
Wild           GACCTGGTGGGGACAACACTCAATAAATACTGGAGAGCAAGGCCTACGTGAATATAACTT  7723
Domestic       GACCTGGTGGGGACAACACTCAATAAATACTGGAGAGCAAGGCCTACGTGAATATAACTT  8145
               ************************************************************

Dromedary      CAATGGCCAAGTAAGCACTGGGTTCCAAATGCTGTCCTCGGAGACATCTTTTAAATGCGT  7982
Wild           CAATGGCCAAGTAAGCACTGGGTTCCAAATGCTGTCCTCGGAGACATCTTTTAAATGCAT  7783
Domestic       CAATGGCCAAGTAAGCACTGGGTTCCAAATGCTGTCCTCGGAGACATCTTTTAAACGCAT  8205
               ******************************************************* ** *

Dromedary      AAGTCATGTTACGCCCTTGCTTAAAAGCACTGCTTCTCATTGCATTTATGATGCAATTAA  8042
Wild           AAGTCATGTTACGCCCTTGCTTAAAAGCACTGCTTCTCATTGCATTTATGATGCAATTAA  7843
Domestic       AAGTCATGTTACGCCCTTGCTTAAAAGCACTGCTTCTCATTGCATTTATGATGCAATTAA  8265
               ************************************************************

Dromedary      AACTCCTTCATCTGGCTTTCATATTCAGGTCTCACAAATTCTGTCCCCAGCCTACACCTC  8102
Wild           AACTCCTTCATCTGGCTTTCATATTCAGGTCCCACAAATTCTGTCCCCAGCCTACACCTC  7903
Domestic       AACTCCTTCATCTGGCTTTCATATTCAGGTCCCACAAATTCTGTCCCCAGCCTACACCTC  8325
               ******************************* ****************************

Dromedary      TGACCATGCTCTGTGACACCCTCTCCCTCTCATAAGGCCCCTCTGCAGTCTCTGCCATGCC  8162
Wild           TGACCATGCTCTGTGACACCCTCTCCCTCTCATAAGGCCCCTCTGCAGTCTCTGCCATGCC  7963
Domestic       TGACCATGCTCTGTGACACCCTCTCCCTCTCATAAGGCCCCTCTGCAGTCTCTGCCATGCC  8385
               ************************************************************

Dromedary      CTCTCCATCCCTTGCTCCCTTGCCTAAATCCTACTTCTGCTATTCTTATTTTAAATGTCA  8222
Wild           CTCTCCATCCCTTGCTCCCTTGCCTAAATCCTACTTCTGCTATTCTTATTTTAAATGTCA  8023
Domestic       CTCTCCATCCCTTGCTCCCTTGCCTAAATCCTACTTCTGCTATTCTTATTTTAAATGTCA  8445
               ************************************************************

Dromedary      CTCCTCAGGCCTTGCCCCCTGACTACCTCTCCACCATCTGAATTATACGTCTAATTAAAT  8282
Wild           CTCCTCAGGCCTTGCCCCCTGCCTACCTCTCCACCATCTGAATTATACGTCTAATTAAAT  8083
Domestic       CTCCTCAGGCCTTGCCCCCTGCCTACCTCTCCACCATCTGAATTATACGTCTAATTAAAT  8505
               ********************* **************************************

Dromedary      TTTTGCTACAGCACCAAAACATGTGTCTAAATCCAAATCCCATGATCTTAAATGCTATCT  8342
Wild           TTTGGCTACAGCACCAAAACATGTGTCTAAATCCAAATCCCATGATCTTAAATGCTATCT  8143
Domestic       TTTGGCTACAGCACCAAAACATGTGTCTAAATCCAAATCCCATGATCTTAAATGCTATCT  8565
               *** ********************************************************

Dromedary      TGTCTCCCAAATGAAGAAAACGTTCTCGTGAAAATAAAAAAGAAAGGTCTGGAAGTGGTC  8402
Wild           TGTCTCCCAAATGAAGAAAACGTTCTCGTGAAAATAAAAAAGAAAGGTCTGGAAGTGGTC  8203
Domestic       TGTCTCCCAAATGAAGAAAACGTTCTCGTGAAAATAAAAAAGAAAGGTCTGGAAGTGGTC  8625
               ************************************************************
```

```
Dromedary     AAGGTGGCTAAAATAGGCGGCAGCCTGTCTGCTGGCTCTGGCTTATGAGGTTCTGGTTAT  8462
Wild          AAGGTGGCTAAAATAGGCGGCAGCCTGTCTGCTGGCTCTGGCTTATGAGGTTCTGGTTAT  8263
Domestic      AAGGTGGCTAAAATAGGCGGCAGCCTGTCTGCTGGCTCTGGCTTATGAGGTTCTGGTTAT  8685
              ************************************************************

Dromedary     GGACAAAGAGCAGGCTCTGTCTGAGGAGACTGAAATGAATAAGGTCCTTGGGGTTTCAAC  8522
Wild          GGACAAAGAGCAGGCTCTGTCTGAGGAGACTGAAATGAATAAGGTCCTTGGGGTTTCAAC  8323
Domestic      GGACAAAGAGCAGGCTCTGTCTGAGGAGACTGAAATGAATAAGGTCCTTGGGGTTTCAAC  8745
              ************************************************************

Dromedary     AAAATAAAGATGCGATGGAATATTCTGTAAACAGGTCGAGGGAAGTGGCTTTGCAAAGGA  8582
Wild          AAAATAAAGATGCGATGGAATATTCTGTAAACAGGTCGAGGGAAGTGGCTTTGCAAAGGA  8383
Domestic      AAAATAAAGATGCGATGGAATATTCTGTAAACAGGTCGAGGGAAGTGGCTTTGCAAAGGA  8805
              ************************************************************

Dromedary     ATATGCTTTCAGAGGATCCTGCAGAACTGAATTAAGAGACTGGACAGCAGTAAGAAAACG  8642
Wild          ATATGCTTTCAGAGGATCCTGCAGAACTGAATTAAGAGACTGGACAGCAGTAAGAAAACG  8443
Domestic      ATATGCTTTCAGAGGATCCTGCAGAACTGAATTAAGAGACTGGACAGCAGTAAGAAAACG  8865
              ************************************************************

Dromedary     AGCAAGGAAAGAGGGTGAGACTTGTAAGCAGCCACAGGGATCCAACAATGAATGGATGGT  8702
Wild          AGCAAGGAAAGAGGGTGAGACTTGTAAGCAGCCACAGGGATCCAACAATGAATGGATGGT  8503
Domestic      AGCAAGGAAAGAGGGTGAGACTTGTAAGCAGCCACAGGGATCCAACAATGAATGGATGGT  8925
              ************************************************************

Dromedary     TGACTGGATGCTGTGAATATTTTGGGAAGTATTGGGGGACTCTGGGAACAAGTAGCTTGG  8762
Wild          TGACTGGATGCTGTGAATATTTTGGGAAGTATTGGGGGACTCTGGGAACAAATAGCTTGG  8563
Domestic      TGACTGGATGCTGTGAATATTTTGGGAAGTATTGGGGGACTCTGGGAACAAGTAGCTTGG  8985
              *************************************************** ********

Dromedary     ACAGGAATATCAGGAAGTACTCAACCATTGTGACGCTACAGTATTGACTACATTCCTTAA  8822
Wild          ACAGGAATATCAGGAAGTACTCAACCAGTGTGACGCTACAGTATTGACTACATTCCTTAA  8623
Domestic      ACAGGAATATCAGGAAGTACTCAACCAGTGTGACGCTACAGTATTGACTACATTCCTTAA  9045
              *************************** ********************************

Dromedary     GTTTTCTGAGCACTATTCTCCCTGGAGAGGCTTCTCCACTAACATGGCCTCTCTGTCCCT  8882
Wild          GTTTTCTGAGCACTATTCTCCCTGGAGAGGCTTCTCCACTAACATGGCCTCTCTGTCCCT  8683
Domestic      GTTTTCTGAGCACTATTCTCCCTGGAGAGGCTTCTCCACTAACATGGCCTCTCTGTCCCT  9105
              ************************************************************

Dromedary     CATCAAGTTCTCGCAAAACCCCTAGTCCTCTGGAAACTCCTGCTTCACTTCTATTTTCTG  8942
Wild          CATCAAGTTCTCGCAAAACCCCTAGTCCTCTGGAAACTCCTGCTTCACTTCTATTTTCTG  8743
Domestic      CATCAAGTTCTCGCAAAACCCCTAGTCCTCTGGAAACTCCTGCTTCACTTCTATTTTCTG  9165
              ************************************************************

Dromedary     AGCTCTATCCTGCAGAAAGGGCCAGAAGCAAGAAAATAAATCATGATCAGTCTTGGAAGT  9002
Wild          AGCTCTATCCTGCAGAAAGGGCCAGAAGCAAGAAAATAAATCATGATCAGTCTTGGAAGT  8803
Domestic      AGCTCTATCCTGCAGAAAGGGCCAGAAGCAAGAAAATAAATCATGATCAGTCTTGGAAGT  9225
              ************************************************************

Dromedary     AGAAATAGTGACTACACAAGAAGGCCAGGGATGGGTTTTTCAGGAGAGTTAAAAGTTGAA  9062
Wild          AGAAATAGTGACTACACAAGAAGGCCAGGGATGGGTTTTTCAGGAGAGTTAAAAGTTGAA  8863
Domestic      AGAAATAGTGACTACACAAGAAGGCCAGGGATGGGTTTTTCAGGAGAGTTAAAAGTTGAA  9285
              ************************************************************

Dromedary     AGTTTCTGGGGTATATTTAATAGCACATCTTGTGCTCTCAGAAGTGGGTGTAAATCGGGG  9122
Wild          AGTTTCTGGGGTATATTTAATAGCACATCTTGTGCTCTCAGAAGTGGGTGTAAATCGGGG  8923
Domestic      AGTTTCTGGGGTATATTTAATAGCACATCTTGTGCTCTCAGAAGTGGGTGTAAATCGGGG  9345
              ************************************************************

Dromedary     TACAGTCCTAGACCACCCAAGAATGAGGCAGGCCTACAAGGAACAGACATGAATGCTGTT  9182
Wild          TACAGTCCTAGACCACCCAAGAATGAGGCAGGCCTACAAGGAACAGACATGAATGCTGTT  8983
Domestic      TACAGTCCTAGACCACCCAAGAATGAGGCAGGCCTACAAGGAACAGACATGAATGCTGTT  9405
              ************************************************************

Dromedary     TAAAATCACTAAAGCTCCTGACTTCAGACATATTCTATTGTAGTCAAAAATCTATACATT  9242
Wild          TAAAATCACTAAAGCTCCTGACTGCAGACATATTCTATTGTAGTCAAAAATCTATACATT  9043
Domestic      TAAAATCACTAAAGCTCCTGACTGCAGACATATTCTATTGTAGTCAAAAATCTATACATT  9465
              *********************** ************************************
```

```
Dromedary      TATATTATATTCAGAGATAGATAAAATTAGTGATATTAGGGTTATTGTGTCAGTCATTAA  9302
Wild           TATATTATATTCAGAGATACATAAAATTAGTGATATTAGGGTTATTGTGTCAGTCATTAA  9103
Domestic       TATATTATATTCAGAGATACATAAAATTAGTGATATTAGGGTTATTGTGTCAGTCATTAA  9525
               ******************* ****************************************

Dromedary      AAAGCAAGATATTTAGTATTAAGAATTGCTAAGGAAAACTCTACCTTAAAACTGTAATCC  9362
Wild           AAAGCAAGATATTTAGTATTAAGAATTGCTAAGGAAAACTCTACCTTAAAACTGTAATCC  9163
Domestic       AAAGCAAGATATTTAGTATTAAGAATTGCTAAGGAAAACTCTACCTTAAAACTGTAATCC  9585
               ************************************************************

Dromedary      AAATAGTCCAAAACAGAGATCCTATGAAGTGTGCAGAAATAGGCTGATGGCTCCTGAAAG  9422
Wild           AAATAGTCCAAAACAGAGATCCTATGAAGTGTGCAGAAATAGGCTGATGGCTCCTGAAAG  9223
Domestic       AAATAGTCCAAAACAGAGATCCTATGAAGTGTGCAGAAATAGGCTGATGGCTCCTGAAAG  9645
               ************************************************************

Dromedary      GCGGCAGCTGTGGCTGGCTCCCAGCCACCCTTTGCCTAGAGACAGTTGCTAGGCAGTACT  9482
Wild           GCGGTAGCTGTGGCTGGCTCCCAGCCACCCTTTGCCTAGAGACAGTTGCTAGGCAGTACT  9283
Domestic       GCGGTAGCTGTGGCTGGCTCCCAGCCACCCTTTGCCTAGAGACAGTTGCTAGGCAGTACT  9705
               **** *******************************************************

Dromedary      AGATTTGCTCACAAATGCACTTTTTACCCAATGATCAGTATTATGGTAACACACATGCCA  9542
Wild           AGATTTGCTCACAAATGCACTTTTTACCCAATGATCAGTATTATGGTAACACACATGCCA  9343
Domestic       AGATTTGCTCACAAATGCACTTTTTACCCAATGATCAGTATTATGGTAACACACATGCCA  9765
               ************************************************************

Dromedary      CCTCCCAATCAATTTTTCTGCTCCTATTTCATTAAAGGCAAGAGAGTCTGGAAAACATTA  9602
Wild           CCTCCCAATCAATTTTTCTGCTCCTATTTCATTAAAGGCAAGAGAGTCTGGAAAACATTA  9403
Domestic       CCTCCCAATCAATTTTTCTGCTCCTATTTCATTAAAGGCAAGAGAGTCTGGAAAACATTA  9825
               ************************************************************

Dromedary      CTGATGGCGGCTAATAGTCTTACGGCCACTGAATGGTGCTGGGCTAGTTCCTGGACAAAA  9662
Wild           CTGATGGCGGCTAATAGTCTTACGGCCACTGAATGGTGCTGGGCTAGTTCCTGGACAAAA  9463
Domestic       CTGATGGCGGCTAATAGTCTTACGGCCACTGAATGGTGCTGGGCTAGTTCCTGGACAAAA  9885
               ************************************************************

Dromedary      AGATTCAGATCTTTGTCTTCTAGGTCTAGGAATAGTTAGAGAAGCCCCAGAGCTACATAT  9722
Wild           AGATTCAGATCTTTGTCTTCTAGGTCTAGGAATAGTTACAGAAGCCCCAGAGCTACATAT  9523
Domestic       AGATTCAGATCTTTGTCTTCTAGGTCTAGGAATAGTTAGAGAAGCCCCAGAGCTACATAT  9945
               ************************************** *********************

Dromedary      TTCGTTTGTTCTTAATTCCTTTGAGCCTGAGTGTAAATGTTGAGCAGAATGACGGAATAA  9782
Wild           TTCGCTTGTTCTTAATTCCTTTGAGCCTGAGTGTAAATGTTGAGCAGAATGATGGAATAA  9583
Domestic       TTCGCTTGTTCTTAATTCCTTTGAGCCTGAGTGTAAATGTTGAGCAGAATGATGGAATAA  10005
               **** ************************************************ *******

Dromedary      TGGATCTGAGACTCATTTTCTTTCACTTTCTTTTGTTGACTCATAAAAAAACTACTACTG  9842
Wild           TGGATCTGAGACTCATTTTCTTTCACTTTCTTTTGTTGACTCATAAAAAAACTACTACTG  9643
Domestic       TGGATCTGAGACTCATTTTCTTTCACTTTCTTTTGTTGACTCATAAAAAAACTACTACTG  10065
               ************************************************************

Dromedary      CAACTCATTTAACTTAAACTCACATTGAAAATGCTTAGTAGACAGATCCTGAGAATTTGA  9902
Wild           CAACTCATTTAACTTAAACTCACATTGAAAATGCTTAGTAGACAGATCCTGAGAATTTGA  9703
Domestic       CAACTCATTTAACTTAAACTCACATTGAAAATGCTTAGTAGACAGATCCTGAGAATTTGA  10125
               ************************************************************

Dromedary      AATATGTAAAGAAAACAAACATGTCTAAATTCCCATTTCCTCCACAAAGCTTTCCTCAGT  9962
Wild           AATATGTAAAGAAAACAAAGATGTCTAAATTCCCATTTCCTCCACAAAGCTTTCCTCAGT  9763
Domestic       AATATGTAAAGAAAACAAAGATGTCTAAATTCCCATTTCCTCCACAAAGCTTTCCTCAGT  10185
               ******************* ****************************************

Dromedary      TCTTCACACTCAAACTGACAGAACACACTCATCAGTAACTTCCCAGTCAGTCCACTGGCA  10022
Wild           TCTTCACACTCAAACTGACAGAACACACTCATCAGTAACTTCCCAGTCAGTCCACTGGCA  9823
Domestic       TCTTCACACTCAAACTGACAGAACACACTCATCAGTAACTTCCCAGTCAGTCCACTGGCA  10245
               ************************************************************

Dromedary      TCTCCATCTAGAACTAGAATGCTAGTCGGTCAAGAATCTTAACTCATAATGAGAGTTGAA  10082
Wild           TCTCCATCTAGAACTAGAATGCTAGTCGGTCAAGAATCTTAACTCATAATGAGAGTTGAA  9883
Domestic       TCTCCATCTAGAACTAGAATGCTAGTCGGTCAAGAATCTTAACTCATAATGAGAGTTGAA  10305
               ************************************************************
```

```
Dromedary      ACTTATATAGTGCTTATTCTGTATCAAGCACTAATTTGAATACTTCACATGTATTATTCA 10142
Wild           ACTTATATAGTGCTTATTCTGTATCAAGCACTAATTTGAATACTTCACATGTATTATTCA 9943
Domestic       ACTTATATAGTGCTTATTCTGTATCAAGCACTAATTTGAATACTTCACATGTATTATTCA 10365
               ************************************************************

Dromedary      CATCAACTTCATGAAGTAGAAACTATTATCATTCCTATTTTTCAAGTGAGAAAATTGGGG 10202
Wild           CATCAACTTCATGAAGTAGAAACTATTATCATTCCTATTTTTCAAGTGAGAAAATTGGGG 10003
Domestic       CATCAACTTCATGAAGTAGAAACTATTATCATTCCTATTTTTCAAGTGAGAAAATTGGGG 10425
               ************************************************************

Dromedary      CACAGAGGGGCTATATAATCTGCCCAAGGTCACAAGGTAAGTGGCAGAGTTGGGAATCAA 10262
Wild           CACAGAGGGGCTATATAATCTGCCCAAGGTCACAAGGTAAGTGGCAGAGTTGGGAATCAA 10063
Domestic       CACAGAGGGGCTATATAATCTGCCCAAGGTCACAAGGTAAGTGGCAGAGTTGGGAATCAA 10485
               ************************************************************

Dromedary      ACCCAGACACACTGGTTCCAGAATCCATGCTCTCAACCACTAATTCTTACCATAGGTTCC 10322
Wild           ACCCAGACACACTGGTTCCAGAATCCATGCTCTCAACCACTAATTGTTATCATAGGTTCC 10123
Domestic       ACCCAGACACACTGGTTCCAGAATCCATGCTCTCAACCACTAATTGTTATCATAGGTTCC 10545
               ********************************************* *** **********

Dromedary      TTCCAGTTAAAAAATTCCTACACATTATCTAGGTCAACCTCATTTGACCAAAGAGAATCA 10382
Wild           TTCCAGTTAAAAAATTCCTACACATTATCTAGGTCAACCTCATTTGACCAAAGAGAATCA 10183
Domestic       TTCCAGTTAAAAAATTCCTACACATTATCTAGGTCAACCTCATTTGACCAAAGAGAATCA 10605
               ************************************************************

Dromedary      AAGGAGGAGAGATA-TCAGTTACATAAAGTTATATAAAGTAACGTTTTTCAAATTTTAAT 10441
Wild           AAGGAGGAGAGATATTCAGTTACATAAAGTTATATAAAGTAACGTTTTTCAAATTTTAAT 10243
Domestic       AAGGAGGAGAGATATTCAGTTACATAAAGTTATATAAAGTAACGTTTTTCAAATTTTAAT 10665
               ************** *********************************************

Dromedary      ATGGTGTGAATCACCTAGTGGTCTTTTAAAATGCAGATTTTAATTTAGTAGATAAAGGAT 10501
Wild           ATGGTGTGAATCACCTAGTGGTCTTTTAAAATGCAGATTTTAATTTAGTAGATAAAGGTT 10303
Domestic       ATGGTGTGAATCACCTAGTGGTCTTTTAAAATGCAGATTTTAATTTAGTAGATAAAGGTT 10725
               ********************************************************** *

Dromedary      GAAGACTGAGATTCTACATTTTTAACATGTCCCCACATGATACCGATTTTACAAGTCCAT 10561
Wild           GAAGACTGAGATTCTACATTTTTAACATGTCCCCACATGATACCGATTTTACAAGTCCAT 10363
Domestic       GAAGACTGAGATTCTACATTTTTAACATGTCCCCACATGATACCGATTTTACAAGTCCAT 10785
               ************************************************************

Dromedary      GTACCACACTTGGAGTAACAAGAACAAAGAGAAATGCTGAGACTGGAACTCAGTTCTGAC 10621
Wild           GTACCACACTTGGAGTAACAAGAACAAAGAGAAATGCTGAGACTGGAACTCAGTTCTGAC 10423
Domestic       GTACCACACTTGGAGTAACAAGAACAAAGAGAAATGCTGAGACTGGAACTCAGTTCTGAC 10845
               ************************************************************

Dromedary      TTATTCCTTCCACTGTCATCCCAAACATTATGCTAATTGGGTAAGGAGGGAGAAAAAAGT 10681
Wild           TTATTCCTTCCACTGTCATCCCAAACATTATGCTAATTGGGTAAGGAGGGAGAAAAAAGT 10483
Domestic       TTATTCCTTCCACTGTCATCCCAAACATTATGCTAATTGGGCAAGGAGGGAGAAAAAAGT 10905
               ***************************************** ******************

Dromedary      GACTGAGAGAATTTACTGTGGAAAAAAATTGACTTCATTTTGAATCATTTTCTTGTTGATT 10741
Wild           GACTGAGAGAATTTACTGTGGAAAAAAATTGACTTCATTTTGAATCATTTTCTTGTTGATT 10543
Domestic       GACTGAGAGAATTTACTGTGGAAAAAAATTGACTTCATTTTGAATCATTTTCTTGTTGATT 10965
               ************************************************************

Dromedary      ATAAGTAGCGAATAAATTTTATGGCGCTTTTTTCCCACATGTATTTTAATTTTGTAGTAG 10801
Wild           ATAAGTAGCGAATAAATTTTATGGTGCTTTTTTCCCACATGTATTTTAATTTTGTAGTAG 10603
Domestic       ATAAGTAGCGAATAAATTTTATGGTGCTTTTTTCCCACATGTATTTTAATTTTGTAGTAG 11025
               ************************ ***********************************

Dromedary      TATAAATGCTACATTTAGGTAGTGCTAAGATTTACCAGCTCTCTCAGATCTGCACAAGCA 10861
Wild           TATAAATGCTACATTTAGGTAGTGCTAAGATTTACCAGCTCTCTCAGATCTGCACAAGCA 10663
Domestic       TATAAATGCTACATTTAGGTAGTGCTAAGATTTACCAGCTCTCTCAGATCTGCACAAGCA 11085
               ************************************************************

Dromedary      TTGAGCTTGTCTAAGATTTATCATCTATAAATTCATTATTCACTTCTCTATACTCTTTAA 10921
Wild           TTGAGCTTGTCTAAGATTTATCATCTATAAATTCATTATTCACTTCTCTATACTCTTTAA 10723
Domestic       TTGAGCTTGTCTAAGATTTATCATCTATAAATTCATTATTCACTTCTCTATACTCTTTAA 11145
               ************************************************************
```

```
Dromedary      TTTATTGCTCTGGTAGTGACTTAGGGATGTGGGATTGAAGGAAGAAAGAGTATGTGGTAG 10981
Wild           TTTATTGCTCTGGTAGTGACTTAGGGATGTGGGATTGAAGGAAGAAAGAGTATGTGGTAG 10783
Domestic       TTTATTGCTCTGGTAGTGACTTAGGGATGTGGGATTGAAGGAAGAAAGAGTATGTGGTAG 11205
               ************************************************************

Dromedary      GAACACACGCTTTGTAAGACCCCAGAAAGCTGGAGTCAGGACACCACTCCTGAATATTTG 11041
Wild           GAACACATGCTTTGTAAGACCCCAGAAAGCTGGAGTCAGGACACCACTCCTGAATATTTG 10843
Domestic       GAACACATGCTTTGTAAGACCCCAGAAAGCTGGAGTCAGGACACCACTCCTGAATATTTG 11265
               ******* ****************************************************

Dromedary      GGCAGTCATTCTGTTTTTCCCACTGGGGCATATATTGCCTATGCTCTTCGTAAATAGTAA 11101
Wild           GGCAGTCATTCTGTTTTTCCCACTGGGGCATATATTGCCTATGCTCTTCGTAAATAGTAA 10903
Domestic       GGCAGTCATTCTGTTTTTCCCACTGGGGCATATATTGCCTATGCTCTTCGTAAATAGTAA 11325
               ************************************************************

Dromedary      TGTCCCTGGTATAAAAAGTACAGGACAAAATTAAGCTTCCCAGATCTGCAAAGACTTCCC 11161
Wild           TGTCCCTGGTATAAAAAGTACAGGACAAAATTAAGCTTCCCAGATCTGCAAAGACTTCCC 10963
Domestic       TGTCCCTGGTATAAAAAGTACAGGACAAAATTAAGCTTCCCAGATCTGCAAAGACTTCCC 11385
               ************************************************************

Dromedary      TTGCACAGCTAGTGTTAACAGTTGTAAATCCCTCTTTGAGGGGCTCTGGTAGCCACAGGA 11221
Wild           TTGCACAGCTAGTGTTAACAGTTGTAAATCCCTCTTTGAGGGGCTCTGGTAGCCACAGGA 11023
Domestic       TTGCACAGCTAGTGTTAACAGTTGTAAATCCCTCTTTGAGGGGCTCTGGTAGCCACAGGA 11445
               ************************************************************

Dromedary      AGCTGCACTGAGATATACCGGGATGTGTGGCCGGCTGGCTTCCACTAGGTCTGTGTGACC 11281
Wild           AGCTGCACTGAGATATACCGGGATGTGTGGCCGGCTGGCTTCCACTAGGTCTGTGTGACC 11083
Domestic       AGCTGCACTGAGATATACCGGGATGTGTGGCCGGCTGGCTTCCACTAGGTCTGTGTGACC 11505
               ************************************************************

Dromedary      TTGGTGAAGTACACAAATACGTCAGAAACTTGAGGGGAGTGAGTGTGTATGTTCTCTCTC 11341
Wild           TTGGTGAAGTACACAAATACGTCAGAAACTTGAGGGGAGTGAGTGTGTATGTTCTCTCTC 11143
Domestic       TTGGTGAAGTACACAAATACGTCAGAAACTTGAGGGGAGTGAGTGTGTATGTTCTCTCTC 11565
               ************************************************************

Dromedary      TAGTCATCTATCGTTTATTAAACCTATAAAGT--AAAAAAAAAAAAAGGAGCACACCAAT 11399
Wild           TAGTCATCTATCGTTTATTAAACCTATAAAGTAAAAAAAAAAAAAAAGGAGCACACCAAT 11203
Domestic       TAGTCATCTATCGTTTATTAAACCTATAAAGT-AAAAAAAAAAAAAAGGAGCACACCAAT 11624
               ********************************  **************************

Dromedary      TCTTCTGATGTCATTTATGTATGCTATGCCCATCATTTAAATAAAAACCACCTATTCCCC 11459
Wild           TCTTCTGATGTCATTTATGTATACTATGCCCATCATTTAAATAAAAACCACCTATT-CCC 11262
Domestic       TCTTCTGATGTCATTTATGTATACTATGCCCATCATTTAAATAAAAACCACCTATTCCCC 11684
               ********************** ********************************* ***

Dromedary      CCCCTCCTTTTTCCTGCCACTCTCACCCTACCCAAATCTGTGATTCTGCAGAACAAAATC 11519
Wild           CCCCCCCTTTTTCCTGCCACTCTCACCCTACCCAAATCTGTGATTCTGCAGAACAAAATC 11322
Domestic       CCCCCCCTTTTTCCTGCCACTCTCACCCTACCCAAATCTGTGATTCTGCAGAACAAAATC 11744
               **** *******************************************************

Dromedary      TGTTTGCTTTGTCTTCCCTTGCCTAAAGCCCTAATTCTGTAATTTACTCTAATGCACCCT 11579
Wild           TGTTTGCTTTGTCTTCCCTTGCCTAAAGCCCTAATTCTGTAATTTACTCTAATGCACCCT 11382
Domestic       TGTTTGCTTTGTCTTCCCTTGCCTAAAGCCCTAATTCTGTAATTTACTCTAATGCACCCT 11804
               ************************************************************

Dromedary      TAAACAATTCTGTATCTAGGAGCTTCTACTGTAGATTTTAAGTAAACTTAACCACCAACT 11639
Wild           TAAACAATTCTGTATCTAGGAGCTTCTACTGCAGATTTTAAGTAAACTTAACCACCAACT 11442
Domestic       TAAACAATTCTGTATCTAGGAGCTTCTACTGCAGATTTTAAGTAAACTTAACCACCAACT 11864
               ******************************* ****************************

Dromedary      GAAGATGTTTTGCTGTTGTTAGAGAAGGACATAAAATAGAACATTATGCCTTACATTTAT 11699
Wild           GAAGATGTTTTGCTGTTGTTAGAGAAGGACATAAAATAGAACATTATGCCTTACATTTAT 11502
Domestic       GAAGATGTTTTGCTGTTGTTAGAGAAGGACATAAAATAGAACATTATGCCTTACATTTAT 11924
               ************************************************************

Dromedary      TTTGGATAAATTTAGAATACCAGATAAAATAGCTCTTTAAAAAGACCTGAAAAATAAACC 11759
Wild           TTTGGATAAATTTAGAATACCAGATAAAATAGCTCTTTAAAAAGACCTGAAAAATAAACC 11562
Domestic       TTTGGATAAATTTAGAATACCAGATAAAATAGCTCTTTAAAAAGACCTGAAAAATAAACC 11984
               ************************************************************
```

```
Dromedary      TTGTGGGTTTCTTTCATAGATGAGAACTAACGCATTATTCAGGGATTAATTTTTTTTTAA  11819
Wild           TTGTGGATTTCTTTCATAGATGAGAACTAACGCATTATTCAGGGATTAA-TTTTTTTTAA  11621
Domestic       TTGTGGATTTCTTTCATAGATGAGAACTAACGCATTATTCAGGGATTAA-TTTTTTTTAA  12043
               ****** ****************************************** **********

Dromedary      AGACACTGTAAAGATGCTTTCAGATCACTATCATATTTTTATACATATTTATAAACATGA  11879
Wild           AGACACTGTAAAGATGCTTTCAGATCACTATCATATTTTTATACATATTTATAAACATGA  11681
Domestic       AGACACTGTAAAGATGCTTTCAGATCACTATCATATTTTTATACATATTTATAAACATGA  12103
               ************************************************************

Dromedary      TTTTCTGTTAATAAATTTTAATTATATAATGATACTTCCCAGTATACACTGTACCCTCAT  11939
Wild           TTTTCTGTTAATAAATTTTAATTATATAATGATACTTCCCAGTATACACTGTACCCTCAT  11741
Domestic       TTTTCTGTTAATAAATTTTAATTATATAATGATACTTCCCAGTATACACTGTACCCTCAT  12163
               ************************************************************

Dromedary      TTTTAAAATGCATTACTCCTGGAGTAATTAATCAATCTGAATTATTAAAGACATTATTGT  11999
Wild           TTTTAAAATGCATTACTCCTGGAGTAATTAATCAATCTGAATTATTAAAGACATTATTGT  11801
Domestic       TTTTAAAATGCATTACTCCTGGAGTAATTAATCAATCTGAATTATTAAAGACATTATTGT  12223
               ************************************************************

Dromedary      TTTTAAAAAGATTTTGAGGGTCATCAAAAATATCTCATGTCATATATTTTGCATAGCTTC  12059
Wild           TTTTAAAAAGATTTTGAGGGTCATCAAAAATATCTCATGTCATATATTTTGCATAGCTTC  11861
Domestic       TTTTAAAAAGATTTTGAGGGTCATCAAAAATATCTCATGTCATATATTTTGCATAGCTTC  12283
               ************************************************************

Dromedary      CTATGAGTCATCTGTTGAAAATCAATAGCTTTGAGACCTGCCATGCTGTTTTATTCATTT  12119
Wild           CTATGAGTCATCTGTTGAAAATCAATAGCTTTGAGACCTGCCATGCTGTTTTATTCATTT  11921
Domestic       CTATGAGTCATCTGTTGAAAATCAATAGCTTTGAGACCTGCCATGCTGTTTTATTCATTT  12343
               ************************************************************

Dromedary      GCCTCTTCCTTCCAAAATATAATTAAGTTTACGGGGGAGGGAGGATTTATTTTGTTTCCC  12179
Wild           GCCTCTTCCTTCCAAAATATAATTAAGTTTACGGGGGAGGGAGGATTTATTTTGTTTCCC  11981
Domestic       GCCTCTTCCTTCCAAAATATAATTAAGTTTACGGGGGAGGGAGGATTTATTTTGTTTCCC  12403
               ************************************************************

Dromedary      TCAGTATTACACTTATTAATTTATAGAAAGGCATCTTGCCTCATGAACTTTTTTTTAAAT  12239
Wild           TCAGTATTACACTTATTAATTTATAGAAAGGCATCTTGCCTCATGAACTTTTTTTTAAAT  12041
Domestic       TCAGTATTACACTTATTAATTTATAGAAAGGCATCTTGCCTCATGAACTTTTTTTTAAAT  12463
               ************************************************************

Dromedary      TGAGGCATAGTTGATTTATAATATTATATTAGTTTCAGGTGTACAACCTAGTGATTCAAA  12299
Wild           TGAGGCATAGTTGATTTACAATATTATATTAGTTTCAGGTGTACAACCTAGTGATTCAAA  12101
Domestic       TGAGGCATAGTTGATTTACAATATTATATTAGTTTCAGGTGTACAACCTAGTGATTCAAA  12523
               ****************** *****************************************

Dromedary      ATTTTTATAGATGATACTCCATTTATAATTACTATAAAATACTGGCTATATCCCCTGTGC  12359
Wild           ATTTTTATAGATGATACTCCATTTATAATTACTATAAAATACTGGCTATATCCCCTGTGC  12161
Domestic       ATTTTTATAGATGATACTCCATTTATAATTACTATAAAATACTGGCTATATCCCCTGTGC  12583
               ************************************************************

Dromedary      AGTACAATATGTCCTTGTAGCTCATTTACTTTATACATAGTAGTTTGTACCTCTTAATCC  12419
Wild           AGTACAATATGTCCTTGTAGCTCATTTACTTTATACATAGTAGTTTGTACCTCTTAATCC  12221
Domestic       AGTACAATATGTCCTTGTAGCTCATTTACTTTATACATAGTAGTTTGTACCTCTTAATCC  12643
               ************************************************************

Dromedary      CCTACTTCTATCTTGTCCCTCCCTCTTCCCTCTCCCTACTGGGAACCACTAGCTTGTTCC  12479
Wild           CCTACTTCTATCTTGTCCCTCCCTCTTCCCTCTCCCTACTGGGAACCACTAGCTTGTTCC  12281
Domestic       CCTACTTCTATCTTGTCCCTCCCTCTTCCCTCTCCCTACTGGGAACCACTAGCTTGTTCC  12703
               ************************************************************

Dromedary      CTATATCTGTGAATCTGCTTCCTTTTTGTTATATTATGTAGTTTGTTTTATTGTTTAGAT  12539
Wild           CTATATCTGTGAATCTGCTTCCTTTTTGTTATATTATGTAGTTTGTTTTATTGTTTAGAT  12341
Domestic       CTATATCTGTGAATCTGCTTCCTTTTTGTTATATTATGTAGTTTGTTTTATTGTTTAGAT  12763
               ************************************************************

Dromedary      CCCACATATAAATGATAACATACAGTATTTATCTTTCTCTGTCTGACTTATTTCCCTAAG  12599
Wild           TCCACATATAAATGATAACATACAGTATTTATCTTTCTCTGTCTGACTTATTTCCCTAAG  12401
Domestic       TCCACATATAAATGATAACATACAGTATTTATCTTTCTCTGTCTGACTTATTTCCCTAAG  12823
                ***********************************************************
```

```
Dromedary      CATAATACCCTCCAGGTCCATCCATGTTGCTGGAAATCCTATGAACTTTTATTTTAGTAG  12659
Wild           CATAATACCCTCCAGGTCCATCCATGTTGCTGGAAATCCTATGAACTTTTATTTTAGTAG  12461
Domestic       CATAATACCCTCCAGGTCCATCCATGTTGCTGGAAATCCTATGAACTTTTATTTTAGTAG  12883
               ************************************************************

Dromedary      AAATTGCCTTTAGAGTGCCATGTCAGCCACCATGATCTTTGTGACAGATTTCCCAACACA  12719
Wild           AAATTGCCTTTAGAGTGCCATGTCAGCCACCATGATCTTTGTGACAGATTTCCCAACACA  12521
Domestic       AAATTGCCTTTAGAGTGCCATGTCAGCCACCATGATCTTTGTGACAGATTTCCCAACACA  12943
               ************************************************************

Dromedary      TATCCCAAAATAGTGGTGGGAAAGAGAAAGCTCTTTCAGAAACTTCATTATTCCAGCATT  12779
Wild           TATCCCAAAATAGTGGTGGGAAAGAGAAAGCTCTTTCAGAAAATTCATTATTCCAGCATT  12581
Domestic       TATCCCAAAATAGTGGTGGGAAAGAGAAAGCTCTTTCAGAAAATTCATTATTCCAGCATT  13003
               ****************************************** *****************

Dromedary      GATTTCTGCACTGATTACAGAGTGGCTGATTTCTTGGTTCACTCCATTTAAGTCAAGTCC  12839
Wild           GATTTCTGCACTGATTACAGAGTGGCTGATTTCTTGGTTCACTCCATTTAAGTCAAGTCC  12641
Domestic       GATTTCTGCACTGATTACAGAGTGGCTGATTTCTTGGTTCACTCCATTTAAGTCAAGTCC  13063
               ************************************************************

Dromedary      AAATTCGTACTTTGTAGAAAGCTGTTTCTAAGAGCACAAGAGAATCTAAAACCATTTCAT  12899
Wild           AAATTCGTACTTTGTAGAAAGCTGTTTTTAAGAGCACAAGAGAATCTAAAACCATTTCAT  12701
Domestic       AAATTCGTACTTTGTAGAAAGCTGTTTTTAAGAGCACAAGAGAATCTAAAACCATTTCAT  13123
               *************************** ********************************

Dromedary      TAGGGTGATTTTGGAGACAAAAAGTCCTCTGAATTATGTAGAGAACATCATTTCACTGAC  12959
Wild           TAGGGTGATTTTGGAGACAAAAAGTCCTCTGAATTATGTAGAGAACATCATTTCACTGAC  12761
Domestic       TAGGGTGATTTTGGAGACAAAAAGTCCTCTGAATTATGTAGAGAACATCATTTCACTGAC  13183
               ************************************************************

Dromedary      CAAGATGGGCTTCACGGAGTCAACTTACTGCAGTAAGTCTCATAATCAGGAGTTATCTGC  13019
Wild           CAAGATGGGCTTCACGGAGTCAACTTACTGCAGTAAGTCTCATAATCAGGAGTTATCTGC  12821
Domestic       CAAGATGGGCTTCACGGAGTCAACTTACTGCAGTAAGTCTCATAATCAGGAGTTATCTGC  13243
               ************************************************************

Dromedary      ATTTGGCAATGCTATAGACTGGCACACTCCAAACTTAGGAGCATAAGGATTTGAAAGAAT  13079
Wild           ATTTGGCAATGCTATAGACTGGCACACTCCAAACTTAGGAGCATAAGGATTTGAAAGAAT  12881
Domestic       ATTTGGCAATGCTATAGACTGGCACACTCCAAACTTAGGAGCATAAGGATTTGAAAGAAT  13303
               ************************************************************

Dromedary      TAAGATGTTTTCTGAAAATGCATTTGAATTAAATGCAGAAGACTTGGTGGAAAAAGGATT  13139
Wild           TAAGATGTTTTCTGAAAATGCATTTGAATTAAATGCAGAAGACTTGGTGGAAAAAGGATT  12941
Domestic       TAAGATGTTTTCTGAAAATGCATTTGAATTAAATGCAGAAGACTTGGTGGAAAAAGGATT  13363
               ************************************************************

Dromedary      TAAAAGCTAATGGTACTAACTCTTGATAACCCTCAGGAAAGCACCAGACTGATTTTACTG  13199
Wild           TAAAAGCTAATGGTACTAACTCTTGATAACCCTCAGGAAAGCACCAGACTGATTTTACTG  13001
Domestic       TAAAAGCTAATGGTACTAACTCTTGATAACCCTCAGGAAAGCACCAGACTGATTTTACTG  13423
               ************************************************************

Dromedary      CATATGGACTAAATGGACTTCATACTCAAATACCAAGTTTTACCAGAAAAGGAAGATTTG  13259
Wild           CATATGGACTAAATGGACTTCATACTCAAATACCAAGTTTTACCAGAAAAGGAACATTTG  13061
Domestic       CATATGGACTAAATGGACTTCATACTCAAATACCAAGTTTTACCAGAAAAGGAACATTTG  13483
               ****************************************************** *****

Dromedary      GGAGGAGCAAACAAAGGGAAAAAAGTCACAGGTAAAATTCTGGAGTAAAATAAAACAATA  13319
Wild           GGAGGAGCAAACAAAGGGAAAAAAGTCACAGGTAAAATTCTGGAGTAAAATAAAACAATA  13121
Domestic       GGAGGAGCAAACAAAGGGAAAAAAGTCACAGGTAAAATTCTGGAGTAAAATAAAACAATA  13543
               ************************************************************

Dromedary      TAAAAGAAAAGTTAATACAAGCTAACAAAATTAGGGTGCTAGGGAACAAAGATGATTTGA  13379
Wild           TAAAAGAAAAGTTAATACAAGCTAACAAAATTAGGGTGCTAGGGAACAAAGATGATTTGA  13181
Domestic       TAAAAGAAAAGTTAATACAAGCTAACAAAATTAGGGTGCTAGGGAACAAAGATGATTTGA  13603
               ************************************************************

Dromedary      CAATGAAAATCAAGAAACAATCAATAAACTAATTTTTAAATATGTGCTTTAAAAAGATTG  13439
Wild           CAATGAAAATCAAGAAACAATCAATAAACTAATTTTTAAATATGTGCTTTAAAAAGATTG  13241
Domestic       CAATGAAAATCAAGAAACAATCAATAAACTAATTTTTAAATATGTGCTTTAAAAAGATTG  13663
               ************************************************************
```

```
Dromedary      ATGAGAAATGAGGAAAACTGAGTAAGATTTAAGTAGACTTTAAAAATGAGATTAAGATAG  13499
Wild           ATGAGAAATGAGGAAAACTGAGTAAGATTTAAGTAGACTTTAAAAATGAGATTAAGATAG  13301
Domestic       ATGAGAAATGAGGAAAACTGAGTAAGATTTAAGTAGACTTTAAAAATGAGATTAAGATAG  13723
               ************************************************************

Dromedary      AAAATAATTAAAAGATTCAAAGTCTCATACAGAGATAGTTCCTGTATGTTCTACACTTTG  13559
Wild           AAAATAATTAAAAGATTCAAAGTCTCATACAGAGATAGTTCCTGTATGTTCTACACTTTG  13361
Domestic       AAAATAATTAAAAGATTCAAAGTCTCATACAGAGATAGTTCCTGTATGTTCTACACTTTG  13783
               ************************************************************

Dromedary      TGTAAGAAAAGTGATCTTAACAAGTGCTAAGCCTCAGCATTACTGTCAATAAACTTTTCC  13619
Wild           TGTAAGAAAAGTGATCTTAACAATTGCTAAGCCTCAGCATTACTGTCAATAAACTTTTCC  13421
Domestic       TGTAAGAAAAGTGATCTTAACAATTGCTAAGCCTCAGCATTACTGTCAATAAACTTTTCC  13843
               *********************** ************************************

Dromedary      ATATTAACACAAACATTAAATTAATATGAAATAGTAATTGATATAATTAGTAGTAAGCAT  13679
Wild           ATATTAACACAAACATTAAATTAATATGAAATAGTAATTGATATAATTAGTAGTAAGCAT  13481
Domestic       ATATTAACACAAACATTAAATTAATATGAAATAGTAATTGATATAATTAGTAGTAAGCAT  13903
               ************************************************************

Dromedary      TTCTAAGAAATGAAAGTGACGTCAAATTATAAAAAGCTGAACATTATTGTTGAGTCAAAG  13739
Wild           TTCTAAGAAATGAAAGTGACGTCAAATTATAAAAAGCTGAACATTATTGTTGAGTCAAAG  13541
Domestic       TTCTAAGAAATGAAAGTGACGTCAAATTATAAAAAGCTGAACATTATTGTTGAGTCAAAG  13963
               ************************************************************

Dromedary      AATAATGAGAATGATATTTAGAATCATGTAACATGAAAAATTTATTTGGATTAAAGAATA  13799
Wild           AATAATGAGAATGATATTTAGAATCATGTAACATGAAAAATTTATTTGGATTAAAGAATA  13601
Domestic       AATAATGAGAATGATATTTAGAATCATGTAACATGAAAAATTTATTTGGATTAAAGAATA  14023
               ************************************************************

Dromedary      GCATGATTTGAATTGCTTCACTCACAAAAATAAGAGCAGCTAAAAGTCTGTGCTAAAGTG  13859
Wild           GCATGATTTGAATTGCTTCACTCACAAAAATAAGAGCAGCTAAAAGTCTGTGCTAAAGTG  13661
Domestic       GCATGATTTGAATTGCTTCACTCACAAAAATAAGAGCAGCTAAAAGTCTGTGCTAAAGTG  14083
               ************************************************************

Dromedary      GGACTGATAAGCAAGCAAATAAGAGAAATTAGTCCAACTAGTGTGAGAAGAGAAGCTACC  13919
Wild           GGACTGATAAGCAAGCAAATAAGAGAAATTAGTCCAACTAGTGTGAGAAGAGAAGCTACC  13721
Domestic       GGACTGATAAGCAAGCAAATAAGAGAAATTAGTCCAACTAGTGTGAGAAGAGAAGCTACC  14143
               ************************************************************

Dromedary      ATGGTCTTGTCAAGGTCTTACTGTATCTTTAAAGAGACAAACTGATCAGAAGCTTCAGAC  13979
Wild           ATGGTCTTGTCAAGGTCTTACTGTATCTTTAAAGAGACAAACTGATCAGAAGCTTCAGAC  13781
Domestic       ATGGTCTTGTCAAGGTCTTACTGTATCTTTAAAGAGACAAACTGATCAGAAGCTTCAGAC  14203
               ************************************************************

Dromedary      AAGGTAGAAATCGCTACATCAAATGAACTGCTTCAGGGATCCGCTTAAGTCATCCTTTCT  14039
Wild           AAGGTAGAAATCGCTACATCAAATGAACTGCTTCAGGGATCCGCTTAAGTCATCCTTTCT  13841
Domestic       AAGGTAGAAATCGCTACATCAAATGAACTGCTTCAGGGATCCGCTTAAGTCATCCTTTCT  14263
               ************************************************************

Dromedary      CCATTTCTAGCCCACCTACAACCTGATTTGAAGGTGGGTCTACACAAGAAGGGCACCAGC  14099
Wild           CCATTTCTAGCCCACCTACAACCTGATTTGAAGGTGGGTCTACACAAGAAGGGCACCAGC  13901
Domestic       CCATTTCTAGCCCACCTACAACCTGATTTGAAGGTGGGTCTACACAAGAAGGGCACCAGC  14323
               ************************************************************

Dromedary      ACACTTGTCAGTGTGTTCATTCTCACTTTGAATTCAAACACAGCACTCATCAATGGCCTA  14159
Wild           ACATTTGTCAGTGTGTTCATTCTCACTTTAAATTCAAACACAGCACTCATCAATGGCCTA  13961
Domestic       ACATTTGTCAGTGTGTTCATTCTCACTTTGAATTCAAACACAGCACTCATCAATGGCCTA  14383
               *** ************************* ******************************

Dromedary      ATGTGAAGCAGCCACTTTTTCAATAACAAACTCACCACTAGGTTGCAGTCTTAGCTCAAC  14219
Wild           ATGTGAAGCAGCCACTTTTTCAATAACAAACTCACCACTAGGTTGCAGTCTTAGCTCAAC  14021
Domestic       ATGTGAAGCAGCCACTTTTTCAATAACAAACTCACCACTAGGTTGCAGTCTTAGCTCAAC  14443
               ************************************************************

Dromedary      CGCTCCTTTAAGAACAAACTACAACATATAAAATAAATAAGCTGAAAGGATATATTGTAC  14279
Wild           CGCTCCTTTAAGAACAAACTACAACATATAAAATAAATAAGCTGAAAGGATATATTGTAC  14081
Domestic       CGCTCCTTTAAGAACAAACTACAACATATAAAATAAATAAGCTGAAAGGATATATTGTAC  14503
               ************************************************************
```

```
Dromedary     AACACACAGAATATAGTCAAAACTTTGTAATGACTATAAATAGAATATAATACTTAAAAG 14339
Wild          AACACACAGAATATAGTCAAAACTTTGTAATGACTATAAATAGAATATAATACTTAAAAG 14141
Domestic      AACACACAGAATATAGTCAAAACTTTGTAATGACTATAAATAGAATATAATACTTAAAAG 14563
              ************************************************************

Dromedary     TTGTGAATTGTTATGTTGTACACCTGAAACATATAGTATTATACATCAACTCTACCTCAA 14399
Wild          TTGTGAATTGTTATGTTGTACACCTGAAACATATAGTATTATACATCAACTCTACCTCAA 14201
Domestic      TTGTGAATTGTTATGTTGTACACCTGAAACATATAGTATTATACATCAACTCTACCTCAA 14623
              ************************************************************

Dromedary     TTTAAAAAAAAAAAACCGTTCACAAGCTGTTAACCATTTTAATGGTGAAACACCATTACT 14459
Wild          TTT-AAAAAAAAAAACCGTTCACAAGCTGTTAACCATTTTAATGGTGAAACACCATTACT 14260
Domestic      TTT-AAAAAAAAAAACCGTTCACAAGCTGTTAACCATTTTAATGGTGAAACACCATTACT 14682
              *** ********************************************************

Dromedary     TATCTCTTGTGACAGAGCTGATACAAGCCACACTTAGTTTATCAGTCAGTTTACTAGCCA 14519
Wild          TATCTCTTGTGACAGAGCTGATACAAGCCACACTTAGTTTATCAGTCAGTTCACTAGCCA 14320
Domestic      TATCTCTTGTGACAGAGCTGATACAAGCCACACTTAGTTTATCAGTCAGTTCACTAGCCA 14742
              *************************************************** ********

Dromedary     ATCTAGATTCCGTGCAGATACATTGTTTTTCAAAAGCATCAAAACTCAAAAATCACCTGT 14579
Wild          ATCTAGATTCCGTGCAGATACATTGTTTTTCAAAAGCATCAAAACTCACAAATCACCTGT 14380
Domestic      ATCTAGATTCCATGCAGATACATTGTTTTTCAAAAGCATCAAAACTCACAAATCACCTGT 14802
              *********** ************************************* ***********

Dromedary     CAAAATGTTCTCACTCTTCAGCAGGCAGCCTCCTTTAGGGTTTTCTTGATCACGTAGCCA 14639
Wild          CAAAATGTTCTCACTCTTCAGCAGGCAGCCTCCTTTAGGGTTTTCTTGATCACGTAGCCA 14440
Domestic      CAAAATGTTCTCACTCTTCAGCAGGCAGCCTCCTTTAGGGTTTTCTTGATCACGTAGCCA 14862
              ************************************************************

Dromedary     AAAGAAAAAAAATAATTCCTTGGGTACTTTAAGCCTCTAGTTTCAGTATAGGGCTAATGG 14699
Wild          AAAGAAAAAAAATAATTCCTTGGGTACTTTAAGCCTCTAGTTTCAGTATAGGGCTAATGG 14500
Domestic      AAAGAAAAAAAATTATTCCTTGGGTACTTTAAGCCTCTAGTTTCAGTATAGGGCTAATGG 14922
              ************* **********************************************

Dromedary     TTAAATAACAAGTACATAATTACCTTTCTCTTTGTTTCTAGTGACGGTGCTAGGTCCTAA 14759
Wild          TTAAATAACAAGTACATAATTACCTTTCTCTTTGTTTCTAGTGACTGTGCTAGGTCCTAA 14560
Domestic      TTAAATAACAAGTACATAATTACCTTTCTCTTTGTTTCTAGTGACTGTGCTAGGTCCTAA 14982
              ********************************************* **************

Dromedary     GACCTCCTTGTAAGAGTCAGCCTTACTGTGCTGATGCCTATGAAAAATCCACCTGTCTGT 14819
Wild          GACCTCCTTGTAAGAGTCAGCCTTACTGTGCTGATGCCTATGAAAAATCCACCTGTCTGT 14620
Domestic      GACCTCCTTGTAAGAGTCAGCCTTACTGTGCTGATGCCTATGAAAAATCCACCTGTCTGT 15042
              ************************************************************

Dromedary     TATCATTGTAGGCATGCTCACTAGTCAGCCCCCAAATGAGCTTCAGGTGATACTCATTCT 14879
Wild          TATCATTGTAGGCATGCTCACTAGTCAGCCCCCAAATGAGCTTCAGGTGATATTCATTCT 14680
Domestic      TATCATTGTAGGCATGCTCACTAGTCAGCCCCCAAATGAGCTTCAGGTGATATTCATTCT 15102
              **************************************************** *******

Dromedary     TTAATCCCTAAAGGCATACTTTTAAAGGAACTTGACACTTTTTATTTTTAAAGGGAAAAC 14939
Wild          TTAATCCCTAAAGGCATACTTTTAAAGGAACTTGACACTTTTTATTTTTAAAGGGAAAAC 14740
Domestic      TTAATCCCTAAAGGCATACTTTTAAAGGAACTTGACACTTTTTATTTTTAAAGGGAAAAC 15162
              ************************************************************

Dromedary     CCCAGAGATAGACAGAAAT-AAAATAAGGAAATGAAACAATGTCTTACTTGTCATGCATT 14998
Wild          CCCAGAGATAGACAGAAATAAAAATAAGGAAATGAAACAATGTCTTACTTGTCATGCATT 14800
Domestic      CCCAGAGATAGACAGAAATAAAAATAAGGAAATGAAACAATGTCTTACTTGTCATGCATT 15222
              ******************* ****************************************

Dromedary     TGAATCCTCTTGAATACTTTTTTAATGGAAGTGCCCATAAAAGTAGTGTTTCCCTTTCAC 15058
Wild          TGAATCCCCTTGAATACTTTTTTAATGGAAGTGCCCATAAAAGTAGTGTTTCCCTTTCAC 14860
Domestic      TGAATCCTCTTGAATACTTTTTTAATGGAAGTGCCCATAAAAGTAGTGTTTCCCTTTCAC 15282
              ******* ****************************************************

Dromedary     GAACGACTGATAAGTTTTGTGGATGTTTTGGCAGTGGAAGGGTGGTGGGGGAGGGAGATA 15118
Wild          GAACGACTGATAAGTTTTGTGAATGTTTTGGCAGTGGAAGGGTGGTGGGGGAGGGAGATA 14920
Domestic      GAACGACTGATAAGTTTTGTGAATGTTTTGGCAGTGGAAGGGTGGTGGGGGAGGGAGATA 15342
              ********************* **************************************
```

```
Dromedary      GTTATCACAATTTTTTTCTGGAGGAACTAGCTAGATATTTAGTTTGAATATATATTTTAA  15178
Wild           GTTATCACAATTTTTTTCTGGAGGAACTAGCTAGATATTTAGTTTGAATATATATTTTAA  14980
Domestic       GTTATCACAATTTTTTTCTGGAGGAACTAGCTAGATATTTAGTTTGAATATATATTTTAA  15402
               ************************************************************

Dromedary      AATCATTTCCAATAGGTAACAAGGTATCCAATTATAAATTTAAAATACATTATATGTAAC  15238
Wild           AATCATTTCCAATAGGTAACAAGGTATCCAATTATAAATTTAAAATACATTATATGTAAC  15040
Domestic       AATCATTTCCAATAGGTAACAAGGTATCCAATTATAAATTTAAAATACATTATATGTAAC  15462
               ************************************************************

Dromedary      CACACCAAACTCTCTAAAATTCCAAAAAGATAGAGTAACTGGTAATAATCACATAAAACA  15298
Wild           CACACCAAACTCTCTAAAATTCCAAAAAGATAGAGTAACTGGTAATAATCACATAAAACA  15100
Domestic       CACACCAAACTCTCTAAAATTCCAAAAAGATAGAGTAACTGGTAATAATCACATAAAACA  15522
               ************************************************************

Dromedary      GACAACATTCATCTAAAGCCTAATACATGCCAGACCTCGTAGTTGATGCCTGAAGTGCAC  15358
Wild           GACAACATTCATCTAAAGCCTAATACATGCCAGACCTCGTAGTTGATGCCTGAAGTGCAC  15160
Domestic       GACAACATTCATCTAAAGCCTAATACATGCCAGACCTCGTAGTTGATGCCTGAAGTGCAC  15582
               ************************************************************

Dromedary      TGTTTCATTTAATCTGCCCAACAAGCCTGTAAGGTAGATCCTTTTATTATCAGACAGTCT  15418
Wild           TGTTTCATTTAATCTGCCCAACAAGCCTGTAAGGTAGATTCTTTTATTATCAGACAGTCT  15220
Domestic       TGTTTCATTTAATCTGCCCAACAAGCCTGTAAGGTAGATTCTTTTATTATCAGACAGTCT  15642
               *************************************** ********************

Dromedary      TTTCCTTTGTAGTGCGGAACTGTAAAAATGACTGTGCCACTGAAACTGCGCAAAGAGAGC  15478
Wild           TTTCCTTTGTAGTGCGGAACTGTAAAAATGACTGTGCCACTGAAACTGCGCAAAGAGAGC  15280
Domestic       TTTCCTTTGTAGTGCGGAACTGTAAAAATGACTGTGCCACTGAAACTGCGCAAAGAGAGC  15702
               ************************************************************

Dromedary      TCAGTCATCAATGGGAAAAATTACAACTGATCCATGACTTCAAAAAATTTTAGAAAATTC  15538
Wild           TCAGTCATCAATGGGAAAAATTACAACTGATCCATGACTTCAAAAAATTTTAGAAAATTC  15340
Domestic       TCAGTCATCAATGGGAAAAATTACAACTGATCCATGACTTCAAAAAATTTTAGAAAATTC  15762
               ************************************************************

Dromedary      TCCTACTGCTGATTAAATGTAGAGGGAAACTTACAAATCTATGAAACTGATGTTAGCATA  15598
Wild           TCCTACTGCTGATTAAATGTAGAGGGAAACTTACAAATCTATGAAACTGATGTTAGCATA  15400
Domestic       TCCTACTGCTGATTAAATGTAGAGGGAAACTTACAAATCTATGAAACTGATGTTAGCATA  15822
               ************************************************************

Dromedary      GAATAATTTCAAACATTAGAAACATACCAAACCAGAGTGTTTTATTTCTCTGTAAAAAAC  15658
Wild           GAATAATTTCAAACATTAGAAACATACCAAACCAGAGTGTTTTATTTCTCTGTAAAAAAC  15460
Domestic       GAATAATTTCAAACATTAGAAACATACCAAACCAGAGTGTTTTATTTCTCTGTAAAAAAC  15882
               ************************************************************

Dromedary      TTATCAAGAGTAGTTTGAACAGTTCCAGCCCCCTTCTCCTTGTTTTATAACTTACATTAC  15718
Wild           TTATCAAGAGTAGTTTGAACAGTTCCAGCCCCCTTCTCCTTGTTTTATAACTTACATTAC  15520
Domestic       TTATCAAGAGTAGTTTGAACAGTTCCAGCCCCCTTCTCCTTGTTTTATAACTTACATTAC  15942
               ************************************************************

Dromedary      AGAGCAAGAGTCTTTCCTCTGCCTTGGCAAATGGTCATTCTCCTTTCTTAGTTTGGATCA  15778
Wild           AGAGCAAGAGTCTTTCCTCTGCCTTGGCAAATGGTCATTCTCCTTTCTTAGTTTGGATCA  15580
Domestic       AGAGCAAGAGTCTTTCCTCTGCCTTGGCAAATGGTCATTCTCCTTTCTTAGTTTGGATCA  16002
               ************************************************************

Dromedary      GCTTACAATATTTTACCGTTTGTGCTTTCCACGTCAGGAAATATCTCCAAGATTACCTTT  15838
Wild           GCTTACAATATTTTACCGTTTGTGCTTTCCACGTCAGGAAATATCTCCAAGATTACCTTT  15640
Domestic       GCTTACAATATTTTACCGTTTGTGCTTTCCACGTCAGGAAATATCTCCAAGATTACCTTT  16062
               ************************************************************

Dromedary      ACTGTGAAGATTTCTGCCAGTATCACTTTCTCTGGGACATCTTCCTTTTCTTGTCACAAC  15898
Wild           ACTGTGAAGATTTCTGCCAGTATCACTTTCTCTGGGACACCTTCCTTTTCTTGTCACAAC  15700
Domestic       ACTGTGAAGATTTCTGCCAGTATCACTTTCTCTGGGACACCTTCCTTTTCTTGTCACAAC  16122
               *************************************** ********************

Dromedary      CATTTTCCTCCTTTGTGTTGATAAGGTAGCCTTCACTTAGTTGCTCGACTTCAGGTCTAG  15958
Wild           CATTTTCCTCCTTTGTGTTGATAAGGTAGCCTTCACTTAGTTGCTCGACTTCAGGTCTAG  15760
Domestic       CATTTTCCTCCTTTGTGTTGATAAGGTAGCCTTCACTTAGTTGCTCGACTTCAGGTCTAG  16182
               ************************************************************
```

```
Dromedary      GGTCCCTCCAACGGCAGCAGTGTCAACTGCCCCACAGTCAGCTATGTCTTCTATGACTTT 16018
Wild           GGTCCCTCCAACGGCAGCAGTGTCAACTGCCCCACAGTCAGCTATGTCTTCTATGACTTT 15820
Domestic       GGTCCCTCCAACGGCAGCAGTGTCAACTGCCCCACAGTCAGCTATGTCTTCTATGACTTT 16242
               ************************************************************

Dromedary      ATTTATGTTTGATCTGAATTTCACCACCATTAACCTGAGGGCCAGCATTACCACTTTTCA 16078
Wild           ATTTATGTTTGATCTGAATTTCACCACCATTAACCTGAGGGCCAGCATTACCACTTTTCA 15880
Domestic       ATTTATGTTTGATCTGAATTTCACCACCATTAACCTGAGGGCCAGCATTACCACTTTTCA 16302
               ************************************************************

Dromedary      TTTCGTTGCTGCATTTCATCTCTTTAGGCCAATTTCTCCTTTGGCTAGCCACTTTTGTAA 16138
Wild           TTTCGTTGCTGCATTTCATCTCTTTAGGCCAATTTCTCCTTTGGCTAGCCACTTTTGTAA 15940
Domestic       TTTCGTTGCTGCATTTCATCTCTTTAGGCCAATTTCTCCTTTGGCTAGCCACTTTTGTAA 16362
               ************************************************************

Dromedary      AATGTCCTATGGTTTTATCACCAGGAGCCACAGAGGCAACACAACTACAGACTTCACTGT 16198
Wild           AATGTCCTATGGTTTTATCACCAGGAGCCACAGAGGCAACACAACTACAGACTTCACTGT 16000
Domestic       AATGTCCTATGGTTTTATCACCAGGAGCCACAGAGGCAACACAACTACAGACTTCACTGT 16422
               ************************************************************

Dromedary      CTGGGCATGAACTGAATAATAGATGCACAGTGACCAACCACTGATAGACTTTGAAAAAAA 16258
Wild           CTGGGCATGAACTGAATAATAGATGCACAGTGACCAACCACTGATAGACTTTGAAAAAAA 16060
Domestic       CTGGGCATGAACTGAATAATAGATGCACAGTGACCAACCACTGATAGACTTTGAAAAAAA 16482
               ************************************************************

Dromedary      CACTGGAGAATTTTGAGCAGAAGAGAATGGTGTAGGAGAAATGAAAAAAACATCTGTCCT 16318
Wild           CACTGGAGAATTTTGAGCAGAAGAGAATGGTGTAGGAGAAATGAAAAAAACATCTGTCCT 16120
Domestic       CACTGGAGAATTTTGAGCAGAAGAGAATGGTGTAGGAGAAATGAAAAAAACATCTGTCCT 16542
               ************************************************************

Dromedary      TTTCCTCAGGCCTCGTGATTAATGGGAAGGAGACGCAGGGAAGAGAGGTAGATGGTGAGA 16378
Wild           TTTCCTCAGGCCTGGTGATTAATGGGAAGGAGACGCAGGGAAGAGAGGTAGATGGTGAGA 16180
Domestic       TTTCCTCAGGCCTGGTGATTAATGGGAAGGAGACGCAGGGAAGAGAGGTAGATGGTGAGA 16602
               ************* **********************************************

Dromedary      AAAGCAGACGTGGGGCTCC-TTCTGCTCTCCTGGTTTTGGCCGGGAGATGGGAGGGTACT 16437
Wild           AAAGCAGACGTGGGGCTCCTTTCTGCTCTCCTGGTTTTGGCCGGGAGATGGGAGGGTACT 16240
Domestic       AAAGCAGACGTGGGGCTCCTTTCTGCTCTCCTGGTTTTGGCCGGGAGATGGGAGGGTACT 16662
               ******************* ****************************************

Dromedary      CAGGAAGTGAAGCTCTTGCTCCATTCTCTGGAAGAAAGTTCTCTCACGTTGCCCCTCCCA 16497
Wild           CAGGAAGTGAAGCTCTTGCTCCATTCTCTGGAAGAAAGTTCTCTCACGTTGCCCCTCCCA 16300
Domestic       CAGGAAGTGAAGCTCTTGCTCCATTCTCTGGAAGAAAGTTCTCTCACGTTGCCCCTCCCA 16722
               ************************************************************

Dromedary      CCAACATGGAGCAGTTCCAACACTTTAACACAGCATGGGCAGCCAGAGGGAGGCCAAGAG 16557
Wild           CCAACATGGAGCAGTTCCAACACTTTAACACAGCATGGGCAGCCAGAGGGAGGCCAAGAG 16360
Domestic       CCAACATGGAGCAGTTCCAACACTTTAACACAGCATGGGCAGCCAGAGGGAGGCCAAGAG 16782
               ************************************************************

Dromedary      GTTAGCTTTCCTGGCTGAGAATCTTTCCAAACGGTGGCCCGGTTGGGAAGGTGACCCTCC 16617
Wild           GTTAGCTTTCCTGGCTGAGAATCTTTCCAAACGGTGGCCCGGTTGGGAAGGTGACCCTCC 16420
Domestic       GTTAGCTTTCCTGGCTGAGAATCTTTCCAAACGGTGGCCCGGTTGGGAAGGTGACCCTCC 16842
               ************************************************************

Dromedary      CCAGAAGCTGGCTATGGGGTACACTTCTCAGGCAGGACCCAATGGCAGTCACAGAGTTCA 16677
Wild           CCGGAAGCTGGCTATGGGGTACGCTTCTCAGGCAGGACCCAATGGCAGTCACAGAGTTCA 16480
Domestic       CCGGAAGCTGGCTATGGGGTACGCTTCTCAGGCAGGACCCAATGGCAGTCACAGAGTTCA 16902
               ** ******************* *************************************

Dromedary      GAGGGGAGCCTTGCTCACTCAGAGTGTGCTCCCTGGATGGGTGGCATCAGCATCACCCTC 16737
Wild           GAGGGGAGCCTTGCTCACTCAGAGTGTGCTCCCTGGATGGGTGGCATCAGCATCACCCTC 16540
Domestic       GAGGGGAGCCTTGCTCACTCAGAGTGTGCTCCCTGGATGGGTGGCATCAGCATCACCCTC 16962
               ************************************************************

Dromedary      ACCTGGGAGTTTGTTAGAAATTTAAATTCTCAGGCCCTACCCTAAATCTACTTAATCAGA 16797
Wild           ACCTGGGAGTTTGTTAGAAATTTAAATTCTCAGGCCCTACCCTAAATCTACTTAATCAGA 16600
Domestic       ACCTGGGAGTTTGTTAGAAATTTAAATTCTCAGGCCCTACCCTAAATCTACTTAATCAGA 17022
               ************************************************************
```

```
Dromedary     ATCTCTGGGAGTGAGGCCAGGGATTTGTGTTTTAACAAGCTCTGCAGATGATTTTTATTC  16857
Wild          ATCTCTGGGAGTGAGGCCAGGGATTTTTGTTTTAACAAGCTCTGCAGATGATTTTTATTC  16660
Domestic      ATCTCTGGGAGTGAGGCCAGGGATTTTTGTTTTAACAAGCTCTGCAGATGATTTTTATTC  17082
              ************************** *********************************

Dromedary     ATGTTTAAGTTTGAGAAGCACTGGTCCATAGCACCAACAGAAGCTAAAAGTGAGGCTGAG  16917
Wild          ATGTTTAAGTTTGAGAAGCACTGGTCCATAGCGCCAACAGAAGCTAAAAGTGAGGCTGAG  16720
Domestic      ATGTTTAAGTTTGAGAAGCACTGGTCCATAGCGCCAACAGAAGCTAAAAGTGAGGCTGAG  17142
              ******************************** ***************************

Dromedary     TCTGTCAGTGCATCCCACGGAGGCTTCACCCAAGCCAAAAGGACCAGTGGTCTCCTTCAG  16977
Wild          TCTGTCAGTGCATCCCACGGAGGCTTCACCCAAGCCAAAAGGACCAGTGGTCTCCTTCAG  16780
Domestic      TCTGTCAGTGCATCCCACGGAGGCTTCACCCAAGCCAAAAGGACCAGTGGTCTCCTTCAG  17202
              ************************************************************

Dromedary     CCATCAGGCGATAGGGGCTGTTGTGCAAAGGAGGTGGCAGAAGGGAGACCCCTGGGGCCC  17037
Wild          CCATCAGGCGATAGGGGCTGTTGTGCAAAGGAGGTGGCAGAAGGGAGACCCCTGGGGCCC  16840
Domestic      CCATCAGGCGATAGGGGCTGTTGTGCAAAGGAGGTGGCAGAAGGGAGACCCCTGGGGCCC  17262
              ************************************************************

Dromedary     CAGGAGCCTGTCTTGCCCCTGCCCCCAACCCCCATGCCTTCAGATCACATAAGCCACATA  17097
Wild          CAGGAGCCTGTCTTGCCCCTGCCCCCAACCCCCATGCCTTCAGATCACATAAGCCACATA  16900
Domestic      CAGGAGCCTGTCTTGCCCCTGCCCCCAACCCCCATGCCTTCAGATCACATAAGCCACATA  17322
              ************************************************************

Dromedary     CCTCTCTCCCCAGATATCTGGCCCTCCTGGAGGAGGAGAGAGGATTGATAAGAGACTCGG  17157
Wild          CCTCTCTCCCCAGATATCTGGCCCTCCTGGAGGAGGAGAGAGGATTGATAAGAGACTCGG  16960
Domestic      CCTCTCTCCCCAGATATCTGGCCCTCCTGGAGGAGGAGAGAGGATTGATAAGAGACTCGG  17382
              ************************************************************

Dromedary     TATTTTATGCAAGAAAACCCAAGCACTACCTAGAAGAACAATTTAAAATACCGGACAAAA  17217
Wild          TATTTTATGCAAGAAAACCCAAGCACTACCTAGAAGAACAATTTAAAATACCGGACAAAA  17020
Domestic      TATTTTATGCAAGAAAACCCAAGCACTACCTAGAAGAACAATTTAAAATACCGGACAAAA  17442
              ************************************************************

Dromedary     TTTTAAACTGGATTGGACATTGTTGATTTTTTTTTTTTTTTGACTGTTTTGTTTTTCTAA  17277
Wild          TTTTAAACTGGATTGGACATTGTTGG--TTTTTTTTTTTTTGACTGTTTTGTTTTTCTAA  17078
Domestic      TTTTAAACTGGATTGGACATTGTTGG--TTTTTTTTTTTTTGACTGTTTTGTTTTTCTAA  17500
              *************************   ********************************

Dromedary     GCAGAGGATGGCGAGCATGGGCAGAAGACCAGATCAGCTGCAAAGTAAAAAAGCTACAAG  17337
Wild          GCAGAGGATGGCGAGCATGGGCAGAAGACCAGATCAGCTGCAAAGTAAAAAAGCTACAAG  17138
Domestic      GCAGAGGATGGCGAGCATGGGCAGAAGACCAGATCAGCTGCAAAGTAAAAAAGCTACAAG  17560
              ************************************************************

Dromedary     TTACCTGCACACTTCAATTATTGTTTTACAATTACAATATGTTAACATGCATTCTAAGTT  17397
Wild          TTACCTGCACACTTCAATTACTGTTTTACAATTACAATATGTTAACATGCATTCTAAGTT  17198
Domestic      TTACCTGCACACTTCAATTACTGTTTTACAATTACAATATGTTAACATGCATTCTAAGTT  17620
              ******************** ***************************************

Dromedary     TCCAAGAGTGGAAGACTGTGTAGCATTTCCACACTTATTTCAACACAGAGCCCTTTTCCT  17457
Wild          TCCAAGAGTGAAAGACTGTGTAGCATTTCCACACTTATTTCAACACAGAGCCCTTTTCCT  17258
Domestic      TCCAAGAGTGAAAGACTGTGTAGCATTTCCACACTTATTTCAACACAGAGCCCTTTTCCT  17680
              ********** *************************************************

Dromedary     CCAAGAATAATAAAAGCTAACCCTGACTGTACACTTACTATCTGCCAGGGACTACATTAA  17517
Wild          CCAAGAATAATAAAAGCTAACCCTGACTGTACACTTACTATCTGCCAGGGACTACATTAA  17318
Domestic      CCAAGAATAATAAAAGCTAACCCTGACTGTACACTTACTATCTGCCAGGGACTACATTAA  17740
              ************************************************************

Dromedary     GCAAAAATACTTCAATAGAATAGACCAGAAAAACATAAAAGTAGATTCCATATCATTCCC  17577
Wild          GCAAAAATACTTCAATAGAATAGACCAGAAAAACATAAAAGTAGATTCCATATCATTCCC  17378
Domestic      GCAAAAATACTTCAATAGAATAGACCAGAAAAACATAAAAGTAGATTCCATATCATTCCC  17800
              ************************************************************

Dromedary     ACTTTAGCAGAGGAGATCTGCAGTGTTAACTAATGAATCTTAAGTCTCAGGGGTCCTCAC  17637
Wild          ACTTTAGCAGAGGAGATCTGCAGTGTTAACTAATGAATCTTAAGTCTCAGGGGTCCTCAC  17438
Domestic      ACTTTAGCAGAGGAGATCTGCAGTGTTAACTAATGAATCTTAAGTCTCAGGGGTCCTCAC  17860
              ************************************************************
```

```
Dromedary     TTGCATAGGCCCCTTCCAAGGCCCTATATCTAATTTTGTATTTATAATTTTGTATTTTTT 17697
Wild          TTGCATAGGCCCCTTCCAAGGCCCTATATCTAATTTTGTATTTATAATTTTGTATTTTTT 17498
Domestic      TTGCATAGGCCCCTTCCAAGGCCCTATATCTAATTTTGTATTTATAATTTTGTATTTTTT 17920
              ************************************************************

Dromedary     TCTTTAAAAGGGCTACTGTATTTCAGCTCTACTATCACTGGCCACCTGCCTACCACCTAC 17757
Wild          TCTTTAAAAGGGCTACTGTATTTCAGCTCTACTATCACTGGCCACCTGCCTACCACCTAC 17558
Domestic      TCTTTAAAAGGGCTACTGTATTTCAGCTCTACTATCACTGGCCACCTGCCTACCACCTAC 17980
              ************************************************************

Dromedary     CTTAAATGCAAGGTTTGTAATAGAGCTTACCTGGTAAAGAAAAATAACTGGGGAAAAGGT 17817
Wild          CTTAAATGCAAGGTTTGTAATAGAGCTTACCTGGTAAAGAAAAATAACTGGGGAAAAGGT 17618
Domestic      CTTAAATGCAAGGTTTGTAATAGAGCTTACCTGGTAAAGAAAAATAACTGGGGAAAAGGT 18040
              ************************************************************

Dromedary     CTGCTTTAGCCACTTGCATCTAAGAGACTGAGGAAGTGACATATAGGGTGCTTAAGGAAC 17877
Wild          CTGCTTTAGCCACTTGCATCTAAGAGACTGAGGAAGTGACATATAGGGTGCTTAAGGAAC 17678
Domestic      CTGCTTTAGCCACTTGCATCTAAGAGACTGAGGAAGTGACATATAGGGTGCTTAAGGAAC 18100
              ************************************************************

Dromedary     TTACTCAGTTAAACCATTAGTTTATCTCAAGAGCTGGGAGTTGAGCCCTGGTTGTCTGAC 17937
Wild          TTACTCAGTTAAACCATTAGTTTATCTCAAGAGCTGGGAGTTGAGCCCTGGTTGTCTGAC 17738
Domestic      TTACTCAGTTAAACCATTAGTTTATCTCAAGAGCTGGGAGTTGAGCCCTGGTTGTCTGAC 18160
              ************************************************************

Dromedary     CCCACCCCCCATCCCCTTAATCGCTAGGCTATACTGCCTCTTTCCAACTTGAGGTGTTAT 17997
Wild          CCCACCCCCCATCCCCTTAATCGCTAGGCTATACTGCCTCTTTCCAACTTGAGGTGTTAT 17798
Domestic      CCCACCCCCCATCCCCTTAATCGCTAGGCTATACTGCCTCTTTCCAACTTGAGGTGTTAT 18220
              ************************************************************

Dromedary     TTCCATGGTAAACTAGTTTTGGAACTTGCTTATAGTAGATTAGGTCAGGCATCTTGGGGA 18057
Wild          TTCCATGGTAAACTAGTTTTGGAACTTGCTTATAGTAGATTAGGTCAGGCATCTTGGGGA 17858
Domestic      TTCCATGGTAAACTAGTTTTGCAACTTGCTTATAGTAGATTAGGTCAGGCATCTTGGGGA 18280
              ********************* **************************************

Dromedary     AAAAAAAAATTAATCCACCTACATACACTCATGCAAAGTTAAATAGCCATGTGAGAGTTT 18117
Wild          AAAAAAAAATTAATCCACCTACATACACTCATGCAAAGTTAAATAGCCATGTGAGACTTT 17918
Domestic      AAAAAAAAATTAATCCACCTACATACACTCATGCAAAGTTAAATAGCCATGTGAGACTTT 18340
              ******************************************************** ***

Dromedary     AAGAATAAAGCAAGCCCAGTTCTGCCAGATAGGAAACTAAGTCCAGGTGGCTGGCTCCCA 18177
Wild          AAGAATAAAGCAAGCCCAGTTCTGCCAGATAGGAAACTAAGTCCAGGTGGCTGGCTCCCA 17978
Domestic      AAGAATAAAGCAAGCCCAGTTCTGCCAGATAGGAAACTAAGTCCAGGTGGCTGGCTCCCA 18400
              ************************************************************

Dromedary     CCTCACATCCAGCTCTGGGCAGGCCACAGGAGGGAGGAAGACATTTAGGAACCTAGAGCC 18237
Wild          CCTCACATCCAGCTCTGGGCAGGCCACAGGAGGGAGGAAGACATTTAGGAACCTAGAGCC 18038
Domestic      CCTCACATCCAGCTCTGGGCAGGCCACAGGAGGGAGGAAGACATTTAGGAACCTAGAGCC 18460
              ************************************************************

Dromedary     AGGCTTAGCAATGGAAACAAACCCTGTGAGTGGTCCTTAGGGATCAGAAGGTAGCTGAAG 18297
Wild          AGGCTTAGCAATGGAAACAAACCCTGTGAGTGGTCCTTAGGGATCAGAAGGTAGCTGAAG 18098
Domestic      AGGCTTAGCAATGGAAACAAACCCTGTGAGTGGTCCTTAGGGATCAGAAGGTAGCTGAAG 18520
              ************************************************************

Dromedary     TCGCAATGGGGAGAGAAAACGATGGTGAGGGTGGAGGGAAGAGCCAGGGGGCCACATGGA 18357
Wild          TCGCAATGGGGAGAGAAAACGATGGTGAGGGTGGAGGGAAGAGCCAGGGGGCCACATGGA 18158
Domestic      TCGCAATGGGGAGAGAAAACGATGGTGAGGGTGGAGGGAAGAGCCAGGGGGCCACATGGA 18580
              ************************************************************

Dromedary     TTGAGGTAGAGTGTGCTAATAGTAAATTCTTTTTTCTCATTTTTCTGTGGGAACCTAAAA 18417
Wild          TTGAGGTAGAGTGTGCTAATAGTAAATTCTTTTTTCTCATTTTTCTGTGGGAACCTAAAA 18218
Domestic      TTGAGGTAGAGTGTGCTAATAGTAAATTCTTTTTTCTCATTTTTCTGTGGGAACCTAAAA 18640
              ************************************************************

Dromedary     CTGGAGTTGTAATCATAGTCAGCCAAACTGGGACCAGAATGAGAATACAGAGAACACACG 18477
Wild          CTGGAGTTGTAATCATAGTCAGCCAAACTGGGACCAGAATGAGAATACAGAGAACACACG 18278
Domestic      CTGGAGTTGTAATCATAGTCAGCCAAACTGGGACCAGAATGAGAATACAGAGAACACACG 18700
              ************************************************************
```

```
Dromedary      TCTTCTGTTTAACGTTGCAAGTTTGACCAACACGAAAGGCTTGCAAGTTTGAGTAGAAAT  18537
Wild           TCTTCTGTTTAACGTTGCAAGTTTGACCAACACGAAAGGCTTGCAAGTTTGAGTAGAAAT  18338
Domestic       TCTTCTGTTTAACGTTGCAAGTTTGACCAACACGAAAGGCTTGCAAGTTTGAGTAGAAAT  18760
               ************************************************************

Dromedary      TAGAGCTCTGCACAACAAGGCATTTGGTGAGATTTCACAGACATGCACACACACACGCAC  18597
Wild           TAGAGCTCTGCACAACAAGGCATTTGGTGAGATTTCACAGACATGCACACACACACGCAC  18398
Domestic       TAGAGCTCTGCACAACAAGGCATTTGGTGAGATTTCACAGACATGCACACACACACGCAC  18820
               ************************************************************

Dromedary      ATACTGCATGAGCACAAACCTCCCCGACCCAGAACAGTGTCCGTTACATTGATCTCATGG  18657
Wild           ATACTGCATGAGCACAAACCTCCCCGACCCAGAACAGTGTCCGTTACATTGATCTCATGG  18458
Domestic       ATACTGCATGAGCACAAACCTCCCCGACCCAGAACAGTGTCCGTTACATTGGTCTCATGA  18880
               *************************************************** ******* 

Dromedary      AGATGAATCTCCTGGGATGATCAACTGGGAACAGCCATGTGAAAATAGGGTAGAGCCTGG  18717
Wild           AGATGAATCTCCTGGGATGATCAACTGGGAACAGCCATGTGAAAATAGGGTAGAGCCTGG  18518
Domestic       AGATGAATCTCCTGGGATGATCAACTGGGAACAGCCATGTGAAAATAGGGTAGAGCCTGG  18940
               ************************************************************

Dromedary      ACCTAGCTATTGGGAAGCCAGAGGAGGTAGTGCCCAACTGGCCCCTGAGCAGCTCCCCAT  18777
Wild           ACCTAGCTATTGGGAAGCCAGAGGAGGTAGTGCCCAACTGGCCCCTGAGCAGCTCCCCAT  18578
Domestic       ACCTAGCTATTGGGAAGCCAGAGGAGGTAGTGCCCAACTGGCCCCTGAGCAGCTCCCCAT  19000
               ************************************************************

Dromedary      TCCTTACTTGCTGACCAGGAAAGAAAATAAAAAATAAAGCTATTTGCTTTGTGATATGAA  18837
Wild           TCCTTACTTGCTGACCAGGAAAGAAAATAAAAAATAAAGCTATTTGCTTTGTGATATGAA  18638
Domestic       TCCTTACTTGCTGACCAGGAAAGAAAATAAAAAATAAAGCTATTTGCTTTGTGATATGAA  19060
               ************************************************************

Dromedary      TAGTGCCTCTAATCGAGGGATTGAGAATACACCAAGAGAGAAGAGAATATACAGTTGAGA  18897
Wild           TAGTGCCTCTAATCGAGGGATTGAGAATACACCAAGAGAGAAGAGAATACACAGTTGAGA  18698
Domestic       TAGTGCCTCTAATCGAGGGATTGAGAATACACCAAGAGAGAAGAGAATACACAGTTGAGA  19120
               ************************************************* **********

Dromedary      ATACACCCTTGAAAGGGAAAGTAGGAAATAACGGAACTCAGATTAAGAAGCACACACATC  18957
Wild           ATACACCCTTGAAAGGGAAAGTAGGAAATAACGGAACTCAGATTAAGAAGCACACACATC  18758
Domestic       ATACACCCTTGAAAGGGAAAGTAGGAAATAACGGAACTCAGATTAAGAAGCACACACATC  19180
               ************************************************************

Dromedary      TCTTCTCTCTTCTCCTCTCCTTTCCTCTCCACTTTTCTTCTCTCCCCCGTACCCATGCAC  19017
Wild           TCTTCTCTCTTCTCCTCTCCTTTCCTCTCCACTTTTCTTCTCTCCCCCATACCCATGCAC  18818
Domestic       TCTTCTCTCTTCTCCTCTCCTTTCCTCTCCACTTTTCTTCTCTCCCCCGTACCCATGCAC  19240
               ************************************************ ***********

Dromedary      AAATGCATGCATACACACACATACACACTTACACTCACATTCCCTATGAGAAATACAAGC  19077
Wild           AAATGCATGCATACACACACATACACACTTACACTCACATTCCCTATGAGAAATACAAGC  18878
Domestic       AAATGCATGCATACACACACATACACACTTACACTCACATTCCCTATGAGAAATACAAGC  19300
               ************************************************************

Dromedary      CAAGCCTTAGGCTTTTGTTCAAGGTAAATGTCACTAACCAAGGCAATATATTGTGCCAAA  19137
Wild           CAAGCCTTAGGCTTTTGTTCAAGGTAAATGTCACTAACCAAGGCAATATATTGTGCCAAA  18938
Domestic       CAAGCCTTAGGCTTTTGTTCAAGGTAAATGTCACTAACCAAGGCAATATATTGTGCCAAA  19360
               ************************************************************

Dromedary      AAATAAAGATGAGCTTAATGGAAAAAACAGTTCGATCTCTAAGGAACATAAGCATTTTGA  19197
Wild           AAATAAAGATGAGCTTAATGGAAAAAACAGTTCGATCTCTAAGGAACATAAGCATTTTGA  18998
Domestic       AAATAAAGATGAGCTTAATGGAAAAAACAGTTCGATCTCTAAGGAACATAAGCATTTTGA  19420
               ************************************************************

Dromedary      AATAAAGATAACAAATTCAAACAAAGTACAACAAACTTTAACAACATCAAAGACAGAAAA  19257
Wild           AATAAAGATAACAAATTCAAACAAAGTACAACAAACTTTAACAACATCAAAGACAGAAAA  19058
Domestic       AATAAAGATAACAAATTCAAACAAAGTACAACAAACTTTAACAACATCAAAGACAGAAAA  19480
               ************************************************************

Dromedary      AATTCTAAAAGGCTTCCAGGGGATAAAAGAAGAAATAATAACAAAAAAGAAATTAAAAAA  19317
Wild           AATTCTAAAAGGCTTCCAGGGGATAAAAGAAGAAATAATAACAAAAAAGAAATTAAAAAA  19118
Domestic       AATTCTAAAAGGCTTCCAGGGGATAAAAGAAGAAATAATAACAAAAAAGAAATTAAAAAA  19540
               ************************************************************
```

```
Dromedary      ACCTTACTGAAAAAAGTAGGAATCAGGCATTAAACTTCCAATAGTAATACTTAGAAGACA  19377
Wild           ACCTCACTGAAAAAAGTAGGAATCAGGCATTAAACTTCCAATAGTAATACTTAGAAGACA  19178
Domestic       ACCTCACTGAAAAAAGTAGGAATCAGGCATTAAACTTCCAATAGTAATACTTAGAAGACA  19600
               **** *******************************************************

Dromedary      CTGGAGTTATATTTTCAAAGTCTGGAAGGAAAAAGCATTTAAACCTAGACTCTTATTTCC  19437
Wild           CTAGAGTTATATTTTCAAAGTCTGGAAGGAAAAAGCATTTAAACCTAGACTCTTATTTCC  19238
Domestic       CTGGAGTTATATTTTCAAAGTCTGGAAGGAAAAAGCATTTAAACCTAGACTCTTATTTCC  19660
               ** *********************************************************

Dromedary      ATATAAACTATAAATGCAAGGAGAAATAAATATTACAAAAAGTTTATCATACATCCTCTT  19497
Wild           ATATAAACTATAAATGCAAGGAGAAATAAATATTACAAAAAGTTTATCATACATCCTCTT  19298
Domestic       ATATAAACTATAAATGCAAGGAGAAATAAATATTACAAAAAGTTTATCATACATCCTCTT  19720
               ************************************************************

Dromedary      TGAAATAATTATTATAGGTCATACTTCCAGGAAAAGAGCTATGAATCCAGTAGCAGGCAG  19557
Wild           TGAAATAATTATTATAGGTCATACTTCCAGGAAAAGAGCTATGAATCCAGTAGCAGGCAG  19358
Domestic       TGAAATAATTATTATAGGTCATACTTCCAGGAAAAGAGCTATGAATCCAGTAGCAGGCAG  19780
               ************************************************************

Dromedary      TGAGATATGTGTGAATGAGTAAAGCATTGAGCACTTTATTATTGTTTAAATGAGCAATTA  19617
Wild           TAAGATATGTGTGAATGAGTAAAGCACTGAGCACTTTATTATTGTTTAAATGAGCAATTA  19418
Domestic       TAAGATATGTGTGAATGAGTAAAGCACTGAGCACTTTATTATTGTTTAAATGAGCAATTA  19840
               * ************************* ********************************

Dromedary      CTACAAATAAACCCACTTGATCAATATGAATATGGAAGTTAAATGCTAAATGATACAAGT  19677
Wild           CTACAAATAAACCCACTTGATCAATATGAATATGGAAGTTAAATGCTAAATGATACAAGT  19478
Domestic       CTACAAATAAACCCACTTGATCAATATGAATATGGAAGTTAAATGCTAAATGATACAAGT  19900
               ************************************************************

Dromedary      AAGAAAGGAGTCTGGGGTGCACCGATATACGTCAAAGTGTGTTAAGAACAAGATAAGATG  19737
Wild           AAGAAAGGAGTCTGGGGTGCACCGATATACGTCAAAGTGTGTTAAGAACAAGATAAGATG  19538
Domestic       AAGAAAGGAGTCTGGGGTGCACCGATATACGTCAAAGTGTGTTAAGAACAAGATAAGATG  19960
               ************************************************************

Dromedary      ATTGGTTTATTTTGGGTAAAAGATTGAGATATTGAAAAACTTTAGGTAATGCCCCTGGTT  19797
Wild           ATTGGTTTATTTTGGGTAAAAGATTGAGATATTGAAAAACTTTTGGTAATGCCCCTAGTT  19598
Domestic       ATTGGTTTATTTTGGGTAAAAGATTGAGATATTGAAAAACTTTTGGTAATGCCCCTGGTT  20020
               ******************************************* ************ ***

Dromedary      CAGTAACTTAGGGGTAACTACCAGAAGAATAAAAGTAGAATAAGTAACTTTCAAAACAGC  19857
Wild           CAGTAACTTAGGGATAACTACCAGAAGAATAAAAGTAGAATAAGTAACTTTCAAAACAGC  19658
Domestic       CAGTAACTTAGGGATAACTACCAGAAGAATAAAAGTAGAATAAGTAACTTTCAAAACAGC  20080
               ************* **********************************************

Dromedary      AGAAGCAAACTGATTATCCAATAAATGGCAGGAAAGGAGACAAAGAACAAAACTCAGGAA  19917
Wild           AGAAGCAAACTGATTATCCAATAAATGGCAGGAAAGGAGACAAAGAACAAAACTCAGGAA  19718
Domestic       AGAAGCAAACTGATTATCCAATAAATGGCAGGAAAGGAGACAAAGAACAAAACTCAGGAA  20140
               ************************************************************

Dromedary      GAAACCATGGTGTCTTTTCTTAGCTTCCCTCAAAGAAGAGAGCCTGAATCAAGGTCATGT  19977
Wild           GAAACCATGGTGTCTTTTCTTAGCTTCCCTCAAAGAAGAGAGCCTGAATCAAGGTCATGT  19778
Domestic       GAAACCATGGTGTCTTTTCTTAGCTTCCCTCAAAGAAGAGAGCCTGAATCAAGGTCATGT  20200
               ************************************************************

Dromedary      ATGCAGGTAGTTTACTTTGGAAAAGAACCCCTAGAGATGGGAATAAGGGGCTGGCAACAG  20037
Wild           ATGCAGGTAGTTTACTTTGGAAAAGAACCCCTAGAGATGGGAATAAGGGGCTGGCAACAG  19838
Domestic       ATGCAGGTAGTTTACTTTGGAAAAGAACCCCTAGAGATGGGAATAAGGGGCTGGCAACAG  20260
               ************************************************************

Dromedary      TGAAAGAATGAGGGAAAGCCAACTTAAGAGTGCATTATCAAGTTAATCAGCATAGTGGGA  20097
Wild           TGAAAGAATGAGGGAAAGCCAACTTAAGAGTGCATTATCAAGTTAATCAGCATAGTGGGC  19898
Domestic       TGAAAGAATGAGGGAAAGCCAACTTAAGAGTGCATTATCAAGTTAATCAGCATAGTGGGC  20320
               ***********************************************************

Dromedary      AACTAGAGCTCAATCCACTAGGGTCTCCTGAGGACCCATGTAGAATGCACTTCAGAATTA  20157
Wild           AACTAGAGCTCAATCCACTAGGGTCTCCTGAGGACCCATGTAGAATGCACTTCAGAATTA  19958
Domestic       AACTAGAGCTCAATCCACTAGGGTCTCCTGAGGACCCATGTAGAATGCACTTCAGAATTA  20380
               ************************************************************
```

```
Dromedary      TCTACCCAAGGGATAGAAGAGAGGACTATCTATCCTCCAGCCCATATGCTTCACTTATCA  20217
Wild           TCTACCAAAGGGATAGAAGAGAGGACTATCTATTCTCCAGCCCATATGCTTCACTTATCA  20018
Domestic       TCTACCAAAGGGATAGAAGAGAGGACTATCTATCCTCCAGCCCATATGCTTCACTTATCA  20440
               ****** ************************** **************************

Dromedary      AAGGTTACTCATGGTTGTTAACTCATCTGAACTTCTTGGTTTGCCCATGTCACAATGGTG  20277
Wild           AAGGTTACTCATGGTTGTTAACTCATCTGAACTTCTTGGTTTGCCCATGTCACAATGGTG  20078
Domestic       AAGGTTACTCATGGTTGTTAACTCATCTGAACTTCTTGGTTTGCCCATGTCACAATGGTG  20500
               ************************************************************

Dromedary      AGAGGTTCTTGCCAATGTCCCAGGCAGTGGTGGTAGTGAAGTCCTAGAGTAGAAAGCAGA  20337
Wild           AGAGGTTCTTGCAGATGTCCCAGGCAGTGGTGGTAGTGAAGTCCTAGAGTAGAAAGCAGA  20138
Domestic       AGAGGTTCTTGCAGATGTCCCAGGCAGTGGTGGTAGTGAAGTCCTAGAGTAGAAAGCAGA  20560
               ************  **********************************************

Dromedary      GACCCAGTGTGCAGCCAAGACAACATGCTGTCAGGTTACACCTGTATCCAGGCGGTGGCT  20397
Wild           GACCCAGTGTGCAGCCAAGACAACATGCTGTCAGGTTACACCTGTATCCAGGTGGTGGCT  20198
Domestic       GACCCAGTGTGCAGCCAAGACAACATGCTGTCAGGTTACACCTGTATCCAGGTGGTGGCT  20620
               **************************************************** *******

Dromedary      TTAACAATGACTGAAGTAAAAAGATGAGCCAAAAGATGGCAGCAATAATTGTAATTATAG  20457
Wild           TTAACAATGACTGAAGTAAAAAGATGAGCCAAAAGATGGCAGCAATAATTGTAATTATAG  20258
Domestic       TTAACAATGACTGAAGTAAAAAGATGAGCCAAAAGATGGCAGCAATAATTGTAATTATAG  20680
               ************************************************************

Dromedary      TCACTGTAAATGGATTCAACTTATTAGATTAAAGAAATGCAGGACTGTGTTATTTAAACT  20517
Wild           TCACTGTAAATGGATTCAACTTATTAGATCAAAGAAATGCAGGACTGTGTT-TTTAAACT  20317
Domestic       TCACTGTAAATGGATTCAACTTATTAGATTAAAGAAATGCAGGACTGTGTTATTTAAACT  20740
               ***************************** ********************* ********

Dromedary      TCAAAGGGCACATACAAAATATTAATAAACTGTGCTTCCAAAGACTTGACACAACCTGTA  20577
Wild           TCAAAGGGCACATACAAAATATTAATAAACTGTGCTTCCAAAGACTTGACACAACCTGTA  20377
Domestic       TCAAAGGGCACATACAAAATATTAATAAACTGTGCTTCCAAAGACTTGACACAACCTGTA  20800
               ************************************************************

Dromedary      TTCTCTGACTCTAAGGAAATAAATTAGAATGGAAAAACATTCACAATATTCACAAGGTTG  20637
Wild           TTCTCTGACTCTAAGGAAATAAATTAGAATGGAAAAACATTCACAATATTCACAAGGTTG  20437
Domestic       TTCTCTGACTCTAAGGAAATAAATTAGAATGGAAAAACATTCACAATATTCACAAGGTTG  20860
               ************************************************************

Dromedary      TCCTTCTCCTCCTCCTTTCTTGGTTATAGCAAATTATATCACTGGATGAGTCCTTCTATT  20697
Wild           TCCTTCTCCTCCTCCTTTCTTGGTTATAGCAAATTATATCACTGGATGAGTCCTTCTATT  20497
Domestic       TCCTTCTCCTCCTCCTTTCTTGGTTATAGCAAATTATATCACTGGATGAGTCCTTCTATT  20920
               ************************************************************

Dromedary      GAACAAATATAAATATATTGGACAAATAATTTTTAAAAATCTTCTTAAAATTACTGCAGA  20757
Wild           GAACAAATATAAATATATTGGACAAATAATTTTAAAAAATCTTCTTAAAATTACTGCAGA  20557
Domestic       GAACAAATATAAATATATTGGACAAATAATTTTAAAAAATCTTCTTAAAATTACTGCAGA  20980
               ********************************* **************************

Dromedary      GCTAACAAGATTGTGAGAAATTACCAGGTGAAGCCTGAAGGAGGCAGAGGAACCTAAAGA  20817
Wild           GCTAACAAGATTGTGAGAAATTACCAGGCGAAGCCTGAAGGAGGCAGAGGAACCTAAAGA  20617
Domestic       GCTAACAAGATTGTGAGAAATTACCAGGCGAAGCCTGAAGGAGGCAGAGGAACCTAAAGA  21040
               **************************** *******************************

Dromedary      GGCGACCCAATACAGAGTCCTCTTTTGGCCTGAAGGTGTTAACCAATCCCAGCAAATCTG  20877
Wild           GGCGACCCAATACAGAGTCCTCTTTTGGCCTGAAGGTGTTAACCAATCCCAGCAAATCTG  20677
Domestic       GGCGACCCAATACAGAGTCCTCTTTTGGCCTGAAGGTGTTAACCAATCCCAGCAAATCTG  21100
               ************************************************************

Dromedary      AGCTTTCATTCTGATAACTTCATTTGAGTCAGAAGACCAGGGGGAAAAAAAAGCAAGGGT  20937
Wild           AGCTTTCATTCTGATAACTTCATTTGAGTCAGAAGACCAGGGGGAAAAAAAAGCAAGGGT  20737
Domestic       AGCTTTCATTCTGATAACTTCATTTGAGTCAGAAGACCAGGGGGAAAAAAAAGCAAGGGT  21160
               ************************************************************

Dromedary      CAGACAAGATGAGGAATCTAATACTAGACATTTCCCACAATAAGCTGAGATCTCAAAGAC  20997
Wild           CAGACAAGATGAGGAATCTAATACTAGACTTTTCCCACAATAAGCTGAGATCTCAAAGAC  20797
Domestic       CAGACAAGATGAGGAATCTAATACTAGACTTTTCCCACAATAAGCTGAGATCTCAAAGAC  21220
               ***************************** ******************************
```

```
Dromedary       ACTCTAAGACTAAGGGTTAACTAGAAATGAACCAACACATTTGTATTACTTCAGTATTCA  21057
Wild            ACTCTAAGACTAAGGGTTAACTAGAAATGAACCAACACATTTGTATTACTTCAGTATTCA  20857
Domestic        ACTCTAAGACTAAGGGTTAACTAGAAATGAACCAACACATTTGTATTACTTCAGTATTCA  21280
                ************************************************************

Dromedary       TTTCCATCATTGAGGTAGACCAAGGGACCTTAAGTCTTGAACTTGCTTTAATGTGGCCCT  21117
Wild            TTTCCATCACTGAGGTAGACCAAGGGACCTTAAGTCTTGAACTTGCTTTAATGTGGCCCT  20917
Domestic        TTTCCATCACTGAGGTAGACCAAGGGACCTTAAGTCTTGAACTTGCTTTAATGTGGCCCT  21340
                ********* **************************************************

Dromedary       GTTATGGTGATAAAATTAATGTCCCTAGATGTCCCTAGGTATCTGGCAGAAGTCAATTCA  21177
Wild            GTTATGGTGATAAAATTAATGTCCCTAGATGTCCCTAGGTATCTGGCAGAAGTCAATTCA  20977
Domestic        GTTATGGTGATAAAATTAATGTCCCTAGATGTCCCTAGGTATCTGGCAGAAGTCAATTCA  21400
                ************************************************************

Dromedary       CACTTTCTCTGGAGTAAAAGTTACTTTATTTTAGATCTCAGAATTTCCTGTAATATTTTC  21237
Wild            CACTTTCTCTGGAGTAAAAGTTACTTTATTTTAGATCTCAGAATTTCCTGTAATATTTTC  21037
Domestic        CACTTTCTCTGGAGTAAAAGTTACTTTATTTTAGATCTCAGAATTTCCTGTAATATTTTC  21460
                ************************************************************

Dromedary       TAAGTACAATGGCTACAAGGCAGCCAAAGATAACGAAGTTAATAAACAAAACACTACAAA  21297
Wild            TAAGTACAATGGCTACAAGGCAGCCAAAGATAACGAAGTTAATAAACAAAACACTACAAA  21097
Domestic        TAAGTACAATGGCTACAAGGCAGCCAAAGATAACGAAGTTAATAAACAAAACACTACAAA  21520
                ************************************************************

Dromedary       CAAGAACCGGGGAAAACAGCAGACAAAAGAAACAGATCTGAAGACCGTCAGAAACCAGAA  21357
Wild            CAAGAACCGGGGAAAACAGCAGACAAAAGAAACAGATCTGAAGACCGTCAGAAACCAGAA  21157
Domestic        CAAGAACCGGGGAAAACAGCAGACAAAAGAAACAGATCTGAAGACCGTCAGAAACCAGAA  21580
                ************************************************************

Dromedary       TTATCAGCCACAGACTATAAAACAACTATGTTTAAACAAATAAGACAAATTTGTGTGTGT  21417
Wild            TTATCAGCCACAGACTATAAAACAACTATGTTTAAACAAATAAGACAAATTTGTGTGTGT  21217
Domestic        TTATCAGCCACAGACTATAAAACAACTATGTTTAAACAAATAAGACAAATTTGTGTGTGT  21640
                ************************************************************

Dromedary       GTGTGGTGGGGGGGTCTGCAGGGGAGAAGAACCTATGAAAACTGATAGATCATATTCAAA  21477
Wild            GTGTGGTGGGGGGGTCTGCAGGGGAGAAGAACCTATGAAAACTGATAGATCATATTCAAA  21277
Domestic        GTGTGGTGGGGGGGTCTGCAGGGGAGAAGAACCTATGAAAACTGATAGATCATATTCAAA  21700
                ************************************************************

Dromedary       AGGAAGAAAACAAAACCTTCAGAACTGAAAAATACAATGTCCCAAAGTAAGAACTCAGTG  21537
Wild            AGGAAGAAAACAAAACCTTCAGAACTGAAAAATACAATGTCCCAAAGTAAGAACTCAGTG  21337
Domestic        AGGAAGAAAACAAAACCTTCAGAACTGAAAAATACAATGTCCCAAAGTAAGAACTCAGTG  21760
                ************************************************************

Dromedary       GACAGATTAAATAGTGATTACAAACAGCTGAAGAGAGAACTGGTATAAGATAAGAAGAAA  21597
Wild            GACAGATTAAATAGTGATTACAAACAGCTGAAGAGAGAACTGGTATAAGATAAGAAGAAA  21397
Domestic        GACAGATTAAATAGTGATTACAAACAGCTGAAGAGAGAACTGGTATAAGATAAGAAGAAA  21820
                ************************************************************

Dromedary       ATATCCAGAATGCAGCCCTGGGAGATGAAAAGATGGAATATACAGAAGAAAAAGTAATTT  21657
Wild            ATATCCAGAATGCAGCCCTGGGAGATGAAAAGATGGAATATACAGATGAAAAAGTAATTT  21457
Domestic        ATATCCAGAATGCAGCCCTGGGAGATGAAAAGATGGAATATACAGATGAAAAAGTAATTT  21880
                ********************************************** *************

Dromedary       GTTTAAAGGATAGAGTAGATGGTCTAACATAATTAATTGGAGTCTCAGAAGGAAATATGA  21717
Wild            GTTTAAAGGATAGAGTAGATGGTCTAACATAATTAATTGGAGTCTCAGAAGGAAATATGA  21517
Domestic        GTTTAAAGGATAGAGTAGATGGTCTAACATAATTAATTGGAGTCTCAGAAGGAAATATGA  21940
                ************************************************************

Dromedary       AAGAGACCAATATTTTAAAAGACAACTGAGAATTTTACGGAATTTATGAAAGACACTATT  21777
Wild            AAGAGACCAATATTTTAAAAGACAACTGAGAATTTTACGGAATTTATGAAAGACACTATT  21577
Domestic        AAGAGACCAATATTTTAAAAGACAACTGAGAATTTTATGGAATTTATGAAAGACACTATT  22000
                ************************************* **********************

Dromedary       TTATAAGTTCACAAAATCCAAATAACCAACAGTCAAATTTATAGGTGATTTCTGTACAGC  21837
Wild            TTATAAGTTCACAAAATCCAAATAACCAACAGTCAAATTTATAGGTGATTTCTGTACAGC  21637
Domestic        TTATAAGTTCACAAAATCCAAATAACCAACAGTCAAATTTATAGGTGATTTCTGTACAGC  22060
                ************************************************************
```

```
Dromedary      AATGATGGAAAACTAATAGTAAGTAAAATGGTATCTTTGTTGTACTGAAAAATAATAGTC  21897
Wild           AATGATGGAAAACTAACAGTAAGTAAAATGGTATCTTTGTTGTACTGAAAAATAATAGTC  21697
Domestic       AATGATGGAAAACTAACAGTAAGTAAAATGGTATCTTTGTTGTACTGAAAAATAATAGTC  22120
               **************** *******************************************

Dromedary      AAACTAGATTCAGCAACACTATTTTTCAAGAATAAAGGCAAAATAAAGACACTTTGTGAC  21957
Wild           AAACTAGATTCAGCAACACTATTTTTCAAGAATAAAGGCAAAATAAAGACACTTTGTGAC  21757
Domestic       AAACTAGATTCAGCAACACTATTTTTCAAGAATAAAGGCAAAATAAAGACACTTTGTGAC  22180
               ************************************************************

Dromedary      AAAAACGAAAGCATTTAGCACAAATACACTCACAGTATAGACAATTCTAAAGCATTGGCC  22017
Wild           AAAAATGAAAGCATTTAGCACAAATACACTCACAGTATAGACAATTCTAAAGCATTGGCC  21817
Domestic       AAAAATGAAAGCATTTAGCACAAATACACTCACAGTATAGACAATTCTAAAGCATTGGCC  22240
               ***** ******************************************************

Dromedary      GAAGAAATGAAGAATGAAGAAAATGATAACTATGTAAGTAAACCTACATAAATGACGGGT  22077
Wild           GAAGAAATGAAGAATGAAGAAAATGATAACTATGTAAGTAAACCTACATAAATGACGGGT  21877
Domestic       GAAGAAATGAAGAATGAAGAAAATGATAACTATGTAAGTAAACCTACATAAATGACGGGT  22300
               ************************************************************

Dromedary      GACATAAAACAATAGGAATAATGTCTTATAAAGTTTAAAAAATTATGTATTTTTCCTGAA  22137
Wild           GACATAAAACAATAGGAATAATGTCTTATAAAGTTTAAAAAATTATGTATTTTTCCTGAA  21937
Domestic       GACATAAAACAATAGGAATAATGTCTTATAAAGTTTAAAAAATTATGTATTTTTCCTGAA  22360
               ************************************************************

Dromedary      ATAGTGAACCTGGTAATTCCAATCAATGCACCTGCTGAGAACAACTAGAAAAGCTGGTAT  22197
Wild           ATAGTGAACCTGGTAATTCCAATCAATGCACCTGCTGAGAACAACTAGAAAAGCTGGTAT  21997
Domestic       ATAGTGAACCTGGTAATTCCAATCAATGCACCTGCTGAGAACAACTAGAAAAGCTGGTAT  22420
               ************************************************************

Dromedary      TAGAGAGCCTGTGAATAATTACAGGGCTAAGGTTCAGGAGAAGAAAGAAACAGATAGGAA  22257
Wild           TAGAGAGCCTGTGAATAATTACAGGGCTAAGGTTCAGGAGAAGAAAGAAACAGATAGGAA  22057
Domestic       TAGAGAGCCTGTGAATAATTACAGGGCTAAGGTTCAGGAGAAGAAAGAAACAGATAGGAA  22480
               ************************************************************

Dromedary      GCCTGCTATTTGTAACATCTATTAATTTCTGAAAAATCAGCTAATAGGCTGAAAGCTGAA  22317
Wild           GCCTGCTATTTGTAACATCTATTAATTTCTGAAAAATCAGCTAATAGGCTGAAAGCTGAA  22117
Domestic       GCCTGCTATTTGTAACATCTATTAATTTCTGAAAAATCAGCTAATAGGCTGAAAGCTGAA  22540
               ************************************************************

Dromedary      GTGAGCTTTTCATAGATTCAAAGGGTTAGTAAGACAAAAATGAAGTTCAGGACCAATTAA  22377
Wild           GTGAGCTTTTCATAGATTCAAAGGGTTAGTAAGACAAAAATGAAGTTCAGGACCAATTAA  22177
Domestic       GTGAGCTTTTCATAGATTCAAAGGGTTAGTAAGACAAAAATGAAGTTCAGGACCAATTAA  22600
               ************************************************************

Dromedary      GTGGTAATGTAGGGCTAGTAACACTAGCTTAAATTAGCATCCCAAAATGCTACATGCTAG  22437
Wild           GTGGTAATGTAGGGCTAGTAACACTAGCTTAAATTAGCATCCCAAAATGCTACATGCTAG  22237
Domestic       GTGGTAATGTAGGGCTAGTAACACTAGCTTAAATTAGCATCCCAAAATGCTACATGCTAG  22660
               ************************************************************

Dromedary      GAATAAATGAACTAAAATAGAAGAGCCAGCTTCAAATCACCTCAATAACTGGATTGAATT  22497
Wild           GAATAAATGAACTAAAATAGAAGAGCCAGCTTCAAATCACCTCAATAACTGGATTGAATT  22297
Domestic       GAATAAATGAACTAAAATAGAAGAGCCAGCTTCAAATCACCTCAATAACTGGATTGAATT  22720
               ************************************************************

Dromedary      AAAGTAATCTGGGATCGCTAGTGCCCCTGGACATCTGCCTGCAAGAATAAATCTTTTCTG  22557
Wild           AAAGTAATCTGGGATCGCTAGTGCCCCTGGACATCTGCCTGCAAGAATAAATCTTTTCTG  22357
Domestic       AAAGTAATCTGGGATCGCTAGTGCCCCTGGACATCTGCCTGCAAGAATAAATCTTTTCTG  22780
               ************************************************************

Dromedary      GAGGAAAAACAGCATCATTCTGCCCCTCAAATTTTTCTACAATTTTTCATATCCAATTCC  22617
Wild           GAGGAAAAACAGCATCATTCTGCCCCTCAAATTTTTCTACAATTTTTCATATCCAATTCC  22417
Domestic       GAGGAAAAACAGCATCATTCTGCCCCTCAAATTTTTCTACAATTTTTCATATCCAATTCC  22840
               ************************************************************

Dromedary      AGCACTCAATATAGAAAACAAAACATAGTAAGGAACACGCTAAAAAAGTAAGAGAAACAA  22677
Wild           AGCACTCAATATAGAAAACAAAACATAGTAAGGAACACGCTAAAAAAGTAAGAGAAACAA  22477
Domestic       AGCACTCAATATAGAAAACAAAACATAGTAAGGAACACGCTAAAAAAGTAAGAGAAACAA  22900
               ************************************************************
```

```
Dromedary      TAGATAATAGAAAGAGATGCACAAGGATACAGATATTATTAGATGTAGATACTTAAAATA 22737
Wild           TAGATAATAGAAAGAGATGCACAAGGATACAGATATTATTAGATGTAGATACTTAAAATA 22537
Domestic       TAGATAATAGAAAGAGATGCACAAGGATACAGATATTATTAGATGTAGATACTTAAAATA 22960
               ************************************************************

Dromedary      ACGTTTAATATTTTCAAGGAACTACAAATAAAATTTAGATTTTGGCAGGGAACTAAAAAC 22797
Wild           ACGTTTAATATTTTCAAGGAACTACAAATAAAATTTAGATTTTGGCAGGGAACTAAAAAC 22597
Domestic       ACGTTTAATATTTTCAAGGAACTACAAATAAAATTTAGATTTTGGCAGGGAACTAAAAAC 23020
               ************************************************************

Dromedary      TATATGAAAGAGCTGAGTAGAAATTCTAGAACTAAGAAGTACAATATCAGCTCAATGTAT 22857
Wild           TATATGAAAGAGCTGAGTAGAAATTCTAGAACTAAGAAGTACAATATCAGCTCAATGTAT 22657
Domestic       TATATGAAAGAGCTGAGTAGAAATTCTAGAACTAAGAAGTACAATATCAGCTCAATGTAT 23080
               ************************************************************

Dromedary      TTTTTTCAAGGCAGATTACTCACGGCTCAAGAGAAAATTAACGAACTAGAATACTGACTA 22917
Wild           TTTTTTCAAGGCAGATTACTCACGGCTCAAGAGAAAATTAACGAACTAGAATACTGACTA 22717
Domestic       TTTTTTCAAGGCAGATTACTCACGGCTCAAGAGAAAATTAACGAACTAGAATACTGACTA 23140
               ************************************************************

Dromedary      CAAGAAAATATTCAAACTAAAGGATAAAGAGAAGAAAGAATGGAAAATAGAGAAAACAGT 22977
Wild           CAAGAAAATATTCAAACTAAAGGATAAAGAGAAGAAAGAATGGAAAATAGAGAAAACAGT 22777
Domestic       CAAGAAAATATTCAAACTAAAGGATAAAGAGAAGAAAGAATGGAAAATAGAGAAAACAGT 23200
               ************************************************************

Dromedary      GTAAAAAACTAGAAATTACAGTGGGGTAGTCTGAAATATAGGTAATTAGAGTTCCAGAAA 23037
Wild           GTAAAAAACTAGAAATTACAGTGGGGTAGTCTGAAATATAGGTAATTAGAGTTCCAGAAA 22837
Domestic       GTAAAAAACTAGAAATTACAGTGGGGTAGTCTGAAATATAGGTAATTAGAGTTCCAGAAA 23260
               ************************************************************

Dromedary      GAGAGGAGAGAGAAAATGGGATAGAGGCAACATTTGAAGAGGTGACAGCTGATAATTTTT 23097
Wild           GAGAGGAGAGAGAAAATGGGATAGAGGCAACATTTGAAGAGGTGACAGCTGGTAATTTTT 22897
Domestic       GAGAGGAGAGAGAAAATGGGATAGAGGCAACATTTGAAGAGGTGA-AGCTGGTAATTTTT 23319
               ********************************************* ***** ********

Dromedary      CAAATAAGCCAGATTCAAAAGAACGATGAACTGAAAGCAGAATAAACAAAAGAAACACAC 23157
Wild           CAAATAAGCCAGATTCAAAAGAACGATGAACTGAAAGCAGAATAAACAAAACAAACACAC 22957
Domestic       CAAATAAGCCAGATTCAAAAGAACGATGAACTGAAAGCAGAATAAACAAAACAAACACAC 23379
               *************************************************** ********

Dromedary      TAGGGCAAACAATTATAAAACTGCTAAAACAAAGACAAAGAGAAAAAAATCTTAAAAGCA 23217
Wild           TAGGGCAAACAATTATAAAACTGCTAAAACAAAGACAAAGAGAAAAAAATCTTAAAAGCA 23017
Domestic       TAGGGCAAACAATTATAAAACTGCTAAAACAAAGACAAAGAGAAAAAAATCTTAAAAGCA 23439
               ************************************************************

Dromedary      GATTACCTACACACATACACAAAAAGCAATACAATTCACAGCTGTCTTCTCAACAGAAAA 23277
Wild           GATTACCTACACACATACACAAAAAGCAATACAATTCACAGCTGTCTTCTCAACAGAAAA 23077
Domestic       GATTACCTACACACATACACAAAAAGCAATACAATTCACAGCTGTCTTCTCAACAGAAAA 23499
               ************************************************************

Dromedary      AATAAAAATAAAAAAGAACCTAGAAGACAATAAAATGATAGCTTTGAAGAGCTGGAAAAA 23337
Wild           AATAAAAATAAAAAAGAACCTAGAAGACAATAAAATGATAGCTTTGAAGGGCTGGAAAAA 23137
Domestic       AATAAAAATAAAAAAGAACCTAGAAGACAATAAAATGATAGCTTTGAAGGGCTGGAAAAA 23559
               ************************************************* **********

Dromedary      ATTCCATAACCAACTAAAATGTCCTCAAAAATGAACATTAAATAAGGATATTTTCAGACA 23397
Wild           ATTCCATAACCAACTAAAATGTCCTCAAAAATGAACATTAAATAAGGATATTTTCAGACA 23197
Domestic       ATTCCATAACCAACTAAAATGTCCTCAAAAATGAACATTAAATAAGGATATTTTCAGACA 23619
               ************************************************************

Dromedary      AATAAAGCAGAGAGAACTGGTCACCAAAAGACCCATTCTAGATGCAGAAGGAAAATGTCA 23457
Wild           AATAAAGCAGAGAGAACTGGTCACCAAAAGACCCATTCTAAATGCAGAAGGAAAATGTCA 23257
Domestic       AATAAAGCAGAGAGAACTGGTCACCAAAAGACCCATTCTAAATGCAGAAGGAAAATGTCA 23679
               **************************************** *******************

Dromedary      TATGGAGACTTGGAGATGTAGGAGAGAGTGAAGAGTGAATAATAACTATCTGGGTAAATT 23517
Wild           TATGGAGACTTGGAGATGTAGGAGAGAGTGAAGAATGAATAATAACTATCTGGGTAAATT 23317
Domestic       TATGGAGACTTGGAGATGTAGGAGAGAGTGAAGAATGAATAATAACTATCTGGGTAAATT 23739
               ********************************** *************************
```

```
Dromedary      AAATAAGTTATAAACAAAATCGTATATATATAATTCCTTTTGAATAGAAAAGTGTGTGTA  23577
Wild           AAATAAGTTATAAACAAAATCGTATATATATAATTCCTTTTGAATAGAAAAGTGTGTGTA  23377
Domestic       AAATAAGTTATAAACAAAATCGTATATATATAATTCCTTTTGAATAGAAAAGTGTGTGTA  23799
               ************************************************************

Dromedary      TACAAACATTTTATCCACACATATATGTATGTATATGTATATATATGCATGCATGCTTAT  23637
Wild           TACAAACATTTTATCCACACATATATGTATGTATATGTATATATATGCATGCATGCTTAT  23437
Domestic       TACAAACATTTTATCCACACATATATGTATGTATATGTATATATATGCATGCATGCTTAT  23859
               ************************************************************

Dromedary      GTATATATGCATAGACCTACATACCTATATGTACACATATATGCCTGTGTATTATGCTGA  23697
Wild           GTATATATGCATAGACCTACATACCTATATGTACACATATATGCCTGTGTATTATGCTGA  23497
Domestic       GTATATATGCATAGACCTACATACCTATATGTACACATATATGCCTGTGTATTATGCTGA  23919
               ************************************************************

Dromedary      GTGTCCTTAGTGAGACTCTTGATGTTTTTATTGGATTGTCC--CTCTCAGTAACTAATTT  23755
Wild           GTGTCCTTAGTGAGACTCTTGATGTTTTTATTGGATTGTCCCTCTCTCAGTAACTAATTT  23557
Domestic       GTGTCCTTAGTGAGACTCTTGATGTTTTTATTGGATTGTCCCTCTCTCAGTAACTAATTT  23979
               *****************************************  *****************

Dromedary      AAATAAGTTAAAGAGAACATCATGAGAAAAATTTCAAAATATTTAGAACTCAATGAAAAG  23815
Wild           AAATAAGTTAAAGAGAACATCATGAGAAAAATTTCAAAATATTTAGAACTCAATGAAAAG  23617
Domestic       AAATAAGTTAAAGAGAACATCATGAGAAAAATTTCAAAATATTTAGAACTCAATGAAAAG  24039
               ************************************************************

Dromedary      GATTATTAAGAGAAAAAGGGAGGAAGACATAAATAACAATATTCTGAACAAAAAAAGTTA  23875
Wild           GATTATTAAGAGAAAAAGGGAGGAAGACATAAATAACAATATTCTGAACAAAAAAAGTTA  23677
Domestic       GATTATTAAGAGAAAAAGGGAGGAAGACATAAATAACAATATTCTGAACAAAAAAAGTTA  24099
               ************************************************************

Dromedary      AAATAGACAAATGAATAACAGATTTTTAAAAATTATGACAGAATACCATGAACAAGTTAG  23935
Wild           AAATAGACAAATGAATAACAGATTTTTAAAAATTATGACAGAATACCATGAACAAGTTAG  23737
Domestic       AAATAGACAAATGAATAACAGATTTTTAAAAATTATGACAGAATACCATGAACAAGTTAG  24159
               ************************************************************

Dromedary      TGAATCTGAAAACTTGATGAAATGAAAACAAAAATTGAAAAAGATAAAATTTCAAAATTG  23995
Wild           TGAATCTGAAAACTTGATGAAATGAAAACAAAAATTGAAAAAGATAAAATTTCAAAATTG  23797
Domestic       TGAATCTGAAAACTTGATGAAATGAAAACAAAAATTGAAAAAGATAAAATTTCAAAATTG  24219
               ************************************************************

Dromedary      CCCAAGTGAAAGAAAACTTGAATAGACCAATTGGTACTAAGAAAATCTATTGAAAAATTT  24055
Wild           CCCAAGTGAAAGAAAACTTGAATAGACCAATTGGTACTAAGAAAATCTATTGAAAAATTT  23857
Domestic       CCCAAGTGAAAGAAAACTTGAATAGACCAATTGGTACTAAGAAAATCTATTGAAAAATTT  24279
               ************************************************************

Dromedary      TTTTAACTCTTTTAAAACTGGCTATGTCTATACTGTTTTACAGGTAAGATCCACAAGAAA  24115
Wild           TTTTAACTCTTTTAAAACTGGCTATGTCTATACTGTTTTACAGGTAAGATCCACAAGAAA  23917
Domestic       TTTTAACTCTTTTAAAACTGGCTATGTCTATACTGTTTTACAGGTAAGATCCACAAGAAA  24339
               ************************************************************

Dromedary      CCCATAATCTCCAATTAGTGGGAAAGCTACTCAATTAATTTTATAATACTAGTATACTCT  24175
Wild           CCCATAATCTCCAATTAGTGGGAAAGCTACTCAATTAATTTTATAATACTAGTATACTCT  23977
Domestic       CCCATAATCTCCAATTAGTGGGAAAGCTACTCAATTAATTTTATAATACTAGTATACTCT  24399
               ************************************************************

Dromedary      TCACATAAAATCTGATAAGGATAGGAGAAGAAAAGGAAATTATAGGCCAATTTCACATAT  24235
Wild           TCACATAAAATCTGATAAGGATAGGAGAAGAAAAGGAAATTATAGGCCAATTTCACATAT  24037
Domestic       TCACATAAAATCTGATAAGGATAGGAGAAGAAAAGGAAATTATAGGCCAATTTCACATAT  24459
               ************************************************************

Dromedary      AAAAATTGACGCAAAAATACTAAACAAAATATTAAATAATAGAATCCAATGTGCTATAAT  24295
Wild           AAAAATTGACGCAAAAATACTAAACAAAATATTAAATAATAGAATCCAATGTGCTATAAT  24097
Domestic       AAAAATTGACGCAAAAATACTAAACAAAATATTAAATAATAGAATCCAATGTGCTATAAT  24519
               ************************************************************

Dromedary      AAATATTAATAATGACAACAAAAGCAACAACACACTCTGATCAAGTAGGATTCACACCTG  24355
Wild           AAATATTAATAATGACAACAAAAGCAACAACACACTCTGATCAAGTAGGATTCACACCTG  24157
Domestic       AAATATTAATAATGACAACAAAAGCAACAACACACTCTGATCAAGTAGGATTCACACCTG  24579
               ************************************************************
```

```
Dromedary      GAAGTAAAGTTGGAAGGAGGGAAGGATCAACTTAACAGATGTAGAAAAAGAATCTGACAA  24415
Wild           GAAGTAAAGTTGGAAGGAGGGAAGGACCAACTTAACAGATGTAGAAAAAGAATCTGACAA  24217
Domestic       GAAGTAAAGTTGGAAGGAGGGAAGGACCAACTTAACAGATGTAGAAAAAGAATCTGACAA  24639
               ************************** *********************************

Dromedary      AGCCCAACAGCTATATATGATAAATAGCTCATAAGAAAACTAGAAATGGAAGGGAATTCC  24475
Wild           AGCCCAACAGCTATATATGATAAATAGCTCATAAGAAAACTAGAAATGGAAGGGAATTCC  24277
Domestic       AGCCCAACAGCTATATATGATAAATAGCTCATAAGAAAACTAGAAATGGAAGGGAATTCC  24699
               ************************************************************

Dromedary      CTTGCTTTGATAAAATTGTCTATCAAAAACCTACAGCAAGAGTCATACTAAAGGAAAAAC  24535
Wild           CTTGCTTTGATAAAATTGTCTATCAAAAACCTACAGCAAGAGTCATACTAAAGGAAAAAC  24337
Domestic       CTTGCTTTGATAAAATTGTCTATCAAAAACCTACAGCAAGAGTCATACTAAAGGAAAAAC  24759
               ************************************************************

Dromedary      TTTAAAAGCATTCCCTTTGCATTCAGGAACAAGATAAAGATCCTCATTATTATTGATATA  24595
Wild           TTTAAAAGCATTCCCTTTGCATTCAGGAACAAGATAAAGATCCTCATTATTATTGATATA  24397
Domestic       TTTAAAAGCATTCCCTTTGCATTCAGGAACAAGATAAAGATCCTCATTATTATTGATATA  24819
               ************************************************************

Dromedary      GACACTGTAGGTCCTGGTCAATAAAAGACAACACCAAAAATAAAAAAGAAATAAGAGGTA  24655
Wild           GACACTGTAGGTCCTGGTCAATAAAAGACAACACCAAAAATAAAAAAGAAATAAGAGGTA  24457
Domestic       GACACTGTAGGTCCTGGTCAATAAAAGACAACACCAAAAATAAAAAAGAAATAAGAGGTA  24879
               ************************************************************

Dromedary      GAACATGAAGTTAGCACAGAAAAAATTTGTAGCTTCCCCCCAACAATTACAAAACAAGACA  24715
Wild           GAACATGAAGTTCGCACAGAAAAAATTTGTAGCTTCCCCCCAACAATTACAAAACAAGACA  24517
Domestic       GAACATGAAGTTCGCACAGAAAAAATTTGTAGCTTCCCCCCAACAATTACAAAACAAGACA  24939
               ************ ***********************************************

Dromedary      TCACAACAGTAACAAAAACGTTAGTAGTCCCTCTACATAAACCAACAATGAAAACATAAG  24775
Wild           TCACAACAGTAACAAAAACGTTAGTAGTCCCTCTACATAAACCAACAATGAAAACATAAG  24577
Domestic       TCACAACAGTAACAAAAACGTTAGTAGTCCCTCTACATAAACCAACAATGAAAACATAAG  24999
               ************************************************************

Dromedary      GAAAACCTATCTGTTAAAATCCTTGAAAGGTTAAAAATAAGGTTACAAAATTGCATTAAA  24835
Wild           GAAAACCTATCTGTTAAAATCCTTGAAAGGTTAAAAATAAGGTTACAAAATTGCATTAAA  24637
Domestic       GAAAACCTATCTGTTAAAATCCTTGAAAGGTTAAAAATAAGGTTACAAAATTGCATTAAA  25059
               ************************************************************

Dromedary      ATTTACAAGTAAGATGAATAATAAGAGGGATATACTATGTATATGACTGGAATCATGACA  24895
Wild           ATTTACAAGTAAGCTGAATAATAAGAGGGATATACTATGTATATGACTGGAATCATGACA  24697
Domestic       ATTTACAAGTAAGCTGAATAATAAGAGGGATATACTATGTATATGACTGGAATCATGACA  25119
               ************* **********************************************

Dromedary      AGTATAAGTGAGAAGGAAATAAGTTATTCTAAATATAGGAATATTAAATCTGAAAACAGA  24955
Wild           AGTGTAAGTGAGAAGGAAATAAGTTATTCTAAATATAGGAATATTAAATCTGAAAACAGA  24757
Domestic       AGTGTAAGTGAGAAGGAAATAAGTTATTCTAAATATAGGAATATTAAATCTGAAAACAGA  25179
               *** ********************************************************

Dromedary      ATGTTGAGAGAAAAAAATAAACAGAAGCAAATTTGTACAATTTATGTAAAATTTATTAACA  25015
Wild           ATGTTGAGAGAAAAAAATAAACAGAAGCAAATTTGTACAATTTATGTAAAATTTATTAACA  24817
Domestic       ATGTTGAGAGAAAAAAATAAACAGAAGCAAATTTGTACAATTTATGTAAAATTTATTAACA  25239
               ************************************************************

Dromedary      AACAAAAACTATACTATGTATTTTTAAGCATATGTATGTGTGCATGTCCATACTGACACA  25075
Wild           AACAAAAACTATACTATGTATTTTTAAGCATATGTATGTGTGCATGTCCATACTGACACA  24877
Domestic       AACAAAAACTATACTATGTATTTTTAAGCATATGTATGTGTGCATGTCCATACTGACACA  25299
               ************************************************************

Dromedary      CGCACACATTCCTGATGAACTCTATTTCTTGATCATGATGTGTTTTTATATAAACCCATA  25135
Wild           TGCACACATTCCTGATGAACTCTATTTCTTGATCATGATGTGTTTTTATATATACCCATA  24937
Domestic       TGCACACATTCCTGATGAACTCTATTTCTTGATCATGATGTGTTTTTATATATACCCATA  25359
                *************************************************** *******

Dromedary      TATTCTATTTTAAGGACAAACATTAAGGACCCAGGAGGCTTATTACTGCCTGTACAAGTG  25195
Wild           TATTCTATTTTAAGAACAAACATTAAGGACCCAGGAGGCTTATTACTGCCTGTACAAGTG  24997
Domestic       TATTCTATTTTAAGAACAAACATTAAGGACCCAGGAGGCTTATTACTGCCTGTACAAGTG  25419
               ************** *********************************************
```

```
Dromedary     CCTTAAACGGTAACCTAGACTGGTCCTGAAAGATTTAATCTCTCTAAATCCATAGTTAAG  25255
Wild          CCTTAAACGGTAACCTAGACTGGTCCTGAAAGATTTAATCTCTCTAAATCCATAGTTAAG  25057
Domestic      CCTTAAACGGTAACCTAGACTGGTCCTGAAAGATTTAATCTCTCTAAATCCATAGTTAAG  25479
              ************************************************************

Dromedary     TTAATCTAGTTTAGTTGGAAGAGATGATGCTGGCATACTCACTCAGAATGATAATAAAAA  25315
Wild          TTAATCTAGTTTAGTTGGAAGAGATGATGCTGGCATACTCACTCAGAATGATAATAAAAA  25117
Domestic      TTAATCTAGTTTAGTTGGAAGAGATGATGCTGGCATACTCACTCAGAATGATAATAAAAA  25539
              ************************************************************

Dromedary     TCCTCCAAATGCATTTATAGTGTAATATCATACCTCTGCTTGTGTTTATTAATGCACTAT  25375
Wild          TCCTCCAAATGCATTTATAGTGTAATATCATACCTCTGCTTGTGTTTATTAATGCACTAT  25177
Domestic      TCCTCCAAATGCATTTATAGTGTAATATCATACCTCTGCTTGTGTTTATTAATGCACTAT  25599
              ************************************************************

Dromedary     CACTTTATGGCTAATGGACACTGGGCACTCCGAGGCTCAGTCTCCCATCTGAGATGATGT  25435
Wild          CACTTTATGGCTAATGGACACTGGGCACTCCAAGGCTCAGTCTCCCATCTGAGATGATGT  25237
Domestic      CACTTTATGGCTAATGGACACTGGGCACTCCAAGGCTCAGTCTCCCATCTGAGATGATGT  25659
              ******************************* ****************************

Dromedary     ACTACTCAGTAACTCGATTACCAGTGATGGAATCGAGATTTCATTTCTGGGGTGGAAACA  25495
Wild          ACTACTCAGTAACTCGATTACCAGTGATGGAATCGAGATTTCATTTCTGGGGTGGAAACA  25297
Domestic      ACTACTCAGTAACTCGATTACCAGTGATGGAATCGAGATTTCATTTCTGGGGTGGAAACA  25719
              ************************************************************

Dromedary     AGCAAAGTAGCAGACAGACTCCAGCTCAGGTTGTGTTTTGTTTTGTGTTCAAAGTAGGCA  25555
Wild          AGCAAAGTAGCAGACAGACTCCAGCTCAGGTTGTGTTTTGTTTTGTGTTCAAAGTAGGCA  25357
Domestic      AGCAAAGTAGCAGACAGACTCCAGCTCAGGTTGTGTTTTGTTTTGTGTTCAAAGTAGGCA  25779
              ************************************************************

Dromedary     TTTTGACGAGGTATCTGGGCCACCTGGCAGCTCCCACTCACCCCTCCATTTGTAATCACC  25615
Wild          TTTTGACGAGGTATCTGGGCCACCTGGCAGCTCCCACTCACCCCTCCATTTGTAATCACC  25417
Domestic      TTTTGACGAGGTATCTGGGCCACCTGGCAGCTCCCACTCACCCCTCCATTTGTAATCACC  25839
              ************************************************************

Dromedary     TTGCTGTGCCCACATCTACACCAGACTTTTCCCTGTGCTCTGCGGGGTTCTCTGCTCTTC  25675
Wild          TTGCTGTGCCCACATCTACACCAGACTTTTCCCTGTGCTCTGCGGGGTTCTCTGCTCTTC  25477
Domestic      TTGCTGTGCCCACATCTACACCAGACTTTTCCCTGTGCTCTGCGGGGTTCTCTGCTCTTC  25899
              ************************************************************

Dromedary     CAGAAGCCTAAGTTACCTGCCCTTATCCACAGAATTAGAATCCTTGCTTTCCAGTTCTTG  25735
Wild          CAGAAGCCTAAGTTACCTGCCCTTATCCACAGAATTAGAATCCTTGCTTTCCAATTCTTG  25537
Domestic      CAGAAGCCTAAGTTACCTGCCCTTATCCACAGAATTAGAATCCTTGCTTTCCAATTCTTG  25959
              ***************************************************** ******

Dromedary     ACATCCCTGTGACATCACTGTACTCAGATTCTCACAGAGAATGAAGTTCTTTCTAATCTT  25795
Wild          ACATCCCTGTGACATCACTGTACTCAGATTCTCACAGAGAATGAAGTTCTTTCTAATCTT  25597
Domestic      ACATCCCTGTGACATCACTGTACTCAGATTCTCACAGAGAATGAAGTTCTTTCTAATCTT  26019
              ************************************************************

Dromedary     TGGAAATATTAGGAGTCATAAAGAAATCGTGCTGATCTAGCACCCTTTCTTTGAGCTTCT  25855
Wild          TGGAAATATTAGGAGTCATAAAGAAATCGTGCTGATCTAGCACCCTTTCTTTGAGCTTCT  25657
Domestic      TGGAAATATTAGGAGTCATAAAGAAATCGTGCTGATCTAGCACCCTTTCTTTGAGCTTCT  26079
              ************************************************************

Dromedary     AAGCGTCTGAGAGTTTGACCCTGGGACTCAGTGGAGACCCTCTGCCTCAATCAGTGTGCC  25915
Wild          AAGCGTCTGAGAGTTTGACCCTGGGACTCAGTGGAGACCCTCTGCCTCAATCAGTGTGCC  25717
Domestic      AAGCGTCTGAGAGTTTGACCCTGGGACTCAGTGGAGACCCTCTGCCTCAATCAGTGTGCC  26139
              ************************************************************

Dromedary     CCACATTTGTAATCTCCTTTGGCAGACTTACATGTGTGTAACAAAGGCTAAGGATGGTTC  25975
Wild          CCACATTTGTAATCTCCTTTGGCAGACTTACATGTGTGTAACAAAGGCTAAGGATGGTTC  25777
Domestic      CCACATTTGTAATCTCCTTTGGCAGACTTACATGTGTGTAACAAAGGCTAAGGATGGTTC  26199
              ************************************************************

Dromedary     TCAGAGATGGTAGCCATAGCAGCAGACAGTGCCAGCACCCATTAACAGCTTTAAGACATG  26035
Wild          TCAGAGATGGTAGCCATAGCAGCAGACAGTGCCAGCACCCATTAACAGCTTTAAGACATG  25837
Domestic      TCAGAGATGGTAGCCATAGCAGCAGACAGTGCCAGCACCCATTAACAGCTTTAAGACATG  26259
              ************************************************************
```

```
Dromedary      ACCAGAAGCATTTCATTGCCCAATTTTATCTTCTTAACCATCACTGATTATAATTTCTGT 26095
Wild           ACCAGAAGCATTTCATTGCCCAATTTTATCTTCTTAACCATCACTGATTATAATTTCTGT 25897
Domestic       ACCAGAAGCATTTCATTGCCCAATTTTATCTTCTTAACCATCACTGATTATAATTTCTGT 26319
               ************************************************************

Dromedary      ATCATTTTTGAGACCAAGTGAATCTATGGTAATAAATAATAATGTTATTTAAGTTACATA 26155
Wild           ATCATTTTTGAGACCAAGTGAATCTATGGTAATAAATAATAATGTTATTTAAGTTACATA 25957
Domestic       ATCATTTTTGAGACCAAGTGAATCTATGGTAATAAATAATAATGTTATTTAAGTTACATA 26379
               ************************************************************

Dromedary      GTCATCCTATATAAGACAAGGAAATAAACTGCTATTTTTATTATTATTCTATTAAAGTAT 26215
Wild           GTCATCCTATATAAGACAAGGAAATAAACTGCTATTTTTATTATTATTCTATTAAAGTAT 26017
Domestic       GTCATCCTATATAAGACAAGGAAATAAACTGCTATTTTTATTATTATTCTATTAAAGTAT 26439
               ************************************************************

Dromedary      CCACTACTGTATTTGTTCACCTTATTTGCTATCATTATTATCCACTCTCTTTATTGTAAT 26275
Wild           CCACTACTGTATTTGTTCACCTTATTTGCTATCATTATTATCCACTCTCTTTATTGTAAT 26077
Domestic       CCACTACTGTATTTGTTCACCTTATTTGCTATCATTATTATCCACTCTCTTTATTGTAAT 26499
               ************************************************************

Dromedary      TTTGATTTCAACCTACATCTGAGGAAGTCTGGGCTAAATCCGGGGTTTAAAAGAAGTAAA 26335
Wild           TTTGATTTCAACCTACATCTGAGGAAGTCTGGGCTAAATCCGGGGTTTAAAAGAAGTAAA 26137
Domestic       TTTGATTTCAACCTACATCTGAGGAAGTCTGGGCTAAATCCGGGGTTTAAAAGAAGTAAA 26559
               ************************************************************

Dromedary      GGGCCTTTAAAAAAAAATAAATACTTCATTCACACCCTGATGTCTAAGGGAAACTAGTGTA 26395
Wild           GGGCCTTTAAAAAAAAATAAATACTTCATTCACACCCTGATGTCTAAGGGAAACTAGTGTA 26197
Domestic       GGGCCTTTAAAAAAAAATAAATACTTCATTCACACCCTGATGTCTAAGGGAAACTAGTGTA 26619
               ************************************************************

Dromedary      GAACAGGACGGAAAACCCACTGTTTTGTAAGAGTCTGGAAAACAATTGCTCTTTATTCTC 26455
Wild           GAACAGGACGGAAAACCCACTGTTTTGTAAGAGTCTGGAAAACAATTGCTCTTTATTCTC 26257
Domestic       GAACAGGACGGAAAACCCACTGTTTTGTAAGAGTCTGGAAAACAATTGCTCTTTATTCTC 26679
               ************************************************************

Dromedary      TGTGCAAATTTGAGTACAATGATGTCATGGGGATGCTTGGCCATTTTATAGGTGATTTAT 26515
Wild           TGTGCAAATTTGAGTACAATGATGTCATGGGGATGCTTGGCCATTTTATAGGTGATTTAT 26317
Domestic       TGTGCAAATTTGAGTACAATGATGTCATGGGGATGCTTGGCCATTTTATAGGTGATTTAT 26739
               ************************************************************

Dromedary      TGTCTTACAATGTTATATTGCGTCTGAATATTGTCAGTTTTCAAAATAAAGGTAATTTTC 26575
Wild           TGTCTTACAATGTTATATTGCGTCTGAATATTGTCAGTTTTCAAAATAAAGGTAATTTTC 26377
Domestic       TGTCTTACAATGTTATATTGCGTCTGAATATTGTCAGTTTTCAAAATAAAGGTAATTTTC 26799
               ************************************************************

Dromedary      ATGTTACTGAAAGGAAATAATAATATTTGTTGGCCAAATATACCCTTATTATGTGATACT 26635
Wild           ATGTTACTGAAAGGAAATAATAATATTTGTTGGCCAAATATACCCTCATTATGTGATACT 26437
Domestic       ATGTTACTGAAAGGAAATAATAATATTTGTTGGCCAAATATACCCTCATTATGTGATACT 26859
               ********************************************** *************

Dromedary      TATGGGAGCAGCAGCTATGACCGAGTGGCCCAGACATTCAGATGTTGGGCCACAGTATTT 26695
Wild           TATGGGAGCAGCAGCTATGACCAAGTGGCCCAGACATTCAGATGTTGGGTCACAGTATTT 26497
Domestic       TATGGGAGCAGCAGCTATGACCAAGTGGCCCAGACATTCAGATGTTGGGTCACAGTATTT 26919
               ********************** ************************** **********

Dromedary      TGTTGTGATTTTCTCTAGATCTCACCATATGCCAGGCTGGTGGTTTTCAACATGTGGTTC 26755
Wild           TGTTGTGATTTTCTCTAGATCTCACCATATGCCAGGCTGGTGGTTTTCAACATGTGGTTC 26557
Domestic       TGTTGTGATTTTCTCTAGATCTCACCATATGCCAGGCTGGTGGTTTTCAACATGTGGTTC 26979
               ************************************************************

Dromedary      CTGGGCCAACAGCATCATTATCATAAGAGAACTTGTGAAGATGCAAATTCTCAGGACCTA 26815
Wild           CTGGGCCAACAGCATCATTATCATAAGAGAACTTGTGAAGATGCAAATTCTCAGGACCTA 26617
Domestic       CTGGGCCAACAGCATCATTATCATAAGAGAACTTGTGAAGATGCAAATTCTCAGGACCTA 27039
               ************************************************************

Dromedary      CACCAGATTTCCTGGATCAGAAACTCGAGTTGAGGGGTCCAGCAATCTCTGTTTTAAGAA 26875
Wild           CACCAGATTTCCTGGATCAGAAACTCGAGTTGAGGGGTCCAGCAATCTCTGTTTTAAGAA 26677
Domestic       CACCAGATTTCCTGGATCAGAAACTCGAGTTGAGGGGTCCAGCAATCTCTGTTTTAAGAA 27099
               ************************************************************
```

```
Dromedary     AAACTCCAGGTAATCCTGATGCTTGCTAAGGCTTGAAGGCCACTGCCCCAGGTCAATTAT  26935
Wild          AAACTGCAGGTAATCCTGATGCTTGCTAAGGCTTGAAGGCCACTGCCCCAGGTCAATTAT  26737
Domestic      AAACTGCAGGTAATCCTGATGCTTGCTAAGGCTTGAAGGCCACTGCCCCAGGTCAATTAT  27159
              ***** ******************************************************

Dromedary     ACCCTCAAGATGTAGCACTCATTTATAGCACCATGGAGGGCTAAATGAACCAAATTTTCT  26995
Wild          ACCCTCAAGATGTAGCACTCATTGATAGCACCATGGAGGGCTAAATGAACCAAATTTTCT  26797
Domestic      ACCCTCAAGATGTAGCACTCATTGATAGCACCATGGAGGGCTAAATGAACCAAATTTTCT  27219
              *********************** ************************************

Dromedary     GATGCTGGGCTATTGCTTTTGTTATAAAATTCCCTGGGGCTCATAGGGCAAGTCCACCCC  27055
Wild          GATGCTGGGCTATTGCTTTTGTTATAAAATTCCCTGGGGCTCATAGGGCAAGTCCACCCC  26857
Domestic      GATGCTGGGCTATTGCTTTTGTTATAAAATTCCCTGGGGCTCATAGGGCAAGTCCACCCC  27279
              ************************************************************

Dromedary     TTGAAGTTATAGTTTCTCTAGAAGAAAATGGACAGTCCTACATTGAGAACTCTAGAAAGA  27115
Wild          TTGAAGTTATAGTTTCTCTAGAAGAAAATGGACAGTCCTACATTGAGAACTCTAGAAAGA  26917
Domestic      TTGAAGTTATAGTTTCTCTAGAAGAAAATGGACAGTCCTACATTGAGAACTCTAGAAAGA  27339
              ************************************************************

Dromedary     GGCAGATCCTAGGATGGGAAGACACGTTAGCCTGCAGGTGAATGGGGATTATTTTCTCCT  27175
Wild          GGCAGATCCTAGGATGGGAAGAAACGTTAGCCTGCAGGTGAATGGGGATTATTTTCTCCT  26977
Domestic      GGCAGATCCTAGGATGGGAAGAAACGTTAGCCTGCAGGTGAATGGGGATTATTTTCTCCT  27399
              ********************** *************************************

Dromedary     TCCTAGGCCTAGTGATTATGTGGACCTTGAGGAGTGGAGGGAAAGGTCTGTTCCGTTTAG  27235
Wild          TCCTAGGCCTAGTGATTATGTGGACCTTGAGGAGTGGAGGGAAAGGTCTGTTCCGTTTAG  27037
Domestic      TCCTAGGCCTAGTGATTATGTGGACCTTGAGGAGTGGAGGGAAAGGTCTGTTCCGTTTAG  27459
              ************************************************************

Dromedary     GACAGAAAGGCCAAGTGGAGAAGAGCCCCTCCCCAGCAGCTGGGATGTGGTGGAGCAACC  27295
Wild          GACAGAAAGGCCAAGTGGAGAAGAGCCCCTCCCCAGCAGCTGGGATGTGGTGGAGCAACC  27097
Domestic      GACAGAAAGGCCAAGTGGAGAAGAGCCCCTCCCCAGCAGCTGGGATGTGGTGGAGCAACC  27519
              ************************************************************

Dromedary     TCCCAGCAGAGGGTCTGCTGGAGTTCTGGAAATGAAACTGTCCACCCCATGTAGAGAGAT  27355
Wild          TCCCAGCAGAGGGTCTGCTGGAGTTCTGGAAATGAAACTGTCCACCTCATGTAGAGAGAT  27157
Domestic      TCCCAGCAGAGGGTCTGCTGGAGTTCTGGAAATGAAACTGTCCACTTCATGTAGAGAGAT  27579
              **********************************************  ************

Dromedary     CATGAGACCATTGTCAGCAGAGGTGGTGGCCTGGAAGTTTGTTGAGGAAGAAGATTGTGT  27415
Wild          CATGAGACCATTGTCAGCAGAGGTGGTGGCCTGGAAGTTTGTTGAGGAAGAAGATTGTGT  27217
Domestic      CATGAGACCATTGTCAGCAGAGGTGGTGGCCTGGAAGTTTGTTGAGGAAGAAGATTGTGT  27639
              ************************************************************

Dromedary     GACAATTCTTGGGATACTGTCTTAAAAAAAACCTGTTAAGAGTATAGAGGCTAAAGGCAG  27475
Wild          GACAATTCTTGGGATACTGTCTTAAAAAAAACCTGTTAAGAGTATAGAGGCTAAAGGCAG  27277
Domestic      GACAATTCTTGGGATACTGTCTTAAAAAAAACCTGTTAAGAGTATAGAGGCTAAAGGCAG  27699
              ************************************************************

Dromedary     GTTGGGTTTTGCAGGCAATGTAATGTTGGCAAAGCGAATGAAGGTTATACCCACAGGCTG  27535
Wild          GTTGGGTTTTGCAGGCAATGTAATGTTGGCAAAGCGAATGAAGGTTATACCCACAGGCTG  27337
Domestic      GTTGGGTTTTGCAGGCAATGTAATGTTGGCAAAGCGAATGAAGGTTATACCCACAGGCTG  27759
              ************************************************************

Dromedary     GAGAATCCTAGACCTGCATTTATACCTGCTGGACAGCCTGGGTACCACAAACAACTTCAC  27595
Wild          GAGAATCCTAGACCTGCATTTATACCTGCTGGACAGCCTGGGTACCACAAACAACTTCAC  27397
Domestic      GAGAATCCTAGACCTGCATTTATACCTGCTGGACAGCCTGGGTACCACAAACAACTTCAC  27819
              ************************************************************

Dromedary     TCCTCACTTACTGATCTCTGATTCTAATTCTGACTGCCTGCTTTCCCATATCGCACAAAA  27655
Wild          TCCTCACTTACTGATCTCTGATTCTAATTCTGACTGCCTGCTTTCCCATATCGCACAAAA  27457
Domestic      TCCTCACTTACTGATCTCTGATTCTAATTCTGACTGCCTGCTTTCCCATATCGCACAAAA  27879
              ************************************************************

Dromedary     CACCTACCTTTTCTCTT-ACCCTGGCCTTTTAATACATACAGATCAATGAGATTTGAAAC  27714
Wild          CACCTACCTTTTCTCTTGACCCTGGCCTTTTAATACATACAGATCAATGAGATTTGAAAC  27517
Domestic      CACCTACCTTTTCTCTTGACCCTGGCCTTTTAATACATACAGATCAATGAGATTTGAAAC  27939
              ***************** ******************************************
```

```
Dromedary      GCAGGAAATACAGTTTCACAGTTAATTCAAAGATAATGTAAAAATATATCTTATTAATAT  27774
Wild           GCAGGAAATACAGTTTCACAGTTAATTCAAAGGTAATGTAAAAATATATCTTATTAATAT  27577
Domestic       GCAGGAAATACAGTTTCACAGTTAATTCAAAGGTAATGTAAAAATATATCTTATTAATAT  27999
               ******************************** ***************************

Dromedary      AGATTATTCTTCCATATCTTCTACAATATTATAAAGCACCTATGAAGCAATCAGAAAGTA  27834
Wild           AGATTATTCTTCCATATCTTCTACAATATTATAAAGCACCTATGAAGCAATCAGAAAGTA  27637
Domestic       AGATTATTCTTCCATATCTTCTACAATATTATAAAGCACCTATGAAGCAATCAGAAAGTA  28059
               ************************************************************

Dromedary      AAGAAAGCAGAAATCCCACAGAAGAATTTTGAGAGGGCTAGCCAGACTTTAGCCTCCTCC  27894
Wild           AAGAAAGCAGAAATCCCACAGAAGAATTTTGAGAGGGCTAGCCAGACTTCAGCCTCCTCC  27697
Domestic       AAGAAAGCAGAAATCCCACAGAAGAATTTTGAGAGGGCTAGCCAGACTTCAGCCTCCTCC  28119
               ************************************************* **********

Dromedary      AGTAAGAATTTGAGAACGTGGGACAAATTTCAGTTAGAGTCCCAGTCAACCTATTAGTTC  27954
Wild           AGTAAGAATTTGAGAACGTGGGACAAATTTCAGTTAGAGTCCCAGTCAACCTATTAGTTC  27757
Domestic       AGTAAGAATTTGAGAACGTGGGACAAATTTCAGTTAGAGTCCCAGTCAACCTATTAGTTC  28179
               ************************************************************

Dromedary      CCTTTGACTTTTCTTTGAGAGTCAGAATTTTATGTTTAAGGTTTTTTAGAAGAAAATATT  28014
Wild           CCTTTGA-----CTTTGAGAGTCAGAATTTTATGTTTAAGGTTTTTTAGAAGAAAATATT  27812
Domestic       CCTTTGA-----CTTTGAGAGTCAGAATTTTATGTTTAAGGTTTTTTAGAAGAAAATATT  28234
               *******     ************************************************

Dromedary      TTCTTTTCCTTGATCTTGAAATGAGGGCTTCATGGAATTTTATTCAGAATTCTAAGGAAT  28074
Wild           TTCTTTTCCTTGATCTTGAAATGAGGGCTTCATGGAATTTTATTCAGAATTCTAAGGAAT  27872
Domestic       TTCTTTTCCTTGATCTTGAAATGAGGGCTTCATGGAATTTTATTCAGAATTCTAAGGAAT  28294
               ************************************************************

Dromedary      TCAGAAGAAACAAACAAACAAACAAACAAACAAACTGTAGCTGGGCAACATAGTGTCCAC  28134
Wild           TCAGAAG----AAACAAACAAACAAACAAACAAACTGTAGCTGGGCAACATAGCGTCCAC  27928
Domestic       TCAGAAG----AAACAAACAAACAAACAAACAAACCGTAGCTGGGCAACATAGCGTCCAC  28350
               *******    ************************ ***************** ******

Dromedary      CACCTGTTCCATCACCTGCTTTCTGTTCTCTCTAAACTTTTTAATCTCCCATTCTCTTTC  28194
Wild           CACCTGTTCCATCACCTGCTTTCTGTTCTCTCTAAACTTTTTAATCTCCCATTCTCTTTC  27988
Domestic       CACCTGTTCCATCACCTGCTTTCTGTTCTCTCTAAACTTTTTAATCTCCCATTCTCTTTC  28410
               ************************************************************

Dromedary      TGACCCCATAGACCCCCTCCTAGACACTTCTTATGGAGGTGCCAAATGAACATCTTGTCT  28254
Wild           TGACCCCATAGACCCCCTCCTAGACACTTCTTATGGAGGTGCCAAATGAACATCTTGTCT  28048
Domestic       TGACCCCATAGACCCCCTCCTAGACACTTCTTATGGAGGTGCCAAATGAACATCTTGTCT  28470
               ************************************************************

Dromedary      GAGATCTTCCCTGACTTGTGCCCCTGAATCCAGCCATTTGAATGACTGTAGCACTCTGTC  28314
Wild           GAGATCTTCCCTGACTTGTGCCCCTGAATCCAGCCATTTGAATGACTGTAGCACTCTGTC  28108
Domestic       GAGATCTTCCCTGACTTGTGCCCCTGAATCCAGCCATTTGAATGACTGTAGCACTCTGTC  28530
               ************************************************************

Dromedary      AGGGGATACCACATTCTACAGGGAGGGGTTGCCATATAGGCACCAAGAATAATTCCCAGC  28374
Wild           AGGGGATACCACATTCTACAGGGAGGGGTTGCCATATAGGCACCAAGAATAATTCCCAGC  28168
Domestic       AGGGGATACCACATTCTACAGGGAGGGGTTGCCATATAGGCACCAAGAATAATTCCCAGC  28590
               ************************************************************

Dromedary      TAAGTCATTGGGAATTACATGTGGCCCTTCCAAACATTTCATAGGAATTTTTTTATAATA  28434
Wild           TAAGTCATTGGGAATTACATGTGGCCCTTCCAAACATTTCATAGGAATTTTTTTATAATA  28228
Domestic       TAAGTCATTGGGAATTACATGTGGCCCTTCCAAACATTTCATAGGAATTTTTTTATAATA  28650
               ************************************************************

Dromedary      ACAAATTTAGCTTTCTTTATAAAACTGGAAGTGATTACATTGGTTAATATATCTTTTTGA  28494
Wild           ACAAATTTAGCTTTCTTTATAAAACTGGAAGTGATTACATTGGTTAATATATCTTTTTGA  28288
Domestic       ACAAATTTAGCTTTCTTTATAAAACTGGAAGTGATTACATTGGTTAATATATCTTTTTGA  28710
               ************************************************************

Dromedary      GTTGTCTTTGCATAAAATTTGGACAACTTCTCTCTCAATTTCTATGTGGAGCAGAAACTG  28554
Wild           GTTGTCTTTGCATAAAATTTGGACAACTTCTCTCTCAATTTCTATGTGGAGCAGAAACTG  28348
Domestic       GTTGTCTTTGCATAAAATTTGGACAACTTCTCTCTCAATTTCTATGTGGAGCAGAAACTG  28770
               ************************************************************
```

```
Dromedary      TGTCACCAGAAGAGTTTAGTGGCAGGTGTACTGGATCATCAGTAATGAGCTGGCTGTGTT  28614
Wild           TGTCACCAGAAGAGTTTAGTGGCAGGTGTACTGGATCATCAGTAATGAGCTGGCTGTGTT  28408
Domestic       TGTCACCAGAAGAGTTTAGTGGCAGGTGTACTGGATCATCAGTAATGAGCTGGCTGTGTT  28830
               ************************************************************

Dromedary      AACTTGGGCTAGTTACCTAACTTCTCTGGGCCCTCATGTCTTCATCCATAAAATGAAGGA  28674
Wild           AACTTGGGCTAGTTACCTAACTTCTCTGGGCCCTCATGTCTTCATCCATAAAATGAAGGA  28468
Domestic       AACTTGGGCTAGTTACCTAACTTCTCTGGGCCCTCATGTCTTCATCCATAAAATGAAGGA  28890
               ************************************************************

Dromedary      GTTGGACTCTGATCTTTAACAATATTTAATAGTCTTTAAGGTCTGGTCCAGGCATTAATT  28734
Wild           GTTGGACTCTGATCTTTAACAATATTTAATAGTCTTTAAGGTCTGGTCCAGGCATAAATT  28528
Domestic       GTTGGACTCTGATCTTTAACAATATTTAATAGTCTTTAAGGTCTGGTCCAGGCATAAATT  28950
               ******************************************************* ****

Dromedary      TTTTTCAGTGAAGGTACTGGGGATTGAACCTAGGACCTTGTGCATGTTAAGCATGCACTC  28794
Wild           TTTTTCAGTGAAGGTACTGGGGATTGAACCTAGGACCTTGTGCATGTTAAGCATGCACTC  28588
Domestic       TTTTTCAGTGAAGGTACTGGGGATTGAACCTAGGACCTTGTGCATGTTAAGCATGCACTC  29010
               ************************************************************

Dromedary      TATCACAGAGCTATACCTCTAGCCCAGACATAAATACTTCTGATGCTGTAAAATTCAATT  28854
Wild           TATCACAGAGCTATACCTCTAGCCCAGACATAAATACTTCTGATGCTGTAAAATTCAATT  28648
Domestic       TATCACAGAGCTATACCTCTAGCCCAGACATAAATACTTCTGATGCTGTAAAATTCAATT  29070
               ************************************************************

Dromedary      TGATCTGTTCTTTTGCATCACATCACATTGTCTAGTTGAGTTTCCCGCACAGAATACTCA  28914
Wild           TGATCTGTTCTTTTGCATCACATCACATTGTCTAGTTGAGTTTCCCGCACAGAATACTCA  28708
Domestic       TGATCTGTTCTTTTGCATCACATCACATTGTCTAGTTGAGTTTCCCGCACAGCATACTCA  29130
               **************************************************** *******

Dromedary      TAAGTGTTATTGGATGACTATTAGACCTCGTTTCCAAAACTGTGTCCTGGCATTGCTTTC  28974
Wild           TAAGTGTTATTGGATGACTATTAGACCTCGTTTCCAAAACTGTGTCCTGGCATCGCTTTC  28768
Domestic       TAAGTGTTATTGGATGACTATTAGACCTCGTTTCCAAAACTGTGTCCTGGCATCGCTTTC  29190
               ***************************************************** ******

Dromedary      TTGAGACATCCGTGCCTGACTTTGTATGACTCCACTCTTTCAGCAACGCCCTTCTGGAAC  29034
Wild           TTGAGACATCTGTGCCTGACTTTGTATGACTCCACTCTTTCAGCAACGCCCTTCTGGAAC  28828
Domestic       TTGAGACATCTGTGCCTGACTTTGTATGACTCCACTCTTTCAGCAACGCCCTTCTGGAAC  29250
               ********** *************************************************

Dromedary      AAGCAGTACTCAGGCTGGTGAAGGATGAGGCTGGACATCATCCTGAAATCAAATAGACTC  29094
Wild           AAGCAGTACTCAGGCTGGTGAAGGATGAGGCTGGACATCATCCTGAAATCAAATAGACTC  28888
Domestic       AAGCAGTACTCAGGCTGGTGAAGGATGAGGCTGGACATCATCCTGAAATCAAATAGACTC  29310
               ************************************************************

Dromedary      ATCCAAATAGCCTACTCCAAATTGGCAAACTGGCCGAATTCCATAGTTCTCAAACTAAAA  29154
Wild           ATCCAAATAGCCTACTCC-AATTGGCAAACTGGCCGAATTCCATAGTTCTCAAACTAAAA  28947
Domestic       ATCCAAATAGCCTACTCC-AATTGGCAAACTGGCCGAATTCCATAGTTCTCAAACTAAAA  29369
               ****************** *****************************************

Dromedary      TAAAGACTCATTTTACTTCCTTCTCCAACTTGAAGTCCTGAGACTAAGTAGATGTAAATG  29214
Wild           TAAAGACTCATTTTACTTCCTTCTCCAACTTGAAGTCCTGAGACTAAGTAGATGTAAATG  29007
Domestic       TAAAGACTCATTTTACTTCCTTCTCCAACTTGAAGTCCTGAGACTAAGTAGATGTAAATG  29429
               ************************************************************

Dromedary      AATAAGAAGACAAAAACAATAACAGAACAAAACTTGAATAAAAACTTATTTAAATAATT  29274
Wild           AATAAGAAGACAAAAACAATAACAGAACAAAACTTGAATAAAAACTTATTTAAATAATT  29067
Domestic       AATAAGAAGACAAAAACAATAACAGAACAAAACTTGAATAAAAACTTATTTAAATAATT  29489
               ************************************************************

Dromedary      GCCTATTTTATGCTAGCTTTTGTGTTAGATACTTCAAATGTCTTTTTTGTTTACTATCTT  29334
Wild           GCCTATTTTATGCTAGCTTTTCTGTTAGATACTTCAAATGTCTTTTTTGTTTACTATCTT  29127
Domestic       GCCTATTTTATGCTAGCTTTTCTGTTAGATACTTCAAATGTCTTTTTTGTTTACTATCTT  29549
               ********************* **************************************

Dromedary      AACAATCTGGTGAAGTAAGTACTATTCTTCCCATTTTACAGATAAAGACATGGTCTCAGA  29394
Wild           AACAATCTGGTGAAGTAAGTACTATTCTTCCCATTTTACAGATAAAGACATGGTCTCAGA  29187
Domestic       AACAATCTGGTGAAGTAAGTACTATTCTTCCCATTTTACAGATAAAGACATGGTCTCAGA  29609
               ************************************************************
```

```
Dromedary      GAAATCTGGTGACCTGTCAAAGGTCACACAACTAGTAAAGACAGAATCAATTAATCCCAA  29454
Wild           GAAATCTGGTGACCTGTCAAAGGTCACACAACTAGTAAAGACAGAATCAATTAATCCCAA  29247
Domestic       GAAATCTGGTGACCTGTCAAAGGTCACACAACTAGTAAAGACAGAATCAATTAATCCCAA  29669
               ************************************************************

Dromedary      CTCAGTTTGAACGCCCCTTCCACTATTTTAGATATTGACTAATAAACATGTTAAGAGCAT  29514
Wild           CTCAGTTTGAACGCCCCTTCCACTATTTTAGATATTGACTAATAAACATG-TAAGAGCAT  29306
Domestic       CTCAGTTTGAACGCCCCTTCCACTATTTTAGATATTGACTAATAAACATG-TAAGAGCAT  29728
               ************************************************** *********

Dromedary      AGATATTTGAGTAAAAAAGAACATTATTCCACATAAATATGGCCCTTCTCCTCAGTACAC  29574
Wild           AGATATTTGAGTAAAAAAGAACATTATTCCACATAAATATGGCCCTTCTCCTCAGTACAC  29366
Domestic       AGATATTTGAGTAAAAAAGAACATTATTCCACATAAATATGGCCCTTCTCCTCAGTACAC  29788
               ************************************************************

Dromedary      CTAGAGGTGGCCCACACAGTTGCCAAAGATTACTTAGCAGTCTCTAATTTCAACTCTTCC  29634
Wild           CTAGAGATGGCCCACACAGTTGCCAAAGATTACTTAGCAGTCTCTAATTTCAACTCTTCC  29426
Domestic       CTAGAGATGGCCCACACAGTTGCCAAAGATTACTTAGCAGTCTCTAATTTCAACTCTTCC  29848
               ****** *****************************************************

Dromedary      CTCTCCAGCTTATGTTTCTTGCTGGCAGAATAGTAGGGTCTAGGAATAGAATCTCCTCAG  29694
Wild           CTCTCCAGCTTATGTTTCTTGCTGGCAGAATAGTAGGGTCTAGGAATAGAATCTCCTCAG  29486
Domestic       CTCTCCAGCTTATGTTTCTTGCTGGCAGAATAGTAGGGTCTAGGAATAGAATCTCCTCAG  29908
               ************************************************************

Dromedary      TTGGGTGAATTTAAGCTGTTCCTCAGTGATGGTCAACACTTAACATCACCACGACAAAAA  29754
Wild           TTGGGTGAATTTAAGCTGTTCCTCAGTGATGGTCAACACTTAACATCACCACGACAAAAA  29546
Domestic       TTGGGTGAATTTAAGCTGTTCCTCAGTGATGGTCAACACTTAACATCACCACGACAAAAA  29968
               ************************************************************

Dromedary      ACATACCTCTGGAGTTGTTAGACAATTGTTTACTCTAAATTCTAATGAGAAAACATGTTT  29814
Wild           ACATACCTCTGGAGTTGCTAGACAATTGTTTACTCTAAATTCTAATGAGAAAACATGTTT  29606
Domestic       ACATACCTCTGGAGTTGCTAGACAATTGTTTACTCTAAATTCTAATGAGAAAACATGTTT  30028
               ***************** ******************************************

Dromedary      TAATGAACTATTGATAGTTTCTTTTTTGAGATGGAGACAGAGAATATAATCACTTAAAAG  29874
Wild           TAATGAACTATTGATAGTTTCTTTTTTGAGATGGAGACAGAGAATATAATCACTTAAAAG  29666
Domestic       TAATGAACTATTGATAGTTTCTTTTTTGAGATGGAGACAGAGAATATAATCACTTAAAAG  30088
               ************************************************************

Dromedary      CATTTTGTTGAACCGGTTCCTTTTCTTGGATATTTAGGATGCTGTTGCTTGCTCTGAAGT  29934
Wild           CATTTTGTTGAACCGGTTCCTTTTCTTGGATATTTAGGATGCTGTTGCTTGCTCTGAAGT  29726
Domestic       CATTTTGTTGAACCGGTTCCTTTTCTTGGATATTTAGGATGCTGTTGCTTGCTCTGAAGT  30148
               ************************************************************

Dromedary      ACCTCACCTGGACTATGATTAGGATTGCACAGACAAGAATTAATCTGGATACAAGAGATA  29994
Wild           ACCTCACCTGGACTATGATTAGGATTGCACAGACAAGAATTAATCTGGATACAAGAGATA  29786
Domestic       ACCTCACCTGGACTATGATTAGGATTGCACAGACAAGAATTAATCTGGATACAAGAGATA  30208
               ************************************************************

Dromedary      AAAGAACTTATGGCTGCCAAATTCAGAATCTCCCATGAGCGCAGAAAACAGATGATAAGG  30054
Wild           AAAGAACTTATGGCTGCCAAATTCAGAATCTCCCATGAGCGCAGAAAACAGATGATAAGG  29846
Domestic       AAAGAACTTATGGCTGCCAAATTCAGAATCTCCCATGAGCGCAGAAAACAGATGATAAGG  30268
               ************************************************************

Dromedary      AGAGAGGATAGAAAATGTAAAATGACCAACACCCAGGAGCTAGAAAACAATTAAGAACAA  30114
Wild           AGAGAGGATAGAAAATGTAAAATGACCAACACCCAGGAGCTAGAAAACAATTAAGAACAA  29906
Domestic       AGAGAGGATAGAAAATGTAAAATGACCAACACCCAGGAGCTAGAAAACAATTAAGAACAA  30328
               ************************************************************

Dromedary      AGAAGTCAGCAGAGGCAGAAGACAGATACTGGATGATCAGAATTCTGATTAGGGTAAATA  30174
Wild           AGAAGTCAGCAGAGGCAGAAGACAGATACTGGATGATCAGAATTCTGATTAGGGTAAATA  29966
Domestic       AGAAGTCAGCAGAGGCAGAAGACAGATACTGGATGATCAGAATTCTGATTAGGGTAAATA  30388
               ************************************************************

Dromedary      CCTCCAGAGAGTGGCACAGGTGGTTAGGAAAGCAGGAAGGCGCTTTTTCTAGGGTAGGAA  30234
Wild           CCTCCAGAGAGTGGCACAGGTGGTTAGGAAAGCAGGAAGGCGCTTTTTCTAGGGTAGGAA  30026
Domestic       CCTCCAGAGAGTGGCACAGGTGGTTAGGAAAGCAGGAAGGCGCTTTTTCTAGGGTAGGAA  30448
               ************************************************************
```

```
Dromedary      CTACAGCAATTCAATAAAACTGAATACATTTTATAAGGCTGAGTGTACTTGGAATATATA  30294
Wild           CTACAGCAATTCAATAAAACTGAATACATTTTATAAGGCTGAGTGTACTTGGAATATATA  30086
Domestic       CTACAGCAATTCAATAAAACTGAATACATTTTATAAGGCTGAGTGTACTTGGAATATATA  30508
               ************************************************************

Dromedary      AACTGGCAACCTCCAGGGATAAAATTTTCAGATATCCTTGAATCTCAGTGCCGTTTGGCT  30354
Wild           AACTGGCAACCTCCAGGGATAAAATTTTCAGATATCCTAGAATCTCAGTGCCGTTTGGCT  30146
Domestic       AACTGGCAACCTCCAGGGATAAAATTTTCAGATATCCTAGAATCTCAGTGCCGTTTGGCT  30568
               ************************************** *********************

Dromedary      CCTTAGAACAGATACCATAAAGGCGTATAAGGCCAGGTAGATGTAAGCATAAAAATACAA  30414
Wild           CCTTAGAACAGATACCATAGAGGCGTATAAGGCCAGGTAGATGTAAGCATAAAAATACAA  30206
Domestic       CCTTAGAACAGATACCATAGAGGCGTATAAGGCCAGGTAGATGTAAGCATAAAAATACAA  30628
               ******************* ****************************************

Dromedary      TTAAATCCTAGTATGATGTGTCCATACTTTTGATTTGTATCATGTCTCATATTATATGCC  30474
Wild           TTAAATCCTAGTATGATGTGTCCATACTTTTGATTTGTATCATGTCTCATATTATATGCC  30266
Domestic       TTAAATCCTAGTATGATGTGTCCATACTTTTGATTTGTATCATGTCTCATATTATATGCC  30688
               ************************************************************

Dromedary      TTATGAATAGGAAACATAATATATTCTCGCCTCTTTTGCTATAGTTTAAAAACTTTATAA  30534
Wild           TTATGAATAGGAAACATAATATATTCTCGCCTCTTTTGCTATAGTTTAAAAACTTTATAA  30326
Domestic       TTATGAATAGGAAACATAATATATTCTCGCCTCTTTTGCTATAGTTTAAAAACTTTATAA  30748
               ************************************************************

Dromedary      ATTAGGTGCCAATCTTGTCTCACCACATGAATACTGCAGTTAAGCTCCATTGTTCCTACT  30594
Wild           ATTAGGTGCCAATCTTGTCTCACCACATGAATACTGCAGTTAAGCTCCATTGTTCCTACT  30386
Domestic       ATTAGGTGCCAATCTTGTCTCACCACATGAATACTGCAGTTAAGCTCCATTGTTCCTACT  30808
               ************************************************************

Dromedary      TATGCTTCTGGCACTAGTTCTTTGTCATTCATTCCAGTCCTCATTCATTCATTGTTCAAG  30654
Wild           TATGCTTCTGGCACTAGTTCTTTGTCATTCATTCCAGTCCTCATTCATTCATTGTTCAAG  30446
Domestic       TATGCTTCTGGCACTAGTTCTTTGTCATTCATTCCAGTCCTCATTCATTCATTGTTCAAG  30868
               ************************************************************

Dromedary      CACTTATTTGTGACACTTTCTCTGTGCCAGGAACATTCTAGTCCTCATTCATTCATTCCT  30714
Wild           CACTTATTTGTGACACTTTCTCTGTGCCAGGAACATTCTAGTCCTCATTCATTCATTCCT  30506
Domestic       CACTTATTTGTGACACTTTCTCTGTGCCAGGAACATTCTAGTCCTCATTCATTCATTCCT  30928
               ************************************************************

Dromedary      TGTTCAAGCACTTATTTCTGGCACTTTCTCTGTCCGAGGAAATCTGCCAGATGCTGGAAG  30774
Wild           TGTTCAAACACTTATTTCTGGCACTTTCTCTGTGCGAGGAAATCTGCCAGATGCTGGAAG  30566
Domestic       TGTTCAAGCACTTATTTCTGGCACTTTCTCTGTGCGAGGAAATCTGCCAGATGCTGGAAG  30988
               ******* ************************* **************************

Dromedary      AAGCGATGAGTAAGAGAAGAGTCCCATCGTCAAGGAGTTCTATTCAGTGGAATAGCCAGG  30834
Wild           AAGCGATGAGTAAGAGAAGAGTCCTATCGTCAAGGAGTTCTATTCAGTGGAATAGCCAGG  30626
Domestic       AAGCGATGAGTAAGAGAAGAGTCCCATCGTCAAGGAGTTCTATTCAGTGGAATAGCCAGG  31048
               ************************ ***********************************

Dromedary      TTCACAAACAGAAAACCCCAGTACAGTACGACAATGCTGGGGCAGAGGTATGTCAGGATT  30894
Wild           TTCACAAACAGAAAACCCCAGTACAGTACGACAATGCTGGGGCAGAGGTATGCCAGGATT  30686
Domestic       TTCACAAACAGAAAACCCCAGTACAGTACGACAATGCTGGGGCAGAGGTATGCCAGGATT  31108
               **************************************************** *******

Dromedary      TTAGAGGCAGGAACAGGATGAGAACATTACTAGGAAGCCCCAAGTGTCCAGCTGAGAATG  30954
Wild           TTAGAGGCAGGAACAGGATGAGAACATTACTAGGAAGCCCCAAGTGTCCAGCTGAGAATG  30746
Domestic       TTAGAGGCAGGAACAGGATGAGAACATTACTAGGAAGCCCCAAGTGTCCAGCTGAGAATG  31168
               ************************************************************

Dromedary      GAGACAGTGAGTTTGCAGCAGCATCAACTCACTCTGCTGCTAGAGAAAAATCACAAAATA  31014
Wild           GAGACAGTGAGTTTGCAGCAGCATCAACTCACTCTGCTGCTAGAGAAAAATCACAAAATA  30806
Domestic       GAGACAGTGAGTTTGCAGCAGCATCAACTCACTCTGCTGCTAGAGAAAAATCACAAAATA  31228
               ************************************************************

Dromedary      TATCAATCCACATAAAGAAGCAAAGAAACCAAGTGGATTTGCTAGAGATCTACAGATTTG  31074
Wild           TATCAATCCGCATAAAGAAGCAAAGAAACCAAGTGGATTTGCTAGAGATCTACAGATTTG  30866
Domestic       TATCAATCCGCATAAAGAAGCAAAGAAACCAAGTGGATTTGCTAGAGATCTACAGATTTG  31288
               ********* **************************************************
```

```
Dromedary      CAAGGGCGGTAGGGCAGGAAAAAGAGGGATCCAGGGTTACGGGGTACTTTTCATGAAAGA  31134
Wild           CAAGGGTGGTAGGGCAGGAAAAAGAGGGATCCAGGGTTACGGGGTACTTTTCATGAAAGA  30926
Domestic       CAAGGGTGGTAGGGCAGGAAAAAGAGGGATCCAGGGTTACGGGGTACTTTTCATGAAAGA  31348
               ****** *****************************************************

Dromedary      GCTCAGTCTAAAGTAGCTACGCAAGAAAGAAAGCTAAAAGAGGTCAGGAGCTTGTAGGTT  31194
Wild           GCTCAGTCTAAAGTAGCTACGCAAGAAAGAAAGCTAAAAGAAGTCAGGAGCTTGTAGGTT  30986
Domestic       GCTCAGTCTAAAGTAGCTACGCAAGAAAGAAAGCTAAAAGAGGTCAGGAGCTTGTAGGTT  31408
               ***************************************** ******************

Dromedary      AGAAGAAAAGAAGGATCAAATCTCAATGGGATTTAAAGGGCAAGAATTCAGAGACAGTGA  31254
Wild           AGAAGAAAAGAAGGATCAAATCTCAATGGGATTTAAAGGGCAAGAATTCAGAGACAGTGA  31046
Domestic       AGAAGAAAAGAAGGATCAAATCTCAATGGGATTTAAAGGGCAAGAATTCAGAGACAGTGA  31468
               ************************************************************

Dromedary      ATTAGGCATTTTGGTTCAATAACTCAATCAGCCAATAATTAGGATATTCATATAACTTAT  31314
Wild           ATTAGGCATTTTGGTTCAATAACTCAATCAGCCAATAATTAGGATATTCATATAACTTAT  31106
Domestic       ATTAGGCATTTTGGTTCAATAACTCAATCAGCCAATAATTAGGATATTCATATAACTTAT  31528
               ************************************************************

Dromedary      TACTCAAATAGTGAAGGGAAACAAATGATTATTAATCATGACTCCAGGCGGCAGATGTAA  31374
Wild           TACTCAAATAGTGAAGGGAAACAAATGATTATTAATCATGACTCCAGGCGGCAGATGTAA  31166
Domestic       TACTCAAATAGTGAAGGGAAACAAATGATTATTAATCATGACTCCAGGCGGCAGATGTAA  31588
               ************************************************************

Dromedary      ACTGGGACAATCCTAGGATGCCTAGCTGCCCTGTCACTAATCCATGTGCTAGATACTGTG  31434
Wild           ACTGGGACAATCCTAGGATGCCTAGCTGCCCTGTCACTAATCCATGTGCTAGATACTGTG  31226
Domestic       ACTGGGACAATCCTAGGATGCCTAGCTGCCCTGTCACTAATCCATGTGCTAGATACTGTG  31648
               ************************************************************

Dromedary      CTAGGTACTGGGGATAAGGTGAACAACAAAGCAGAAATGGTGCCTGTCACCATGGAGTTC  31494
Wild           CTAGGTACTGGGGATAAGGTGAACAACAAGGCAGAAATGGTGCCTGTCACCATGGAGTTC  31286
Domestic       CTAGGTACTGGGGATAAGGTGAACAACAAGGCAGAAATGGTGCCTGTCACCATGGAGTTC  31708
               ***************************** ******************************

Dromedary      ATAGTCATACAGGAATGACAAACACTGAACAAGAAATTATTACTGTGATGAATGGTGCTA  31554
Wild           ATAGTCATACAGGAATGACAAACACTGAACAAGAAATTATTACTGTGATGAATGGTGCTA  31346
Domestic       ATAGTCATACAGGAATGACAAACACTGAACAAGAAATTATTACTGTGATGAATGGTGCTA  31768
               ************************************************************

Dromedary      ACAAGAGAGATGAAGGAGCCAGGAGAGTTTATTTTGATTTTAAAAATGTTTTTTTCTATT  31614
Wild           ACAAGAGAGATGAAGGAGCCAGGAGAGTTTATTTTGATTTTAAAAATGTTTTTTTCTATT  31406
Domestic       ACAAGAGAGATGAAGGAGCCAGGAGAGTTTATTTTGATTTTAAAAATGTTTTTTTCTATT  31828
               ************************************************************

Dromedary      GAAGTATAATCAGTTTACAAAGTAGTGTCAATTTCTGGTATACAGAATAAAGTTTCAGTC  31674
Wild           GAAGTATAATCAGTTTACAAAGTAGTGTCAATTTCTGGTATACAGAATAAAGTTTCAGTC  31466
Domestic       GAAGTATAATCAGTTTACAAAGTAGTGTCAATTTCTGGTATACAGAATAAAGTTTCAGTC  31888
               ************************************************************

Dromedary      ATATGTACATACATATATTCCTTTTCCTGTTCTTTTTCATTATAGGTTACTACAATATAT  31734
Wild           ATATGTACATACATATATTCCTTTTCCTGTTCTTTTTCATTATAGGTTACTACAATATAT  31526
Domestic       ATATGTACATACATATATTCCTTTTCCTGTTCTTTTTCATTATAGGTTACTACAATATAT  31948
               ************************************************************

Dromedary      TGAATGTAGATCCCTGTGCTATACGGTATAAATTTGTTTATCTATTTTATACATAGTAGT  31794
Wild           TGAATGTAGATCCCTGTGCTATACGGTATAAATTTGTTTATCTATTTTATACATAGTAGT  31586
Domestic       TGAATGTAGATCCCTGTGCTATACGGTATAAATTTGTTTATCTATTTTATACATAGTAGT  32008
               ************************************************************

Dromedary      TAGTATCTGCAAATCTCAATCTCCCAATTTATCCCTTCCCACCCTCTTCCCCCCAGTAAC  31854
Wild           TAGTATCTGCAAATCTCAATCTCCCAATTTATCCCTTCCCACCCTCTTCCCCCCAGTAAC  31646
Domestic       TAGTATCTGCAAATCTCAATCTCCCAATTTATCCCTTCCCACCCTCTTCCCCCCAGTAAC  32068
               ************************************************************

Dromedary      CATAAGTTTGTTTTCTATGTCTGTGAGTCTGTTTCTGTTTTGTAAATACGTTCATTTGTG  31914
Wild           CATAAGTTTGTTTTCTATGTCTGTGAGTCTGTTTCTGTTTTGTAAATACGTTCATTTGTG  31706
Domestic       CATAAGTTTGTTTTCTATGTCTGTGAGTCTGTTTCTGTTTTGTAAATACGTTCATTTGTG  32128
               ************************************************************
```

```
Dromedary      TCTTTTTTTTAAGATTCCACATATTAGTGATATCATATGGAATTTTTCTTTCTCTTTCTG  31974
Wild           TCTTTTTTTTAAGATTCCACATATTAGTGATATCATATGGAATTTTTCTTTCTCTTTCTG  31766
Domestic       TCTTTTTTTTAAGATTCCACATATTAGTGATATCATATGGAATTTTTCTTTCTCTTTCTG  32188
               ************************************************************

Dromedary      GTTTATTTCACTTAGAATGATGATCTCCAGGTCCATCCATGTTGCAGGAAATGGCATTAT  32034
Wild           GTTTATTTCACTTAGAATGATGATCTCCAGGTCCATCCATGTTGCAGGAAATGGCATTAT  31826
Domestic       GTTTATTTCACTTAGAATGATGATCTCCAGGTCCATCCATGTTGCAGGAAATGGCATTAT  32248
               ************************************************************

Dromedary      TTTATTTCTTATGGCTGAGTAGTATTTCATTGTGTGTATATGTATATTTCTGTGTATACC  32094
Wild           TTTATTTCTTATGGCTGAGTAGTATTTCATTGTGTGTATATGTATATTTCTGTGTATACC  31886
Domestic       TTTATTTCTTATGGCTGAGTAGTATTTCATTGTGTGTATATGTATATTTCTGTGTATACC  32308
               ************************************************************

Dromedary      ACAACTTCTTTATTTAGTCATCTGTCAATGGACATTTAGGTTGTTTACATATCATGGCTA  32154
Wild           ACAACTTCTTTATTTAGTCATCTGTCAATGGACATTTAGGTTGTTTACATATCATGGCTA  31946
Domestic       ACAACTTCTTTATTTAGTCATCTGTCAATGGACATTTAGGTTGTTTACATATCATGGCTA  32368
               ************************************************************

Dromedary      TTGTAAATAGTGCTGCTGTGAACATTGGGGTGCATGTAGCTTTTCGAATTGGAGTTCCTT  32214
Wild           TTGTAAATAGTGCTGCTGTGAACATTGGGGTGCATGTAGCTTTTCGAATTAGAGTTCCTT  32006
Domestic       TTGTAAATAGTGCTGCTGTGAACATTGGGGTGCATGTAGCTTTTCGAATTAGAGTTCCTT  32428
               ************************************************** *********

Dromedary      CTGGATATATGCCCAGGAGTGGGATTGTTGGATCATATTGTGAGTCTATTTTTACTTTTT  32274
Wild           CTGGATATATGCCCAGGAGTGGGATTGTTGGATCATATTGTAAGTCTATTTTTACTTTTT  32066
Domestic       CTGGATATATGCCCAGGAGTGGGATTGTTGGATCATATTGCAAGTCTATTTTTACTTTTT  32488
               ****************************************  ******************

Dromedary      TAAGGAGTCTCCATACTGTTTTCCATAGTGGCTACACCAAACTACATTTCCACCAACAGC  32334
Wild           TAAGGAGTCTCCATACTGTTTTCCATAGTGGCTACACCAAACTACATTTCCACCAACAGC  32126
Domestic       TAAGGAGTCTCCATACTGTTTTCCATAGTGGCTACACCAAACTACATTTCCACCAACAGC  32548
               ************************************************************

Dromedary      ATAGAAGGGTTCCCTTTTCTCCACAGCTAAGAGAGTTTATTACAAAGGAAACCAACATCT  32394
Wild           ATAGAAGGGTTCCCTTTTCTCCACAGCTAAGAGAGTTTATTACAAAGGAAACCAACATCT  32186
Domestic       ATAGAAGGGTTCCCTTTTCTCCACAGCTAAGAGAGTTTATTACAAAGGAAACCAACATCT  32608
               ************************************************************

Dromedary      TTTCAGAGGTCAGCAAAGGGTCCCCTTAGGAGATGACATTTAAGCTGAGACTGCAAAAGT  32454
Wild           TTTCAGAGGTCAGCAAAGGGTCCCCTTAGGAGATGACATTTAAGCTGAGACTGCAAAAGT  32246
Domestic       TTTCAGAGGTCAGCAAAGGGTCCCCTTAGGAGATGACATTTAAGCTGAGACTGCAAAAGT  32668
               ************************************************************

Dromedary      GAGTAGGAGCTTGAAGGTAGAAAGGGGAGGTATGAGTGCCCAGGCAGAGGGAAGAGTGTT  32514
Wild           GAGTAGGAGCTTGAAGGTAGAGAGGGGAGGTATGAGTGCCCAGGCAGAGGGAAGAGTGTT  32306
Domestic       GAGTAGGAGCTCGAAGGTAGAGAGGGGAGGTATGAGTGCCCAGGCAGAGGGAAGAGTGTT  32728
               *********** ********* **************************************

Dromedary      TACTCAGGCCTTGGGATGGGAAAGGATGTTACATTTGATTTCTAAATTCTAACAGATTTT  32574
Wild           TACTCAGGCCTTGGGATGGGAAAGGATGTTACATTTGATTTCTAAATTCTAACAGATTTT  32366
Domestic       TACTCAGGCCTTGGGATGGGAAAGGATGTTACATTTGATTTCTAAATTCTAACAGATTTT  32788
               ************************************************************

Dromedary      TAAAAATCCTTATGGGTCTTTTTATCTTGCTGTTGTTTTCTTTGCTTATGGTTTAAATTC  32634
Wild           TAAAAATCCTTATGGGTCTTTTTATCTTGCTGTTGTTTTCTTTGCTTATGGTTTAAATTC  32426
Domestic       TAAAAATCCTTATGGGTCTTTTTATCTTGCTGTTGTTTTCTTTGCTTATGGTTTAAATTC  32848
               ************************************************************

Dromedary      CTTCCTTCATCGCTTTGAACATTCTAAACACACTTTGTAAACATTTCATTCAGGTCGTTC  32694
Wild           CTTCCTTCATCGCTTTGAACATTCTAAACACACTTTGTAAACATTTCATTCAGGTCGTTC  32486
Domestic       CTTCCTTCATCGCTTTGAACATTCTAAACACACTTTGTAAACATTTCATTCAGGTCGTTC  32908
               ************************************************************

Dromedary      TACCCTTTCAAGGTTTGGCTCCTATTTCTTTCATCTGCTGACTCTTCGTGGACAGTTGTT  32754
Wild           TACCCTTTCAAGGTTTGGCTCCTATTTCTTTCATCTGCTGACTCTTCGTGGACAGTTGTT  32546
Domestic       TACCCTTTCAAGGTTTGGCTCCTATTTCTTTCATCTGCTGACTCTTCGTGGACAGTTGTT  32968
               ************************************************************
```

```
Dromedary      TGTTTTATGGTATGATATATAACTGGTGACTGTGAGTTAGTCATGAGAAGAGATTCTCCG 32814
Wild           TGTTTTATGGTATGATATATAACTGGTGACTGTGAGTTAGTCATGAGAAGAGATTCTCCG 32606
Domestic       TGTTTTATGGTATGATATATAACTGGTGACTGTGAGTTAGTCATGAGAAGAGATTCTCCG 33028
               ************************************************************

Dromedary      TGGGGACATAGTCTACAGGGTGCTATTCTTTTATCGCAACTTGAGCGAAAGAATCAGCTT 32874
Wild           TGGGGACATAGTCTACAGGGTGCTATTCTTTTATCGCAACTTGAGCGAAAGAATCAGCTT 32666
Domestic       TGGGGACATAGTCTACAGGGTGCTATTCTTTTATCGCAACTTGAGCGAAAGAATCAGCTT 33088
               ************************************************************

Dromedary      TCTTTTCAGGTTGATTTCCCAGCTTCAGGTCCCACCATACAGGCCATGTGATTTTAGACT 32934
Wild           TCTTTTCAGGTTGATTTCCCAGCTTCAGGTCCCACCATACAGGCCATGTGATTTTAGACT 32726
Domestic       TCTTTTCAGGTTGATTTCCCAGCTTCAGGTCCCACCATACAGGCCATGTGATTTTAGACT 33148
               ************************************************************

Dromedary      CTGTGTGTAGTGCAGACCTGGGGTTTTGATTTCCTGCAGGTGAAAACTTCTCAGTTGTGA 32994
Wild           CTGTGTGTAGTGCAGACCTGGGGTTTTGATTTCCTGCAGGTGAAAACTTCTCAGTTGTGA 32786
Domestic       CTGTGTGTAGTGCAGACCTGGGGTTTTGATTTCCTGCAGGTGAAAACTTCTCAGTTGTGA 33208
               ************************************************************

Dromedary      TCTTGGGATACTATAAACCTTCTTGTTTATCTGGGCTGATGGGCAGAGTACCCTTAGTCT 33054
Wild           TCTTGGGATACTATAAACCTTCTTGTTTATCTGGGCTGATGGGCAGAGTACCCTTAGTCT 32846
Domestic       TCTTGGGATACTATAAACCTTCTTGTTTATCTGGGCTGATGGGCAGAGTACCCTTAGTCT 33268
               ************************************************************

Dromedary      TCATTTCAAAGTCAGCACAACCCTCTGTGACTTCCAGATTTATCTAAATAGCTCAGTTCA 33114
Wild           TCATTTCAAAGTCAGCACAACCCTCTGTGACTTCCAGATTTATCTAAATAGCTCAGTTCA 32906
Domestic       TCATTTCAAAGTCAGCACAACCCTCTGTGACTTCCAGATTTATCTAAATAGCTCAGTTCA 33328
               ************************************************************

Dromedary      AGCTTACCTCCCAGGGCCTGGAGCTTAGACCCGCCTTCCAGGGCAGTTAGTTGTTAGAAC 33174
Wild           AGCTTACCTCCCAGGGCCTGGAGCTTAGACCCGCCTTCCAGGGCAGTTAGTTGTTAGAAC 32966
Domestic       AGCTTACCTCCCAGGGCCTGGAGCTTAGACCCGCCTTCCAGGGCAGTTAGTTGTTAGAAC 33388
               ************************************************************

Dromedary      CAATTCCAGCTTCTAAGGCCCATATCTGGTTGCAATAATTCTATGAGCTGTTTGGGCATA 33234
Wild           CAATTCCAGCTTCTAAGGCCCATATCTGGTTGCAATAATTCTATGAGCTGTTTGGGCATA 33026
Domestic       CAATTCCAGCTTCTAAGGCCCATATCTGGTTGCAATAATTCTATGAGCTGTTTGGGCATA 33448
               ************************************************************

Dromedary      AGAATCTGCTTAACACACTGACTTGAAGCTTCCTCTTTAATTCTGACACTTGGGCGACAC 33294
Wild           AGAATCTGCTTAACACACTGACTTGAAGCTTCCTCTTTAATTCTGACACTTGGGCGACAC 33086
Domestic       AGAATCTGCTTAACACACTGACTTGAAGCTTCCTCTTTAATTCTGACACTTGGGCGACAC 33508
               ************************************************************

Dromedary      TCTTTCTTGGCTTCAAGCTTAGCTACATACTTTAAGAAGTTTTAGCCATATTTTATCCTG 33354
Wild           TCTTTCTTGGCTTCAAGCTTAGCTACATACTTTAAGAAGTTTTAGCCATATTTTATCCTG 33146
Domestic       TCTTTCTTGGCTTCAAGCTTAGCTACATACTTTAAGAAGTTTTAGCCATATTTTATCCTG 33568
               ************************************************************

Dromedary      TATTTAGAGCAGAAGAGTGAAGAATTCTTTGGCATCAGCTCATGATATAATACAGTAAAT 33414
Wild           TATTTAGAGCAGAAGAGTGAAGAATTCTTTGGCATCAGCTCATGATATAATACAGTAAAT 33206
Domestic       TATTTAGAGCAGAAGAGTGAAGAATTCTTTGGCATCAGCTCATGATATAATACAGTAAAT 33628
               ************************************************************

Dromedary      TTTAGAAACTTTTTAAAACGTATTCATTCATCAAAAAAATTGTGGAGCACCTATGGTGTT 33474
Wild           TTTAGAAACTTTTTAAAACGTATTCATTCATCAAAAAAATTGTGGAGCACCTATGGTGTT 33266
Domestic       TTTAGAAACTTTTTAAAACGTATTCATTCATCAAAAAAATTGTGGAGCACCTATGGTGTT 33688
               ************************************************************

Dromedary      CTAAGTATTGTAGGACTTAAGAACAGAAAATGAGAAAAATAAAGTTAAAGAATGAGACGG 33534
Wild           CTAAGTATTGTAGGACTTAAGAACAGAAAATGAGAAAGATAAAGTTAAAGAATGAGACGG 33326
Domestic       CTAAGTATTGTAGGACTTAAGAACAGAAAATGAGAAAGATAAAGTTAAAGAATGAGACGG 33748
               ************************************* **********************

Dromedary      ATTTGTGTAAATGACGTTAAGAATCTGGATTTTTCTTCAGAGCAAATGGGCAGCTAGTGA 33594
Wild           ATTTGTGTAAATGACGTTAAGAATCTGGATTTTTCTTCAGAGCAAATGGGCAGCTAGTGA 33386
Domestic       ATTTGTGTAAATGACGTTAAGAATCTGGATTTTTCTTCAGAGCAAATGGGCAGCTAGTGA 33808
               ************************************************************
```

```
Dromedary      ACGATTTTAAAATAAGTTTGCATCTTAAAAATGCAGAATGGAGAATGGATTGGAGCCCAA  33654
Wild           ACGATTTTAAAATAAGTTTGCATCTTAAAAATGCAGAATGGAGAATGGATTGGAGCCCAA  33446
Domestic       ACGATTTTAAAATAAGTTTGCATCTTAAAAATGCAGAATGGAGAATGGATTGGAGCCCAA  33868
               ************************************************************

Dromedary      AAAGAACAGATGTGGGAGGAAGTGTTCAACAGTCCAGTGATTGATGATGGTGGCTTGAAC  33714
Wild           AAAGAACAGATGTGGGAGGAAGTGTTCAACAGTCCAGTGATTGATGATGGTGGCTTGAAC  33506
Domestic       AAAGAACAGATGTGGGAGGAAGTGTTCAACAGTCCAGTGATTGATGATGGTGGCTTGAAC  33928
               ************************************************************

Dromedary      TGGACAGTGAAGACAGATCAAAGTGACTGGACTTAAGAGATATTCAGGTAGTAGTAATAA  33774
Wild           TGGACAGTGAAGACAGATCAAAGTGACTGGACTTAAGAGATATTCAGGTAGTAGTAATAA  33566
Domestic       TGGACAGTGAAGACAGATCAAAGTGACTGGACTTAAGAGATATTCAGGTAGTAGTAATAA  33988
               ************************************************************

Dromedary      CTTACTTATGGGCTTAACTGGGGAAGGGATGGAGATGAGTAAAATATCACTCCCAGGTGC  33834
Wild           CTTACTTATGGGCTTAACTGGGGAAGGGATGGAGATGAGTAAAATTTCACTCCCAGGTGC  33626
Domestic       CTTACTTATGGGCTTAACTGGGGAAGGGATGGAGATGAGTAAAATTTCACTCCCAGGTGC  34048
               ********************************************* **************

Dromedary      TTGGCTTGAGTATCTGAGGGGTTGTTAATGGCACTTAATGAGCTAGAGAGTACAAGGAGA  33894
Wild           TTGGCTTGAGTATCTGAGGGGTTGTTAATGGCACTTAATGAGCTAGAGAGTACAAGGAGA  33686
Domestic       TTGGCTTGAGTATCTGAGGGGTTGTTAATGGCACTTAATGAGCTAGAGAGTACAAGGAGA  34108
               ************************************************************

Dromedary      AGGAGCAGATTTAATAGGAAACATCTCAAGTTCAGTTTTGGGCATGTTGAGTGTAAGGCA  33954
Wild           AGGAGCAGATTTAATAGGAAACATCTCAAGTTCAGTTTTGGGCATGTTGAGTGTAAGGCA  33746
Domestic       AGGAGCAGATTTAATAGGAAACATCTCAAGTTCAGTTTTGGGCATGTTGAGTGTAAGGCA  34168
               ************************************************************

Dromedary      CCAGACAGCCCAGTGGAGATATCAAGTAAGCAGTAAGCTAATTGTGTTGGAGACTCAGAG  34014
Wild           CCAGACAGCCCAGTGGAGATATCAAGTAAGCAGTAAGCTAATTGTGTTGGAGACTCAGAG  33806
Domestic       CCAGACAGCCCAGTGGAGATATCAAGTAAGCAGTAAGCTAATTGTGTTGGAGACTCAGAG  34228
               ************************************************************

Dromedary      CATAAGTCTGAGCTGGAAATAAAAAGAAAATTGAGTTTTTTGGAGTAGATGAGATTTCCT  34074
Wild           CATAAGTCTGAGCTGGAAATAAAAAGAAAATTGAGTTTTTTGGAGTAGATGAGATTTCCT  33866
Domestic       CATAAGTCTGAGCTGGAAATAAAAAGAAAATTGAGTTTTTTGGAGTAGATGAGATTTCCT  34288
               ************************************************************

Dromedary      GAAGAGAGGGTAGAGTGATCCTAAAATTCATACAGAACCACAAAAGACCCTCAATTGCCA  34134
Wild           GAAGAGAGGGTAGAGTGATCCTAAAATTCATACAGAACCACAAAAGACCCTCAATTGCCA  33926
Domestic       GAAGAGAGGGTAGAGTGATCCTAAAATTCATATAGAACCACAAAAGACCCTCAATTGCCA  34348
               ******************************** ***************************

Dromedary      AAGCAATCTTGAGAAAAAAAGAACAAAGCTGAAATTATCATCTTCCCAGACATAAGACTA  34194
Wild           AAGCAATCTTGAGAAAAAAAGAACAAAGCTGAAGTTATCATCTTCCCAGACATAAGACTA  33986
Domestic       AAGCAATCTTGAGAAAAAAAGAACAAAGCTGAAGTTATCATCTTCCCAGACATAAGACTA  34408
               ********************************* **************************

Dromedary      TATTACAAAGGTACAATAATCAAAATAGCATGGTACTGGCACAAAAACAGATATATAGTA  34254
Wild           TATTACAAAGGTACAATAATCAAAATAGCATGGTACTGGCACAAAAACAGATATATAGTA  34046
Domestic       TATTACAAAGGTACAATAATCAAAATAGCATGGTACTGGCACAAAAACAGATATATAGTA  34468
               ************************************************************

Dromedary      GCTACCAGAGGGGAGAGGGAAGGAGAAGGGACAAGTTATGGGTATGAGATTAAGAGGTTA  34314
Wild           GCTACCAGAGGGGAGAGGGAAGGAGAAGGGACAAGTTATGGGTATGAGATTAAGAGGTTA  34106
Domestic       GCTACCAGAGGGGAGAGGGAAGGAGAAGGGACAAGTTATGGGTATGAGATTAAGAGGTTA  34528
               ************************************************************

Dromedary      AAAACTACTATGTATAAAATAGATGAGCAACAAGGATATATTGTATAGCACAGGGAATTA  34374
Wild           AAAACTACTATGTATAAAATAGATGAGCAACAAGGATATATTGTATAGCACAGGGAATTA  34166
Domestic       AAAACTACTATGTATAAAATAGATGAGCAACAAGGATATATTGTATAGCACAGGGAATTA  34588
               ************************************************************

Dromedary      TAGCTGTTATCTTGTAATAACTTTCAATGGAGTATAATCTATATAAACACTGAATCACTA  34434
Wild           TAGCTGTTATCTTGTAATAACTTTCAATGGAGTATAATCTATATAAACACTGAATCACTA  34226
Domestic       TAGCTGTTATCTTGTAATAACTTTCAATGGAGTATAATCTATATAAACACTGAATCACTA  34648
               ************************************************************
```

```
Dromedary      CACTGTACACCTGAAACTAATATAATATTGTAAATCAAATGTACTTAAATAAAAACCAAA  34494
Wild           CGCTGTACACCTGAAACTAATATAATATTGTAAATCAAATGTACTTAAATAAAAACCAAA  34286
Domestic       AGCTGTACACCTGAAACTAATATAATATTGTAAATCAAATGTACTTAAATAAAAACCAAA  34708
                 **********************************************************

Dromedary      CAACCCCCCCGCCCCCAAAAAAGAGATAGAGGGTAGACCAGAAACAGAAGAGGGTTTGTA  34554
Wild           CAACACCCCCGCCCCCAAAAAAGAGATAGAGGGTAGACCAGAAACAGAAGAGGGTTTGTA  34346
Domestic       CAACACCCCCGCCCCCAAAAAAGAGATAGAGGGTAGACCAGAAACAGAAGAGGGTTTGTA  34768
               **** *******************************************************

Dromedary      AGTCCCCTTGAGGAATTCCATCACTTAATGGCTGGGTAGAGGTAAAGAGCCAAGAAAAGA  34614
Wild           AGTCCCCTTGAGGAATTCCATCACTTAATGGCTGGGTAGAGGTAAAGAGCCAAGAAAAGA  34406
Domestic       AGTCCCCTTGAGGAATTCCATCACTTAATGGCTGGGTAGAGGTAAAGAGCCAAGAAAAGA  34828
               ************************************************************

Dromedary      ACAGCTGGAAGGAAGCGTTATATCAAGGGTGACATTCTTCCCCTTAAGAAGGAATGGCTT  34674
Wild           ACAGCTGGAAGGAAGCGTTATATCAAGGGTGACATTCTTCCCCTTAAGAAGGAATGGCTT  34466
Domestic       ACAGCTGGAAGGAAGCGTTATATCAAGGGTGACATTCTTCCCCTTAAGAAGGAATGGCTT  34888
               ************************************************************

Dromedary      GACAATATTTGCTGATAGGAAGAACACCAAAGAGAAAGACTATAATTCCATGGAGAGAAA  34734
Wild           GACAATATTTGCTGATAGGAAGAACACCAAAGAGAAAGACTATAATTCCATGGAGAGAAA  34526
Domestic       GACAATATTTGCTGATAGGAAGAACACCAAAGAGAAAGACTATAATTCCATGGAGAGAAA  34948
               ************************************************************

Dromedary      GTACAACTGCTAACATAAAGTACCTGAGAAGCTGGAAGAGGATAGGGATTCAGGGCAGAG  34794
Wild           GTACAACTGCTAACATAAAGTACCTGAGAAGCTGGAAGAGGATAGGGATTCAGGGCAGAG  34586
Domestic       GTACAACTGCTAACATAAAGTACCTGAGAAGCTGGAAGAGGATAGGGATTCAGGGCAGAG  35008
               ************************************************************

Dromedary      GTAGAAGGATTGGTCTTTGACAGGAGGCAACATTGTAACAAGAGGGAAGGAAGACAGGAT  34854
Wild           GTAGAAGGATTGGTCTTTGACAGGAGGCAACATTGTAACAAGAGGGAAGGAAAACAGGAT  34646
Domestic       GTAGAAGGACTGGTCTTTGACAGGAGGCAACATTGTAACAAGAGGGAAGGAAAACAGGAT  35068
               ********* ****************************************** *******

Dromedary      GGGTACAGATGCAGATAGGTTTGTAAAGCTGATGTTTGAAAGGCTGTGGTAGTTCTCTGA  34914
Wild           GGGTACAGATGCAGATAGGTTTGTAAAGCTGATGTTTGAAAGGCTGTGGTAGTTCTCTGA  34706
Domestic       GGGTACAGATGCAGATAGGTTTGTAAAGCTGATGTTTGAAAGGCTGTGGTAGTTCTCTGA  35128
               ************************************************************

Dromedary      CAGTGTGTTACTTTCTCTGTAAAGCAGATGAGCTCATGTGCTCCATGTACTAGGGTAGAC  34974
Wild           CAGTGTGTTACTTTCTCTGTAAAGCAGATGAGCTCATGTGCTCCATGTACTAGGGTAGAC  34766
Domestic       CAGTGTGTTACTTTCTCTGTAAAGCAGATGAGCTCATGTGCTCCATGTACTAGGGTAGAC  35188
               ************************************************************

Dromedary      AGAAGGACAGAGGCTTGAGAAGACTTTAGGATTGAACTGACTATGAAGACAATGGCTTTC  35034
Wild           AGAAGGACAGAGGCTTGAGAAGACTTTAGGATTGAACTGACTATGAAGACAATGGCTTTC  34826
Domestic       AGAAGGACAGAGGCTTGAGAAGACTTTAGGATTGAACTGACTATGAAGACAATGGCTTTC  35248
               ************************************************************

Dromedary      CAGGCAATGCTGATGACACAGAAATCTGCCAGTTCATATGATTTTTCCCTATTAGTGCTC  35094
Wild           CAGGCAATGCTGATGACACAGAAATCTGCCAGTTCATATGACTTTTCCCTATTAGTGCTC  34886
Domestic       CAGGCAATGCTGATGACACAGAAATCTGCCAGTTCATATGATTTTTCCCTATTAGTGCTC  35308
               ***************************************** ******************

Dromedary      TTCAGTCTGAGTACAAACAGAAAAGGTGGGTGGGTAACTAGATATATCTAGCTTGGGCTT  35154
Wild           TTCAGTCTGAGTACAAACAGAAAAGGTGGGTGGGTAACTAGATATATCTAGCTTGGGCTT  34946
Domestic       TTCAGTCTGAGTACAAACAGAAAAGGTGGGTGGGTAACTAGATATATCTAGCTTGGGCTT  35368
               ************************************************************

Dromedary      TGTCAGCTTGGGGCCTTGACAGACAGGTGCTACAAAAAGACAATGAGGCAAAGGTAAGTT  35214
Wild           TGTCAGCTTGGGGCCTTGACAGACAGGTGCTACAAAAAGACAATGAGGCAAAGGTAAGTT  35006
Domestic       TGTCAGCTTGGGGCCTTGACAGACAGGTGCTACAAAAAGACAATGAGGCAAAGGTAAGTT  35428
               ************************************************************

Dromedary      AGAATATTGACAGGAAAGTGGCTAAGATGATGGCTTATGGACTATGCATTGGATAAAGGA  35274
Wild           AGAATATTGACAGGAAAGTGGCTAAGATGATGGCTTATGGACTATGCATTGGATAAAGGA  35066
Domestic       AGAATATTGACAGGAAAGTGGCTAAGATGATGGCTTATGGACTATGCATTGGATAAAGGA  35488
               ************************************************************
```

```
Dromedary      GGAAACTAAATACAGAAAGGAGATGATGAGAGGAAGACAGCAGAATGGCACAAACTGAAT 35334
Wild           GGAAACTAAATACAGAAAGGAGATGATGAGAGGAAGACAGCAGAATGGCACAAACTGAAT 35126
Domestic       GGAAACTAAATACAGAAAGGAGATGATGAGAGGAAGACAGCAGAATGGCACAAACTGAAT 35548
               ************************************************************

Dromedary      GTTTGTACCCTGCCAAAATTCCTATGTTGAAATCCTAACCCCCAAGGA-GATGGTATTAG 35393
Wild           GTTTGTACCCTGCCAAAATTCCTATGTTGAAATCCTAACCCCCAAGGACGATGGTATTAG 35186
Domestic       GTTTGTACCCTGCCAAAATTCCTATGTTGAAATCCTAACCCCCAAGGACGATGGTATTAG 35608
               ************************************************ ***********

Dromedary      GAGGCGGGGCTTTTGGGAAGTGGAACCTTCATGGAGGGAATTACTGCCCTTATAAAAGAG 35453
Wild           GAGGCGGGGCTTTTGGGAAGTGGAACCTTCATGGAGGGAATTACTGCCCTTATAAAAGAG 35246
Domestic       GAGGCGGGGCTTTTGGGAAGTGGAACCTTCATGGAGGGAATTACTGCCCTTATAAAAGAG 35668
               ************************************************************

Dromedary      ATTCCATAGAGTGCCCTAACCCCCTTCCACCTTATGAGTGTACAGAGAGGAGGTGCTGGC 35513
Wild           ATTCCATAGAGTGCCCTAACCCCCTTCCACCTTATGAGTGTACAGAGAGGAGGTGCTGGC 35306
Domestic       ATTCCATAGAGTGCCCTAACCCCCTTCCACCTTATGAGTGTACAGAGAGGAGGTGCTGGC 35728
               ************************************************************

Dromedary      TATGAACCAGAAGAAGGCTCTGACCCAATCACCTGGCACCCTGATCGCCAGCAGCCAGGA 35573
Wild           TATGAACCAGAAGAAGGCTCTGACCCAATCACCTGGCACCCTGATCGCCAGCAGCCAGGA 35366
Domestic       TATGAACCAGAAGAAGGCTCTGACCCAATCACCTGGCACCCTGATCGCCAGCAGCCAGGA 35788
               ************************************************************

Dromedary      CTATGGGAAATAATTTCTGCTGTTTATAAGCTACCCAGTCTGTGATATTTCGTTATAGTA 35633
Wild           CTATGGGAAATAATTTCTGCTGTTTATAAGCTACCCAGTCTGTGATATTTCGTTATAGTA 35426
Domestic       CTATGGGAAATAATTTCTGCTGTTTATAAGCTACCCAGTCTGTGATATTTCGTTATAGTA 35848
               ************************************************************

Dromedary      ACCCTAGCAGACTAATACAAACGATCAATGAACTGAAAGTTGCAAGTGAAGTAGTGGTAG 35693
Wild           ACCCTAGCAGACTAATACAAACGATCAATGAACTGAAAGTTGCAAGTGAAGTAGTGGTAG 35486
Domestic       ACCCTAGCAGACTAATACAAACGATCAATGAACTGAAAGTTGCAAGTGAAGTAGTGGTAG 35908
               ************************************************************

Dromedary      ATCTAAGTGACAAAGCCAAAAAGGTCAGAGACTATAAACTAAGAATGTGAAGTTTGCAGA 35753
Wild           ATCTAAGTGACAAAGCCAAAAAGGTCAGAGACTATAAACTAAGAATGTGAAGTTTGCAGA 35546
Domestic       ATCTAAGTGACAAAGCCAAAAAGGTCAGAGACTATAAACTAAGAATGTGAAGTTTGCAGA 35968
               ************************************************************

Dromedary      AGTGGTTTTAAAGGTTTTAAAACACTTCTCAGTGATGAGTTCCAGAGTTTGATTCTGGCC 35813
Wild           AGTGGTTTTAAAGGTTTTAAAACACTTCCCAGTGATGAGTTCCAGAGTTTGATTCTGGCC 35606
Domestic       AGTGGTTTTAAAGGTTTTAAAACACTTCCCAGTGATGAGTTCCAGAGTTTGATTCTGGCC 36028
               **************************** *******************************

Dromedary      ATGGTAGAGGTATTAGTCAGGATAATATAAGCTATTCTGCATGAAAAGATTGACTCCAAT 35873
Wild           ATGGTAGAGGTATTAGTCAGGATAATATAAGCTATTCTGCATGAAAAGATTGACTCCAAT 35666
Domestic       ATGGTAGAGGTATTAGTCAGGATAATATAAGCTATTCTGCATGAAAAGATTGACTCCAAT 36088
               ************************************************************

Dromedary      CTCAGATTAAAATCTCAAAGAAAACAAAGCTTTATTTATCACTCATGCTGTTTTATTTTG 35933
Wild           CTCAGATTAAAATCTCAAAGAAAACAAAGCTTTATTTATCACTCATGCTGTTTTATTTTG 35726
Domestic       CTCAGATTAAAATCTCAAAGAAAACAAAGCTTTATTTATCACTCATGCTGTTTTATTTTG 36148
               ************************************************************

Dromedary      AAATGCTGCCATTTCAACACATAGCTTCCAGGTTTATACAGCAGGGGAGGAAAAGATGGC 35993
Wild           AAATGCTGCCATTTCAACACATAGCTTCCAGGTTTATACAGCAGGGGAGGAAAAGATGGC 35786
Domestic       AAATGCTGCCATTTCAACACATAGCTTCCAGGTTTATACAGCAGGGGAGGAAAAGATGGC 36208
               ************************************************************

Dromedary      ACTGAGTTAGTTCTTAAATGCTTGGATCTGGAAGCAAAACATGTCCTTTAGGCTCACAGT 36053
Wild           ACTGAGTTAGTTCTTAAATGCTTGGATCTGGAAGCAAAACATGTCCTTTAGGCTCACAGT 35846
Domestic       ACTGAGTTAGTTCTTAAATGCTTGGATCTGGAAGCAAAACATGTCCTTTAGGCTCACAGT 36268
               ************************************************************

Dromedary      CTTTTGTCCAGAAGTAGTCATATGGCCCCATCTATTTGCAATGGGGCTGGGAAATGTAAA 36113
Wild           CCTTTGTCCAGAAGTAGTCATATGGCCCCATCTATTTGCAATGGGGCTGGGAAATGTAAA 35906
Domestic       CCTTTGTCCAGAAGTAGTCATATGGCCCCATCTATTTGCAATGGGGCTGGGAAATGTAAA 36328
               * **********************************************************
```

```
Dromedary      GGAACACATGGATATTTAGTGAGCAATAAATGTCTCTTCACAGCAGCTGAGACGGATGAG  36173
Wild           GGAACACATGGATATTTAGTGAGCAATAAATGTCTCTTCACAGCAGCTGAGACGGATGAG  35966
Domestic       GGAACACATGGATATTTAGTGAGCAATAAATGTCTCTTCACAGCAGCTGAGACGGATGAG  36388
               ************************************************************

Dromedary      GAAGGAGATGATTAAAAACAAAGTGATTACAGAGCGTCATCCTTGATGATTGCCAGGGTT  36233
Wild           GAAGGAGATGATTAAAAACAAAGTGATTACAGAGCGTCATCCTTGATGATTGCCAGGGTT  36026
Domestic       GAAGGAGATGATTAAAAACAAAGTGATTACAGAGCGTCATCCTTGATGATTGCCAGGGTT  36448
               ************************************************************

Dromedary      GTGTTAGAAAAGAAACCCGAATGAGGTGCTAGAGTTTTCAGTGAAGGAGGTGGGCAGTTG  36293
Wild           GTGTTAGAAAAGAAACCCGAATGAGGTGCTAGAGTTTTCAGTGAAGGAGGTGGGCAGTTG  36086
Domestic       GTGTTAGAAAAGAAACCCGAATGAGGTGCTAGAGTTTTCAGTGAAGGAGGTGGGCAGTTG  36508
               ************************************************************

Dromedary      CAAGTTGGCAAATGAAAGCAATGAGAAGAAAAAGAGTTGGATAGTGAGTCTTAAAGAGAG  36353
Wild           CAAGTTGGCAAATGAAAGCAATGAGAAGAAAAAGAGTTGGATAGTGAGTCTTAAAGAGAG  36146
Domestic       CAAGTTGGCAAATGAAAGCAATGAGAAGAAAAAGAGTTGGATAGTGAGTCTTAAAGAGAG  36568
               ************************************************************

Dromedary      TGGGATTTGTCCGTGGGTAGAGAAGCAAGTTCCCTGGGACAATACATTTGGACACAATAA  36413
Wild           TGGGATTTGTCCGTGGGTAGAGAAGCAAGTTCCCTGGGACAATACATTTGGACACAATAA  36206
Domestic       TGGGATTTGTCCGTGGGTAGAGAAGCAAGTTCCCTGGGACAATACATTTGGACACAATAA  36628
               ************************************************************

Dromedary      AGAAATTTATCACCTGGGCAATAAGAATCCAGCCATTCCATTCGACCTCATGGAAACAGG  36473
Wild           AGAAATTTATCACCTGGGCAATAAGAATCCAGCCATTCCATTCGACCTCATGGAAACAGG  36266
Domestic       AGAAATTTATCACCTGGGCAATAAGAATCCAGCCATTCCATTCGACCTCATGGAAACAGG  36688
               ************************************************************

Dromedary      ACTATTGCTCAATAGAGTCTTTCAGAGAGCTAAGAATCGCCATAAAGCTGGTCTTCATAA  36533
Wild           ACTATTGCTCAATAAAGTCTTTCAGAGAGCTAAGAATCGCCATAAAGCTGGTCTTCATAA  36326
Domestic       ACTATTGCTCAATAAAGTCTTTCAGAGAGCTAAGAATCGCCATAAAGCTGGTCTTCATAA  36748
               ************** *********************************************

Dromedary      ACACAGAATGTTGACTGGGTGGCAGGAAATTATTTCTGAATATTAATTGATATCTTTCCT  36593
Wild           ACACAGAATGTTGACTGGGTGGCAGGAAATTATTTCTGAATATTAATTGATATCTTTCCT  36386
Domestic       ACACAGAATGTTGACTGGGTGGCAGGAAATTATTTCTGAATATTAATTGATATCTTTCCT  36808
               ************************************************************

Dromedary      TGTACTTCTTTCTCTCCTTTCGTATTTGCCCCTATAAAGAGCTAGTTTGTGCTCACTGCC  36653
Wild           TGTACTTCTTTCTCTCCTTTCATATTTGCCCCTATAAAGAGCTAGTTTGTGCTCACTGCC  36446
Domestic       TGTACTTCTTTCTCTCCTTTCATATTTGCCCCTATAAAGAGCTAGTTTGTGCTCACTGCC  36868
               ********************* **************************************

Dromedary      TTAGGGTAAGGACTGTGGCAAGGTGGAAAGCAAAGGACCTGAAATACTACTCCTAAGTTC  36713
Wild           TTAGGGTAAGGACTGTGGCAAGGTGGAAAGCAAAGGACCTGAAATACTACTCCTAAGTTC  36506
Domestic       TTAGGGTAAGGACTGTGGCAAGGTGGAAAGCAAAGGACCTGAAATACTACTCCTAAGTTC  36928
               ************************************************************

Dromedary      TAGTCTACAAACAGCCTAGGTCATGACAAGAGTGGAAAGGTATGGTAGGCAGAACAATGC  36773
Wild           TAGTCTACAAACAGCCTAGGCCATGACAAGAGTGGAAAGGTGTGGTAGGCAGAACAATGC  36566
Domestic       TAGTCTACAAACAGCCTAGGCCATGACAAGAGTGGAAAGGTGTGGTAGGCAGAACAATGC  36988
               ******************** ******************** ******************

Dromedary      TCCCCCCTCAAAGATGTCCATGTCTTAGCCCTCGGAACCTGTGAGTGTGGTAGGCTACAC  36833
Wild           TCCCCCCTCAAAGATGTCCATGTCTTAGCCCTCGGAACCTGTGAGTGTGGTAGGCTACAC  36626
Domestic       TCCCCCCTCAAAGATGTCCATGTCTTAGCCCTCGGAACCTGTGAGTGTGGTAGGCTACAC  37048
               ************************************************************

Dromedary      TGCAAGGGGGAAATAAGATCACAGATGAATTAAAATTACTAATCAGCTGACCTGAAATTA  36893
Wild           TGCAAGGGGGAAATAAGATCACAGATGAATTAAAATTACTAATCAGCTGACCTGAAATTA  36686
Domestic       TGCAAGGGGGAAATAAGATCACAGATGAATTAAAATTACTAATCAGCTGACCTGAAATTA  37108
               ************************************************************

Dromedary      AGGACATTTAAATGTAATGACAAGAGTCTTTGCATATGGAAGAGAGAGGCAGAAGAGTTA  36953
Wild           AGGACATTTAAATGTAATGACAAGAGTCTTTGCATATGGAAGAGAGAGGCAGAAGAGTTA  36746
Domestic       AGGACATTTAAATGTAATGACAAGAGTCTTTGCATATGGAAGAGAGAGGCAGAAGAGTTA  37168
               ************************************************************
```

```
Dromedary     GTGTCAGAGCTATACAGCATCAAAAGACTTCCCTGGCTATCACTGGCTTGGAAGACGGAA  37013
Wild          GTGTCAGAGCTATACAGCATCAAAAGACTTCCCTGGCTATCACTGGCTTGGCAGACGGAA  36806
Domestic      GTGTCAGAGCTATACAGCATCAAAAGACTTCCCTGGCTATCACTGGCTTGGCAGACGGAA  37228
              *************************************************** ********

Dromedary     GGGGGACATGAACCAAGAAATGTGGGCCATCTTGAGAAGCTGGAAAAGAAATGCATTCAC  37073
Wild          GGGGGACATGAACCAAGAAATGTGGGCCATCTTGAGAAGCTGGAAAAGAAATGCATTCAC  36866
Domestic      GGGGGACATGAACCAAGAAATGTGGGCCATCTTGAGAAGCTGGAAAAGAAATGCATTCAC  37288
              ************************************************************

Dromedary     CCCTAGAGCCCTCAGAGGGAACACAGCTCTGCCAACACTGCTTTTAGCCCAGTGAGACCC  37133
Wild          CCCTAGAGCCCTCAGAGGGAACACAGCTCTGCCAACACTGCTTTTAGCCCAGTGAGACCC  36926
Domestic      CCCTAGAGCCCTCAGAGGGAACACAGCTCTGCCAACACTGCTTTTAGCCCAGTGAGACCC  37348
              ************************************************************

Dromedary     ATTTCAGACTTCTGAACTTCAGAACTCCAAGATAAATCTGTGTTGTTTCCAGTCACTAAA  37193
Wild          ATTTCAGACTTCTGAACTTCAGAACTCCAAGATAAATCTGTGTTGTTTCCAGTCACTAAA  36986
Domestic      ATTTCAGACTTCTGAACTTCAGAACTCCAAGATAAATCTGTGTTGTTTCCAGTCACTAAA  37408
              ************************************************************

Dromedary     TGTGCAGTGATATGTTACAGCCCCAGTAGGAAGTTAATACAAAAGGCAAAGAAAAGGTTT  37253
Wild          TGTGCAGTGATATGTTACAGCCCCAGTAGGAAGTTAATACAAAAGGCAAAGAAAAGGTTT  37046
Domestic      TGTGCAGTGATATGTTACAGCCCCAGTAGGAAGTTAATACAAAAGGCAAAGAAAAGGTTT  37468
              ************************************************************

Dromedary     TGAGAGTTGGCAGTCGGAGGAGGGAGTTCTCTAGGAGGAGGATAGGTCAAGCGATATCCC  37313
Wild          TGAGAGTTGGCAGTCGGAGGAGGGAGTTCTCTAGGAGGAGGATAGGTCAAGCGATATCCC  37106
Domestic      TGAGAGTTGGCAGTCGGAGGAGGGAGTTCTCTAGGAGGAGGATAGGTCAAGCGATATCCC  37528
              ************************************************************

Dromedary     AGCAGAAGCTGAGCAGAACCTGGAGATTAGTTAACAGCAGAAGAGAAAAGCCCAGGACCC  37373
Wild          AGCAGAAGCTGAGCAGAACCTGGAGATTAGTTAACAGCAGAAGAGAAAAGCCCAGGACCC  37166
Domestic      AGCAGAAGCTGAGCAGAACCTGGAGATTAGTTAACAGCAGAAGAGAAAAGCCCAGGACCC  37588
              ************************************************************

Dromedary     TGCACTCCAGGCCAACATACAGGGAGGCAGCAAACTCCCTGAAACAGAGGTGTGTGATCC  37433
Wild          TGCACTCCAGGCCAACATACAGGGAGGCAGCAAACTCCCTGAAACAGAGGTGTGTGATCC  37226
Domestic      TGCACTCCAGGCCAACATACAGGGAGGCAGCAAACTCCCTGAAACAGAGGTGTGTGATCC  37648
              ************************************************************

Dromedary     GTGCTTCACACACGGTAACGTGCAAGGAGACGGCAAGACCCTGATGGAGGACGGCTGTGT  37493
Wild          GTGCTTCACACACGGTAACGTGCAAGGAGACGGCAAGACCCTGATGGAGGACGGCTGTGT  37286
Domestic      GTGCTTCACACACGGTAACGTGCAAGGAGACGGCAAGACCCTGATGGAGGACGGCTGTGT  37708
              ************************************************************

Dromedary     GCTTAGTCAGAAGGCACTTTCATGAGCCTTATGAAGAGTTCATTTCATTTCAGCTGACAG  37553
Wild          GCTTAGTCAGAAGGCACTTTCATGAGCCTTATGAAGAGTTCACTTCATTTCAGCTGACAG  37346
Domestic      GCTTAGTCAGAAGGCACTTTCATGAGCCTTATGAAGAGTTCACTTCATTTCAGCTGACAG  37768
              ****************************************** *****************

Dromedary     TATTTTGCCCTCTTTTCCAGTGCTCTACTCGTAACATTCTTTTTGTTTAGTGTGGTGACT  37613
Wild          TATTTTGCCCTCTTTTCCAGTGCTCTACTCGTAACATTCTTTTTGTTTAGTGTGGTGACT  37406
Domestic      TATTTTGCCCTCTTTTCCAGTGCTCTACTCGTAACATTCTTTTTGTTTAGTGTGGTGACT  37828
              ************************************************************

Dromedary     CTGTAAAGTGCTAACCTGGTTAGCCTGAACTAAATTTCTTGGTTTTCCTTTCCTATAGGT  37673
Wild          CTGTAAAGTGTTAACCTGGTTAGCCTGAACTAAATTTCTTGGTTTTCCTTTCCTATAGGT  37466
Domestic      CTGTAAAGTGCTAACCTGGTTAGCCTGAACTAAATTTCTTGGTTTTCCTTTCCTATAGGT  37888
              ********** *************************************************

Dromedary     TAAGGTGTGCCCCAAGAAAGATTCTGGTGGGCGATGTGGAGGGCGGAAGGGAAGGAGCAT  37733
Wild          TAAGGTGTGCCCCAAGAAAGATTCTGGTGGGCGATGTGGAGGGCGGAAGGGAAGGAGCAT  37526
Domestic      TAAGGTGTGCCCCAAGAAAGATTCTGGTGGGCGATGTGGAGGGCGGAAGGGAAGGAGCAT  37948
              ************************************************************

Dromedary     CCTTTATGTAGCTCAACTGTGTGGTCACTGACCAACTGGCTCATCTCCAGGGTGTGAGGC  37793
Wild          CCTTTATGTAGCTCAACTGTGTGGTCACTGACCAACTGGCTCATCTCCAGGGTGTGAGGC  37586
Domestic      CCTTTATGTAGCTCAACTGTGTGGTCACTGACCAACTGGCTCATCTCCAGGGTGTGAGGC  38008
              ************************************************************
```

```
Dromedary      AGCGGACGCTGCTGAAACCACTCCACCTCCGCCCCCCACCCCCATCCTCCTGCCGCTTCT  37853
Wild           AGCGGACGCTGCTGAAACCACTCCACCTCCACCCCCCACCCCCATCCTCCTGCCGCTTCT  37646
Domestic       AGCGGACGCTGCTGAAACCACTCCACCTCCACCCCCCACCCCCATCCTCCTGCCGCTTCT  38068
               ****************************** *****************************

Dromedary      CTGACTCCTGAGCCAAGGGTAGCTGTTTAGCTCCGTGAAGAAAGACCCACCCCCCACCCT  37913
Wild           CTGACTCCTGAGCCAAGGGTAGCTGTTTAGCTCCGTGAAGAAAGACCCACCCCCCACCCT  37706
Domestic       CTGACTCCTGAGCCAAGGGTAGCTGTTTAGCTCCGTGAAGAAAGACCCACCCCCCACCCT  38128
               ************************************************************

Dromedary      CACCATGAGGGGCAGTGGCCATAAGAACTGACACAGTCTTCCATCCATCCTGTGGGCTCC  37973
Wild           CACCATGAGGGACAGTGGCCATAAGAACTGACACAGTCTTCCATCCATCCTGTGGGCTCC  37766
Domestic       CACCATGAGGGACAGTGGCCATAAGAACTGACACAGTCTTCCATCCATCCTGTGGGCTCC  38188
               *********** ************************************************

Dromedary      ACCTCAAGCTGTGGGTTCTGGCTGGTTCTTGCTCTTTCCTACATCACATCCCTCTTTCCT  38033
Wild           ACCTCAAGCTGTGGGTTCTGGCTGGTTCTTGCTCTTTCCTACATCACATCCCTCTTTCCT  37826
Domestic       ACCTCAAGCTGTGGGTTCTGGCTGGTTCTTGCTCTTTCCTACATCACATCCCTCTTTCCT  38248
               ************************************************************

Dromedary      TCCCAACTGCTTGCCAGGGACTTCCAGGTCCAGGTTCAGAGGTGAAGACAGCAACCTTAC  38093
Wild           TCCCAACTGCTTGCCAGGGACTTCCAGGTCCAGGTTCAGAGGTGAAGACAGCAACCTTAC  37886
Domestic       TCCCAACTGCTTGCCAGGGACTTCCAGGTCCAGGTTCAGAGGTGAAGACAGCAACCTTAC  38308
               ************************************************************

Dromedary      AGAGACTGCTTAACTAGATCCCCCATTACGTAAGGTCAAATTCCTGCAATAAGCCCCTCG  38153
Wild           AGAGACTGCTTAACTAGAT-CCCCATTACGTAAGGTCAAATTCCTGCAATAAACCCCTCG  37945
Domestic       AGAGACTGCTTAACTAGAT-CCCCATTACGTAAGGTCAAATTCCTGCAATAAACCCCTCG  38367
               ******************* ******************************** *******

Dromedary      AATATGATATGATATAAGATAATGTATATGTCTATATACACCACACTCCTCTGAACCCCT  38213
Wild           AATATGATATGATATAAGATAATGTGTATGTCTATATACACCACACTCCTCTGAACCCCT  38005
Domestic       AATATGATATGATATAAGATAATGTGTATGTCTATATACACCACACTCCTCTGAACCCCT  38427
               ************************* **********************************

Dromedary      TGAGTTTCCATCATATTTATTTCTGATATTACCAAAAATTCTTTTCAATGTCTGTGATTT  38273
Wild           TGAGTTTCCATCATATTTATTTCTGATATTACCAAAAATTCTTTTCAATTTCTTTGATTT  38065
Domestic       TGAGTTTCCATCATATTTATTTCTGATATTATCAAAAATTCTTTTCAATTTCTTTGATTT  38487
               ****************************** ****************** *** ******

Dromedary      GTAGGAATTACATTTAATGTCTTTCATGGATTATTATCCTGGTCATTTAATCTTTTAACT  38333
Wild           GTAGGAATTACATTTAATGTCTTTCATGGATTATTATCCTGGTCATTTAATCTTTTAACT  38125
Domestic       GTAGGAATTACATTTAATGTCTTTCATGGATTATTATCCTGGTCATTTAATCTTTTAACT  38547
               ************************************************************

Dromedary      TACCCCTCACATTCTTTGAATACCATTTTTCCTGGACCAGTGGAAAGTAAGTGACTCATT  38393
Wild           TACCCCTCACATTCTTTGAATACCATTTTTCCTGGACCAGTGGAAAGTAAGTGACTCATT  38185
Domestic       TACCCCTCACATTCTTTGAATACCATTTTTCCTGGACCAGTGGAAAGTAAGTGACTCATT  38607
               ************************************************************

Dromedary      CAGATAAAGTAATCACTTGTTTCCTATTTAAAGCTATCTTTAAAACTCCTCACCTCTCCT  38453
Wild           CAGATAAAGTAATCACTTGTTTCCTATTTAAAGCTATCTTTAAAACTCATTACCTCTCCT  38245
Domestic       CAGATAAAGTAATCACTTGTTTCCTATTTAAAGCTATCTTTAAAACTCATTACCTCTCCT  38667
               ************************************************ * *********

Dromedary      TTAACACCCTCCCAAAAGCATTGAAAGGATCATTCAAAATACATAATGGATATAATGCTA  38513
Wild           TTAACACCCTCCCAAAAGCACTGAAAGGATCATTCAAAATACATAATGGATATAATGCTA  38305
Domestic       TTAACACCCTCCCAAAAGCATTGAAAGGATCATTCAAAATACATAATGGATATAATGCTA  38727
               ******************** ***************************************

Dromedary      ATAGGGAACCAGTGATCCATGAATAAATTACGAATAGTGACAGAAGGCAAGGCGACTTTT  38573
Wild           ATAGGGAACCAGTGATCCATGAATAAATTACGAATAGTGACAGAAGGCAAGGCGACTTTT  38365
Domestic       ATAGGGAACCAGTGATCCATGAATAAATTACGAATAGTGACAGAAGGCAAGGCGACTTTT  38787
               ************************************************************

Dromedary      ATGATTCAGCTCAACAGACTTTGCAGGCAGGAAAACATCCTGTAATCAATCTTGTGATTG  38633
Wild           ATGATTCAGCTCAACAGACTTTGCAGGCAGGAAAACATCCTGTAATCAATCTTGTGATTG  38425
Domestic       ATGATTCAGCTCAACAGACTTTGCAGGCAGGAAAACATCCTGTAATCAATCTTGTGATTG  38847
               ************************************************************
```

```
Dromedary      CTGAAAACATGGTTCAAAATAAAAAATGAAGTAATCTTGCACATATAGCCAGAAACCTAA 38693
Wild           CTGAAAACATGGTTCAAAATAAAAAATGAAGTAATCTTGCACATATAGCCAGAAACCTAA 38485
Domestic       CTGAAAACATGGTTCAAAATAAAAAATGAAGTAATCTTGCACATATAGCCAGAAACCTAA 38907
               ************************************************************

Dromedary      GAACAAACACTTGCAAACTTAGAGCTCCACTTCTCGGGCTCCCTTTTAAACAAAACAGGC 38753
Wild           GAACAAACACTTGCAAACTTAGAGCTCCACTTCTCGGGCTCCCTTTTAAACAAAACAGGC 38545
Domestic       GAACAAACACTTGCAAACTTAGAGCTCCACTTCTCGGGCTCCCTTTTAAACAAAACAGGC 38967
               ************************************************************

Dromedary      TGCACAGAAGGAGTGGGGGATCTGATAGTCAGATACGCCTGGGTCGAAATCATGGCTCCG 38813
Wild           TGCACAGAAGAAGTGGGGGATCTGATAGTCAGATACGCCTGGGTCGAAATCATGGCTCCG 38605
Domestic       TGCACAGAAGGAGTGGGGGATCTGATAGTCAGATACGCCTGGGTCGAAATCATGGCTCCG 39027
               ********** *************************************************

Dromedary      ACATTCTGTAGCTCTGGGATTTTAAGCTGATGTTTTCACTTTCAGAAGCTCAGTTTTCTC 38873
Wild           ACATTCTGTAGCTCTGGGATTTTAAGCTGATGTTTTCACTTTCAGAAGCTCAGTTTTCTC 38665
Domestic       ACATTCTGTAGCTCTGGGATTTTAAGCTGATGTTTTCACTTTCAGAAGCTCAGTTTTCTC 39087
               ************************************************************

Dromedary      ATCTACAAAGTAGCAGTAATGCTACCAGCCTCATAGGATTGTGGAAAAGATAATAAATAA 38933
Wild           ATCTACAAAGTAGCAGTAATGCTACCAGCCTCATAGGATTGTGGAAAAGATAATAAATAA 38725
Domestic       ATCTACAAAGTAGCAGTAATGCTACCAGCCTCATAGGATTGTGGAAAAGATAATAAATAA 39147
               ************************************************************

Dromedary      TATGTAAAAGTTCTGACACATAAAAGGCACTAGGGGTCACCACCTATTATTATTAAATAC 38993
Wild           TATGTAAAAGTTCTGACACATAAAAGGCACTAGGGGTCACCACCTATTATTATTAAATAC 38785
Domestic       TATGTAAAAGTTCTGACACATAAAAGGCACTAGGGGTCACCACCTATTATTATTAAATAC 39207
               ************************************************************

Dromedary      TCCAAATAGCCCGTGCATCCCCTTTGTTAATACCTTTAAATATATATGTATATTAAATAT 39053
Wild           TCCAAATAGCCCGTGCATCCCCTTTGTTAATACCTTTAAATATATATGTATATTAAATAT 38845
Domestic       TCCAAATAGCCCGTGCATCCCCTTTGTTAATACCTTTAAATATATATGTATATTAAATAT 39267
               ************************************************************

Dromedary      ATGTATATATTGATATATAATATAATATATATTAAATAGCTATATATACATATCTGAAAA 39113
Wild           ATGTATATATTGATATATAATATAATATATATTAAATAGCTATATATACATATCTGAAAA 38905
Domestic       ATGTATATATTGATATATAATATAATATATATTAAATAGCTATATATACATATCTGAAAA 39327
               ************************************************************

Dromedary      CAATGTCCTGGAAATCCCTACAGGTCGGTGCATATGTACTTTCCAATTGCTTAATCTGGA 39173
Wild           CAATGTCCTGGAAATCCCTACAGGTCGGTGCATATGTACTTTCCAATTGCTTAATCTGGA 38965
Domestic       CAATGTCCTGGAAATCCCTACAGGTCGGTGCATATGTACTTTCCAATTGCTTAATCTGGA 39387
               ************************************************************

Dromedary      ACTTATCTTATGTTCCAGCCACAAGAGCCATGTTCACCAAAACTCTGTCTACAGAATCTG 39233
Wild           ACTTATCTTATGTTCCAGCCACAAGAGCCATGTTCACCAAAACTCTGTCTACAGAATCTG 39025
Domestic       ACTTATCTTATGTTCCAGCCACAAGAGCCATGTTCACCAAAACTCTGTCTACAGAATCTG 39447
               ************************************************************

Dromedary      AAGTCAGATTAAAGCAATGTGAGACCGTGTCCTCTTTATGGCTGGGGGAAGGTGGACGCT 39293
Wild           AAGTCAGATTAAAGCAATGTGAGACCGTGTCCTCTTTATGGCTGGGGGAAGGTGGACGCT 39085
Domestic       AAGTCAGATTAAAGCAATGTGAGACCGTGTCCTCTTTATGGCTGGGGGAAGGTGGACGCT 39507
               ************************************************************

Dromedary      CTTGGCAGGAACACTGAAGCCTCAGAGAAAGTAAGGCCCTCGTCCTTCATCAGAGAGCTG 39353
Wild           CTTGGCAGGAACACTGAAGCCTCAGAGAAAGTAAGGCCCTCGTCCTTCATCAGAGAGCTG 39145
Domestic       CTTGGCAGGAACACTGAAGCCTCAGAGAAAGTAAGGCCCTTGTCCTTCATCAGAGAGCTG 39567
               **************************************** *******************

Dromedary      CTGCTTCAGTAGCTGCACTAAGTGCCTGGTCACAAGACAGGGGAGGACAGGAAGAGGCCA 39413
Wild           CTGCTTCAGTAGCTGCACTAAGTGCCTGGTCACAAGACANNNNNNNNNNNN----NNNNN 39201
Domestic       CTGCTTCAGTAGCTGCACTAAGTGCCTGGTCACAAGACAGGGGAGGACAGGAAGAGGCCA 39627
               ***************************************

Dromedary      GGCCCTTCCCGGCCCAACTTGAAGGTGAGTGGGTGAGCGGACTGCGTTCCACTCTGACGG 39473
Wild           NNNNNNNNNNGGCCCAACTTGAAGGTGAGTGGGTGAGCGGACTGCGTTCCACTCTGACGG 39261
Domestic       GGCCCTTCCCGGCCCAACTTGAAGGTGAGTGGGTGAGCGGACTGCGTTCCACTCTGACGG 39687
                         **************************************************
```

```
Dromedary     CCTCCAGCTGTTTTCCCACCTTCTTCTGTAGGTTTCCATGGTCAAGTTACCAAGCCAGGG 39533
Wild          CCTCCAGCTGTTTTCCCACCTTCTTCTGTAGGTTTCCATGGTCAAGTTACCAAGCCAGGG 39321
Domestic      CCTCCAGCTGTTTTCCCACCTTCTTCTGTAGGTTTCCATGGTCAAGTTACCAAGCCAGGG 39747
              ************************************************************

Dromedary     AATTCCCAAAGTGAAAATACATTGACAGCTGTGTTTGGAATCTATTACCAACATTTTAAC 39593
Wild          AATTCCCAAAGTGAAAATACATTGACAGCTGTGTTTGGAATCTATTACCAACATTTTAAC 39381
Domestic      AATTCCCAAAGTGAAAATACATTGACAGCTGTGTTTGGAATCTATTACCAACATTTTAAC 39807
              ************************************************************

Dromedary     AATTATACACAGGGCCAAATTTGACCTCGAAATTACTGGAAACTTATACCCAAAAGACTG 39653
Wild          AATTATACACAGGGCCAAATTTGACCTCGAAATTACTGGAAACTTATACCCAAAAGACTG 39441
Domestic      AATTATACACAGGGCCAAATTTGACCTCGAAATTACTGGAAACTTATACCCAAAAGACTG 39867
              ************************************************************

Dromedary     AATGAAAATGAAGAGAGCACATAAATAAAAATGACCCCCACGAATAAAGTGAAATTATGG 39713
Wild          AATGAAAATGAAGAGAGCACATAAATAAAAATGACCCCCACGAATAAAGTGAAATTATGG 39501
Domestic      AATGAAAATGAAGAGAGCACATAAATAAAAATGACCCCCACGAATAAAGTGAAATTATGG 39927
              ************************************************************

Dromedary     GTGAAGAAAGGAAGACATAAAAACAAAACATTATCCTGTTACAGACAATAGTTTAAAAAA 39773
Wild          GTGAAGAAAGGAAGACATGAAAACAAAACATTATCCTGTTACAGACAATAGTTTAAAAAA 39561
Domestic      GTGAAGAAAGGAAGACATGAAAACAAAACATTATCCTGTTACAGACAATAGTTTAAAAAA 39987
              ****************** *****************************************

Dromedary     TTCTGGTGCCAAGGGTATGAATCTTTACTTCTATAACCTTAAATAAAACTCTGTTTCCTC 39833
Wild          TTCTGGTGCCAAGGGTATGAATCTTTACTTCTATAACCTTAAATAAAACTCTGTTTCCTC 39621
Domestic      TTCTGGTGCCAAGGGTATGAATCTTTACTTCTATAACCTTAAATAAAACTCTGTTTCCTC 40047
              ************************************************************

Dromedary     CTACCTGTGCTACAAGGAATCCAAGAATATTTATTTTCTGATTCCTTGCCACATGTATTA 39893
Wild          CTACCTGTGCTACAAGGAATCCAAGAATATTTATTTTCTGATTCCTTGCCACATGTATTA 39681
Domestic      CTACCTGTGCTACAAGGAATCCAAGAATATTTATTTTCTGATTCCTTGCCACATGTATTA 40107
              ************************************************************

Dromedary     CTATTATTTTAGGTTAGCATTTAATGACTTTTTTTTTTAAAGCTGTAAACATTCATCTTC 39953
Wild          CTATTATTTTAGGTTAGCATTTAATGACTTTTTTTTTTAAAGCTGTAAACATTCATCTTC 39741
Domestic      CTATTATTTTAGGTTAGCATTTAATGAC-TTTTTTTTTAAAGCTGTAAACATTCATCTTC 40166
              **************************** *******************************

Dromedary     TGCCCTGATATGCATATATGTACTATCTATATATTTCTATCTATGGTCTGAAGTATCTGA 40013
Wild          TGCCCTGATATGCATATATGTA--------------CTATCTATGGTCTGAAGTATCTGA 39787
Domestic      TGCCCTGATATGCATATATGTA--------------CTATCTATGGTCTAAAGTATCTGA 40212
              **********************              ************* **********

Dromedary     AAGTGGTCCAGAAGAGCATCATCTTTATGACTAGAGACATTATTATAGTCTTAAAAATGA 40073
Wild          AAGTGGTCCAGAAGAGCATCATCTTTATGACTAGAGACATTATTATAGTCTTAAAAATGA 39847
Domestic      AAGTGGTCCAGAAGAGCATCATCTTTATGACTAGAGACATTATTATAGTCTTAAAAATGA 40272
              ************************************************************

Dromedary     AAAACTGACAACACAGCATTTCTAGTACTTTCCCTTTCTTCTGCTAGCAATGAGCCCAAG 40133
Wild          AAAACTGACAACACAGCATTTCTAGTACTTTCCCTTTCTTCTGCTAGCAATGAGCCCAAG 39907
Domestic      AAAACTGACAACACAGCATTTCTAGTACTTTCCCTTTCTTCTGCTAGCAATGAGCCCAAG 40332
              ************************************************************

Dromedary     GTAAGTGAAATTCTATTCACAGAAGTTAGAATTAGACAACAGAACTCTGGTTTTAATAGC 40193
Wild          GTAAGTGAAATTCTATTCACAGAAGTTAGAATTAGACAATAGAACTCTGGTTTTAATAGC 39967
Domestic      GTAAGTGAAATTCTATTCACAGAAGTTAGAATTAGACAATAGAACTCTGGTTTTAATAGC 40392
              *************************************** ********************

Dromedary     CAGTCCTCTCCTCTGATCAGATACAGCTATCCCCTGACCAAGGAGATAACAGTTTTTACA 40253
Wild          CAGTCCTCTCCTCTGATCAGATACAGCTATCCCCTGACCAAGGAGATAACAGTTTTTACA 40027
Domestic      CAGTCCTCTCCTCTGATCAGATACAGCTATCCCCTGACCAAGGAGATAACAGTTTTTACA 40452
              ************************************************************

Dromedary     GATCAGATGGACAATGATTCTTAAAAGGCTATTACCCTGGGTTGGCAATGGTTTGGGGAA 40313
Wild          GATCAGATGGACAATGATTCTTAAAAGGCTATTACCCTGGGTTGGCAATGGTTTGGGGAA 40087
Domestic      GATCAGATGGACAATGATTCTTAAAAGGCTATTACCCTGGGTTGGCAATGGTTTGGGGAA 40512
              ************************************************************
```

```
Dromedary      AACAGGCACTTCTGTTCCATTTTTTAAAAGAACTGAAGTATAGTTAGTTTACAATATTGT  40373
Wild           AACAGGCACTTCTGTTCCATTTTTTAAAAGAACTGAAGTATAGTTAGTTTACAATATTGT  40147
Domestic       AACAGGCACTTCTGTTCCATTTTTTAAAAGAACTGAAGTATAGTTAGTTTACAATATTGT  40572
               ************************************************************

Dromedary      GTCAATTTCTGGTGTACAGTGTAATGTTTCAGTCATACATATACATACACATATTCCTTT  40433
Wild           GTCAATTTCTGGTGTGCAGTGTAATGTTTCAGTCATACATATACATACACATATTCCTTT  40207
Domestic       GTCAATTTCTGGTGTGCAGTGTAATGTTTCAGTCATACATATACATACACATATTCCTTT  40632
               *************** ********************************************

Dromedary      CCATATTCTTTTTCATTATAGGCTACTGCAAGGTATTGAATATAGTTCCCTGTGCTACAC  40493
Wild           CCATATTCTTTTTCATTATAGGCTACTGCAAGGTATTGAATATAGTTCCCTGTGCTACAC  40267
Domestic       CCATATTCTTTTTCATTATAGGCTACTGCAAGGTATTGAATATAGTTCCCTGTGCTACAC  40692
               ************************************************************

Dromedary      AGTAGAAATTTGTTCTTCAACCTATTTTATATATAGTAGTTAGTATCTGCAAATGGAAAT  40553
Wild           AGTAGAAATTTGTTCTTC-ACCTATTTTATATATAGTAGTTAGTATCTGCAAATGGAAAT  40326
Domestic       AGTAGAAATTTGTTCTTC-ACCTATTTTATATATAGTAGTTAGTATCTGCAAATGGAAAT  40751
               ****************** *****************************************

Dromedary      AGACACTTTTGAATACTCCTACTGGACACGTAAATCAATACAATTTCCAAAGGATAATTT  40613
Wild           AGACACTTTTGAATACTCCTACTGGACACGTAAATCAATACATTTTCCAAAGGATAATTT  40386
Domestic       AGACACTTTTGAATACTCCTACTGGACACGTAAATCAATACATTTTCCAAAGGATAATTT  40811
               ****************************************** *****************

Dromedary      GGAAAAATGATTTAAATTCTTAAAAATGAATTTATTGTAAGTACATATATGTGTATTTAC  40673
Wild           GGAAAAATGATTTAAATTCTTAAAAATGAATTTATTGTGAGTACATATATGTGTATTTAC  40446
Domestic       GGAAAAATGATTTAAATTCTTAAAAATGAATTTATTGTGAGTACATATATGTGTATTTAC  40871
               ************************************** *********************

Dromedary      AAAAAGATTTACCATCAAGGATATGTTTTGAAAAGTTATGACAGGAATTTGAAAGAACCT  40733
Wild           AAAAAGATTTACCATCAAGGATATGTTTTGAAAAGTTATGACAGGAATTTGAAAGAACCT  40506
Domestic       AAAAAGATTTACCATCAAGGATATGTTTTGAAAAGTTATGACAGGAATTTGAAAGAACCT  40931
               ************************************************************

Dromedary      AAATGTCCTACAAGAAGATATTGATTAAATAGGTAAATACTAATATGACATAATACAGAT  40793
Wild           AAATGTCCTACAAGAAGATATTGATTAAATAGGTAAATACTAATATGACATAATACAGAT  40566
Domestic       AAATGTCCTACAAGAAGATATTGATTAAATAGGTAAATACTAATATGACATAATACAGAT  40991
               ************************************************************

Dromedary      AGTCATTAAATAATAATGTGTAAAAAAATATTTAGTGACTTGGGAAAATATTCACACTATA  40853
Wild           AGTCATTAAATAATAATGTGTAAAAAAATATTTAGTGACTTGGGAAAATATTCACACTGTA  40626
Domestic       AGTCATTAAATAATAATGTGTAAAAAAATATTTAATGACTTGGGAAAATATTCACACTGTA  41051
               ********************************* *********************** **

Dromedary      GTCAGTTAACTTTAGTTTATTTATTGAGTAGATAAACCAAAATGTTAACAGTAATTATTC  40913
Wild           GTCAGTTAACTTTAGTTTATTTATTGAGTAGATAAACCAAAGTGTTAACAGTAATTATTC  40686
Domestic       GTCAGTTAACTTTAGTTTATTTATTGAGTAGATAAACCAAAGTGTTAACAGTAATTATTC  41111
               ***************************************** ******************

Dromedary      CTAAAGGAAGGAATTATGAATGATTTTTAGTTTTATTTTTGTACTTTGTATACTTTTTAA  40973
Wild           CTAAAGGAAGGAATTATGAATGATTTTTAGTTTTATTTTTGTACTTTGTATACTTTTTAA  40746
Domestic       CTAAAGGAAGGAATTATGAATGATTTTTAGTTTTATTTTTGTACTTTGTATACTTTTTAA  41171
               ************************************************************

Dromedary      AATTTTCCATGCTGAATATATGGAATATATATTACTTTATAATCATACTCTTTCTGGTAT  41033
Wild           AATTTTCCATGCTGAATATATGGAATATATATTACTTTATAATCATACTCTTTCTGGTAT  40806
Domestic       AATTTTCCATGCTGAATATATGGAATATATATTACTTTATAATCATACTCTTTCTGGTAT  41231
               ************************************************************

Dromedary      TAAAATGGAGGTTGGAAAGAGGCTTTAGGGCAGACTGAACCCCTTTAGAGTAGTCTTAAA  41093
Wild           TAAAATGGAGGTTGGAAAGAGGCTTTAGGGCAGACTGAACCCCTTTAGAGTAGTCTTAAA  40866
Domestic       TAAAATGGAGGTTGGAAAGAGGCTTTAGGGCAGACTGAACCCCTTTAGAGTAGTCTTAAA  41291
               ************************************************************

Dromedary      TTGAATTATAGTTCTAAAGAGTCCTAAGATTCAGGATTTAAGACTTAGGACTTAGTCCTA  41153
Wild           TTGAATTATAGTTCTAAAGAGTCCTAAGATTCAGGATTTAAGACTTAGGACTTAGTCCTA  40926
Domestic       TTGAATTATAGTTCTAAAGAGTCCTAAGATTCAGGATTTAAGACTTAGGACTTAGTCCTA  41351
               ************************************************************
```

```
Dromedary      AAACTCAGTACTCTGGGGAGTCCTGTGACTTTCTTAGAGTAATGCTAACTTTGTCGGATG 41213
Wild           AAACTCAGTACTCTGGGGAGTCCTGTGACTTTCTTAGAGTAATGCTAACTTTGTCGGATG 40986
Domestic       AAACTCAGTACTCTGGGGAGTCCTGTGACTTTCTTAGAGTAATGCTAACTTTGTCGGATG 41411
               ************************************************************

Dromedary      CCTGCCTTTCAGCATCACAAACACAGCAATCTCCAACTCTCTCTGTTATATGTACAGTCA 41273
Wild           CCTGCCTTTCAGCATCACAAACACAGCAATCTCCAACTCTCTCTGTTATATGTACAGTCA 41046
Domestic       CCTGCCTTTCAGCATCACAAACACAGCAATCTCCAACTCTCTCTGTTATATGTACAGTCA 41471
               ************************************************************

Dromedary      CTGTTTTCACAGCAAAAACATGGTATAATTTGACAAAGGACAGGGTACTTGGAATTAAAG 41333
Wild           CTGTTTTCACAGCAAAAACATGGTATAATTTGACAAAGGACAGGGTACTTGGAATTAGAG 41106
Domestic       CTGTTTTCACAGCAAAAACATGGTATAATTTGACAAAGGACAGGGTACTTGGAATTAGAG 41531
               ********************************************************* **

Dromedary      CTGTCATTTAACATTTAGGTCCTTTCAAATTCCTGTCATTACCCCACTCTTGGCTATATG 41393
Wild           CTGTCATTTAACATTTAGGTCCTTTCAAATTCCTGTCATTACCCCACTCTTGGCTATATG 41166
Domestic       CTGTCATTTAACATTTAGGTCCTTTCAAATTCCTGTCATTACCCCACTCTTGGCTATATG 41591
               ************************************************************

Dromedary      AACTAGTACTGTTATATACATAGCAAAATAACTATGGGCGAGTAAAAGCAATTAACAGAA 41453
Wild           AACTAGTACTGTTATATACATAGCAAAATAACTATGGGCGAGTAAAAGCAATTAACAGAA 41226
Domestic       AACTAGTACTGTTATATACATAGCAAAATAACTATGGGCGAGTAAAAGCAATTAACAGAA 41651
               ************************************************************

Dromedary      TTCTAGAATTCTCAGGGATGGGTGGGTTCTAGAGGAGGCAATTTTTTTTAGGTGTTCATA 41513
Wild           TTCTAGAATTCTCAGGGATGGGTGGGTTCTAGAGGAGGCAATTTTTTTTAGGTGTTCATA 41286
Domestic       TTCTAGAATTCTCAGGGATGGGTGGGTTCTAGAGGAGGCAATTTTTTTTAGGTGTTCATA 41711
               ************************************************************

Dromedary      GATTAAAAAAAAGAAAACCCTTCAGAGTAGGACTGAATTTTGTGCGCCTTGTTTTTAATA 41573
Wild           GATTAAAAAAAAGAAAACCCTTCAGAGTAGGACTGAATTTTGTGCGCCTTGTTTTTAATA 41346
Domestic       GATTAAAAAAAAGAAAACCCTTCAGAGTAGGACTGAATTTTGTGCGCCTTGTTTTTAATA 41771
               ************************************************************

Dromedary      TAACCTCATATATTTAAAGAAGTGTACACTATTTCAAATTCATTACAATTCTAGGAGATG 41633
Wild           TAACCTCATATATTTAAAGAAGTGTACACTATTTTAAATTCATTACAATTCTAGGAGATG 41406
Domestic       TAACCTCATATATTTAAAGAAGTGTACGCTATTTTAAATTCATTACAATTCTAGGAGATG 41831
               *************************** ****** *************************

Dromedary      AAAACATCTAACATAGCTCATTAAAGTAAAATAAATTCTCCTGAAGGACGTCCAAGTCAA 41693
Wild           AAAACATCTAACATAGCTCATTAAAGTAAAATAAATTCTCCTGAAGGACGTCCAAGTCAA 41466
Domestic       AAAACATCTAACATAGCTCATTAAAGTAAAATAAATTCTCCTGAAGGACGTCCAAGTCAA 41891
               ************************************************************

Dromedary      TAGAGGTAAATGATCTCCTGTGACAGAAAAAATAAGTAAATAAAGAACATGAAAAAAAAA 41753
Wild           TAGAGGTAAATGATCTCCTGTGACAGAAAAAATAAGTAAATAAAGAACATGAAAAAAAAA 41526
Domestic       TAGAGGTAAATGATCTCCTGTGACAGAAAAAATAAGTAAATAAAGAACATGAAAAAAAAA 41951
               ************************************************************

Dromedary      CAAAAACAGAACAAGAAAATACTTGCAGTGGGCTTTCTAAGTACATCTGAAGATCAATTC 41813
Wild           CAAAAACAGAACAAGAAAATACTTGCAGTGGGCTTTCTAAGTACATCTGAAGATCAATTC 41586
Domestic       CAAAAACAGAACAAGAAAATACTTGCAGTGGGCTTTCTAAGTACATCTGAAGATCAATTC 42011
               ************************************************************

Dromedary      AGATAACACCTGGGTGCAGAGAGGGTCTGTATGGTCTCAAACAGAAAATCAAGGCAAAGG 41873
Wild           AGATAACACCTGGGTGCAGAGAGGGTCTGTATGGTCTCAAACAGAAAATCAAGGCAAAGG 41646
Domestic       AGATAACACCTGGGTGCAGAGAGGGTCTGTATGGTTTCAAACAGAAAATCAAGGCAAAGG 42071
               *********************************** ************************

Dromedary      GCTGATGTGATGTGCAAAATCAACTATGAGAGATTCAGGCTTAGGGCTGACGGGACAATA 41933
Wild           GCTGATGTGATGTGCAAAATCAACTATGAGAGATTCAGGCTTAGGGCTGACGGGACAATA 41706
Domestic       GCTGATGTGATGTGCAAAATCAACTATGAGAGATTCAGGCTTAGGGCTGACGGGACAATA 42131
               ************************************************************

Dromedary      GTGACCCCTGCTGGAGCCTATC-----TTNNNNNNNNNNNNNNNNNNNNNNNTCAAGAAC 41988
Wild           GTGACCCCTGCTGGAGCCTATCGTTTTGTTTTT-----TTTTTTTCCCAAACTCAAGAAC 41761
Domestic       GTGACCCCTGCTGGAGCCTATCGTTTTTTTTTTNNNNNNNTTTTCCCCAAACTCAAGAAC 42191
               **********************      *                       ********
```

```
Dromedary      CTAAGTTGGGGCCTTTGTAAGGATACTTATAACTTCCAATTCCAAGAATAATTGAGTTTG  42048
Wild           CTAAGTTGGGGCCTTTGTAAGGATACTTATAACTTCCAATTCCAAGAATAATTGAGTTTG  41821
Domestic       CTAAGTTGGGGCCTTTGTAAGGATACTTATAACTTCCAATTCCAAGAATAATTGAGTTTG  42251
               ************************************************************

Dromedary      AAAGTTGGGAGAAGGCTTGGAGAACATGGAGCATTCTCTCTTTACTTCATAACTGATGAA  42108
Wild           AAAGTTGGGAGAAGGCTTGGAGAACATGGAGCATTCTCTCTTTACTTCATAACTGATGAA  41881
Domestic       AAAGTTGGGAGAAGGCTTGGAGAACATGGAGCATTCTCTCTTTACTTCATAACTGATGAA  42311
               ************************************************************

Dromedary      ACAGGCTGTTAGCCCACAGAGTTTAAATGACTTGCTTTAGGTGGCAGAATGGGGATTACC  42168
Wild           ACAGGCTGTTAGCCCACAGAGTTTAAATGACTTGCTTTAGGTGGCAGAATGGGGATTACC  41941
Domestic       ACAGGCTGTTAGCCCACAGAGTTTAAATGACTTGCTTTAGGTGGCAGAATGGGGATTACC  42371
               ************************************************************

Dromedary      AGGAAATAAAGACGCCTGCCCAGTTCTTGGGCCTGTTAAAACAGGTGGAGTTTTCTGTCG  42228
Wild           AGGAAATAAAGACGCCTGCCCAGTTCTTGGGCCTGTTAAAACAGGTGGAGTTTTCTGTCG  42001
Domestic       AGGAAATAAAGACGCCTGCCCAGTTCTTGGGCCTGTTAAAACAGGTGGAGTTTTCTGTCG  42431
               ************************************************************

Dromedary      CAACAAATAAAAGTACCTATAATACACACAATAAGGAGGCCAAGCAAAATCTCAAACTGA  42288
Wild           CAACAAATAAAAGTACCTATAATACACACAATAAGGAGGCCAAGCAAAATCTCAAACTGA  42061
Domestic       CAACAAATAAAAGTACCTATAATACACACAATAAGGAGGCCAAGCAAAATCTCAAACTGA  42491
               ************************************************************

Dromedary      TTTTAAAATAAACTGTTCACTTAATTTGGTTAACTAGGACTCAGTTTTGCTGAAAAAAGA  42348
Wild           TTTTAAAATAAACTGTTCCCTTAATTTGGTTAACTAGGACTCAGTTTTGCTGAAAAAAGA  42121
Domestic       TTTTAAAATAAACTGTTCCCTTAATTTGGTTAACTAGGACTCAGTTTTGCTGAAAAAAGA  42551
               ****************** *****************************************

Dromedary      GATTTCTTTAAAAAAATTTTTTTCCACTTTTTAAAATGGAAGTACTGGGGGTTGAACGCA  42408
Wild           GATTTCTTTAAAAAAATTTTTTTCCACTTTTTAAAATGGAAGTACTGGGGGTTGAATGCA  42181
Domestic       GATTTTTTTAAAAAAATTTTTTTCCACTTTTTAAAATGGAAGTACTGGGGGTTGAACGCA  42611
               ***** ************************************************** ***

Dromedary      AGACCTCACGCAGGCTCAGCATGCGCTCTACCGCTGAGCTATCTGCAAACTCCTCTCCTG  42468
Wild           AGACCTCACGCAGGCTCAGCATGCGCTCTACCGCTGAACTATCTGCAAACTCCTCTCCTG  42241
Domestic       AGACCTCACGCAGGCTCAGCATGCGCTCTACCGCTGAGCTATCTGCAAACTCCTCTCCTG  42671
               ************************************* **********************

Dromedary      CCCAGGACAAATTTCCAGTCCATACTAACAAGCCATTTTTTCCCTTTCCTCTTTGGCCGT  42528
Wild           CCCAGGACAAATTTCCAGTCCATACTAACAAGCCATTTTTTCCCTTTCCTCTTTGGCCGT  42301
Domestic       CCCAGGACAAATTTCCAGTCCATACTAACAAGCCATTTTTTCCCTTTCCTCTTTGGCCGT  42731
               ************************************************************

Dromedary      TAAAAGCTTAAACAACAGCATGTTAGAATCAAGTTTTTTTCTGGAATGCTGAGCTGGCCT  42588
Wild           TATAAGCTTAAACAACAGCATGTTAGAATCAAGTTTATTTCTGGAATGCTGAGCTGGCCT  42361
Domestic       TATAAGCTTAAACAACAGCATGTTAGAATCAAGTTTATTTCCGGAATGCTGAGCTGGCCT  42791
               ** ********************************* **** ******************

Dromedary      GTCCTTCACTAGGTTCCCCATGTTCCCCACCTCTGCTATTTTGATCTGTTTGAAATAGTT  42648
Wild           GTCCTTCACTAGGTTCCCCATGTTCCCCACCTCTGCTATTTTGATCTGTTTGAAATAGTT  42421
Domestic       GTCCTTCACTAGGTTCCCCATGTTCCCCACCTCTGCTATTTTGATCTGTTTGAAATAGTT  42851
               ************************************************************

Dromedary      CTTTGGGCTTGAATATGCTTTTATGTCCTTCCCATTGTTGGCCTGTAAAGATCTCTTGAT  42708
Wild           CTTTGGGCTTGAATATGCTTTTATGTCCTTCCCATTGTTGGCCTGTAAAGATCTCTTGAT  42481
Domestic       CTTTGGGCTTGAATATGCTTTTATGTCCTTCCCATTGTTGGCCTGTAAAGATCTCTTGAT  42911
               ************************************************************

Dromedary      GTTGTACTAAGATAAAGGACAAAAATGAAATAACACATACAGGAATGCTTCTTATACTGG  42768
Wild           GTTGTACTAAGATAAAGGACAAAAATGAAATAACACATACAGGAATGCTTCTTATACTGG  42541
Domestic       GTTGTACTAAGATAAAGGACAAAAATGAAATAACACATACAGGAATGCTTCTTATACTGG  42971
               ************************************************************

Dromedary      GATTTTCTGGAAAACCTGACTCTGACTCTTACCCTCTGCTCAGTGCACTGGAGTCACCCA  42828
Wild           GATTTTCTGGAAAACCTGACTCTGACTCTTACCCTCTGCTCAGTGCACTGGAGTCACCCA  42601
Domestic       GATTTTCTGGAAAACCTGACTCTGACTCTTACCCTCTGCTCAGTGCACTGGAGTCACCCA  43031
               ************************************************************
```

```
Dromedary      TTTCAAGTGCTCTGTCTCTTCATCCTTCTTTCCTCAATCAACATCTCTCTCCACTCCATA  42888
Wild           TTTCAAGTGCTCTGTCTCTTCATCCTTCTTTCCTCAATCAACATCTCTCTCCACTCCATA  42661
Domestic       TTTCAAGTGCTCTGTCTCTTCATCCTTCTTTCCTCAATCAACATCTCTCTCCACTCCATA  43091
               ************************************************************

Dromedary      CATCAGAGTTCCCAAGACGCATCACGACCATGGGGTTCGTGCATTCATTCTCCTGTCACT  42948
Wild           CATCAGAGTTCCCAAGACGCATCACGACCATGGGGTTCGTGCATTCATTCTCCTGTCACT  42721
Domestic       CATCAGAGTTCCCAAGACGCATCACGACCATGGGGTTCGTGCATTCATTCTCCTGTCACT  43151
               ************************************************************

Dromedary      GTACTAGAAGGCAAGATCCTGGTCCCTATTGATATTCCTAGGTAGTTATGGAGACGTTGG  43008
Wild           GTACTAGAAGGCAAGATCCTGGTCCCTATTGATATTCCTAGGTAGTTATGGAGACGTTGG  42781
Domestic       GTACTAGAAGGCAAGATCCTGGTCCCTATTGATATTCCTAGGTAGTTATGGAGACGTTGG  43211
               ************************************************************

Dromedary      CAAAGTCACTTTTCTCCTTCCACATTTCAGTTCTGTGATCTTTAAATAAGTACAATATTG  43068
Wild           CAAAGTCACTTTTCTCCTTCCACATTTCAGTTCTGTGATCTTTAAATAAGTACAATATTG  42841
Domestic       CAAAGTCACTTTTCTCCTTCCACATTTCAGTTCTGTGATCTTTAAATAAGTACAATATTG  43271
               ************************************************************

Dromedary      GCTACCCTTCACAGTGTTTCAGTGAGGTTCAAATAAGATGTAATATTTGAAAGAGCTTTG  43128
Wild           GCTACCCTTCACAGTGTTTCAGTGAGGTTCAAATAAGATGTAATATTTGAAAGAGCTTTG  42901
Domestic       GCTACCCTTCACAGTGTTTCAGTGAGGTTCAAATAAGATGTAATATTTGAAAGAGCTTTG  43331
               ************************************************************

Dromedary      AAAAATAGAAAAAAGTAAGTATGGGACAGTTTTTGAGATACTGTTTTTATTATTTTCTTT  43188
Wild           AAAAATAGAAAAAAGTAAGTATGGGACAGTTTTTGAGATACTGTTTTTATTATTTTCTTT  42961
Domestic       AAAAATAGAAAAAAGTAAGTATGGGACAGTTTTTGAGATACTGTTTTTATTATTTTCTTT  43391
               ************************************************************

Dromedary      GTAATTTTTTTTCTCTCCAACTGGTCAAGCAGGTAGCCAGATTTTTTGTTGATTTATTTG  43248
Wild           GTAATTTTTTTTCTCTCCAACTGGTCAAGCAGGTAGCCAGATTTTTTGTTGATTTATTTG  43021
Domestic       GTAATTTTTTTTCTCTCCAACTGGTCAAGCAGGTAGCCAGATTTTTTGTTGATTTATTTG  43451
               ************************************************************

Dromedary      TTTGTGGAATTTTTTGAATTAAGCTATCTGAGGTTTGAAAGTGCCTCATCTTTTACAGAA  43308
Wild           TTTGTGGAATTTTTTGAATTAAGCTATCTGAGGTTTGAAAGTGCCTCATCTTTTACAGAA  43081
Domestic       TTTGTGGAATTTTTTGAATTAAGCTATCTGAGGTTTGAAAGTGCCTCATCTTTTACAGAA  43511
               ************************************************************

Dromedary      AAGATTATGCTCGGAGCTCCAGAGAAGGAAGGAAGAGGAGAGTTTTAAGGAATGAGAACA  43368
Wild           AAGATTATGCTCGGAGCTCCAGAGAAGGAAGGAAGAGGAGAGTTTTAAGGAATGAGAACA  43141
Domestic       AAGATTATGCTCGGAGCTCCAGAGAAGGAAGGAAGAGGAGAGTTTTAAGGAATGAGAACA  43571
               ************************************************************

Dromedary      AGACAGAAGTTCGGCTCATTCAGACTAGAAAGGTTACAGAGGTTTTTGCTTTCACACTGG  43428
Wild           AGACAGAAGTTCGGCTCATTCAGACTAGAAAGGTTACAGAGGTTTTTGCTTTCACACTGG  43201
Domestic       AGACAGAAGTTCGGCTCATTCAGACTAGAAAGGTTACAGAGGTTTTTGCTTTCACACTGG  43631
               ************************************************************

Dromedary      AGAGCCTGCTCTGAGGATGCCCCTGAGGATGGTGGCAAGACCTCAGGTTAGAAGACTTCA  43488
Wild           AGAGCCTGCTCTGAGGATGCCCCTGAGGACGGTGGCAAGACCTCAGGTTAGAAGACTTCA  43261
Domestic       AGAGCCTGCTCTGAGGATGCCCCTGAGGACGGTGGCAAGACCTCAGGTTAGAAGACTTCA  43691
               ***************************** ******************************

Dromedary      TGACGTCGATATTAAAAGCTGACTGTCCACTTTGCATGGCAGAGAGATAGACAAAAGTAC  43548
Wild           TGACGTCGATATTAAAAGCTGACTGTCCACTTTGCATGGCAGAGAGATAGACAAAAGTAC  43321
Domestic       TGACGTCGATATTAAAAGCTGACTGTCCACTTTGCATGGCAGAGAGATAGACAAAAGTAC  43751
               ************************************************************

Dromedary      AGATCTACATCGATTCATGGGCAAGTGCTATTTGGCTAAGGAGTCGGGGCTTGAAGAAAC  43608
Wild           AGATCTACATCGATTCATGGGCAAGTGCTATTTGGCTAAGGAGTCGGGGCTTGAAGAAAC  43381
Domestic       AGATCTACATCGATTCATGGGCAAGTGCTATTTGGCTAAGGAGTCGGGGCTTGAAGAAAC  43811
               ************************************************************

Dromedary      AGGATTAGGAGATGGGTTAAAAGGAGGTCTGAGAAAGAGTTATATGGATGGGCCTCTCAG  43668
Wild           AGGATTAGGAGATGGGTTAAAAGGAGGTCTGAGAAAGAGTTATATGGATGGGCCTCTCAG  43441
Domestic       AGGATTAGGAGATGGGTTAAAAGGAGGTCTGAGAAAGAGTTATATGGATGGGCCTCTCAG  43871
               ************************************************************
```

```
Dromedary      AGAGGACAGAATGAGATTATTTGTGTTCTATATAGAAGCTTACTAAAAGGTATCCACTGG  43728
Wild           AGAGGACAGAATGAGATTATTTGTGTTCTATATAGAAGCTTACTAAAAGGTATCCACTGG  43501
Domestic       AGGGGACAGAATGAGATTATTTGTGTTCTATATAGAAGCTTACTAAAAGGTATCCACTGG  43931
               ** *********************************************************

Dromedary      AAAAGATTGTCAATAGTCAAGTAAACACTATGACCACTGTGAATGTCACTTAGCTGTCTT  43788
Wild           AAAAGATTGTCAATAGTCAAGTAAACACTATGACCACTGTGAATGTCACTTAGCTGTCTT  43561
Domestic       AAAAGATTGTCAATAGTCAAGTAAACACTATGACCACTGTGAATGTCACTTAGCTGTCTT  43991
               ************************************************************

Dromedary      CCCCAGCCACCCTGGTGCTTGACAGTGGGCCTGGCAAGAATGTGGGACTGGGAACATAGG  43848
Wild           CCCCAGCCACCCTGGTGCTTGACAGTGGGCCTGGCAAGAATGTGGGACTGGGAACATAGG  43621
Domestic       CCCCAGCCACCCTGGTGCTTGACAGTGGGCCTGGCAAGAATGTGGGACTGGGAACATAGG  44051
               ************************************************************

Dromedary      TTCTCTTCACCAAGGCTGATCTGGCTACTTTCCCTGATGAGTGCCTCTCTCATCAACATC  43908
Wild           TTCTCTTCACCAAGGCTGATCTGGCTACTTTCCCTGATGAGTGCCTCTCTCATCAACATC  43681
Domestic       TTCTCTTCACCAAGGCTGATCTGGCTACTTTCCCTGATGAGTGCCTCTCTCATCAACATC  44111
               ************************************************************

Dromedary      AGATATCCACAATGAGCTCCTAAAGTGGCATCAATTCCAGGACTTGCCAGTCACCTGATG  43968
Wild           AGATATCCACAATGAGCTCCTAAAGTGGCATCAATTCCAGGACTTGCCAGTCACCTGATG  43741
Domestic       AGATATCCACAATGAGCTCCTAAAGTGGCATCAATTCCAGGACTTGCCAGTCACCTGATG  44171
               ************************************************************

Dromedary      GCAGATGGATTACACTGAACCTCTGTCATCATGGGGGAAAGACACTTAACCTGGATTTGG  44028
Wild           GCAGATGGATTACACTGAACCTCTGTCGTCATGGGGGAAAGACACTTAACCTGGATTTGG  43801
Domestic       GCAGATGGATTACACTGAACCTCTGTCGTCATGGGGGAAAGACACTTAACCTGGATTTGG  44231
               *************************** ********************************

Dromedary      ATTTGCCTTCTTTGCCTATGATGTTTCTGCTAGCACCACTATCCATAGACTTAGGGACTG  44088
Wild           ATTTGCCTTCTTTGCCTATGATGTTTCTGCTAGCACCACTATCCATAGACTTAGGGACTG  43861
Domestic       ATTTGCCTTCTTTGCCTATGATGTTTCTGCTAGCACCACTATCCATAGACTTAGGGACTG  44291
               ************************************************************

Dromedary      CTTCATTCACTCTTCTGGAATCCCATAAAATATTGCTATAGATGAAGGAATTCCATTAAA  44148
Wild           CTTCATTCACTCTTCTGGAATCCCATAAAATATTGCTATAGATGAAGGAATTCCATTAAA  43921
Domestic       CTTCATTCACTCTTCTGGAATCCCATAAAATATTGCTATAGATGAAGGAATTCCATTAAA  44351
               ************************************************************

Dromedary      AGAATAAGCATTGAAGACATGGTTTCATGTCCTTAGGAAGAAGCTGGCTTGATAGAATGA  44208
Wild           AGAATAAGCATTGAAGACATGGTTTCATGTCCTTAGGAAGAAGCTGGCTTGATAGAATGA  43981
Domestic       AGAATAAGCATTGAAGACATGGTTTCATGTCCTTAGGAAGAAGCTGGCTTGATAGAATGA  44411
               ************************************************************

Dromedary      TGGAATGAACTATTGACAACTCACTTATGTGGCCACTTGTGGGACGGGGGAGCTGTCCCA  44268
Wild           TGGAATGAACTATTGACAACTCACTTATGTGGCCACTTGTGGGACGGGGGAGCTGTCCCA  44041
Domestic       TGGAATGAACTATTGACAACTCACTTATGTGGCCACTTGTGGGACGGGGGAGCTGTCCCA  44471
               ************************************************************

Dromedary      CTAGTTATACTGTATTCTCTGAACCAGCACCAATACATAATGCTGTTTCTCCCACAGCCA  44328
Wild           CTAGTTATACTGTATTCTCTGAACCAGCACCAATACATAATGCTGTTTCTCCCACAGCCA  44101
Domestic       CTAGTTATACTGTATTCTCTGAACCAGCACCAATACATAATGCTGTTTCTCCCACAGCCA  44531
               ************************************************************

Dromedary      GAACACTTAGATCCAGGAACGAAAATGCTCAAGTGGTGGTGGTCTCTTCATTATAATTGC  44388
Wild           GAACACTTAGATCCAGGAACGAAAATGCTCAAGTGGTGGTGGTCTCTTCATTATAATTGC  44161
Domestic       GAACACTTAGATCCAGGAACGAAAATGCTCAAGTGGTGGTGGTCTCTTCATTATAATTGC  44591
               ************************************************************

Dromedary      TAATGACCTACTCACATACTTTTATTTCTAGCCCCACAATGTTATGCTCTTAGTCCCCAA  44448
Wild           TAATGACCTACTCACATACTTTTATTTCTAGCCCCACAATGTTATGCTCTTAGTCCCCAA  44221
Domestic       TAATGACCTACTCACATACTTTTATTTCTAGCCCCACAATGTTATGCTCTTAGTCCCCAA  44651
               ************************************************************

Dromedary      GGGAGGAACACTTTCTCTAGGAAACACCGGAAGGGTTCCACTGAATGGAGTGGAGACTGC  44508
Wild           GGGAGGAACACTTTCTCTAGGAAACACCGGAAGGGTTCCACTGAATGGAGTGGAGACTGC  44281
Domestic       GGGAGGAACACTTTCTCTAGGAAACACCGGAAGGGTTCCACTGAATGGAGTGGAGACTGC  44711
               ************************************************************
```

```
Dromedary      CAAATCAGCAATGTCAATTCTTCATGCCACTGAACCAGTAAGCAAAGAAGGGACTCACTG  44568
Wild           CAAATCAGCAATGTCAATTCTTCATGCCACTGAACCAGTAAGCAAAGAAGGGACTCACTG  44341
Domestic       CAAATCAGCAATGTCAATTCTTCATGCCACTGAACCAGTAAGCAAAGAAGGGACTCACTG  44771
               ************************************************************

Dromedary      TATTGGCTGGGGCAGTTGATCCTAATTATAAAATAGAAAAGGTCAGTATACACAGCAAAG  44628
Wild           TATTGGCTGGGGCAGTTGATCCTAATTATAAAATAGAAAAGGTCAGTATACACAGCAAAG  44401
Domestic       TATTGGCTGGGGCAGTTGATCCTAATTATAAAATAGAAAAGGTCAGTATACACAGCAAAG  44831
               ************************************************************

Dromedary      GGGAGGAGGACTATGTCTGAAAGCCTGGGGATTTTCTGGGGAACTCTGAGTACTCCCATG  44688
Wild           GGGAGGAGGACTATGTCTGAAAGCCTGGGGATTTTCTGGGGAACTCTGAGTACTCCCATG  44461
Domestic       GGGAGGAGGACTATGTCTGAAAGCCTGGGGATTTTCTGGGGAACTCTGAGTACTCCCATG  44891
               ************************************************************

Dromedary      TCCATGGTAAAATCTATTGGGAAATTAGAACAACCCAAGCCAAGCAAGAACCACTAATGG  44748
Wild           TCCATGGTAAAATCTATTGGGAAATTAGAACAACCCAAGCCAAGCAAGAACCACTAATGG  44521
Domestic       TCCATGGTAAAATCTATTGGGAAATTAGAACAACCCAAGCCAAGCAAGAACCACTAATGG  44951
               ************************************************************

Dromedary      TTCAGCTTTTTCAGGAATGAAAGTTTAACTAATAAATAATCACAACCAGTGAAACTATTA  44808
Wild           TTCAGCTTTTTCAGGAATGAAAGTTTAACTAATAAATAATCACAACCAGTGAAACTATTA  44581
Domestic       TTCAGCTTTTTCAGGAATGAAAGTTTAACTAATAAATAATCACAACCAGTGAAACTATTA  45011
               ************************************************************

Dromedary      TCTGAAGACGCAGGGAGCAAAGGAAGGTTAGTAGAAGAAGTGAGGTGCAAATACCAACTA  44868
Wild           TCTGAAGACGCAGGGAGCAAAGGAAGGTTAGTAGAAGAAGTGAGGTGCAAATACCAACTA  44641
Domestic       TCTGAAGACGCAGGGAGCAAAGGAAGGTTAGTAGAAGAAGTGAGGTGCAAATACCAACTA  45071
               ************************************************************

Dromedary      TGGCCCATAAACCTTGTAGAAATGAGAACTGTAATCATCATGCATAGTTACCGATTTTTA  44928
Wild           TGGCCCATAAACCTTGTAGAAATGAGAACTGTAATCATCATGCATAGTTACCGATTTTTA  44701
Domestic       TGGCCCATAAACCTTGTAGAAATGAGAACTGTAATCATCATGCATAGTTACCGATTTTTA  45131
               ************************************************************

Dromedary      AATTTTCCTTTTCATCCCTGAGGTCTTCAGGATCTGAATCTAATTATGGAATAGGCCTGG  44988
Wild           AATTTTCCTTTTCATCCCTGAGGTCTTCAGGATCTGAATCTAATTATGGAATAGGCCTGG  44761
Domestic       AATTTTCCTTTTCATCCCTGAGGTCTTCAGGATCTGAATCTAATTATGGAATAGGCCTGG  45191
               ************************************************************

Dromedary      GTCTGGAAGCAATGAGACCCAGTGGGTTTGACATTGAGGAGAGTCCCTATCTCTATTCAG  45048
Wild           GTCTGGAAGCAATGAGACCCAGTGGGTTTGACCTTGAGGAGAGTCCTTATCTCTATTCAG  44821
Domestic       GTCTGGAAGCAATGAGACCCAGTGGGTTTGACCTTGAGGAGAGTCCTTATCTCTATTCAG  45251
               ******************************** ************* *************

Dromedary      GAATCTGAATCAGATTCCTCTGATAAGTCAACTGCCTCAGGTTAATATTAAAACAGGTTA  45108
Wild           GAATCTGAATCAGATTCCTCTGATAAGTCAACTGCCTCAGGTTAATATTAAAACAGGTTA  44881
Domestic       GAATCTGAATCAGATTCCTCTGATAAGTCAACTGCCTCAGGTTAATATTAAAACAGGTTA  45311
               ************************************************************

Dromedary      CCAGGCAGAGAGAGAGACAAAGATTGTCAGACAGAGGAGGGAGGTTTGTTTCTATTGACAAC  45168
Wild           CCAGGCAGAGAGAGAGACAAAGATTGTCAGACAGAGGAGGGAGGTTTGTTTCTATTGACAAC  44941
Domestic       CCAGGCAGAGAGAGAGACAAAGATTGTCAGACAGAGGAGGGAGGTTTGTTTCTATTGACAAC  45371
               ************************************************************

Dromedary      TGACTTAGTGGGATTTGGGGATTCAAGATAGCTGGATTCACTGGAACCTATCCAAGTGTT  45228
Wild           TGACTTAGTGGGATTTGGGGATTCAAGATAGCTGGATTCACTGGAACCTATCCAAGTGTT  45001
Domestic       TGACTTAGTGGGATTTGGGGATTCAAGATAGCTGGATTCACTGGAACCTATCCAAGTGTT  45431
               ************************************************************

Dromedary      TCCATTCTCAGGATCCCAGTCTCCTAATCAATGCCTAGAAAGCCAGTGCTGTAATGCATA  45288
Wild           TCCATTCTCAGGATCCCAGTCTCCTAATCAATGCCTAGAAAGCCTGTGCTGTAATGCATA  45061
Domestic       TCCATTCTCAGGATCCCAGTCTCCTAATCAATGCCTAGAAAGCCTGTGCTGTAATGCATA  45491
               ******************************************** ***************

Dromedary      AAGCCACAGAATCAAATTTAGGTTTGTTTTTTGGGTGCCTCAGCCCCACTGGTACAAGAA  45348
Wild           AAGCCACAGAATCAAATTTAGGTTTGTTTTTTGGGTGCCTCAGCCCCACTGGTACAAGAA  45121
Domestic       AAGCCACAGAATCAAATTTAGGTTTGTTTTTTGGGTGCCTCAGCCCCACTGGTACAAGAA  45551
               ************************************************************
```

```
Dromedary      ATAAGACATTTTTTTAGGCAGTCAAGGAAATGTTCTGATTCTCCAACAGTTCCCTGAGTT 45408
Wild           ATAAGACATTTTTTTAGGCAGTCAAGGAAATGTTCTGATTCTCCAACAGTTCCCTGAGTT 45181
Domestic       ATAAGACATTTTTTTAGGCAGTCAAGGAAATGTTCTGATTCTCCAACAGTTCCCTGAGTT 45611
               ************************************************************

Dromedary      AAACATCTAAATCTCTGGGTTTGTCATTTTTTTCTGGCAAGCTTTCCAGTGCAGTCAGAA 45468
Wild           AAACATCTAAATCTCTGGGTTTGTCA-TTTTTTCTGGCAAGCTTTCCAGTGCAGTCAGAA 45240
Domestic       AAACATCTAAATCTCTGGGTTTGTCA-TTTTTTCTGGCAAGCTTTCCAGTGCAGTCAGAA 45670
               ************************** *********************************

Dromedary      CCACCCCACATCCCTTATAATCTCCACCATGATACTTTACTAACAGCCACTACATTTCCT 45528
Wild           CCACCCCACATCCCTTATAATCTCCACCATGATACTTTACTAACAGCCACTACATTTCCT 45300
Domestic       CCACCCCACATCCCTTATAATCTCCACCATGATACTTTACTAACAGCCACTACATTTCCT 45730
               ************************************************************

Dromedary      AAATTCTTATCATTGAATGGTACCTGATTCCAGAAAACAATGGGATCATTTAAGGAACAT 45588
Wild           AAATTCTTGTCATTGAATGGTACCTGATTCCAGAAAACAATGGGATCATTTAAGGAACAT 45360
Domestic       AAATTCTTGTCATTGAATGGTACCTGATTCCAGAAAACAATGGGATCATTTAAGGAACAT 45790
               ******** ***************************************************

Dromedary      TTTGTCACTGCATGGTATGGACTACCAGTGTCCTGGTTTCCACTGACAGTAAGACCACTA 45648
Wild           TTTGTCACTGCATGGTATGGACTACCAGTGTCCTGGTTTCCACTGACAGTAAGACCACTA 45420
Domestic       TTTGTCACTGCATGGTATGGACTACCAGTGTCCTGGTTTCCACTGACAGTAAGACCACTA 45850
               ************************************************************

Dromedary      CTGATTTCAAATCTAATCTGTGTTTATGCTCATGTTCACTACGTGCTCATATCATATGCT 45708
Wild           CTGATTTCAAATCTAATCTGTGTTTATGCTCATGTTCAGTACGTGCTCATATCATATGCT 45480
Domestic       CTGATTTCAAATCTAATCTGTGTTTATGCTCATGTTCAGTACGTGCTCATATCATATGCT 45910
               ************************************** *********************

Dromedary      AAGTTGCTTGGGAGGAAAGGGGATACAAATGAAGGAAACAAATGGGCCCTGACCTCAAGT 45768
Wild           AAGTTGCTTGGGAGGAAAGGGGATACAAATGAAGGAAACAAATGGGCCCTGACCTCAAGT 45540
Domestic       AAGTTGCTTGGGAGGAAAGGGGATACAAATGAAGGAAACAAATGGGCCCTGACCTCAAGT 45970
               ************************************************************

Dromedary      ATGTTGTGATCTTATAATCAGTAGCTAAGAGAGTTCTGCTGAACTCAGAGAGCAAACAGC 45828
Wild           ATGTTGTGATCTTATAATCAGTAGCTAAGAGAGTTCTGCTGAACTCAGAGAGCAAACAGC 45600
Domestic       ATGTTGTGATCTTATAATCAGTAGCTAAGAGAGTTCTGCTGAACTCAGAGAGCAAACAGC 46030
               ************************************************************

Dromedary      ATTCTCTGAGCTGCCTCATTCCCTCTTCACTAGAGGACCAGTGGGCACTTCTATTTGTTA 45888
Wild           ATTCTCTGAGCTGCCTCATTCCCTCTTCACTAGAGGACCAGTGGGCACTTCTATTTGTTA 45660
Domestic       ATTCTCTGAGCTGCCTCATTCCCTCTTCACTAGAGGACCAGTGGGCACTTCTATTTGTTA 46090
               ************************************************************

Dromedary      AGCTGAGATAGAGAGGGAAGTAGTAGTTTCTTGGAGTCCCAAGTAGTGACCCCATATTTC 45948
Wild           AGCTGAGATAGAGAGGGAAGTAGTAGTTTCTTGGAGTCCCAAGTAGTGACCCCATATTTC 45720
Domestic       AGCTGAGATAGAGAGGGAAGTAGTAGTTTCTTGGAGTCCCAAGTAGTGACCCCATATTTC 46150
               ************************************************************

Dromedary      TCACAGAAACAGTGTTATAAATGATGGTTACATTTCCACACTAATTCCTCCAAGCCTATT 46008
Wild           TCACAGAAACAGTGTTATAAATGATGGTTACATTTCCACACTAATTCCTCCAAGCCTATT 45780
Domestic       TCACAGAAACAGTGTTATAAATGATGGTTACATTTCCACACTAATTCCTCCAAGCCTATT 46210
               ************************************************************

Dromedary      ATAGGCCCCCCAAAGTTACTGAAATACAGTGCTATTTGTATATAAAATACAGTATATTTC 46068
Wild           ATAGGCCCCCCAAAGTTACTGAAATACAGTGCTATTTGTATATAAAATACAGTATATTTC 45840
Domestic       ATAGGCCCCCCAAAGTTACTGAAATACAGTGCTATTTGTATATAAAATACAGTATATTTC 46270
               ************************************************************

Dromedary      TGGCAAAATTATGAATAAGCTTTAAGTGAATGACCAGGAAACCTAATTACCATCTAGATA 46128
Wild           TGGCAAAATTATGAATAAGCTTTAAGTGAATGACCAGGAAACCTAATTACCATCTAGATA 45900
Domestic       TGGCAAAATTATGAATAAGCTTTAAGTGAATGACCAGGAAACCTAATTACCATCTAGATA 46330
               ************************************************************

Dromedary      TTGTTTCTATTGTAAAACCTATTTCAGATCTAATATAAGACATTAAAAATATTCTTCCAC 46188
Wild           TTGTTTCTATTGTAAAACCTATTTCAGATCTAATATAAGACATTAAAAATATTCTTCCAC 45960
Domestic       TTGTTTCTATTGTAAAACCTATTTCAGATCTAATATAAGACATTAAAAATATTCTTCCAC 46390
               ************************************************************
```

```
Dromedary      TTTCCCTCCCACCTAAGCACATATACTGTACCCTAGCATCTGTGGCTAAAAGGGAATCCT  46248
Wild           TTTCCCTCCCACCTAAGCACATATACTGTACCCTAGCATCTGTGGCTAAAAGGGAATCCT  46020
Domestic       TTTCCCTCCCACCTAAGCACATATACTGTACCCTAGCATCTGTGGCTAAAAGGGAATCCT  46450
               ************************************************************

Dromedary      CTCTTCTTGGTCACCTCTCAACAACACCATGTGGTCTGGCAGTAAAGGAATCATTCCTGG  46308
Wild           CTCTTCTTGGTCACCTCTCAACAACACCATGTGGTCTGGCAGTAAAGGAATCATTCCTGG  46080
Domestic       CTCTTCTTGGTCACCTCTCAACAACACCATGTGGTCTGGCAGTAAAGGAATCATTCCTGG  46510
               ************************************************************

Dromedary      TGGCTTTGGGAATGTTTGGCAGCAGGGACGGGAGGCAGAAGAGGGTCTTCTCAATGCACA  46368
Wild           TGGCTTTGGGAATGTTTGGCAGCAGGGACGGGAGGCAGAAGAGGGTCTTCTCAATGCACA  46140
Domestic       TGGCTTTGGGAATGTTTGGCAGCAGGGACGGGAGGCAGAAGAGGGTCTTCTCAATGCACA  46570
               ************************************************************

Dromedary      GTGGGCAGGCTGACCCCAATAGTAGGACACCAGAGACAGCATTCACTCCATCTGTTTCAA  46428
Wild           GTGGGCAGGCTGACCCCAATAGTAGGACACCAGAGACAGCATTCACTCCATCTGTTTCAA  46200
Domestic       GTGGGCAGGCTGACCCCAATAGTAGGACACCAGAGACAGCATTCACTCCATCTGTTTCAA  46630
               ************************************************************

Dromedary      GATAGAATCCTCTTCTTAGGGACACCTTTACTTCTTTCAGAATCAATCCTTTACAAACTG  46488
Wild           GATAGAATCCTCTTCTTAGGGACACCTTTACTTCTTTCAGAATCAATCCTTTACAAACTG  46260
Domestic       GATAGAATCCTCTTCTTAGGGACACCTTTACTTCTTTCAGAATCAATCCTTTACAAACTG  46690
               ************************************************************

Dromedary      CTAAATAACTTGCCAAAGGCCACTCAGCAAGAAAGTGTCAAAGCTAAGGTTCAAAGTTAG  46548
Wild           CTAAATAACTTGCCAAAGGCCACTCAGCAAGAAAGTGTCAAAGCTAAGGTTCAAAGTTAG  46320
Domestic       CTAAATAACTTGCCAAAGGCCACTCAGCAAGAAAGTGTCAAAGCTAAGGTTCAAAGTTAG  46750
               ************************************************************

Dromedary      GTCTTTCCTACTCCGAAGCCCATGTACCTTCCTCTATATTCTTCAGTACTTTGTATAAAA  46608
Wild           GTCTTTCCTACTCCAAAGCCCATGTACCTTCCTCTATATTCTTCAGTACTTTGTATAAAA  46380
Domestic       GTCTTTCCTACTCCAAAGCCCATGTACCTTCCTCTATATTCTTCAGTACTTTGTATAAAA  46810
               ************** *********************************************

Dromedary      ATATAATCACTTTTAAAATAGCCCACAGTGCCATTTCCCACCAGTGTATCCTGATAAACG  46668
Wild           ATATAATCACTTTTAAAATAGCCCACAGTGCCATTTCCCACCAGTGTATCCTGATAAACG  46440
Domestic       ATATAATCACTTTTAAAATAGCCCACAGTGCCATTTCCCACCAGTGTATCCTGATAAACG  46870
               ************************************************************

Dromedary      AATGTGCCTTACCTCAAGCTTCCTACTTACTTTTGATAGCTATGGTCTCAAACACATTAA  46728
Wild           AATGTGCCTTACCTCAAGCTTCCTACTTACTTTTGATAGCTATGGTCTCAAACACATTAA  46500
Domestic       AATGTGCCTTACCTCAAGCTTCCTACTTACTTTTGATAGCTATGGTCTCAAACACATTAA  46930
               ************************************************************

Dromedary      AATCACCTCGAAGCTTTAAAATTACCTGAGCTCTACTCCAGATCAATGAAATTAGACTCT  46788
Wild           AATCACCTCGAAGCTTTAAAATTACCTGAGCTCTACTCCAGGTCAATGAAATTAGACTCT  46560
Domestic       AATCACCTCGAAGCTTTAAAATTACCTGAGCTCTACTCCAGGTCAATGAAATTAGACTCT  46990
               ***************************************** ******************

Dromedary      CTGAGGGTAGGG-GTTTTTTTTTAAGTTCCCCATTTGATTCCAGTGCACGTCCAGGGCTGA  46847
Wild           CTGAGGGTAGGGTTTTTTTTTTTAAGTTCCCCATTTGATTCCAGTGCACGTCCAGGGCTGA  46620
Domestic       CTGAGGGTAGGGTTTTTTTTTTTAAGTTCCCCATTTGATTCCAGTGCACGTCCAGGGCTGA  47050
               ************  **********************************************

Dromedary      GAACCACTGGTAATCAAATCTAAGACAAAGGCTTGAAGTTGACAGCTATAATATGCTGCC  46907
Wild           GAACCACTGGTAATCAAATCTAAGACAAAGGCTTGAAGTTGACAGCTATAATATGCTGCC  46680
Domestic       GAACCACTGGTAATCAAATCTAAGACAAAGGCTTGAAGTTGACAGCTATAATATGCTGCC  47110
               ************************************************************

Dromedary      TGTATAAGCAAGGCACTGAGTGTCATATTAAAGAAAATACATACAGATTAAAGAGAATGC  46967
Wild           TGTATAAGCAAGGTACTGAGTGTCATATTAAAGAAAATACATACAGATTAAAGAGAATGC  46740
Domestic       TGTATAAGCAAGGTACTGAGTGTCATATTAAAGAAAATACATACAGATTAAAGAGAATGC  47170
               ************* **********************************************

Dromedary      ATCTGAGCATTTCACAGTGTGTCTGGCATATGGTAGAAACAAAATAAATGGTAGCATTAA  47027
Wild           ATCTGAGCATTTCACAGTGTGTCTGGCATATGGTAGAAACAAAATAAATGGTAGCATTAA  46800
Domestic       ATCTGAGCATTTCACAGTGTGTCTGGCATATGGTAGAAACAAAATAAATGGTAGCATTAA  47230
               ************************************************************
```

```
Dromedary      AACTGGACACGCTATAGTGAAAGGAAAGTGGGGTTGGAGTTGGATAGACCTGATTTCAGA  47087
Wild           AACTGGACATGCTATAGTGAAAGGAAAGTGGGGTTGGAGTTGGATAGACCTGATTTCAGA  46860
Domestic       AACTGGACATGCTATAGTGAAAGGAAAGTGGGGTTGGAGTTGGATAGACCTGATTTCAGA  47290
               ********* **************************************************

Dromedary      TCCCAGTGTTACTATTTTTAGTCTTTCCTAAGTTAAATATGCTGTGGCTTAGTTTCCTAG  47147
Wild           TCCCAGTGTTACTATTTTTAGTCTTTCCTAAGTTAAATATGCTGTGGCTTAGTTTCCTAG  46920
Domestic       TCCCAGTGTTACTATTTTTAGTCTTTCCTAAGTTAAATATGCTGTGGCTTAGTTTCCTAG  47350
               ************************************************************

Dromedary      TTTGTACAATAGTTACAACAAATCCTATCTTATAGAATTGCTGTGAGAATTAAATAAAAT  47207
Wild           TTTGTACAATAGTTACAACAAATCCTATCTTATAGAATTGCTGTGAGAATTAAATAAAAT  46980
Domestic       TTTGTACAATAGTTACAACAAATCCTATCTTATAGAATTGCTGTGAGAATTAAATAAAAT  47410
               ************************************************************

Dromedary      GTTTGTGAAACACCTGGCACATTCCTTCTTTTGTTTGTAACCCACCTATGCTAATAAAAC  47267
Wild           GTTTGTGAAACACCTGGCACATTCCTTCTTTTGTTTGTAACCCACCTATGCTAATAAAAC  47040
Domestic       GTTTGTGAAACACCTGGCACATTCCTTCTTTTGTTTGTAACCCACCTATGCTAATAAAAC  47470
               ************************************************************

Dromedary      AGCCACTGAAAACAGTTACAGAATATTCCTGACATTTAGAAATGTTCTGTTATAGGTGTG  47327
Wild           AGCCACTAAAAACAGTTACAGAATATTCCTGACATTTAGAAATGTTCTGTTATAGGTGTG  47100
Domestic       AGCCACTAAAAACAGTTACAGAATATTCCTGACATTTAGAAATGTTCTGTTATAGGTGTG  47530
               ******* ****************************************************

Dromedary      GAAATGTATAAGTTCTGTATTTAGTATAATAAATTCAGATAACAGTGTAGATTCTATTTA  47387
Wild           GAAATGTATAAGTTCTGTATTTAGTATAATAAATTCAGATAACAGTGTAGATTCTATTTA  47160
Domestic       GAAATGTATAAGTTCTGTATTTAGTATAATAAATTCAGATAACAGTGTAGATTCTATTTA  47590
               ************************************************************

Dromedary      TTCTATACTATTTCCTACTCTACTGTAGAATAAAGCATCAAGATTGGTGACCTGGTATGA  47447
Wild           TTCTATACTATTTCCTACTCTACTGTAGAATAAAGCATCAAGATTGGTGACCTGGTATGA  47220
Domestic       TTCTATACTATTTCCTACTCTACTGTAGAATAAAGCATCAAGATTGGTGACCTGGTATGA  47650
               ************************************************************

Dromedary      TAAAGAGGGTTCTTGAAGAGAGGGCTGGTGGGTAATGAGCTGGGACAGAAATGCAGGCAT  47507
Wild           TAAAGAGGGTTCTTGAAGAGAGGGCTGGTGGGTAGTGAGCTGGGACAGAAATGCAGGCAT  47280
Domestic       TAAAGAGGGTTCTTGAAGAGAGGGCTGGTGGGTAGTGAGCTGGGACAGAAATGCAGGCAT  47710
               ********************************** *************************

Dromedary      GTGGACTCTTCCATCCATGTTATGCTGTTGCCCGGACCTGACCCCAGTGCAGATCAAAGT  47567
Wild           CTGGACTCTTCCATCCATGTTATGCTGTTGCCCGGACCTGACCCCAGTGCAGATCAAAGT  47340
Domestic       CTGGACTCTTCCATCCATGTTATGCTGTTGCCCGGACCTGACCCCAGTGCAGATCAAAGT  47770
                ***********************************************************

Dromedary      GGCTCAGAACACAGTGGGAAGCCAGGTTAGACTGTCACCACTGCCTTCATGAAACTGAGT  47627
Wild           GGCTCAGAACACAGTGGGAAGCCAGGTTAGACTGTCACCACTGCCTTCATGAAACTGAGT  47400
Domestic       GGCTCAGAACACAGTGGGAAGCCAGGTTAGACTGTCACCACTGCCTTCATGAAACTGAGT  47830
               ************************************************************

Dromedary      CTAGAACAGCCATTAGCCGCTTACGTCTCAGTGCTGTCAGTGCCTGATGTAGGGGATTGC  47687
Wild           CTAGAACAGCCATTAGCTGCTTACGTCTCAGTGCTGTCAGTGCCTGATGTAGGGGATTGC  47460
Domestic       CTAGAACAGCCATTAGCTGCTTACGTCTCAGTGCTGTCAGTGCCTGATGTAGGGGATTGC  47890
               ***************** ******************************************

Dromedary      AATCAAGATGGAATGTGTCAGCAGTGTTCAAACAAACATCACTGCTCCATCTTGGGAGCA  47747
Wild           AATCAAGATGGAATGTGTCAGCAGTGTTCAAACAAACATCACTGCTCCATCTTGGGAGCA  47520
Domestic       AATCAAGATGGAATGTGTCAGCAGTGTTCAAACAAACATCACTGCTCCATCTTGGGAGCA  47950
               ************************************************************

Dromedary      AAGCCTATAAATAGTAGATCTGGATATGGCTCACACCAGACCCTTGTGTAATTATCTGTC  47807
Wild           AAGCCTATAAATAGTAGATCTGGATATGGCTCACACCAGACCCTTGTGTAATTATCTGTC  47580
Domestic       AAGCCTATAAATAGTAGATCTGGATATGGCTCACACCAGACCCTTGTGTAATTATCTGTC  48010
               ************************************************************

Dromedary      TTCACAGGATGTGCCTGGCACAAAGCGCTAAGAAATCTGGAACACTGCCTGACATATACA  47867
Wild           TTCACAGGATGTGCCTGGCACAAAGCGCTAAGAAATCTGGAACACTGCCTGACATATACA  47640
Domestic       TTCACAGGATGTGCCTGGCACAAAGCGCTAAGAAATCTGGAACACTGCCTGACATATACA  48070
               ************************************************************
```

```
Dromedary     AGGTACTCAATAAGTGTTCCTTGAATTAAACTGACCTGTGTCCTCTGCCTGGAACACTTT 47927
Wild          AGGTACTCAATAAGTGTTCCTTGAATTAAACTGACCTGTGTCCTCTGCCTGGAACACTTT 47700
Domestic      AGGTACTCAATAAGTGTTCCTTGAATTAAACTGACCTGTGTCCTCTGCCTGGAACACTTT 48130
              ************************************************************

Dromedary     CCTGACTGGTACTAGATGTTAACATTTACTGGGTCTTAACGACTGCCTGGCCCTGTACTA 47987
Wild          CCTGACTGGTACTAGATGTTAACATTTACTGGGTCTTAACGACTGCCTGGCCCTGTACTA 47760
Domestic      CCTGACTGGTACTAGATGTTAACATTTACTGGGTCTTAACGACTGCCTGGCCCTGTACTA 48190
              ************************************************************

Dromedary     GGCACTTTATATAGAGATCATCTCATTCATCTGGCAAACTCCTGCCCATCCTTCAAGATC 48047
Wild          GGCACTTTATATAGAGATCATCTCATTCATCTGGCAAACTCCTGCCCATCCTTCAAGATC 47820
Domestic      GGCACTTTATATAGAGATCATCTCATTCATCTGGCAAACTCCTGCCCATCCTTCAAGATC 48250
              ************************************************************

Dromedary     TAGCTCAAATGTCACCTATTCGGTTAAACCTTCTTGACTCCCCAAGGCAGAGTCCTCCGC 48107
Wild          TAGCTCAAATGTCACCTATTCGGTTAAACCTTCTTGACTCCCCAAGGCAGAGTCCTCTGC 47880
Domestic      TAGCTCAAATGTCACCTATTCGGTTAAACCTTCTTGACTCCCCAAGGCAGAGTCCTCTGC 48310
              ********************************************************* **

Dromedary     GCTTCTGCACTGACAGCTCTTTGCTCAGACTTCAGAAAAGCACTTACCACCTTCTACTTT 48167
Wild          GCTTCTGCACTGACAGCTCTTTGCTCAGACTTCAGAAAAGCACTTACCACCTTCTACTTT 47940
Domestic      GCTTCTGCACTGACAGCTCTTTGCTCAGACTTCAGAAAAGCACTTACCACCTTCTACTTT 48370
              ************************************************************

Dromedary     AACATATCTGTTTACAAGTCTGTCTCCCCCATTGCACTGTGAGGTCATCAGGGACATCAT 48227
Wild          AACATATCTGTTTACAAGTCTGTCTCCCCCATTGCACTGTGAGGTCATCAGGGACATCAT 48000
Domestic      AACATATCTGTTTACAAGTCTGTCTCCCCCATTGCACTGTGAGGTCATCAGGGACATCAT 48430
              ************************************************************

Dromedary     GTCTTTATTACTTTTTTCTTCATTAGTGGTACTTAATGCAGTATCTGATGAAATGAATTT 48287
Wild          GTCTTTATTACTTTTTTCTTCATTAGTGGTACTTAATGCAGTATCTGATGAAATGAATTT 48060
Domestic      GTCTTTATTACTTTTTTCTTCATTAGTGGTACTTAATGCAGTATCTGATGAAATGAATTT 48490
              ************************************************************

Dromedary     TCAAAACTGCTTGCAATTCTGCTGACTAAATGATGTTACACTGAATGCTTTTACTTATAA 48347
Wild          TCAAAACTGCTTGCAATTCTGCTGACTAAATGATGTTACACTGAATGCTTTTACTTATAA 48120
Domestic      TCAAAACTGCTTGCAATTCTGCTGACTAAATGATGTTACACTGAATGCTTTTACTTATAA 48550
              ************************************************************

Dromedary     AACTACATCCTTTTCCACATAGAAGCCAGCTAATATTTTTATGTAACTGAGGAAGGACAA 48407
Wild          AACTACATCCTTTTCCACATAGAAGCCAGCTAATATTTTTATGTAACTGAGGAAGGACAA 48180
Domestic      AACTACATCCTTTTCCACATAGAAGCCAGCTAATATTTTTATGTAACTGAGGAAGGACAA 48610
              ************************************************************

Dromedary     TTTTTATATAACTGAGGAAGAATGAATCCTCTGTCTCAGAATCTGAACCAGTGAAGAGTG 48467
Wild          TTTTTATATAACTGAGGAAGAATGAATCCTGTGTCTCAGAATCTGAACCAGTGAAGAGTG 48240
Domestic      TTTTTATATAACTGAGGAAGAATGAATCCTGTGTCTCAGAATCTGAACCAGTGAAGAGTG 48670
              ****************************** *****************************

Dromedary     GGATATGAGCTCTATCTGCACACACCAACTGTGAGAATCAACATCTCCCGGGAAGTGCCT 48527
Wild          GGATATGAGCTCTATCTGCACACACCAACTGTGAGAATCAACATCTCCCGGGAAGTGCCT 48300
Domestic      GGATATGAGCTCTATCTGCACACACCAACTGTGAGAATCAACATCTCCCAGGAAGTGCCT 48730
              ************************************************* **********

Dromedary     AATACGCTTCCACTAGCACCTACCTTGGGACCAAATTGGTGTTCTCAGGGTATCTGTAGT 48587
Wild          AATACGCTTCCACTAGCACCTACCTTGGGACCAAATTGGTGTTCTCAGAGTATCTGTAGT 48360
Domestic      AATACGCTTCCACTAGCACCTACCTTGGGACCAAATTGGTGTTCTCAGAGTATCTGTAGT 48790
              ************************************************ ***********

Dromedary     TCCTGGTAACTGTCTTCTGGCAGGACCTATGGTCTGCAACAAAGAAAAAGAAACATGAGA 48647
Wild          TCCTGGTAACTGTCTTCTGGCAGGACCTATGGTCTGCAACAAAGAAAAAGAAACATGAGA 48420
Domestic      TCCTGGTAACTGTCTTCTGGCAGGACCTATGGTCTGCAACAAAGAAAAAGAAACATGAGA 48850
              ************************************************************

Dromedary     GGATTTATACATAAATACAAACACACCGCATTTTTCAATAAAGCATTATCACCTGAAATC 48707
Wild          GGATTTATACATAAATACAAACACACCGCATTTTTCAATAAAGCATTATCACCTGAAATC 48480
Domestic      GGATTTATACATAAATACAAACACACCGCATTTTTCAATAAAGCATTATCACCTGAAATC 48910
              ************************************************************
```

```
Dromedary      TTAAACTCTAATTATAAAATGGAGAAATAATACTTGCCTTATATACTTAGTGAGGATTAA  48767
Wild           TTAAACTCTAATTATAAAATGGAGAAATAATACTTGCCTTATATACTTAGTGAGGATTAA  48540
Domestic       TTAAACTCTAATTATAAAATGGAGAAATAATACTTGCCTTATATACTTAGTGAGGATTAA  48970
               ************************************************************

Dromedary      ATGAGATAATGTATATGAAGTGCCTGATATAGTGACTAATTTATTGCAAACT--AAAAAA  48825
Wild           ATGAGATAATGTATATGAAGTGCCTGATATAGTGACTAATTCATTGCAAACTAAAAAAAA  48600
Domestic       ATGAGATAATGTATATGAAGTGCCTGATATAGTGACTAATTTATTGCAAACTAAAAAAAA  49030
               ***************************************** **********  ******

Dromedary      AAAAAAAATGTGGTAGCTATTTTATTATCCCACAACCAGGCAATACTCCACTAAACTTCC  48885
Wild           AAAAAAAATGTGGTAGCTATTTTATTATCCCACAACCAGGCAATACTCCACTAACCTTCC  48660
Domestic       AAAAAAAATGTGGTAGCTATTTTATTATCCCACAACCAGGCAATACTCCACTAACCTTCC  49090
               ****************************************************** *****

Dromedary      AGCTTTCTAACTCCTTTATTTGCAATACATAGTGTGGAGCTGGGAGCTTATTGAGAAAAT  48945
Wild           AGCTTTCTAACTCCTTTATTTGCAATACATAGTGTGGAGCTGGGAGCTTATTGAGAAAAT  48720
Domestic       AGCTTTCTAACTCCTTTATTTGCAATACATAGTGTGGAGCTGGGAGCTTATTGAGAAAAT  49150
               ************************************************************

Dromedary      TTCCTTGACTCCCAGGTGTCGATGTGAGCAAGTGGTTGAATAATGGTGCCATTTAGTGAA  49005
Wild           TTCCTTGACTCCCAGGTGTCGATGTGAGCAAGTGGTTGAATAATGGTGCCATTTAGTGAA  48780
Domestic       TTCCTTGACTCCCAGGTGTCGATGTGAGCAAGTGGTTGAATAATGGTGCCATTTAGTGAA  49210
               ************************************************************

Dromedary      ACGGAGAAGACTGGGTGGGGAGGAGAGGACTGGTGGCAAGTCAAGAGTTCTGTTTTGGCC  49065
Wild           ACGGAGAAGACTGGGTGGGGAGGAGGGGACTGGTGGCAAATCAAGAGTTCTGTTTTGGCC  48840
Domestic       ACGGAGAAGACTGGGTGGGGAGGAGGGGACTGGTGGCAAATCAAGAGTTCTGTTTTGGAC  49270
               ************************* ************* ****************** *

Dromedary      ATGGTATGTTGGAAATTCGTATTAACTACCCCAAAAAGGATGTAAAGTAGACTGCTGAAT  49125
Wild           AAGGTATGTTGGAAATTCGTATTAACTACCCCAAAAAGGATGTAAAGTAGACTGCTGAAT  48900
Domestic       AAGGTATGTTGGAAATTCGTATTAACTACCCCAAAAAGGATGTAAAGTAGACTGCTGAAT  49330
               * **********************************************************

Dromedary      ACGTCTGGAGCAAACAGCAAATATTTAATATTAAAGGTCATAGGACTGGATGAAATATCT  49185
Wild           ACGTCTGGAGCAAACAGCAAATATTTAATATTAAAGGTCATAGGACTGGATGAAATATCT  48960
Domestic       ACGTCTGGAGCAAACAGCAAATATTTAATATTAAAGGTCATAGGACTGGATGAAATATCT  49390
               ************************************************************

Dromedary      CTGGAGAAAGCATACATATAATAAATGTCATTTTTTTCATAGTTATAAAACCTATTCTGT  49245
Wild           CTGGAGAAAGCATACATATAATAAATGTCATTTTTTTCATAGTTATAAAACCTATTCTGT  49020
Domestic       CTGGAGAAAGCATACATATAATAAATGTCATTTTTTTCATAGTTATAAAACCTATTCTGT  49450
               ************************************************************

Dromedary      TCTAAAATTTTTTTCTGCTTAGTATATTATAATCATCTTTCTATGTCAGTATATACTTAC  49305
Wild           TCTAAAATTTTTTTCTGCTTAGTATATTATAATCATCTTTCTATGTCAGTATATACTTAC  49080
Domestic       TCTAAAATTTTTTTCTGCTTAGTATATTATAATCATCTTTCTATGTCAGTATATACTTAC  49510
               ************************************************************

Dromedary      ATTTTGCATGGAGGTAGATAAATAATTGATTTAATTTATTTCTAATTGTTGGACAAGTAG  49365
Wild           A-TTTGCATGGAGGTAGATAAATAATTGATTTAATTTATTTCTAATTGTTGGACAAGTAG  49139
Domestic       A-TTTGCATGGAGGTAGATAAATAATTGATTTAATTTATTTCTAATTGTTGGACAAGTAG  49569
               * **********************************************************

Dromedary      GGTATTTGCAATTTTTCAATGTTATAAATTGTATAAATACTTTTGAGACTTTGTGCAAGT  49425
Wild           GGTATTTGCAATTTTTCAATGTTATAAATTGTATAAATACTTTTGAGACTTTGTGCAAGT  49199
Domestic       GGTATTTGCAATTTTTCAATGTTATAAATTGTATAAATACTTTTGAGACTTTGTGCAAGT  49629
               ************************************************************

Dromedary      TGATCTGTGGAAAAAAATTCTAGAAAATAGAACTTCTGGATCAAAGCGCATAGCATATCTA  49485
Wild           TGATCTGTGGAAAAAAATTCTAGAAAATAGAACTTCTGGATCAAAGCGCATAGCATATCTA  49259
Domestic       TGATCTGTGGAAAAAAATTCTAGAAAATAGAACTTCTGGATCAAAGCGCATAGCATATCTA  49689
               ************************************************************

Dromedary      AAATTTTGAAAGAGAATGCCAAAATGCCCTACAAACTGGATAAAAATTAAAATCTACACT  49545
Wild           AAATTTTGAAAGAGAATGCCAAAATGCCCTACAAACTGGATAAAAATTAAAATCTACACT  49319
Domestic       AAATTTTGAAAGAGAATGCCAAAATGCCCTACAAACTGGATAAAAATTAAAATCTACACT  49749
               ************************************************************
```

```
Dromedary      CCCACCAAGAGGGCAAGAGTGGGCTCTTTTCCAATGTTTATTCTAACACTTGAGTGAGAA  49605
Wild           CCCACCAAGAGGGCAAGAGTGGGCTCTTTTCCAATGTTTATTCTAACACTTGAGTGAGAA  49379
Domestic       CCCACCAAGAGGGCAAGAGTGGGCTCTTTTCCAATGTTTATTCTAACACTTGAGTGAGAA  49809
               ************************************************************

Dromedary      ATGGTATTTCACTGATACTTTAATCTTATTCTTACTTATGAGGGAGGTTGAATATCTTTT  49665
Wild           ATGGTATTTCACTGATACTTTAATGTTATTCTTACTTATGAGGGAGGTTGAATATCTTTT  49439
Domestic       ATGGTATTTCACTGATACTTTAATGTTATTCTTACTTATGAGGGAGGTTGAATATCTTTT  49869
               ************************ ***********************************

Dromedary      TATTTATTGGAAATAGATAAAATTTATTTTTGGTTTATTGGAAATTTGTATTTCTTTTGT  49725
Wild           TATTTATTGGAAATAGATAAAATTTATTTTTGGTTTATTGGAAATTTGTATTTCTTTTGT  49499
Domestic       TATTTATTGGAAATAGATAAAATTTATTTTTGGTTTATTGGAAATTTGTATTTCTTTTGT  49929
               ************************************************************

Dromedary      CATTTTTCTACTGGGTTGTTAACTTTTTTCTTATTCTTAAGGGTTCTGGAAGGTAAGACA  49785
Wild           CATTTTTCTACTGGGTTGTTAACTTTTTTCTTATTCTTAAGGGTTCTGGAAGGTAAGACA  49559
Domestic       CATTTTTCTACTGGGTTGTTAACTTTTTTCTTATTCTTAAGGGTTCTGGAAGGTAAGACA  49989
               ************************************************************

Dromedary      AATTTAGCCTTTTTCCTGTCATAAGCATTTCATTTCCTCATCTTTGTTATGTTTGACTTC  49845
Wild           AATTTAGCCTTTTTCCTGTCATAAGCGTTTCATTTCCTCATCTTTGTTATGTTTGACTTC  49619
Domestic       AATTTAGCCTTTTTCCTGTCATAAGCGTTTCATTTCCTCATCTTTGTTATGTTTGACTTC  50049
               ************************** *********************************

Dromedary      CAAGGAGACTTCTACCTCGGGCTACATTTGATTATCTGGTACTGTACCTGCCTTTCTGCT  49905
Wild           CAAGGAGAATTCTACCTTGGGCTACATTTGATTATCTGGTACTGTACCTGCCTTTCTGCT  49679
Domestic       CAAGGAGAATTCTACCTTGGGCTACATTTGATTATCTGGTACTGTACCTGCCTTTCTGCT  50109
               ******** ******** ******************************************

Dromedary      GTAAACGATTAGAAAACAGGGTAGAATATGCAAAACAACTGCTTCAGAACTGGACACAGA  49965
Wild           GTAAACGATTAGAAAACAGGGTAGAATATGCAAAACAACTGCTTCAGAACTGGACACAGA  49739
Domestic       GTAAACGATTAGAAAACAGGGTAGAATATGCAAAACAACTGCTTCAGAACTGGACACAGA  50169
               ************************************************************

Dromedary      CAGAATAAGACTGTGATCCCTAAGAGAAGGGAAACAAGTCAATGAGCCATAGAACTGCCC  50025
Wild           CAGAATAAGACTGTGATCCCTAAGAGAAGGGAAACAAGTCAATGAGCCATAGAACTGCCC  49799
Domestic       CAGAATAAGACTGTGATCCCTAAGAGAAGGGAAACAAGTCAATGAGCCATAGAACTGCCC  50229
               ************************************************************

Dromedary      TAGAGGCACATTTTGGATCACAGTGCAGAGAAGGGAAAGTCAGGCAGAGCACAATAATCT  50085
Wild           TAGAGGCACATTTTGGATCACAGTGCAGAGAAGGGAAAGTCAGGCAGAGCACAATAATCT  49859
Domestic       TAGAGGCACATTTTGGATCACAGTGCAGAGAAGGGAAAGTCAGGCAGAGCACAATAATCT  50289
               ************************************************************

Dromedary      TGCTAAGTTGAGGAACCAAAGATCAGAAATCTAGGAGGTCAAGGACACTGGGTATTGTGG  50145
Wild           TGCTAAGTTGAGGAACCAAAGATCAGAAATCTAGGAGGTCAAGGACACTGGGTATTGTGG  49919
Domestic       TGCTAAGTTGAGGAACCAAAGATCAGAAATCTAGGAGGTCAAGGACACTGGGTATTGTGG  50349
               ************************************************************

Dromedary      GATAGGGTGCTGGAGAGGACGGAGCTATTTGAAGAAAAACTCCAAAAATCTGTGCAGGGT  50205
Wild           GATAGGGTGCTGGAGAGGACGGAGCTATTTGAAGAAAAACTCCAAAAATCTGTGCAGGGT  49979
Domestic       GATAGGGTGCTGGAGAGGACGGAGCTATTTGAAGAAAAACTCCAAAAATCTGTGCAGGGT  50409
               ************************************************************

Dromedary      TCCCTTGAGTCTGAAATAGGCTGCAAATGAATAGAGTGAAATTTTATGGGCCAGGCAATG  50265
Wild           TCCCTTGAGTCTGAAATAGGCTGCAAATGAATAGAGTGAAATTTTATGGGCCAGGCAATG  50039
Domestic       TCCCTTGAGTCTGAAATAGGCTGCAAATGAATAGAGTGAAATTTTATGGGCCAGGCAATG  50469
               ************************************************************

Dromedary      AACACCGGGGGAACTGTAAGTGGAACACTTCTTCAAGGTCACAATGGGCTCCAAGATCTG  50325
Wild           AACACCGGGGGAACTGTAAGTGGAACACTTCTTCAAGGTCACAATGGGCTCCAAGATCTG  50099
Domestic       AACACCGGGGGAACTGTAAGTGGAACACTTCTTCAAGGTCACAATGGGCTCCAAGATCTG  50529
               ************************************************************

Dromedary      ACCAGCCAGAGTAAAAGACATGTTGAGTATCCAGGGCATTAATTAACCTTAGAAAGGTCA  50385
Wild           ACCAGCCAGAGTAAAAGACATGTTGAGTATCCAGGGCATTAATTAACCTTAGAAAGGTCA  50159
Domestic       ACCAGCCAGAGTAAAAGACATGTTGAGTATCCAGGGCATTAATTAACCTTAGAAAGGTCA  50589
               ************************************************************
```

```
Dromedary      TGTGTTCATAATTAGATTAAACTAGTCCTGGACTAAAGGCTACTGTAGACCCACCCCAGC  50445
Wild           TGTGTTCATAATTAGATTAAACTAGTCCTGGACTAAAGGCTACTGTAGACCCACCCCAGC  50219
Domestic       TGTGTTCATAATTAGATTAAACTAGTCCTGGACTAAAGGCTACTGTAGACCCACCCCAGC  50649
               ************************************************************

Dromedary      AAACTTGAAAGGCAGCTTCAAAAACATCAAGCTGATCTACAAGAAACTATCTGCTTGTCT  50505
Wild           AAACTTGAAAGGCAGCTTCAAAAACATCAAGCTGATCTACAAGAAACTATCTGCTTGTCT  50279
Domestic       AAACTTGAAAGGCAGCTTCAAAAACATCAAGCTGATCTACAAGAAACTATCTGCTTGTCT  50709
               ************************************************************

Dromedary      AAACCAATTTTAATATTCGTTAAAGGAAGACAATGAAATTCAGACACTTTATAACGTAAT  50565
Wild           AAACCAATTTTAATATTCGTTAAAGGAAGACAATGAAATTCAGACACTTTATAACGTAAT  50339
Domestic       AAACCAATTTTAATATTCGTTAAAGGAAGACAATGAAATTCAGACACTTTATAACGTAAT  50769
               ************************************************************

Dromedary      GTTTATAATTCCCTCCACCCAATAAAAAAGTTAGCAGACATATAAATAAACAAGAAAATG  50625
Wild           GTTTATAATTCCCTCCACCCAATAAAAAAGTTAGCAGACATATAAATAAACAAGAAAATG  50399
Domestic       GTTTATAATTCCCTCCACCCAATAAAAAAGTTAGCAGACATATAAATAAACAAGAAAATG  50829
               ************************************************************

Dromedary      TGACTCAAATCAGGGGGAAAAAACAGTCAATGTAAACAGACACACAAAAAGACACTGATG  50685
Wild           TGACTCAAATCAGGGGGAAAAAACAGTCAATGTAAACAGACACACAAAAAGACACTGATG  50459
Domestic       TGACTCAAATCAGGGGGAAAAAACAGTCAATGTAAACAGACACACAAAAAGACACTGATG  50889
               ************************************************************

Dromedary      ATGGAATTTGCAGGCAAGGATTTTAAAAGTCATTAAAAATATGCTCTAAGCTTTAAAAGA  50745
Wild           ATGGAATTTGCAGGCAAGGATTTTAAAAGTCATTAAAAATATGCTCTAAGCTTTAAAAGA  50519
Domestic       ATGGAATTTGCAGGCAAGGATTTTAAAAGTCATTAAAAATATGCTCTAAGCTTTAAAAGA  50949
               ************************************************************

Dromedary      AAACATGAACATAATAAGGAGAGACATGGAAGATATAAAAGAACCAAATGGATTTTCTAG  50805
Wild           AAACATGAACATAATAAGGAGAGACATGGAAGATATAAAAGAACCAAATGGATTTCCTAG  50579
Domestic       AAACATGAACATAATAAGGAGAGACATGGAAGATATAAAAGAACCAAATGGATTTCCTAG  51009
               ******************************************************* ****

Dromedary      GGATGAAGTACAATATCTGGGATGAAAATTTCACTTGATGAGATTAAGAGATGATTAGAT  50865
Wild           GGATGAAGTACAATATCTGGGATGAAAATTTCACTTGATGAGATTAAGAGATGATTAGAT  50639
Domestic       GGATGAAGTACAATATCTGGGATGAAAATTTCACTTGATGAGATTAAGAGATGATTAGAT  51069
               ************************************************************

Dromedary      ACTGCAGAAAAAAAATCAATGAACCAGAAAAATAGGAAATAGCTGTCAAAACTGAAACACA  50925
Wild           ACTGCAGAAAAAAAATCAATGAACCAGAAAAATAGGAAATAGCTGTCAAAACTGAAACACA  50699
Domestic       ACTGCAGAAAAAAAATCAATGAACCAGAAAAATAGGAAATAGCTGTCAAAACTGAAACACA  51129
               ************************************************************

Dromedary      CAGAGATAAAGAGAATGAAAAATATTAATACAGCCTCTGTGACCTGTGGGACAACATCAA  50985
Wild           CAGAGATAAAGAGAATGAAAAATATTAATACAGCCTCTGTGACCTGTGGGACAACATCAA  50759
Domestic       CAGAGATAAAGAGAATGAAAAATATTAATACAGCCTCTGTGACCTGTGGGACAACATCAA  51189
               ************************************************************

Dromedary      ATTGTCTTATTAATATATGTGCAATTACAGTCCCAGAAAGAGAGGAGGAAGAAAAAAATAT  51045
Wild           ATTGTCTTATTAATATATGTGCAATTACAGTCCCAGAAAGAGAGGAGGAAGAAAAAAATAT  50819
Domestic       ATTGTCTTATTAATATATGTGCAATTACAGTCCCAGAAAGAGAGGAGGAAGAAAAAAATAT  51249
               ************************************************************

Dromedary      TTGAATAAATCATAGTTAAACTTTTCTCAAAATTGATGAAAACTATAAACCCCCAAATCA  51105
Wild           TTGAATAAATCATAGTTAAAGTTTTCTCAAAATTGATGAAAACTATAAACCCCCAAATCA  50879
Domestic       TTGAATAAATCATAGTTAAAGTTTTCTCAAAATTGATGAAAACTATAAACCCCCAAATCA  51309
               ******************** ***************************************

Dromedary      AGGGAAGCCAATAAACCACAAGCAGGATTAACACAAAGAAAACCACACCAAGGCACATCA  51165
Wild           AGGGAAGCCAATAAACCACAAGCAGGATTAACACAAAGAAAACCACACCAAGGCACATCA  50939
Domestic       AGGGAAGCCAATAAACCACAAGCAGGATTAACACAAAGAAAACCACACCAAGGCACATCA  51369
               ************************************************************

Dromedary      TCTTAAAATTGCTGAGAACCAATGATAAAGTCTTAAAAGTAGTCAAAGAAAGACATACCA  51225
Wild           TCTTAAAATTGCTGAGAACCAATGATAAAGTCTTAAAAGTAGTCAAAGAAAAACATACCA  50999
Domestic       TCTTAAAATTGCTGAGAACCAATGATAAAGTCTTAAAAGTAGTCAAAGAAAGACATACCA  51429
               *************************************************** ********
```

```
Dromedary     CACATAAGAACAATGGTAAGTATTACTGCTAATGTCCAGAACAATGTAATATAGAGGACA  51285
Wild          CATATAAGAACAATGATAAGTATTACTGCTAATGTCCAGAACAATGTAATATAGAGGACA  51059
Domestic      CATATAAGAACAATGATAAGTATTACTGCTAATGTCCAGAACAATGTAATATAGAGGACA  51489
              ** ************ ********************************************

Dromedary     ATAAAATGATATCTTTAAAATGCTTAAAGGAAAATGATGATAACAGAGAATTTTATATCC  51345
Wild          ATAAAATGATATCTTTAAAATGCTTAAAGGAAAATGATGATAACAGAGAATTTTATATCC  51119
Domestic      ATAAAATGATATCTTTAAAATGCTTAAAGGAAAATGATGATAACAGAGAATTTTATATCC  51549
              ************************************************************

Dromedary     AGTGAAAATATCCCTCAAAAATGACATACTTCTAAATAATCAATAGGTCAAAGAAATAAG  51405
Wild          AGTGAAAATATCCCTCAAAAATGACATACTTCTAAATAATCAATAGGTCAAAGAAATAAG  51179
Domestic      AGTGAAAATATCCCTCAAAAATGACATACTTCTAAATAATCAATAGGTCAAAGAAATAAG  51609
              ************************************************************

Dromedary     CACAAGAAAAAATTAGAAAATATTTTATATTGTGCATGACTTTAATGTTTACTTTGCTATG  51465
Wild          CACAAGAAAAAATTAGAAAATATTTTATATTGTGCATGACTTTAATGTTTACTTTGCTATG  51239
Domestic      CACAAGAAAAAATTAGAAAATATTTTATATTGTGCATGACTTTAATGTTTACTTTGCTATG  51669
              ************************************************************

Dromedary     CTGAAATTTGAAAGTTTTATCTGGACATGCTTATCAGGCTACAATAGAAAAGACTGATAA  51525
Wild          CTGAAATTTGAAAGTTTTATCTGGACATGCTTATCAGGCTACAATAGAAAAGACTGATAA  51299
Domestic      CTGAAATTTGAAAGTTTTATCTGGACATGCTTATCAGGCTACAATAGAAAAGACTGATAA  51729
              ************************************************************

Dromedary     ATGTGCCTAGTTAATTTTAAATTTCATCATGGCAAAGAAAACCTCAAACACATGGACAGA  51585
Wild          ATGTGCCTAGTTAATTTTAAATTTCATCATGGCAAAGGAAACCTCAAACACATGGACAGA  51359
Domestic      ATGTGCCTAGTTAATTTTAAATTTCATCATGGCAAAGGAAACCTCAAACGCATGGACAGA  51789
              ************************************* *********** **********

Dromedary     CAAGTGCTTAGAATGTATGCTACCCACTCCAAAACTTACATCATGCTATTCAAAACACAC  51645
Wild          CAAGTGCTTAGAATGTATGCTACCCACTCCAAAACTTACATCATGCTATTCAAAAAACAC  51419
Domestic      CAAGTGCTTAGAATGTATGCTACCCACTCCAAAACTTACATCATGCTATTCAAAAAACAC  51849
              ******************************************************* ****

Dromedary     TCACATTTCCTTCTCATACTTTTATGCTTTCATTTTTGAGATCTAAATTTTTGATGAATA  51705
Wild          TCATATTTCCTTCTCATACTTTTATGCTTTCATTTTTGAGATCTAAATTTTTGATGAATA  51479
Domestic      TCATATTTCCTTCTCATACTTTTATGCTTTCATTTTTGAGATCTAAATTTTTGATGAATA  51909
              *** ********************************************************

Dromedary     CAAAATTGTCCCTGTTTTCATGGAGCTTACAGTCTAGTGGCCAAGGCTCCCTGGGCTATT  51765
Wild          CAAAATTGTCCCTGTTTTCATGGAGCTTACAGTCTAGTGGCCAAGGCTCCCTGGGCTATT  51539
Domestic      CAAAATTGTCCCTGTTTTCATGGAGCTTACAGTCTAGTGGCCAAGGCTCCCTGGGCTATT  51969
              ************************************************************

Dromedary     TCTGTTGCAATACCAATACACAAGCCATCTATTTAATGGCTTAACTGAAAATGACACTTT  51825
Wild          TCTGTTGCAATACCAATACACAAGCCATCTATTTAATGGCTTAACTGAAAATGACACTTT  51599
Domestic      TCTGTTGCAATACCAATACACAAGCCATCTATTTAATGGCTTAACTGAAAATGACACTTT  52029
              ************************************************************

Dromedary     TATCACTTATGTGATTTTCTTACATGCTTGTGTCTATTTTGACTCTTCTTTCTGTTTTAC  51885
Wild          TATCACTTATGTGATTTTCTTACATGCTTGTGTCTATTTTGACTCTTCTTTCTGTTTTAC  51659
Domestic      TATCACTTATGTGATTTTCTTACATGCTTGTGTCTATTTTGACTCTTCTTTCTGTTTTAC  52089
              ************************************************************

Dromedary     TTCTCTTTTTATTATTCTATTAATACCATGCTATTTTAAACTACTAAGCCTTAAAATGTT  51945
Wild          TTCTCTTTTTATTATTCTATTAATACCATGCTATTTTAAACTACTAAGCCTTAAAATGTT  51719
Domestic      TTCTCTTTTTATTATTCTATTAATACCATGCTATTTTAAACTACTAAGCCTTAAAATGTT  52149
              ************************************************************

Dromedary     TTCACATCCATTAATGCCAGTCCCCTCTCATTCCTCTTCCTTATCAAAATTTACCTACCT  52005
Wild          TTCACATCCATTAATGCCAGTCCCCTCTCATTCCTCTTCCTTATCAAAATTTACCTACCT  51779
Domestic      TTCACATCCATTAATGCCAGTCCCCTCTCATTCCTCTTCCTTATCAAAATTTACCTACCT  52209
              ************************************************************

Dromedary     ATTCTGAAATATTTGTTACATCTAAAAGATCGATTGAGGTTTTAATTGGAAATTAGGTTG  52065
Wild          ATTCTGAAATATTTGTTACATCTAAAAGATCGATTGAGGTTTTAATTGGAAATTAGGTTG  51839
Domestic      ATTCTGAAATATTTGTTACATCTAAAAGATCGATTGAGGTTTTAATTGGAAATTAGGTTG  52269
              ************************************************************
```

```
Dromedary      CATTTATAGACTAAAGAGAATGACATTTATACATCATTCTTTCTATTCAAGAAAAGAATA  52125
Wild           CATTTATAGACTAAAGAGAATGACATTTATACATCATTCTTTCTATTCAAGAAAAGAATA  51899
Domestic       CATTTATAGACTAAAGAGAATGACATTTATACATCATTCTTTCTATTCAAGAAAAGAATA  52329
               ************************************************************

Dromedary      TGTCTTTCCATTTATTATTTGAGTTTTAATATTTTCTTCATATGGAGCAGCGGGCTTTTT  52185
Wild           TGTCTTTCCATTTATTATTTGAGTTTTAATATTTTCTTCATATGGAGCAGCGGGCTTTTT  51959
Domestic       TGTCTTTCCATTTATTATTTGAGTTTTAATATTTTCTTCATATGGAGCAGCGGGCTTTTT  52389
               ************************************************************

Dromedary      CCCTGGTTGTACATTACAATCACTTAGATGGTTTTTAAAAATACAGATGTTTAGGCCCTG  52245
Wild           CCCTGGTTGTACATTACAATCACTTAGATGGTTTTTAAAAATACAGATGTTTAGGCCCTG  52019
Domestic       CCCTGGTTGTACATTACAATCACTTAGATGGTTTTTAAAAATACAGATGTTTAGGCCCTG  52449
               ************************************************************

Dromedary      TTACCAAAGGCTTAACTGGTTTGGTGTGGAGCATAGGAGTTGTGTATTTATTAAGAGCTT  52305
Wild           TTACCAAAGGCTTAATTGGTTTGGTGTAGAGCATAGGAGTTGTGTATTTATTAAGAGCTT  52079
Domestic       TTACCAAAGGCTTAATTGGTTTGGTGTGGAGCATAGGAGTTGTGTATTTATTAAGAGCTT  52509
               *************** *********** ********************************

Dromedary      AACCCCATTCACAGTTAACAACTACCAGTGCAGGGCCTTGCATACTTATAAGCTGAGTTC  52365
Wild           AACCCCATTCACAGTTAACAACTACCAGTGCAGGGCCTTGCATACTTATAAGCTGAGTTC  52139
Domestic       AACCCCATTCACAGTTAACAACTACCAGTGCAGGGCCTTGCATACTTATAAGCTGAGTTC  52569
               ************************************************************

Dromedary      TTCATAGCTGTTTTTATTTGCTTTGGCTTAGTTTTGGCTGTTATTTTGAATGGCATCTTT  52425
Wild           TTCATAGCTGTTTTTATTTGCTTTGGCTTAGTTTTGGCTGTTATTTTGAATGGCATCTTT  52199
Domestic       TTCATAGCTGTTTTTATTTGCTTTGGCTTAGTTTTGGCTGTTATTTTGAATGGCATCTTT  52629
               ************************************************************

Dromedary      TTTGTTCATTATATTTTAAAACTGATTACTTTTTACATAAAGAAAACCTATGATTGCAGA  52485
Wild           TTTGTTCATTATATTTTAAAATTGATTACTTTTTACATAAAGAAAATCTATGATTGCAGA  52259
Domestic       TTTGTTCATTATATTTTAAAATTGATTACTTTTTACATAAAGAAAATCTATGATTGCAGA  52689
               ********************* ************************ *************

Dromedary      GTGCACTAATTTTGTAACATGTCACCTTACTGAATTTGTTTCATATGTTTTTCAGTCAGT  52545
Wild           GTGCACTAATTTTGTAACATGTCACCTTACTGAATTTGTTTCATATGTTTTTCAGTCAGT  52319
Domestic       GTGCACTAATTTTGTAACATGTCACCTTACTGAATTTGTTTCATATGTTTTTCAGTCAGT  52749
               ************************************************************

Dromedary      TCTTTATATTTTTATAGGCATTAAATTATACCACCTGCAAAAATGACAACTGTTCCTTTT  52605
Wild           TCTTTATATTTTTATAGGCATTAAATTATACCACCTGCAAAAATGACAACTGTTCCTTTT  52379
Domestic       TCTTTATATTTTTATAGGCATTAAATTATACCACCTGCAAAAATGACAACTGTTCCTTTT  52809
               ************************************************************

Dromedary      CTTATATTTATGCCTCCTTCCACTATCTTGTTCATATTCCATAAGAATAAAAGTAATAGT  52665
Wild           CTTATATTTATGCCTCCTTCCACTATCTTGTTCATATTCCATAAGAATAAAAGTAATAGT  52439
Domestic       CTTATATTTATGCCTCCTTCCACTATCTTGTTCATATTCCATAAGAATAAAAGTAATAGT  52869
               ************************************************************

Dromedary      TATGGTTGAGTGTATCCTTAACATATTTCTGATTTTAATGAGAATATGTATAGTGTTTCA  52725
Wild           TATGGTTGAGTGTATCCTTAACATATTTCTGATTTTAATGAGAATATGTATAGTGTTTCA  52499
Domestic       TATGGTTGAGTGTATCCTTAACATATTTCTGATTTTAATGAGAATATGTATAGTGTTTCA  52929
               ************************************************************

Dromedary      TCATTAAGCATAATGCTGATTTATCACTTTAAGAAAATATCCATCTACTCATATTTTACT  52785
Wild           TCATTAAGCATAATGCTGATTTATCACTTTAAGAAAATATCCATCTACTCATATTTTACT  52559
Domestic       TCATTAAGCATAATGCTGATTTATCACTTTAAGAAAATATCCATCTACTCATATTTTACT  52989
               ************************************************************

Dromedary      GAAGGTTGTTTTTTTTTTTTTAACAAGGAATGGTCCTAACTAAAGAATTCCTATTTAAATT  52845
Wild           GAAGGTTGTTTTTTTTTTTTTAACAAGGAATGGTCCTAACTAAAGAATTCCTATTTAAATT  52619
Domestic       GAAGGTTGTTTTTTTTTTTTTAACAAGGAATGGTCCTAACTAAAGAATTCCTATTTAAATT  53049
               ************************************************************

Dromedary      CTTTCAAGTGTTTTACAGGACTATATTAAGATGATCATGTTTTCCTCCTCCTCTTACCTA  52905
Wild           CTTTCAAGTGTTTTACAGGACTATATTAAGATGATCATGTTTTCCTCCTCCTCTTACCTA  52679
Domestic       CTTTCAAGTGTTTTACAGGACTATATTAAGATGATCATGTTTTCCTCCTCCTCTTACCTA  53109
               ************************************************************
```

```
Dromedary      TTAATATGTTGAACGAACCATGTTTGTGCTGCTGGAATGACTATGTTTCATTTCAGTATA 52965
Wild           TTAATATGTTGAAAGAACCATGTTTGTGCTGCTGGAATGACTATGTTTCATTTCAGTATA 52739
Domestic       TTAATATGTTGAAAGAACCATGTTTGTGCTGCTGGAATGACTATGTTTCATTTCAGTATA 53169
               ************* **********************************************

Dromedary      ATGTTCTTTCAATGTACTACAGAACTCTACTTCTAATATTTAATTTAAAAATTCAGGTGG 53025
Wild           ATGTTCTTTCAATGTACTACAGAACTCTACTTCTAATATTTAATTTAAAAATTCAGGTGG 52799
Domestic       ATGTTCTTTCAATGTACTACAGAACTCTACTTCTAATATTTAATTTAAAAATTCAGGTGG 53229
               ************************************************************

Dromedary      GTAGTCTTTTCTTGCTTTTATTGTCATGTGTTAGCATCAGTAGCTCAATTTTTTTTTGTA 53085
Wild           GTAGTCTTTTCTTGCTTTTATTGTCATGTGTTAGCATCAGTAGCTCAA-TTTTTTTTGTA 52858
Domestic       GTAGTCTTTTCTTGCTTTTATTGTCATGTGTTAGCATCAGTAGCTCAA-TTTTTTTTGTA 53288
               ************************************************ ***********

Dromedary      AGCTTCCTGTATTAATTTTTATTCTTTAATTTATTAAAATTCTAAGACAGTGGCTTTCAG 53145
Wild           AGCTTCCTGTATTAATTTTTATTCTTTAATTTATTAAAATTCTAAGTCAGTGGCTTTCAG 52918
Domestic       AGCTTCCTGTATTAATTTTTATTCTTTAATTTATTAAAATTCTAAGACAGTGGCTTTCAG 53348
               ********************************************* **************

Dromedary      AGTATTTTTATTGTGACCCAGAGTAAGAAAGGCAGCTTTACTGGAGACATGGAAGAAGAG 53205
Wild           AGTATTTTTATTGTGACCCAGAGTAAGAAAGGCAGCTTTACTGGAGACATGGAAGAAGAG 52978
Domestic       AGTATTTTTATTGTGACCCAGAGTAAGAAAGGCAGCTTTACTGGAGACATGGAAGAAGAG 53408
               ************************************************************

Dromedary      TCTGTTTCTTTTCCAGTATCTCTGTGCTTCCTTCCTCCCCGTGGGATGAAGACATAACTC 53265
Wild           TTTGTTTCTTTTCCAGCATCTCTGTGCTTCCTTCCTCCCCGTAGGATGAAGACATAACTC 53038
Domestic       TCTGTTTCTTTTCCAGCATCTCTGTGCTTCCTTCCTCCCCGTAGGATGAAGACATAACTC 53468
               * ************** ************************** ****************

Dromedary      ACCTCCCCTGCCTTTAACATGTCCAACTCATCACAAATCCCCAGTGAGTAGCACCTGAGT 53325
Wild           ACCTCCCCTGCCTTTAACATGTCCAACTCATCACAAATCCCCAGTGAGTAGCACCTGAGT 53098
Domestic       ACCTCCCCTGCCTTTAACATGTCCAACTCATCACAAATCCCCAGTGAGTAGCACCTGAGT 53528
               ************************************************************

Dromedary      TACTCAAGTTTTGCTGTTATACTTTTAGACTTTAAAAAAATTTAGTTTTCAAAATAGATA 53385
Wild           TACTCAAGTTTTGCTGTTATACTTTTAGACTTTAAAAAAATTTAGTTTTCAAAATAGATA 53158
Domestic       TACTCAAGTTTTGCTGTTATACTTTTAGACTTTAAAAAAATTTAGTTTTCAAAATAGATA 53588
               ************************************************************

Dromedary      ATACATTCTCATGGGTCAAAACCAGAACATACAAAAAGTTATATTCACCTAACTCATGTT 53445
Wild           ATACATTCTCATGGGTCAAAACCAGAACATACAAAAAGTTATATTCACCTAACTCATGTT 53218
Domestic       ATACATTCTCATGGGTCAAAACCAGAACATACAAAAAGTTATATTCACCTAACTCATGTT 53648
               ************************************************************

Dromedary      TCTATTAACCCAGTTCCTCCCCTCCAGATGTAGCTACTCTTCTTAATTGCTTACATATAC 53505
Wild           TCTATTAACCCAGTTCCTCCCCTCCAGATGTAGCTACTCTTCTTAATTGCTTACATATAC 53278
Domestic       TCTATTAACCCAGTTCCTCCCCTCCAGATGTAGCTACTCTTCTTAATTGCTTACATATAC 53708
               ************************************************************

Dromedary      TTAGTTCCAGAGTTGTTTAACGCACATACAAGCAAAGTTCAAATATGTGTTCTTATATTT 53565
Wild           TTAGTTCCAGAGTTGTTTAACGCACATACAAGCAAAGTTCAAATATGTGTTCTTATATTT 53338
Domestic       TTAGTTCCAGAGTTGTTTAACGCACATACAAGCAAAGTTCAAATATGTGTTCTTATATTT 53768
               ************************************************************

Dromedary      CTCTCTTTACCAGAGCATAACATAATTTTTTGATCTCTGTGTGTGTGTATTTTTTGTTTT 53625
Wild           CTCTCTTTACCAGAGCATAACATAATTTTTTGATCTCTGTGTGTGTGTATTTTTTGTTTT 53398
Domestic       CTCTCTTTACCAGAGCATAACATAATTTTTTGATCTCTGTGTGTGTGTATTTTTTGTTTT 53828
               ************************************************************

Dromedary      TGTCTTGTTTTGTTTTTAATTAATATGTTTTGGAAACTTTTCCTTACCTGTTCATAATTT 53685
Wild           TGTCTTGTTTTGTTTTTAATTAATATGTTTTGGAAACTTTTCCTTACCTGTTCATAATTT 53458
Domestic       TGTCTTGTTTTGTTTTTAATTAATATGTTTTGGAAACTTTTCCTTACCTGTTCATAATTT 53888
               ************************************************************

Dromedary      GCTTTCTCATTCTTTTGTACAGTGGCACAGTATTCCAGTATGTGAATGCATCACAGTTTT 53745
Wild           GCTTTCTCATTCTTTTGTACAGTGGCACAGTATTCCAGTATGTGAATGCATCACAGTTTT 53518
Domestic       GCTTTCTCATTCTTTTGTACAGTGGCACAGTATTCCAGTATGTGAATGCATCACAGTTTT 53948
               ************************************************************
```

```
Dromedary      TACTTAATTAGACCTTTATTGATAGAAAAAAAGAGAAAGAAATGCAGCTTTAAAAGGAGA  53805
Wild           TACTTAATTAGACCTTTATTGATAGAAAAAAAGAGAAAGAAATGCAGCTTTAAAAGGAGA  53578
Domestic       TACTTAATTAGACCTTTATTGATAGAAAAAAAGAGAAAGAAATGCAGCTTTAAAAGGAGA  54008
               ************************************************************

Dromedary      TCTAGCATAAGTATACGCACTGTAACAAGTTTCACATAATACTTGCTCTATGAGTATTCA  53865
Wild           TCTAGCATAAGTATACGCACTGTAACAAGTTTCACATAATACTTGCTCTATGAGTATTCA  53638
Domestic       TCTAGCATAAGTATACGCACTGTAACAAGTTTCACATAATACTTGCTCTATGAGTATTCA  54068
               ************************************************************

Dromedary      CTCCGTACTATTCTATTTCTTTAAAAAAATGCTCATCTCAAAACACTATTTTGAAAAATA  53925
Wild           CTCCGTACTATTCTATTTCTTTAAAAAAATGCTCATCTCAAAACACTATTTTGAAAAATA  53698
Domestic       CTCCGTACTATTCTATTTCTTTAAAAAAATGCTCATCTCAAAACACTATTTTGAAAAATA  54128
               ************************************************************

Dromedary      CTGTATGTTATTTGAAGATTTGGAAGAATTATCTATAAACTGATGTTTTTCAGAAAGTAG  53985
Wild           CTGTATGTTATTTGAAGATTTGGAAGAATTATCTATAAACTGATGTTTTTCAGAAAGTAG  53758
Domestic       CTGTATGTTATTTGAAGATTTGGAAGAATTATCTATAAACTGATGTTTTTCAGAAAGTAG  54188
               ************************************************************

Dromedary      TTCTCTAGCAATTATTCCATTTTCTTTCTTGCTTATGTTTTTTCTAGACAATCTATACCA  54045
Wild           TTCTCTAGCAATTATTCCATTTTCTTTCTTGCTTATGTTTTTTCTAGACAATCTATACCA  53818
Domestic       TTCTCTAGCAATTATTCCATTTTCTTTCTTGCTTATGTTTTTTCTAGACAATCTATACCA  54248
               ************************************************************

Dromedary      TCTTAATTTCACACAGGTCTTCAAATTTATTTGCATAATACTTTCGCAGTACACTTTTTA  54105
Wild           TCTTAATTTCACACAGGTCTTCAAATTTATTTGCATAATACTTTCGCAGTACACTTTTTA  53878
Domestic       TCTTAATTTCACACAGGCCTTCAAATTTATTTGCATAATACTTTCGCAGTACACTTTTTA  54308
               ***************** ******************************************

Dromedary      AATTCTTTAATTCTTTCACAGGGTCTGTAAATCGTTTCTCTCTCTCGTGCCTTCTTTATG  54165
Wild           AATTCTTTAATTCTTTCACAGGGTCTGTAAATCGTTTCTCTCTCTCGTGCCTTCTTTATG  53938
Domestic       AATTCTTTAATTCTTTCACAGGGTCTGTAAATCGTTTCTCTCTCTCGTGCCTTCTTTATG  54368
               ************************************************************

Dromedary      TTAGTCAGCTTAGCTAGTATATCGGGATTTTTATTTTCTCCTTAAGTAAAGAAATTAGAG  54225
Wild           TTAGTCAGCTTAGCTAGTATATTGGGATTTTTATTTTCTCCTTAAGTAAAGAAATTAGAG  53998
Domestic       TTAGTCAGCTTAGCTAGTATATTGGGATTTTTATTTTCTCCTTAAGTAAAGAAATTAGAG  54428
               ********************** *************************************

Dromedary      ATTTATATTATCAATTCTATTGTTTCTGATTTCTGATTTTACCTTTTTATTTCTCTTCTG  54285
Wild           ATTTATATTATCAATTCTATTGTTTCTGATTTCTGATTTTACCTTTTTATTTCTCTTCTG  54058
Domestic       ATTTATATTATCAATTCTATTGTTTCTGATTTCTGATTTTACCTTTTTATTTCTCTTCTG  54488
               ************************************************************

Dromedary      CTTTCTGAGGTTTTTTGTTTGTTTGTTTGTTTTGTTTGTTTTCTTTCTTTTTTTTTTTCT  54345
Wild           CTTTCTGAGGTTTTTT-TTTGTTTGTTT---------------TTTTTTTTTTG-GTTTT  54101
Domestic       CTTTCTGAGGTTTTTT-TTTGTTTGTTT---------------TTTTTTTTTGGTTTTTT  54532
               **************** ***********               *** *****    ** *

Dromedary      TTTTTTTGCTTTCTGAGGTTTAACTTGTCTGTTTTCTACTTTTTTGAGTTAAATAGTATG  54405
Wild           TTTTTTTGCTTTCTGAGGTTTAACTTGTCTGTTTTCTACTTTTTTGAGTTAAATAGTATG  54161
Domestic       TTTTTTTGCTTTCTGAGGTTTAACTTGTCTGTTTTCTACTTTTTTGAGTTAAATAGTATG  54592
               ************************************************************

Dromedary      TTATTTTTCTTCTTTCTTGTACTTGCATTTGAAACCATGACTATCTGATGAACATAGCAG  54465
Wild           TTATTTTTCTTCTTTCTTGTACTTGCATTTGAAACCATGACTATCTGATGAACATAGCAG  54221
Domestic       TTATTTTTCTTCTTTCTTGTACTTGCATTTGAAACCATGACTATCTGATGAACATAGCAG  54652
               ************************************************************

Dromedary      TAGCTGTATCCTATAGGTTAGGTGTGTAATGTTTCAA--ATTACTTTGTACACATTTTGC  54523
Wild           TAGCTGTATCCTATAGGTTAGGTGTGTAATGTTTCAATTATTACTTTGTACACATTTTGC  54281
Domestic       TAGCTGTATCCTATAGGTTAGGTGTGTAATGTTTCAATTATTACTTTGTACACATTTTGC  54712
               *************************************  *********************

Dromedary      AACATCAATTTGATTTCCTCTTTGTAAAAAGGTTGTTTTAAATAGTTTTTATTTTTCCAA  54583
Wild           AACATCAATTTGATTTCCTCTTTGTAAAAAGGTTGTTTTAAATAGTTTTTATTTTTCCAA  54341
Domestic       AACATCAATTTGATTTCCTCTTTGTAAAAAGGTTGTTTTAAATAGTTTTTATTTTTCCAA  54772
               ************************************************************
```

```
Dromedary      GTAGGGTAGATGTTAACTTTTATTTTGGTCATTCATTTTTTGTTGAATTGCATGGTTACC  54643
Wild           GTAGGGTAGATGTTAACTTTTATTTTGGTCATTCATTTTTTGTTGAATTGCATGGTTACC  54401
Domestic       GTAGGGTAGATGTTAACTTTTATTTTGGTCATTCATTTTTTGTTGAATTGCATGGTTACC  54832
               ************************************************************

Dromedary      AGAGAATGTGGTCTGGATTATTTCTAATTTTTGGAATTAATTGAGGGATCTTTTGTGTAG  54703
Wild           AGAGAATGTGGTCTGGATTATTTCTAATTTTTGGAATTAATTGAGGGATCTTTTGTGTAG  54461
Domestic       AGAGAATGTGGTCTGGATTATTTCTAATTTTTGGAATTAATTGAGGGATCTTTTGTGTAG  54892
               ************************************************************

Dromedary      GGGGGCTAATAATATAGTCAATTTGGTAAATGTTTCATGAGCTTTTAAAAATAAGATGAT  54763
Wild           GGGGGCCAATAATATAGTCAATTTGGTAAATGTTTCATGAGCTTTTAAAAATAAGATGAT  54521
Domestic       GGGGGCCAATAATATAGTCAATTTGGTAAATGTTTCATGAGCTTTTAAAAATAAGATGAT  54952
               ****** *****************************************************

Dromedary      TTTCAGTTAACAAGATACCTATTTCATCTATTTCATCTACCAAGTCTATTTCATTAACCT  54823
Wild           TTTCAGTTAACAAGATACCTATTTCATCTATTTCATCTACCAACTCTATTTCATTAACCT  54581
Domestic       TTTCAGTTAACAAGATACCTATTTCATCTATTTCATCTACCAACTCTATTTCATTAACCT  55012
               ******************************************* ****************

Dromedary      CATGGACTTCATTGTTCAAATATATCCTTACCAACTTTTGATGACTTGATCCATGAAATG  54883
Wild           CATGGACTTCATTGTTCAAATATATCCTTACCAACTTCTGATGACTTGATCCATGAAATG  54641
Domestic       CATGGACTTCATTGTTCAAATATATCCTTACCAACTTCTGATGACTTGATCCATGAAATG  55072
               ************************************* **********************

Dromedary      AATTAAAGTAGGGTACTACTATTTTGTTTCTAATCATTTTTCTTTGTAGTTCTGCGTTTT  54943
Wild           AATTAAAGTAGGGTACTACTATTTTGTTTCTAATCATTTTTCTTTGTAGTTCTGCGTTTT  54701
Domestic       AATTAAAGTAGGGTACTACTATTTTGTTTCTAATCATTTTTCTTTGTAGTTCTGCGTTTT  55132
               ************************************************************

Dromedary      TTCTTTATGTGTTTTATTGTATTATGTAGTGCTTTAAGATTTATGAAAGGTGTATCCTCT  55003
Wild           TTCTTTATGTGTTTTATTGTATTATGTAGTGCTTTAAGATTTATGAAAGGTGTATCCTCT  54761
Domestic       TTCTTTATGTGTTTTATTGTATTATGTAGTGCTTTAAGATTTATGAAAGGTGTATCCTCT  55192
               ************************************************************

Dromedary      CTGTAGATCTTTTACCATCATAAAATTTCCTCCTTCATCTTGTATTATACTTTTTGCCTT  55063
Wild           CTGTAGATCTTTTACCATCATAAAATTTCCTCCTTCATCTTGTATTATACTTTTTGCCTT  54821
Domestic       CTGTAGATCTTTTACCATCATAAAATTTCCTCCTTCATCTTGTATTATACTTTTTGCCTT  55252
               ************************************************************

Dromedary      AAATTTCACCCTATCTGAGTGATATCATGACCCTAACTTTCTTTTTGTTCACCTTTGCCT  55123
Wild           AAATTTCACCCTATCTGAGTGATATCATGACCCCAACTTTCTTTTTGTTCACCTTTGCCT  54881
Domestic       AAATTTCACCCTATCTGAGTGATATCATGACCCCAACTTTCTTTTTGTTCACCTTTGCCT  55312
               ********************************* **************************

Dromedary      AGCATATCTTTTCCCACTTTTTTCTCTCTAGTTTTCTAGTCATTTTGCTTTAGATCTACC  55183
Wild           GGCATATCTTTTCCCACTTTTTTCTCTCTAGTTTTCTAGTCATTTTGCTTTAGATCTACC  54941
Domestic       GGCATATCTTTTCCCACTTTTTTCTCTCTAGTTTTCTAGTCATTTTGCTTTAGATCTACC  55372
                ***********************************************************

Dromedary      TCTGTGTACATCATATATGCCTATCCCTCACTTTACAAAAGTTAAATTTATAAATTTTCA  55243
Wild           TCTGTGTACATCATATATGCCTATCCCTCACTTTACAAAAGTTAAATTTATAAATTTTCA  55001
Domestic       TCTGTGTACATCATATATGCCTATCCCTCACTTTACAAAAGTTAAATTTATAAATTTTCA  55432
               ************************************************************

Dromedary      ATTTTGCAAAAAGTTCTAATACAAATTAATATTAGTAATATAAATATTGTTAGGTACTAC  55303
Wild           ATTTTGCAAAAAGTTCTAATACAAATTAATATTAGTAATATAAATATTGTTAGGTACTAC  55061
Domestic       ATTTTGCAAAAAGTTCTAATACAAATTAATATTAGTAATATAAATATTGTTAGGTACTAC  55492
               ************************************************************

Dromedary      TGGTACAATTGTTATTAAATACTGCCAATGATTTTAACTTTTACCTGGCATCAGGCACTG  55363
Wild           TGGTACAATTGTTATTAAATACTGCCAATGATTTTAACTTTTACCTGGCATCAGGCACTG  55121
Domestic       TGGTACAATTGTTATTAAATACTGCCAATGATTTTAACTTTTACCTGGCATCAGGCACTG  55552
               ************************************************************

Dromedary      TTCTTGATGCCTTACATTATTTTCTCATTTAGTCCTCACAACTTCACCAATATTTCCATT  55423
Wild           TTCTTGATGCCTTACATTATTTTCTCATTTAGTCCTCACAACTTCACCAATATTTCCATT  55181
Domestic       TTCTTGATGCCTTACATTATTTTCTCATTTAGTCCTCACAACTTCACCAATATTTCCATT  55612
               ************************************************************
```

```
Dromedary      CTGCAGATGAGGAAATGAACGTTTCCTAATTTGTCCAAAGTCAGGCAGCTCATAGAGAGT  55483
Wild           CTGCAGATGAGGAAATGAACGTTTCCTAATTTGTCCAAAGTCAGGCAGCTCATAGAGAGT  55241
Domestic       CTGCAGATGAGGAAATGAACGTTTCCTAATTTGTCCAAAGTCAGGCAGCTCATAGAGAGT  55672
               ************************************************************

Dromedary      GGCTAGCAAAAGAGTTTATTTATTCATGGGTTTTGGAGAGGGGAGTCTTTATAATTTTGT  55543
Wild           GGCTAGCAAAAGAGTTTATTTATTCATGGGTTTTGGAGAGGGGAGTCTTTATAATTTTGT  55301
Domestic       GGCTAGCAAAAGAGTTTATTTATTCATGGGTTTTGGAGAGGGGAGTCTTTATAATTTTGT  55732
               ************************************************************

Dromedary      TTCTAGTCACCCATTTGTTCTCCTCTTTTCACTCGTAAGTCTCCAGTGTACATACCATTT  55603
Wild           TTCTAGTCATCCATTTGTTCTCCTCTTTTCACTCGTAAGTCTCCAGTGTACATACCATTT  55361
Domestic       TTCTAGTCACCCATTTGTTCTCCTCTTTTCACTCGTAAGTCTCCAGTGTACATACCATTT  55792
               ********* **************************************************

Dromedary      CAGCTACACCCAGGAAACTGTTGGTTTGCCAAATAGTCCAACTTCTTTCCATAATTTTGG  55663
Wild           CAGCTACACCCAGGAAACTGTTGGTTTGCCAAATAGTCCAACTTCTTTCCATAATTTTGG  55421
Domestic       CAGCTACACCCAGGAAACTGTTGGTTTGCCAAATAGTCCAACTTCTTTCCATAATTTTGG  55852
               ************************************************************

Dromedary      ACTTTCTCAAGTGCTGCTTTAACTATTACCCACTCTTCTCTAGGGGCAGCACGGTGTTGT  55723
Wild           ACTTTCTCAAGTGCTGCTTTAACTATTACCCACTCTTCTCTAGGGGCAGCACGGTGTTGT  55481
Domestic       ACTTTCTCAAGTGCTGCTTTAACTATTACCCACTCTTCTCTAGGGGCAGCACGGTGTTGT  55912
               ************************************************************

Dromedary      ATATAAATATACTCAATGCCTACCCACAACCAGTGAGTGTTAAACGAATATTTCATGAAA  55783
Wild           ATATAAATATACTCAATGCCTACCCACAACCAGTGAGTGTTAAACGAATATTTCATGAAA  55541
Domestic       ATATAAATATACTCAATGCCTACCCACAACCAGTGAGTGTTAAACGAATATTTCATGAAA  55972
               ************************************************************

Dromedary      CGAAATTCTAAGTAGAGTCTTTATCAAATTTTATCAAGTATTTAAATTTCAGCTGTTCTC  55843
Wild           CGAAATTCTAAGTAGAGTCTTTATCAAATTTTATCAAGTATTTAAATTTCAGCTGTTCTC  55601
Domestic       CGAAATTCTAAGTAGAGTCTTTATCAAATTTTATCAAGTATTTAAATTTCAGCTGTTCTC  56032
               ************************************************************

Dromedary      TCATCAGATTTCTCCTCTGAGTCTAACTGAACATGTAAAATATTAGACTTATTCATATTT  55903
Wild           TCATCAGATTTCTCCTCTGAGTCTAACTGAACATGTAAAATATTAGATTTATTCATATTT  55661
Domestic       TCATCAGATTTCTCCTCTGAGTCTAACTGAACATGTAAAATATTAGATTTATTCATATTT  56092
               *********************************************** ************

Dromedary      AAAATTAAGGTAGAAAATTTGATTATATTACAGTGGATTTAAACTTACTGTATCATGCAA  55963
Wild           AAAATTAAGGTAGAAAATTTGATTATATTACAGTGGATTTAAACTCACTGTATCATGCAA  55721
Domestic       AAAATTAAGGTAGAAAATTTGATTATATTACAGTGGATTTAAACTCACTGTATCATGCAA  56152
               ********************************************* **************

Dromedary      AAATGTCTAATATTTAACACATATTTTGAGTCTTAATAATAGTAATGCCTTACTTATGGA  56023
Wild           AAATGTCTAATATTTAACACATATTTTGAGTCTTAATAATAGTAATGCCTTACTTATGGA  55781
Domestic       AAATGTCTAATATTTAACACATATTTTGAGTCTTAATAATAGTAATGCCTTACTTATGGA  56212
               ************************************************************

Dromedary      TTTTATTCAATAAATGTATGTTATAAAAAAGAGAGAAGATACATAAAACTAAATTCAAGG  56083
Wild           TTTTATTTAATAAATGTATGTTATAAAAAAGAGAGAAGATACATAAAACTAAATTCAAGG  55841
Domestic       TTTTATTTAATAAATGTATGTTATAAAAAAGAGAGAAGATACATAAAACTAAATTCAAGG  56272
               ******* ****************************************************

Dromedary      TTTAATCTGAAGTCCTTAAGACCTTAGAAAAATTGGTAGTATATAGGTGATGTAGTTCTT  56143
Wild           TTTAATCTGAAGTCCTTAAGACCTTAGAAAAATTGGTAGTATATAGGTGATGTAGTTCTT  55901
Domestic       TTTAATCTGAAGTCCTTAAGACCTTAGAAAAATTGGTAGTATATAGGTGATGTAGTTCTT  56332
               ************************************************************

Dromedary      AGAGTTCCCAGCAGGGGCCAGAATGTTCATGTTAAATCTAATTAAATGAAAATGTTTTTA  56203
Wild           AGAGTTCCCAGCAGGGGCCAGAATGTTCATGTTAAATCTAATTAAATGAAAATGTTTTTA  55961
Domestic       AGAGTTCCCAGCAGGGGCCAGAATGTTCATGTTAAATCTAATTAAATGAAAATGTTTTTA  56392
               ************************************************************

Dromedary      CTACAATTCAAGACTGTACTCATTTTTAAACCAATATGAAAGTCAGATAGAACTCTTTAT  56263
Wild           CTACAATTCAAGACTGTACTCATTTTTAAACCAATATGATAGTCAGATAGAACTCTTTAT  56021
Domestic       CTACAATTCAAGACTGTACTCATTTTTAAACCAATATGAAAGTCAGATAGAACTCTTTAT  56452
               *************************************** ********************
```

```
Dromedary      AATTAAAATTACTAGAAACTTCTGACTTATTAATTTAAATTTTCAAGAATGTAAATATAT 56323
Wild           AATTAAAATTACTAGAAACTTCTGACTTATTAATTTAAATTTTCAAGAATGTAAATATAT 56081
Domestic       AATTAAAATTACTAGAAACTTCTGACTTATTAATTTAAATTTTCAAGAATGTAAATATAT 56512
               ************************************************************

Dromedary      AATTCAAAATATCATAAAATAAGCTTAACAGAAATAGTAATGTTTTGTAACAAAAGGCTG 56383
Wild           AATTCAAAATATCATAAAATAAGCTTAACAGAAATAGTAATGTTTTGTAACAAAAGGCTG 56141
Domestic       AATTCAAAATATCATAAAATAAGCTTAACAGAAATAGTAATGTTTTGTAACAAAAGGCTG 56572
               ************************************************************

Dromedary      ACCCCCAATATCTGAGCAAATGGGTATCCAAAGTACGTTCAGTCAGTAGCTACTAAAACC 56443
Wild           ACCCCCAATATCTGAGCAAATGGGTATCCAAAGTACGTTCAGTCAGTAGCTACTAAAACC 56201
Domestic       ACCCCCAATATCTGAGCAAATGGGTATCCAAAGTACGTTCAGTCAGTAGCTACTAAAACC 56632
               ************************************************************

Dromedary      CCTACACTGATTGCCAAACTCAGCTCATTGGGTCTTTCAGTTGCCAAGCCAGACGATGGA 56503
Wild           CCTACACTGATTGCCAAACTCAGCTCATTGGGTCTTTCAGTTGCCAAGCCAGACGATGGA 56261
Domestic       CCTACACTGATTGCCAAACTCAGCTCATTGGGTCTTTCAGTTGCCAAGCCAGACGATGGA 56692
               ************************************************************

Dromedary      CTGGGTCCAAGAACAAAGGGCACCACTATGGCATTGAGCCAGGAAATTGAGTACTGACCT 56563
Wild           CTGGGTCCAAGAACAAAGGGCACCACTATGGCATTGAGCCAGGAAATTGAGTACTGACCT 56321
Domestic       CTGGGTCCAAGAACAAAGGGCACCACTATGGCATTGAGCCAGGAAATTGAGTACTGACCT 56752
               ************************************************************

Dromedary      TATTTGTTAATTTTAGAACATTAAAAAAAAAATTCTTAACTCAATATAAAAGAAGTACTG 56623
Wild           TATTTGTTAATTTTAGAACATTAAAAAAAAAATTCTTAACTCAATATAAAAGAAGTACTG 56381
Domestic       TATTTGTTAATTTTAGAACATTAAAAAAAAAATTCTTAACTCAATATAAAAGAAGTACTG 56812
               ************************************************************

Dromedary      GTGATTCAAATGTATTTCAATGCAAATTTCCTTTGTAAATCAAATAAACTGTTTTTAATT 56683
Wild           GTGATTCAAATGTATTTCAATGCAAATTTCCTTTGTAAATCAAATAAACTGTTTTTAATT 56441
Domestic       GTGATTCAAATGTATTTCAATGCAAATTTCCTTTGTAAATCAAATAAACTGTTTTTAATT 56872
               ************************************************************

Dromedary      TAATTCTAATTTAAGTCACGTAACTTCTCTCTGGGCCTGTTTCTCCATCTGTTCATGAGT 56743
Wild           TAATTCTAATTTAAGTCACGTAACTTCTCTCTGGGCCTGTTTCTCCATCTGTTCATGAGT 56501
Domestic       TAATTCTAATTTAAGTCACGTAACTTCTCTCTGGGCCTGTTTCTCCATCTGTTCATGAGT 56932
               ************************************************************

Dromedary      GGGTTAGATATGATAATCTTAAAATTCTGATGTTCCTAAAATTCAATTTTTTTTCCCTTA 56803
Wild           GGGTTAGATATGATAATCTTAAAATTCTGATGTTCCTAAAATTCAATTTTTTTTCCCTTA 56561
Domestic       GGGTTAGATATGATAATCTTAAAATTCTGATGTTCCTAAAATTCAATTTTTTTTCCCTTA 56992
               ************************************************************

Dromedary      GGCAAAAAGCATCATTTTTACCCTGTCCCTAGTTCTTGGCATAGTCCCTGTCACAAACTA 56863
Wild           GGCAAAAAGCATCATTTTTACCCTGTCCCTAGTTCTTGGCATAGTCCCTGTCACAAACTA 56621
Domestic       GGCAAAAAGCATCATTTTTACCCTGTCCCTAGTTCTTGGCATAGTCCCTGTCACAAACTA 57052
               ************************************************************

Dromedary      GGTGCTTAGTAAGTGTTTAATGAGCAAATAAATGAATAAACAAAACAAATGAATGCATTT 56923
Wild           GGTGCTTAGTAAGTGTTTAATGAGCAAATAAATGAATAAACAAAACAAATGAATGCATTT 56681
Domestic       GGTGCTTAGTAAGTGTTTAATGAGCAAATAAATGAATAAACAAAACAAATGAATGCATTT 57112
               ************************************************************

Dromedary      TTAGAGAGCTCTTTCTAGGAATTTACAAAAGAATTTGGAAATCAGATGCCTGTGAAAGCC 56983
Wild           TTAGAGAGCTCTTTCTAGGAATTTACAAAAGAATTTGGAAATCAGATGCCTGTGAAAGCC 56741
Domestic       TTAGAGAGCTCTTTCTAGGAATTTACAAAAGAATTTGGAAATCAGATGCCTGTGAAAGCC 57172
               ************************************************************

Dromedary      AGTCTTCTGTGAATTATG-AAAAAAAATGCTTTAGGCAGTTCCAAGGAAATAAGAAAATG 57042
Wild           AGTCTTCTGTGAATTATGAAAAAAAAATGCTTTAGGCAGTTCCAAGGAAATAAGAAAATG 56801
Domestic       AGTCTTCTGTGAATTATGAAAAAAAAATGCTTTAGGCAGTTCCAAGGAAATAAGAAAATG 57232
               ****************** *****************************************

Dromedary      TTTTCTGAATACTCTTGGGAAGAACTGAATTCTTCTATGGCTATCACTACTTTCTAGTCA 57102
Wild           TTTTCTGAATACTCTTGGGAAGAACTGAATTCTTCTATGGCTATCACTACTTTCTAGTCA 56861
Domestic       TTTTCTGAATACTCTTGGGAAGAACTGAATTCTTCTATGGCTATCACTACTTTCTAGTCA 57292
               ************************************************************
```

```
Dromedary      GTAACTGAGTAAAGAGGGAGTTAATGTTTAAAAAGGATTTTATACTTTAAATTTAAGATC 57162
Wild           GTAACTGAGTAAAGAGGGAGTTAATGTTTAAAAAGGATTTTATACTTTAAATTTAAGATC 56921
Domestic       GTAACTGAGTAAAGAGGGAGTTAATGTTTAAAAAGGATTTTATACTTTAAATTTAAGATC 57352
               ************************************************************

Dromedary      TAAATCTAAAAAAAAATTTTTTAATTATTTGAAGAAACATCCTCTGTATTGAGTCTATGG 57222
Wild           TAAATCTAAAAAAAATTTTTTTAATTATTTGAAGAAACATCCTCTGTATTGAGTCTATGG 56981
Domestic       TAAATCTAAAAAAAATTTTTTTAATTATTTGAAGAAACATCCTCTGTATTGAGTCTATGG 57412
               *************** ********************************************

Dromedary      TTTCCCAAGCTCCGCCAAAACAAAATCCTGAAAACTACAAGGTTGTTCTCTTGGGGAGTG 57282
Wild           TTTCCCAAGCTCCGCCAAAACAAAATCCTGAAAACTACAAGGTTGTTCTCTTGGGGAGTG 57041
Domestic       TTTCCCAAGCTCCGCCAAAACAAAATCCTGAAAACTACAAGGTTGTTCTCTTGGGGAGTG 57472
               ************************************************************

Dromedary      GTTGTTTACCAGAAGGCTTAATTCTTAATAATAAGGGTAAAGCTTTCAGAACTAAAGATA 57342
Wild           GTTGTTTACCAGAAGGCTTAATTCTTAATAATAAGGGTAAAGCTTTCAGAACTAAAGATA 57101
Domestic       GTTGTTTACCAGAAGGCTTAATTCTTAATAATAAGGGTAAAGCTTTCAGAACTAAAGATA 57532
               ************************************************************

Dromedary      TGCCTCAGCCAATTCAATCTACATTGTCATCTGCCCTGGAAACCATGTACTTGTTCACTT 57402
Wild           TGCCTCTGCCAATTCAATCTACATTGTCATCTGCCCTGGAAACCATGTACTTGTTCACTT 57161
Domestic       TGCCTCTGCCAATTCAATCTACATTGTCATCTGCCCTGGAAACCATGTACTTGTTCACTT 57592
               ****** *****************************************************

Dromedary      GTTCTAAAGTAAATGAAACCATCATGAAGGAAATCAATTATCAAAAATCACAACAATGAA 57462
Wild           GTTCTAAAGTAAATGAAACCATCATGAAGGAAATCAATTATCAAAAATCACAACAATGAA 57221
Domestic       GTTCTAAAGTAAATGAAACCATCATGAAGGAAATCAATTATCAAAAATCACAACAATGAA 57652
               ************************************************************

Dromedary      ACTTCTTATACTCGGTTTCTAATTTGTAAATTTTAAAAATATTACTTCATTAAATCTGGG 57522
Wild           ACTTCTTATACTCGGTTTCTAATTTGTAAATTTTAAAAATATTACTTCATTAAATCTGGG 57281
Domestic       ACTTCTTATACTCGGTTTCTAATTTGTAAATTTTAAAAATATTACTTCATTAAATCTGGG 57712
               ************************************************************

Dromedary      GGAAAATGTATAACTGCAGGCATAAGTTTTGTGCCTTCTTAATCCAAGGAGGTGGTCAGT 57582
Wild           GGAAAATGTATAATTGCAGGCATAAGTTTTGTGCCTTCTTAATCCAAGGAGGTGGTCAGT 57341
Domestic       GGAAAATGTATAACTGCAGGCATAAGTTTTGTGCCTTCTTAATCCAAGGAGGTGGTCAGT 57772
               ************* **********************************************

Dromedary      AGTATGGAAGCTTAGACCAAATGTGAGGATGTGGCTGTAGCAAATTTGAGAGCCTAACTA 57642
Wild           AGTATGGAAGCTTAGACCAAATGTGAGAATGTGGCTGTAGCAAATTTGAGAGCCTAACTA 57401
Domestic       AGTATGGAAGCTTAGACCAAATGTGAGGATGTGGCTGTAGCAAATTTGAGAGCCTAACTA 57832
               *************************** ********************************

Dromedary      GAGACTGCTGAAACATAGGTCAGGGAATCTGAAACATCCCCACCTGCTATTACACCACAT 57702
Wild           GAGACTGCTGAAACATAGGTCAGGGAATCTGAAACATCCCCACCTGCTATTACACCACAT 57461
Domestic       GAGACTGCTGAAACATAGGTCAGGGAATCTGAAACATCCCCACCTGCTATTACACCACAT 57892
               ************************************************************

Dromedary      AAACAAAAAGAAAATTTAAGTTCAGTTTAAAAAGGGCTAAATTAGGGAATCACAAACTTT 57762
Wild           AAACAAAAAGAAAATTTAAGTTCAGTTTAAAAAGGGCTAAATTAGTGAATCACAAACTTT 57521
Domestic       AAACAAAAAGAAAATTTAAGTTCAGTTTAAAAAGGGCTAAATTAGTGAATCACAAACTTT 57952
               ********************************************* **************

Dromedary      TTCCAAACATCAGCCACTGCCTTTGGGCAAAGGATTTAAGTAGCTCTGCTTATTTGCTAG 57822
Wild           TTCCAAACATCAGCCACTGCCTTTGGGCAAAGGATTTAAGTAGCTCTGCTTATTTGCTAG 57581
Domestic       TTCCAAACATCAGCCACTGCCTTTGGGCAAAGGATTTAAGTAGCTCTGCTTATTTGCTAG 58012
               ************************************************************

Dromedary      TAATGGAATAAAGACATTATTTGAGATAGATTCTCAAAACTTGAATAATGTCAAGATTCT 57882
Wild           TAATGGAATAAAGACATTATTTGAGATAGATTCTCAAAACTTGAATAATGTCAAGATTCT 57641
Domestic       TAATGGAATAAAGACATTATTTGAGATAGATTCTCAAAACTTGAATAATGTCAAGATTCT 58072
               ************************************************************

Dromedary      TTTCCAAGGAAAAACAATTTTTTGAGAACCACCTTAAATAAAAGTCAACCCCTATCACTT 57942
Wild           TTTCCAAGGAAAAACAATTTTTTGAGAACCACCTTAAATAAAAGTCAACCCCTATCACTT 57701
Domestic       TTTCCAAGGAAAAACAATTTTTTGAGAACCACCTTAAATAAAAGTCAACCCCTATCACTT 58132
               ************************************************************
```

```
Dromedary       TAGATGCCACTTGAAGCCAACTTGATTATAGTAAAAAATTAAACTGTAAACACTGTTTAA  58002
Wild            TAGATGCCACTTGAAGCCAACTTGATTATAGTAAAAAATTAAACTGTAAACACTGTTTAA  57761
Domestic        TAGATGCCACTTGAAGCCAACTTGATTATAGTAAAAAATTAAACTGTAAACACTGTTTAA  58192
                ************************************************************

Dromedary       CAACACTATAGACATTATATTTGCAGACACTGTAGAAAATAAAGCATAAAAATTGAAGCA  58062
Wild            CAACACTATAGACATTATATTTGCAGACACTGTAGAAAATAAAGCATAAAAATTGAAGCA  57821
Domestic        CAACACTATAGACATTATATTTGCAGACACTGTAGAAAATAAAGCATAAAAATTGAAGCA  58252
                ************************************************************

Dromedary       TCATTCAGATGTAAGGTGGTTGGTTACCTGGATGCTCCAAAATATGCATCAAGTACTAAG  58122
Wild            TCATTCAGATGTAAGGTGGTTGGTTACCTGGATGCTCCAAAATATGCATCAAGTACTAAG  57881
Domestic        TCATTCAGATGTAAGGTGGTTGGTTACCTGGATGCTCCAAAATATGCATCAAGTACTAAG  58312
                ************************************************************

Dromedary       TTTTGTGAAATTACTTCTGGAACTCACAGACACCCTTGGGGAGCTAAGGAACTTAGCTGT  58182
Wild            TTTTGTGAAATTACTTCTGGAACTCACAGACACCCTTGGGGAGCTAAGGAACTTAGCTGT  57941
Domestic        TTTTGTGAAATTACTTCTGGAACTCACAGACACCCTTGGGGAGCTAAGGAACTTAGCTGT  58372
                ************************************************************

Dromedary       AGAGAGCATTCTGGGAAAACAGGTTTACTTCTGGTTGACCTGAGCTGTCTCCTCACAGAG  58242
Wild            AGAGAGCATTCTGGGAAAACAGGTTTACTTCTGGTTGACCTGGGCTGTCTCCTCACAGAG  58001
Domestic        AGAGAGCATTCTGGGAAAACAGGTTTACTTCTGGTTGACCTGGGCTGTCTCCTCACAGAG  58432
                ****************************************** *****************

Dromedary       CTATGCCTTGTTTTAAACTGAGGCAATAAACTCCATCCCAGTTTTAGGGGAAAGTGCCTT  58302
Wild            CTATGCCTTGTTTTAAACTGAGGCAATAAACTCCATCCCAGTTTTAGGGGAAAGTGCCTT  58061
Domestic        CTATGCCTTGTTTTAAACTGAGGCAATAAACTCCATCCCAGTTTTAGGGGAAAGTGCCTT  58492
                ************************************************************

Dromedary       CCTGCTGGTATGAGGAGAAAGCAGGGAGCGAAGGAGTACTTTACGAAAAAGAAAATGTAG  58362
Wild            CCTGCTGGTATGAGGAGAAAGCAGGGAGCGAAGGAGTACTTTACGAAAAAGAAAATGTAG  58121
Domestic        CCTGCTGGTATGAGGAGAAAGCAGGGAGCGAAGGAGTACTTTACGAAAAAGAAAATGTAG  58552
                ************************************************************

Dromedary       TAGAAGACTATAACATGTATCATCAGTTCACAGTTCCACCCACTGGGTCAAGCAAAGAAG  58422
Wild            TAGAAGACTATAACATGTATCATCAGTTCACAGTTCCACCCACTGGGTCAAGCAAAGAAG  58181
Domestic        TAGAAGACTATAACATGTATCATCAGTTCACAGTTCCACCCACTGGGTCAAGCAAAGAAG  58612
                ************************************************************

Dromedary       ACTGTCCAGCCTTGGCGTTGCCTAAACATGCATGCGCGTGCGCGCGCGCG----------  58472
Wild            ACTGTCCAGCCTTGGCATTGCCTAAACATGCATGCGCGTGCGCGCGCACG----------  58231
Domestic        ACTGTCCAGCCTTGGCATTGCCTAAACATGCATACGCGTGCGCGCGCACGCACACACACA  58672
                **************** **************** ************* **

Dromedary       --------------CGCGCACACACACACACAGCCTTTTGAGATTTCTTTTCTCTGGCTA  58518
Wild            --CACACACACACACACACACACACACACACAGCCTTTTGAGATTTCTTTTCTCTGGCTA  58289
Domestic        CACACACACACACACACACACACACACACACAGCCTTTTGAGATTTCTTTTCTCTGGCTA  58732
                              * * ******************************************

Dromedary       CCTTTGACGCTCCTTAAACTCAGTTTGCCTTAAATAGGACTGTGTTAGGTAAGGGCTCTG  58578
Wild            CCTTTGACGCTCCTTAAACTCAGTTTGCCTTAAATAGGACTGTGTTAGGTAAGGGCTCTG  58349
Domestic        CCTTTGACGCTCCTTAAACTCAGTTTGCCTTAAATAGGACTGTGTTAGGTAAGGGCTCTG  58792
                ************************************************************

Dromedary       TAGTCTTAAACAGTGATGTCACAGCCAGCATGGTCACTCCCTAGCAAGGTCTTGTTAAGA  58638
Wild            TAGTCTTAAACAGTGATGTCACAGCCAGCATGGTCACTCCCTAGCAAGGTCTTGTTAAGA  58409
Domestic        TAGTCTTAAACAGTGATGTCACAGCCAGCATGGTCACTCCCTAGCAAGGTCTTGTTAAGA  58852
                ************************************************************

Dromedary       GCCACACAGTAGTCATAGATCATTTAAATGCAAAAGCTCCACTGGGATGTTCAAGTGCTC  58698
Wild            GCCACACAGTAGTCATAGATCATTTAAATGCAAAAGCTCCACTGGGATGTTCAAGTGCTC  58469
Domestic        GCCACACAGTAGTCATAGATCATTTAAATGCAAAAGCTCCACTGGGATGTTCAAGTGCTC  58912
                ************************************************************

Dromedary       AGTGCGCTACCACAGAGCTGTGAAAAGGGGCGAGGACAGAGGAACGTACCAGTACTGGTA  58758
Wild            AGTGCGCTACCACAGAGCTGTGAAAAGGGGCGAGGACAGAGGAACGTACCAGTACTGGTA  58529
Domestic        AGTGCGCTACCACAGAGCTGTGAAAAGGGGCGAGGACAGAGGAACGTACCAGTACTGGTA  58972
                ************************************************************
```

```
Dromedary      CTCCATGCCAGTACAAGTAGGAGGGAATCCAATAGAAATTCTACGAATCATAGTCAGAGC  58818
Wild           CTCCATGCCAGTACAAGTAGGAGGGAATCCAATAGAAATGCTACGAATCATAGTCAGAGC  58589
Domestic       CTCCATGCCAGTACAAGTAGGAGGGAATCCAATAGAAATGCTACGAATCATAGTCAGAGC  59032
               *************************************** ********************

Dromedary      AAACAAAAAACACTACAGAAGCACTTAGAAAGTCTATAAATATAAACATAGCAATATAAA  58878
Wild           AAACAAAAAACACTACAGAAGCACTTAGAAAGTCTATAAATATAAACATAGCAATATAAA  58649
Domestic       AAACAAAAAACACTACAGAAGCACTTAGAAAGTCTATAAATATAAACATAGCAATATAAA  59092
               ************************************************************

Dromedary      TACCTACTGAAAAAATTTGCAAGTTGAATTTTCTTTTAATTAAAAGATGTTCATTCATTTA  58938
Wild           TACCTACTGAAAAAATTTGCAAGTTGAATTTTCTTTTAATTAAAAGATGTTCATTCATTTA  58709
Domestic       TACCTACTGAAAAAATTTGCAAGTTGAATTTTCTTTTAATTAAAAGATGTTCATTCATTTA  59152
               ************************************************************

Dromedary      TTGGTTCAATTATCAGTAAACACCCTTTTGGCAACCTAATTATGTGCTAGGTATTAGACT  58998
Wild           TTGGTTCAATTATCAGTAAACACCCTTTTGGCAACCTAATTATGTGCTAGGTATTAGAC-  58768
Domestic       TTGGTTCAATTATCAGTAAACACCCTTTTGGCAACCTAATTATGTATTAGACTT------  59206
               *********************************************  ***   *

Dromedary      TGAAGCTGAAGAGACAAAAATGAGCAAAATAATTCCGTTTTTAAGGAGCTCGTAGTCTAA  59058
Wild           -GAAGCTGAAGAGACAAAAATGAGCAAAATAATTCCGTTTTTAAGGAGCTCGTAGTCTAA  58827
Domestic       -GAAGCTGAAGAGACAAAAATGAGCAAAATAATTCCGTTTTTAAGGAGCTCGTAGTCTAA  59265
                ***********************************************************

Dromedary      TGGGGGAGGGAGGAGTAGAGATGGGAAGATAAGTAAGGACACAAAAGCAAGCATTTGAGA  59118
Wild           TGGGGGAGGGAGGAGTAGAGATGGGAAGATAAGTAAGGACACAAAAGCAAGCATTTGAGA  58887
Domestic       TGGGGGAGGGAGGAGTAGAGATGGGAAGATAAGTAAGGACACAAAAGCAAGCATTTGAGA  59325
               ************************************************************

Dromedary      ATTATAATAATCTATATAATTTTAACACTAAGTCTGGATCTAGTATTTTTTTCAGTGGAA  59178
Wild           ATTATAATAATCTATATAATTTTAACACTAAGTCTGGATCTAGTATTTTTTTCAGTGGAA  58947
Domestic       ATTATAATAATCTATATAATTTTAACACTAAGTCTGGATCTAGTATTTTTTTCAGTGGAA  59385
               ************************************************************

Dromedary      AAATGAGGAAGCCAAATTACAACTTAATTTTTATTCTTTTTGTTTCAAATGTATAATGAA  59238
Wild           AAATGAGGAAGCCAAATTATAACTTAATTTTTATTCTTTTTGTTTCAAATGTATAATGAA  59007
Domestic       AAATGAGGAAGCCAAATTATAACTTAATTTTTATTCTTTTTGTTTCAAATGTATAATGAA  59445
               ******************* ****************************************

Dromedary      TAAACTAGAGTTCCCACTTTGGAAAGGAAGGAGGAGAGAAATCACATTTTGAACTCTTCC  59298
Wild           TAAACTAGAGTTCCCACTTTGGAAAGGAAGGAGGAGAGAAATCACATTTTGAAGTCTTCC  59067
Domestic       TAAACTAGAGTTCCCACTTTGGAAAGGAAGGAGGAGAGAAATCACATTTTGAAGTCTTCC  59505
               ***************************************************** ******

Dromedary      TGAATTAATCCTACCTCTGAAGGACAGTGGAATGCAGAAATTTTGTTAACTGGGACATTA  59358
Wild           TGAATTAATCCTACCTCTGAAGGACAGTGGAATGCAGAAATTTTGTTAACTGGGACATTA  59127
Domestic       TGAATTAATCCTACCTCTGAAGGACAGTGGAATGCAGAAATTTTGTTAACTGGGACATTA  59565
               ************************************************************

Dromedary      TCATAACTCACTGGCATAATATAAATGTTATCATCCCAAGACTGACCTGAAAACTCCCTA  59418
Wild           TCATAACTCACTGGCATAATATAAATGTTATCATCCCAAGACTGACCTGAAAACTCCCTA  59187
Domestic       TCATAACTCACTGGCATAATATAAATGTTATCATCCCAAGACTGACCTGAAAACTCCCTA  59625
               ************************************************************

Dromedary      GATAAATCCCTCAAGACCAGATTTCAGTCTATACCCCAAAACTGACAAATGTTGTATAAA  59478
Wild           GATAAATCCCTCAAGACCAGATTTCAGTCTATACCCCAAAACTGACAAATGTTGTATAAA  59247
Domestic       GATAAATCCCTCAAGACCAGATTTCAGTCTATACCCCAAAACTGACAAATGTTGTATAAA  59685
               ************************************************************

Dromedary      TGTACTCTAAATAACCTCCAAATTAGTAGTTGCTGATTATAATACAGTAATGTTTATATT  59538
Wild           TGTACTTTAGATAACCTCCAAATTAGTAGTTGCTGATTATAATACAGTAATGTTTATATT  59307
Domestic       TGTACTTTAGATAACCTCCAAATTAGTAGTTGCTGATTATAATACAGTAATGTTTATATT  59745
               ****** ** **************************************************

Dromedary      TCTGCTCTTGGCAATAATTATGTATCAGTAGGTAAATAAGACCATATTTGATGTCTCATT  59598
Wild           TCTGCTCTTGGCAATAATTCTGTATCAGTAGGTAAATAAGACCATATTTGATGTCTCATT  59367
Domestic       TCTGCTCTTGGCAATAATTATGTATCAGTAGGTAAATAAGACCATATTTGATGTCTCATT  59805
               ******************* ****************************************
```

```
Dromedary      AAAACCAGAGGTATTAGGAGGAGGGGACTTCAAGCTCAAAAAAGTTCAATCGAAGCCGTG  59658
Wild           AAAACCAGAGGTATTAGGAGGAGGGGACTTCAAGCTCAAAAAAGTTCAATCGAAGCCGTG  59427
Domestic       AAAACCAGAGGTATTAGGAGGAGGGGACTTCAAGCTCAAAAAAGTTCAATCGAAGCCGTG  59865
               ************************************************************

Dromedary      TAAAAATTATTACTTTGGGGCACTGTTCTCAACGTGTGATCTGGGGAATCCCTGGAGTGA  59718
Wild           TAAAAATTATTACTTTGGGGCACTGTTCTCAACGTGTGATCTGGGGAATCCCTGGAGTGA  59487
Domestic       TAAAAATTATTACTTTGGGGCACTGTTCTCAACGTGTGATCTGGGGAATCCCTGGAGTGA  59925
               ************************************************************

Dromedary      AGGCCCTTTCAGTCTGTGGAGTAAAAACTATTTTCAGAGGGGTGGGTACAGCCCAGTGGA  59778
Wild           AGGCCCTTTCAGTCTGTGGAGTAAAAACTATTTTCAGAGGGGTGGGTACAGCCCAGTGGA  59547
Domestic       AGGCCCTTTCAGTCTGTGGAGTAAAAACTATTTTCAGAGGGGTGGGTACAGCCCAGTGGA  59985
               ************************************************************

Dromedary      GAGAACATGCTTAGCGTGTACAAGCACGCACGAGGTCCTGAGTTCAATCCCCAGTACCTC  59838
Wild           GAGAACATGCTTAGCGTGTACAAGCACGCACGAGGTCCTGAGTTCAATCCCCAGTACCTC  59607
Domestic       GAGAACATGCTTAGCGTGTACAAGCACGCACGAGGTCCTGAGTTCAATCCCCAGTACCTC  60045
               ************************************************************

Dromedary      CATTAAAAAAAAAAAAAATTAAAAATGAATAAAAATAGAAACTATTTTCATAATAAAGTTA  59898
Wild           CATT-AAAAAAAAAAAATTAAAAATGAATAAAAATAGAAAC--TTTTCATAATAAAGTTA  59664
Domestic       CATT-AAAAAAAAAAAATTAAAAATGAATAAAAATAGAAAC--TTTTCATAATAAAGTTA  60102
               **** ************************************  *****************

Dromedary      AGATTTTTTTGCTACTCTCATTCTTTCTTATGCGTATAGTGGGGCTCTCCAGAGGTTCTG  59958
Wild           AGATTTTTTTGCTACTCTCATTCTTTCTTATGCGTATAGTGGGGCTCTCCAGAGGTTCTG  59724
Domestic       AGATTTTTTTGCTACTCTCATTCTTTCTTATGCGTATAGTGGGGCTCTCCAGAGGTTCTG  60162
               ************************************************************

Dromedary      GGATATGTGATGACATCATCACTCTGTCAGCCAATAGAATATAGATGTGCATATGCCTTA  60018
Wild           GGATATGTGATGACATCATCACTCTGTCAGCCAATAGAATATAGATGTGCATATGCCTTA  59784
Domestic       GGATATGTGATGACATCATCACTCTGTCAGCCAATAGAATATAGATGTGCATATGCCTTA  60222
               ************************************************************

Dromedary      TTCTAAAATTTCTCATTGTTAATTTATAATGTGTTAAATATTGATAGATAGATATGACCC  60078
Wild           TTCTAAAATTTCTCATTGTTAATTTATAATGTGTTAAATATT----GATAGATATGACCC  59840
Domestic       TTCTAAAATTTCTCATTGTTAATTTATAATGTGTTAAATATT----GATAGATATGACCC  60278
               ******************************************    **************

Dromedary      ACACAAACAAAAGCTTTTGAGGATCCTAAATAACTTTTAAGCAGAAAGGAAATCCTGAGA  60138
Wild           ACACAAACAAAAGCTTTTGAGGATCCTAAATAACTTTTAAGCAGAAAGGAAATCCTGAGA  59900
Domestic       ACACAAACAAAAGCTTTTGAGGATCCTAAATAACTTTTAAGCAGAAAGGAAATCCTGAGA  60338
               ************************************************************

Dromedary      CCAAAAGGTTTGAGAACTGCTTTAGGGGATACACCTCAAAAGAAAGGGTATACCTCATCA  60198
Wild           CCAAAAGGTTTGAGAACTGCTTTAGGGGATACACCTCAAAAGAAAGGGTATACCTCATCA  59960
Domestic       CCAAAAGGTTTGAGAACTGCTTTAGGGGATACACCTCAACAGAAAGGGTATACCTCATCA  60398
               *************************************** ********************

Dromedary      AATTTTCTCCTGTTCTACATGAAGAAATTAGACAGTAGGTTTCATGAAGACAGTGGCTGT  60258
Wild           AATTTTCTCCTGTTCTACATGAAGAAATTAGACAGTAGGTTTCATGAAGACAGTGGCTGT  60020
Domestic       AATTTTCTCCTGTTCTACATGAAGAAATTAGACAGTAGGTTTCATGAAGACAGTGGCTGT  60458
               ************************************************************

Dromedary      TTCTGTCTGGCTCATTGTTTTCTCCTCAGAATCTAGCACAGTGCCTGGCATGTAATAGGC  60318
Wild           TTCTGTCTGGCTCATTGTTTTCTCCTCAGAATCTAGCACAGTGCCTGGCATGTAATAGGC  60080
Domestic       TTCTGTCTGGCTCATTGTTTTCTCCTCAGAATCTAGCACAGTGCCTGGCATGTAATAGGC  60518
               ************************************************************

Dromedary      ATTAGAATATTTCATAAATAACAAATGAATTAATACAGTTGACACTCAAAACAGTGCAGA  60378
Wild           ATTAGAATATTTCATAAGTAACAAATGGATTAATACAGTTGACACTCAAAACAGTGCAGA  60140
Domestic       ATTAGAATATTTCATAAATAACAAATGGATTAATACAGTTGACACTCAAAACAGTGCAGA  60578
               ***************** ********* ********************************

Dromedary      GGTTAGGAGTGCCAACTGTCCTTGGAGTCAAAAATCCGTATATAACTTTATAGTTGGCTC  60438
Wild           GGTTAGGAGTGCCAACTGTCCTTGGAGTCAAAAATCCGTATATAACTTTATAGTTGGCTC  60200
Domestic       GGTTAGGAGTGCCAACTGTCCTTGGAGTCAAAAATCCGTATATAACTTTATAGTTGGCTC  60638
               ************************************************************
```

```
Dromedary      TCTGTATCCAAGCTTCTGCATCTGAGGATTTAACCAATAGCAGATGGTGTCCTACTGTTG  60498
Wild           TCTGTATCCAAGCTTCTGCATCTGAGGATTTAACCAATAGCAGATGGTGTCCTACTGTTG  60260
Domestic       TCTGTATCCAAGCTTCTGCATCTGAGGATTTAACCAATAGCAGATGGTGTCCTACTGTTG  60698
               ************************************************************

Dromedary      TATGCATTTAGTGAAAAAAATGTGTATATACATGGACCCATGCAGGTTAATCCCGTGTTG  60558
Wild           TATGCATTTAGTGAAAAAAATGTGCATATACATGGACCCATGCAGGTTAATCCCGTGTTG  60320
Domestic       TATGCATTTAGTGAAAAAAATGTGCATATACATGGACCCATGCAGGTTAATCCCGTGTTG  60758
               ************************ ***********************************

Dromedary      TCCAAGGGTCCACTGTACATACAGGAATACATTGCTATATGAAGGCTTTGCTAACAGAAT  60618
Wild           TCCAAGGGTCCACTGTACATACAGGAATACATTGCTATATGAAGGCTTTGCTAACAGAAT  60380
Domestic       TCCAAGGGTCCACTGTACATACAGGAATACATTGCTATATGAAGGCTTTGCTAACAGAAT  60818
               ************************************************************

Dromedary      TATTCAAGGTCACATAAAATGTCAGCAACAAACCCAGAACTATATTCCAGATCTCCTGTC  60678
Wild           TATTCAAGGTCACATAAAATGTCAGCAACAAACCCAGAACTATATTCCAGATCTCCTGTC  60440
Domestic       TATTCAAGGTCACATAAAATGTCAGCAACAAACCCAGAACTATATTCCAGATCTCCTGTC  60878
               ************************************************************

Dromedary      TTCTATTCCTCTACTGTTGCTCCCTGGCTTAAGGATACTTTTGTTTTTGTTACATTTTAT  60738
Wild           TTCTATTCCTCTACTGTTGCTCCCTGGCTTAAGGATACTTTTGTTTTTGTTACATTTTAT  60500
Domestic       TTCTATTCCTCTACTGTTGCTCCCTGGCTTAAGGATACTTTTGTTTTTGTTACATTTTAT  60938
               ************************************************************

Dromedary      TCTGTAACCTATCGAAAGCATGGATAATCGGATGTCACTGATGTGCGGTGATGACCTTCT  60798
Wild           TCTGTAACCTATCGAAAGCATGGATAATCGGATGTCACTGATGTGCGGTGATGACCTTCT  60560
Domestic       TCTGTAACCTATCGAAAGCATGGATAATCGGATGTCACTGATGTGCGGTGATGACCTTCT  60998
               ************************************************************

Dromedary      CATGCTGTTATGTGAGTTGAGCACCACACAGTTTTTTTCTGGTCTTGAATGTAATCAGAA  60858
Wild           CATGCTGTTATGTGAGTTGAGCACCACACAGTTTTTTTCTGGTCTTGAATGTAATCAGAA  60620
Domestic       CATGCTGTTATGTGAGTTGAGCACCACACAGTTTTTTTCTGGTCTTGAATGTAATCAGAA  61058
               ************************************************************

Dromedary      CAGTGTGATACCAAAGTGCTGTCATTATAAGGAAATGGCAAGAGTGATCAGATAAAATAT  60918
Wild           CAGTGTGATACCAAAGTGCTGTCATTATAAGGAAATGGCAAGAGTGATCAGATAAAATAT  60680
Domestic       CAGTGTGATACCAAAGTGCTGTCATTATAAGGAAATGGCAAGAGTGATCAGATAAAATAT  61118
               ************************************************************

Dromedary      AACTTTCTGGTTTTTCCCACAATAGAGTTGAATTTTTTATGATTCATTCTTATTCTAGTT  60978
Wild           AACTTTCTGGTTTTTCCCACAATAGAGTTGAATTTTTTATGATTCATTCTTATTCTAGTT  60740
Domestic       AACTTTCTGGTTTTTCCCACAATAGAGTTGAATTTTTTATGATTCATTCTTATTCTAGTT  61178
               ************************************************************

Dromedary      CCCAGTCTCCTCCTCATTTCTTTGGCCAGATCATTAAACTTCTGGTAAGCTTTTACATTA  61038
Wild           CCCAGTCTCCTCCTCATTTCTTTGGCCAGATCATTAAACTTCTGGTAAGCTTTTACATTA  60800
Domestic       CCCAGTCTCCTCCTCATTTCTTTGGCCAGATCATTAAACTTCTGGTAAGCTTTTACATTA  61238
               ************************************************************

Dromedary      ATGCACTCAGTATTCCCAGACCACAAAGAGTAAAACCATAAATGTTTTTAGGGAGATATG  61098
Wild           ATGCACTCAGTATTCCCAGACCACAAAGAGTAAAACCATAAATGTTTTTAGGGAGATATG  60860
Domestic       ATGCACTCAGTATTCCCAGACCACAAAGAGTAAAACCATAAATGTTTTTAGGGAGATATG  61298
               ************************************************************

Dromedary      GTTTATACTCTCAAAGTTGTTTTGGAACCCCTCTGCAACCCAGTATTTGTAAGAGAGATG  61158
Wild           GTTTATACTCTCAAAGTTGTTTTGGAACCCCTCTGCAACCCAGTATTTGTAAGAGAGATG  60920
Domestic       GTTTATACTCTCAAAGTTGTTTTGGAACCCCTCTGCAACCCAGTATTTGTAAGAGAGATG  61358
               ************************************************************

Dromedary      AGAGAAATTTGCTTGCTACTTTACTTATGCTTACAGAAATATAAGGCACACCCAGTACAA  61218
Wild           AGAGAAATTTGCTTGCTACTTTACTTATGCTTACAGAAATATAAGGCACACCCAGTACAA  60980
Domestic       AGAGAAATTTGCTTGCTACTTTACTTATGCTTACAGAAATATAAGGCACACCCAGTACAA  61418
               ************************************************************

Dromedary      AGAAAAAAAGGGGGAAAGAAAAATACAAGTCTTGTGAAGAGCTTCACTGGCCACCTAAAT  61278
Wild           AGAAAAAAAGGGGGAAAGAAAAATACAAGTCTTGTGAAGAGCTTCACTGGCCACCTAAAT  61040
Domestic       AGAAAAAAAGGGGGAAAGAAAAATACAAGTCTTGTGAAGAGCTTCACTGGCCACCTAAAT  61478
               ************************************************************
```

```
Dromedary     TCTGACTCCCG-CGAGAGACTGCTTTCAAAGATGGTAAACCTAAGACAAAGCCAAGTCGG  61337
Wild          TCTGACTCCCG-CGAGAGACTGCTTTCAAAGATGGTAAACCTAAGACAAAGCCAAGTCGG  61099
Domestic      TCTGACTCCCGCCGAGAGACTGCTTTCAAAGATGGTAAACCTAAGACAAAGCCAAGTCGG  61538
              *********** ************************************************

Dromedary     CAGACCATGCCCTCCCAGCCAACTAGAAGGAATTCTCTACTCACTCATCCTAATGACACA  61397
Wild          CAGACCATGCCCTCCCAGCCAACTAGAAGGAATTCTCTACTCACTCATCCTAATGACACA  61159
Domestic      CAGACCATGCCCTCCCAGCCAACTAGAAGGAATTCTCTACTCACTCATCCTAATGACACA  61598
              ************************************************************

Dromedary     GTCCTGTTTTTAGGAAAGCAGCCAAAGTGAGGCCCCCAGCAGTCAGAGCACAGTGAAGGT  61457
Wild          GTCCTGTTTTTAGGAAAGCAGCCAAAGTGAGGCCCCCAGCAGTCAGAGCACAGTGAAGGT  61219
Domestic      GTCCTGTTTTTAGGAAAGCAGCCAAAGTGAGGCCCCCAGCAGTCAGAGCACAGTGAAGGT  61658
              ************************************************************

Dromedary     TCCTACTGCTGGTGAACCTTTCTGCTCTGGGGCCTTGGGAAGGTTGCAATCACCAAAACC  61517
Wild          TCCTACTGCTGGTGAACCTTTCTGCTCTGGGGCCTTGGGAAGGTTGCAATCACCAAAACC  61279
Domestic      TCCTACTGCTGGTGAACCTTTCTGCTCTGGGGCCTTGGGAAGGTTGCAATCACCAAAACC  61718
              ************************************************************

Dromedary     TGAGAAGGAATATATGTTCTTGAAACATCTAGTGCACCTCTTAACTCATTCAATACTTAC  61577
Wild          TGAGAAGGAATATATGTTCTTGAAACATCTAGTGCACCTCTTAACTCATTCAATACTTAC  61339
Domestic      TGAGAAGGAATATATGTTCTTGAAACATCTAGTGCACCTCTTAACTCATTCAATACTTAC  61778
              ************************************************************

Dromedary     CTCTGGAGAAGTTGATTAGATACAGTTGATTCTCATTATTCACAGTAGTTAGGTTTTATC  61637
Wild          CTCTGGAGAAGTTGATTAGATACAGTTGATTCTCATTATTCACAGTAGTTAGGTTTTATC  61399
Domestic      CTCTGGAGAAGTTGATTAGATACAGTTGATTCTCATTATTCACAGTAGTTAGGTTTTATC  61838
              ************************************************************

Dromedary     AAGTCACTGCCAACACTGAATGAATGATTACTGAACCACTGCTCCTAAGGTATACACAAG  61697
Wild          AAGTCACTGCCAACACTGAATGAATGATTACTGAACCACTGCTCCTAAGGTATACACAAG  61459
Domestic      AAGTCACTGCCAACACTGAATGAATGATTACTGAACCACTGCTCCTAAGGTATACACAAG  61898
              ************************************************************

Dromedary     GTTAGGTTCCTGTGAGCCTCTGGTTACATTTCATCAACCAATCAATATATTATCTTGCTT  61757
Wild          GTTAGGTTCCTGTGAGCCTCTGGTCACATTTCATCAACCAATCAATATATTATCTTGCTT  61519
Domestic      GTTAGGTTCCTGTGAGCCTCTGGTCACATTTCATCAACCAATCAATATATTATCTTGCTT  61958
              ************************ ***********************************

Dromedary     TATCTATCTA------------TCTATCTATCTATCTATCTATCTATCTATCTATCTATC  61805
Wild          TATCTATCTATCTGTCTGTCTGTCTGTCTGTCTGTCTATCTATCTATCTATCTATCTATC  61579
Domestic      TATCTATCTATCTGTCTGTCTGTCTGTCTGTCTGTCTATCTATCTATCTATCTATCTATC  62018
              **********            *** *** *** **************************

Dromedary     TATCTTGTGTTTGTTTTAAATTATCTTATTTAATATATACTGTTGATGCATTACCATTGG  61865
Wild          TATCTTGTGTTTGTTTTAAATTATCTTATTTAATATATACTGTTGATGCATTACCATTGG  61639
Domestic      TATCTTGTGTTTGTTTTAAATTATCTTATTTAATATATACTGTTGATGCATTACCATTGG  62078
              ************************************************************

Dromedary     GTTCACAGCCAAAAGCACTGTTAACTCAAACCTAAATGAAGGTTACATAACACGTATTTG  61925
Wild          GTTCACAGCCAAAAGCACTGTTAACTCAAACCTAAATGAAGGTTACATAACACGTATTTG  61699
Domestic      GTTCACAGCCAAAAGCACTGTTAACTCAAACCTAAATGAAGGTTACATAACACGTATTTG  62138
              ************************************************************

Dromedary     TCTGTAGGGAAAATTTTTTTTTCCTAAGTCATATCAACCTTTCTTTTTACAAATTATGGT  61985
Wild          TCTGTAGGGAAAATTTTTTTTTCCTAAGTCATATCAACCTTTCTTTTTACAAATTATGGT  61759
Domestic      TCTGTAGGGAAAATTTTTTTTTCCTAAGTCATATCAACCTTTCTTTTTACAAATTATGGT  62198
              ************************************************************

Dromedary     AAAATGTACATAACATAAAATTGACCATTTAGCCATTTTTAACTACAGCTCAGTGGCATT  62045
Wild          AAAATGTACATAACATAAAATTGACCATTTAGCCATTTTTAACTACAGCTCAGTGGCATT  61819
Domestic      AAAATGTACATAACATAAAATTGACCATTTAGCCATTTTTAACTACAGCTCAGTGGCATT  62258
              ************************************************************

Dromedary     AAGTATGTTTACTTTGTTGTGCAGCCATGATCAACATCCTTCTCCAGAACTATCTGATTT  62105
Wild          AAGTATGTTTACTTTGTTGTGCAGCCATGATCAACATCCTTCTCCAGAACTATCTGATTT  61879
Domestic      AAGTATGTTTACTTTGTTGTGCAGCCATGATCAACATCCTTCTCCAGAACTATCTGATTT  62318
              ************************************************************
```

```
Dromedary      TCCCAAATTGAAAACTCTGTATCTCTGAAACAGTAGCTTTCCCCTATCCCACCCTACTCC  62165
Wild           TCCCAAATTGAAAACTCTGTATCTCTGAAACAGTAGCTTTCCCCTATCCCACCCTACTCC  61939
Domestic       TCCCAAATTGAAAACTCTGTATCTCTGAAACAGTAGCTTTCCCCTATCCCACCCTACTCC  62378
               ************************************************************

Dromedary      TCCTCCCAGGACCTGGCAACCACCATTCTACTTTCCATCACAGCCTTCCTGTGCTTTGGG  62225
Wild           TCCTCCCAGGACCTGGCAACCACCATTCTACTTTCCATCACAGCCTTCCTGTGCTTTGGA  61999
Domestic       TCCTCCCAGGACCTGGCAACCACCATTCTACTTTCCATCACAGCCTTCCTGTGCTTTGGA  62438
               ***********************************************************

Dromedary      GCACAGCACCTCAGCACTATTCTTGGGAGCCATTTTAAACAGCAGAATCACTGCCACCAC  62285
Wild           GCACAGCACCTCAGCACTATTCTTGGGAGCCATTTTAAACAGCAGAATCACTGCCACCAC  62059
Domestic       GCACAGCACCTCAGCACTATTCTTGGGAGCCATTTTAAACAGCAGAATCACTGCCACCAC  62498
               ************************************************************

Dromedary      CCCCCACCAACACACAGAAATGTGAAAAACATGGCACTATATAGACTGCAAAAAGGGCAT  62345
Wild           CCCCCACCAACACACAGAAATGTGAAAAACATGGCACTATATAGACTGCAAAAAGGGCAT  62119
Domestic       CCCCCACCAACACACAGAAATGTGAAAAACATGGCACTATATAGACTGCAAAAAGGGCAT  62558
               ************************************************************

Dromedary      TCATTTACAGTATGAGAGCTGAAACAAAGCAGAGAATTGCCTTGTTTGACCTCATTTGAA  62405
Wild           TCATTTACAGTATGAGAGCTGAAACAAAGCAGAGAATTGCCTTGTTTGACCTCAATTGAA  62179
Domestic       TCATTTACAGTATGAGAGCTGAAACAAAGCAGAGAATTGCCTTGTTTGACCTCAATTGAA  62618
               ****************************************************** *****

Dromedary      GTCCTGTGAGTTTGGAAGCCTCAGTTTCCCCTGTTCTGTGCATGTGCATGAATGACTATA  62465
Wild           GTCCTGTGAGTTTGGAAGCCTCAGTTTCCCCTGTTCTGTGCATGTGCATGAATGACTATA  62239
Domestic       GTCCTGTGAGTTTGGAAGCCTCAGTTTCCCCTGTTCTGTGCATGTGCATGAATGACTATA  62678
               ************************************************************

Dromedary      AAAGTGAATTGATTCTGGGTTAAAAATAAATTTCGGCAAGTATACAAATCTGCAAACATG  62525
Wild           AAAGTGTATTGATTCTGGGTTAAAAATAAATTTCGGCAAGTATACAAATCTGCAAACATG  62299
Domestic       AAAGTGTATTGATTCTGGGTTAAAAATAAATTTCGGCAAGTATACAAATCTGCAAACATG  62738
               ****** *****************************************************

Dromedary      GAATCCATGAATAATGAGCATCCACCGTATTTGGAAATGATGACCTTGGTTTCCTTTATT  62585
Wild           GAATCCATGAATAATGAGCATCCACCGTATTTGGAAATGATGACCTTGGTTTCCTTTATT  62359
Domestic       GAATCCATGAATAATGAGCATCCACCGTATTTGGAAATGATGACCTTGGTTTCCTTTATT  62798
               ************************************************************

Dromedary      CCAGGTGGTAAGAGTAAAGAGCTGAAAAGTGCCTTGTTAATAGGGCTGCCTTAGAACCTT  62645
Wild           CCAGGTGGTAAGAGTAAAGAGCTGAAAAGTGCCTTGTTAATAGGGCTGCCTTAGAACCTT  62419
Domestic       CCAGGTGGTAAGAGTAAAGAGCTGAAAAGTGCCTTGTTAATAGGGCTGCCTTAGAACCTT  62858
               ************************************************************

Dromedary      TTACTTGCCTCAGAGGCCTAGAGAAGTGGCCTATGTGAATTGATAATGGGCAGCTGCAAC  62705
Wild           TTACTTGCCTCAGAGGCCTAGAGAAGTGGCCTATGTGAATTGATAATTGGCAGCTGCAAC  62479
Domestic       TTACTTGCCTCAGAGGCCTAGAGAAGTGGCCTATGTGAATTGATAATTGGCAGCTGCAAC  62918
               *********************************************** ************

Dromedary      CTGAAGCAGTTCTAGTTCACCTGGAGGGTAAATATAAGCATAATCTCAAGCCCTTCTGCA  62765
Wild           CTGAAGCAGTTCTAGTTCACATGGAGGGTAAATATAAGCATAATCTCAAGCCCTTCTGCA  62539
Domestic       CTGAAGCAGTTCTAGTTCACATGGAGGGTAAATATAAGCATAATCTCAAGCCCTTCTGCA  62978
               ******************** ***************************************

Dromedary      TGAAACAAAGACCAAGCACTCAAGTACCAGTTATCAATCACTATGTGACAGGCACTATAC  62825
Wild           TGAAACAAAGACCAAGCACTCAAGTACCAGTTATCAATCACTATGTGACAGGCACTATAC  62599
Domestic       TGAAACAAAGACCAAGCACTCAAGTACCAGTTATCAATCACTATGTGACAGGCACTATAC  63038
               ************************************************************

Dromedary      TCGGCAATTTACATGCTTTATTGAATTACATCGCCCCAAAATGCTATAATGAAACTGAAG  62885
Wild           TCAGCAATTTACATGCTTTATTGAATTACATCGCCCCAAAATGCTATAATGAAACTGAAG  62659
Domestic       TCAGCAATTTACATGCTTTATTGAATTACATCGCCCCAAAATGCTATAATGAAACTGAAG  63098
               ** *********************************************************

Dromedary      GTTAGAGAAGTTAAGTATCTCATCCATTATTACATAGTTAGAACTGGCAAAGTTGAGATT  62945
Wild           GTTAGAGAAGTTAAGTATCTCATCCATTATTACATAGTTAGAACTGGCAAAGTTGAGATT  62719
Domestic       GTTAGAGAAGTTAAGTATCTCATCCATTATTACATAGTTAGAACTGGCAAAGTTGAGATT  63158
               ************************************************************
```

```
Dromedary      TGAACTCAGGTCTGTCTGACTGCAGAGCCTGAGGTCCCAACTGCTATGCAATTCTAATCA 63005
Wild           TGAACTCAGGTCTGTCTGACTGCAGAGCCTGAGGTCCCAACTGCTATGCAATTCTAATCA 62779
Domestic       TGAACTCAGGTCTGTCTGACTGCAGAGCCTGAGGTCCCAACTGCTATGCAATTCTAATCA 63218
               ************************************************************

Dromedary      AGTTTAAAAAAAGTTTGATTTACTCAGAAGTGTATAGGAGCATAAGTTATAATTACTATA 63065
Wild           AGTTTAAAAACAGTTTGATTTACTCAGAAGTGTATAGGAGCATAAGTTATAATTACTATA 62839
Domestic       AGTTTAAAAACAGTTCGATTTACTCAGAAGTGTATAGGAGCATAAGTTATAATTACTATA 63278
               ********** **** ********************************************

Dromedary      ACATTATGAAGATTTACATGTTGAAAAAGAAATTTACCAAACAAATTAAACTTAATAAGC 63125
Wild           ACATTATGAAGATTTACATGTTGAAAAAGAAATTTACCAAACAAATTAAACTTAATAAGC 62899
Domestic       ACATTATGAAGATTTACATGTTGAAAAAGAAATTTACCAAACAAATTAAACTTAATAAGC 63338
               ************************************************************

Dromedary      CTGGTCTGATATTGCTCCACAACAAAGAATATCTGAAATTCTTCAGGGCATCTGGTTTGT 63185
Wild           CTGGTCTGATATTGCTCCACAACAAAGAATATCTGAAATTCTTCAGGGCATCTGGTTTGT 62959
Domestic       CTGGTCTGATATTGCTCCACAACAAAGAATATCTGAAATTCTTCAGGGCATCTGGTTTGT 63398
               ************************************************************

Dromedary      GTCTGGTTTTCCTTAATCTTTAATGATGGGCAAATCTAATGCATTATGTAAGGCCATTTT 63245
Wild           GTCTGGTTTTCCTTAATCTTTAATGATGGGCAAATCTAATGCATTATGTAAGGCCATTTT 63019
Domestic       GTCTGGTTTTCCTTAATCTTTAATGATGGGCAAATCTAATGCATTATGTAAGGCCATTTT 63458
               ************************************************************

Dromedary      TTCTCAAGAGATGTAGATACCTCTTAAGAATTTGATGAAAATGCATTAACTTTTCAGGCT 63305
Wild           TTCTCAAGAGATGTAGATACCTCTTAAGAATTTGATGAAAATGCATTAACTTTTCAGGCT 63079
Domestic       TTCTCAAGAGATGTAGATACCTCTTAAGAATTTGATGAAAATGCATTAACTTTTCAGGCT 63518
               ************************************************************

Dromedary      ACTGAGTTGCATTTTAGTGCACTGAGGCAGTAAATTAGTGTACAATGTGCAAAAGTAGTG 63365
Wild           ACTGAGTTGCATTTTAGTGCACTGAGGCAGTAAATTAGTGTACAATGTGCAAAAGTAGTG 63139
Domestic       ACTGAGTTGCATTTTAGTGCACTGAGGCAGTAAATTAGTGTACAATGTGCAAAAGTAGTG 63578
               ************************************************************

Dromedary      ACCTAAAAAAATAAATATTTGATATGAACCACTGCATTCTCTTGGAAAAAAAAAAGTAATG 63425
Wild           ACCTAAAAAAATAAATATTTGATATGAACCACTGCATTCTCTTGGAAAAAAAAAAGTAATG 63199
Domestic       ACCTAAAAAAATAAATATTTGATATGAACCACTGCATTCTCTTGGAAAAAAAAAAGTAATG 63638
               ************************************************************

Dromedary      GGTCAACTCTCTTAGGAGTCCTTAGCTTCCCAAAAAGGAGTAGGAAGAATAATCTCCTGT 63485
Wild           GGTCAACTCTCTTAGGAGTCCTTAGCTTCCCAAAAAGGAGTAGGAAGAATAATCTCCTGT 63259
Domestic       GGTCAACTCTCTTAGGAGTCCTTAGCTTCCCAAAAAGGAGTAGGAAGAATAATCTCCTGT 63698
               ************************************************************

Dromedary      GGCCTGGAAACAGCTTCTGTTTCTCGCTGGCTATGTTTGTTTAGCTCTTTAATAGTTCAT 63545
Wild           GGCCTGGAAACAGCTTCTGTTTCTCGCTGGCTATGTTTGTTTAGCTCTTTAATAGTTCAT 63319
Domestic       GGCCTGGAAACAGCTTCTGTTTCTCGCTGGCTATGTTTGTTTAGCTCTTTAATAGTTCAT 63758
               ************************************************************

Dromedary      TTGATTAGATCTTGTGGCTCCCAAAGCTAAGGTTCAGAGTTTGATCCCTACAGAGGCCAC 63605
Wild           TTGATTAGATCTTGTGGCTCCCAAAGCTAAGGTTCAGAGTTTGATCCCTACAGAGGCCAC 63379
Domestic       TTGATTAGATCTTGTGGCTCCCAAAGCTAAGGTTCAGAGTTTGATCCCTACAGAGGCCAC 63818
               ************************************************************

Dromedary      TTAAATTTAGAGAACAAAAAGCTCTATTCTCTGCTCCCAGACCTCACCCCAAATCCCTGC 63665
Wild           TTAAATTTAGAGAACAAAAAGCTCTATTCTCTGCTCCCAGACCTCACCCCAAATCCCTGC 63439
Domestic       TTAAATTTAGAGAACAAAAAGCTCTATTCTCTGCTCCCAGACCTCACCCCAAATCCCTGC 63878
               ************************************************************

Dromedary      CAGGTGTCTGCCCTCTGGTCAAATGAGAAGCTGGCAAAGGGGTGCAAACCTATCACAGTG 63725
Wild           CAGGTGTCTGCCCTCTGGTCAAATGAGAAGCTGGCAAAGGGGTGCAAACCTATCACAGTG 63499
Domestic       CAGGTGTCTGCCCTCTGGTCAAATGAGAAGCTGGCAAAGGGGTGCAAACCTATCACAGTG 63938
               ************************************************************

Dromedary      TAGGCAAACAGAAAAAGGGGCACCCTTCATTATGGTGCTGCTTCCCTTGTATGTGCTTAC 63785
Wild           TAGGCAAACAGAAAAAGGGGCACCCTTCATTATGGTGCTGCTTCCCTTGTATGTGCTTAC 63559
Domestic       TAGGCAAACAGAAAAAGGGGCACCCTTCATTATGGTGCTGCTTCCCTTGTATGTGCTTAC 63998
               ************************************************************
```

```
Dromedary      AATATTTGGATATAACTTACAGAGAATAGAGCCTACATTTTTAACTCTTACCACTGGAAA  63845
Wild           AATATTTGGATATAACTTACAGAGAATAGAGCTTACATTTTTAACTCTTACCACTGGAAA  63619
Domestic       AATATTTGGATATAACTTACATAGAATAGAGCCTACATTTTTAACTCTTACCACTGGAAA  64058
               ********************* ********** ***************************

Dromedary      TCTGAGGCAAACTTCATTACCAGTCATAAAATTCATTGTCTTCTCAGTTATTCTAAGCTT  63905
Wild           TCTGAGGCAAACTTCATTACCAGTCATAAAATTCATTGTCTTCTCAGTTATTCTAAGCTT  63679
Domestic       TCTGAGGCAAACTTCATTACCAGTCATAAAATTCATTGTCTTCTCAGTTATTCTAAGCTT  64118
               ************************************************************

Dromedary      ATTCTAAATTCAGGGAGCTGACATAATCCTCTTGGTAATAAACAATGAAAAACACATCTT  63965
Wild           ATTCTAAATTCAGGGAGCTGACATAATCCTCTTGGTAATAAACAATGAAAAACACATCTT  63739
Domestic       ATTCTAAATTCAGGGAGCTGACATAATCCTCTTGGTAATAAACAATGAAAAACACATCTT  64178
               ************************************************************

Dromedary      CTGAGCAACATTAATCTGCAGCTTTAGGACAGGAAATAACTTAATAGTAATCAGTCAAAA  64025
Wild           CTGAGCAACATTAATCTGCAGCTTTAGGACAGGAAATAACTTAATAGTAATCAGTCAAAA  63799
Domestic       CTGAGCAACATTAATCTGCAGCTTTAGGACAGGAAATAACTTAATAGTAATCAGTCAAAA  64238
               ************************************************************

Dromedary      GTTGAGCACAATCTTCACATAAATAAAAGATATTAAATTATTTAAAAATAATTCTATGTG  64085
Wild           ATTGAGCACAATCTTCACATAAATAAAAGATATTAAATTATTTAAAAATAATTCTATGTG  63859
Domestic       ATTGAGCACAATCTTCACATAAATAAAAGATATTAAATTATTTAAAAATAATTCTATGTG  64298
                ***********************************************************

Dromedary      CAATATAGCATTAGGATCAGTATGATTTTCATCATGTGCTAAGAATTTAGACAGGCAAAT  64145
Wild           CAATATAGCATTAGGATCAGTATGATTTTCATCATGTGCTAAGAATTTAGACAGGCAAAT  63919
Domestic       CAATATAGCATTAGGATCAGTATGATTTTCATCATGTGCTAAGAATTTAGACAGGCAAAT  64358
               ************************************************************

Dromedary      GAGTTTCTCAAATCATAGCTGAAAATATTTTACTAGTATTACAATCTTTTCAAATTCAGG  64205
Wild           GAGTTTCTCAAATCATAGCTGAAAATATTTTACTAGTATTACAATCTTTTCAAATTCAGG  63979
Domestic       GAGTTTCTCAAATCATAGCTGAAAATATTTTACTAGTATTACAATCTTTTCAAATTCAGG  64418
               ************************************************************

Dromedary      ACTTCCTAATTTAAATCTTTCCTAATTTAAATCTAAATATTTCCCTAATCACACAGAACT  64265
Wild           ACTTCCTAATTTAAATCTTTCCTAATTTAAATCTAAATATTTCCCTAATCACACAGAACT  64039
Domestic       AC----------------TTCCTAATTTAAATCTAAATATTTCCCTAATCACACAGAACT  64462
               **                ******************************************

Dromedary      AAAAATAATTTAAGACAGCAAATAAAATTCTTTTTACTTCAAATGTTTGCCTAAATAATA  64325
Wild           AAAAATAATTTAAGACAGCAAATAAAATTCTTTTTACTTCAAATGTTTGCCTAAATAATA  64099
Domestic       AAAAATAATTTAAGACAGCAAATAAAATTCTTTTTACTTCAAATGTTTGCCTAAATAATA  64522
               ************************************************************

Dromedary      TAAAATCATTTTATTTTTGGAGG-AAAAAAAATTTCAACTTTTAAAGTACGAAGTGTAAA  64384
Wild           TAAAATCATTTTATTTTTGGAGGAAAAAAAAATTTCAACTTTTAAAGTACGAAGTGTAAA  64159
Domestic       TAAAATCATTTTATTTTTGGAGGAAAAAAAAATTTCAACTTTTAAAGTACGAAGTGTAAA  64582
               *********************** ************************************

Dromedary      TTAAGATTTACTTAGATTATAATTTTAAAATTTCCCACATAAAGATTAATAAGATTTAAA  64444
Wild           TTAAGATTTACTTAGATTGTAATTTTAAAATTTCCCACATAAAGATTAATAAGATTTAAA  64219
Domestic       TTAAGATTTACTTAGATTGTAATTTTAAAATTTCCCACATAAAGATTAATAAGATTTAAA  64642
               ****************** *****************************************

Dromedary      TGTAGTTTATATTAGTGTTAACATAGATTTTAATTTTTCAAATGTCACATAGATATTTCA  64504
Wild           TGTAGTTTATATTAGTGTTAACATAGATTTTAATTTTTCAAATGTCACATAGATATTTCA  64279
Domestic       TGTAGTTTATATTAGTGTTAACATAGATTTTAATTTTTCAAATGTCACATAGATATTTCA  64702
               ************************************************************

Dromedary      TTATTTGTAGATTTATTTCTTTTATGAAGTAGTCAAATGAATCAGCTCACCCTTGACTGT  64564
Wild           TTATTTGTAGATTTATTTCTTTTATGAAGTAGTCAAATGAATCAGCTCACCCTTGACTGT  64339
Domestic       TTATTTGTAGATTTATTTCTTTTATGAAGTAGTCAAATGAATCAGCTCACCCTTGACTGT  64762
               ************************************************************

Dromedary      AACAAAATACTGTTTGGTGACTTGTGACAGACAGGGTTTTAACCTCTGACAGCGAGATTC  64624
Wild           AACAAAATACTGTTTGGTGACTTGTGACAGACAGGGTTTTAACCTCTGACAGCGAGATTC  64399
Domestic       AACAAAATACTGTTTGGTGACTTGTGACAGACAGGGTTTTAACCTCTGACAGCGAGATTC  64822
               ************************************************************
```

```
Dromedary      ATTGTGGAGCAAGAGCCAATCATAGATCCTGACGACACTTGTCTCATCAAAGTTGGAATA  64684
Wild           ATTGTGGAGCAAGAGCCAATCATAGATCCTGACGACACTTGTCTCATCAAAGTTGGAATA  64459
Domestic       ATTGTGGAGCAAGAGCCAATCATAGATCCTGACGACACTTGTCTCATCAAAGTTGGAATA  64882
               ************************************************************

Dromedary      TAAAAAGCCACTTGGAATACAGTATAAAAGATTCACTGGTGTGGCAAGTTGTCTCTCAGG  64744
Wild           TAAAAAGCCACTTGGAATACAGTATAAAAGATTCACTGGTGTGGCAAGTTGTCTCTCAGG  64519
Domestic       TAAAAAGCCACTTGGAATACAGTATAAAAGATTCACTGGTGTGGCAAGTTGTCTCTCAGG  64942
               ************************************************************

Dromedary      CTGTGCAGGCATTAAAATTTTGCTTGGCATTACTCAAAAGCAAAAGAAAAATAAAAGGAA  64804
Wild           CTGTGCAGGCATTAAAATTTTCCTTGGCATTACTCAAAAGCAAAAGAAAAATAAAAGGAA  64579
Domestic       CTGTGCAGGCATTAAAATTTTCCTTGGCATTACTCAAAAGCAAAAGAAAAATAAAAGGAA  65002
               ********************* **************************************

Dromedary      GAAATAAGAACAAGGGAAAAGATTGTCTTGATTTTAAAATCATGCAAAAACTGCAAATCT  64864
Wild           GAAATAAGAACAAGGGAAAAGATTGTCTTGATTTTAAAATCATGCAAAAACTGCAAATCT  64639
Domestic       GAAATAAGAACAAGGGAAAAGATTGTCTTGATTTTAAAATCATGCAAAAACTGCAAATCT  65062
               ************************************************************

Dromedary      ATGTTTATATTTACCTGTTTATGCTGATTGTTGCTGGTCCAGTGGATCTGAATGAGAACA  64924
Wild           ATGTTTATATTTACCTGTTTATGCTGATTGTTGCTGGTCCAGTGGATCTGAATGAGAACA  64699
Domestic       ATGTTTATATTTACCTGTTTATGCTGATTGTTGCTGGTCCAGTGGATCTGAATGAGAACA  65122
               ************************************************************

Dromedary      ACGAACAAAAAGAAAATGTGGAAAAAGAGGGGCTGTGTAATGCATGTATGTGGAGACAAA  64984
Wild           ACGAACAAAAAGAAAATGTGGAAAAAGAGGGGCTGTGTAATGCATGTATGTGGAGACAAA  64759
Domestic       ACGAACAAAAAGAAAATGTGGAAAAAGAGGGGCTGTGTAATGCATGTATGTGGAGACAAA  65182
               ************************************************************

Dromedary      ACACTAAATCTTCAAGACTAGAAGCTATAAAAATTCAAATCCTCAGTAAACTTCGCCTGG  65044
Wild           ACACTAAATCTTCAAGACTAGAAGCTATAAAAATTCAAATCCTCAGTAAACTTCGCCTGG  64819
Domestic       ACACTAAATCTTCAAGACTAGAAGCTATAAAAATTCAAATCCTCAGTAAACTTCGCCTGG  65242
               ************************************************************

Dromedary      AAACAGCTCCTAACATCAGCAAAGATGCTATAAGACAACTTTTGCCCAAAGCTCCTCCGC  65104
Wild           AAACAGCTCCTAACATCAGCAAAGATGCTATAAGACAACTTTTGCCCAAAGCTCCTCCAC  64879
Domestic       AAACAGCTCCTAACATCAGCAAAGATGCTATAAGACAACTTTTGCCCAAAGCTCCTCCAC  65302
               ********************************************************** *

Dromedary      TCCGGGAACTGATTGATCAGTACGATGTCCAGAGAGATGACAGCAGTGATGGCTCCTTGG  65164
Wild           TCCGGGAACTGATTGATCAGTACGATGTCCAGAGAGATGACAGCAGTGATGGCTCCTTGG  64939
Domestic       TCCGGGAACTGATTGATCAGTACGATGTCCAGAGAGATGACAGCAGTGATGGCTCCTTGG  65362
               ************************************************************

Dromedary      AAGATGATGATTACCACGCTACGACGGAAACAATCATTACCATGCCTACAGAGTGTAAGT  65224
Wild           AAGATGATGATTACCACGCTACGACGGAAACAATCATTACCATGCCTACAGAGTGTAAGT  64999
Domestic       AAGATGATGATTACCACGCTACGACGGAAACAATCATTACCATGCCTACAGAGTGTAAGT  65422
               ************************************************************

Dromedary      AGTCCTATTAGTGTATATCAACAATTCTGCTGACTGTTGTTCTAGTGTTTATGAGAAACA  65284
Wild           AGTCCTATTAGTGTATATCAACAATTCTGCTGACTGTTGTTCTAGTGTTTATGAGAAACA  65059
Domestic       AGTCCTATTAGTGTATATCAACAATTCTGCTGACTGTTGTTCTAGTGTTTATGAGAAACA  65482
               ************************************************************

Dromedary      GATCTATTTTCAGGCTCTTTTAACAGGCTGTTCGTGTGTATGTAAGTAGGAGGGAAAAGA  65344
Wild           GATCTATTTTCAGGCTCTTTTAACAGGCTGTTCGTGTGTATGTAAGTAGGAGGGAAAAGA  65119
Domestic       GATCTATTTTCAGGCTCTTTTAACAGGCTGTTCGTGTGTATGTAAGTAGGAGGGAAAAGA  65542
               ************************************************************

Dromedary      GTTTCATTTTTCAAGATTTCATGAGAAATCTAATGAGACTGAGAGCTGCTGCATTATATA  65404
Wild           GTTTCATTTTTCAAGATTTCATGAGAAATCTAATGAGACTGAGAGCTGCTGCATTATATA  65179
Domestic       GTTTCATTTTTCAAGATTTCATGAGAAATCTAATGAGACTGAGAGCTGCTGCATTATATA  65602
               ************************************************************

Dromedary      TTCCCTGAGGGAGCTAAAAAGCTAAAAATAAAAAATAAAATGCTTGCATAGCATTAATAT  65464
Wild           TTCCCTGAGGGAGCTAAAAAGCTAAAAATAAAAAATAAAATGCTTGCATAGCATTAATAT  65239
Domestic       TTCCCTGAGGGAGCTAAAAAGCTAAAAATAAAAAATAAAATGCTTGCATAGCATTAATAT  65662
               ************************************************************
```

```
Dromedary      TATATAGTTTAGCATGACCAATATGCTTATGTTTTCACAGCTTAATACCACCAAGGCAAG  65524
Wild           TATATAGTTTAGCATGACCAATATGCTTATGTTTTCACAGCTTAATACCACCAAGGCAAG  65299
Domestic       TATATAGTTTAGCATGACCAATATGCTTATGTTTTCACAGCTTAATACCACCAAGGCAAG  65722
               ************************************************************

Dromedary      GATTGTAAGATACTGCAAACAATGTGAAAAACACATGAAATTTGTAACTGCAGTTAGTTA  65584
Wild           GATTGTAAGATACTGCAAACAATGTGAAAAACACATGAAATTTGTAACTGCAGTTAGTTA  65359
Domestic       GATTGTAAGATACTGCAAACAATGTGAAAAACACATGAAATTTGTAACTGCAGTTAGTTA  65782
               ************************************************************

Dromedary      TCTGAAATATGCATTTATAACAGCAGACTTTTTGACTAATAACAGAGAAAGGAAGAAGTT  65644
Wild           TCTGAAATATGCATTTATAACAGCAGACTTTTTGACTAATAACAGAGAAAGGAAGAAGTT  65419
Domestic       TCTGAAATATGCATTTATAACAGCAGACTTTTTGACTAATAACAGAGAAAGGAAGAAGTT  65842
               ************************************************************

Dromedary      TGTAGATGTTGAAACCTATTTGGGCATTTGCTGACAACCTAGAATGACTTCTGTTATTCA  65704
Wild           TGTAGATGTTGAAACCTATTTGGGCATTTGCTGACAACCTAGAATGACTTCTGTTATTCA  65479
Domestic       TGTAGATGTTGAAACCTATTTGGGCATTTGCTGACAACCTAGAATGACTTCTGTTATTCA  65902
               ************************************************************

Dromedary      AAACTATTTCTCACAGTGTTTTTATGTTCTTCACAAATTACAATATCTAATTTTGAAAGC  65764
Wild           AAACTATTTCTCACAGTGTTTTTATGTTCTTCACAAATTACAATATCTAATTTTGAAAGC  65539
Domestic       AAACTATTTCTCACAGTGTTTTTATGTTCTTCACAAATTACAATATCTAATTTTGAAAGC  65962
               ************************************************************

Dromedary      TATTACACTGGAAAGTATAAAAATATTTTTAAAAAATTTAATGTATTAGTAAGAGCAATG  65824
Wild           TATTACACTGGAAAGTATAAAAATATTTTTAAAAAATTTAATGTATTAGTAAGAGCAATG  65599
Domestic       TATTACACTGGAAAGTATAAAAATATTTTTAAAAAATTTAATGTATTAGTAAGAGCAATG  66022
               ************************************************************

Dromedary      ATGAAGTAAACATAGCATAATAGAAATCATGAGCTAATGATTAGAAAATGCCAAGAAATA  65884
Wild           ATGAAGTAAACATAGCATAATAGAAATCATGAGCTAATGATTAGAAAATGCCAAGAAATA  65659
Domestic       ATGAAGTAAACATAGCATAATAGAAATCATGAGCTAATGATTAGAAAATGCCAAGAAATA  66082
               ************************************************************

Dromedary      AACATTTTAATCAAATAGGTTATGGCTCACAAAGTCCTACTTATACCTTGGCCATGGTGC  65944
Wild           AACATTTTAATCAAATAGGTTATGGCTCACAAAGTCCTACTTATACCTTGGCCATGGTGC  65719
Domestic       AACATTTTAATCAAATAGGTTATGGCTCACAAAGTCCTACTTATACCTTGGCCATGGTGC  66142
               ************************************************************

Dromedary      TATTGTTGAGAATACCCGGTCTGCAGATTTCCAGGCAGGCACACTGCTTAATAACCTTCT  66004
Wild           TATTGTTGAGAATACCCGGTCTGCACATTTCCAGGCAGGCACACTGCTTAATAACCTTCT  65779
Domestic       TATTGTTGAGAATACCCGGTCTGCACATTTCCAGGCAGGCACACTGCTTAATAACCTTCT  66202
               ************************* **********************************

Dromedary      AAAATATGATTTTATTATTCATGGAAGGGAGGACTGTTACTTGCAGTAGCTACATTTCTT  66064
Wild           AAAATATGATTTTATTATTCATGGAAGGGAGGACTGTTACTTGCAGTAGCTACATTTCTT  65839
Domestic       AAAATATGATTTTATTATTCATGGAAGGGAGGACTGTTACTTGCAGTAGCTACATTTCTT  66262
               ************************************************************

Dromedary      CTGAAAGATAATATATTTCATATCTTTCCTGTTGCAGTCAGTTGAAAACTATACTCAAGG  66124
Wild           CTGAAAGATAATATATTTCATATCTTTCCTGTTGCAGTCAGTTGAAAACTATACTCAAGG  65899
Domestic       CTGAAAGATAATATATTTCATATCTTTCCTGTTGCAGTCAGTTGAAAACTATACTCAAGG  66322
               ************************************************************

Dromedary      AAAGGGAGACAGGCACCTTAACAGAGAAGGCATGACAAGAAAGATTTTTGTGCCATGTGT  66184
Wild           AAAGGGAGACAGGCACCTTAACAGAGAAGGCATGACAAGAAAGATTTTTGTGCCATGTGT  65959
Domestic       AAAGGGAGACAGGCACCTTAACAGAGAAGGCATGACAAGAAAGATTTTTGTGCCATGTGT  66382
               ************************************************************

Dromedary      CTGTGATCTTGCTTTATCCACTTTAAACTGGACACAAAACAGTTTCAAAGTATTGTCTTT  66244
Wild           CTGTGATCTTGCTTTATCCACTTTAAACTGGACACAAAACAGTTTCAAAGTATTGTCTTT  66019
Domestic       CTGTGATCTTGCTTTATCCACTTTAAACTGGACACAAAACAGTTTCAAAGTATTGTCTTT  66442
               ************************************************************

Dromedary      CTTATTAAGTAATTAGGTTATAATGCAACAAATAATTTTCCTTTAAGACTGTGCTATCAG  66304
Wild           CTTATTAAGTAATTAGGTTATAATGCAACAAATAATTTTCCTTTAAGACTGTGCTATCAG  66079
Domestic       CTTATTAAGTAATTAGGTTATAATGCAACAAATAATTTTCCTTTAAGACTGTGCTATCAG  66502
               ************************************************************
```

```
Dromedary      ATAGTCCTGGAGTAGATCTGCCTTATTTATAAATAATCATGGAAAACCAAAAGGAAAGAA  66364
Wild           ATAGTCCTGGAGTAGATCTGCCTTATTTATAAATAATCATGGAAAACCAAAAGGAAAGAA  66139
Domestic       ATAGTCCTGGAGTAGATCTGCCTTATTTATAAATAATCATGGAAAACCAAAAGGAAAGAA  66562
               ************************************************************

Dromedary      ATTTTTAAGTGTTTCTCCTTACAATGACAGCCTGCCCCTAAAGACAATGTTCTCTAACTT  66424
Wild           ATTTTTAAGTGTTTCTCCTTACAATGACAGCCTGCCCCTAAAGACAATGTTCTCTAACTT  66199
Domestic       ATTTTTAAGTGTTTCTCCTTACAATGACAGCCTGCCCCTAAAGACAATGTTCTCTAACTT  66622
               ************************************************************

Dromedary      TTGAGATAGCCTGAATGCGACATTTAAATTTTGGTATTAATTAGCTGCTAGTTTTGTTCC  66484
Wild           TTGAGATAGCCTGAATGCGACATTTAAATTTTGGTGCTAATTAGCTGCTAGTTTTGTTCC  66259
Domestic       TTGAGATAGCCTGAATGCGACATTTAAATTTTGGTGCTAATTAGCTGCTAGTTTTGTTCC  66682
               ***********************************  ***********************

Dromedary      TTTCAAAGGCTATCCCAGCGCCAAAGCACAACAGATGTACTATATTTTCTACTAATTCCC  66544
Wild           TTTAAAAGGCTATCCCAGCGCCAAAGCACAACAGATGTACTATATTTTCTACTAATTCCC  66319
Domestic       TTTAAAAGGCTATCCCAGCGCCAAAGCACAACAGATGTACTATATTTTCTACTAATTCCC  66742
               *** ********************************************************

Dromedary      GTTAGTTGCTCAGTGTCTTGTCCCCAGGTAATTCAGGCCTGGGGGAAGGGTTCCTTCTTC  66604
Wild           GTTAGTTGCTCAGTGTCTTGTCCCCAGGTAATTCAGGCCTGGGGGAAGGGTTCCTTCTTC  66379
Domestic       GTTAGTTGCTCAGTGTCTTGTCCCCAGGTAATTCAGGCCTGGGGGAAGGGTTCCTTCTTC  66802
               ************************************************************

Dromedary      CAGACTGATTGGTACAGCTGCTCAGTAAGTGTAACTACTCAGATTCCCAAAGAATTCTAA  66664
Wild           CAGACTGATTGGTACAGCTGCTCAGTAAGTGTAACTACTCAGATTCCCAAAGAATTCTAA  66439
Domestic       CAGACTGATTGGTACAGCTGCTCAGTAAGTGTAACTACTCAGATTCCCAAAGAATTCTAA  66862
               ************************************************************

Dromedary      GTGGATGTTCCTCCACGGTGTCTCTTGTTCTCTCTAATCATCATCATTTTAAAATTTCGT  66724
Wild           GTGGATGTTCCTCCACGGTGTCTCTTGTTCTCTCTAATCATCATCATTTTAAAATTTCGT  66499
Domestic       GTGGATGTTCCTCCACGGTGTCTCTTGTTCTCTCTAATCATCATCATTTTAAAATTTCGT  66922
               ************************************************************

Dromedary      CCACTGTTCATTCCTTCATAGAATTTTCCTTAGTTCACAGTTCTCTGGAAAGGAAGCAGG  66784
Wild           CCACTGTTCATTCCTTCATAGAATTTTCCTTAGTTCACAGTTCTCTGGAAAGGAAGTAGG  66559
Domestic       CCACTGTTCATTCCTTCATAGAATTTTCCTTAGTTCACAGTTCTCTGGAAAGGAAGTAGG  66982
               ******************************************************** ***

Dromedary      TTCCTCATAAACAGCTGAAAAAAAAATAGAGCCAAAAATTCTAAACAGCTATAGTAGCTAT  66844
Wild           TTCCTCATAAACAGCTGAAAAAAAAATAGAGCCAAAAATTCTAAACAGCTATAGTAGCTAT  66619
Domestic       TTCCTCATAAACAGCTGAAAAAAAAATAGAGCCAAAAATTCTAAACAGCTATAGTAGCTAT  67042
               ************************************************************

Dromedary      TTTGTTTGA-TTTTTTTTTGAGTTATGAATGAAATTCTACATAGTTTTTCACTTAAGAAG  66903
Wild           TTTGTTTGATTTTTTTTTTTGAGTTATGAATGAAATTCTACATAGTTTTTCACTTAAGAAG  66679
Domestic       TTTGTTTGATTTTTTTTTTTGAGTTATGAATGAAATTCTACATAGTTTTTCACTTAAGAAG  67102
               ********* **************************************************

Dromedary      ACTAATAAGTATTTATTATTCAATAGAAAAATGCTCACTGATTAATATGGAGGGGTTTGT  66963
Wild           ACTAATAAGTATTTATTATTCAATAGAAAAATGCTCACTGATTAATATGGAGGGGTTTGT  66739
Domestic       ACTAATAAGTATTTATTATTCAATAGAAAAATGCTCACTGATTAATATGGAGGGGTTTGT  67162
               ************************************************************

Dromedary      TCATTTTCATGAAAAATAATCTCAATAACTCTTTTCTTTTCTTATTCATTTATAAGCTGA  67023
Wild           TCATTTTCATGAAAAATAATCTCAATAACTCTTTTCTTTTCTTATTCATTTATAAGCTGA  66799
Domestic       TCATTTTCATGAAAAATAATCTCAATAACTCTTTTCTTTTCTTATTCATTTATAAGCTGA  67222
               ************************************************************

Dromedary      TCTTCTAATGCAAGTAGAAGGAAAACCCAAATGTTGCTTCTTTAAGTTTAGCTCTAAAAT  67083
Wild           TCTTCTAATGCAAGTAGAAGGAAAACCCAAATGTTGCTTCTTTAAGTTTAGCTCTAAAAT  66859
Domestic       TCTTCTAATGCAAGTAGAAGGAAAACCCAAATGTTGCTTCTTTAAGTTTAGCTCTAAAAT  67282
               ************************************************************

Dromedary      ACAATACAATAAAGTAGTAAAGGCCCAATTGTGGATCTATCTGAGACCCGTACAGACTCC  67143
Wild           ACAATACAATAAAGTAGTAAAGGCCCAATTGTGGATCTATCTGAGACCCGTCCAGACTCC  66919
Domestic       ACAATACAATAAAGTAGTAAAGGCCCAATTGTGGATCTATCTGAGACCCGTCCAGACTCC  67342
               *************************************************** ********
```

```
Dromedary       TACAACAGTGTTTGTGCAAATCCTGAGACTCATCAAACCCATGAAAGACGGTACAAGGTA  67203
Wild            TACAACAGTGTTTGTGCAAATCCTGAGACTCATCAAACCCATGAAAGACGGTACAAGGTA  66979
Domestic        TACAACAGTGTTTGTGCAAATCCTGAGACTCATCAAACCCATGAAAGACGGTACAAGGTA  67402
                ************************************************************

Dromedary       TACTGGAATCCGATCTCTGAAACTTGACATGAACCCAGGCACTGGTATTTGGCAGAGCAT  67263
Wild            TACTGGAATCCGATCTCTGAAACTTGACATGAACCCAGGCACTGGTATTTGGCAGAGCAT  67039
Domestic        TACTGGAATCCGATCTCTGAAACTTGACATGAACCCAGGCACTGGTATTTGGCAGAGCAT  67462
                ************************************************************

Dromedary       TGATGTGAAGACAGTGTTGCAAAATTGGCTCAAACAACCTGAATCCAACTTAGGCATTGA  67323
Wild            TGATGTGAAGACAGTGTTGCAAAATTGGCTCAAACAACCTGAATCCAACTTAGGCATTGA  67099
Domestic        TGATGTGAAGACAGTGTTGCAAAATTGGCTCAAACAACCTGAATCCAACTTAGGCATTGA  67522
                ************************************************************

Dromedary       AATCAAAGCTTTAGATGAGAATGGTCATGATCTTGCTGTAACCTTCCCAGGACCAGGAGA  67383
Wild            AATCAAAGCTTTAGATGAGAATGGTCATGATCTTGCTGTAACCTTCCCAGGACCAGGAGA  67159
Domestic        AATCAAAGCTTTAGATGAGAATGGTCATGATCTTGCTGTAACCTTCCCAGGACCAGGAGA  67582
                ************************************************************

Dromedary       AGATGGTTTGGTAAGTGATAACTGAAAATAACACTCTAAAAACCTTGTCATGTTTTTATT  67443
Wild            AGATGGTTTGGTAAGTGATAACTGAAAATAACACTCTAAAAACCTTGTCATGTTTTTATT  67219
Domestic        AGATGGTTTGGTAAGTGATAACTGAAAATAACACTCTAAAAACCTTGTCATGTTTTTATT  67642
                ************************************************************

Dromedary       CATAATGTGAATGAATAGTAGTGGAAAATAACTACCAGTTTCCTATGCTCATAAGCCAGA  67503
Wild            CATAATGTGAATGAATAGTAGTGGAAAATAACTACCAGTTTCCTATGCTCATAAGCCAGA  67279
Domestic        CATAATGTGAATGAATAGTAGTGGAAAATAACTACCAGTTTCCTATGCTCATAAGCCAGA  67702
                ************************************************************

Dromedary       CAAAGATAACTTACCCCAATGGTAGCCCTGTACCCAATAAAAGTAGGTGTTTAGTTTCAT  67563
Wild            CAAAGATAACTTACCCCAATGGTAGCCCTGTACCCAATAAAAGTAGGTGTTTAGTTTCAT  67339
Domestic        CAAAGATAACTTACCCCAATGGTAGCCCTGTACCCAATAAAAGTAGGTGTTTAGTTTCAT  67762
                ************************************************************

Dromedary       ATCCTATGAAACACCCTCTTGATACTTTTACTTTGCATGAGGATTTAAAAGAAAAAAGTT  67623
Wild            ATCCTATGAAACACCCTCTTGATACTTTTACTTTGCATGAGGATTTAAAAGAAAAAAGTT  67399
Domestic        ATCCTATGAAACACCCTCTTGATACTTTTACTTTGCATGAGGATTTAAAAGAAAAAAGTT  67822
                ************************************************************

Dromedary       ATAACACAGTCCTTAACTTCTAAGGGAATTTTTTTTGAATTGGGAATGAAATATAAAATG  67683
Wild            ATAACACAGTCCTTAACTTCTAAGGGAA-TTTTTTTGAATTGGGAATGAAATATAAAATG  67458
Domestic        ATAACACAGTCCTTAACTTCTAAGGGAA-TTTTTTTGAATTGGGAATGAAATATAAAATG  67881
                **************************** *******************************

Dromedary       CTTTTTCATTGATATGCACTACGGTCATATGAATAAAAACATGAAATCTTCATAGTAGAT  67743
Wild            CTTTTTCATTGATATGCACTACGGTCATATGAATAAAAACATGAAATCTTCATAGTAGAT  67518
Domestic        CTTTTTCATTGATATGCACTACGGTCATATGAATAAAAACATGAAATCTTCATAGTAGAT  67941
                ************************************************************

Dromedary       TCTAGTACATATTCAACAAAACATTTTTTCCCTAGAAGAGTATCAAATGTGTTAAAATTT  67803
Wild            TCTAGTACATATTCAACAAAACATTTTTTCCCTAGAAGAGTATCAAATGTGTTAAAATTT  67578
Domestic        TCTAGTACATATTCAACAAAACATTTTTTCCCTAGAAGAGTATCAAATGTGTTAAAATTT  68001
                ************************************************************

Dromedary       TTTTTGGCTTAATAGGGCAGGAAAAAAACCTCTAAAATTATAATTAAAATAAAAAACTTT  67863
Wild            TTTTTGGCTTAATAGGGCAGGAAAAAAACCTCTAAAATTATAATTAAAATAAAAAACTTT  67638
Domestic        TTTTTGGCTTAATAGGGCAGGAAAAAAACCTCTAAAATTATAATTAAAATAAAAAACTTT  68061
                ************************************************************

Dromedary       TATTTATAGCAATTAACTATAATATGTTTAGGCTTATATATTATTAAATATATATTTAAG  67923
Wild            TATTTATAGCAATTAACTATAATATGTTTNNNNN--NNNNNNNNNAATATATATTTAAG  67696
Domestic        TATTTATAGCAATTAACTATAATATGTTTAGCCTTATATATTATTAAATATATATTTAAG  68121
                *****************************                 **************

Dromedary       ATCTCTCATGATAAATATGTTCATTATTCTGTAGGGTGTTGATGCAATAATGTATATGTA  67983
Wild            ATCTCTCATGATAAATATGTTCATTATTCTGTAGGGTGTTGATGCAATAATGTATATGTA  67756
Domestic        ATCTCTCATGATAAATATGTTCATTATTCTGTAGGGTGTTGATGCAATAATGTATATGTA  68181
                ************************************************************
```

```
Dromedary      GATTACTTTCTGAATTACTCCTAATAAAATTAAAATTTTCAGGCTAGTTAACCTGTGCCA  68043
Wild           GATTACTTTCTGAATTACTCCTAATAAAATTAAAATTTTCAGGCTAGTTAACCTGTGCCA  67816
Domestic       GATTACTTTCTGAATTACTCCTAATAAAATTAAAATTTTCAGGCTAGTTAACCTGTGCCA  68241
               ************************************************************

Dromedary      CCCAGCTTCTTTCTGAACTGTTTTGTCTTTCCATTCTTTTGCAAGAGTTTACTTAGGTAA  68103
Wild           CCCAGCTTCTTTCTGAACTGTTTTGTCTTTCCATTCTTTTGCAAGAGTTTACTTAGGTAA  67876
Domestic       CCCAGCTTCTTTCTGAACTGTTTTGTCTTTCCATTCTTTTGCAAGAGTTTACTTAGGTAA  68301
               ************************************************************

Dromedary      TGCCAACTAATTTAATATCAGGCCAAACAGATGATAATGCTTTATATTTTATAAAAATTA  68163
Wild           TGCCAACTAATTTAATATCAGGCCAAACAGATGATAATGCTTTATATTTTATAAAAATTA  67936
Domestic       TGCCAACTAATTTAATATCAGGCCAAACAGATGATAATGCTTTATATTTTATAAAAATTA  68361
               ************************************************************

Dromedary      ATAAAAATCA-TTTTAAAACCTACTATAAATTTAGAGTTACTCTTCTGGCTTACCTGTGC  68222
Wild           ATAAAAATCATTTTTAAAACCTACTATAAATTTAGAGTTACTCTTCTGGCTTACCTGTGC  67996
Domestic       ATAAAAATCATTTTTAAAACCTACTATAAATTTAGAGTTACTCTTCTGGCTTACCTGTGC  68421
               ********** *************************************************

Dromedary      TTGTGTTTATTTCTGGTTTCAAAAATTTATTTAATGTGATATTTTTTCCTTCCATTTATT  68282
Wild           TTGTGTTTATTTCTGGTTTCAAAAATTTATTTAATGTGATATTTTTTCCTTCCATTTATT  68056
Domestic       TTGTGTTTATTTCTGGTTTCAAAAATTTATTTAATGTGATATTTTTTCCTTCCATTTATT  68481
               ************************************************************

Dromedary      GATAAAATTTACAACAAAAGATTACACTTACAAGCAATAAATGTTATCTTTTTAGTTTTT  68342
Wild           GATAAAATTTACAACAAAAGATTACACTTACAAGCAATAAATGTTATCTTTTTAGTTTTT  68116
Domestic       GATAAAATTTACAACAAAAGATTACACTTACAAGCAATAAATGTTATCTTTTTAGTTTTT  68541
               ************************************************************

Dromedary      AAATGGTCTTATTTATAAAGATCATATGGTTAGTAAGTCACATCTACTTTAAATGAAAAC  68402
Wild           AAATGGTCTTATTTATAAAGATCATATGGTTAGTAAGTCATATCTACTTTAAATGAAAAC  68176
Domestic       AAATGGTCTTATTTATAAAGATCATATGGTTAGTAAGTCATATCTACTTTAAATGAAAAC  68601
               **************************************** *******************

Dromedary      ATATTTTTTAAGAGATTACATAATTTTCCAAGTGAACTATTTTTCTTTAACTATGCTACA  68462
Wild           ATATTTTTTAAGAGATTACATAATTTTCCAAGTGAACTATTTTTCTTTAACTATGCTACA  68236
Domestic       ATATTTTTTAAGAGATTACATAATTTTCCAAGTGAACTATTTTTCTTTAACTATGCTACA  68661
               ************************************************************

Dromedary      AATGTTATTGACTCCCAAAATGATGTTACTGTTTTTTATAGTCTTAAATAACAATAATTA  68522
Wild           AATGTTATTGACTCCCAAAATGATGTTACTGTTTTTTATAGTCTTAAATAACAATAATTA  68296
Domestic       AATGTTATTGACTCCCAAAATGATGTTACTGTTTTTTATAGTCTTAAATAACAATAATTA  68721
               ************************************************************

Dromedary      CCAGGTCTATTTTGATTTTGATATAGGATAAAAAATACTATTATTTTATTTAAGAATGTGG  68582
Wild           CCAGGTCTATTTTGATTTTGATATAGGATAAAAAATACTATTATTTTATTTAAGAATGTGG  68356
Domestic       CCAGGTCTATTTTGATTTTGATATAGGATAAAAAATACTATTATTTTATTTAAGAATGTGG  68781
               ************************************************************

Dromedary      TTTTTTTATAGGTAGCATTTTAATCATTAAAGTTGGTGATGTGACAAATTTTAATTATTA  68642
Wild           TTTTTTTATAGGTAGCATTTTAATCATTAAAGTTGGTGATGTGACAAATTTTAATTATTA  68416
Domestic       TTTTTTTATAGGTAGCATTTTAATCATTAAAGTTGGTGATGTGACAAATTTTAATTATTA  68841
               ************************************************************

Dromedary      TTAAACAGATGGTTAATTTGATGTATTTCTCAGATTCTTCCATATTCAAGGAAAAAAGTC  68702
Wild           TTAAACAGATGGTTAATTTGATGTATTCCTCAGATTCTTCCATATTCAAGGAAAAAAGTC  68476
Domestic       TTAAACAGATGGTTAATTTGATGTATTCCTCAGATTCTTCCATATTCAAGGAAAAAAGTC  68901
               *************************** ********************************

Dromedary      TCAAATTCATGAAAAGATTGGGGCAGAGGAAGAATAAGCAGATTATTGTTTAAATATCTA  68762
Wild           TCAAATTCATGAAAAGATTGGGGCAGAGGAAGAATAAGCAGATTATTGTTTAAATATCTA  68536
Domestic       TCAAATTCATGAAAAGATTGGGGCAGAGGAAGAATAAGCAGATTATTGTTTAAATATCTA  68961
               ************************************************************

Dromedary      AATAGAAGACTTTTTCAGTGAAGGAATAAAGGAAATATTATCAGTATCTTCTTCTGAATC  68822
Wild           AATAGAAGACTTTTTCAGTGAAGGAATAAAGGAAATATTATCAGTATCTTCTTCTGAATC  68596
Domestic       AATAGAAGACTTTTTCAGTGAAGGAATAAAGGAAATATTATCAGTATCTTCTTCTGAATC  69021
               ************************************************************
```

```
Dromedary      TGTCCCTCTCTTTCTTGGAGTTTGCCTTTCCAACCC---AATATACCTACCACCACCTTC  68879
Wild           TGTCCCTCTCTTTCTTGGAGTTTGCCTTTCCAACCC---AATATACCTACCACCACCTTC  68653
Domestic       TGTCCCTCTCTTTCTTGGAGTTTGCCTTTCCAACCCAATAATATACCTACCACCACCTTC  69081
               ************************************   *********************

Dromedary      ATTACCCCACCTTCCTTTTTTCCATTACAGTCCACACAGTGCTGGGAGGTAACTATTTTG  68939
Wild           ATTACCCCACCTTCCTTTTTTCCATTACAGTCCACACAGTGCTGGGAGGTAACTATTTTG  68713
Domestic       ATTACCCCACCTTCCTTTTTTCCATTACAGTCCACACAGTGCTGGGAGGTAACTATTTTG  69141
               ************************************************************

Dromedary      TTTTGGTGTTAGTATCCAAGTTTCCCCAAATAAGACCTAGTGAATGGAAGATGGATGTGT  68999
Wild           TTTTGGTGTTAGTATCCAAGTTTCCCCAAATAAGACCTAGTGAATGGAAGATGGATGTGT  68773
Domestic       TTTTGGTGTTAGTATCCAAGTTTCCCCAAATAAGACCTAGTGAATGGAAGATGGATGTGT  69201
               ************************************************************

Dromedary      GTACCTGTCCCTCCAGGAGTCATCAGACATATTTAGCCACCATATTTAATCAACAAACAG  69059
Wild           GTACCTGTCCCTCCAGGAGTCATCAGACATATTTAGCCACCATATTTAATCAACAAACAG  68833
Domestic       GTACCTGTCCCTCCAGGAGTCATCAGACATATTTAGCCACCATATTTAATCAACAAACAG  69261
               ************************************************************

Dromedary      GAAGAGAGGAAGCTAACCTCTCCTCCCTCTTCTTTCCTCCCCCTCCTTCTCTTCCCTCTC  69119
Wild           GAAGAGAGGAAGCTAACCTCTCCTCCCTCTTCTTTCCTCCCCCTCCTTCTCTTCCCTCTC  68893
Domestic       GAAGAGAGGAAGCTAACCTCTCCTCCCTCTTCTTTCCTCCCCCTCCTTCTCTTCCCTCTC  69321
               ************************************************************

Dromedary      TCCCTTTCTTCCTATAAATATTTTCAGAGCATCTATTATGTGCCAGGCATTCAGATACTC  69179
Wild           TCCCTTTCTTCCTATAAATATTTTCAGAGCATCTATTATGTGCCAGGCATTCAGATACTC  68953
Domestic       TCCCTTTCTTCCTATAAATATTTTCAGAGCATCTATTATGTGCCAGGCATTCAGATACTC  69381
               ************************************************************

Dromedary      AAACTGGGGAAAACAAGAACAAAAAAAGACACAGATATGACCACAGGAGAATGTATATTG  69239
Wild           AAACTGGGGAAAACAAGAACAAAAAAAGACACAGATATGACCACAGGAGAATGTATATTG  69013
Domestic       AAACTGGGGAAAACAAGAACAAAAAAAGACACAGATATGACCACAGGAGAATGTATATTG  69441
               ************************************************************

Dromedary      CTGCTATATTGTTCTGAGCCATAAGGGAAGAATCAAGCCTAGTATAAATTAAAATTCCTT  69299
Wild           CTGCTATATTGTTCTGAGCCATAAGGGAAGAATCAAGCCTAGTATAAATTAAAATTCCTT  69073
Domestic       CTGCTATATTGTTCTGAGCCATAAGGGAAGAATCAAGCCTAGTATAAATTAAAATTCCTT  69501
               ************************************************************

Dromedary      AATGCTGTGCCTTTTAAAAACAAATGTGGTATAAGCAAAATGATTAGCTTTTGTCTTCAA  69359
Wild           AATGCTGTGCCTTTTAAAAACAAATGTGGTATAGGCAAAATGATTAGCTTTTGTCTTCAA  69133
Domestic       AATGCTGTGCCTTTTAAAAACAAATGTGGTATAAGCAAAATGATTAGCTTTTGTCTTCAA  69561
               ********************************* **************************

Dromedary      TAATGAGTCCCTGAGGTAGGAAAGTATTTCAGCATCTATTAGTATTACTAACTCTTCTTT  69419
Wild           TAATGAGTCCCTGAGGTAGGAAAGTATTTCAGGATCTATTAGTATTACTAACTCTTCTTT  69193
Domestic       TAATGAGTCCCTGAGGTAGGAAAGTATTTCAGGATCTATTAGTATTACTAACTCTTCTTT  69621
               ******************************** ***************************

Dromedary      CCTTTTCATATAGAATCCCTTTTTAGAAGTCAAGGTAACAGACACACCAAAAAGATCCAG  69479
Wild           CCTTTTCATATAGAATCCCTTTTTAGAAGTCAAGGTAACAGACACACCAAAAAGATCCAG  69253
Domestic       CCTTTTCATATAGAATCCCTTTTTAGAAGTCAAGGTAACAGACACACCAAAAAGATCCAG  69681
               ************************************************************

Dromedary      GAGAGATTTTGGACTTGACTGTGATGAGCACTCAACAGAATCTCGATGCTGTCGATACCC  69539
Wild           GAGAGATTTTGGACTTGACTGTGATGAGCACTCAACAGAATCTCGATGCTGTCGATACCC  69313
Domestic       GAGAGATTTTGGACTTGACTGTGATGAGCACTCAACAGAATCTCGATGCTGTCGATACCC  69741
               ************************************************************

Dromedary      TCTAACTGTGGATTTTGAAGCTTTTGGATGGGATTGGATTATTGCACCTAAGAGATATAA  69599
Wild           TCTAACTGTGGATTTTGAAGCTTTTGGATGGGATTGGATTATTGCACCTAAGAGATATAA  69373
Domestic       TCTAACTGTGGATTTTGAAGCTTTTGGATGGGATTGGATTATTGCACCTAAGAGATATAA  69801
               ************************************************************

Dromedary      GGCCAATTACTGCTCTGGAGAGTGTGAATTTGTATTTTTACAAAAATATCCTCATACTCA  69659
Wild           GGCCAATTACTGCTCTGGAGAGTGTGAATTTGTATTTTTACAAAAATATCCTCATACTCA  69433
Domestic       GGCCAATTACTGCTCTGGAGAGTGTGAATTTGTATTTTTACAAAAATATCCTCATACTCA  69861
               ************************************************************
```

```
Dromedary       CCTTGTGCACCAAGCAAACCCCAGAGGTTCGGCAGGTCCCTGCTGTACTCCCACAAAGAT  69719
Wild            TCTTGTGCACCAAGCAAACCCCAGAGGTTCGGCAGGTCCCTGCTGTACTCCCACAAAGAT  69493
Domestic        TCTTGTGCACCAAGCAAACCCCAGAGGTTCGGCAGGTCCCTGCTGTACTCCCACAAAGAT  69921
                 ***********************************************************

Dromedary       GTCTCCAATTAATATGCTATATTTTAATGGCAAAGAACAAATAATATATGGGAAAATTCC  69779
Wild            GTCTCCAATTAATATGCTATATTTTAATGGCAAAGAACAAATAATATATGGGAAAATTCC  69553
Domestic        GTCTCCAATTAATATGCTATATTTTAATGGCAAAGAACAAATAATATATGGGAAAATTCC  69981
                ************************************************************

Dromedary       AGCTATGGTAGTAGATCGCTGTGGGTGCTCATGAGGTTTCTATTTGGTTCATAACTTCCT  69839
Wild            AGCTATGGTAGTAGATCGCTGTGGGTGCTCATGAGGTTTGTATTTGGTTCATAACTTCCT  69613
Domestic        AGCTATGGTAGTAGATCGCTGTGGGTGCTCATGAGGTTTCTATTTGGTTCATAACTTCCT  70041
                *************************************** ********************

Dromedary       AAAATGTGGAAGGTCTTCCCCTCAACAATTTTGAAACTGTGAAATTATATACCACAGGCT  69899
Wild            AAAATGTGGAAGGTCTTCCCCTCAACAATTTTGAAACTGTGAAATTATATACCACAGGCT  69673
Domestic        AAAATGTGGAAGGTCTTCCCCTCAACAATTTTGAAACTGTGAAATTATATACCACAGGCT  70101
                ************************************************************

Dromedary       TTAAGCCTAGAGTATGCTACAGTCACTTAAGCACAAGCTACAGTATATGAACTAAAAGAG  69959
Wild            TTAAGCCTAGAGTATGCTACAGTCACTTAAGCACAAGCTACAGTATATGAACTAAAAGAG  69733
Domestic        TTAAGCCTAGAGTATGCTACAGTCACTTAAGCACAAGCTACAGTATATGAACTAAAAGAG  70161
                ************************************************************

Dromedary       AGAATATATGCAATGGTTGGCATTTAACCATCAAAACAAATCATACAATAAAAAGTTTTA  70019
Wild            AGAATATATGCAATGGTTGGCATTTAACCATCAAAACAAATCATACAATAAAAAGTTTTA  69793
Domestic        AGAATATATGCAATGGTTGGCATTTAACCATCAAAACAAATCATACAATAAAAAGTTTTA  70221
                ************************************************************

Dromedary       TGATTTCCTAAGTTTTCGAGCTAGGAGATCAAATTCCATTTATGTTCATATATATTACAA  70079
Wild            TGATTTCCTAAGTTTTCGAGCTAGGAGATCAAATTCCATTTATGTTCATATATATTACAA  69853
Domestic        TGATTTCCTAAGTTTTCGAGCTAGGAGATCAAATTCCATTTATGTTCATATATATTACAA  70281
                ************************************************************

Dromedary       CATATGCAGGTAAATGAAAGCAATTCTCCTTGTGTTCTGGTGAATTAAAGGAGTTTGCTA  70139
Wild            CATATGCAGGTAAATGAAAGCAATTCTCCTTGTGTTCTGGTGAATTAAAGGAGTTTGCTA  69913
Domestic        CATATGCAGGTAAATGAAAGCAATTCTCCTTGTGTTCTGGTGAATTAAAGGAGTTTGCTA  70341
                ************************************************************

Dromedary       TGTCTATTTCTTTACAGTTTCATTTAATATTTACAGAAAAATCTATATGTAGTATTGGTA  70199
Wild            TGTTTATTTCTTTACAGTTTCATTTAATATTTACAGAAAAATCTATATGTAGTATTGGTA  69973
Domestic        TGTCTATTTCTTTACAGTTTCATTTAATATTTACAGAAAAATCTATATGTAGTATTGGTA  70401
                *** ********************************************************

Dromedary       AAATGCAGGATTGTGATATACCATTATTTGAATCATCCTTAAACACTTGAATTTATATTG  70259
Wild            AAATGCAGGATTGTGATATACCATTATTTGAATCATCCTTAAACACTTGAATTTATATTG  70033
Domestic        AAATGCAGGATTGTGATATACCATTATTTGAATCATCCTTAAACACTTGAATTTATATTG  70461
                ************************************************************

Dromedary       TATGATAGCATATCTGGTAAGATGAGATTCCACAAAAATAGGGATGGTACACCATATGCA  70319
Wild            TATGACAGCATATCTGGTAAGATGAGATTCCACAAAAATAGGGATGGTACACCATATGCA  70093
Domestic        TATGACAGCATATCTGGTAAGATGAGATTCCACAAAAATAGGGATGGTACACCATATGCA  70521
                ***** ******************************************************

Dromedary       AGTTCCCATTCCTATTCTGATTCATACAGTACATTAACAATTCATGCCAATGGTGCTAAT  70379
Wild            AGTTCCCATTCCTATTCTGATTCATACAGTACATTAACAATTCATGCCAATGGTGCTAAT  70153
Domestic        AGTTCCCATTCCTATTCTGATTCATACAGTACATTAACAATTCATGCCAATGGTGCTAAT  70581
                ************************************************************

Dromedary       ACAATAGGCTGAATGGCTGATGTTATCAGGCTTAACAAATAAAAAACATACAGTAATGTA  70439
Wild            ACAATAGGCTGAATGGCTGATGTTATCAGGCTTAACAAATAAAAAACATACAGTAATGTA  70213
Domestic        ACAATAGGCTGAATGGCTGATGTTATCAGGCTTAACAAATAAAAAACATACAGTAATGTA  70641
                ************************************************************

Dromedary       ATAAGTTTCTCCTTTCTTTAGGTGCATTTTCATACTCCTCTAAATGGGGAATGGATTTTC  70499
Wild            ATAAGTTTCTCCTTTCTTCAGGTGCATTTTCATACTCCTCCAAATGGGGAATGGATTTTC  70273
Domestic        ATAAGTTTCTCCTTTCTTCAGGTGCATTTTCATACTCCTCCAAATGGGGAATGGATTTTC  70701
                ****************** ********************** *******************
```

```
Dromedary      TTTAATGAAATAAAAACTTTTTTTTAGAGGTCAGCATTCAATTCTGTAGCATACCTGGAG  70559
Wild           TTTAATGAAATAAAAACTTTTTTTTAGAGGTCAGCATTCAATTCTGTAGCATACCTGGAG  70333
Domestic       TTTAATGAAATAAAAACTTTTTTTTAGAGGTCAGCATTCAATTCTGTAGCATACCTGGAG  70761
               ************************************************************

Dromedary      AAACTGCATTTAAAAGGCAGCCAAAAAATATTCATCTTTATCAAAATTTCAAAATTATAG  70619
Wild           AAACTGCATTTAAAAGGCAGCCAAAAAATATTCATCTTTATCAAAATTTCAAAATTATAG  70393
Domestic       AAACTGCATTTAAAAGGCAGCCAAAAAATATTCATCTTTATCAAAATTTCAAAATTATAG  70821
               ************************************************************

Dromedary      CTTGCCTTTGCAACACTTCAGTTTTTATGATAAAATAATGGAAATGACTGATTCTATCAA  70679
Wild           CTTGCCTTTGCAACACTTCAGTTTTTATGATAAAATAATGGAAATGACTGATTCTATCAA  70453
Domestic       CTTGCCTTTGCAACACTTCAGTTTTTATGATAAAATAATGGAAATGACTGATTCTATCAA  70881
               ************************************************************

Dromedary      TATTGTATAAAAGACTTTGAAACAATTGCATTTATATAATATGTATACAATATTGTTTTG  70739
Wild           TATTGTATAAAAGACTTTGAAACAATTGCATTTATATAATATGTATACAATATTGTTTTG  70513
Domestic       TATTGTATAAAAGACTTTGAAACAATTGCATTTATATAATATGTATACAATATTGTTTTG  70941
               ************************************************************

Dromedary      TAAATAAATGTCTCCTTTTTTATTTACTTCGGTATATTTTTACAGTAAGGACATTTCAAA  70799
Wild           TAAATAAATGTCTCCTTTTTTATTTACTTCGGTATATTTTTACAGTAAGGACATTTCAAA  70573
Domestic       TAAATAAATGTCTCCTTTTTTATTTACTTCGGTATATTTTTACAGTAAGGACATTTCAAA  71001
               ************************************************************

Dromedary      TTAAGTATTAAGGCACAGAAACATGTAATGTATGATGGAAAAGCAACTGCTTATATTTCG  70859
Wild           TTAAGTATTAAGGCACAGAAACATGTAATGTATGATGGAAAAGCAACTGCTTATATTTCG  70633
Domestic       TTAAGTATTAAGGCACAGAAACATGTAATGTATGATGGAAAAGCAACTGCTTATATTTCG  71061
               ************************************************************

Dromedary      GAGCAAATTAGCAGATTAAATAGTGATCTTAAAACTCCATATGCTAATGGTTAGATGGTT  70919
Wild           GAGCAAATTAGCAGATTAAATAGTGATCTTAAAACTCCATATGCTAATGGTTAGATGGTT  70693
Domestic       GAGCAAATTAGCAGATTAAATAGTGATCTTAAAACTCCATATGCTAATGGTTAGATGGTT  71121
               ************************************************************

Dromedary      ATATTACAATCATTTTATATTTTTTTACATTATTAACATTCACTTATGGATTCATGATGG  70979
Wild           ATATTACAATCATTTTATATTTTTTTACATTATTAACATTCACTTATGGATTCATGATGG  70753
Domestic       ATATTACAATCATTTTATATTTTTTTACATTATTAACATTCACTTATGGATTCATGATGG  71181
               ************************************************************

Dromedary      CTGTATAATGTGAATGTGTGAAATTTCAATGGTTTACTGTCATTGTATTCAAATCTCAAC  71039
Wild           CTGTATAATGTGAATGTGTGAAATTTCAATGGTTTACTGTCATTGTATTCAAATCTCAAC  70813
Domestic       CTGTATAATGTGAATGTGTGAAATTTCAATGGTTTACTGTCATTGTATTCAAATCTCAAC  71241
               ************************************************************

Dromedary      GTTCCATAATTTTAATACTTATAAATATTATTAAGCGTACCAAAATGATTTAACTCTATT  71099
Wild           GTTCCATAATTTTAATACTTATAAATATTATTAAGCGTACCAAAATGATTTAACTCTATT  70873
Domestic       GTTCCATAATTTTAATACTTATAAATATTATTAAGCATACCAAAATGATTTAACTCTATT  71301
               ************************************ ***********************

Dromedary      ATCTGAAATCAGAATAATAAACTGATGCTATGTTAAGAATTGTTAATTTTATTTTATAAT  71159
Wild           ATCTGAAATCAGAATAATAAACTGATGCTATGTTAAGAATTGTTAATTTTATTTTATAAT  70933
Domestic       ATCTGAAATCAGAATAATAAACTGATGCTATGTTAAGAATTGTTAATTTTATTTTATAAT  71361
               ************************************************************

Dromedary      TCGATAATGAATATATTTCTGCATATATTTACTTCTATTTTGTAAATTAGGATTTTTTAA  71219
Wild           TCGATAATGAATATATTTCTGCATATATTTACTTCTATTTTGTAAATTAGGATTTTTTAA  70993
Domestic       TCGATAATGAATATATTTCTGCATATATTTACTTCTATTTTGTAAATTAGGATTTTTTAA  71421
               ************************************************************

Dromedary      TCAAATACATTGTACTTATGACTAAGTGAAATGATTTCTTACATCTAATGTGTAGAAACA  71279
Wild           TCAAATACATTGTACTTATGACTAAGTGAAATGATTTCTTACATCTAATGTGTAGAAACA  71053
Domestic       TCAAATACATTGTACTTATGACTAAGTGAAATGATTTCTTACATCTAATGTGTAGAAACA  71481
               ************************************************************

Dromedary      ACATAAATTATATTAAAGAGTTTTCACCTTTTTTGAAAGACACAACAGTTTTATGCTATA  71339
Wild           ACATAAATTATATTAAAGAGTTTTCACCTTTTTTGAAAGACACAACAGTTTTATGCTATA  71113
Domestic       ACATAAATTATATTAAAGAGTTTTCACCTTTTTTGAAAGACACAACAGTTTTATGCTATA  71541
               ************************************************************
```

```
Dromedary     ATGATTAATTCTAGATTTCTGGTTTTCACTTTATTATAAAAGTTAAAAAACTTAGCACAA  71399
Wild          ATGATTAATTCTAGATTTCTGGTTTTCACTTTATTATAAAAGTTAAAAAACTTAGCACAA  71173
Domestic      ATGATTAATTCTAGATTTCTGGTTTTCACTTTATTATAAAAGTTAAAAAACTTAGCACAA  71601
              ************************************************************

Dromedary     AAGTTTGGTTTAGAAATTTTAGATCTGCTACTCTAGTTCTCATGAGTGAAATTCCTGTTA  71459
Wild          AAGTTTGGTTTAGAAATTTTAGATCTGCTACTCTAGTTCTCATGAGTGAAATTCCTGTTA  71233
Domestic      AAGTTTGGTTTAGAAATTTTAGATCTGCTACTCTAGTTCTCATGAGTGAAATTCCTGTTA  71661
              ************************************************************

Dromedary     AATTGGTTCTGGTTAAGTTGCTTTAAACATATGAAAGCCAGGACTAGTTATATCTGTTTC  71519
Wild          AATTGGTTCTGGTTAAGTTGCTTTAAACATATGAAAGCCAGGACTAGTTATATCTGTTTC  71293
Domestic      AATTGGTTCTGGTTAAGTTGCTTTAAACATATGAAAGCCAGGACTAGTTATATCTGTTTC  71721
              ************************************************************

Dromedary     ATTTCTCTTTATCTTGACCTGAAAACTATTTATATGTTTTCATAGGTTCAATTTCCAAAT  71579
Wild          ATTTCTCTTTATCTTGACCTGAAAACTATTTATATGTTTTCATAGGTTCAATTTCCAAAT  71353
Domestic      ATTTCTCTTTATCTTGACCTGAAAACTATTTATATGTTTTCATAGGTTCAATTTCCAAAT  71781
              ************************************************************

Dromedary     GCATTGCAGTTGGCAAGGGTATATGGTCCTAGAGTTACAAGCTGCCTTACTGAAGCCACA  71639
Wild          GCATTGCAGTTGGCAAGGGTATATGGTCCTAGAGTTACAAGCTGCCTTACTGAAGCCACA  71413
Domestic      GCATTGCAGTTGGCAAGGGTATATGGTCCTAGAGTTACAAGCTGCCTTACTGAAGCCACA  71841
              ************************************************************

Dromedary     GGGACACAGGGAAACTGCATCTTTTTCCTAGCACTTAATGATACTGACACCCTTATCTGA  71699
Wild          GGGACACAGGGAAACTGCATCTTTTTCCTAGCACTTAATGATACTGACACCCTTATCTGA  71473
Domestic      GGGACACAGGGAAACTGCATCTTTTTCCTAGCACTTAATGATACTGACACCCTTATCTGA  71901
              ************************************************************

Dromedary     GCCTTAGGGGCACCAATTTTCAAAGTAAATTGAAAAATAATTATAAAGTGCCTAGAAATC  71759
Wild          GCCTTAGGGGCACCAATTTTCAAAGTAAATTGAAAAATAATTATAAAGTGCCTAGAAATC  71533
Domestic      GCCTTAGGGGCACCAATTTTCAAAGTAAATTGAAAAATAATTATAAAGTGCCTAGAAATC  71961
              ************************************************************

Dromedary     CTTGAGTGCAACATTGTACATAAATATTCTCGAGGTGAGCTCCCTTCTCCACCTCTCATT  71819
Wild          CTTGAGTGCAACATTGTACATAAATATTCTCGAGGTGAGCTCCCTTCTCCACCTCTCATT  71593
Domestic      CTTGAGTGCAACATTGTACATAAATATTCTCGAGGTGAGCTCCCTTCTCCACCTCTCATT  72021
              ************************************************************

Dromedary     AGTTTTGTAACTTCGCTCTAAAGCAGTGACTAAGTGTAATGGGGGAGGTGATTAAAATTC  71879
Wild          AGTTTTGTAACTTCGCTCTAAAGCAGTGACTAAGTGTAATGGGGGAGGTGATTAAAATTC  71653
Domestic      AGTTTTGTAACTTCGCTCTAAAGCAGTGACTAAGTGTAATGGGGGAGGTGATTAAAATTC  72081
              ************************************************************

Dromedary     TACCTGGTTCTTAAATAAAAAATTTATAAGTAATATAAATGTAATTTTTAACCATGAATG  71939
Wild          TACCTGGTTCTTAAATAAAAAATTTATAAGTAATATAAATGTAATTTTTAACCATGAATG  71713
Domestic      TACCTGGTTCTTAAATAAAAAATTTATAAGTAATATAAATGTAATTTTTAACCATGAATG  72141
              ************************************************************

Dromedary     ATATTACAGCAATTCATACTCATACAAAGTGCAGTACTATTTCTAATTTGAATACTGACA  71999
Wild          ATATTACAGCAATTCATACTCATACAAAGTGCAGTACTATTTCTAATTTGAATACTGACA  71773
Domestic      ATATTACAGCAATTCATACTCATACAAAGTGCAGTACTATTTCTAATTTGAATACTGACA  72201
              ************************************************************

Dromedary     TATTTAATGCTGACAAGACTGGGGCTGTAAGGGAGTTGCCCCTGTGGGGGAAGGAGCCAG  72059
Wild          TATTTAATGCTGACAAGACTGGGGCTGTAAGGGAGTTGCCCCTGTGGGGGAAGGAGCCAG  71833
Domestic      TATTTAATGCTGACAAGACTGGGGCTGTAAGGGAGTTGCCCCTGTGGGGGAAGGAGCCAG  72261
              ************************************************************

Dromedary     CTGTCATGATAGGGTGAGGCCACTATTCAGTTGCCATCATCTGAACCTACTTCTTTCAAG  72119
Wild          CTGTCATGATAGGGTGAGGCCACTATTCAGTTGCCATCATCTGAACCTACTTCTTTCAAG  71893
Domestic      CTGTCATGATAGGGTGAGGCCACTATTCAGTTGCCATCATCTGAACCTACTTCTTTCAAG  72321
              ************************************************************

Dromedary     ACCATGAACATACAGGACCTAAAAGTCTGGGGCAGCAGGCCCATAAACACACCTTAGGCT  72179
Wild          ACCATGAACATACAGGACCTAAAAGTCTGGGGCAGCAGGCCCATAAACACACCTTAGGCT  71953
Domestic      ACCATGAACATACAGGACCTAAAAGTCTGGGGCAGCAGGCCCATAAACACACCTTAGGCT  72381
              ************************************************************
```

```
Dromedary      TTGGAGTACAAATAACCGGGGACCTTGATCTCTATAGTGTGGACTGTGAGGAAAGAACAC  72239
Wild           TTGGAGTACAAATAACCGGGGACCTTGATCTCTATAGTGTGGACTGTGAGGAAAGAACAC  72013
Domestic       TTGGAGTACAAATAACCGGGGACCTTGATCTCTATAGTGTGGACTGTGAGGAAAGAACAC  72441
               ************************************************************

Dromedary      CCTCAGTTTCCAAGGACCCAGTCACAGGACTCCCAATCTTTATACTCACCCTTACCATGA  72299
Wild           CCTCAGTTTCCAAGGACCCAGTAACAGGACTCCCAATCTTTATACTCACCCTTACCATGA  72073
Domestic       CCTCAGTTTCCAAGGACCCAGTCACAGGACTCCCAATCTTTATACTCACCCTTACCATGA  72501
               ********************** *************************************

Dromedary      AGCCCCAGGGTCACATTACCAACCTCTCCATTTGGTAGCCCTCTTTGAAACCCATATGAC  72359
Wild           AGCCCCAGGGTCACATTACCAACCTCTCCATTTGGTAGCCCTCTTTGAAACCCATATGAC  72133
Domestic       AGCCCCAGGGTCACATTACCAACCTCTCCATTTGGTAGCCCTCTTTGAAACCCATATGAC  72561
               ************************************************************

Dromedary      CATGAAAGGAAGCATTTATTTTGGGACAAGACCTAGGCATTTCTTTTTCAATTTTGTCTA  72419
Wild           CATGAAAGGAAGCATTTATTTTGGGACAAGACCTAGGCATTTCTTTTTCAATTTTGTCTA  72193
Domestic       CATGAAAGGAAGCATTTATTTTGGGACAAGACCTAGGCATTTCTTTTTCAATTTTGTCTA  72621
               ************************************************************

Dromedary      GGTCCTACAAATGGCAAAGCCACTGCCAGTCTGCCTATGGGCACATCACTTTCCTTTTCA  72479
Wild           GGTCCTACAAATGGCAAAGCCACTGCCAGTCTGCCTATGGGCACATCACTTTCCTTTTCA  72253
Domestic       GGTCCTACAAATGGCAAAGCCACTGCCAGTCTGCCTATGGGCACATCACTTTCCTTTTCA  72681
               ************************************************************

Dromedary      GCACCCCAAGAATATTCATTTGTAATATGTAATACTACTTTCTGAGATTTTCAGAAAATG  72539
Wild           GCACCCCAAGAATATTCATTTGTAATATGTAATACTACTTTCTGAGATTTTCAGAAAATG  72313
Domestic       GCACCCCAAGAATATTCATTTGTAATATGTAATACTACTTTCTGAGATTTTCAGAAAATG  72741
               ************************************************************

Dromedary      CAATACGAAGTTTAATGCAGTGTATCAGTTTTACTATTCTGAATGTTTTGTACGCCTTCC  72599
Wild           CAATACGAAGTTTAATGCAGTGTATCAGTTTTACTATTCTGAATGTTTTGTACGCCTTCC  72373
Domestic       CAATACGAAGTTTAATGCAGTGTATCAGTTTTACTATTCTGAATGTTTTGTACGCCTTCC  72801
               ************************************************************

Dromedary      CATACTTACCGCTAACCTTAAAAAACAACGATAAAAATAAAAACAAAAAAATCCTTTTAA  72659
Wild           CATACTTACCGCTAACCTTAAAAAACAACGATAAAAATAAAAACAAAAAAATCCTTTTAA  72433
Domestic       CATACTTACCGCTAACCTTAAAAAACAACGATAAAAATAAAAACAAAAAAATCCTTTTAA  72861
               ************************************************************

Dromedary      ACTCTATGGCTCCTTTTGTATTCAAGTTGGTTTTTGCTCTTCTGAATAGAACCCTATTTT  72719
Wild           ACTCTATGGCTCCTTTTGTATTCAAGTTGGTTTTTGCTCTTCTGAATAGAACCCTATTTT  72493
Domestic       ACTCTATGGCTCCTTTTGTATTCAAGTTGGTTTTTGCTCTTCTGAATAGAACCCTATTTT  72921
               ************************************************************

Dromedary      ATTCAAATAAAAAGGCAGCATTTTGACACTGAGAGTAACTAAATTAGAACTCAATTATTC  72779
Wild           ATTCAAATAAAAAGGCAGCATTTTGACACTGAGAGTAACTAAATTAGAACTCAATTATTC  72553
Domestic       ATTCAAATAAAAAGGCAACATTTTGACACTGAGAGTAACTAAATTAGAACTCAATTATTC  72981
               ***************** ******************************************

Dromedary      TCTCGAAAACAAAAACAAATCTATTTCATTTTAAACCAGTCATTTTAGGATATCAGTGGC  72839
Wild           TCTCGAAAACAAAAACAAATCTATTTCATTTTAAACCAGTCATTTTAGGATATCAGTGGC  72613
Domestic       TCTCGAAAACAAAAACAAATCTATTTCATTTTAAACCAGTCATTTTAGGATATCAGTGGC  73041
               ************************************************************

Dromedary      CCTTTTGGATCTGTTGAAAACTAGTGACACTCTCAACAGGAAAAATAAAAATCACAATTT  72899
Wild           CCTTTTGGATCTGTTGAAAACTAGTGACACTCTCACCAGGAAAAATAAAAATCACAATTT  72673
Domestic       CCTTTTGGATCTGTTGAAAACTAGTGACACTCTCACCAGGAAAAATAAAAATCACAATTT  73101
               *********************************** ************************

Dromedary      TGCATATAATTTCAGGGAGTTTAGGGCCTTCTGAGAGCTCATTCATAGACTCTGGTCTAT  72959
Wild           TGCATATAATTTCAGGGAGTTTAGGGCCTTCTGAGAGCTCATTCATAGACTCTGGTCTAT  72733
Domestic       TGCATATAATTTCAGGGAGTTTAGGGCCTTCTGAGAGCTCATTCATAGACTCTGGTCTAT  73161
               ************************************************************

Dromedary      AAACCCTTAAGAAGGATACCAAATTTTCCTTGTTAAGATAAAAATTATTTATGAAGTAAT  73019
Wild           AAACCCTTAAGAAGGATACCAAATTTTCCTTGTTAAGATAAAAATTATTTATGAAGTAAT  72793
Domestic       AAACCCTTAAGAAGGATACCAAATTTTCCTTGTTAAGATAAAAATTATTTATGAAGTAAT  73221
               ************************************************************
```

```
Dromedary      AAAAATATATATATATAAGGGTCTGCCTCCCAGTTAATCAGTTTTAATCATTAAACGATT  73079
Wild           AAAAATATATATATATAAGGGTCTGCCTCCCAGTTAATCAGTTTTAATCATTAAACGATT  72853
Domestic       AAAAAAATATATATATAAGGGTCTGCCTCCCAGTTAATCAGTTTTAATCATTAAACGATT  73281
               ***** ******************************************************

Dromedary      TGAATGTTCAAAAAGGAAAATAAACATCAAATCAGCCTGCATGTCTTAACATGGCTAGAA  73139
Wild           TGAATGTTCAAAAAGGAAAATAAACATCAAATCAGCCTGCATGTCTTAACATGGCTAGAA  72913
Domestic       TGAATGTTCAAAAAGGAAAATAAACATCAAATCAGCCTGCATGTCTTAACATGGCTAGAA  73341
               ************************************************************

Dromedary      CTTTTACTAATTACCAAATATTCTGTGTTTCTTAAAGACTATCCAATCACCAACTGGCAC  73199
Wild           CTTTTACTAATTACCAAATATTCTGTGTTTCTTAAAGACTATCCAATCACCAACTGGCAC  72973
Domestic       CTTTTACTAATTACCAAATATTCTGTGTTTCTTAAAGACTATCCAATCACCAACTGGCAC  73401
               ************************************************************

Dromedary      CTCTAATAGATTAAACTAGACAACTAAATACAAAGCACTTCAAATTATGAGTTCACATTC  73259
Wild           CTCTAATAGATTAAACTAGACAACTAAATACAAAGCACTTCAAATTATGAGTTCACATTC  73033
Domestic       CTCTAATAGATTAAACTAGACAACTAAATACAAAGCACTTCAAATTATGAGTTCACATTC  73461
               ************************************************************

Dromedary      TTTTTATGTATCAAAAACAAAATCAAGAAATCATTAGGGCTTCTGATGTCTGGGTGTCAG  73319
Wild           TTTTTATGCATCAAAAACAAAATCAAGAAATCATTAGGGCTTCTGATGTCTGGGTGTCAG  73093
Domestic       TTTTTATGCATCAAAAACAAAATCAAGAAATCATTAGGGCTTCTGATGTCTGGGTGTCAG  73521
               ******** ***************************************************

Dromedary      AATTAGGAGGGCATGAGATGATAGTATTTACTGAGGACCAGATATGGACTAGGAACTTTC  73379
Wild           AATTAGGAGGGCATGAGATGATAGTATTTACTGAGGACCAGATATGGACTAGGAACTTTC  73153
Domestic       AATTAGGAGGGCATGAGATGATAGTATTTACTGAGGACCAGATATGGACTAGGAACTTTC  73581
               ************************************************************

Dromedary      ATGATGTGCTCAGTGTCGGGAGACATTATTGTCCCCAATTTACAGAAGAGAACTTTGAGG  73439
Wild           ATGATGTGCTCAGTGTCGGGAGACATTATTGTCCCCAATTTACAGAAGAGAACTTTGAGG  73213
Domestic       ATGATGTGCTCAGTGTCGGGAGACATTATTGTCCCCAATTTACAGAAGAGAACTTTGAGG  73641
               ************************************************************

Dromedary      ACTGAGACAATAGGAGGTACCTCAGTGGCTGGCCTGGATTTGAGTTAAGAGCAGTTTGAC  73499
Wild           ACTGAGACAATAGGAGGTACCTCAGTGGCTGGCCTGGATTTGAGTTAAGAGCAGTTTGAC  73273
Domestic       ACTGAGACAATAGGAGGTACCTCAGTGGCTGGCCTGGATTTGAGTTAAGAGCAGTTTGAC  73701
               ************************************************************

Dromedary      ACAATGATCAATTGCCCTGGTTACAACAATTCTTCACCAAGTCCTAATGAAAGAGTGAAA  73559
Wild           ACAATGATCAATTGCCCTGGTTACAACAATTCTTCACCAAGTCCTAATGAAAGAGTGAAA  73333
Domestic       ACAATGATCAATTGCCCTGGTTACAACAATTCTTCACCAAGTCCTAATGAAAGAGTGAAA  73761
               ************************************************************

Dromedary      CTATATATTTTAAAATTCATATTTTGTAACATATCTTTATCTAGATTCAGCACTGGTTTC  73619
Wild           CTATATATTTTAAAATTCATATTTTGTAACATATCTTTATCTAGATTCAGCACTGGTTTC  73393
Domestic       CTATATATTTTAAAATTCATATTTTGTAACATATCTTTATCTAGATTCAGCACTGGTTTC  73821
               ************************************************************

Dromedary      TGCAAAAGGTCTTGTGGCTGCATTTTAATGAGGGAGGTTAGAGTTGGTATCAGGTAGAAG  73679
Wild           TGCAAAAGGTCTTGTGGCTGCATTTTAATGAGGGAGGTTAGAGTTGGTATCAGGTAGAAG  73453
Domestic       TGCAAAAGGTCTTGTGGCTGCATTTTAATGAGGGAGGTTAGAGTTGGTATCAGGTAGAAG  73881
               ************************************************************

Dromedary      AAGAGCAGGTGATTAGCAAAGCAGGTAAATGTACACACAGAGATATAAGTGGGTCAGTGT  73739
Wild           AAGAGCAGGTGATTAGCAAAGCAGGTAAATGTACACACAGAGATATAAGTGGGTCAGTGT  73513
Domestic       AAGAGCAGGTGATTAGCAAAGCAGGTAAATGTACACACAGAGATATAAGTGGGTCAGTGT  73941
               ************************************************************

Dromedary      CATAGGATGCGAGAGCTTGAAAAGACCCTAGAGATAACTAATTTACATATGACAGAATTG  73799
Wild           CATAGGATGCGAGAGCTTGAAAAGACCCTAGAGATAACTAATTTACATATGACAGAATTG  73573
Domestic       CATAGGATGCGAGAGCTTGAAAAGACCCTAGAGATAACTAATTTACATATGACAGAATAG  74001
               ********************************************************** *

Dromedary      AGGTCTAAAGAGAGGGTTGCCCAAAGTTATAGAGTGAATTGGTTCAAGGACTGAGACAAG  73859
Wild           AGGTCTAAAGAGAGGGTTGCCCAAAGTTATAGAGTGAATTGGTTCAAGGACTGAGACAAG  73633
Domestic       AGGTCTAAAGAGAGGGTTGCCCAAAGTTATAGAGTGAATTGGTTCAAGGACTGAGACAAG  74061
               ************************************************************
```

```
Dromedary      AGCTCTGAAATGGTAGAATAGAATAGATGCTGGGTCAGAGTGAGAAAGAACAGTTCCCAA  73919
Wild           AGCTCTGAAATGGTAGAATAGAATAGATCCTGGGTCAGAGTGAGAAAGAACAGTTCCCAA  73693
Domestic       AGCTCTGAAATGGTAGAATAGAATAGATCCTGGGTCAGAGTGAGAAAGAACAGTTCCCAA  74121
               **************************** *******************************

Dromedary      AGACAAAATACAGGAGAGCTAAAAACAAAACAAAACAAAAGTTCTGACTCATGGTTTGTT  73979
Wild           AGACAAAATACAGGAGAGCTAAAAACAAAACAAAACAAAAGTTCTGACTCATGGTTTGTT  73753
Domestic       AGACAAAATACAGGAGAGCTAAAAACAAAACAAAACAAAAGTTCTGACTCATGGTTTGTT  74181
               ************************************************************

Dromedary      AGCTGGGGAATGTCAATATCAGACACTAGATAAATGTCCATAGGATTATAACAATTTACT  74039
Wild           AGCTGGGGAATGTCAATATCAGACACTAGATAAATGTCCATAGGATTATAACAATTTACT  73813
Domestic       AGCTGGGGAATGTCAATATCAGACACTAGATAAATGTCCATAGGATTATAACAATTTACT  74241
               ************************************************************

Dromedary      TCTACTTAAACAAATCTTTTAACAACACGGAGATTTTCTTGGACAAATAGACACTAACGG  74099
Wild           TCTACTTAAACAAATCTTTTAACAACACGGAGATTTTCTTGGACAAATAGACACTAATGG  73873
Domestic       TCTACTTAAACAAATCTTTTAACAACACGGAGATTTTCTTGGACAAATAGACACTAATGG  74301
               ********************************************************* **

Dromedary      GAAATGTATGCTAGCATGTAATTAGATCCACTACTTTATTGCATTATAGTGATACAAATT  74159
Wild           GAAATGTATGCTAGCATGTAATTAGATCCACTACTTTATTGCATTATAGTGATACAAATT  73933
Domestic       GAAATGTATGCTAGCATGTAATTAGATCCACTACTTTATTGCATTATAGTGATACAAATT  74361
               ************************************************************

Dromedary      GAAATAATAATTAATTACTAAATAATAGTTTTCAAAGTACGTTCACATTAATGATACAAT  74219
Wild           GAAATAATAATTAATTACTAAATAATAGTTTTCAAAGTACGTTCACATTAATGATACAAT  73993
Domestic       GAAATAATAATTAATTACTAAATAATAGTTTTCAAAGTACGTTCACATTAATGATACAAT  74421
               ************************************************************

Dromedary      TTTATCTTCATAATGCTGAAAATTAAGTATTATTATTATGACCATTTCATAGAGGGAATT  74279
Wild           TTTATCTTCATAATGCTGAAAATTAAGTATTATTATTATGACCATTTCATAGAGGGAATT  74053
Domestic       TTTATCTTCATAATGCTGAAAATTAAGTATTATTATTATGACCATTTCATAGAGGGAATT  74481
               ************************************************************

Dromedary      GAGGCTAAAATAATCAAACTGCCTAAGGAAATGTAGCATTTATATTTGTATATTAATTAT  74339
Wild           GAGGCTAAAATAATCAAACTGCCTAAGGAAATGTAGCATTTATATTTGTATATTAATTAT  74113
Domestic       GAGGCTAAAATAATCAAACTGCCTAAGGAAATGTAGCATTTATATTTGTATATTAATTAT  74541
               ************************************************************

Dromedary      ATAAGAAATAGAAATTTAAATCAAATAAGTTATACTTGTTTCATCATTTATTCCAATAGT  74399
Wild           ATAAGAAATAGAAATTTAAATCAAATAAGTTATACTTGTTTCATCATTTATTCCAATAGT  74173
Domestic       ATAAGAAATAGAAATTTAAATCAAATAAGTTATACTTGTTTCATCATTTATTCCAATAGT  74601
               ************************************************************

Dromedary      ACTTATTAGAAATGTAGCATGACAAAAATCCATTAAAAATATCAAATGATCACTTTAAAA  74459
Wild           ACTTATTAGAAATGTAGCATGACAAAAATCCATTAAAAATATCAAATGATCACTTTAAAA  74233
Domestic       ACTTATTAGAAATGTAGCATGACAAAAATCCATTAAAAATATCAAATGATCACTTTAAAA  74661
               ************************************************************

Dromedary      AATAATAAGACTCATGAATACTACCCGGTAATTTAACTAAGAAATTAAACCACAGACTAA  74519
Wild           AACAATAAGACTCATGAATACTACCAGGTAATTTAACTAAGAAATTAAACCACAGACTAA  74293
Domestic       AATAATAAGACTCATGAATACTACCCGGTAATTTAACTAAGAAATTAAACCACAGACTAA  74721
               ** ********************* ***********************************

Dromedary      AATGTGATATGCAAGTCATGACTTGCCTGGCATGTGACTTGTAGTGTTGAAATTTCATTT  74579
Wild           AATGTGATATGCAAGTCATGACTTGCCTGGCATGTGACTTGTAGTGTTGAAATTTCATTT  74353
Domestic       AATGTGATATGCAAGTCATGACTTGCCTGGCATGTGACTTGTAGTGTTGAAATTTCATTT  74781
               ************************************************************

Dromedary      GCATGGTCATTCAGTTCCAGTTTACACATTGCAAAACATATCAAAGTAAACTATACTAAT  74639
Wild           GCATGGTCATTCAGTTCCAGTTTACACATTGCAAAACGTATCAAAGTAAACTATACTAAT  74413
Domestic       GCATGGTCATTCAGTTCCAGTTTACACATTGCAAAACGTATCAAAGTAAACTATACTAAT  74841
               ************************************* **********************

Dromedary      TCTGGTCATCCCTGAGTACGATGTCAAGCAATGCAAAATGGTACTAAATTTAGTTCATTC  74699
Wild           TCTGGTCATCCCTGAGTACGATGTCAAGCAATGCAAAATGGTACTAAATTTAGTTCATTC  74473
Domestic       TCTGGTCATCCCTGAGTACGATGTCAAGCAATGCAAAATGGTACTAAATTTAGTTCATTC  74901
               ************************************************************
```

```
Dromedary      ATGTCTAGATGGAATTTTGCAGAGGAAGTCTTTGGAAAATTAACACATTTTCATTAGATA  74759
Wild           ATGTCTAGATGGAATTTTGCAGAGGAAGTCTTTGGAAAATTAACACATTTTCATTAGATA  74533
Domestic       ATGTCTAGATGGAATTTTGCAGAGGAAGTCTTTGGAAAATTAACACATTTTCATTAGATA  74961
               ************************************************************

Dromedary      GCATTCATTTATAGCAGGATTATCTTTGTTACCATTTAAAACACTAGACAGAGGGAATAT  74819
Wild           GCATTCATTTATAGCAGGATTATCTTTGTTACCATTTAAAACACTAGACAGAGGGAATAT  74593
Domestic       GCATTCATTTATAGCAGGATTATCTTTGTTACCATTTAAAACACTAGACAGAGGGAATAT  75021
               ************************************************************

Dromedary      TTACATATATTTTACTAAATTACCTATCAAAAGACCTTAATGAATATCACATCCTGTCAA  74879
Wild           TTACATATATTTTACTAAATTACCTATCAAAAGACCTTAATGAATATCACATCCTGTCAA  74653
Domestic       TTACATATATTTTACTAAATTACCTATCAAAAGACCTTAATGAATATCACATCCTGTCAA  75081
               ************************************************************

Dromedary      GAGCACAAGATGTTCTTGAATGTAAGATTTTACATATGAAGCTGCCTAATTTATCTGACT  74939
Wild           GAGCACAAGATGTTCTTGAATGTAAGATTTTACATATGAAGCTGCCTAATTTATCTGACT  74713
Domestic       GAGCACAAGATGTTCTTGAATGTAAGATTTTACATATGAAGCTGCCTAATTTATCTGACT  75141
               ************************************************************

Dromedary      GGCATAAAATGTATGTATAATAATGATAGACAACTCCCTAATAAACTGTTATAATGGTTG  74999
Wild           GGCATAAAATGTATGTATAATAATGATAGACAACTCCCTAATAATCTGTTATAATGGTTG  74773
Domestic       GGCATAAAATGTATGTATAATAATGATAGACAACTCCCTAATAATCTGTTATAATGGTTG  75201
               ******************************************** ***************

Dromedary      AATAGTTCACTTCAGCTATGAGTTCACAAAACACTAAATATTCCTAATACAGAAATGCAT  75059
Wild           AATAGTTCACTTCAGCTATGAGTTCACAAAACACTAAATATTCCTAATACAGAAATGCAT  74833
Domestic       AATAGTTCACTTCAGCTATGAGTTCACAAAACACTAAATATTCCTAATACAGAAATGCAT  75261
               ************************************************************

Dromedary      AACTTGGCAATGAAGCAAAGACATATATGCTTAGTTTTCATTAATTCCAGTTTTGGATTA  75119
Wild           AACTTGGCAATGAAGCAAAGACATATATGCTTAGTTTTCATTAATTCCAGTTTTGGATTA  74893
Domestic       AACTTGGCAATGAAGCAAAGACATATATGCTTAGTTTTCATTAATTCCAGTTTTGGATTA  75321
               ************************************************************

Dromedary      CTGTAAGATAG-AAAAAAATTTTAACAGTATAATATGCTATTATTTCTGCTCCATAATAA  75178
Wild           CTGTAAGATAGAAAAAAAATTTTAACAGTATAATATGCTATTATTTCTGCTCCATAATAA  74953
Domestic       CTGTAAGATAGAAAAAAAATTTTAACAGTATAATATGCTATTATTTCTGCTCCATAATAA  75381
               *********** ************************************************

Dromedary      AGGTGATTATTTATAAAACACACATATATATTTACTTTCTTAAAAAATTGACTTTTATAA  75238
Wild           AGGTGATTATTTATAAAACACACATATATATTTACTTTCTTAAAAAATTGACTTTTATAA  75013
Domestic       AGGTGATTATTTATAAAACACACATATATATTTACTTTCTTAAAAAATTGACTTTTATAA  75441
               ************************************************************

Dromedary      AATTTTCTTCTGTTAGTTCTACATACGCACTATAGGCAAAACACTATTCAATGGAATTAA  75298
Wild           AATTTTCTTCTGTTAGTTCTACATATGCACTATAGGCAAAACACTATTCAATGGAATTAA  75073
Domestic       AATTTTCTTCTGTTAGTTCTACATATGCACTATAGGCAAAACACTATTCAATGGAATTAA  75501
               ************************* **********************************

Dromedary      CTTATAGAGACCTAACATATATTCCCAGATTCTGATCTTAAGAAGATCTATTGGTAAGTC  75358
Wild           CTTATAGAGACCTAACATATATTCCCAGATTCTGATCTTAAGGAGATCTGTTGGTAAGTC  75133
Domestic       CTTATAGAGACCTAACATATATTCCCAGATTCTGATCTTAAGGAGATCTGTTGGTAAGTC  75561
               ***************************************** ****** ***********

Dromedary      CTCCTGAGGAAGGCCAACAGATAAATTAGATTCAATTAGTCTTAAAGGTAGTTTCAAACC  75418
Wild           CTCCTGAGGAAGGCCAACAAATAAATTAGATTCAATTAGTCTTAAAGGTAGTTTCAAACC  75193
Domestic       CTCCTGAGGAAGGCCAACAAATAAATTAGATTCAATTAGTCTTAAAGGTAGTTTCAAACC  75621
               ****************** *****************************************

Dromedary      CAAAATGTTAAGCAATTAACCTACTAATTCATAACTTTAATCTGTTTTCCTAACCATCCA  75478
Wild           CAAAATGTTAAGCAATTAACCTACTAATTCATAACTTTAATCTGTTTTCCTAACCATCCA  75253
Domestic       CAAAATGTTAAGCAATTAACCTACTAATTCATAACTTTAATCTGTTTTCCTAACCATCCA  75681
               ************************************************************

Dromedary      AGATAACTCTATTAAGAAAACTTTTTTCCTATAAATGTTACGTTC-TTTTTTTTTTTCTA  75537
Wild           AGATAACTCTATTAAGAAAACTTTTTTCCTATAAATGTTACGTTCTTTTTTTTTTTTCTA  75313
Domestic       AGATAACTCTATTAAGAAAACTTTTTTCCTATAAATGTTACGTTCTTTTTTTTTTTTCTA  75741
               ********************************************* **************
```

```
Dromedary      ACATCTTATGAAGGACCCTATTCAGAACAAAATTTGATTATGCGGTTTTGGCCATGGATC  75597
Wild           ACATCTTATGAAGGACCCTATTCAGAACAAAATTTGATTATGCGGTTTTGGCCATGGATC  75373
Domestic       ACATCTTATGAAGGACCCTATTCAGAACAAAATTTGATTATGCGGTTTTGGCCATGGATC  75801
               ************************************************************

Dromedary      CTCCACAAGACTTTCCCAGATCCGCTCCCACGGACACTAACTCTGACTAAGAAGCCAAAC  75657
Wild           CTCCACAAGACTTTCCCAGATCCGCTCCCACGGACACTAACTCTGACTAAGAAGCCAAAC  75433
Domestic       CTCCACAAGACTTTCCCAGATCCGCTCCCACGGACACTAACTCTGACTAAGAAGCCAAAC  75861
               ************************************************************

Dromedary      GTCTCTTGGCTCTACTCCCATGGAACTCAGAATTTTGGCACTACCTCTCCACTCACCAAA  75717
Wild           GTCTCTTGGCTCTACTCCCATGGAACTCAGAATTTTGGCACTACCTCTCCACTCACCAAA  75493
Domestic       GTCTCTTGGCTCTACTCCCATGGAACTCAGAATTTTGGCACTACCTCTCCACTCACCAAA  75921
               ************************************************************

Dromedary      AACAAAACATGTCAATACAGGAACGTTTGCCTACATCATGACATCAGATCCTCATAATGC  75777
Wild           AACAAAACATGTCAATACAGGAACGTTTGCCTACATCATGACATCAGATCCTCATAATGC  75553
Domestic       AACAAAACATGTCAATACAGGAATGTTTGCCTACATCATGACATCAGATCCTCATAATGC  75981
               *********************** ************************************

Dromedary      TAAGATAGAATTCTGAAAGTGAAAATTAAGCATAAAACACCAATTTACTGAGCACACTGA  75837
Wild           TAAGATAGAATTCTGAAAGTGAAAATTAAGCATAAAACACCAATTTACTGAGCACACTGA  75613
Domestic       TAAGATAGAATTCTGAAAGTGAAAATTAAGCATAAAACACCAATTTACTGAGCACACTGA  76041
               ************************************************************

Dromedary      AGTGACTATGGGAGAAAACTCAAATTGTCAAATGGAAACAATCAAGCAGTCATTGAGGCA  75897
Wild           AGTGACTGTGGGAGAAAACTCAAATTGTCAAATGGAAACAATCAAGCAGTCATTGAGGCA  75673
Domestic       AGTGACTGTGGGAGAAAACTCAAATTGTCAAATGGAAACAATCAAGCAGTCATTGAGGCA  76101
               ******* ****************************************************

Dromedary      ACTGCTTGGCTTGAGCTCCAAGAATATCTTCCTGAAAAGCTGCATACTTCTCAGCAACTC  75957
Wild           ACTGCTTGGCTTGAGCTCCAAGAATATCTTCCTGAAAAGCTGCATACTTCTCAGCAACTC  75733
Domestic       ACTGCTTGGCTTGAGCTCCAAGAATATCTTCCTGAAAAGCTGCATACTTCTCAGCAACTC  76161
               ************************************************************

Dromedary      AGGAAATGGCATGAGTTATGTGGCTACCGGCTCCTTCCTTCTACCAGGAAACTCAGGTCA  76017
Wild           AGGAAATGGCATGAGTTATGTGGCTACCGGCTCCTTCCTTCTACCAGGAAACTCAGGTCA  75793
Domestic       AGGAAATGGCATGAGTTATGTGGCTACCGGCTCCTTCCTTCTACCAGGAAACTCAGGTCA  76221
               ************************************************************

Dromedary      TCTTGGGCTCTGAAGCAGGCCAGGTGCCATGGGACAGAAATTATGCCAAGAAAGAGCAGA  76077
Wild           TCTTGGGCTCTGAAGCAGGCCAGGTGCCATGGGACAGAAATTATGCCAAGAAAGAGCAGA  75853
Domestic       TCTTGGGCTCTGAAGCAGGCCAGGTGCCATGGGACAGAAATTATGCCAAGAAAGAGCAGA  76281
               ************************************************************

Dromedary      TGAGAGTAGTCTTCTGTACTTATGGTTGCGCCTTGGCAGCAAGTAAAATTGTCCCAAAGT  76137
Wild           TGAGAGTAGTCTTCTGTACTTATGGTTGTGCCTTGGCAGCAAGTAAAATTGTCCCAAAGT  75913
Domestic       TGAGAGTAGTCTTCTGTACTTATGGTTGTGCCTTGGCAGCAAGTAAAATTGTCCCAAAGT  76341
               **************************** *******************************

Dromedary      CTGGAATCTACTGGGACTTATGCCTTATAGTAAACTTAAGTTCCTTACAGCTCCAGAGTC  76197
Wild           CTGGAATCTACTGGGACTTATGCCTTATAGTAAACTTAAGTTCCTTACAGCTCCAGAGTC  75973
Domestic       CTGGAATCTACTGGGACTTATGCCTTATAGTAAACTTAAGTTCCTTACAGCTCCAGAGTC  76401
               ************************************************************

Dromedary      GGACAGAATTACAAGAATTTGCAAAAGACTGATATGGGTTCCTGTCTGGGTTCCCATCTA  76257
Wild           GGACAGAATTACAAGAATTTGCAAAAGACTGATATGGGTTCCTGTCTGGGTTCCCATCTA  76033
Domestic       GGACAGAATTACAAGAATTTGCAAAAGACTGATATGGGTTCCTGTCTGGGTTCCCATCTA  76461
               ************************************************************

Dromedary      CTCATAGGCCAAAATGCATCCCTATGGCTGAAACAAACTGCACCAGGTGGGACTCTCCTG  76317
Wild           CTCATAGGCCAAGATGCATCCCTATGGCTGAAACAAACTGCACCAGGTGGGACTCTCCTG  76093
Domestic       CTCACAGGCCAAGATGCATCCCTATGGCTGAAACAAACTGCACCAGGTGGGACTCTCCTG  76521
               **** ******* ***********************************************

Dromedary      GGAGAACAGCCCTGCAAGGAAGTGCCAGCAAAGCTCTCTCAGAGGTGGGTAGACAATAGT  76377
Wild           GGAGAACAGCCCTGCAAGGAAGTGCCAGCAAA-CTCTCTCAGAGGTGGGTAGACAATAGT  76152
Domestic       GGAGAACAGCCCTGCAAGGAAGTGCCAGCAAAGCTCTCTCAGAGGTGGGTAGACAATAGT  76581
               ******************************** ***************************
```

```
Dromedary      GAGCCCCACCTTGCTTGACCAGATGTTTGATCAAAGATTCCTGGTACTGGCCAAAGAGAG  76437
Wild           GAGCCCCACCTTGCTTGACCAGATGTTTGATCAAAGATTCCTGGTACTGGCCAAAGAGAG  76212
Domestic       GAGCCCCACCTTGCTTGACCAGATGTTTGATCAAAGATTCCTGGTACTGGCCAAAGAGAG  76641
               ************************************************************

Dromedary      CAGTGTCATTTGAGGGCAAAATTTTTCCCAAAGGCTAAAATTTGGCTTTATACCATGTAC  76497
Wild           CAGTGTCATTTGAGGGCAAAATTTTTCCCAAAGGCTAAAATTTGGCTTTATACCATGTAC  76272
Domestic       CAGTGTCATTTGAGGGCAAAATTTTTCCCAAAGGCTAAAATTTGGCTTTATACCATGTAC  76701
               ************************************************************

Dromedary      TGACACACCATGTATTGTGTTGACACAACATACCGGTTAGCCAGGTCTCATATATCCTGT  76557
Wild           TGACACACCATGTATTGTGTTGACACAACATACCGGTTAGCCAGGTCTCATATATCCTGT  76332
Domestic       TGACACACCATGTATTGTGTTGACACAACATACCGGTTAGCCAGGTCTCATATATCCTGT  76761
               ************************************************************

Dromedary      TCAGCTTGGCAAGAATAAACTACCCATAGGATCTTGGACAAGAAAAGCATGCACATTTAG  76617
Wild           TCAGCTTGGCAAGAATAAACTACCCATAGGATCTTGGACAAGAAAAGCATGCACATTTAG  76392
Domestic       TCAGCTTGGCAAGAATAAACTACCCATAGGATCTTGGACAAGAAAAGCATGCACATTTAG  76821
               ************************************************************

Dromedary      TTACAAAAGAAAAGCACATTATATAGGTATTATTATCTCCATTTTTAAAATGAGGAAGTT  76677
Wild           TTACAAAAGAAAAGCACATTATATAGGTATTATTATCTCCATTTTTAAAATGAGGAAGTT  76452
Domestic       TTACAAAAGAAAAGCACATTATATAGGTATTATTATCTCCATTTTTAAAATGAGGAAGTT  76881
               ************************************************************

Dromedary      GAGGTTCAGAGATGGTTGTGACTAATGAGTGACTAAGACAGGTGAATACATAATGGGAGA  76737
Wild           GAGGTTCAGAGATGGTTGTGACTAATGAGTGACTAAGACAGGTGAATACATAATGGGAGA  76512
Domestic       GAGGTTCAGAGATGGTTGTGACTAATGAGTGACTAAGACAGGTGAATACATAATGGGAGA  76941
               ************************************************************

Dromedary      AATACAAATACAAATGCAGCAGTATAATTTTTCAGCTATTAGTTCAAATGTGAAAAAAAT  76797
Wild           AATACAAATACAAATGCAGCAGTATAATTTTTCAGCTATTAGTTCAAATGTGAAAAAAAT  76572
Domestic       AATACAAATACAAATGCAGCAGTATAATTTTTCAGCTATTAGTTCAAATGTGAAAAAAAT  77001
               ************************************************************

Dromedary      ACAATTTTCTGATTGTTGCAAAACTGATGTCAGCTTTTACCTCAAGCTTCCTGGTGCTAT  76857
Wild           ACAATTTTCTGATTGTTGCAAAACTGATGTCAGCTTTTACCTCAAGCTTCCTGGTGCTAT  76632
Domestic       ACAATTTTCTGATTGTTGCAAAACTGATGTCAGCTTTTACCTCAAGCTTCCTGGTGCTAT  77061
               ************************************************************

Dromedary      AACACCATTTCTCCTTTATAAAGGCAACACTCTGATGTCAATGGAAACTGCTCTAATAAT  76917
Wild           AACACCATTTCTCCTTTATAAAGGCAACACTCTGATGTCAATGGAAACTGCTCTAATAAT  76692
Domestic       AACACCATTTCTCCTTTATAAAGGCAACACTCTGATGTCAATGGAAACTGCTCTAATAAT  77121
               ************************************************************

Dromedary      GCTTATCATCGCTCTGGACCTCACCGAGAAAATGATACAGAATATTCCCCTGCTCTCACA  76977
Wild           GCTTATCATCACTCTGGACCTCACCGAGAAAATGATACAGAATATTCCCCTGCTCTCACA  76752
Domestic       GCTTATCATCACTCTGGACCTCACCGAGAAAATGATACAGAATATTCCCCTGCTCTCACA  77181
               ********** *************************************************

Dromedary      TCTCCACACTTACAGGAGGACTGTAATGTGTCTCTCCTCCTGACATCCAGCTCAGTCTGT  77037
Wild           TCTCCACACTTACAGGAGGACTGTAATGTGTCTCTCCTCCTGACATCCAGCTCAGTCTGT  76812
Domestic       TCTCCACACTTACAGGAGGACTGTAATGTGTCTCTCCTCCTGACATCCAGCTCAGTCTGT  77241
               ************************************************************

Dromedary      GAAGCATGACACCAAAAAGGATCACTGTCCGAAATTTTTCTCCTCCCATCAAATTCACTC  77097
Wild           GAAGCATGACACCAAAAA-GATCACTGTCCGAAATTTTTCTCCTCCCATCAAATTCACTC  76871
Domestic       GAAGCATGACACCAAAAA-GATCACTGTCCGAAATTTTTCTCCTCCCATCAAATTCACTC  77300
               ****************** *****************************************

Dromedary      CCATCATAAGCTCATGAGATCTTTATGTGGGGAGATATAGAAATAGGTTCTGGCTTAACT  77157
Wild           CCATCATAAGCTCATGAGATCTTTATGTGGGGAGATATAGAAATAGGTTCTGGCTTAACT  76931
Domestic       CCATCATAAGCTCATGAGATCTTTATGTGGGGAGATATAGAAATAGGTTCTGGCTTAACT  77360
               ************************************************************

Dromedary      GAATCTATTTACCAACACTTCATATTTCATACCTGAACCCTAGACCCTTGAATTTAAAAT  77217
Wild           GAATCTATTTACCAACACTTCATATTTCATACCTGAACCCTAGACCCTTGAATTTAAAAT  76991
Domestic       GAATCTATTTACCAACACTTCATATTTCATACCTGAACCCTAGACCCTTGAATTTAAAAT  77420
               ************************************************************
```

```
Dromedary      TCTGCTAAATACTTGCTATCATTTTTTTTTTCTGATTCTGATCTGTAAAGTACCCCCTGG  77277
Wild           TCTGCTAAATACTTGCTATCATTTTTTTTTTCTGATTCTGATCTGTAAAGTACCCCCTGG  77051
Domestic       TCTGCTAAATACTTGCTATCATTTTTTTTTTCTGATTCTGATCTGTAAAGTACCCCCTGG  77480
               ************************************************************

Dromedary      AAAAATATAACCTTTTAATGAATCAGTGTCCATTCTAATACTACAAAGGTAAATTAAATT  77337
Wild           AAAAATATAACCTTTTAATGAATCAGTGTCCATTCTAATACTACAAAGGTAAATTAAATT  77111
Domestic       AAAAATATAACCTTTTAATGAATCAGTGTCCATTCTAATACTACAAAGGTAAATTAAATT  77540
               ************************************************************

Dromedary      ACATATTATCCTTTCATCTACAAATGCACTAAATTTTTATGTTACTATCTAGTATTATTA  77397
Wild           ACATATTATCCTTTCACCTACAAATGCACTAAATTTTTATGTTACTATCTAGTATTATTA  77171
Domestic       ACATATTATCCTTTCACCTACAAATGCACTAAATTTTTATGTTACTATCTAGTATTATTA  77600
               **************** *******************************************

Dromedary      GTAAGGTGATCAGCAATGAACACTTTATACATTGCCTGTAGAAAAGTCCTGATCAGTTGC  77457
Wild           GTAAGGTGATCAGCAATGAACACTTTATACATTGCCTGTAGAAAAGTCCTGATCAGTTGC  77231
Domestic       GTAAGGTGATCAGCAATGAACACTTTATACATTGCCTGTAGAAAAGTCCTGATCAGTTGC  77660
               ************************************************************

Dromedary      CTTCCAATATTTAACAAGATCCTTAATGTTTCTATACAGTGATTTTACTTCTAAAATTTT  77517
Wild           CTTCCAATATTTAACAAGATCCTTAATGTTTCTATACAGTGATTTTACTTCTAAAATTTT  77291
Domestic       CTTCCAATATTTAACAAGATCCTTAATGTTTCTATACAGTGATTTTACTTCTAAAATTTT  77720
               ************************************************************

Dromedary      ATCCAAAGAAAACAAAGATGTAAATATTTATGTTAAATCACAATGTTATTTACTGTTCTT  77577
Wild           ATCCAAAGAAAACAAAGATGTAAATATTTATGTTAAATCACAATGTTATTTACTGTTCTT  77351
Domestic       ATCCAAAGAAAACAAAGATGTAAATATTTATGTTAAATCACAATGTTATTTACTGTTCTT  77780
               ************************************************************

Dromedary      TAAAACTAGAAATAATGTATCCAACACTAGGGAATGGTTACATTGATTATGATAGATAAT  77637
Wild           TAAAACTAGAAATAATGTATCCAACACTAGGGAATGGTTACGTTGATTATGATAGATAAT  77411
Domestic       TAAAACTAGAAATAATGTATCCAACACTAGGGAATGGTTACGTTGATTATGATAGATAAT  77840
               ***************************************** ******************

Dromedary      AATAGCATATTAAAATTTAAATACAACCTGAGGGCCCAGCAAACACCATGAGGATGAGAG  77697
Wild           AATAGCATATTAAAATTTAAATACAACCTGAGGGCCCAGCAAACACCATGAGGATGAGAG  77471
Domestic       AATAGCATATTAAAATTTAAATACAACCTGAGGGCCCAGCAAACACCATGAGGATGAGAG  77900
               ************************************************************

Dromedary      TGAGAGGTAGAAAAAAACTAAGCCTTCACTGATATTATTGAGATACTGATCTTACTGAAC  77757
Wild           TGAGAGGTAGAAAAAAACTAAGCCTTCACTGATATTATTGAGATACTGATCTTACTGAAC  77531
Domestic       TGAGAGGTAGAAAAAAACTAAGCCTTCACTGATATTATTGAGATACTGATCTTACTGAAC  77960
               ************************************************************

Dromedary      CTAGAGCTGCCTTACATCTGGACTTGTAGTTATATGAGATACTGTATTTTTCTACTGCTT  77817
Wild           CTAGAGCTGCCTTACATCTGGACTTGTAGTTATATGAGATACTGTATTTTTCTACTGCTT  77591
Domestic       CTAGAGCTGCCTTACATCTGGACTTGTAGTTATATGAGATACTGTATTTTTCTACTGCTT  78020
               ************************************************************

Dromedary      AAGCTGTATTTGACTTGGGATTTCTGTTATTTACAGCTGAAATATTCTAAATAAGTAAAT  77877
Wild           AAGCTGTATTTGACTTGGGATTTCTGTTATTTACAGCTGAAATATTCTAAATAAGTAAAT  77651
Domestic       AAGCTGTATTTGACTTGGGATTTCTGTTATTTACAGCTGAAATATTCTAAATAAGTAAAT  78080
               ************************************************************

Dromedary      ATGTGTACAGCATGTATATACATACATATATCAGATATCTATGATTACCTTTAATTTTAA  77937
Wild           ATGTGTACAGCATGTATATACATACATATATCAGATATCTATGATTACCTTTAATTTTAA  77711
Domestic       ATGTGTACAGCATGTATATACATACATATATCAGATATCTATGATTACCTTTAATTTTAA  78140
               ************************************************************

Dromedary      CAGAGATGCCAATTTGTACTCTGTAGAGGTTTTATAAATTTATATTTCCATTAATAATGT  77997
Wild           CAGAGATGCCAATTTGTACTCTGTAGAGGTTTTATAAATTTATATTTCCATTAATAATGT  77771
Domestic       CAGAGATGCCAATTTGTACTCTGTAGAGGTTTTATAAATTTATATTTCCATTAATAATGT  78200
               ************************************************************

Dromedary      ATGAGGTTATCTATTTTCATACACCCTCATCATTTCTATTTCTAGTCTGATAGGTGAAAA  78057
Wild           ATGAGGTTATCTATTTTCATACACCCTCATCATTTCTATTTCTAGTCTGATAGGTGAAAA  77831
Domestic       ATGAGGTTATCTATTTTCATACACCCTCATCATTTCTATTTCTAGTCTGATAGGTGAAAA  78260
               ************************************************************
```

```
Dromedary      ATGGTATCTGATCATGATTTTAATCATATTTTCTTATTTTGAATGAGGTTATTAACATTT 78117
Wild           ATGGTATCTGATCATGATTTTAATCATATTTTCTTATTTTGAATGAGGTTATTAACATTT 77891
Domestic       ATGGTATCTGATCATGATTTTAATCATATTTTCTTATTTTGAATGAGGTTATTAACATTT 78320
               ************************************************************

Dromedary      TCATGTTTAAGGATTATCTGTACTTCCTTTTCTATTAACTGTTTGCTTTTATCCTTTGCT 78177
Wild           TCATGTTTAAGGATTATCTGTACTTCCTTTTCTATTAACTGTTTGCTTTTATCCTTTGCT 77951
Domestic       TCATGTTTAAGGATTATCTGTACTTCCTTTTCTATTAACTGTTTGCTTTTATCCTTTGCT 78380
               ************************************************************

Dromedary      TATTTTTTTCTACTAAGTTGTTGACATTAAAATAAAACCTATATACTTAAGATATTAGCT 78237
Wild           TATTTTTTTCTACTAAGTTGTTGACATTAAAATAAAACCTATATACTTAAGATATTAGCT 78011
Domestic       TATTTTTTTCTACTAAGTTGTTGACATTAAAATAAAACCTATATACTTAAGATATTAGCT 78440
               ************************************************************

Dromedary      ACAACTATTCCCAGATTTTCATTTGTCTTTTGACTTTGTGGTTTCCTGTTTTTTAATGCA 78297
Wild           ACAACTATTCCCAGATTTTCATTTGTCTTTTGACTTTGTGGTTTCCTGTTTTTTAATGCA 78071
Domestic       ACAACTATTCCCAGATTTTCATTTGTCTTTTGACTTTGTGGTTTCCTGTTTTTTAATGCA 78500
               ************************************************************

Dromedary      GAATTAAAATATTTGTATGATGTTACTTACATATTTTAGTCTTTTTTATGACTTCAAGTT 78357
Wild           GAATTAAAATATTTGTATGATGTTACTTACATATTTTAGTCTTTTTTATGACTTCAAGTT 78131
Domestic       GAATTAAAATATTTGTATGATGTTACTTACATATTTTAGTCTTCTTTATGACTTCAAGTT 78560
               ******************************************* ****************

Dromedary      TTTGTCATACTTAGAGTCTTTTACATATATGCTTATATTTCTAATATATCATATTTTCTT 78417
Wild           TTTGTCATACTTAGAGTCTTTTACATATATGCTTATATTTCTAATATATCATATTTTCTT 78191
Domestic       TTTGTCATACTTAGAGTCTTTTACATATATGCTTATATTTCTAATATATCATATTTTCTT 78620
               ************************************************************

Dromedary      CTAATATTTTTATGGTTTGTTATTTACTTTTAAAATCTTTAATCCATGTCAACTCTATTT 78477
Wild           CTAATATTTTTATGGTTTGTTATTTACTTTTAAAATCTTTAATCCATGTCAACTCTATTT 78251
Domestic       CTAATATTTTTATGGTTTGTTATTTACTTTTAAAATCTTTAATCCATGTCAACTCTATTT 78680
               ************************************************************

Dromedary      CATAGTGTGAGGTAGGAACCAATTTACTTTTCTTCAGATGACTATCCATTTGTCACCATT 78537
Wild           CATAGTGTGAGGTAGGAACCAATTTACTTTTCTTCAGATGACTATCCATTTGTCACCATT 78311
Domestic       CATAGTGTGAGGTAGGAATCAATTTACTTTTCTTCAGATGACTATCCATTTGTCACCATT 78740
               ****************** *****************************************

Dromedary      TCTAATACTATCTTCCCCTTAGTGATTTGAAATGTGGAAGGGTGCTTAGTGGACACATTC 78597
Wild           TCTAATACTATCTTCCCCTTAGTGATTTGAAATGTGGAAGGGTGCTTAGTGGACACATTC 78371
Domestic       TCTAATACTATCTTCCCCTTAGTGATTTGAAATGTGGAAGGGTGCTTAGTGGACACATTC 78800
               ************************************************************

Dromedary      TGACCACATGGATCCTTAGATCCAAAAAGTCATGTTCATAGAATTTAGCAATAATATGTG 78657
Wild           TGACCACATGGATCCTTAGATCCAAAAAGTCATGTTCATAGAATTTAGCAATAATATGTG 78431
Domestic       TGACCACATGGATCCTTAGATCCAAAAAGTCATGTTCATAGAATTTAGCAATAATATGTG 78860
               ************************************************************

Dromedary      ATGCCTAGGGTCAAATTAGTTACTCTCTTGAAATCTAACTACAATCAATACTAATGAATA 78717
Wild           ATGCCTAGGGTCAAATTAGTTACTCTCTTGAAATCTAACTACAATCAATACTAATGAGTA 78491
Domestic       ATGCCTAGGGTCAAATTAGTTACTCTCTTGAAATCTAACTACAATCAATACTAATGAGTA 78920
               ********************************************************* **

Dromedary      TGTAAACAGTAGAATGATTGATGATAACAGGGTTCCTGTCCAGTCAGAAATGACCCGATA 78777
Wild           TGTAAACAGTAGAATGATTGATGATAACAGGGTTCCTGTCCAGTCAGAAATGACCCGATA 78551
Domestic       TGTAAACAGTAGAATGATTGATGATAACAGGGTTCCTGTCCAGTCAGAAATGACCCGATA 78980
               ************************************************************

Dromedary      CATTCATGCATATTCATATTCATGCATTTATTCCATCACAAATTTTTAGTCTACTATTGT 78837
Wild           CATTCATGCATATTCATATTCATGCATTTATTCCATCACAAATTTTTAGTCTACTATTGT 78611
Domestic       CATTCATGCATATTCATATTCATGCATTTATTCCATCACAAATTTTTAGTCTACTATTGT 79040
               ************************************************************

Dromedary      TGTATTTTAATTCCTACTTCACTTTAGGTATGGGACATTTTGTATAGGACAGTGTCCCTC 78897
Wild           TGTATTTTAATTCCTACTTCACTTTAGGTATGGGACATTTTGTATAGGACAGTGTCCCTC 78671
Domestic       TGTATTTTAATTCCTACTTCACTTTAGGTATGGGACATTTTGTATAGGACAGTGTCCCTC 79100
               ************************************************************
```

```
Dromedary     ACCTCTACTGAAGCCAAGCTCCCTTTGTGTCACAACTCCGGAGGAGAAAATATTACACAT  78957
Wild          ACCTCTACTGAAGCCAAGCTCCCTTTATGTCACAACTCCGGAGGAGAAAATATTACACAT  78731
Domestic      ACCTCTACTGAAGCCAAGCTCCCTTTATGTCACAACTCCGGAGGAGAAAATATTACACAT  79160
              ************************** *********************************

Dromedary     CCCTCCTCCCCTAAAGTCACAAAAACTTCTCAGTCGCATCACTCAATGTATTATTCAGCC  79017
Wild          CCCTCCTCCCCTAAAGTCACAAAAACTTCTCAGTCGCATCACTCAATGTATTATTCAGCC  78791
Domestic      CCCTCCTCCCCTAAAGTCACAAAAACTTCTCAGTCGCATCACTCAATGTATTATTCAGCC  79220
              ************************************************************

Dromedary     TCCCAAAGGATCAATGTCTTTCTTCCTCTGTTATCAGAAGAAGAATGCAAGAAAATTATT  79077
Wild          TCCCAAAGGATCAATGTCTTTCTTCCTCTGTTATCAGAAGAAGAATGCAAGAAAATTATT  78851
Domestic      TCCCAAAGGATCAATGTCTTTCTTCCTCTGTTATCAGAAGAAGAATGCAAGAAAATTATT  79280
              ************************************************************

Dromedary     CAGAGGGCCTCTGGTACTTTTCCTCTGAAATAACATATGAATTCTGTTCATATTTGTATG  79137
Wild          CAGAGGGCCTCTGGTACTTTTCCTCTGAAATAACATATGAATTCTGTTCATATTTGTATG  78911
Domestic      CAGAGGGCCTCTGGTACTTTTCCTCTGAAATAACATATGAATTCTGTTCATATTTGTATG  79340
              ************************************************************

Dromedary     CTAGCCTATCAGATTTTACCAATTCATTGAGTCCTACATACCATCGCAGAAACTGTAGAG  79197
Wild          CTAGCCTATCAGATTTTACCAATTCATTGAGTCCTACATACCATCGCAGAAACTGTAGAG  78971
Domestic      CTAGCCTATCAGATTTTACCAATTCATTGAGTCCTACATACCATCGCAGAAACTGTAGAG  79400
              ************************************************************

Dromedary     AGAGAAGAGGAGAGAAATAAATTACTGTTTTTCTCTTGAGAAGCTCACAGTCTAGTGAGA  79257
Wild          AGAGAAGAGGAGAGAAATAAATTACTGTTTTTCTCTTGAGAAGCTCACAGTCTAGTGAGA  79031
Domestic      AGAGAAGAGGAGAGAAATAAATTACTGTTTTTCTCTTGAGAAGCTCACAGTCTAGTGAGA  79460
              ************************************************************

Dromedary     GCAGAAAAGCACACTGTCAACTTTAATAAAATGTGACAAATGGTTTACAAGGGGTGCTGT  79317
Wild          GCAGAAAAGCACACTGTCAACTTTAATAAAATGTGACAAATGGTTTACAAGGGGTGCTGT  79091
Domestic      GCAGAAAAGCACACTGTCAACTTTAATAAAATGTGACAAATGGTTTACAAGGGGTGCTGT  79520
              ************************************************************

Dromedary     GGGAGAGAAGAGGAAAATTACCTCAGTCATACTAGGAGTTCACTAGAGACTTCAAGGAGA  79377
Wild          GGGAGAGAAGAGGAAAATTACCTCAGTCATACTAGGAGTTCACTAGAGACTTCAAGGAGA  79151
Domestic      GGGAGAGAAGAGGAAAATTACCTCAGTCATACTAGGAGTTCACTAGAGACTTCAAGGAGA  79580
              ************************************************************

Dromedary     AGGTGATGTTAGCAATGTCTTAAATAATGAATAAAAGTTTGATGGATGGGTAAGGAGGAA  79437
Wild          AGGTGATGTTAGCAATGTCTTAAATAATGAATAAAAGTTTGATGGATGGGTAAGGAGGAA  79211
Domestic      AGGTGATGTTAGCAATGTCTTAAATAATGAATAAAAGTTTGATGGATGGGTAAGGAGGAA  79640
              ************************************************************

Dromedary     AAATAAATGAGGAAGAAGAAGGACATTCCAAGACAACACCCCCAACCCCCCCCCCCnACA  79497
Wild          AAATAAATGAGGAAGAAGAAGGACATTCCAAGACAACACCCCCA----------------  79255
Domestic      AAATAAATGAGGAAGAAGAAGGACATTCCAAGACAAC-----------------------  79677
              *************************************
```
