# Supplementary material for: Beyond the Big Five: Investigating Myostatin Structure, Polymorphism and Expression in Camelus dromedarius
Source: Front Genet. 2019 Jun 7;10:502. doi: 10.3389/fgene.2019.00502 (PMC6566074; doi:10.3389/fgene.2019.00502)
Supplement: FIGURE S4 — Putative SNPs in the myostatin coding region, as inferred from alignment among previously published myostatin sequences and sequences generated in this study. (A) Consensus sequence of the myostatin coding region. Highlighted, in yellow, the exonic region, and, in red, the variant sites. Numbering of polymorphism positions refers to the contig AGVR01040332. (B) Summary table of the inferred polymorphisms showing the nature of the putative variants, together with the reference literature, and the prediction of the variant effects (in case of a missense mutation, the alternative amino acids are reported, otherwise “none” is entered). [file Image_4.pdf]

Supplementary Figure S4.

Camelus dromedarius MSTN coding region (numbering based on *Camelus ferus* MSTN couting AGVR01040332)

A

53241CTCTCAGGCTGTGCAGGCATTAAAATTTTGCTTGGCATTACTCAAAAGCAAAAGAAAAATAAAAGGAAGAAATAA
GAACAAGGGAAAAGATTGTCTTGATTTTAAAATC**ATG**CAAAAACTGCAAATCTATGTTTATATTTACCTGTTTATGCTGA
TTGTTGCTGGTCCAGTGGATCTGAATGAGAACA**A**CGA**A**CAAAAAGAAAATGTGGAAAAAGAGGGGCTGTGTAATGCATGT
ATGTGGAGACAAAACACTAAATCTTCAAGACTAGAAGCTATAAAAATTCAAATCCTCAGTAAACTTCGCCTGGAAACAGC
TCCTAACATCAGCAAAGATGCTATAAGACAACTTTTGCCCAAAGCTCCTCCGCTCCGGGAACTGATTGATCAGTACGATG
TCCAGAGAGATGACAGCAGTGA**T**GGCTCCTTGGAAGATGATGATTACCACGCTACGACGGAAACAATCATTACCATGCC**T**
ACAGAGTCTGATCTTCTA**ATGC**AAGT**AG**AAG**G**AAAACCCAAATGTTGCTTCTTTAAGTTTAGCTCTAAAATACAATACAA
TAAAGTAGTAAAGGCCCAATTGTGGATCTATCTGAGACCCGTACAGACTCCTACAACAGTGTTTGTGCAAATCCTGAGAC
TCATCAAACCCATGAAAGACGGTACAAGGTATACTGGAATCCGATCTCTGAAACTTGACATGAACCCAGGCACTGGTATT
TGGCAGAGCATTGATGTGAAGACAGTGTTGCAAAATTGGCTCAAACAACCTGAATCCAACTTAGGCATTGAAATCAAAGC
TTTAGATGAGAATGGTCATGATCTTGCTGTAACCTTCCCAG**G**ACCAGGAGAAGA**T**GG**TT**TGAATCCCTTTTTAGAAGTCA
AGGTAACAGACACACCAAAAAGATCCAGGAGAGATTTTGGACTTGACTGTGATGAGCACTCAACAGAATCTCGATGCTGT
CGATACCCTCTAACTGTGGATTTTGAAGCTTTTGGATGGGATTGGATTATTGCACCTAAGAGATATAAGGCCAATTACTG
CTCTGGAGAGTGTGAATTTGTATTTTTACAAAAATATCCTCATACTCACCTTGTGCACCAAGCAAACCCCAGAGGTTCGG
CAGGTCCCTGCTGTACTCCCACAAAGATGTCTCCAATTAATATGCTATATTTTAATGGCAAAGAACAAATAATATATGGG
AAAATTCCAGCTATGGTAGTAGATCGCTGTGGGTGCTCA**TGA**GGTTTCTATTTGGTTCA**T**AACTTC**C**TAAAATGTGGAAG
GTCTTCCCCTCAACAATTTTGAAACTGTGAAATTATATACCACAGGCTTTAAGCCTAGAGTATGCTACAGTCACTTAAGC
ACAAGCTACAGTATATGAACTAAAAGAGAGAATATATGCAATGGTTGGCATTTAACCATCAAAACAAATCATACAATAAA
AAGTTTTATGATTTCCT

In red, putative polymorphisms within *Camelus dromedaries* inferred from the alignment of publicly available sequences of myostatin (see table below for references) and the sequences generated in this study.

B

Position	This study	Others	Ref	Predict effect
189	A	G	Shah et al. 2006	Asn/Ser
193	A	G	Shah et al. 2006	none
418	T	C	Shah et al. 2006	none
475	T	C	Muzzachi et al. 2015	none
494-497	ATGG	GCAG	Agrawal et al 2017	Met/Ala Gln/Glu
502-503	AG	GC	Agrawal et al 2017	none Glu/Gln
507	G	A	Agrawal et al 2017	Gly/Glu
837	G	A	Agrawal et al 2017	Gly/glu
850	T	A	Agrawal et al 2017	Asp/Glu
853-854	TT	AC	Agrawal et al 2017	none none
1255	T	C	Muzzachi et al. 2015	
1262	C	A	Muzzachi et al. 2015	

Supplementary Figure S4. Putative SNPs in the myostatin coding region, as inferred from alignment among previously published myostatin sequences and sequences generated in this study. (A) Consensus sequence of the myostatin coding region. Highlighted, in yellow, the exonic region, and, in red, the variant sites. Numbering of polymorphism positions refers to the contig AGVR01040332. **(B)** Summary table of the inferred polymorphisms showing the nature of the putative variants, together with the reference literature, and the prediction of the variant effects (in case of a missense mutation, the alternative amino acids are reported, otherwise "none" is entered).
