# Supplementary material for: Beyond the Big Five: Investigating Myostatin Structure, Polymorphism and Expression in Camelus dromedarius
Source: Front Genet. 2019 Jun 7;10:502. doi: 10.3389/fgene.2019.00502 (PMC6566074; doi:10.3389/fgene.2019.00502)
Supplement: FIGURE S5 — Plot of the Bayesian clustering analysis perfoemed on the nine considered dromedaries using the 69 identified SNPs. The plot shows the results obtained for K = 7, that was identified as the most likely output by visual inspection of the probability values associated to each tested K value (from 1 to 9). Numbers indicate different samples (1, United Arab Emirates; 2, Qatar; 3-4-5, Kingdom of Saudi Arabia; 6, Austria; 7, Kenya; 8, Sudan; 9, Pakistan). Colors indicate the seven different clusters. The proportion of each individual sample in each inferred cluster is shown in the y-axis. [file Image_5.pdf]

Supplementary Figure S5.

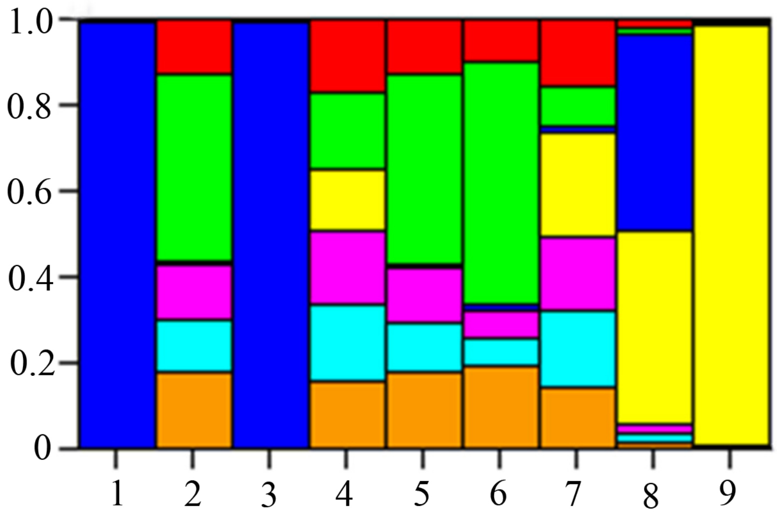

Supplementary Figure S5. Plot of the Bayesian clustering analysis perfoemed on the nine considered dromedaries using the 69 identified SNPs. The plot shows the results obtained for K = 7, that was identified as the most likely output by visual inspection of the probability values associated to each tested K value (from 1 to 9). Numbers indicate different samples (1, UAE; 2, Qatar; 3-4-5, Kingdom of Saudi Arabia; 6, Austria; 7, Kenya; 8, Sudan; 9, Pakistan). Colors indicate the seven different clusters. The proportion of each individual sample in each inferred cluster is shown in the y-axis
