# Supplementary material for: Beyond the Big Five: Investigating Myostatin Structure, Polymorphism and Expression in Camelus dromedarius
Source: Front Genet. 2019 Jun 7;10:502. doi: 10.3389/fgene.2019.00502 (PMC6566074; doi:10.3389/fgene.2019.00502)
Supplement: FIGURE S6 — Results of the Human Splicing Finder analysis for the three intronic SNPs detected in this study. (A) G66148C. (B) A66460G. (C) T66461C. [file Image_6.pdf]

Supplementary Figure S6

A

Predicted signal	Prediction algorithm	cDNA Position	Interpretation
ESE Site Broken	1 - RESCUE ESE Hexamers<br>2 - PESE Octamers from Zhang & Chasin<br>3 - EIEs from Zhang et al.<br>4 - HSF Matrices - 9G8<br>5 - ESR Sequences from Goren et al.	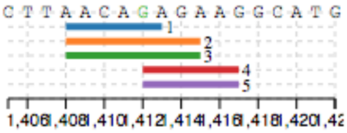	Alteration of an exonic ESE site. Potential alteration of splicing.

B

Predicted signal	Prediction algorithm	cDNA Position	Interpretation
New Donor Site	1 - HSF Matrices	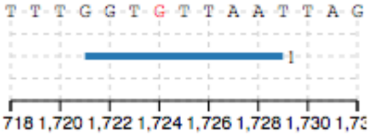	Activation of an exonic cryptic donor site. Potential alteration of splicing.
New ESS Site	1 - Sironi et al. - Motif 2<br>2 - IIEs from Zhang et al.<br>3 - Fas-ESS hexamers	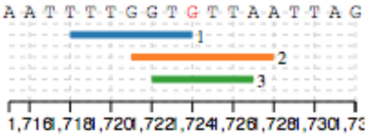	Creation of an exonic ESS site. Potential alteration of splicing.

C

No significant splicing motif alteration detected.
This mutation has probably no impact on splicing.

Supplementary Figure S6. Results of the Human Splicing Finder analysis for the three intronic SNPs detected in this study. (A) G66148C. **(B)** A66460G. **(C)** T66461C.
