# Supplementary material for: Beyond the Big Five: Investigating Myostatin Structure, Polymorphism and Expression in Camelus dromedarius
Source: Front Genet. 2019 Jun 7;10:502. doi: 10.3389/fgene.2019.00502 (PMC6566074; doi:10.3389/fgene.2019.00502)
Supplement: FIGURE S7 — Graphical outline of the major predicted regulatory motifs in the 1.5 kb proximal to the transcription initiation site of the C. dromedarius myostatin gene. TATA boxes (highlighted in yellow), E-boxes (highlighted in gray), CREBP1 (light green), CREB_01 (orange), MYOGNF1 (blue), CEBP_01 (purple), MEF (pink), MYOD (dark green), and the CCAAT box (highlighted in purple) are presented. [file Image_7.pdf]

Supplementary Figure S7

C_EBP CREBP1
GGCATCTGGTTTGTGTCTGGTTTTCCTTAATCTTTAATGATGGGCAAATCTAATGCATTATGTAAGGCCATTTTTTCTCA

MYOGNF1
AGAGATGTAGATACCTCTTAAGAATTTGATGAAAATGCATTAACTTTTCAGGCTACTGAGTTGCATTTTAGTGCACTGA

C_EBP
GGCAGTAAATTAGTGTACAATGTGCAAAAGTAGTGACCTAAAAAATAAATATTTGATATGAACCACTGCATTCTCTTG

GAAAAAAAAAAGTAATGGGTCAACTCTCTTAGGAGTCCTTAGCTTCCCAAAAAGGAGTAGGAAGAATAATCTCCTGTG

MYOGNF1 C_EBP
GCCTGGAAACAGCTTCTGTTTCTCGCTGGCTATGTTTGTTTAGCTCTTTAATAGTTCATTTGATTAGATCTTGTGGCTCC

CAAAGCTAAGGTTCAGAGTTTGATCCCTACAGAGGCCACTTAAATTTAGAGAACAAAAAGCTCTATTCTCTGCTCCCAG

MYOD MYOGNF1 MYOD
ACCTCACCCCAAATCCCTGCCAGGTGTCTGCCCTCTGGTCAAATGAGAAGCTGGCAAAGGGGTGCAAACCTATCACAG

C_EBP C_EBP
TGTAGGCAAACAGAAAAAGGGGCACCCTTCATTATGGTGCTGCTTCCCTTGTATGTGCTTACAATATTTGGATATAACT

C_EBP
TACAGAGAATAGAGCCTACATTTTTAACTCTTACCACTGGAAATCTGAGGCAAACTTCATTACCAGTCATAAAATTCAT

MYOD CREBP1 MYOD
TGTCTTCTCAGTTATTCTAAGCTTATTCTAAATTCAGGGAGCTGACATAATCCTCTTGGTAATAAACAATGAAAAACACA

TCTTCTGAGCAACATTAATCTGCAGCTTTAGGACAGGAAATAACTTAATAGTAATCAGTCAAAAGTTGAGCACAATCTT

MEF2 MYOD
CACATAAATAAAAGATATTAAATTATTTAAAAATAATTCTATGTGCAATATAGCATTAGGATCAGTATGATTTTCATCAT

MYOD
GTGCTAAGAATTTAGACAGGCAAATGAGTTTCTCAAATCATAGCTGAAAATATTTTACTAGTATTACAATCTTTTCAAAT

C_EBP MEF2 C_EBP MEF2 C_EBP
TCAGGACTTCCTAATTTAAATCTTTCCTAATTTAAATCTAAATATTTCCCTAATCACACAGAACTAAAAATAATTTAAGAC

MEF2
AGCAAATAAAAATTCTTTTTACTTCAAATGTTTGCCTAAATAATATAAAATCATTTTATTTTTGGAGGAAAAAAAATTTCA

C_EBP
ACTTTTAAAGTACGAAGTGTAAATTAAGATTTACTTAGATTATAATTTTAAAATTTCCCACATAAAGATTAATAAGATTT

C_EBP C_EBP
AAATGTAGTTTATATTAGTGTTAACATAGATTTTAATTTTTCAAATGTCACATAGATATTTCATTATTTGTAGATTTATTT

MYOD C_EBP CREB
CTTTTATGAAGTAGTCAAATGAATCAGCTCACCCTTGACTGTAACAAAATACTGTTTGGTGACTTGTGACAGACAGGGT

MYOD C_EBP MYOD
TTTAACCTCTGACAGCGAGATTCATTGTGGAGCAAGAGCCAATCATAGATCCTGACGACACTTGTCTCATCAAAGTTG

MYOD MYOD
GAATATAAAAAGCCACTTGGAATACAGTATAAAAGATTCACTGGTGTGGCAAGTTGT

Supplementary Figure S7. Graphical outline of the major predicted regulatory motifs in the 1.5 kb proximal to the transcription initiation site of the *C. dromedarius* myostatin gene. TATA boxes (highlighted in yellow), E-boxes (highlighted in grey), CREBP1 (light green), CREB_01 (orange), MYOGNF1 (blue), CEBP_01 (purple), MEF (pink), MYOD (dark green), and the CCAAT box (highlighted in purple) are presented.
