# Supplementary material for: Beyond the Big Five: Investigating Myostatin Structure, Polymorphism and Expression in Camelus dromedarius
Source: Front Genet. 2019 Jun 7;10:502. doi: 10.3389/fgene.2019.00502 (PMC6566074; doi:10.3389/fgene.2019.00502)
Supplement: FIGURE S8 — Graphical evaluation of the Digital Droplet PCR performance. (A) 2D-dot plot of fluorescent signals detected in the Digital Droplet PCR experiments. Fluorescence results are plotted as two-dimensional dot plots. Gray dots correspond to empty droplets. Blue dots correspond to droplets containing at least one copy of the sequence complementary to Probe 1. Green dots correspond to droplets containing at least one copy of the sequence complementary to Probe 2. Orange dots correspond to droplets containing at least one copy of the sequence complementary to Probe 1 and at least one copy of the sequence complementary to Probe 2 (double-positive droplets). The observed pattern is relatively well balanced and dot clouds are well separated, suggesting (i) the absence of significant bias in the amplification of the two regions targeted by the two considered probes (exon1/exon2, and exon2/exon3) and (ii) the applicability of the BIORAD proprietary auto-analysis tool for target quantification. (B) Number of droplets generated in the Digital Droplet PCR experiments. Results for eight dromedary skeletal muscles, each from two different animals (biological replicate), analyzed in duplicate (technical replicate) are shown. [file Image_8.pdf]

Supplementary Figure S8

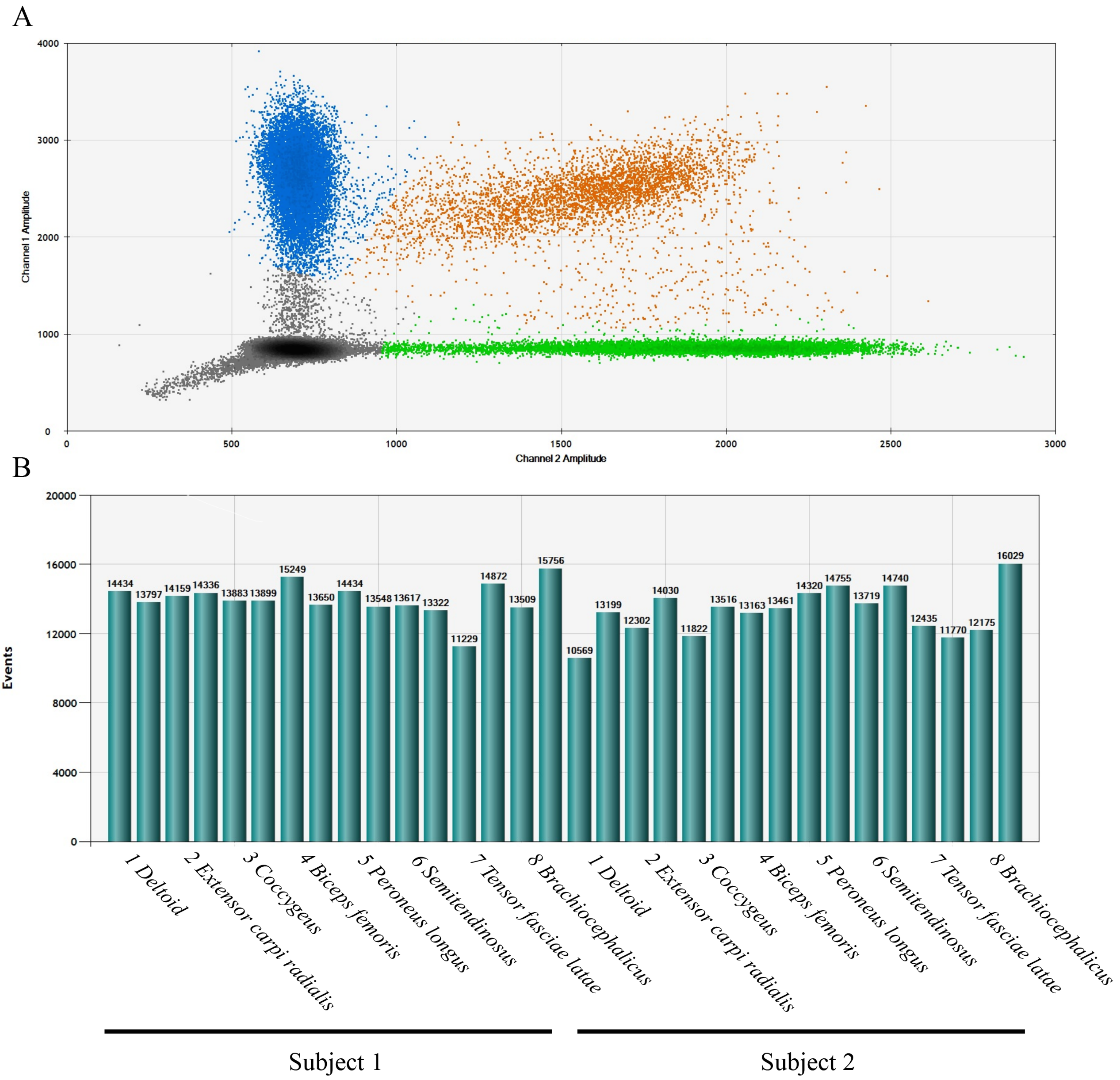

Supplementary Figure S8. Graphical evaluation of the Digital Droplet PCR performance. (A) 2D-dot plot of fluorescent signals detected in the Digital Droplet PCR experiments. Fluorescence results are plotted as two-dimensional dot plots. Grey dots correspond to empty droplets. Blue dots correspond to droplets containing at least one copy of the sequence complementary to Probe 1. Green dots correspond to droplets containing at least one copy of the sequence complementary to Probe 2. Orange dots correspond to droplets containing at least one copy of the sequence complementary to Probe 1 and at least one copy of the sequence complementary to Probe 2 (double-positive droplets). The observed pattern is relatively well balanced and dot clouds are well separated, suggesting (i) the absence of significant bias in the amplification of the two regions targeted by the two considered probes (exon1/exon2, and exon2/exon3) and (ii) the applicability of the BIORAD proprietary auto-analysis tool for target quantification. **(B)** Number of droplets generated in the Digital Droplet PCR experiments. Results for eight dromedary skeletal muscles, each from two different animals (biological replicate), analyzed in duplicate (technical replicate) are shown.
