# Supplementary material for: Beyond the Big Five: Investigating Myostatin Structure, Polymorphism and Expression in Camelus dromedarius
Source: Front Genet. 2019 Jun 7;10:502. doi: 10.3389/fgene.2019.00502 (PMC6566074; doi:10.3389/fgene.2019.00502)
Supplement: DATA SHEET S1 — Amino acidic sequences of the 83 non redundant myostatin proteins used for building the maximum likelihood tree presented in Figure 3. The data are in the “multiple sequence alignment” format. [file Data_Sheet_1.PDF]

>Camelus.dromedarius.XP_010990257
---------------------------------------------------MQKLQIYVY
IYLF-----------MLIVAGPVDLNEN--------------------------------
--------------------------------------------------------NEQK
ENVEKEGLCNACMWRQNTKSSRLEAIKIQILSKLRLETAPNISKDAIRQLLPKAPPLREL
IDQYDV-------QRDDS-----------------------------SDGSLEDDDYHAT
TETIITMPTESDLLMQVEGKPKCCFFKFSSKIQYNKVVKAQLWIYLRPVQ----------
-----------------------------------TPTTVFVQILRLIKPMKDGTRYTGI
RSLKLDMNPGTGIWQSIDVKTVLQNWLKQPESNLGIEIKALDE--------------NGH
DLAVTFPGPGEDGLNPFLEVKVT-----DTPKRSRRDF-GLDCDEHSTESRCCRYPLTVD
FEAFGWDWIIAPKRYKANYCSGECEFVFLQKYPHTHLVHQANPRGSAGPCCTPTKMSPIN
MLYFNGKEQIIYGKIPAMVVDRCGCS
>Vicugna.pacos.XP_006210045
---------------------------------------------------MQKLQIYVY
IYLF-----------MLIVAGPVDLNEN--------------------------------
--------------------------------------------------------NEQK
ENVEKEGLCNACMWRQNTKSSRLEAIKIQILSKLRLETAPNISKDAIRQLLPKAPPLREL
IDQYDV-------QRDDS-----------------------------SDGSLEDDDYHAT
TETIITMPTESDLLMQVEGKPKCCFFKFSSKIQYNKVVKAQLWIYLRPVQ----------
-----------------------------------TPTTVFVQILRLIKPMKDGTRYTGI
RSLKLDMNPGTGIWQSIDVKTVLQNWLKQPESNLGIEIKALDE--------------NGH
DLAVTFPGPGEDGLNPFLEVKVT-----DTPKRSRRDF-GLDCDEHSTESRCCRYPLTVD
FEAFGWDWIIAPKRYKANYCSGECEFVFLQKYPHTHLVHQANPRGSAGPCCTPTKMSPIN
MLYFNGKEQIIYGKIPAMVVDRCGCS
>Camelus.bactrianus.XP_010958338
---------------------------------------------------MQKLQIYVY
IYLF-----------MLIVAGPVDLNEN--------------------------------
--------------------------------------------------------NEQK
ENVEKEGLCNACMWRQNTKSSRLEAIKIQILSKLRLETAPNISKDAIRQLLPKAPPLREL
IDQYDV-------QRDDS-----------------------------SDGSLEDDDYHAT
TETIITMPTESDLLMQVEGKPKCCFFKFSSKIQYNKVVKAQLWIYLRPVQ----------
-----------------------------------TPTTVFVQILRLIKPMKDGTRYTGI
RSLKLDMNPGTGIWQSIDVKTVLQNWLKQPESNLGIEIKALDE--------------NGH
DLAVTFPGPGEDGLNPFLEVKVT-----DTPKRSRRDF-GLDCDEHSTESRCCRYPLTVD
FEAFGWDWIIAPKRYKANYCSGECEFVFLQKYPHTHLVHQANPRGSAGPCCTPTKMSPIN
MLYFNGKEQIIYGKIPAMVVDRCGCS
>Camelus.ferus.XP_006189285
---------------------------------------------------MQKLQIYVY
IYLF-----------MLIVAGPVDLNEN--------------------------------
--------------------------------------------------------NEQK
ENVEKEGLCNACMWRQNTKSSRLEAIKIQILSKLRLETAPNISKDAIRQLLPKAPPLREL
IDQYDV-------QRDDS-----------------------------SDGSLEDDDYHAT
TETIITMPTESDLLMQVEGKPKCCFFKFSSKIQYNKVVKAQLWIYLRPVQ----------
-----------------------------------TPTTVFVQILRLIKPMKDGTRYTGI
RSLKLDMNPGTGIWQSIDVKTVLQNWLKQPESNLGIEIKALDE--------------NGH
DLAVTFPGPGEDGLNPFLEVKVT-----DTPKRSRRDF-GLDCDEHSTESRCCRYPLTVD
FEAFGWDWIIAPKRYKANYCSGECEFVFLQKYPHTHLVHQANPRGSAGPCCTPTKMSPIN
MLYFNGKEQIIYGKIPAMVVDRCGCS
>Homo.sapiens.ABI48419
---------------------------------------------------MQKLQLCVY
IYLF-----------MLIVAGPVDLNEN--------------------------------
--------------------------------------------------------SEQK
ENVEKEGLCNACTWRQNTKSSRIEAIKIQILSKLRLETAPNISKDVIRQLLPKAPPLREL
IDQYDV-------QRDDS-----------------------------SDGSLEDDDYHAT
TETIITMPTESDFLMQVDGKPKCCFFKFSSKIQYNKVVRAQLWIYLRPVE----------
-----------------------------------TPTTVFVQILRLIKPMKDGTRYTGI
RSLKLDMNPGTGIWQSIDVKTVLQNWLKQPESNLGIEIKALDE--------------NGH
DLAVTFPGPGEDGLNPFLEVKVT-----DTPKRSRRDF-GLDCDEHSTESRCCRYPLTVD
FEAFGWDWIIAPKRYKANYCSGECEFVFLQKYPHTHLVHQANPRGSAGPCCTPTKMSPIN
MLYFNGKEQIIYGKIPAMVVDRCGCS

```
>Bos.taurus.NP_001001525
---------------------------------------------------MQKLQISVY
IYLF-----------MLIVAGPVDLNEN--------------------------------
--------------------------------------------------------SEQK
ENVEKEGLCNACLWRENTTSSRLEAIKIQILSKLRLETAPNISKDAIRQLLPKAPPLLEL
IDQFDV-------QRDAS-----------------------------SDGSLEDDDYHAR
TETVITMPTESDLLTQVEGKPKCCFFKFSSKIQYNKLVKAQLWIYLRPVK----------
-----------------------------------TPATVFVQILRLIKPMKDGTRYTGI
RSLKLDMNPGTGIWQSIDVKTVLQNWLKQPESNLGIEIKALDE--------------NGH
DLAVTFPEPGEDGLTPFLEVKVT-----DTPKRSRRDF-GLDCDEHSTESRCCRYPLTVD
FEAFGWDWIIAPKRYKANYCSGECEFVFLQKYPHTHLVHQANPRGSAGPCCTPTKMSPIN
MLYFNGEGQIIYGKIPAMVVDRCGCS
>Bubalus.bubalis.NP_001277896
---------------------------------------------------MQKLQISVY
IYLF-----------MLIVAGPVDLNEN--------------------------------
--------------------------------------------------------SEQK
ENVEKEGLCNACLWRENTTSSRLEAIKIQILSKLRLETAPNISKDAIRQLLPKAPPLLEL
IDQFDV-------QRDAG-----------------------------SDGSLEDDDYHAR
TDAVITMPTESDLLTQVEGKPKCCFFQFSSKIQYNKLVKAQLWIYLRPVK----------
-----------------------------------TPATVFVQILRLIKPMKDGTRYTGI
RSLKLDMNPGTGIWQSIDVKTVLQNWLKQPESNLGIEIKALDE--------------NGH
DLAVTFPEPGEDGLTPFLEVKVT-----DTPKRSRRDF-GLDCDERSTESRCCRYPLTVD
FEAFGWDWIIAPKRYKANYCSGECEFVFLQKYPHTHLVHQANPRGSAGPCCTPTKMSPIN
MLYFNGEGQIIYGKIPAMVVDRCGCS
>Bos.mutus.XP_005907489
---------------------------------------------------MQKLQISVY
IYLF-----------MLIVAGPVDLNEN--------------------------------
--------------------------------------------------------SEQK
ENVEKEGLCNACLWRENTTSSRLEAIKIQILSKLRLETAPNISKDAIRQLLPKAPPLLEL
IDQFDV-------QRDAS-----------------------------SDGSLEDDDYHAR
TETVITMPTESDLLTQVEGKPKCCFFKFSSKIQYNKLVKAQLWIYLRPVK----------
-----------------------------------TPATVFVQILRLIKPMKDGTRYTGI
RSLKLDMNPGTGIWQSIDVKTVLQNWLKQPESNLGIEIKALDE--------------NGH
DLAVTFPEPGEDGLTPFLEVKVT-----DTPKRSRRDF-GLDCDEHSTESRCCRYPLTVD
FEAFGWDWIIAPKRYKANYCSGECEFVFLQKYPHTHLVHQANPRGSAGPCCTPTKMSPIN
MLYFNGEGQIIYGKIPAMVVDRCGCS
>Bison.bison.XP_010861205
---------------------------------------------------MQKLQISVY
IYLF-----------MLIVAGPVDLNEN--------------------------------
--------------------------------------------------------SEQK
ENVEKEGLCNACLWRENTTSSRLEAIKIQILSKLRLETAPNISKDAIRQLLPKAPPLLEL
IDQFDV-------QRDAS-----------------------------SDGSLEDDDYHAR
TETVITMPTESDLLTQVEGKPKCCFFKFSSKIQYNKLVKAQLWIYLRPVK----------
-----------------------------------TPATVFVQILRLIKPMKDGTRYTGI
RSLKLDMNPGTGIWQSIDVKTVLQNWLKQPESNLGIEIKALDE--------------NGH
DLAVTFPEPGEDGLTPFLEVKVT-----DTPKRSRRDF-GLDCDEHSTESRCCRYPLTVD
FEAFGWDWIIAPKRYKANYCSGECEFVFLQKYPHTHLVHQANPRGSAGPCCTPTKMSPIN
MLYFNGEGQIIYGKIPAMVVDRCGCS
>Bos.indicus.XP_019825045
---------------------------------------------------MQKLQISVY
IYLF-----------MLIVAGPVDLNEN--------------------------------
--------------------------------------------------------SEQK
ENVEKEGLCNACLWRENTTSSRLEAIKIQILSKLRLETAPNISKDAIRQLLPKAPPLLEL
IDQFDV-------QRDAS-----------------------------SDGSLEDDDYHAR
TETVITMPTESDLLTQVEGKPKCCFFKFSSKIQYNKLVKAQLWIYLRPVK----------
-----------------------------------TPATVFVQILRLIKPMKDGTRYTGI
RSLKLDMNPGTGIWQSIDVKTVLQNWLKQPESNLGIEIKALDE--------------NGH
DLAVTFPEPGEDGLTPFLEVKVT-----DTPKRSRRDF-GLDCDEHSTESRCCRYPLTVD
FEAFGWDWIIAPKRYKANYCSGECEFVFLQKYPHTHLVHQANPRGSAGPCCTPTKMSPIN
MLYFNGEGQIIYGKIPAMVVDRCGCS
```

```
>CrystalStructure.5ntu
------------------------------------------------------------
------------------------------------------------------------
------------------------------------------------------------
---------------QNTKSSRIEAIKIQILSKLRLETAPNISKDVIRQLLPKAPPLREL
IDQYD-----------------------------------------------EDDDYHAT
TETIITMPTE-----------KCCFFKFSSKIQYNKVVKAQLWIYLRPVE----------
-----------------------------------TPTTVFVQILRLIKPMKDGTRYTGI
RSLKLDMNPGTGIWQSIDVKTVLQNWLAAPASNLGIEIKALDE--------------NGH
DLAVTFPGPGEDGLNPFLEVKVT-----DTPKRSRRDF-GLDCDEHSTESRCCRYPLTVD
FEAFGWDWIIAPKRYKANYCSGECEFVFLAAYPHTHLVHQANPRGSAGPCCTPTKMSPIN
MLYFNGKEQIIYGKIPAMVVDRCGCS
>Sus.scrofa.NP_999600
---------------------------------------------------MQKLQIYVY
IYLF-----------MLIVAGPVDLNEN--------------------------------
--------------------------------------------------------SEQK
ENVEKEGLCNACMWRQNTKSSRLEAIKIQILSKLRLETAPNISKDAIRQLLPKAPPLREL
IDQYDV-------QRDDS-----------------------------SDGSLEDDDYHAT
TETIITMPTESDLLMQVEGKPKCCFFKFSSKIQYNKVVKAQLWIYLRPVK----------
-----------------------------------TPTTVFVQILRLIKPMKDGTRYTGI
RSLKLDMNPGTGIWQSIDVKTVLQNWLKQPESNLGIEIKALDE--------------NGH
DLAVTFPGPGEDGLNPFLEVKVT-----DTPKRSRRDF-GLDCDEHSTESRCCRYPLTVD
FEAFGWDWIIAPKRYKASYCSGECEFVFLQKYPHTHLVHQANPRGSAGPCCTPTKMSPIN
MLYFNGKEQIIYGKIPAMVVDRCGCS
>Physeter.catodon.XP_007104504
---------------------------------------------------MQKLQIYVY
IYLF-----------MLMVAGPVDLNEN--------------------------------
--------------------------------------------------------SEQK
ENVEKEGLCNACMWRQNTKSSRLEAIKIQILSKLRLETAPNISKDAIRQLLPKAPPLREL
IDQYDV-------QRDDS-----------------------------SDGSLEDDDYHAT
TETVITMPTESDLLMQVEGKPKCCFFKFSSKIQYNKVVKAQLWIYLRPVK----------
-----------------------------------TPTTVFVQILRLIKPMKDGTRYTGI
RSLKLDMNPGTGIWQSIDVKTVLQNWLKQPESNLGIEIKALDE--------------NGH
DLAVTFPGPGEDGLNPFLEVKVT-----DTPKRSRRDF-GLDCDEHSTESRCCRYPLTVD
FEAFGWDWIIAPKRYKANYCSGECEFVFLQKYPHTHLVHQANPRGSAGPCCTPTKMSPIN
MLYFNGKEQIIYGKIPAMVVDRCGCS
>Balaenoptera.acutorostrata.XP_007171534
---------------------------------------------------MQKLQIYVY
IYLF-----------MLIVAGPVDLNEN--------------------------------
--------------------------------------------------------SEQK
ENVEKEGLCNACMWRQNTKSSRLEAIKIQILSKLRLETAPNISKDAIRQLLPKAPPLREL
IDQYDV-------QRDDS-----------------------------SDGSLEDDDYHAT
TETVITMPTESDLLTQVEGKPKCCFFKFSSKIQYNKVVKAQLWIYLRPVK----------
-----------------------------------TPTTVFVQILRLIKPMKDGTRYTGI
RSLKLDMNPGTGIWQSIDVKTVLQNWLKQPESNLGIEIKALDE--------------NGH
DLAVTFPGPGEDGLNPFLEVKVT-----DTPKRSRRDF-GLDCDEHSTESRCCRYPLTVD
FEAFGWDWIIAPKRYKANYCSGECEFVFLQKYPHTHLVHQANPRGSAGPCCTPTKMSPIN
MLYFNGKEQIIYGKIPAMVVDRCGCS
>Felis.catus.XP_003991021
---------------------------------------------------MQKLQIYVY
IYLF-----------MLIVAGPVDLNEN--------------------------------
--------------------------------------------------------SEQK
ENVEKEGLCNACTWRQNTKSSRIEAIKIQILSKLRLETAPNISKDAIRQLLPKAPPLREL
IDQYDV-------QRDDS-----------------------------SDGSLEDDDYHAT
TETIITMPTESDLLMQVEGKPKCCFFKFSSKIQYNKVVKAQLWIYLRPVK----------
-----------------------------------TPTTVFVQILRLIKPMKDGTRYTGI
RSLKLDMNPGTGIWQSIDVKTVLQNWLKQPESNLGIEIKALDE--------------NGH
DLAVTFPGPGEDGLNPFLEVKVT-----DTPKRSRRDF-GLDCDEHSTESRCCRYPLTVD
FEAFGWDWIIAPKRYKANYCSGECEFVFLQKYPHTHLVHQANPRGSAGPCCTPTKMSPIN
MLYFNGKEQIIYGKIPAMVVDRCGCS
```

```
>Panthera.pardus.XP_019317683
---------------------------------------------------MQKLQIYVY
IYLF-----------MLIVAGPVDLNEN--------------------------------
--------------------------------------------------------SEQK
ENVEKEGLCNACTWRQNTKSSRIEAIKIQILSKLRLETAPNISKDAIRQLLPKAPPLREL
IDQYDV-------QRDDS-----------------------------SDGSLEDDDYHAT
TETIITMPTESDLLMQAEGKPKCCFFKFSSKIQYNKVVKAQLWIYLRPVK----------
-----------------------------------TPTTVFVQILRLIKPMKDGTRYTGI
RSLKLDMNPGTGIWQSIDVKTVLQNWLKQPESNLGIEIKALDE--------------NGH
DLAVTFPGPGEDGLNPFLEVKVT-----DTPKRSRRDF-GLDCDEHSTESRCCRYPLTVD
FEAFGWDWIIAPKRYKANYCSGECEFVFLQKYPHTHLVHQANPRGSAGPCCTPTKMSPIN
MLYFNGKEQIIYGKIPAMVVDRCGCS
>Miniopterus.natalensis.XP_016070995
---------------------------------------------------MQKLQIYVY
IYLF-----------MLIVAGPVDLNEN--------------------------------
--------------------------------------------------------SEQK
ENVEKEGLCNACTWRQNTKSSRIEAIKIQILSKLRLETAPNISKDAIRQLLPKAPPLREL
IDQYDV-------QRDDS-----------------------------SDGSLEDDDYHAT
TETIITMPTESDLLMQVEGKPKCCFFKFSSKIQFNKVVKAQLWIYLRPVK----------
-----------------------------------TPTTVFVQILRLIKPMKDGTRYTGI
RSLKLDMNPGTGIWQSIDVKTVLQNWLKQPESNLGIEIKALDE--------------NGH
DLAVTFPGPGEDGLNPFLEVKVT-----DTPKRSRRDF-GLDCDEHSTESRCCRYPLTVD
FEAFGWDWIIAPKRYKANYCSGECEFVFLQKYPHTHLVHQANPRGSAGPCCTPTKMSPIN
MLYFNGKEQIIYGKIPAMVVDRCGCS
>Orcinus.orca.XP_004276982
---------------------------------------------------MQKLQIYVC
IYLF-----------MLIVAGPVDLNEN--------------------------------
--------------------------------------------------------SEQK
ENVEKEGLCNACMWRQNTKSSRLEAIKIQILSKLRLETAPNISKDAIRQLLPKAPPLREL
IDQYDV-------QRDDS-----------------------------SDGSLEDDDYHAT
TETVITMPTESDLLMQVEGKPKCCFFKFSSKIQYNKVVKAQLWIYLRPVK----------
-----------------------------------TPTTVFVQILRLIKPMKDGTRYTGI
RSLKLDMNPGTGIWQSIDVKTVLQNWLKQPESNLGIEIKALDE--------------NGH
DLAVTFPGPGEDGLNPFLEVKVT-----DTPKRSRRDF-GLDCDEHSTESRCCRYPLTVD
FEAFGWDWIIAPKRYKANYCSGECEFVFLQKYPHTHLVHQANPRGSAGPCCTPTKMSPIN
MLYFNGKEQIIYGKIPAMVVDRCGCS
>Desmodus.rotundus.XP_024420048
---------------------------------------------------MQKLQIYAY
IYLF-----------MLIVAGPVDLNEN--------------------------------
--------------------------------------------------------SEQK
ENVEKEGLCNACTWRQNTKSSRIEAIKIQILSKLRLETAPNISKDAIRQLLPKAPPLREL
IDQYDV-------QRDDS-----------------------------SDGSLEDDDYHAT
TETIITMPTESDLLMQVEGKPKCCFFKFSSKIQFNKVVKAQLWIYLRPVQ----------
-----------------------------------TPTTVFVQILRLIKPMKDGTRYTGI
RSLKLDMNPGTGIWQSIDVKTVLQNWLKQPESNLGIEIKALDE--------------NGH
DLAVTFPGPGEDGLNPFLEIKVT-----DTPKRSRRDF-GLDCDEHSTESRCCRYPLTVD
FEAFGWDWIIAPKRYKANYCSGECEFVFLQKYPHTHLVHQANPRGSAGPCCTPTKMSPIN
MLYFNGKEQIIYGKIPAMVVDRCGCS
>Callorhinus.ursinus.XP_025743187
---------------------------------------------------MQKLQIYVY
IYLF-----------MLIVAGPVDLNEN--------------------------------
--------------------------------------------------------SEQK
ENVEKEGLCNACTWRQNTKSSRIEAIKIQILSKLRLETAPNISKDAIRQLLPKAPPLREL
IDQYDV-------QRDDS-----------------------------SDGSLEDDDYHAT
TETIITMPTESDLLTQVEGKPKCCFFKFSSKIQYNKVVKAQLWIYLRPVK----------
-----------------------------------TPTTVFVQILRLIKPMKDGTRYTGI
RSLKLDMNPGTGIWQSIDVKTVLQNWLKQPESNLGIEIKALDE--------------NGH
DLAVTFPGPGEDGLNPFLEVKVT-----DTPKRSRRDF-GLDCDEHSTESRCCRYPLTVD
FEAFGWDWIIAPKRYKANYCSGECEFVFLQKYPHTHLVHQANPRGSAGPCCTPTKMSPIN
MLYFNGKEQIIYGKIPAMVVDRCGCS
```

>Rousettus.aegyptiacus.XP_016002878
---------------------------------------------------MQKLQIYVY
IYLF-----------MLIVAGPVNLNEN--------------------------------
--------------------------------------------------------SEQK
ENVEKEGLCNACTWRQNTKSSRIEAIKIQILSKLRLETAPNISKDAIRQLLPKAPPLREL
IDQYDV-------QRDDS-----------------------------SDGSLEDDDYHAT
TETIITMPSESDLLMQVEGKPKCCFFKFSSKIQYNKVVKAQLWIYLRPVK----------
-----------------------------------TPTTVFVQILRLIKPMKDGTRYTGI
RSLKLDMNPGTGIWQSIDVKTVLQNWLKQPESNLGIEIKALDE--------------NGH
DLAVTFPGPGEDGLNPFLEVKVT-----DTPKRSRRDF-GLDCDEHSTESRCCRYPLTVD
FEAFGWDWIIAPKRYKANYCSGECEFVFLQKYPHTHLVHQANPRGSAGPCCTPTKMSPIN
MLYFNGKEQIIYGKIPAMVVDRCGCS
>Microcebus.murinus.XP_012632327
---------------------------------------------------MQKLQIYVY
IYLF-----------MLIVAGPVDLNEN--------------------------------
--------------------------------------------------------SEQK
ENVEKEGLCNACTWRQNTKSSRIEAIKIQILSKLRLETAPNISKDAIRQLLPKAPPLREL
IDQYDV-------QRDDS-----------------------------SDGSLEDDDYHAT
TETIITMPTESDLLMQVDGKPKCCFFKFSSKVQYNKVVKAQLWIYLRPVK----------
-----------------------------------TPTTVFVQILRLIKPMKDGTRYTGI
RSLKLDMNPGTGIWQSIDVKTVLQNWLKQPESNLGIEIKALDE--------------NGH
DLAVTYPGPGEDGLNPFLEVKVT-----DTPKRSRRDF-GLDCDEHSTESRCCRYPLTVD
FEAFGWDWIIAPKRYKANYCSGECEFVFLQKYPHTHLVHQANPRGSAGPCCTPTKMSPIN
MLYFNGKEQIIYGKIPAMVVDRCGCS
>Condylura.cristata.XP_004674495
---------------------------------------------------MQKLQIYVY
IYLF-----------MLIVAGPVNLNEN--------------------------------
--------------------------------------------------------SEQK
ENVEKEGLCNACTWRQNTKSSRIEAIKIQILSKLRLETAPNISKDAIRQLLPKAPPLREL
IDQYDV-------QRDDS-----------------------------SDGSLEDDDYHAT
TETIITMPTESDLLMQVEGKPKCCFFKFSSKIQYNKVVKAQLWIYLRPVK----------
-----------------------------------SPTTVFVQILRLIKPMKDGTRYTGI
RSLKLDMNPGTGIWQSIDVKTVLQNWLKQPESNLGIEIKALDE--------------NGH
DLAVTFPGPGEDGLNPFLEVKVT-----DTPKRSRRDF-GLDCDEHSTESRCCRYPLTVD
FEAFGWDWIIAPKRYKANYCSGECEFVFLQKYPHTHLVHQANPRGSAGPCCTPTKMSPIN
MLYFNGKEQIIYGKIPAMVVDRCGCS
>Phyllostomus.discolor.XP_028365503
---------------------------------------------------MQKLQIYVY
IYLF-----------MLIVAGPVDLNEN--------------------------------
--------------------------------------------------------SEQK
ENVEKEGLCNACTWRQNTKSSRIEAIKIQILSKLRLETAPNISKDAIRQLLPKAPPLREL
IDQYDV-------QRDDS-----------------------------SDGSLEDDDYHAT
TETIITMPTESDLLVQVEGKPKCCFFKFSSKIQFNKVVKAQLWIYLRPVE----------
-----------------------------------TPTTVFVQILRLIKPMKDGTRYTGI
RSLKLDMNPGTGIWQSIDVKTVLQNWLKQPESNLGIEIKALDE--------------NGH
DLAVTFPGPGEDGLNPFLEIKVT-----DTPKRSRRDF-GLDCDEHSTESRCCRYPLTVD
FEAFGWDWIIAPKRYKANYCSGECEFVFLQKYPHTHLVHQANPRGSAGPCCTPTKMSPIN
MLYFNGKEQIIYGKIPAMVVDRCGCS
>Ceratotherium.simum.XP_004426790
---------------------------------------------------MQKLQIYVY
IYLF-----------MLIVAGPVDLNEN--------------------------------
--------------------------------------------------------SEQK
ENVEKEGLCNACTWRQNTKSSRIEAIKIQILSKLRLETAPNISKDAIRQLLPKAPPLREL
IDQYDV-------QRDDS-----------------------------SDGSLEDDDYHAT
TETIITMPTESDLLMQVEGKPKCCFFKFSSKIQYNKVVKAQLWIYLRPVK----------
-----------------------------------TPTTVFVQILRLIKPMKDGTRYTGI
RSLKLDMNPGTGIWQSIDVKTVLQNWLKQPESNLGIEIKALDE--------------NGH
DLAVTFPRPGEDGLNPFLEVKVT-----DTPKRSRRDF-GLDCDEHSTESRCCRYPLTVD
FEAFGWDWIIAPKRYKANYCSGECEFVFLQKYPHTHLVHQANPRGSAGPCCTPTKMSPIN
MLYFNGKEQIIYGKIPAMVVDRCGCS

>Hipposideros.armiger.XP_019482023
---------------------------------------------------MQKLQIYVS
IYLF-----------MLIVAGPVDLNEN--------------------------------
--------------------------------------------------------SEQK
ENVEKEGLCNACTWRQNTKSSRIEAIKIQILSKLRLETAPNISKDAIRQLLPKAPPLREL
IDQYDV-------QRDDS-----------------------------SDGSLEDDDYHAT
TETIITMPTESDLLMQVEGKPKCCFFKFSSKIQYNKVVKAQLWIYLRPVN----------
-----------------------------------TPTTVFVQILRLIKPMKDGTRYTGI
RSLKLDMNPGTGIWQSIDVKTVLQNWLKQPESNLGIEIKALDE--------------NGH
DLAVTFPGPGEDGLNPFLEVKVT-----DTPKRSRRDF-GLDCDEHSTESRCCRYPLTVD
FEAFGWDWIIAPKRYKANYCSGECEFVFLQKYPHTHLVHQANPRGSAGPCCTPTKMSPIN
MLYFNGKEQIIYGKIPAMVVDRCGCS
>Neomonachus.schauinslandi.XP_021558792
---------------------------------------------------MQKLQIYVY
IYLF-----------MLIVAGPVDLNEN--------------------------------
--------------------------------------------------------SEQK
ENVEKEGLCNACTWRQNTKSSRIEAIKIQILSKLRLETAPNISKDAIRQLLPKAPPLREL
IDQYDV-------QRDDS-----------------------------SDGSLEDDDYHAT
TETIITMPTESDLLTQVEGNPKCCFFKFSSKIQYNKVVKAQLWIYLRPVK----------
-----------------------------------TPTTVFVQILRLIKPMKDGTRYTGI
RSLKLDMNPGTGIWQSIDVKTVLQNWLKQPESNLGIEIKALDE--------------NGH
DLAVTFPGPGEDGLNPFLEVKVT-----DTPKRSRRDF-GLDCDEHSTESRCCRYPLTVD
FEAFGWDWIIAPKRYKANYCSGECEFVFLQKYPHTHLVHQANPRGSAGPCCTPTKMSPIN
MLYFNGKEQIIYGKIPAMVVDRCGCS
>Ailuropoda.melanoleuca.XP_002918707
---------------------------------------------------MQKLQIYVY
IYLF-----------MLIVAGPVDLNEN--------------------------------
--------------------------------------------------------SEQK
ENVEKEGLCNACTWRQNTKSSRIEAIKIQILSKLRLETAPNISKDAIRQLLPKAPPLREL
IDQYDV-------QRDDS-----------------------------SDGSLEDDDYHVT
TETIITMPSESDLLMQVEGKPKCCFFKFSSKIQYNKVVKAQLWIYLRPVK----------
-----------------------------------TPTTVFVQILRLIKPMKDGTRYTGI
RSLKLDMNPGTGIWQSIDVKTVLQNWLKQPESNLGIEIKALDE--------------NGH
DLAVTFPGPGEDGLNPFLEVKVT-----DTPKRSRRDF-GLDCDEHSAESRCCRYPLTVD
FEAFGWDWIIAPKRYKANYCSGECEFVFLQKYPHTHLVHQANPRGSAGPCCTPTKMSPIN
MLYFNGKEQIIYGKIPAMVVDRCGCS
>Odobenus.rosmarus.XP_004392460
---------------------------------------------------MQKLQIYVY
IYLF-----------MLIVAGPVDLNEN--------------------------------
--------------------------------------------------------SEQK
ENVEKEGLCNACTWRQNTKSSRIEAIKIQILSKLRLETAPNISKDAIRQLLPKAPPLREL
IDQYDV-------QRDDS-----------------------------SDGSLEDDDYHAT
TETIITMPTESDLLTQVEGKPKCCFFKFSSKIQYNKVVKAQLWIYLRPVK----------
-----------------------------------TPTTVFVQILRLIKPMKDGTRYTGI
RSLKLDMNPGAGIWQSIDVKTVLQNWLKQPESNLGIEIKALDE--------------NGH
DLAVTFPGPGEDGLNPFLEVKVT-----DTPKRSRRDF-GLDCDEHSTESRCCRYPLTVD
FEAFGWDWIIAPKRYKANYCSGECEFVFLQKYPHTHLVHQANPRGSAGPCCTPTKMSPIN
MLYFNGKEQIIYGKIPAMVVDRCGCS
>Balearica.regulorum.XP_010309399
---------------------------------------------------MQKLAIYVY
IYLF-----------MLISVDPVALDDS--------------------------------
--------------------------------------------------------SQPT
ENAEKDGLCNACTWRQNTKSSRIEAIKIQILSKLRLEQAPNISRDVIKQLLPKAPPLQEL
IDQYDV-------QRDDS-----------------------------SDGSLEDDDYHAT
TETIITMPTESDLLVQMEGKPKCCFFKFSSKIQYNKVVKAQLWIYLRQVQ----------
-----------------------------------KPTTVFVQILRLIKPMKDGTRYTGI
RSLKLDMNPGTGIWQSIDVKTVLQNWLKQPESNLGIEIKAFDE--------------NGQ
DLAVTFPGPGEDGLNPFLEVRVT-----DTPKRSRRDF-GLDCDEHSTESRCCRYPLTVD
FEAFGWDWIIAPKRYKANYCSGECEFVFLQKYPHTHLVHQANPRGSAGPCCTPTKMSPIN
MLYFNGKEQIIYGKIPAMVVDRCGCS

```
>Nipponia.nippon.XP_009465237
---------------------------------------------------MQKLAIYVY
IYLF-----------MLISVDPVALDDS--------------------------------
--------------------------------------------------------SQPT
ENAEKDGLCNACTWRQNTKSSRIEAIKIQILSKLRLEQAPNISRDVIKQLLPKAPPLQEL
IDQYDV-------QRDDS-----------------------------SDGSLEDDDYHAT
TETIITMPTESDFLVQMEGKPKCCFFKFSSKIQYNKVVKAQLWIYLRQVQ----------
-----------------------------------KPTTVFVQILRLIKPMKDGTRYTGI
RSLKLDMNPGTGIWQSIDVKTVLQNWLKQPESNLGIEIKAFDE--------------NGR
DLAVTFPGPGEDGLNPFLEVRVT-----DTPKRSRRDF-GLDCDEHSTESRCCRYPLTVD
FEAFGWDWIIAPKRYKANYCSGECEFVFLQKYPHTHLVHQANPRGSAGPCCTPTKMSPIN
MLYFNGKEQIIYGKIPAMVVDRCGCS
>Calypte.anna.XP_008495173
---------------------------------------------------MQKLAVYVY
IYLF-----------MLISVDPVALGDS--------------------------------
--------------------------------------------------------SQPT
ENAEKDGLCNACTWRQNTKSSRIEAIKIQILSKLRLEHAPNISRDVIRQLLPKAPPLQEL
IDQYDV-------QRDDS-----------------------------SDGSLEDDDYHAT
TETIITMPTESDFLVQMEGKPKCCFFKFSSKIQYNKVVKAQLWIYLRQVQ----------
-----------------------------------KPTTVFVQILRLIKPMKDGTRYTGI
RSLKLDMNPGTGIWQSIDVKTVLQNWLKQPESNLGIEIKAFDE--------------NGR
DLAVTFPGPGEDGLNPFLEVRVT-----DTPKRSRRDF-GLDCDEHSTESRCCRYPLTVD
FEAFGWDWIIAPKRYKANYCSGECEFVFLQKYPHTHLVHQANPRGSAGPCCTPTKMSPIN
MLYFNGKEQIIYGKIPAMVVDRCGCS
>Gavia.stellata.XP_009808681
---------------------------------------------------MQKLVIYVY
IYLF-----------MLISVDPVALDDS--------------------------------
--------------------------------------------------------SQPT
ENAEKDGLCNACTWRQNTKSSRIEAIKIQILSKLRLEQAPNISRDVIKQLLPKAPPLQEL
IDQYDV-------QRDDS-----------------------------SDGSLEDDDYHAT
TETIITMPTESDFLVQMEGKPKCCFFKFSSKIQYNKVVKAQLWIYLRQVQ----------
-----------------------------------KPTTVFVQILRLIKPMKDGTRYTGI
RSLKLDMNPGTGIWQSIDVKTVLQNWLKQPESNLGIEIKAFDE--------------NGR
DLAVTFPGPGEDGLNPFLEVRVT-----DTPKRSRRDF-GLDCDEHSTESRCCRYPLTVD
FEAFGWDWIIAPKRYKANYCSGECEFVFLQKYPHTHLVHQANPRGSAGPCCTPTKMSPIN
MLYFNGKEQIIYGKIPAMVVDRCGCS
>Cariama.cristata.XP_009701031
---------------------------------------------------MQKLAVYVY
IYLF-----------MLISVDPVALDDS--------------------------------
--------------------------------------------------------SQPT
ENAEKDGLCNACTWRQNTKSSRIEAIKIQILSKLRLEQAPNISRDVIKQLLPKAPPLQEL
IDQYDV-------QRDDS-----------------------------SDGSLEDDDYHAT
TETIITMPTESDFLVQMEGKPKCCFFKFSSKIQYNKVVKAQLWIYLRQVQ----------
-----------------------------------KPTTVFVQILRLIKPMKDGTRYTGI
RSLKLDMNPGTGIWQSIDVKTVLQNWLKQPESNLGIEIKAFDE--------------NGR
DLAVTFPGPGEDGLNPFLEVRVT-----DTPKRSRRDF-GLDCDEHSTESRCCRYPLTVD
FEAFGWDWIIAPKRYKANYCSGECEFVFLQKYPHTHLVHQANPRGSAGPCCTPTKMSPIN
MLYFNGKEQIIYGKIPAMVVDRCGCS
>Dromaius.novaehollandiae.XP_025953574
---------------------------------------------------MQKVAIYVY
IYLF-----------MLISVDPVALDDS--------------------------------
--------------------------------------------------------SQPT
ENAEKDGLCNACTWRQNTKSSRIEAIKIQILSKLRLEQAPNISRDVIKQLLPKAPPLQEL
IDQYDV-------QRDDS-----------------------------SDGSLEDDDYHAT
TETIITMPTESDFLVQMEGKPKCCFFKFSSKIQYNKVVKAQLWIYLRQVQ----------
-----------------------------------KPTTVFVQILRLIKPMKDGTRYTGI
RSLKLDMNPGTGIWQSIDVKTVLQNWLKQPESNLGIEIKAFDE--------------NGQ
DLAVTFPGPGEDGLNPFLEVRVT-----DTPKRSRRDF-GLDCDEHSTESRCCRYPLTVD
FEAFGWDWIIAPKRYKANYCSGECEFVFLQKYPHTHLVHQANPRGSAGPCCTPTKMSPIN
MLYFNGKEQIIYGKIPAMVVDRCGCS
```

>Fulmarus.glacialis.XP_009576651
---------------------------------------------------MQKLAIYVY
IYLF-----------MLISVDPVALDDG--------------------------------
--------------------------------------------------------SQPA
ENAEKDGLCNACTWRQNTKSSRIEAIKIQILSKLRLEQAPNISRDVIKQLLPKAPPLQEL
IDQYDV-------QRDDS-----------------------------SDGSLEDDDYHAT
TETIITMPTESDFLVQMEGKPKCCFFKFSSKIQYNKVVKAQLWIYLRQVQ----------
-----------------------------------KPTTVFVQILRLIKPMKDGTRYTGI
RSLKLDMNPGTGIWQSIDVKTVLQNWLKQPESNLGIEIKAFDE--------------NGR
DLAVTFPGPGEDGLNPFLEVRVT-----DTPKRSRRDF-GLDCDEHSTESRCCRYPLTVD
FEAFGWDWIIAPKRYKANYCSGECEFVFLQKYPHTHLVHQANPRGSAGPCCTPTKMSPIN
MLYFNGKEQIIYGKIPAMVVDRCGCS
>Anas.platyrhynchos.XP_005011469
---------------------------------------------------MQKLAIYVY
IYLF-----------MLISVDPVALDDG--------------------------------
--------------------------------------------------------SQPT
ENAEKDGLCNACTWRQNTKSSRIEAIKIQILSKLRLEQAPNISRDVIKQLLPKAPPLQEL
IDQYDV-------QRDDS-----------------------------SDGSLEDDDYHAT
TETIITMPTESDFLVQMEGKPKCCFFKFSSKIQYNKVVKAQLWIYLRQVQ----------
-----------------------------------KPTTVFVQILRLIKPMKDGTRYTGI
RSLKLDMNPGTGIWQSIDVKTVLQNWLKQPESNLGIEIKAFDE--------------NGR
DLAVTFPGPGEDGLNPFLEVRVT-----DTPKRSRRDF-GLDCDEHSTESRCCRYPLTVD
FEAFGWDWIIAPKRYKANYCSGECEFVFLQKYPHTHLVHQANPRGSAGPCCTPTKMSPIN
MLYFNGKEQIIYGKIPAMVVDRCGCS
>Apteryx.australis.XP_013807627
---------------------------------------------------MQKLAIYVY
IYLF-----------MLISVDPVALDDS--------------------------------
--------------------------------------------------------SQPT
ENAEKDGLCNACTWRQNTKSSRIEAIKIQILSKLRLEQAPNISRDVIKQLLPKAPPLQEL
IDQYDV-------QRDDS-----------------------------SDGSLEDDDYHAT
TETIITMPTESDFLAQMEGKPKCCFFKFGSKIQYNKVVKAQLWIYLRQVQ----------
-----------------------------------KPTTVFVQILRLIKPMKDGTRYTGI
RSLKLDMNPGTGIWQSIDVKTVLQNWLKQPESNLGIEIKAFDE--------------NGQ
DLAVTFPGPGEDGLNPFLEVRVT-----DTPKRSRRDF-GLDCDEHSTESRCCRYPLTVD
FEAFGWDWIIAPKRYKANYCSGECEFVFLQKYPHTHLVHQANPRGSAGPCCTPTKMSPIN
MLYFNGKEQIIYGKIPAMVVDRCGCS
>Picoides.pubescens.XP_009909511
---------------------------------------------------MQKLAIYVY
LYLF-----------MLISVDPVALDEG--------------------------------
--------------------------------------------------------NQPT
ENADKDGLCNACTWRQNTKSSRIEAIKIQILSKLRLEQAPNISRDVIKQLLPKAPPLQEL
IDQYDV-------QRDDS-----------------------------SDGSLEDDDYHAT
TETIITMPTESDFLVQMEGKPKCCFFKFSSKIQYNKVVKAQLWIYLRQVQ----------
-----------------------------------KPTTVFVQILRLIKPMKDGTRYTGI
RSLKLDMSPGTGIWQSIDVKTVLQNWLKQPESNLGIEIKAFDE--------------NGR
DLAVTFPGPGEDGLNPFLEVRVT-----DTPKRSRRDF-GLDCDEHSTESRCCRYPLTVD
FEAFGWDWIIAPKRYKANYCSGECEFVFLQKYPHTHLVHQANPRGSAGPCCTPTKMSPIN
MLYFNGKEQIIYGKIPAMVVDRCGCS
>Haliaeetus.albicilla.XP_009915599
---------------------------------------------------MQKLAISVY
IYLF-----------MLISVDPVALDDS--------------------------------
--------------------------------------------------------SQPT
ENAEKDGLCNACTWRQNTKSSRIEAIKIQILSKLRLEQAPNISRDVIKQLLPKAPPLQEL
IDQYDV-------QRDDS-----------------------------SDGSLEDDDYHAT
TETIITMPTESDFLVQVEGKPKCCFFKFSSKIQYNKVVKAQLWIYLRQVQ----------
-----------------------------------KPTTVFVQILRLIKPMKDGTRYTGI
RSLKLDMNPGTGIWQSIDVKTVMQNWLKQPESNLGIEIKAFDE--------------NGQ
DLAVTFPGPGEDGLNPFLEVRVT-----DTPKRSRRDF-GLDCDEHSTESRCCRYPLTVD
FEAFGWDWIIAPKRYKANYCSGECEFVFLQKYPHTHLVHQANPRGSAGPCCTPTKMSPIN
MLYFNGKEQIIYGKIPAMVVDRCGCS

```
>Phaethon.lepturus.XP_010290320
----------------------------------------------------MQKLAIWVY
IYLF-----------MLISVDPVALDDS--------------------------------
---------------------------------------------------------SQPA
ENAEKDGLCNACTWRQNTKSSRIEAIKIQILSKLRLEQAPNISRDVIKQLLPKAPPLQEL
IDQYDV-------QRDDS-----------------------------SDGSLEDDDYHAT
TETIITMPTESDFLVQMEGKPKCCFFKFSSKIQYNKVVKAQLWIYLRQVQ----------
------------------------------------KPTTVFVQILRLIKPMKDGTRYTGI
RSLKLDMNPGTGIWQSIDVKTVLQNWLKQPESNLGIEIKAFDE--------------NGQ
DLAVTFPGPGEDGLNPFLEVRVT-----DTPKRSRRDF-GLDCDEHSTESRCCRYPLTVD
FEAFGWDWIIAPKRYKANYCSGECEFVFLQKYPHTHLVHQANPRGSAGPCCTPTKMSPIN
MLYFNGKEQIIYGKIPAMVVDRCGCS
>Colius.striatus.XP_010203312
----------------------------------------------------MQKLATYVY
IYLF-----------MLISVDPLALDDS--------------------------------
---------------------------------------------------------SQPT
ENAEKDGLCNACTWRQNTKSSRIEAIKIQILSKLRLEQAPNISRDVIKQLLPKAPPLQEL
IDQYDV-------QRDDS-----------------------------SDGSLEDDDYHAT
TETIITMPTESDFLVQMEGKPKCCFFKFSSKIQYNKVVKAQLWIYLRQVQ----------
------------------------------------KPTTVFVQILRLIKPMKDGTRYTGI
RSLKLDMNPGTGIWQSIDVKTVLQNWLKQPESNLGIEIKAFDE--------------NGR
DLAVTFPGPGEDGLNPFLEVRVT-----DTPKRSRRDF-GLDCDEHSTESRCCRYPLTVD
FEAFGWDWIIAPKRYKANYCSGECEFVFLQKYPHTHLVHQANPRGSAGPCCTPTKMSPIN
MLYFNGKEQIIYGKIPAMVVDRCGCS
>Aptenodytes.forsteri.XP_009275391
----------------------------------------------------MQKLAIYVY
IYLF-----------MLISVDPVALDDG--------------------------------
---------------------------------------------------------SHPT
ENAEKDGLCNACTWRQNTKSSRIEAIKIQILSKLRLEQAPNISRDVIKQLLPKAPPLQEL
IDQYDV-------QRDDS-----------------------------SDGSLEDDDYHAT
TETIITMPTESDFLVQMEGKPKCCFFKFSSKIQYNKVVKAQLWIYLRQVQ----------
------------------------------------KPTTVFVQILRLIKPMKDGTRYTGI
RSLKLDMNPGTGIWQSIDVKTVLQNWLKQPESNLGIEIKAFDE--------------NGR
DLAVTFPGPGEDGLNPFLEVRVT-----DTPKRSRRDF-GLDCDEHSTESRCCRYPLTVD
FEAFGWDWIIAPKRYKANYCSGECEFVFLQKYPHTHLVHQANPRGSAGPCCTPTKMSPIN
MLYFNGKEQIIYGKIPAMVVDRCGCS
>Phalacrocorax.carbo.XP_009505846
----------------------------------------------------MQKLAIYVY
IYLF-----------MLISVDLVALDDG--------------------------------
---------------------------------------------------------SQPT
ENAEKDGLCNACTWRQNTKSSRIEAIKIQILSKLRLEQAPNISRDVIKQLLPKAPPLQEL
IDQYDV-------QRDDS-----------------------------SDGSLEDDDYHAT
TETIITMPTESDLLVQMEGKPKCCFFKFSSKIQYNKVVKAQLWIYLRQVQ----------
------------------------------------KPTTVFVQILRLIKPMKDGTRYTGI
RSLKLDMNPGTGIWQSIDVKTVLQNWLKQPESNLGIEIKAFDE--------------NGR
DLAVTFPGPGEDGLNPFLEVRVT-----DTPKRSRRDF-GLDCDEHSTESRCCRYPLTVD
FEAFGWDWIIAPKRYKANYCSGECEFVFLQKYPHTHLVHQANPRGSAGPCCTPTKMSPIN
MLYFNGKEQIIYGKIPAMVVDRCGCS
>Acanthisitta.chloris.XP_009075628
----------------------------------------------------MQKLALYVY
IYLF-----------MLISVDPVALDDS--------------------------------
---------------------------------------------------------NQPT
ENAEKDGLCNACTWRQNTKSSRIEAIKIQILSKLRLEQAPNISRDVIKQLLPKAPPLQEL
IDQYDV-------QRDDS-----------------------------SDGSLEDDDYHAT
TETIITMPTESDFLVQMEGKPKCCFFKFSSKIQYNKVVKAQLWIYLRQVQ----------
------------------------------------KPTTVFVQILRLITPMKDGTRYTGI
RSLKLDMNPGTGIWQSIDVKTVLQNWLKQPESNLGIEIKAFDE--------------NGR
NLAVTFPGPGEDGLNPFLEVRVT-----DTPKRSRRDF-GLDCDEHSTESRCCRYPLTVD
FEAFGWDWIIAPKRYKANYCSGECEFVFLQKYPHTHLVHQANPRGSAGPCCTPTKMSPIN
MLYFNGKEQIIYGKIPAMVVDRCGCS
```

>Merops.nubicus.XP_008933453
---------------------------------------------------MQKPVIYVY
IYLF-----------MLISIDPVALDDS--------------------------------
--------------------------------------------------------NQPA
ENAEKDGLCNACTWRQNTKSSRIEAIKIQILSKLRLEQAPNISRDVIKQLLPKAPPLQEL
IDQYDV-------QRDDS-----------------------------SDGSLEDDDYHAT
TETIITMPTESDFLVQMEGKPKCCFFKFSSKIQYNKVVKAQLWIYLRQVQ----------
-----------------------------------KPTTVFVQILRLIKPMKDGTRYTGI
RSLKLDMNPGTGIWQSIDVKTVLQNWLKQPESNLGIEIKAFDE--------------NGR
DLAVTFPGPGEDGLNPFLEVRVT-----DTPKRSRRDF-GLDCDEHSTESRCCRYPLTVD
FEAFGWDWIIAPKRYKANYCSGECEFVFLQKYPHTHLVHQANPRGSAGPCCTPTKMSPIN
MLYFNGKEQIIYGKIPAMVVDRCGCS
>Eurypyga.helias.XP_010150584
---------------------------------------------------MQKLAVYVY
IYLF-----------MLISVDPVALEDG--------------------------------
--------------------------------------------------------SQPA
ENGEKDGLCNACAWRQNTKSSRIEAIKIQILSKLRLEQAPNISRDVIKQLLPKAPPLQEL
IDQYDV-------QRDDS-----------------------------SDGSLEDDDYHAT
TETIITMPTESDFLVQMEGKPKCCFFKFSSKIQYNKVVKAQLWIYLRQVQ----------
-----------------------------------KPTTVFVQILRLIKPMKDGTRYTGI
RSLKLDMNPGTGIWQSIDVKTVLQNWLKQPESNLGIEIKAFDE--------------NGR
DLAVTFPGPGEDGLNPFLEVRVT-----DTPKRSRRDF-GLDCDEHSTESRCCRYPLTVD
FEAFGWDWIIAPKRYKANYCSGECEFVFLQKYPHTHLVHQANPRGSAGPCCTPTKMSPIN
MLYFNGKEQIIYGKIPAMVVDRCGCS
>Pygoscelis.adeliae.XP_009332482
---------------------------------------------------MHKLAIYVY
IYLF-----------MLISVDPVALDDG--------------------------------
--------------------------------------------------------SHPT
ENAEKDGLCNACTWRQNTKSSRIEAIKIQILSKLRLEQAPNISRDVIKQLLPKAPPLQEL
IDQYDV-------QRDDS-----------------------------SDGSLEDDDYHAT
TETIITMPTESDFLVQMEGKPKCCFFKFSSKIQYNKVVKAQLWIYLRQVQ----------
-----------------------------------KPTTVFVQILRLIKPMKDGTRYTGI
RSLKLDMNPGTGIWQSIDVKTVLQNWLKQPESNLGIEIKAFDE--------------NGR
DLAVTFPGPGEDGLNPFLEVRVT-----DTPKRSRRDF-GLDCDEHSTESRCCRYPLTVD
FEAFGWDWIIAPKRYKANYCSGECEFVFLQKYPHTHLVHQANPRGSAGPCCTPTKMSPIN
MLYFNGKEQIIYGKIPAMVVDRCGCS
>Melopsittacus.undulatus.XP_005145638
---------------------------------------------------MQKLAIYVY
IYLF-----------MLISVDPVALDDG--------------------------------
--------------------------------------------------------SQPT
ENTEKDGLCNACTWRQNTKSSRIEAIKIQILSKLRLEQAPNISRDVIKQLLPKAPPLQEL
IDQYDV-------QRDDS-----------------------------SDGSLEDDDYHAT
TETIITMPTESDFLVQMEGKPKCCFFKFSSKIQYNKVVKAQLWIYLRQVQ----------
-----------------------------------KPTTVFVQILRLIKPMKDGTRYTGI
RSLKLDMNPGTGIWQSIDVKTVLQNWLKQPESNLGIEIKAFDE--------------NGQ
DLAVTFPRPGEDGLNPFLEVRVT-----DTPKRSRRDF-GLDCDEHSTESRCCRYPLTVD
FEAFGWDWIIAPKRYKANYCSGECEFVFLQKYPHTHLVHQANPRGSAGPCCTPTKMSPIN
MLYFNGKEQIIYGKIPAMVVDRCGCS
>Gekko.japonicus.XP_015261067
---------------------------------------------------MLKLKMYLY
VYLF-----------MLIIPGPADLNES--------------------------------
--------------------------------------------------------HQAN
ENIEKEGLCNACTWRQSTKSSRIEAIKIQILSKLRLEQAPNISRDAIRQLLPKAPPLQEL
IDQYDV-------QRDDS-----------------------------SDGSLEDDDYHAT
TETIITMPTESDFPMPVEGKPKCCFFKFSSKIQYNKVVKAQLWIYLRQVQ----------
-----------------------------------RPTTVFVQILRLIRPMKDGTTSTAI
RSLKLDMNPGTGIWQSIDVKTVLQNWLKQPESNLGIEIKALDE--------------KGR
DLAVTYPGPGEEGLNPFLEVRIT-----DTPKRSRRDF-GLDCDEHSTESRCCRYPLTVD
FEAFGWDWIIAPKRYKANYCSGECEFVFLQKYPHTHLVHQANPRGSAGPCCTPTKMSPIN
MLYFNGKEQIIYGKIPAMVVDRCGCS

```
>Pogona.vitticeps.XP_020658523
---------------------------------------------------MQKFKRYLY
IYLL-----------VLIIPGPVDLNEN--------------------------------
--------------------------------------------------------NQAN
ENIEKDGPCNACTWRQSTKSSRIEAIKIQILSKLRLEQAPNISRDAIRQLLPKAPPLQEL
IDQYDV-------QRDDS-----------------------------SDGSLEDDDYHAT
TETIITMPTESDFPIPVEGKPKCCFFKFSSKIQYNKVVKAQLWIYLRQVQ----------
-----------------------------------RPTTVFVQILRLIRPMQDGTTSTAI
RSLKLDMNPGTGIWQSIDVKSVLQNWLKQPESNLGIEIKALDE--------------KGR
DLAVTYPGPGEDGLNPFLEVRIT-----DTPKRSRRDF-GLDCDEHSTESRCCRYPLTVD
FEAFGWDWIIAPKRYKANYCSGECEFVFLQKYPHTHLVHQANPRGSAGPCCTPTKMSPIN
MLYFNGKEQIIYGKIPAMVVDRCGCS
>Anolis.carolinensis.XP_003217468
---------------------------------------------------MQKFKSYIY
IYLL-----------MLIIPGPVDPNES--------------------------------
--------------------------------------------------------NQAN
ENIEKDGPCNACTWRQSTKSSRIEAIKIQILSKLRLEQAPNISRDAIRQLLPRAPPVQEL
IDQYDV-------QRDDS-----------------------------SDGSLEDDDYHAT
TETIITMPTEADFPMPVEGKPKCCFFKFSSKIQYNKVVKAQLWIYLRPVQ----------
-----------------------------------RPTTVFVQILRLIRPMKDGATSTAI
RSLKLDMNPGTGIWQSIDVKSVMQNWLKQPESNLGIEIKALNE--------------NGR
DLAVTYPGPGEDGLNPFLEVRIT-----DTPKRSRRDF-GLDCDEHSTESRCCRYPLTVD
FEAFGWDWIIAPKRYKANYCSGECEFVFLQKYPHTHLVHQANPRGSAGPCCTPTKMSPIN
MLYFNGKEQIMYGKIPAMVVDRCGCS
>Pseudonaja.textilis.XP_026552688
--------------------------------------------------------MYLC
IYLF-----------MLIIP--VELNES--------------------------------
--------------------------------------------------------NQAN
ENTEKGGLCNACTWRQNTKSSRIEVIKIQILSKLRLEQAPNISRDIIRQLLPKAPPLQEL
IDQYDI-------QRDDN-----------------------------SDDSLEDDDYHAT
TETIITMPTNSDFPISVEGKPKCCFFKFSSKIQYNRVVKAQLWIYLRQVQ----------
-----------------------------------RPTTIFVQILRLIRPMKDGTTSTAI
RSLKLDMNPGTGIWQSIDVKTVLQNWLKQPESNLGIEIKALNE--------------KGR
DIAVTYPGPGEEGLNPFLEVKIT-----DMPKRSRRDF-GLDCDEQSTESRCCRYPLTVD
FEAFGWDWIIAPKRYKANYCSGECEFVFLQKYPHTHLVHQANPRGSAGPCCTPTKMSPIN
MLYFNGKEQIIYGKIPAMVVDRCGCS
>Thamnophis.sirtalis.XP_013915174
--------------------------------------------------------MYLC
IYLF-----------MLIIP--VELNES--------------------------------
--------------------------------------------------------NQAN
ENTEKGGLCSACTWRQNTKSSRIEAIKIQILSKLRLEQAPNISRDIIRQLLPKAPPLQEL
IDQYDI-------QRDDN-----------------------------SDDSLEDDDYHAT
TETIITMPTDSDFPIPVEGKPKCCFFKFSSKIQYNRVVKAQLWIYLRQVQ----------
-----------------------------------RPTTIFVQILRLIRPMKDGTSSIAI
RSLKLDMNPGNGIWQSIDVKTILQNWLKQPESNLGIEIKALNE--------------KGR
DIAVTYPGPGEEGLNPFLEVKIT-----DTPKRSRRDF-GLDCDEHSTESRCCRYPLTVD
FEAFGWDWIIAPKRYKANYCSGECEFVFLQKYPHTHLVHQANPRGSAGPCCTPTKMSPIN
MLYFNGKEQIIYGKIPAMVVDRCGCS
>Notechis.scutatus.XP_026523792
--------------------------------------------------------MHLC
IYLF-----------MLIIP--VELNES--------------------------------
--------------------------------------------------------NQAN
ENTEKGGLCNTCTWRQNTKSSRIEVIKIQILSKLRLEQAPNISRDIIRQLLPKAPPLQEL
IDQYDI-------QRDDN-----------------------------SDDSLEDDDYHAT
TETIITMPTDSDFPISVEGKPKCCFFKFSSKIQYNRVVKAQLWIYLRQVQ----------
-----------------------------------RPTTIFVQILRLIRPMKDGTTSTAI
RSLKLDMNPGTGIWQSIDVKTVLQNWLKQPESNLGIEIKALNE--------------KGR
DIAVTYPGPGEEGLKPFLEVKIT-----DMPKRSRRDF-GLDCDEQSTESRCCRYPLTVD
FEAFGWDWIIAPKRYKANYCSGECEFVFLQKYPHTHLVHQANPRGSAGPCCTPTKMSPIN
MLYFNGKEQIIYGKIPAMVVDRCGCS
```

```
>Python.bivittatus.XP_007425372
---------------------------------------------------MHKLKMYLY
IYLF-----------MLIIP--VELNEN--------------------------------
--------------------------------------------------------NQAN
ENTETGGLCNACTWRQNTKSSRIEAIKIQILSKLRLEQAPNISRDIIRQLLPKAPPLQEL
IDQYDI-------QRDDS-----------------------------SDDSLEDDDYHAT
TETIITMPTDSDFPIPMEGKPTCCFFKFSSKIQYNKVVKAQLWIYLRQVQ----------
-----------------------------------RPTTVFVQILRLIRPMKDGTTSTAI
RSLKLDMNPGTGIWQSIDVKTVLQNWLKQPESNLGIEIKALDE--------------KGR
DLAVTYPGPGEGGLNPFLEVKIT-----DTPKRSRRDF-GLDCDEHSTESRCCRYPLTVD
FEAFGWDWIIAPKRYKANYCSGECEFVFLQKYPHTHLVHQANPRGSAGPCCTPTKMSPIN
MLYFNGKEQIIYGKIPAMVVDRCGCS
>Protobothrops.mucrosquamatus.XP_015672625
--------------------------------------------------------MYLC
IYLF-----------MFIIP--VELNEN--------------------------------
--------------------------------------------------------NQAN
ENTEKGGLCNACTWRQNTKSSRIEAIKIQILSKLRLEQAPNISRDIIRQLLPRAPPLQEL
IDQYDI-------QRDDS-----------------------------SDDSLEDDDYHAT
TETIITMPTDSDFPIPVEGKPKCCFFKFSSKIQYNRVVKAQLWIYLRQVQ----------
-----------------------------------KPTTVFVQILRLIRPMKDGTTSTAI
RSLKLDVNPGTGIWQSIDVKTVLQNWLKQPESNLGIEIKALNE--------------KGR
DIAVTYPGPGEEGLNPFLEVKIT-----DTPKRSRRDF-GLDCDEHSTESRCCRYPLTVD
FEAFGWDWIIAPKRYKANYCSGECEFVFLQKYPHTHLVHQANPRGSAGPCCTPTKMSPIN
MLYFNGKEQIIYGKIPAMVVDRCGCS
>Xenopus.laevis.XP_018094523
---------------------------------------------------MNRLRAWGC
IYLC-----------LLVAFSPVDLTNS--------------------------------
--------------------------------------------------------NKAT
D---KDTLCSACTWRQNSKSSRLEAIKLQILSKLRLEQAPNISKDAIKYLLPKAPPLEEL
IDRYDL-------QSDDS-----------------------------SEGSLEEDDYHAT
TETIITMPTESDYATGDKPK--CCYFKFSSVVQYNKIAKAQLWIYLKPVQ----------
-----------------------------------KRTTAFVQTFRLPKSLNDGARSTGI
RTLKLEMNPGSGTWKSLDVKTALQNWLKQPASTLGIEIKACDE--------------NGR
DLPIAFRGSNEDGLNPFIEVKVM-----DTPKRTRRDF-GLDCDEHSTESRCCRYPLTVD
FEAFGWDWIIAPKRYKANYCSGECGIVFLQKYPHTHLVQQANPRGSAGPCCTPTKMSPIN
MLYFNENEQIIYGKIPAMVVDRCGCS
>Xenopus.tropicalis.XP_002931568
---------------------------------------------------MNRLRAWCC
CYLC-----------LLVAFSPVDLTNS--------------------------------
--------------------------------------------------------NKAT
D---KDTLCSACTWRQNSKSSRLEAIKLQILSKLRLEQAPNISKDAIKYLLPKAPPLEEL
IDQYDL-------QSDDS-----------------------------SEGSLEEDDYHAT
TETIITMPTESD-STGEKQK--CCYFKFSSVVQYNKIAKAQLWIYLKPVQ----------
-----------------------------------KRTTVFVQTFRLPKSLNDGARSTGI
RTLKLEMGPGSGTWKSLDVKTALQNWLKQPASTLGIEIKACDE--------------NGR
DLPIAFRGSNEDGLNPFIEVKVM-----DTPKRSRRDF-GLDCDEHSTESRCCRYPLTVD
FEAFGWDWIIAPKRYKANYCSGECGIVFLQKYPHTHLVQQANPRGSAGPCCTPTKMSPIN
MLYFNENEQIIYGKIPAMVVDRCGCS
>Nanorana.parkeri.XP_018425732
---------------------------------------------------MVKVRVCSY
IYLC-----------ILVMLSPVNMNN---------------------------------
--------------------------------------------------------NQAT
D---KDILCSSCTWRQNSKSSRLEAIKIQILSKLRLEQAPNISKDAIKHLLPKAPPLEEL
IDQYDV-------QGDDS-----------------------------SEGSMEEDDYHAT
TETIITMSTEPDISTTEKQK--CCYFKFSSIMQYNKIAKAQLWIYLKPVQ----------
-----------------------------------RHTTVFVQTFRLPRSSNDGARSAGI
RTLKLEMAPGPGVWKSLDVKAALQNWLKQPESSIGIEIKASDE--------------NGR
DIPLGHRSSSEDGLNPFIEVKVM-----DTPKRSRRDF-GLDCDEHSTESRCCRYPLTVD
FEAFGWDWIIAPKRYKANYCSGECGIVFLQKYPHTHLVQQANPRGSAGPCCTPTKMSPIN
MLYFNENEQIIYGKIPAMVVDRCGCS
```

>Xenopus.tropicalis.XP_002931542
---------------------------------------------------MIKLRAWGC
IYFC-----------MLVVLSPVDLTDN--------------------------------
--------------------------------------------------------NRAT
D---KDTLCSACIWRQNSKSTRLEAIKTQILSKLRLEQAPNISKDAIKHLLPKAPPLQDL
IDKYDV-------QKDES-----------------------------SAGHLEEDDYHVS
AETVIIMPTEFGISIDMKEKPICCFFKFSSKVQLTKISKAQLWIHLKPVQ----------
-----------------------------------KPTTVVVQISRLIKPLKDGTRYTGI
RSLKLEMNPGSGTWQSIDVKTVLQNWLRQPESNLGIEIRAFDG--------------NGQ
DLAVT---SNEDGLSPFMEVKIV-----DTPKRFRRDS-GLDCDEHSTETMCCRYPLTVD
FEAFGWDWVVAPKRYNANYCSGECGIEYLQKYPHGHVVNQANPKGPTGPCCSPTKMSSLN
MLYFNDDAEVIQGKIPAMVIDRCGCI
>Centruroides.sculpturatus.XP_023223240
-----------------------------------------MAPTWTTYVCLITLVSMLA
C---------------MHTSGRITVDGALYGERLEDAIKDFVDEWD--------------
--------------------------------RHNNGSWKLDDVVN-TFKSEEHDNRTED
QEVQMTRNCSRCLMHDDMKALRIDAIKSEILSKLGLRHPPNISG----KAVPKIPPLHQL
LDQYD-------MQKDAPG----------------------EPFMPGPQFEEEVDDYHVT
AEKLISFGQSPSIKWNFPADYRYQYFKFSANVINSHVTGAHLWLYIRPTP----------
-----SSLDS-------------------------IAWIVVYQVMRGETSPS----LLHV
KAKKVDTKLTHGGWATIDVRKIVSRWLRHPKDNLGFAIRSYDS--------------EGR
ELAVTEPKEGEEAWRPFIEMKID----KQRRRRTKRMI-GLNCEENSNEVRCCRYPLTVD
FEEFGWDWIIAPKRYEANYCSGECPYVFLQKYPHTHLVQQANPQGSAGPCCAPRKMSPIS
MLYFDEEYNIIYGMLPGMVVDRCGCS
>Cimex.lectularius.XP_014253049
--------------------------------------------------MTLLGYSLLL
V---------------GCLVGAVESAG---------------------------------
------------------------------------------------------------
-------TCSACLLRTELRSISLANIKEQILAKLGFSAAPNITG----RQIPRIPPLEYI
MDVYS-------MQGDQPQ-----------------------SFKPGPALLEEQDDYSAS
MEKVIAFAQPHPKLRH-WRGQEMLFFKFSDKMLDNKVVRAKLWLYLR-------------
---PGETVENSS------------------------VTITVLRMLRTSSNSDMPTLNTLT
STREARPIRT-GRWVSIDVKKLVVEWFKNPKENMGLGVHAAIPLKDS---------LHTQ
HLIALSSQYEGSSYVPFLEVHIT---DTRK-HRTKRTI-GLNCPENSDEKRCCRYPLTVD
FEEFGWDWIIAPKKYEANYCSGECPLVFLPKYPHTHIIHIANPTGTPGPCCAPRKMSSIS
MLYFDQEFNIIYGLLPGMVVDRCGCA
>Cryptotermes.secundus.XP_023724045
--MTAQRDRLHPEVTRAAMRHLDKEDDDEEATARRRRQRKKTPATDSCGGICVVTVLLVL
V---------------CWCVSPVLAGGNSDTGVATSPFANTTSLYS--------------
--------------------------------SLKLGSRLVRRRDRHPDQEHLAVNETED
EVTTPLPGCQSCIFREGLRNLSLQTIKEEILSKLGMKHAPNTTG----RQLPKIPPLHHL
LQIYEQQQGVPGMQGDEPVRA-------------------TGSFKPGSVVQEEEDDYHAR
TERVIAFAQPYPKLRHGPKGQDVQFFRFSEKVMRNKVLKAHLWMYLRGTTHRQGSKTSHE
GLADAPAPESPGGGYDGGGGG---GGGGDAGWSVPLVNVSVMKVLRGASSSLESPIMKIE
AKSLVRRPSGEGGWVSLNVEELLSRWFENPKENHGIVLHAADE--------------SDR
QIVVTDHEEDNGALVPFVELYTA---DGRK-HRTKRTI-GLNCDETSEEKRCCRYPLTVD
FEEFGWDWIIAPKKYEANYCSGECPYVFLQKYPHTHIVALANPSGTAGPCCAPRKMSPIS
MLYFDNEYNIIYGLLPGMVVDRCGCS
>Limulus.polyphemus.XP_022240786
-MTLTRNRTAVLHQSKVLLGAMCLVLTVCALGVPSPTEKPSNQQDDNIGNLKVLSTMDLE
ARNLTEQIDFLLQQQKELSEEAAENDETN-------------------------------
-----------------------------------------------------HVGKNTE
IVTQGSRRCQKCLTPEQEKKQRLEMIKAQILTKLGMSQPPNITR----KNLQNIPPLYDI
VNRYNL-------QVDVP-----------------------------FFQRDPADEDTAT
TDLAFAFATNTPLEYDQSVEKNVLHFDFSEKVMKSHLKKAHLWLYLQPMK----------
----------------------------------DLQHGGVTVYINQVVRGIESPSLLQH
RVKHVDLRGHHGRWIKLGVRKVVSHWINHPKDNLGLVIQTEDYK-------------GNI
VPISYPSESDEESHRPFIEIEMKK----PQATRSKRDSDGLECDIETTEVRCCRYPLTVD
FVDFKWDWVIAPKRYEANYCSGECPFVFLHRYPHTHLVQQIDPLGSIGPCCAPSKMSSIT
MLYFNDNSNIILGVLPGMVVKRCGCS

```
>Ceratosolen.solmsi.XP_011494685
----------------------------------------------------MIKSVLLL
S---------------LVLLGAFDGPGHLDYIFDTSVKFSFTRWL---------------
---------------------------------------------------YYGFGTTKS
VLAMGSS-CNACRMHEEIRAMSLEAIKEQILNKLGLKQAPNMTG----RALPRIPPISKL
MDMYG-------MQADQP-------------------------LEPGITHHEEIDEFAAK
TESVFAFPQPHQRLRHSKGNLDVLYFKFSDKIVQHRVTRAELSLWIYGAQENR------D
QPSETPGIGEDSEPETI-----------SSPSEGGTLTITLQRIMRGSTDMGGPQLGPPL
TTKYRRPAGRRGLWVTIEVRRMVAEWFKHPRDNLGVAVKIGGANGR-----------RPT
RSFRLVETSPGSENAPYLEVQTQE-LDSRRGARMKRNV-GLNCDEASQETRCCRYKLTVD
FEKFGWDWIIAPKKYDANYCSGDCPMAFLPAYPNTHIVSLAEPPNNTGPCCAPRKLSEIT
MLYFDNEYQIVFSRLPGMVVERCGCS
>Neodiprion.lecontei.XP_015524961
-----------------------------------------------------MTRLLVL
C---------------LVLLGALDGISLSRN-----------------------------
-----------------------------------------------------AVRKTTL
VAQAAGGSCNACRMHEDLRALSLQAIKEQILSKLGLKQAPNMTG----RPLPKIPPISRL
MDLYG-------IQSDQP----------------------VDQVEPGLTRHEEVDEYAAK
TENVIALAQPHPRLRHAKGNLDVLYFKFSDKIVQHRVREAKLSLWVWG------------
--SDHESP-DREVIRSG--------FQQEN--ARGPVTVTLQRILKGSSENGGPLLGPQL
TTKHPRPERNG-DWIVIDVKRMVALWFKHPRDNLGVAIKLQAGSTG----------HRRA
ANSKLIELDPDAEHTPYLDVEIED-LDSRRSGRIKRTV-GLNCDEASQESRCCRYKLTVD
FEKFGWDWIIAPKRYDANYCSGDCPLAFLTEYPNTHIVSLAEPPHNSGPCCTARRMSQIS
MLYFDSEYQIVFTKIPGMVVERCGCS
>Nilaparvata.lugens.XP_022193154
------------------------------------------------------------
------------------------------------------------------------
------------------------------------------------------------
------------------------------------------------------------
----------------------------------------------------------MS
AEKTSSSESENPKIRHGWRGQNILFFKFSEKISLHRVVAAQLWVYVR-------------
-----APIESP-------------------------INISALKVLRGGGSAEEPSVSPIE
GLKTVRPNGTDGSWVSLEVRRLVAEWFKFPRENLGMIVHAVG---------------ARE
HQVVTDIEDDK---VPFLEVHTS---DPRR-LRTKRTI-GLNCDENSEETRCCRFPLTVD
FEEFGWDWIIAPKKYEANYCAGDCPYVFLQKYPHTHIVALANPAGTAGPCCAPRKMSAIS
MLYFDQELNIIYGLLPGMVVDRCGCS
>Athalia.rosae.XP_012258139
-----------------------------------------------------MSRLLVL
G---------------LVLLGALDGISLSRNNDN--------------------------
---------------------------------------------------KSSSRRTTL
VAYAAG-SCNACRMHEDIRALSLQAIKVQILSKLGLKQAPNMTG----RALPKIPPISRL
MDMYG-------IQSDQPI---------------------VDQLEPGITRHEEVDEYAAK
TENVIALAQPHPRLRHSKGNLDVLYFKFSDKIVQHRVTEAKLSLWIWGGG----------
--ESHENPGDPSTRSAG--------FPEQDNLNKGPITVTLQRIVKGTAENGGPVLGPPL
TTKHPRPDRNGGDWIAIDVKKMVAHWFKHPRENLGVAVKL-AGSNGPQTLQQQQQQHVRR
SNFKLLELDPDAEHTPYLDVEIED-VEARRGGRVKRTV-GLNCDEATQESRCCRYKLTVD
FEKFGWDWIIAPKRYDANYCSGDCPMAFMPEYPNTHIVSLAEPPNNSGPCCAARKLSQIS
MLYFDSEYQIVFTKIPGMVVERCGCS
>Cephus.cinctus.XP_015592999
--------------------------------------------------MMIGKHVILG
A---------------MIILGALDGLGPV-------------------------------
---------------------------------------------------GLLTSTHRL
VVDASNGGCNTCRIHEEIRAFSLEMIKEQILSKLGLKQAPNMTG----RAPPRIPPISKL
MDMYG-------MQADQP-----------------------QGLEPGISHHEEYDDYAAK
TESVFALAQPHQRVRHSKGSLDVLYFKFSDKIVQHRVTRAELSLWIWGDN----------
--EGDSHEG---------------------TDAEVPVTITLQRILRGVTESGISLLGPPL
TTKHIRPRGRCGAWVTIELRRMVAEWFKHPRDNLGVAIKIGSP--G-----------HRR
NV-RLVETNPGAEYAPYLEVQTQE-LDTRHGARIKRNV-GLNCDDSSQETRCCRYKLTVD
FEKFGWDWIIAPKKYDANYCSGDCPMAFLPAYPNTHIVSLAEPPNNTGPCCAPRKLSEIT
MLYFDNEYQIVFSRLPGMVVERCGCS
```

```
>Fopius.arisanus.XP_011301623
---------------------------------------------------MITRALFLL
----------------LVVLGAFDLPTSR-------------------------------
----------------------------------------------------VVTWASMA
MANSPNGGCNACRMHEEIRAMSLEAIKEQILNKLGLKQAPNMTG----RALPTIPPISKL
MDMYG-------MQADQP-----------------------SSVEPGITHHEEIDEFSAK
TESVFALAQPHQKLRHSKGSLDVLYFKFSDKVIQHRVTRAELSLWVWGNQ----------
--RDDD--DDDTP---------------DDDTPRGPVSITLQRILRGTTENGSPLLGPPL
TTKHLRPTGKRGTWITIELRRMVAEWFKHPRDNLGVALKIIT--GK-----------TDR
RHTTIVETNPGAEFAPYLEVQTQE-LESRRGARIKRSV-GLNCDEATQETRCCRYKLTVD
FEKFGWDWIIAPKKYDANYCSGDCPMAFLPVYPNTHIVSLAEPPNNTGPCCAPRKLSEIT
MLYFDNEYQIVFTRLPGMVVERCGCS
>Orussus.abietinus.XP_012278233
---------------------------------------------------MITRVLLLS
S---------------LMLLGAFDGPGQQ-------------------------------
---------------------------------------------------QHGRHPHGK
VAYASNG-CNACRMHEEIRALSLEAIKEQILSKLGLKQAPNMTG----RALPRIPPISKL
MDMYG-------MQADQP-----------------------QPLEPGITHHEEVDEFAAK
TETVFALAQPHQRLRHSKGSLDVLYFKFSDKIIQHRVTRAELSLWIWG------------
--TGSRESGDPELDQ----------------ETEGPVSITLQRILRGTTESGGPLLGPPL
TTKHPRPLERRGAWVTVELRRMVSEWFKHPRDNLGVALKISD--GS-----------HRR
NS-RLVETNPGAEFAPYLEVQTQE-LDSRRGARIKRNV-GLNCDEASQETRCCRYKLTVD
FEKFGWDWIIAPKKYDANYCSGDCPMAFLPAYPNTHIVSLAEPPNNTGPCCAPRKLSEIT
MLYFDNEYQIVFSRLPGMVVERCGCS
>Dermatophagoides.pteronyssinus.XP_027194773
MASVSIIANLLFWLFITTTIIISFVNANNNLNDNQIDKQSSSSINISSSSLLMKNRLLLA
DSLLAEQNLNDAIKKLTIVWDLVHNNDNNNNNNLNFSNNNFNNQFLNLSMISLSLQSSKL
QQQEQKTVSHNRSRRSTLSKQNDDDNQMLSDDSSIYSGLSSTENITTSASTMMKNNKQQS
KILRLNRNCTTCLNDEQTRQLRIESIKLNILNKLNMERAPNVSIR----SLPKIPPINSM
LNHLSQMMN---DQSSSPSTLNDLNDSPKIMSKILDLNDYFGPINHQNDDNQDEQEFISA
EKSIVFAQQKPPFDRIVDQELRSQYFKFPSNIYHQHVQKAFLWIYLKGSS----------
-----------------------------------QLKTSSRIVIYQVVRNKQTPLLMIK
SKRISNSTTRKGGWIHLRMEKLLTKWFENPDTNFGIVIHAFDN------------NGQQL
NVIHSDDVEQDSPLRPFMEISVDRKNPLQSSLRRKRTI-GLNCEDKSSEIRCCRYPLTVD
FEQFGWDWIIAPKRYQANYCSGECPFVLMNQYPHTHLIQQIN-LNAIGPCCSPRKMSSIS
MLYLDSDLNVIYGILPNMVVERCGCS
>Wasmannia.auropunctata.XP_011699768
-----------------------------------------MIRPRATLNKMVTRALLLL
S---------------LVFLGAFDAPG---------------------------------
----------------------------------------------------IERT---H
LAMASN--CNACRIHEEIRALSLEAIKEQILNKLGLKQAPNMTG----RAMPRIPPISKL
MDMYG-------MQADQP-------------------------LEPGITHHEEIDEYAAK
TESVFALAQPHQRLRHFKG-LDVLYFKFSDKVVQHRVTRAELSLWIWGMN----------
--KET-ELGEPIDLN---------------DQDAGPVTITLHRIVR--TETGGIVLGTAL
NTKHPRPFGRRGGWITIELRRMVAEWFKHPRDNLGVAVKITGSNGN-----------HRR
N--RLVEISPGAEYAPYLEVQMQE-LDSRRGSRIKRNV-GLNCDEASQETRCCRYKLTVD
FEKFGWDWIIAPKKYDANYCSGDCPMAFLPAYPNTHIVSLAEPPNNTGPCCAPRKLSEIT
MLYFDNEYQIVFSRLPGMVVEKCGCS
>Trichogramma.pretiosum.XP_023317024
------------------------------------------------------------
------------------------------------------------------------
------------------------------------------------------------
-------MCNACRMHEEIRALSLEAIKEQILNKLGMKQAPNMTG----RALPRIPPISKL
MDMYG-------MQADQP-----------------------LGLEPKITHHEEVDDSVAK
TDVVISLAQPHPRLRHSKGSLEVLYFKFSDKVVQHRVTHADLSLWIYGSQEN--------
--RDETENDGLLSLQDYDADSGGGGGGFSQGPSEGTVSILLQRVMRGPTNTSNPQLGPPL
LVKYQRPVGRRGMWVTIPIRRMVAEWFKHPRDNLGVAIKIQIQNGR-----------KVA
KGNRIVATNPDLDIGPFLEVRTQ--LDTRRGSRIKRNV-GLNCDEASQETRCCRYKLTVD
FEKFGWDWIIAPKKYDANYCSGDCPMAFLPVYPNTHIVSLAEPPNNTGPCCAPRKLSEIT
MLYFDNEYQIVFSRLPGMVVERCGCS
```

```
>Camponotus.floridanus.XP_011257959
---------------------------------------------------MVTRALLLL
S---------------LVLLGAFDAPG---------------------------------
----------------------------------------------------IERTHVRH
LAMAGNA-CNACRMHEEIRALSLEAIKEQILNKLGLKQAPNMTG----RALPRIPPISKL
MDMYG-------MQADQP------------------------PLEPGITHHEEIDEYTAK
TESVFALAQPHQRLRHSKGNLDVLYFKFSDKVIQHRVTKAELSLWIWGVN----------
--QETAELGESIDLE---------------DQDAGPVTITLQRILRGATETGGPLLGPPL
TTKHPRLFGRRGGWITIELKRMVAEWFKHPRDNLGVALKITGSGGN-----------HRR
NSPRLVETNPGAEYAPYLEVQMQE-LDSRRSSRIKRNV-GLNCDEASQETRCCRYKLTVD
FEKFGWDWIIAPKKYDANYCSGDCPMAFLPAYPNTHIVSLAEPPNNTGPCCAPRKLSEIT
MLYFDNEYQIVFSRLPGMVVEKCGCS
>Trachymyrmex.zeteki.XP_018302021
---------------------------------------------------MVTRALLLL
S---------------LVLLDAFDAPG---------------------------------
----------------------------------------------------IERAHVRH
LAMAGNA-CNACRMHEEIRALSLEAIKEQILNKLGLKQAPNMTG----RALPRIPPISKL
MDMYG-------MQADQP-------------------------LEPGITHHEEVDEYAAK
TESVFALAQPHQRLRHSKSNSEVLYFKFSDKIVQHRVTRAELSLWIWGVN----------
--QET-ELGEPID------------------QDAGPVTITLHRILRRTTETGEIVLGPPL
TTKHPRPFGRRGGWITIELRRMVAEWFKHPRDNLGVALKITG--SS-----------HRK
NSIRLVETNPGAEYAPYLEVQMQE-LDSRRGSRIKRNV-GLNCDEASQETRCCRYKLTVD
FEKFGWDWIIAPKKYDANYCSGDCPMAFLPAYPNTHIVSLAEPPNNTGPCCAPRKLSEIT
MLYFDNEYQIVFSRLPGMVVEKCGCS
>Ceratina.calcarata.XP_026671545
---------------------------------------------------MVGGAVLLP
L---------------LVLFGAFDAPG---------------------------------
----------------------------------------------------IERTRVKH
LAEAGNT-CNACRMHEEIRALSLEAIKEQILNKLGLKQAPNMTG----RARPRIPPLSKL
MDMYG-------MQADQP-----------------------QPVEPGITHHEEIDEYAAK
TESVFALAQPHQRLRHSKGSLDVLYFKFSDKVVQHRVTRAELSLWIWGNN----------
--REASELDDPGDLESA-----------ESDEDDGPVTITLQRILRGGTESGGPSLGPPL
TTKHPRPVGCRGNWVTIELRRMVAEWFKHPRDNLGVALKISGPGGS-----------HRR
NS-RLVETNPGAEYAPYLEVQTQE-LDSRRGARIKRNV-GLNCDEASQETRCCRYKLTVD
FEKFGWDWIIAPKKYDANYCSGDCPMAFLPAYPNTHIVSLAEPPNNSGPCCAPRKLSEIT
MLYFDNEYQIVFSRLPGMVVERCGCS
>Bombus.impatiens.XP_012245803
---------------------------------------------------MVRGALLLL
L---------------LVLFGAFDVPG---------------------------------
----------------------------------------------------IDRVRIKY
LAKAGNT-CNACRMHEEIRALSLEAIKEQILNKLGLKQAPNMTG----RALPRIPPISKL
MDMYG-------MQADQP-----------------------QPLEPGTPHYEEIDEYAAK
TESVFALAQPHQRLRHSKGSFDVLYFKFSDKVIQHRVTRAELSLWIWGSN----------
--QESSELDEPRDLEST-----------ESHED-GPVTITLQRILRGTTETGGPSLGPPL
TTKHPRPVGCRGNWVTIELRRMVAEWFKHPRDNLGVALKISSPGAN-----------HRR
NA-KLVETNPGAEYAPYLEVQTQE-LDSRRGARIKRNV-GLNCDEASQETRCCRYKLTVD
FEKFGWDWIIAPKKYDANYCSGDCPMAFLPAYPNTHIVSLAEPPNNSGPCCAPRKLSEIT
MLYFDNEYQIVFSRLPGMVVEKCGCS
>Diachasma.alloeum.XP_015115884
---------------------------------------------------MITRGLFLL
----------------LVVLGAFDLPPSR-------------------------------
----------------------------------------------------VVTWASMA
MANSPNGGCNACRMHEEIRAMSLEAIKEQILNKLGLKQAPNMTG----RALPRIPPISKL
MDMYG-------MQADQP-----------------------SSVEPGITHHEEIDEFSAK
TESVFALAQPHQKLRHSKGSLDVIYFKFSDKVIQHRVTRAELSLWIWGTR----------
--RDEDTLDDDSKEADEILED-------DDESPRGPVTITLQRILRGTSDNGTPQLGPPL
TTKHMRPVGKRGTWVTIELRRMVAEWFKHPRDNLGVALKITS--GK-----------SDR
RHSWIVETNPGAEFAPYLEVQTQE-LESRRGARIKRSV-GLNCDEATQETRCCRYKLTVD
FEKFGWDWIIAPKKYDANYCSGDCPMAFLPVYPNTHIVSLAEPPNNTGPCCAPRKLSEIT
MLYFDNEYQIVFTRLPGMVVERCGCS
```

```
>Temnothorax.curvispinosus.XP_024891118
---------------------------------------------------MVTRALLLL
S---------------LVLLGAFDAPG---------------------------------
----------------------------------------------------IERAHVRH
LAMAGNA-CNACRMHEEIRALSLEAIKEQILNKLGLKQAPNMTG----RALPRIPPISKL
MDMYG-------MQADQP-----------------------QPLEPGITHHEEIDEYAAK
TESVFALAQPHQRLRHSKGSLDVLYFKFSDKVVQHRVTRAELSLWIWGVN----------
--QEVTELGEPMDLDG--------------HQDAGPVTITLQRILRGATETGGPLLGPPL
TTKHPRPYGRRGGWITIELRRMVAEWFKHPRDNLGVALKIIGPGSN-----------HRR
NSARLVETNPGAEYAPYLEVQMQE-LDSRRGSRIKRNV-GLNCDEASQETRCCRYKLTVD
FEKFGWDWIIAPKKYDANYCSGDCPMAFLPAYPNTHIVSLAEPPNNTGPCCAPRKLSEIT
MLYFDNEYQIVFSRLPGMVVEKCGCS
>Callorhinchus.milii.XP_007888340
---------------------------------------------------MQTSQALIY
LALFGALGRVGNRDGAHANLTDIKPESL--------------------------------
--------------------------------------------------------EKGP
DSDYNASECSACRWRKENKALRLESIKSQILSKLRLKEAPNISRDTVNQLLPKAPPLQQL
LDQYDI-------QGDDN-----------------------------NDASLEDDDYHAT
TETLITMATEPEPSVQVDDKPKCCSFKISPKIQFNKIVKAHLWIYLRPVK----------
-----------------------------------QTTTVFMQILRLKPVGQEWTNHTPI
RSLKFDINSGTGHWQSIDFKRVLQNWLKQPESNWGIQINASDI--------------NGV
DLAVTSPGQGEEGLQPFLEVKVT-----ETSKRSRRNL-GLDCDEHSTESRCCRYPLTVD
FEAFGWDWIIAPKRYKANYCSGQCEYMFLQKFPHTHLVQQANPRGSAGPCCTPTKMSPIN
MLYFNGREQIIYGKIPAMVVDRCGCS
>Trichinella.spiralis.XP_003375802
------------------------------------------------------------
------------------------------------------------------------
--------------------------------------------------------TESL
KFNEIDDDMVYFQISQETQSNFIESAQLHFYVRSDQISS-------------DTNPAKII
IYQYND----------------------------------------------------AS
GDKRLIKIKEFKRHGRFKGHWDKIDITSSVRLWFSKPERNYGLLIQTVNN----------
----------------------------------------------------NISLTFPP
PVKITDADKYFAQVITFDVHVMDDCFLILENILLTVKISCTKW-----------------
------------HDTTYLNVAVR----DMKAHRRKREIPRMNCGERDNETRCCRFPLVID
FESFGWDWVIAPKKYLAYYCSGECPFRHLQRYLHTHLVQQSNPRGNIGPCCYPTQMAPIL
MVYFNENKEVLVSKIPGMVVSRCGCA
```
