# Supplementary material for: Beyond the Big Five: Investigating Myostatin Structure, Polymorphism and Expression in Camelus dromedarius
Source: Front Genet. 2019 Jun 7;10:502. doi: 10.3389/fgene.2019.00502 (PMC6566074; doi:10.3389/fgene.2019.00502)
Supplement: DATA SHEET S2 — Sequence of the Camelus dromedarius contig-8645394. Exons (highlighted in yellow), 5′ and 3′ UTR regions (underlined), SNPs (highlighted in red), the GAT codon for the aspartic acid essential for BMP/tolloid protease cleavage (highlighted in green), and the 12 nucleotides coding for the RSRR motif (highlighted in gray), needed for recognition by furin convertase, are shown. [file Data_Sheet_2.PDF]

Supplementary Data Sheet S2.

Sequence of the *Camelus dromedarius* contig-8645394. Exons (highlighted in yellow), 5' and 3' UTR regions (underlined), SNPs (highlighted in red), the GAT codon for the aspartic acid essential for BMP/tolloid protease cleavage (highlighted in green), and the 12 nucleotides coding for the RSRR motif (highlighted in grey), needed for recognition by furin convertase, are shown.

>Contig-8645394 *Camelus dromedarius*
AATTAATTAAATTAATTCTTAAATTAATTTAAGATTTTAAGATTAATTAATTTAAGATTTTAAGATTAATTCTTAAATTAATCTGTATTTTAATTAATTA
AATTAATTCTTGTATATCATCACTGTACCTTCTCTTGTTAGTATTAATTTTTTAAGTTCTAGAGACAATGGGAAATAATCTTGAGATGGTCGTACAG
AATTATCATTGTTATGTCCCCATTAACTTCGATTTTTTTCCCACAATGGATATAGCATACCCTTCCCTTAATCATGCATACTCATTTAAAAAAATTAT
GCAACTAAAAATTAAAAACCAAAGTAAAAATAAAAATCACATAGAATACACTCTTCCCCTCAGATAATCATTCTTTCATAAACAGCTGTATGAGATT
ATAGCTACATAATTTTTTTTACCATGAATAGTATATTTACTTCTTCCATAAGGAATAATGTTACCTCACGTGTAGCCCTCCATACCTTTCTGGTATT
GAAACATATTCTAGATAAACAGCATGCTATCAGGGTGTGTGTTTGTGTGTACAAAGTTTAGGGGGGTCATTTGTTTTGATAATAATGTGATCTTAT
TATATAAAACTCTATACATTAAGATTTTCATACTGAAGAGTATTATGAGACTCTGCAAGTCAGCAGCATAAGGAGAAAATACAGTGATTTTAAATA
CAGTTCTGTGAGGCCTTGGGTAACTTTTTAAAAACTCTATGTTTTAGTTCCCTTTCCTATCACGTTGTTACCACCTACTTCACCAGGCTGTGAGG
ATCCAGAGTGCTTCATAAGAACCTAATTTATTCCTTCCAGTGGCTGTGTAGTACCTGACATGGGTGTGCCATACTGAGTCACCTACTGAGGGGC
ATTCACTTTGTTTCCAGTCTTTGACTAGCACAATGTTCAATAAACATAACTGTGTGTACACCTTACCTAGTAGTGATTTTATTCCCATGGGAGAGA
TTCCTAAGCGTGGGATAGGTAAATGTATCATTAATTTCAATGGTTAAAACCATATTTCTTTTCAAAAAGACTGAAACAATGACTATTTCCACATGC
ACAAGAGCACCCTTCTCCCCAAAATTCCTCAGCAATAGATATTTTCTCTCTCAGCTTTTGCTCATCTAATGGCTTTGAGAGGATGCGTCATTGTTA
CTTTACTTCCCTGACTCCCGGCAAGGTGGAGCATGAGTTCACGTTTATTGGCAACTTGGATTTGCCTTCTCCGTATCTCATTTGCCCATTTTTCT
TTTTGGTTATTTATGTTTTTATCCATTAGTGAGCACTTTTGTATGGTTTACAGCTTCTCTATCATGTATTTCAAATAATTTTCCCTGAGTTATAATTT
CCTTTTCCAAAGTAAAATTTTTATTGAAGCACAACATTCATTTCCTTCTGGTTTACATTTGATCTCTTATAAACGAGTTAAAATTTAAAATGTGTGTC
TTCTCCTCCACGGCTTCTTGGTCCCGTCCCGATTAATCAACAACTTGAGGGATTTCTGTGTGTATATGAGACCTATTCTTTCTTTCCTTTAGGCTA
TCTGGAAACTACCTGATACAGTTTTTTCCAGATGAATAGGTTGCTGGGTCAGCACCATTTTAAAAATAACCTGTCTTTTCCCTTTCAACTGAAATA
CCACTTTTGTTATATATCAAATACCCGCATGTACTGGGATCTATTTCTGAGCTCTCTAGTCTCTTCTCTGACTTGTCTCTTTCTAAGCCAAAACCA
AAGCCAATAAGGAGGTCATGATACGCTTCACTCATGCACATGTCACTAGGCTTTACTAGTCTTCTTATTTACATTTCTAATTCCATAGAATCTTTA
AGATCATATTATCCAACCCCCCATCTTATTCAAATTTAAATTAGAATTTATTACATTTGCTTAATTTTGGAAGAAGTCATGTTTTTATGATATTAAAT
CTTTCTATCTAAAAACAACAGATGTTGTTTAATATCTTTGTATAATGTTTTTTAGATAGTCCATTGTTATTTTTTCTCTTTTATAACTTGTTGCTAGTA
CAGAAAAAAGTAATTTATATTTATCTCTCTACCTTAGGTCAAATTCAACTTTAGTATTTCAATGAACATTCTAAACTATCTTCACTGTTAAAGCAGT
CCTTGTTCTCAGCAGACATCCTCATTTCCTACTTTGTTTAAAAAGTTGAGGTTATCAGCTCTCCAGACTTCTCTGTGCACAATCACCTCTTTCAAA
CCCCTTCCTAACATCTTTTCCTCCATTCTCAAAGGGTGAGATTTGTTTCCTCTTCTTCAAGCCCAGGCTCTATTGACTCCAATCCTTTCAAGTTCC
TCAAGGACCTTGCTCCATTAGTTATGCCCCCTTCTCTCCTGCATCTTCCAACTCTGCTACTTATTTCTTTTTTTCAGTCTCTACTCTGTGCTATGTT
AAATTCCCCTTTTCTGTTGTGGAGAGGCTCCTTTAAATCTCTTGGCCAGATTCTGCAGTCTTTCAGCAATTTCCAAGCATCATAACTACATCATTC
TGATTGTTAGTTTTTTTCCTACTCTTTCTTAACTTAGGCTAAAGCTACGGCTCTATCAACTTTATCAGCTTAGGGTGAGAAGAAAGGCCAGAAAAA
TGTCTCCTTCCTTGACTGAGGAAAGTCACAATCCTCTATGTTGGGTATTGTTGCTTCTGTGGTTAACATTGAATAGCCATCAATATCCTCTGGGG
AGCACACGTTCAAATGCTGGCTTTCTCTTCTAATACATGCATGTGCCAGCTAAAGAATGTTGCATACCATCTGTTTCTTTAAGAATGAAGTCACTC
TTCACTGCAGTCACTGACCTTTAACACACCCTGAAAGGAGTTCAGGGTGGAGATCAGGAATGAAGTATTCTGTGCTCTGGGAAAAACTGGCAGA
ACAGGCCTTCAGATAGATTACTTTCAAGGGAAAATTTTATGAACTCAATTTCTTGCATCTTCACATGCCTAGAAAAGCACTAAAATTATTCATGGC
TCCTCATCACTGGCAGCAGCCTTCTACTGAGATGTGTGCTGGACTGCACGTACCCCTTACACTAAAATCATGTATATACTGAACTCCCCCACTA
CCTCTTTGGAGGAGTTCCTCAGAGCTATCTGAAAGGCTGTCTCCCGGGCTATGGTCCTCAGTAAGCCCCAAACAAAACTGAACCTAGAGCTCT
CACATTGTGCTTTTTTTCTTTTTTCAGCTGACACATGGATTCTTAAGTAAGCCTTGGAAAAGAGTCTCTCTATTGTGTAATTTCCTTGAGGGGAGA
TCTGCCTACAAGCATCTATCTCTTCTCCACTCCCCAGTTAGCTTTTACACAGGCAGTCCACCTCACTGCCTGGAGTGGATCCATTCTCTGACCAA
ATGGCCATGGTATTTTGCAACTTCTCCCTAAGATTAGAGTTAACTTCCCCACCCTTTGAATCTGGACTCAGCCGTGAAACTTGCTTCGGCTAGCA
GAACATAAGAAAATGTGACTTATGGATTTGTGTGCATGATACACTTTTATTGTAAAGAAAAACTGAGCCCTATGCTGTAGACAAGAGGCACTCCA
AAGACAATTGAAATTGATAAATTAGGTTGTAGTAGCCTAAGCATAGTTAAGCGTATCAAGGTTAAACACTGTAAGATAATACTAAAGTTGTGAATT
GGAATTAAGAGAAATCCTTAAAGAAGTGGTTACAAACTGGTTTAGCTGTTGCTTATATATGTTGCAAATATCTTCTCCACCCTCCAAAAAGTACCA
TTATCTAGCCTCACAGAGGAATCTTTGGAGAGCAGGTAATTCTAAAGCTATTTAAGCTGTCCCAGTGCCTAGAAAAAGTAAAACCTCCAAATTAT
TTTATAAATTACAACACTGATACTAAAACCTGATAGACTGCAAAAAGATGATCAGTTTTACTTATGAATTTCAATTCAAAAAGACAATAAAATATTA
ACAAACAGCACAGAGAATAATGTGTCATAATCAAATGGGGTTCCTCCCCAACACCTATGCAAGAATATAAGAATAGTTCAATGTACAAAATTAATT
CATGTATTTCAGCATATCAATAAATCCATGGAAAAAAACTATGTTTATACATATAGTCATTGAAAAGGAATTTGATAAGAATCAAATCTGCTCTTGA
TTTTTAAAAAAATACACTCAAAATAAGAGTTGATAAAAACATCCTTTATGAGTATACATATTTTATCTTACCCTTAAGGCTAGCATATTTCCTAGTG
GAGAAGTACTGAAGATATTCTTCTTAAATTTAGGAACTGGACAAAGATGTCCAAAATGACCACTACTACTTCCCAGTGTCTAAATGATTGCCCAG
TATCTCCAAAACTATAGAAAATTAAAGGGATAAAAATTGAAAAAAAAATTGAATTATATCTGTATTTTCTGACGGTTTGACTGCATACCTGAAAAAT
CCAAGAGATAGCAAATGAAACTGATACTAATAAGAGAATTCATCAAGGTAAAAGGATATCAAATTATTAATATACAGAAATTAATCAATTAACATAA

AAAACAATGACAAAGTAATGGGATAAACAGACCCCATTTCAATAACAACAAAAGACAAAATATTCAGGACTTAATAATAAATATCCAGAACCTAAA
GGAGAATCTTTCTAATACAAAGAATATAAAAGTTAACTTGGGAGGATCTAAAGAAAAAAGAACGCTTATGCACTCCTGGTGGGAATGTAAACTGA
TGTAGCCACTATGGAAAACAGTATGGAGATTCCTCAAAAAATTAAAAATAGAACTACCTTCCCATCCAGCAATTCCACTTCTGGGTATTTTTCCAA
AGGAAACAAAAGCACTAACTCGAAAAGATATCCCCATGTTCATAGCAGCATTATTCACAAGAGCCAAGATATGGAAACAACCAAAGTGTCCATCA
GTGAATGAATGGATAAAGAAATTGTGGCATATTTATATTTATATTCAAGTTCATATTTATATAATTCAGTGTAAAAGGAGGAAATCTTGCCATTTGG
GCCTTGAGGGCATTATGCTGTGTGAAATAACTCAGAGAAAGACAAATATTGTATGATCTTACTTACACGTGGAGCCTAAAAAAAAAAAAAACTGAT
CTCATAGACACAGAGAACAGATTGGTGATTGCCAGAGGTAGGGAGTTGGGGTTAGGGGCAAATGGGTGAAGGAGGTCAAAGATGCAAACTTCT
AGTTACAAAATAAGCCTTGGGGATGTAATATACAGCATGGCAACTATACTTAATACTGTTTTGTATATTTGAAAGTTGCTGAGAAAGTTCAGTGAT
AGATATTAACTAGATTTATTGTGATGATCATTTCACAGTATAGACATATACTGAATCATTATGCTGTACACCTGAAACTGATATAATGTTACATGTC
AATTATATCACAGTAAAATTTTTTTAAGTTAACTTGGATAAATGTAAAGATATACTGTATTCTTTAATAGGAAGACTCAATATCATAAAGCAGTATGT
TACATTTATAAATTATTAAGGAATAATCATAATAAAAATACAGGTTATTTATTAGAATTAGATAAACAAATTTTAAAGTTCTCATGAGAAAAATAAGT
GAGCAAAGAAGAAACACCTGGAACATTTTGACAAAGAGCAGTATGGGTACTACTCCTACCAGATATGCAAACATGCTTTAATGCAGCAATAATTC
AAAGTGTAGGATTGGCTCATGAAAAGAGAGACTCATCAAACAGAATAGAAAGTCCAGAGATAGGCTTGAATACATACGGGAATTCAGTGCACAA
TGAAGACTGTAGCCAAGTCAAACACTAAAGACAGACTTTTTGGTAAGTAGTACTGGGGAAACTGGGTAGCAAGTTGGAAAAAAATAAAGCAAAT
TCCATATTGACGTTATTATCAGATTAAACAAATAGGTCCAAATGGATCAAAGATTTAAATGTAATAAAACAAAACTATAGAAGTGTTAGAAAACAA
CATAAGTGGTTTTTATTGTAAACTTGGGAAGTTATTTTATTATAAACGTTCTAACTATGACTCAAATTTTGGAAAGGCTAATAGTTAAAAGTAAACA
CAAACACTTTGGCATGTCAGAAATACGCCATTAGCAGAGTGAAAACAAATAAGATAAAATAAGACATTTGCAACTCATAGCACAGACTGCTTACA
TTCCCAAAATACATAAAAACTTCTAGAAATAGAGGAGAAAAAGACTAACTGTATAGAAAAATAAGCCAAAGATAATGGGCAGGTTATAGGAAAAA
ATCCAATAACAAATAGCCACTAAACACATGAAATGGTGCTAACTTCACTAGTAAAAATGCAAATTAATATTATACTGATATATAATTCTTCAACTAT
TGGATTGGCAAAATCCAAAAGTCCTATGACAAACTGTTGGAGAAGCTTTGGGGAAATGGACACTTTCAAACATTGCTGGTAAGAGTAGAAGATG
GAATAACCATCAGGAGGGAATTTTTGGAAATAACTACCAAAATTTCAGATGCATTTACTCATGATGTAATAATAATCTAATTCCTAGGAATCTCAT
ACATATAAGTGTATATGTATGTATATACACTGAATTAGACACAGATATCATATATAACATACATATAATGTATGTGCCTATCTATATATCTGTGCTA
ACATGAAATGACAGTTGTTCAAGGTTATCCTTTGCAGGATTGTTTGTAAAAGAAAAGGACTGGAACACAGTATCCAGCATTAGGGGACTGGTTG
AATAAATCACAGTAAAACCATACAATCAAAAGCTATGCACCTACAAGAAAGATTAAAGAAGATCTCTGTCAACTACTGACGTGGAGGCATCTCTA
GGATAAATTTTCATGCTTAAAAAAAAGCAAATAATAAATGTACATTTTAGGCCATCTTTTATGTAAGAATACATTGATGCTTACTTGTATTTGCATA
AAGAAACACTGAAAGGACAAAAAATAAACTAATAAAATGGTAATCAATACCAGCAAGAGGAAAAAGGGTGAAAAGGCACAGGTAGGAATGAGAT
TTCTATGTATTCCTTTTACATATATATTGATACTTGAATCAAAAAATTAAAGTTAATAAAAATATCAAGTTTTCTTAGAATCTGATGCCAGTTTTAGA
GCAAAGCTATCTGGATAAACTTTAGCAAAGTAATGCAGTTTTAGATGATTGTATAAGGCAATTGTCCATCTTTTACTTCCAATTAGGTATTTTACTT
TTCAGTGGAACTGAGAACTCAAAAAACTTGATGAAATCATGTGTTGATGGTATCTTTTATGCCAGGTACTGAGGACCCAGTAAGAATACATGTTC
TTGCCCTCAAGGAGTTCAGACTTATGGGGAGACCAAAAATAAGCATGTGATTACCAGCATAAGAATACTAAGCACTACACACAATATGTAAAGTG
ATTAAGTGCCTTCTGCTGCCGAGGAAGACTTCCTAGAAGAGGTGAGAGCCAAGCTAGGCTTAAAGGATGACTAGGAGCTTGAAGGTGACCCCG
GGCAATGTGGGCATTTCAAGCACAGGCAACAGCAAGTGCCACGATCAGTGCTGCTTTCCTATTCTTACAGGATAAACTTCAAGTTCCTCAGGCT
CAAAGCTGCCCTCCGCAGCCTGGCTCCTGCCTCTCTCTCCAGCTGCGTCTTCTGCTCTGGAGGCCTAGACATGTGTAGGTGTGGTAGGAACTC
GAAAGATAGAAGCTGTGGTCAGAGAAGTTTACTACCATAGAAGAGTCCTCCCACAGGGCGGCCCCTGGTCTAGAGGAGCTGCCCAGTGTCCC
CTGGAGTTGCCCATGTCATGTCAGGAGCTCTGATGTAGATGTGGTGGCTGAGCTAGTCAAAGGGAGGTAGCAGGAGTTTGGGATAGAGAGGC
AAATTATGACCTGGTGGGGACAACACTCAATAAATACTGGAGAGCAAGGCCTACGTGAATATAACTTCAATGGCCAAGTAAGCACTGGGTTCCA
AATGCTGTCCTCGGAGACATCTTTTAAATGCGTAAGTCATGTTACGCCCTTGCTTAAAAGCACTGCTTCTCATTGCATTTATGATGCAATTAAAAC
TCCTTCATCTGGCTTTCATATTCAGGTCTCACAAATTCTGTCCCCAGCCTACACCTCTGACCATGCTCTGTGACACCCTCTCCCTCTCATAAGGC
CCTCTGCAGTCTCTGCCATGCCCTCTCCATCCCTTGCTCCCTTGCCTAAATCCTACTTCTGCTATTCTTATTTTAAATGTCACTCCTCAGGCCTTG
CCCCCTGACTACCTCTCCACCATCTGAATTATACGTCTAATTAAATTTTTGCTACAGCACCAAAACATGTGTCTAAATCCAAATCCCATGATCTTA
AATGCTATCTTGTCTCCCAAATGAAGAAAACGTTCTCGTGAAAATAAAAAAGAAAGGTCTGGAAGTGGTCAAGGTGGCTAAAATAGGCGGCAGC
CTGTCTGCTGGCTCTGGCTTATGAGGTTCTGGTTATGGACAAAGAGCAGGCTCTGTCTGAGGAGACTGAAATGAATAAGGTCCTTGGGGTTTC
AACAAAATAAAGATGCGATGGAATATTCTGTAAACAGGTCGAGGGAAGTGGCTTTGCAAAGGAATATGCTTTCAGAGGATCCTGCAGAACTGAA
TTAAGAGACTGGACAGCAGTAAGAAAACGAGCAAGGAAAGAGGGTGAGACTTGTAAGCAGCCACAGGGATCCAACAATGAATGGATGGTTGAC
TGGATGCTGTGAATATTTTGGGAAGTATTGGGGGACTCTGGGAACAAGTAGCTTGGACAGGAATATCAGGAAGTACTCAACCATTGTGACGCTA
CAGTATTGACTACATTCCTTAAGTTTTCTGAGCACTATTCTCCCTGGAGAGGCTTCTCCACTAACATGGCCTCTCTGTCCCTCATCAAGTTCTCG
CAAAACCCCTAGTCCTCTGGAAACTCCTGCTTCACTTCTATTTCTGAGCTCTATCCTGCAGAAAGGGCCAGAAGCAAGAAAATAAATCATGATC
AGTCTTGGAAGTAGAAATAGTGACTACACAAGAAGGCCAGGGATGGGTTTTTCAGGAGAGTTAAAAGTTGAAAGTTTCTGGGGTATATTTAATA
GCACATCTTGTGCTCTCAGAAGTGGGTGTAAATCGGGGTACAGTCCTAGACCACCCAAGAATGAGGCAGGCCTACAAGGAACAGACATGAATG
CTGTTTAAAATCACTAAAGCTCCTGACTTCAGACATATTCTATTGTAGTCAAAAATCTATACATTTATATTATATTCAGAGATAGATAAAATTAGTG
ATATTAGGGTTATTGTGTCAGTCATTAAAAAGCAAGATATTTAGTATTAAGAATTGCTAAGGAAAACTCTACCTTAAAACTGTAATCCAAATAGTC
CAAAACAGAGATCCTATGAAGTGTGCAGAAATAGGCTGATGGCTCCTGAAAGGCGGCAGCTGTGGCTGGCTCCCAGCCACCCTTTGCCTAGA
GACAGTTGCTAGGCAGTACTAGATTTGCTCACAAATGCACTTTTTACCCAATGATCAGTATTATGGTAACACACATGCCACCTCCCAATCAATTTT
TCTGCTCCTATTTCATTAAAGGCAAGAGAGTCTGGAAAACATTACTGATGGCGGCTAATAGTCTTACGGCCACTGAATGGTGCTGGGCTAGTTC
CTGGACAAAAAGATTCAGATCTTTGTCTTCTAGGTCTAGGAATAGTTAGAGAAGCCCCAGAGCTACATATTTCGTTTGTTCTTAATTCCTTTGAGC
CTGAGTGTAAATGTTGAGCAGAATGACGGAATAATGGATCTGAGACTCATTTTCTTTCACTTTCTTTTGTTGACTCATAAAAAAACTACTACTGCA
ACTCATTTAACTTAAACTCACATTGAAAATGCTTAGTAGACAGATCCTGAGAATTTGAAATATGTAAAGAAAACAAACATGTCTAAATTCCCATTTC
CTCCACAAAGCTTTCCTCAGTTCTTCACACTCAAACTGACAGAACACACTCATCAGTAACTTCCCAGTCAGTCCACTGGCATCTCCATCTAGAAC
TAGAATGCTAGTCGGTCAAGAATCTTAACTCATAATGAGAGTTGAAACTTATATAGTGCTTATTCTGTATCAAGCACTAATTTGAATACTTCACAT
GTATTATTCACATCAACTTCATGAAGTAGAAACTATTATCATTCCTATTTTTCAAGTGAGAAAATTGGGGCACAGAGGGGCTATATAATCTGCCCA

AGGTCACAAGGTAAGTGGCAGAGTTGGGAATCAAACCCAGACACACTGGTTCCAGAATCCATGCTCTCAACCACTAATTCTTACCATAGGTTCC
TTCCAGTTAAAAAATTCCTACACATTATCTAGGTCAACCTCATTTGACCAAAGAGAATCAAAGGAGGAGAGATATCAGTTACATAAAGTTATATAA
AGTAACGTTTTTCAAATTTTAATATGGTGTGAATCACCTAGTGGTCTTTTAAAATGCAGATTTTAATTTAGTAGATAAAGGATGAAGACTGAGATTC
TACATTTTTAACATGTCCCCACATGATACCGATTTTACAAGTCCATGTACCACACTTGGAGTAACAAGAACAAAGAGAAATGCTGAGACTGGAAC
TCAGTTCTGACTTATTCCTTCCACTGTCATCCCAAACATTATGCTAATTGGGTAAGGAGGGAGAAAAAAGTGACTGAGAGAATTTACTGTGGAAA
AAATTGACTTCATTTTGAATCATTTTCTTGTTGATTATAAGTAGCGAATAAATTTTATGGCGCTTTTTTCCCACATGTATTTTAATTTTGTAGTAGTA
TAAATGCTACATTTAGGTAGTGCTAAGATTTACCAGCTCTCTCAGATCTGCACAAGCATTGAGCTTGTCTAAGATTTATCATCTATAAATTCATTAT
TCACTTCTCTATACTCTTTAATTTATTGCTCTGGTAGTGACTTAGGGATGTGGGATTGAAGGAAGAAAGAGTATGTGGTAGGAACACACGCTTTG
TAAGACCCCAGAAAGCTGGAGTCAGGACACCACTCCTGAATATTTGGGCAGTCATTCTGTTTTTCCCACTGGGGCATATATTGCCTATGCTCTT
CGTAAATAGTAATGTCCCTGGTATAAAAAGTACAGGACAAAATTAAGCTTCCCAGATCTGCAAAGACTTCCCTTGCACAGCTAGTGTTAACAGTT
GTAAATCCCTCTTTGAGGGGCTCTGGTAGCCACAGGAAGCTGCACTGAGATATACCGGGATGTGTGGCCGGCTGGCTTCCACTAGGTCTGTGT
GACCTTGGTGAAGTACACAAATACGTCAGAAACTTGAGGGGAGTGAGTGTGTATGTTCTCTCTCTAGTCATCTATCGTTTATTAAACCTATAAAG
TAAAAAAAAAAAAAGGAGCACACCAATTCTTCTGATGTCATTTATGTATGCTATGCCCATCATTTAAATAAAAACCACCTATTCCCCCCCCTCCTT
TTTCCTGCCACTCTCACCCTACCCAAATCTGTGATTCTGCAGAACAAAATCTGTTTGCTTTGTCTTCCCTTGCCTAAAGCCCTAATTCTGTAATTT
ACTCTAATGCACCCTTAAACAATTCTGTATCTAGGAGCTTCTACTGTAGATTTTAAGTAAACTTAACCACCAACTGAAGATGTTTTGCTGTTGTTA
GAGAAGGACATAAAATAGAACATTATGCCTTACATTTATTTTGGATAAATTTAGAATACCAGATAAAATAGCTCTTTAAAAAGACCTGAAAAATAAA
CCTTGTGGGTTTCTTTCATAGATGAGAACTAACGCATTATTCAGGGATTAATTTTTTTTTAAAGACACTGTAAAGATGCTTTCAGATCACTATCATA
TTTTTATACATATTTATAAACATGATTTTCTGTTAATAAATTTTAATTATATAATGATACTTCCCAGTATACACTGTACCCTCATTTTTAAAATGCATT
ACTCCTGGAGTAATTAATCAATCTGAATTATTAAAGACATTATTGTTTTTAAAAAGATTTTGAGGGTCATCAAAAATATCTCATGTCATATATTTTG
CATAGCTTCCTATGAGTCATCTGTTGAAAATCAATAGCTTTGAGACCTGCCATGCTGTTTTATTCATTTGCCTCTTCCTTCCAAAATATAATTAAGT
TTACGGGGGAGGGAGGATTTATTTTGTTTCCCTCAGTATTACACTTATTAATTTATAGAAAGGCATCTTGCCTCATGAACTTTTTTTTAAATTGAG
GCATAGTTGATTTATAATATTATATTAGTTTCAGGTGTACAACCTAGTGATTCAAAATTTTTATAGATGATACTCCATTTATAATTACTATAAAATAC
TGGCTATATCCCCTGTGCAGTACAATATGTCCTTGTAGCTCATTTACTTTATACATAGTAGTTTGTACCTCTTAATCCCCTACTTCTATCTTGTCCC
TCCCTCTTCCCTCTCCCTACTGGGAACCACTAGCTTGTTCCCTATATCTGTGAATCTGCTTCCTTTTTGTTATATTATGTAGTTTGTTTTATTGTTT
AGATCCCACATATAAATGATAACATACAGTATTTATCTTTCTCTGTCTGACTTATTTCCCTAAGCATAATACCCTCCAGGTCCATCCATGTTGCTG
GAAATCCTATGAACTTTTATTTTAGTAGAAATTGCCTTTAGAGTGCCATGTCAGCCACCATGATCTTTGTGACAGATTTCCCAACACATATCCCAA
AATAGTGGTGGGAAAGAGAAAGCTCTTTCAGAAACTTCATTATTCCAGCATTGATTTCTGCACTGATTACAGAGTGGCTGATTTCTTGGTTCACT
CCATTTAAGTCAAGTCCAAATTCGTACTTTGTAGAAAGCTGTTTCTAAGAGCACAAGAGAATCTAAAACCATTTCATTAGGGTGATTTTGGAGACA
AAAAGTCCTCTGAATTATGTAGAGAACATCATTTCACTGACCAAGATGGGCTTCACGGAGTCAACTTACTGCAGTAAGTCTCATAATCAGGAGTT
ATCTGCATTTGGCAATGCTATAGACTGGCACACTCCAAACTTAGGAGCATAAGGATTTGAAAGAATTAAGATGTTTTCTGAAAATGCATTTGAATT
AAATGCAGAAGACTTGGTGGAAAAAGGATTTAAAAGCTAATGGTACTAACTCTTGATAACCCTCAGGAAAGCACCAGACTGATTTTACTGCATAT
GGACTAAATGGACTTCATACTCAAATACCAAGTTTTACCAGAAAAGGAAGATTTGGGAGGAGCAAACAAAGGGAAAAAAGTCACAGGTAAAATT
CTGGAGTAAAATAAAACAATATAAAAGAAAAGTTAATACAAGCTAACAAAATTAGGGTGCTAGGGAACAAAGATGATTTGACAATGAAAATCAAG
AAACAATCAATAAACTAATTTTTAAATATGTGCTTTAAAAAGATTGATGAGAAATGAGGAAAACTGAGTAAGATTTAAGTAGACTTTAAAAATGAGA
TTAAGATAGAAAATAATTAAAAGATTCAAAGTCTCATACAGAGATAGTTCCTGTATGTTCTACACTTTGTGTAAGAAAAGTGATCTTAACAAGTGC
TAAGCCTCAGCATTACTGTCAATAAACTTTTCCATATTAACACAAACATTAAATTAATATGAAATAGTAATTGATATAATTAGTAGTAAGCATTTCTA
AGAAATGAAAGTGACGTCAAATTATAAAAAGCTGAACATTATTGTTGAGTCAAAGAATAATGAGAATGATATTTAGAATCATGTAACATGAAAAAT
TTATTTGGATTAAAGAATAGCATGATTTGAATTGCTTCACTCACAAAAATAAGAGCAGCTAAAAGTCTGTGCTAAAGTGGGACTGATAAGCAAGC
AAATAAGAGAAATTAGTCCAACTAGTGTGAGAAGAGAAGCTACCATGGTCTTGTCAAGGTCTTACTGTATCTTTAAAGAGACAAACTGATCAGAA
GCTTCAGACAAGGTAGAAATCGCTACATCAAATGAACTGCTTCAGGGATCCGCTTAAGTCATCCTTTCTCCATTTCTAGCCCACCTACAACCTGA
TTTGAAGGTGGGTCTACACAAGAAGGGCACCAGCACACTTGTCAGTGTGTTCATTCTCACTTTGAATTCAAACACAGCACTCATCAATGGCCTA
ATGTGAAGCAGCCACTTTTTCAATAACAAACTCACCACTAGGTTGCAGTCTTAGCTCAACCGCTCCTTTAAGAACAAACTACAACATATAAAATAA
ATAAGCTGAAAGGATATATTGTACAACACACAGAATATAGTCAAAACTTTGTAATGACTATAAATAGAATATAATACTTAAAAGTTGTGAATTGTTA
TGTTGTACACCTGAAACATATAGTATTATACATCAACTCTACCTCAATTTAAAAAAAAAAAACCGTTCACAAGCTGTTAACCATTTTAATGGTGAAA
CACCATTACTTATCTCTTGTGACAGAGCTGATACAAGCCACACTTAGTTTATCAGTCAGTTTACTAGCCAATCTAGATTCCGTGCAGATACATTGT
TTTTCAAAAGCATCAAAACTCAAAAATCACCTGTCAAAATGTTCTCACTCTTCAGCAGGCAGCCTCCTTTAGGGTTTTCTTGATCACGTAGCCAAA
AGAAAAAAAATAATTCCTTGGGTACTTTAAGCCTCTAGTTTCAGTATAGGGCTAATGGTTAAATAACAAGTACATAATTACCTTTCTCTTTGTTTCT
AGTGACGGTGCTAGGTCCTAAGACCTCCTTGTAAGAGTCAGCCTTACTGTGCTGATGCCTATGAAAAATCCACCTGTCTGTTATCATTGTAGGC
ATGCTCACTAGTCAGCCCCCAAATGAGCTTCAGGTGATACTCATTCTTTAATCCCTAAAGGCATACTTTTAAAGGAACTTGACACTTTTTATTTTT
AAAGGGAAAACCCCAGAGATAGACAGAAATAAAATAAGGAAATGAAACAATGTCTTACTTGTCATGCATTTGAATCCTCTTGAATACTTTTTTAAT
GGAAGTGCCCATAAAAGTAGTGTTTCCCTTTCACGAACGACTGATAAGTTTTGTGGATGTTTTGGCAGTGGAAGGGTGGTGGGGGAGGGAGAT
AGTTATCACAATTTTTTTCTGGAGGAACTAGCTAGATATTTAGTTTGAATATATATTTTAAAATCATTTCCAATAGGTAACAAGGTATCCAATTATAA
ATTTAAAATACATTATATGTAACCACACCAAACTCTCTAAAATTCCAAAAAGATAGAGTAACTGGTAATAATCACATAAAACAGACAACATTCATCT
AAAGCCTAATACATGCCAGACCTCGTAGTTGATGCCTGAAGTGCACTGTTTCATTTAATCTGCCCAACAAGCCTGTAAGGTAGATCCTTTTATTA
TCAGACAGTCTTTTCCTTTGTAGTGCGGAACTGTAAAAATGACTGTGCCACTGAAACTGCGCAAAGAGAGCTCAGTCATCAATGGGAAAAATTA
CAACTGATCCATGACTTCAAAAAATTTTAGAAAATTCTCCTACTGCTGATTAAATGTAGAGGGAAACTTACAAATCTATGAAACTGATGTTAGCAT
AGAATAATTTCAAACATTAGAAACATACCAAACCAGAGTGTTTTATTTCTCTGTAAAAAACTTATCAAGAGTAGTTTGAACAGTTCCAGCCCCCTT
CTCCTTGTTTTATAACTTACATTACAGAGCAAGAGTCTTTCCTCTGCCTTGGCAAATGGTCATTCTCCTTTCTTAGTTTGGATCAGCTTACAATATT
TTACCGTTTGTGCTTTCCACGTCAGGAAATATCTCCAAGATTACCTTTACTGTGAAGATTTCTGCCAGTATCACTTTCTCTGGGACATCTTCCTTT
TCTTGTCACAACCATTTTCCTCCTTTGTGTTGATAAGGTAGCCTTCACTTAGTTGCTCGACTTCAGGTCTAGGGTCCCTCCAACGGCAGCAGTGT

CAACTGCCCCACAGTCAGCTATGTCTTCTATGACTTTATTTATGTTTGATCTGAATTTCACCACCATTAACCTGAGGGCCAGCATTACCACTTTTC
ATTTCGTTGCTGCATTTCATCTCTTTAGGCCAATTTCTCCTTTGGCTAGCCACTTTTGTAAAATGTCCTATGGTTTTATCACCAGGAGCCACAGAG
GCAACACAACTACAGACTTCACTGTCTGGGCATGAACTGAATAATAGATGCACAGTGACCAACCACTGATAGACTTTGAAAAAAACACTGGAGA
ATTTTGAGCAGAAGAGAATGGTGTAGGAGAAATGAAAAAAACATCTGTCCTTTTCCTCAGGCCTCGTGATTAATGGGAAGGAGACGCAGGGAA
GAGAGGTAGATGGTGAGAAAAGCAGACGTGGGGCTCCTTCTGCTCTCCTGGTTTTGGCCGGGAGATGGGAGGGTACTCAGGAAGTGAAGCTC
TTGCTCCATTCTCTGGAAGAAAGTTCTCTCACGTTGCCCCTCCCACCAACATGGAGCAGTTCCAACACTTTAACACAGCATGGGCAGCCAGAGG
GAGGCCAAGAGGTTAGCTTTCCTGGCTGAGAATCTTTCCAAACGGTGGCCCGGTTGGGAAGGTGACCCTCCCCAGAAGCTGGCTATGGGGTA
CACTTCTCAGGCAGGACCCAATGGCAGTCACAGAGTTCAGAGGGGAGCCTTGCTCACTCAGAGTGTGCTCCCTGGATGGGTGGCATCAGCAT
CACCCTCACCTGGGAGTTTGTTAGAAATTTAAATTCTCAGGCCCTACCCTAAATCTACTTAATCAGAATCTCTGGGAGTGAGGCCAGGGATTTGT
GTTTTAACAAGCTCTGCAGATGATTTTTATTCATGTTTAAGTTTGAGAAGCACTGGTCCATAGCACCAACAGAAGCTAAAAGTGAGGCTGAGTCT
GTCAGTGCATCCCACGGAGGCTTCACCCAAGCCAAAAGGACCAGTGGTCTCCTTCAGCCATCAGGCGATAGGGGCTGTTGTGCAAAGGAGGT
GGCAGAAGGGAGACCCCTGGGGCCCCAGGAGCCTGTCTTGCCCCTGCCCCCAACCCCCATGCCTTCAGATCACATAAGCCACATACCTCTCT
CCCCAGATATCTGGCCCTCCTGGAGGAGGAGAGAGGATTGATAAGAGACTCGGTATTTTATGCAAGAAAACCCAAGCACTACCTAGAAGAACA
ATTTAAAATACCGGACAAAATTTTAAACTGGATTGGACATTGTTGATTTTTTTTTTTTTTTTGACTGTTTTGTTTTTCTAAGCAGAGGATGGCGAGCA
TGGGCAGAAGACCAGATCAGCTGCAAAGTAAAAAAGCTACAAGTTACCTGCACACTTCAATTATTGTTTTACAATTACAATATGTTAACATGCATT
CTAAGTTTCCAAGAGTGGAAGACTGTGTAGCATTTCCACACTTATTTCAACACAGAGCCCTTTTCCTCCAAGAATAATAAAAGCTAACCCTGACT
GTACACTTACTATCTGCCAGGGACTACATTAAGCAAAAATACTTCAATAGAATAGACCAGAAAAACATAAAAGTAGATTCCATATCATTCCCACTT
TAGCAGAGGAGATCTGCAGTGTTAACTAATGAATCTTAAGTCTCAGGGGTCCTCACTTGCATAGGCCCCTTCCAAGGCCCTATATCTAATTTTGT
ATTTATAATTTTGTATTTTTTTCTTTAAAAGGGCTACTGTATTTCAGCTCTACTATCACTGGCCACCTGCCTACCACCTACCTTAAATGCAAGGTTT
GTAATAGAGCTTACCTGGTAAAGAAAAATAACTGGGGAAAAGGTCTGCTTTAGCCACTTGCATCTAAGAGACTGAGGAAGTGACATATAGGGTG
CTTAAGGAACTTACTCAGTTAAACCATTAGTTTATCTCAAGAGCTGGGAGTTGAGCCCTGGTTGTCTGACCCCACCCCCCATCCCCTTAATCGC
TAGGCTATACTGCCTCTTTCCAACTTGAGGTGTTATTTCCATGGTAAACTAGTTTTGGAACTTGCTTATAGTAGATTAGGTCAGGCATCTTGGGG
AAAAAAAAAATTAATCCACCTACATACACTCATGCAAAGTTAAATAGCCATGTGAGAGTTTAAGAATAAAGCAAGCCCAGTTCTGCCAGATAGGA
AACTAAGTCCAGGTGGCTGGCTCCCACCTCACATCCAGCTCTGGGCAGGCCACAGGAGGGAGGAAGACATTTAGGAACCTAGAGCCAGGCTT
AGCAATGGAAACAAACCCTGTGAGTGGTCCTTAGGGATCAGAAGGTAGCTGAAGTCGCAATGGGGAGAGAAAACGATGGTGAGGGTGGAGGG
AAGAGCCAGGGGGCCACATGGATTGAGGTAGAGTGTGCTAATAGTAAATTCTTTTTTCTCATTTTTCTGTGGGAACCTAAAACTGGAGTTGTAAT
CATAGTCAGCCAAACTGGGACCAGAATGAGAATACAGAGAACACACGTCTTCTGTTTAACGTTGCAAGTTTGACCAACACGAAAGGCTTGCAAG
TTTGAGTAGAAATTAGAGCTCTGCACAACAAGGCATTTGGTGAGATTTCACAGACATGCACACACACACGCACATACTGCATGAGCACAAACCT
CCCCGACCCAGAACAGTGTCCGTTACATTGATCTCATGGAGATGAATCTCCTGGGATGATCAACTGGGAACAGCCATGTGAAAATAGGGTAGA
GCCTGGACCTAGCTATTGGGAAGCCAGAGGAGGTAGTGCCCAACTGGCCCCTGAGCAGCTCCCCATTCCTTACTTGCTGACCAGGAAAGAAA
ATAAAAAATAAAGCTATTTGCTTTGTGATATGAATAGTGCCTCTAATCGAGGGATTGAGAATACACCAAGAGAGAAGAGAATATACAGTTGAGAA
TACACCCTTGAAAGGGAAAGTAGGAAATAACGGAACTCAGATTAAGAAGCACACACATCTCTTCTCTCTTCTCCTCTCCTTTCCTCTCCACTTTT
CTTCTCTCCCCCGTACCCATGCACAAATGCATGCATACACACACATACACACTTACACTCACATTCCCTATGAGAAATACAAGCCAAGCCTTAGG
CTTTTGTTCAAGGTAAATGTCACTAACCAAGGCAATATATTGTGCCAAAAAATAAAGATGAGCTTAATGGAAAAAACAGTTCGATCTCTAAGGAA
CATAAGCATTTTGAAATAAAGATAACAAATTCAAACAAAGTACAACAAACTTTAACAACATCAAAGACAGAAAAAATTCTAAAAGGCTTCCAGGGG
ATAAAAGAAGAAATAATAACAAAAAAGAAATTAAAAAAACCTTACTGAAAAAAGTAGGAATCAGGCATTAAACTTCCAATAGTAATACTTAGAAGA
CACTGGAGTTATATTTTCAAAGTCTGGAAGGAAAAAGCATTTAAACCTAGACTCTTATTTCCATATAAACTATAAATGCAAGGAGAAATAAATATT
ACAAAAAGTTTATCATACATCCTCTTTGAAATAATTATTATAGGTCATACTTCCAGGAAAAGAGCTATGAATCCAGTAGCAGGCAGTGAGATATGT
GTGAATGAGTAAAGCATTGAGCACTTTATTATTGTTTAAATGAGCAATTACTACAAATAAACCCACTTGATCAATATGAATATGGAAGTTAAATGC
TAAATGATACAAGTAAGAAAGGAGTCTGGGGTGCACCGATATACGTCAAAGTGTGTTAAGAACAAGATAAGATGATTGGTTTATTTTGGGTAAAA
GATTGAGATATTGAAAAACTTTAGGTAATGCCCCTGGTTCAGTAACTTAGGGGTAACTACCAGAAGAATAAAAGTAGAATAAGTAACTTTCAAAA
CAGCAGAAGCAAACTGATTATCCAATAAATGGCAGGAAAGGAGACAAAGAACAAAACTCAGGAAGAAACCATGGTGTCTTTTCTTAGCTTCCCT
CAAAGAAGAGAGCCTGAATCAAGGTCATGTATGCAGGTAGTTTACTTTGGAAAAGAACCCCTAGAGATGGGAATAAGGGGCTGGCAACAGTGA
AAGAATGAGGGAAAGCCAACTTAAGAGTGCATTATCAAGTTAATCAGCATAGTGGGAAACTAGAGCTCAATCCACTAGGGTCTCCTGAGGACCC
ATGTAGAATGCACTTCAGAATTATCTACCCAAGGGATAGAAGAGAGGACTATCTATCCTCCAGCCCATATGCTTCACTTATCAAAGGTTACTCAT
GGTTGTTAACTCATCTGAACTTCTTGGTTTGCCCATGTCACAATGGTGAGAGGTTCTTGCCAATGTCCCAGGCAGTGGTGGTAGTGAAGTCCTA
GAGTAGAAAGCAGAGACCCAGTGTGCAGCCAAGACAACATGCTGTCAGGTTACACCTGTATCCAGGCGGTGGCTTTAACAATGACTGAAGTAA
AAAGATGAGCCAAAAGATGGCAGCAATAATTGTAATTATAGTCACTGTAAATGGATTCAACTTATTAGATTAAAGAAATGCAGGACTGTGTTATTT
AAACTTCAAAGGGCACATACAAAATATTAATAAACTGTGCTTCCAAAGACTTGACACAACCTGTATTCTCTGACTCTAAGGAAATAAATTAGAATG
GAAAAACATTCACAATATTCACAAGGTTGTCCTTCTCCTCCTCCTTTCTTGGTTATAGCAAATTATATCACTGGATGAGTCCTTCTATTGAACAAA
TATAAATATATTGGACAAATAATTTTTAAAAATCTTCTTAAAATTACTGCAGAGCTAACAAGATTGTGAGAAATTACCAGGTGAAGCCTGAAGGAG
GCAGAGGAACCTAAAGAGGCGACCCAATACAGAGTCCTCTTTTGGCCTGAAGGTGTTAACCAATCCCAGCAAATCTGAGCTTTCATTCTGATAA
CTTCATTTGAGTCAGAAGACCAGGGGGAAAAAAAAGCAAGGGTCAGACAAGATGAGGAATCTAATACTAGACATTTCCCACAATAAGCTGAGAT
CTCAAAGACACTCTAAGACTAAGGGTTAACTAGAAATGAACCAACACATTTGTATTACTTCAGTATTCATTTCCATCATTGAGGTAGACCAAGGG
ACCTTAAGTCTTGAACTTGCTTTAATGTGGCCCTGTTATGGTGATAAAATTAATGTCCCTAGATGTCCCTAGGTATCTGGCAGAAGTCAATTCAC
ACTTTCTCTGGAGTAAAAGTTACTTTATTTTAGATCTCAGAATTTCCTGTAATATTTTCTAAGTACAATGGCTACAAGGCAGCCAAAGATAACGAA
GTTAATAAACAAAACACTACAAACAAGAACCGGGGAAAACAGCAGACAAAAGAAACAGATCTGAAGACCGTCAGAAACCAGAATTATCAGCCAC
AGACTATAAAACAACTATGTTTAAACAAATAAGACAAATTTGTGTGTGTGTGTGGTGGGGGGGTCTGCAGGGGAGAAGAACCTATGAAAACTGA
TAGATCATATTCAAAAGGAAGAAAACAAAACCTTCAGAACTGAAAAATACAATGTCCCAAAGTAAGAACTCAGTGGACAGATTAAATAGTGATTA
CAAACAGCTGAAGAGAGAACTGGTATAAGATAAGAAGAAAATATCCAGAATGCAGCCCTGGGAGATGAAAAGATGGAATATACAGAAGAAAAAG

TAATTTGTTTAAAGGATAGAGTAGATGGTCTAACATAATTAATTGGAGTCTCAGAAGGAAATATGAAAGAGACCAATATTTTAAAAGACAACTGAG
AATTTTACGGAATTTATGAAAGACACTATTTTATAAGTTCACAAAATCCAAATAACCAACAGTCAAATTTATAGGTGATTTCTGTACAGCAATGATG
GAAAACTAATAGTAAGTAAAATGGTATCTTTGTTGTACTGAAAAATAATAGTCAAACTAGATTCAGCAACACTATTTTTCAAGAATAAAGGCAAAAT
AAAGACACTTTGTGACAAAAACGAAAGCATTTAGCACAAATACACTCACAGTATAGACAATTCTAAAGCATTGGCCGAAGAAATGAAGAATGAAG
AAAATGATAACTATGTAAGTAAACCTACATAAATGACGGGTGACATAAAACAATAGGAATAATGTCTTATAAAGTTTAAAAAATTATGTATTTTTCC
TGAAATAGTGAACCTGGTAATTCCAATCAATGCACCTGCTGAGAACAACTAGAAAAGCTGGTATTAGAGAGCCTGTGAATAATTACAGGGCTAA
GGTTCAGGAGAAGAAAGAAACAGATAGGAAGCCTGCTATTTGTAACATCTATTAATTTCTGAAAAATCAGCTAATAGGCTGAAAGCTGAAGTGAG
CTTTTCATAGATTCAAAGGGTTAGTAAGACAAAAATGAAGTTCAGGACCAATTAAGTGGTAATGTAGGGCTAGTAACACTAGCTTAAATTAGCAT
CCCAAAATGCTACATGCTAGGAATAAATGAACTAAAATAGAAGAGCCAGCTTCAAATCACCTCAATAACTGGATTGAATTAAAGTAATCTGGGAT
CGCTAGTGCCCCTGGACATCTGCCTGCAAGAATAAATCTTTTCTGGAGGAAAAACAGCATCATTCTGCCCCTCAAATTTTTCTACAATTTTTCATA
TCCAATTCCAGCACTCAATATAGAAAACAAAACATAGTAAGGAACACGCTAAAAAAGTAAGAGAAACAATAGATAATAGAAAGAGATGCACAAGG
ATACAGATATTATTAGATGTAGATACTTAAAATAACGTTTAATATTTTCAAGGAACTACAAATAAAATTTAGATTTTGGCAGGGAACTAAAAACTAT
ATGAAAGAGCTGAGTAGAAATTCTAGAACTAAGAAGTACAATATCAGCTCAATGTATTTTTTTCAAGGCAGATTACTCACGGCTCAAGAGAAAAT
TAACGAACTAGAATACTGACTACAAGAAAATATTCAAACTAAAGGATAAAGAGAAGAAAGAATGGAAAATAGAGAAAACAGTGTAAAAAACTAGA
AATTACAGTGGGGTAGTCTGAAATATAGGTAATTAGAGTTCCAGAAAGAGAGGAGAGAGAAAATGGGATAGAGGCAACATTTGAAGAGGTGAC
AGCTGATAATTTTTCAAATAAGCCAGATTCAAAAGAACGATGAACTGAAAGCAGAATAAACAAAAGAAACACACTAGGGCAAACAATTATAAAAC
TGCTAAAACAAAGACAAAGAGAAAAAAATCTTAAAAGCAGATTACCTACACACATACACAAAAAGCAATACAATTCACAGCTGTCTTCTCAACAG
AAAAAATAAAAATAAAAAAGAACCTAGAAGACAATAAAATGATAGCTTTGAAGAGCTGGAAAAAAATTCCATAACCAACTAAAATGTCCTCAAAAAT
GAACATTAAATAAGGATATTTTCAGACAAATAAAGCAGAGAGAACTGGTCACCAAAAGACCCATTCTAGATGCAGAAGGAAAATGTCATATGGAG
ACTTGGAGATGTAGGAGAGAGTGAAGAGTGAATAATAACTATCTGGGTAAATTAAATAAGTTATAAACAAAATCGTATATATATAATTCCTTTTGA
ATAGAAAAGTGTGTGTATACAAACATTTTATCCACACATATATGTATGTATATGTATATATATGCATGCATGCTTATGTATATATGCATAGACCTAC
ATACCTATATGTACACATATATGCCTGTGTATTATGCTGAGTGTCCTTAGTGAGACTCTTGATGTTTTTATTGGATTGTCCCTCTCAGTAACTAATT
TAAATAAGTTAAAGAGAACATCATGAGAAAAATTTCAAAATATTTAGAACTCAATGAAAAGGATTATTAAGAGAAAAAGGGAGGAAGACATAAATA
ACAATATTCTGAACAAAAAAAGTTAAAATAGACAAATGAATAACAGATTTTTAAAAATTATGACAGAATACCATGAACAAGTTAGTGAATCTGAAAA
CTTGATGAAATGAAAACAAAAATTGAAAAAGATAAAATTTCAAAATTGCCCAAGTGAAAGAAAACTTGAATAGACCAATTGGTACTAAGAAAATCT
ATTGAAAAATTTTTTTAACTCTTTTAAAACTGGCTATGTCTATACTGTTTTACAGGTAAGATCCACAAGAAACCCATAATCTCCAATTAGTGGGAAA
GCTACTCAATTAATTTTATAATACTAGTATACTCTTCACATAAAATCTGATAAGGATAGGAGAAGAAAAGGAAATTATAGGCCAATTTCACATATAA
AAATTGACGCAAAAATACTAAACAAAATATTAAATAATAGAATCCAATGTGCTATAATAAATATTAATAATGACAACAAAAGCAACAACACACTCTG
ATCAAGTAGGATTCACACCTGGAAGTAAAGTTGGAAGGAGGGAAGGATCAACTTAACAGATGTAGAAAAAGAATCTGACAAAGCCCAACAGCTA
TATATGATAAATAGCTCATAAGAAAACTAGAAATGGAAGGGAATTCCCTTGCTTTGATAAAATTGTCTATCAAAAACCTACAGCAAGAGTCATACT
AAAGGAAAAACTTTAAAAGCATTCCCTTTGCATTCAGGAACAAGATAAAGATCCTCATTATTATTGATATAGACACTGTAGGTCCTGGTCAATAAA
AGACAACACCAAAAATAAAAAAGAAATAAGAGGTAGAACATGAAGTTAGCACAGAAAAATTTGTAGCTTCCCCCCAACAATTACAAAACAAGACA
TCACAACAGTAACAAAAACGTTAGTAGTCCCTCTACATAAACCAACAATGAAAACATAAGGAAAACCTATCTGTTAAAATCCTTGAAAGGTTAAAA
ATAAGGTTACAAAATTGCATTAAAATTTACAAGTAAGATGAATAATAAGAGGGATATACTATGTATATGACTGGAATCATGACAAGTATAAGTGAG
AAGGAAATAAGTTATTCTAAATATAGGAATATTAAATCTGAAAACAGAATGTTGAGAGAAAAAATAAACAGAAGCAAATTTGTACAATTTATGTAAA
ATTTATTAACAAACAAAAACTATACTATGTATTTTTAAGCATATGTATGTGTGCATGTCCATACTGACACACGCACACATTCCTGATGAACTCTATT
TCTTGATCATGATGTGTTTTTATATAAACCCATATATTCTATTTTAAGGACAAACATTAAGGACCCAGGAGGCTTATTACTGCCTGTACAAGTGCC
TTAAACGGTAACCTAGACTGGTCCTGAAAGATTTAATCTCTCTAAATCCATAGTTAAGTTAATCTAGTTTAGTTGGAAGAGATGATGCTGGCATAC
TCACTCAGAATGATAATAAAAATCCTCCAAATGCATTTATAGTGTAATATCATACCTCTGCTTGTGTTTATTAATGCACTATCACTTTATGGCTAAT
GGACACTGGGCACTCCGAGGCTCAGTCTCCCATCTGAGATGATGTACTACTCAGTAACTCGATTACCAGTGATGGAATCGAGATTTCATTTCTG
GGGTGGAAACAAGCAAAGTAGCAGACAGACTCCAGCTCAGGTTGTGTTTTGTTTTGTGTTCAAAGTAGGCATTTTGACGAGGTATCTGGGCCAC
CTGGCAGCTCCCACTCACCCCTCCATTTGTAATCACCTTGCTGTGCCCACATCTACACCAGACTTTTCCCTGTGCTCTGCGGGGTTCTCTGCTC
TTCCAGAAGCCTAAGTTACCTGCCCTTATCCACAGAATTAGAATCCTTGCTTTCCAGTTCTTGACATCCCTGTGACATCACTGTACTCAGATTCT
CACAGAGAATGAAGTTCTTTCTAATCTTTGGAAATATTAGGAGTCATAAAGAAATCGTGCTGATCTAGCACCCTTTCTTTGAGCTTCTAAGCGTCT
GAGAGTTTGACCCTGGGACTCAGTGGAGACCCTCTGCCTCAATCAGTGTGCCCCACATTTGTAATCTCCTTTGGCAGACTTACATGTGTGTAAC
AAAGGCTAAGGATGGTTCTCAGAGATGGTAGCCATAGCAGCAGACAGTGCCAGCACCCATTAACAGCTTTAAGACATGACCAGAAGCATTTCAT
TGCCCAATTTTATCTTCTTAACCATCACTGATTATAATTTCTGTATCATTTTTGAGACCAAGTGAATCTATGGTAATAAATAATAATGTTATTTAAGT
TACATAGTCATCCTATATAAGACAAGGAAATAAACTGCTATTTTTATTATTATTCTATTAAAGTATCCACTACTGTATTTGTTCACCTTATTTGCTAT
CATTATTATCCACTCTCTTTATTGTAATTTTGATTTCAACCTACATCTGAGGAAGTCTGGGCTAAATCCGGGGTTTAAAAGAAGTAAAGGGCCTTT
AAAAAAAATAAATACTTCATTCACACCCTGATGTCTAAGGGAAACTAGTGTAGAACAGGACGGAAAACCCACTGTTTTGTAAGAGTCTGGAAAAC
AATTGCTCTTTATTCTCTGTGCAAATTTGAGTACAATGATGTCATGGGGATGCTTGGCCATTTTATAGGTGATTTATTGTCTTACAATGTTATATTG
CGTCTGAATATTGTCAGTTTTCAAAATAAAGGTAATTTTCATGTTACTGAAAGGAAATAATAATATTTGTTGGCCAAATATACCCTTATTATGTGAT
ACTTATGGGAGCAGCAGCTATGACCGAGTGGCCCAGACATTCAGATGTTGGGCCACAGTATTTTGTTGTGATTTTCTCTAGATCTCACCATATG
CCAGGCTGGTGGTTTTCAACATGTGGTTCCTGGGCCAACAGCATCATTATCATAAGAGAACTTGTGAAGATGCAAATTCTCAGGACCTACACCA
GATTTCCTGGATCAGAAACTCGAGTTGAGGGGTCCAGCAATCTCTGTTTTAAGAAAAACTCCAGGTAATCCTGATGCTTGCTAAGGCTTGAAGG
CCACTGCCCCAGGTCAATTATACCCTCAAGATGTAGCACTCATTTATAGCACCATGGAGGGCTAAATGAACCAAATTTTCTGATGCTGGGCTATT
GCTTTTGTTATAAAATTCCCTGGGGCTCATAGGGCAAGTCCACCCCTTGAAGTTATAGTTTCTCTAGAAGAAAATGGACAGTCCTACATTGAGAA
CTCTAGAAAGAGGCAGATCCTAGGATGGGAAGACACGTTAGCCTGCAGGTGAATGGGGATTATTTCTCCTTCCTAGGCCTAGTGATTATGTGG
ACCTTGAGGAGTGGAGGGAAAGGTCTGTTCCGTTTAGGACAGAAAGGCCAAGTGGAGAAGAGCCCCTCCCCAGCAGCTGGGATGTGGTGGA
GCAACCTCCCAGCAGAGGGTCTGCTGGAGTTCTGGAAATGAAACTGTCCACCCCATGTAGAGAGATCATGAGACCATTGTCAGCAGAGGTGGT

GGCCTGGAAGTTTGTTGAGGAAGAAGATTGTGTGACAATTCTTGGGATACTGTCTTAAAAAAAACCTGTTAAGAGTATAGAGGCTAAAGGCAGG
TTGGGTTTTGCAGGCAATGTAATGTTGGCAAAGCGAATGAAGGTTATACCCACAGGCTGGAGAATCCTAGACCTGCATTTATACCTGCTGGACA
GCCTGGGTACCACAAACAACTTCACTCCTCACTTACTGATCTCTGATTCTAATTCTGACTGCCTGCTTTCCCATATCGCACAAAACACCTACCTT
TTCTCTTACCCTGGCCTTTTAATACATACAGATCAATGAGATTTGAAACGCAGGAAATACAGTTTCACAGTTAATTCAAAGATAATGTAAAAATAT
ATCTTATTAATATAGATTATTCTTCCATATCTTCTACAATATTATAAAGCACCTATGAAGCAATCAGAAAGTAAAGAAAGCAGAAATCCCACAGAA
GAATTTTGAGAGGGCTAGCCAGACTTTAGCCTCCTCCAGTAAGAATTTGAGAACGTGGGACAAATTTCAGTTAGAGTCCCAGTCAACCTATTAG
TTCCCTTTGACTTTTCTTTGAGAGTCAGAATTTTATGTTTAAGGTTTTTTAGAAGAAAATATTTTCTTTTCCTTGATCTTGAAATGAGGGCTTCATG
GAATTTTATTCAGAATTCTAAGGAATTCAGAAGAAACAAACAAACAAACAAACAAACAAACTGTAGCTGGGCAACATAGTGTCCACCACCTGTTC
CATCACCTGCTTTCTGTTCTCTCTAAACTTTTTAATCTCCCATTCTCTTTCTGACCCCATAGACCCCCTCCTAGACACTTCTTATGGAGGTGCCAA
ATGAACATCTTGTCTGAGATCTTCCCTGACTTGTGCCCCTGAATCCAGCCATTTGAATGACTGTAGCACTCTGTCAGGGGATACCACATTCTACA
GGGAGGGGTTGCCATATAGGCACCAAGAATAATTCCCAGCTAAGTCATTGGGAATTACATGTGGCCCTTCCAAACATTTCATAGGAATTTTTTTA
TAATAACAAATTTAGCTTTCTTTATAAAACTGGAAGTGATTACATTGGTTAATATATCTTTTTGAGTTGTCTTTGCATAAAATTTGGACAACTTCTCT
CTCAATTTCTATGTGGAGCAGAAACTGTGTCACCAGAAGAGTTTAGTGGCAGGTGTACTGGATCATCAGTAATGAGCTGGCTGTGTTAACTTGG
GCTAGTTACCTAACTTCTCTGGGCCCTCATGTCTTCATCCATAAAATGAAGGAGTTGGACTCTGATCTTTAACAATATTTAATAGTCTTTAAGGTC
TGGTCCAGGCATTAATTTTTTTCAGTGAAGGTACTGGGGATTGAACCTAGGACCTTGTGCATGTTAAGCATGCACTCTATCACAGAGCTATACCT
CTAGCCCAGACATAAATACTTCTGATGCTGTAAAATTCAATTTGATCTGTTCTTTTGCATCACATCACATTGTCTAGTTGAGTTTCCCGCACAGAA
TACTCATAAGTGTTATTGGATGACTATTAGACCTCGTTTCCAAAACTGTGTCCTGGCATTGCTTTCTTGAGACATCCGTGCCTGACTTTGTATGA
CTCCACTCTTTCAGCAACGCCCTTCTGGAACAAGCAGTACTCAGGCTGGTGAAGGATGAGGCTGGACATCATCCTGAAATCAAATAGACTCATC
CAAATAGCCTACTCCAAATTGGCAAACTGGCCGAATTCCATAGTTCTCAAACTAAAATAAAGACTCATTTTACTTCCTTCTCCAACTTGAAGTCCT
GAGACTAAGTAGATGTAAATGAATAAGAAGACAAAAACAATAACAGAACAAAACTTGAATAAAAACTTATTTTAAATAATTGCCTATTTTATGCTAG
CTTTTGTGTTAGATACTTCAAATGTCTTTTTTGTTTACTATCTTAACAATCTGGTGAAGTAAGTACTATTCTTCCCATTTTACAGATAAAGACATGG
TCTCAGAGAAATCTGGTGACCTGTCAAAGGTCACACAACTAGTAAAGACAGAATCAATTAATCCCAACTCAGTTTGAACGCCCCTTCCACTATTT
TAGATATTGACTAATAAACATGTTAAGAGCATAGATATTTGAGTAAAAAAGAACATTATTCCACATAAATATGGCCCTTCTCCTCAGTACACCTAG
AGGTGGCCCACACAGTTGCCAAAGATTACTTAGCAGTCTCTAATTTCAACTCTTCCCTCTCCAGCTTATGTTTCTTGCTGGCAGAATAGTAGGGT
CTAGGAATAGAATCTCCTCAGTTGGGTGAATTTAAGCTGTTCCTCAGTGATGGTCAACACTTAACATCACCACGACAAAAAACATACCTCTGGAG
TTGTTAGACAATTGTTTACTCTAAATTCTAATGAGAAAACATGTTTTAATGAACTATTGATAGTTTCTTTTTTGAGATGGAGACAGAGAATATAATC
ACTTAAAAGCATTTTGTTGAACCGGTTCCTTTTCTTGGATATTTAGGATGCTGTTGCTTGCTCTGAAGTACCTCACCTGGACTATGATTAGGATTG
CACAGACAAGAATTAATCTGGATACAAGAGATAAAAGAACTTATGGCTGCCAAATTCAGAATCTCCCATGAGCGCAGAAAACAGATGATAAGGA
GAGAGGATAGAAAATGTAAAATGACCAACACCCAGGAGCTAGAAAACAATTAAGAACAAAGAAGTCAGCAGAGGCAGAAGACAGATACTGGAT
GATCAGAATTCTGATTAGGGTAAATACCTCCAGAGAGTGGCACAGGTGGTTAGGAAAGCAGGAAGGCGCTTTTTCTAGGGTAGGAACTACAGC
AATTCAATAAAACTGAATACATTTTATAAGGCTGAGTGTACTTGGAATATATAAACTGGCAACCTCCAGGGATAAAATTTTCAGATATCCTTGAAT
CTCAGTGCCGTTTGGCTCCTTAGAACAGATACCATAAAGGCGTATAAGGCCAGGTAGATGTAAGCATAAAAATACAATTAAATCCTAGTATGATG
TGTCCATACTTTTGATTTGTATCATGTCTCATATTATATGCCTTATGAATAGGAAACATAATATATTCTCGCCTCTTTTGCTATAGTTTAAAAACTTT
ATAAATTAGGTGCCAATCTTGTCTCACCACATGAATACTGCAGTTAAGCTCCATTGTTCCTACTTATGCTTCTGGCACTAGTTCTTTGTCATTCAT
TCCAGTCCTCATTCATTCATTGTTCAAGCACTTATTTGTGACACTTTCTCTGTGCCAGGAACATTCTAGTCCTCATTCATTCATTCCTTGTTCAAG
CACTTATTTCTGGCACTTTCTCTGTCCGAGGAAATCTGCCAGATGCTGGAAGAAGCGATGAGTAAGAGAAGAGTCCCATCGTCAAGGAGTTCTA
TTCAGTGGAATAGCCAGGTTCACAAACAGAAAACCCCAGTACAGTACGACAATGCTGGGGCAGAGGTATGTCAGGATTTTAGAGGCAGGAACA
GGATGAGAACATTACTAGGAAGCCCCAAGTGTCCAGCTGAGAATGGAGACAGTGAGTTTGCAGCAGCATCAACTCACTCTGCTGCTAGAGAAA
AATCACAAAATATATCAATCCACATAAAGAAGCAAAGAAACCAAGTGGATTTGCTAGAGATCTACAGATTTGCAAGGGCGGTAGGGCAGGAAAA
AGAGGGATCCAGGGTTACGGGGTACTTTTCATGAAAGAGCTCAGTCTAAAGTAGCTACGCAAGAAAGAAAGCTAAAAGAGGTCAGGAGCTTGT
AGGTTAGAAGAAAAGAAGGATCAAATCTCAATGGGATTTAAAGGGCAAGAATTCAGAGACAGTGAATTAGGCATTTTGGTTCAATAACTCAATCA
GCCAATAATTAGGATATTCATATAACTTATTACTCAAATAGTGAAGGGAAACAAATGATTATTAATCATGACTCCAGGCGGCAGATGTAAACTGG
GACAATCCTAGGATGCCTAGCTGCCCTGTCACTAATCCATGTGCTAGATACTGTGCTAGGTACTGGGGATAAGGTGAACAACAAAGCAGAAATG
GTGCCTGTCACCATGGAGTTCATAGTCATACAGGAATGACAAACACTGAACAAGAAATTATTACTGTGATGAATGGTGCTAACAAGAGAGATGA
AGGAGCCAGGAGAGTTTATTTTGATTTTAAAAATGTTTTTTTCTATTGAAGTATAATCAGTTTACAAAGTAGTGTCAATTTCTGGTATACAGAATAA
AGTTTCAGTCATATGTACATACATATATTCCTTTTCCTGTTCTTTTTCATTATAGGTTACTACAATATATTGAATGTAGATCCCTGTGCTATACGGT
ATAAATTTGTTTATCTATTTTATACATAGTAGTTAGTATCTGCAAATCTCAATCTCCCAATTTATCCCTTCCCACCCTCTTCCCCCCAGTAACCATA
AGTTTGTTTTCTATGTCTGTGAGTCTGTTTCTGTTTTGTAAATACGTTCATTTGTGTCTTTTTTTTAAGATTCCACATATTAGTGATATCATATGGAA
TTTTTCTTTCTCTTTCTGGTTTATTTCACTTAGAATGATGATCTCCAGGTCCATCCATGTTGCAGGAAATGGCATTATTTTATTTCTTATGGCTGAG
TAGTATTTCATTGTGTGTATATGTATATTTCTGTGTATACCACAACTTCTTTATTTAGTCATCTGTCAATGGACATTTAGGTTGTTTACATATCATG
GCTATTGTAAATAGTGCTGCTGTGAACATTGGGGTGCATGTAGCTTTTCGAATTGGAGTTCCTTCTGGATATATGCCCAGGAGTGGGATTGTTG
GATCATATTGTGAGTCTATTTTTACTTTTTTAAGGAGTCTCCATACTGTTTTCCATAGTGGCTACACCAAACTACATTTCCACCAACAGCATAGAA
GGGTTCCCTTTTCTCCACAGCTAAGAGAGTTTATTACAAAGGAAACCAACATCTTTTCAGAGGTCAGCAAAGGGTCCCCTTAGGAGATGACATTT
AAGCTGAGACTGCAAAAGTGAGTAGGAGCTTGAAGGTAGAAAGGGGAGGTATGAGTGCCCAGGCAGAGGGAAGAGTGTTTACTCAGGCCTTG
GGATGGGAAAGGATGTTACATTTGATTTCTAAATTCTAACAGATTTTTAAAAATCCTTATGGGTCTTTTTATCTTGCTGTTGTTTTCTTTGCTTATG
GTTTAAATTCCTTCCTTCATCGCTTTGAACATTCTAAACACACTTTGTAAACATTTCATTCAGGTCGTTCTACCCTTTCAAGGTTTGGCTCCTATTT
CTTTCATCTGCTGACTCTTCGTGGACAGTTGTTTGTTTTATGGTATGATATATAACTGGTGACTGTGAGTTAGTCATGAGAAGAGATTCTCCGTG
GGGACATAGTCTACAGGGTGCTATTCTTTTATCGCAACTTGAGCGAAAGAATCAGCTTTCTTTTCAGGTTGATTTCCCAGCTTCAGGTCCCACCA
TACAGGCCATGTGATTTTAGACTCTGTGTGTAGTGCAGACCTGGGGTTTTGATTTCCTGCAGGTGAAAACTTCTCAGTTGTGATCTTGGGATACT
ATAAACCTTCTTGTTTATCTGGGCTGATGGGCAGAGTACCCTTAGTCTTCATTTCAAAGTCAGCACAACCCTCTGTGACTTCCAGATTTATCTAAA

TAGCTCAGTTCAAGCTTACCTCCCAGGGCCTGGAGCTTAGACCCGCCTTCCAGGGCAGTTAGTTGTTAGAACCAATTCCAGCTTCTAAGGCCC
ATATCTGGTTGCAATAATTCTATGAGCTGTTTGGGCATAAGAATCTGCTTAACACACTGACTTGAAGCTTCCTCTTTAATTCTGACACTTGGGCG
ACACTCTTTCTTGGCTTCAAGCTTAGCTACATACTTTAAGAAGTTTTAGCCATATTTTATCCTGTATTTAGAGCAGAAGAGTGAAGAATTCTTTGG
CATCAGCTCATGATATAATACAGTAAATTTTAGAAACTTTTTAAAACGTATTCATTCATCAAAAAAATTGTGGAGCACCTATGGTGTTCTAAGTATT
GTAGGACTTAAGAACAGAAAATGAGAAAAATAAAGTTAAAGAATGAGACGGATTTGTGTAAATGACGTTAAGAATCTGGATTTTTCTTCAGAGCA
AATGGGCAGCTAGTGAACGATTTTAAAATAAGTTTGCATCTTAAAAATGCAGAATGGAGAATGGATTGGAGCCCAAAAAGAACAGATGTGGGAG
GAAGTGTTCAACAGTCCAGTGATTGATGATGGTGGCTTGAACTGGACAGTGAAGACAGATCAAAGTGACTGGACTTAAGAGATATTCAGGTAGT
AGTAATAACTTACTTATGGGCTTAACTGGGGAAGGGATGGAGATGAGTAAAATATCACTCCCAGGTGCTTGGCTTGAGTATCTGAGGGGTTGTT
AATGGCACTTAATGAGCTAGAGAGTACAAGGAGAAGGAGCAGATTTAATAGGAAACATCTCAAGTTCAGTTTTGGGCATGTTGAGTGTAAGGCA
CCAGACAGCCCAGTGGAGATATCAAGTAAGCAGTAAGCTAATTGTGTTGGAGACTCAGAGCATAAGTCTGAGCTGGAAATAAAAAGAAAATTGA
GTTTTTTGGAGTAGATGAGATTTCCTGAAGAGAGGGTAGAGTGATCCTAAAATTCATACAGAACCACAAAAGACCCTCAATTGCCAAAGCAATCT
TGAGAAAAAAAGAACAAAGCTGAAATTATCATCTTCCCAGACATAAGACTATATTACAAAGGTACAATAATCAAAATAGCATGGTACTGGCACAA
AAACAGATATATAGTAGCTACCAGAGGGGAGAGGGAAGGAGAAGGGACAAGTTATGGGTATGAGATTAAGAGGTTAAAAACTACTATGTATAAA
ATAGATGAGCAACAAGGATATATTGTATAGCACAGGGAATTATAGCTGTTATCTTGTAATAACTTTCAATGGAGTATAATCTATATAAACACTGAA
TCACTACACTGTACACCTGAAACTAATATAATATTGTAAATCAAATGTACTTAAATAAAAACCAAACAACCCCCCCGCCCCCAAAAAAGAGATAGA
GGGTAGACCAGAAACAGAAGAGGGTTTGTAAGTCCCCTTGAGGAATTCCATCACTTAATGGCTGGGTAGAGGTAAAGAGCCAAGAAAAGAACA
GCTGGAAGGAAGCGTTATATCAAGGGTGACATTCTTCCCCTTAAGAAGGAATGGCTTGACAATATTTGCTGATAGGAAGAACACCAAAGAGAAA
GACTATAATTCCATGGAGAGAAAGTACAACTGCTAACATAAAGTACCTGAGAAGCTGGAAGAGGATAGGGATTCAGGGCAGAGGTAGAAGGAT
TGGTCTTTGACAGGAGGCAACATTGTAACAAGAGGGAAGGAAGACAGGATGGGTACAGATGCAGATAGGTTTGTAAAGCTGATGTTTGAAAGG
CTGTGGTAGTTCTCTGACAGTGTGTTACTTTCTCTGTAAAGCAGATGAGCTCATGTGCTCCATGTACTAGGGTAGACAGAAGGACAGAGGCTTG
AGAAGACTTTAGGATTGAACTGACTATGAAGACAATGGCTTTCCAGGCAATGCTGATGACACAGAAATCTGCCAGTTCATATGATTTTTCCCTAT
TAGTGCTCTTCAGTCTGAGTACAAACAGAAAAGGTGGGTGGGTAACTAGATATATCTAGCTTGGGCTTTGTCAGCTTGGGGCCTTGACAGACAG
GTGCTACAAAAAGACAATGAGGCAAAGGTAAGTTAGAATATTGACAGGAAAGTGGCTAAGATGATGGCTTATGGACTATGCATTGGATAAAGGA
GGAAACTAAATACAGAAAGGAGATGATGAGAGGAAGACAGCAGAATGGCACAAACTGAATGTTTGTACCCTGCCAAAATTCCTATGTTGAAATC
CTAACCCCCAAGGAGATGGTATTAGGAGGCGGGGCTTTTGGGAAGTGGAACCTTCATGGAGGGAATTACTGCCCTTATAAAAGAGATTCCATA
GAGTGCCCTAACCCCCTTCCACCTTATGAGTGTACAGAGAGGAGGTGCTGGCTATGAACCAGAAGAAGGCTCTGACCCAATCACCTGGCACCC
TGATCGCCAGCAGCCAGGACTATGGGAAATAATTTCTGCTGTTTATAAGCTACCCAGTCTGTGATATTTCGTTATAGTAACCCTAGCAGACTAAT
ACAAACGATCAATGAACTGAAAGTTGCAAGTGAAGTAGTGGTAGATCTAAGTGACAAAGCCAAAAAGGTCAGAGACTATAAACTAAGAATGTGA
AGTTTGCAGAAGTGGTTTTAAAGGTTTTAAAACACTTCTCAGTGATGAGTTCCAGAGTTTGATTCTGGCCATGGTAGAGGTATTAGTCAGGATAA
TATAAGCTATTCTGCATGAAAAGATTGACTCCAATCTCAGATTAAAATCTCAAAGAAAACAAAGCTTTATTTATCACTCATGCTGTTTTATTTTGAA
ATGCTGCCATTTCAACACATAGCTTCCAGGTTTATACAGCAGGGGAGGAAAAGATGGCACTGAGTTAGTTCTTAAATGCTTGGATCTGGAAGCA
AAACATGTCCTTTAGGCTCACAGTCTTTTGTCCAGAAGTAGTCATATGGCCCCATCTATTTGCAATGGGGCTGGGAAATGTAAAGGAACACATG
GATATTTAGTGAGCAATAAATGTCTCTTCACAGCAGCTGAGACGGATGAGGAAGGAGATGATTAAAAACAAAGTGATTACAGAGCGTCATCCTT
GATGATTGCCAGGGTTGTGTTAGAAAAGAAACCCGAATGAGGTGCTAGAGTTTTCAGTGAAGGAGGTGGGCAGTTGCAAGTTGGCAAATGAAA
GCAATGAGAAGAAAAAGAGTTGGATAGTGAGTCTTAAAGAGAGTGGGATTTGTCCGTGGGTAGAGAAGCAAGTTCCCTGGGACAATACATTTG
GACACAATAAAGAAATTTATCACCTGGGCAATAAGAATCCAGCCATTCCATTCGACCTCATGGAAACAGGACTATTGCTCAATAGAGTCTTTCAG
AGAGCTAAGAATCGCCATAAAGCTGGTCTTCATAAACACAGAATGTTGACTGGGTGGCAGGAAATTATTTCTGAATATTAATTGATATCTTTCCTT
GTACTTCTTTCTCTCCTTTCGTATTTGCCCCTATAAAGAGCTAGTTTGTGCTCACTGCCTTAGGGTAAGGACTGTGGCAAGGTGGAAAGCAAAG
GACCTGAAATACTACTCCTAAGTTCTAGTCTACAAACAGCCTAGGTCATGACAAGAGTGGAAAGGTATGGTAGGCAGAACAATGCTCCCCCCTC
AAAGATGTCCATGTCTTAGCCCTCGGAACCTGTGAGTGTGGTAGGCTACACTGCAAGGGGGAAATAAGATCACAGATGAATTAAAATTACTAAT
CAGCTGACCTGAAATTAAGGACATTTAAATGTAATGACAAGAGTCTTTGCATATGGAAGAGAGAGGCAGAAGAGTTAGTGTCAGAGCTATACAG
CATCAAAAGACTTCCCTGGCTATCACTGGCTTGGAAGACGGAAGGGGGACATGAACCAAGAAATGTGGGCCATCTTGAGAAGCTGGAAAAGAA
ATGCATTCACCCCTAGAGCCCTCAGAGGGAACACAGCTCTGCCAACACTGCTTTTAGCCCAGTGAGACCCATTTCAGACTTCTGAACTTCAGAA
CTCCAAGATAAATCTGTGTTGTTTCCAGTCACTAAATGTGCAGTGATATGTTACAGCCCCAGTAGGAAGTTAATACAAAAGGCAAAGAAAAGGTT
TTGAGAGTTGGCAGTCGGAGGAGGGAGTTCTCTAGGAGGAGGATAGGTCAAGCGATATCCCAGCAGAAGCTGAGCAGAACCTGGAGATTAGT
TAACAGCAGAAGAGAAAAGCCCAGGACCCTGCACTCCAGGCCAACATACAGGGAGGCAGCAAACTCCCTGAAACAGAGGTGTGTGATCCGTG
CTTCACACACGGTAACGTGCAAGGAGACGGCAAGACCCTGATGGAGGACGGCTGTGTGCTTAGTCAGAAGGCACTTTCATGAGCCTTATGAAG
AGTTCATTTCATTTCAGCTGACAGTATTTTGCCCTCTTTTCCAGTGCTCTACTCGTAACATTCTTTTTGTTTAGTGTGGTGACTCTGTAAAGTGCT
AACCTGGTTAGCCTGAACTAAATTTCTTGGTTTTCCTTTCCTATAGGTTAAGGTGTGCCCCAAGAAAGATTCTGGTGGGCGATGTGGAGGGCGG
AAGGGAAGGAGCATCCTTTATGTAGCTCAACTGTGTGGTCACTGACCAACTGGCTCATCTCCAGGGTGTGAGGCAGCGGACGCTGCTGAAACC
ACTCCACCTCCGCCCCCCACCCCCATCCTCCTGCCGCTTCTCTGACTCCTGAGCCAAGGGTAGCTGTTTAGCTCCGTGAAGAAAGACCCACCC
CCCACCCTCACCATGAGGGGCAGTGGCCATAAGAACTGACACAGTCTTCCATCCATCCTGTGGGCTCCACCTCAAGCTGTGGGTTCTGGCTGG
TTCTTGCTCTTTCCTACATCACATCCCTCTTTCCTTCCCAACTGCTTGCCAGGGACTTCCAGGTCCAGGTTCAGAGGTGAAGACAGCAACCTTA
CAGAGACTGCTTAACTAGATCCCCCATTACGTAAGGTCAAATTCCTGCAATAAGCCCCTCGAATATGATATGATATAAGATAATGTATATGTCTAT
ATACACCACACTCCTCTGAACCCCTTGAGTTTCCATCATATTTATTTCTGATATTACCAAAAATTCTTTTCAATGTCTGTGATTTGTAGGAATTACA
TTTAATGTCTTTCATGGATTATTATCCTGGTCATTTAATCTTTTAACTTACCCCTCACATTCTTTGAATACCATTTTTCCTGGACCAGTGGAAAGTA
AGTGACTCATTCAGATAAAGTAATCACTTGTTTCCTATTTAAAGCTATCTTTAAAACTCCTCACCTCTCCTTTAACACCCTCCCAAAAGCATTGAAA
GGATCATTCAAAATACATAATGGATATAATGCTAATAGGGAACCAGTGATCCATGAATAAATTACGAATAGTGACAGAAGGCAAGGCGACTTTTA
TGATTCAGCTCAACAGACTTTGCAGGCAGGAAAACATCCTGTAATCAATCTTGTGATTGCTGAAAACATGGTTCAAAATAAAAAATGAAGTAATC
TTGCACATATAGCCAGAAACCTAAGAACAAACACTTGCAAACTTAGAGCTCCACTTCTCGGGCTCCCTTTTAAACAAAACAGGCTGCACAGAAG

GAGTGGGGGATCTGATAGTCAGATACGCCTGGGTCGAAATCATGGCTCCGACATTCTGTAGCTCTGGGATTTTAAGCTGATGTTTTCACTTTCA
GAAGCTCAGTTTTCTCATCTACAAAGTAGCAGTAATGCTACCAGCCTCATAGGATTGTGGAAAAGATAATAAATAATATGTAAAAGTTCTGACAC
ATAAAAGGCACTAGGGGTCACCACCTATTATTATTAAATACTCCAAATAGCCCGTGCATCCCCTTTGTTAATACCTTTAAATATATATGTATATTAA
ATATATGTATATATTGATATATAATATAATATATATTAAATAGCTATATATACATATCTGAAAACAATGTCCTGGAAATCCCTACAGGTCGGTGCAT
ATGTACTTTCCAATTGCTTAATCTGGAACTTATCTTATGTTCCAGCCACAAGAGCCATGTTCACCAAAACTCTGTCTACAGAATCTGAAGTCAGAT
TAAAGCAATGTGAGACCGTGTCCTCTTTATGGCTGGGGGAAGGTGGACGCTCTTGGCAGGAACACTGAAGCCTCAGAGAAAGTAAGGCCCCTC
GTCCTTCATCAGAGAGCTGCTGCTTCAGTAGCTGCACTAAGTGCCTGGTCACAAGACAGGGGAGGACAGGAAGAGGCCAGGCCCTTCCCGGC
CCAACTTGAAGGTGAGTGGGTGAGCGGACTGCGTTCCACTCTGACGGCCTCCAGCTGTTTTCCCACCTTCTTCTGTAGGTTTCCATGGTCAAG
TTACCAAGCCAGGGAATTCCCAAAGTGAAAATACATTGACAGCTGTGTTTGGAATCTATTACCAACATTTTAACAATTATACACAGGGCCAAATTT
GACCTCGAAATTACTGGAAACTTATACCCAAAAGACTGAATGAAAATGAAGAGAGCACATAAATAAAAATGACCCCCACGAATAAAGTGAAATTA
TGGGTGAAGAAAGGAAGACATAAAAACAAAACATTATCCTGTTACAGACAATAGTTTAAAAAATTCTGGTGCCAAGGGTATGAATCTTTACTTCTA
TAACCTTAAATAAAACTCTGTTTCCTCCTACCTGTGCTACAAGGAATCCAAGAATATTTATTTTCTGATTCCTTGCCACATGTATTACTATTATTTT
AGGTTAGCATTTAATGACTTTTTTTTTTAAAGCTGTAAACATTCATCTTCTGCCCTGATATGCATATATGTACTATCTATATATTTCTATCTATGGTC
TGAAGTATCTGAAAGTGGTCCAGAAGAGCATCATCTTTATGACTAGAGACATTATTATAGTCTTAAAAATGAAAAACTGACAACACAGCATTTCTA
GTACTTTCCCTTTCTTCTGCTAGCAATGAGCCCAAGGTAAGTGAAATTCTATTCACAGAAGTTAGAATTAGACAACAGAACTCTGGTTTTAATAGC
CAGTCCTCTCCTCTGATCAGATACAGCTATCCCCTGACCAAGGAGATAACAGTTTTTACAGATCAGATGGACAATGATTCTTAAAAGGCTATTAC
CCTGGGTTGGCAATGGTTTGGGGAAAACAGGCACTTCTGTTCCATTTTTTAAAAGAACTGAAGTATAGTTAGTTTACAATATTGTGTCAATTTCTG
GTGTACAGTGTAATGTTTCAGTCATACATATACATACACATATTCCTTTCCATATTCTTTTTCATTATAGGCTACTGCAAGGTATTGAATATAGTTC
CCTGTGCTACACAGTAGAAATTTGTTCTTCAACCTATTTTATATATAGTAGTTAGTATCTGCAAATGGAAATAGACACTTTTGAATACTCCTACTG
GACACGTAAATCAATACAATTTCCAAAGGATAATTTGGAAAAATGATTTAAATTCTTAAAAATGAATTTATTGTAAGTACATATATGTGTATTTACAA
AAAGATTTACCATCAAGGATATGTTTTGAAAAGTTATGACAGGAATTTGAAAGAACCTAAATGTCCTACAAGAAGATATTGATTAAATAGGTAAAT
ACTAATATGACATAATACAGATAGTCATTAAATAATAATGTGTAAAAAATATTTAGTGACTTGGGAAAATATTCACACTATAGTCAGTTAACTTTAG
TTTATTTATTGAGTAGATAAACCAAAATGTTAACAGTAATTATTCCTAAAGGAAGGAATTATGAATGATTTTTAGTTTTATTTTTGTACTTTGTATAC
TTTTTAAAATTTTCCATGCTGAATATATGGAATATATATTACTTTATAATCATACTCTTTCTGGTATTAAAATGGAGGTTGGAAAGAGGCTTTAGGG
CAGACTGAACCCCTTTAGAGTAGTCTTAAATTGAATTATAGTTCTAAAGAGTCCTAAGATTCAGGATTTAAGACTTAGGACTTAGTCCTAAAACTC
AGTACTCTGGGGAGTCCTGTGACTTTCTTAGAGTAATGCTAACTTTGTCGGATGCCTGCCTTTCAGCATCACAAACACAGCAATCTCCAACTCTC
TCTGTTATATGTACAGTCACTGTTTTCACAGCAAAAACATGGTATAATTTGACAAAGGACAGGGTACTTGGAATTAAAGCTGTCATTTAACATTTA
GGTCCTTTCAAATTCCTGTCATTACCCCACTCTTGGCTATATGAACTAGTACTGTTATATACATAGCAAAATAACTATGGGCGAGTAAAAGCAATT
AACAGAATTCTAGAATTCTCAGGGATGGGTGGGTTCTAGAGGAGGCAATTTTTTTTAGGTGTTCATAGATTAAAAAAAAGAAAACCCTTCAGAGT
AGGACTGAATTTTGTGCGCCTTGTTTTTAATATAACCTCATATATTTAAAGAAGTGTACACTATTTCAAATTCATTACAATTCTAGGAGATGAAAAC
ATCTAACATAGCTCATTAAAGTAAAATAAATTCTCCTGAAGGACGTCCAAGTCAATAGAGGTAAATGATCTCCTGTGACAGAAAAAATAAGTAAAT
AAAGAACATGAAAAAAAAACAAAAACAGAACAAGAAAATACTTGCAGTGGGCTTTCTAAGTACATCTGAAGATCAATTCAGATAACACCTGGGTG
CAGAGAGGGTCTGTATGGTCTCAAACAGAAAATCAAGGCAAAGGGCTGATGTGATGTGCAAAATCAACTATGAGAGATTCAGGCTTAGGGCTG
ACGGGACAATAGTGACCCCTGCTGGAGCCTATCTTNNNNNNNNNNNNNNNNNNNNNNNNNTCAAGAACCTAAGTTGGGGCCTTTGTAAGGATACT
TATAACTTCCAATTCCAAGAATAATTGAGTTTGAAAGTTGGGAGAAGGCTTGGAGAACATGGAGCATTCTCTCTTTACTTCATAACTGATGAAAC
AGGCTGTTAGCCCACAGAGTTTAAATGACTTGCTTTAGGTGGCAGAATGGGGATTACCAGGAAATAAAGACGCCTGCCCAGTTCTTGGGCCTG
TTAAAACAGGTGGAGTTTTCTGTCGCAACAAATAAAAGTACCTATAATACACACAATAAGGAGGCCAAGCAAAATCTCAAACTGATTTTAAAATAA
ACTGTTCACTTAATTTGGTTAACTAGGACTCAGTTTTGCTGAAAAAAGAGATTTCTTTAAAAAAATTTTTTTCCACTTTTTAAAATGGAAGTACTGG
GGGTTGAACGCAAGACCTCACGCAGGCTCAGCATGCGCTCTACCGCTGAGCTATCTGCAAACTCCTCTCCTGCCCAGGACAAATTTCCAGTCC
ATACTAACAAGCCATTTTTTCCCTTTCCTCTTTGGCCGTTAAAAGCTTAAACAACAGCATGTTAGAATCAAGTTTTTTTCTGGAATGCTGAGCTGG
CCTGTCCTTCACTAGGTTCCCCATGTTCCCCACCTCTGCTATTTTGATCTGTTTGAAATAGTTCTTTGGGCTTGAATATGCTTTTATGTCCTTCCC
ATTGTTGGCCTGTAAAGATCTCTTGATGTTGTACTAAGATAAAGGACAAAAATGAAATAACACATACAGGAATGCTTCTTATACTGGGATTTTCTG
GAAAACCTGACTCTGACTCTTACCCTCTGCTCAGTGCACTGGAGTCACCCATTTCAAGTGCTCTGTCTCTTCATCCTTCTTTCCTCAATCAACAT
CTCTCTCCACTCCATACATCAGAGTTCCCAAGACGCATCACGACCATGGGGTTCGTGCATTCATTCTCCTGTCACTGTACTAGAAGGCAAGATC
CTGGTCCCTATTGATATTCCTAGGTAGTTATGGAGACGTTGGCAAAGTCACTTTTCTCCTTCCACATTTCAGTTCTGTGATCTTTAAATAAGTACA
ATATTGGCTACCCTTCACAGTGTTTCAGTGAGGTTCAAATAAGATGTAATATTTGAAAGAGCTTTGAAAAATAGAAAAAAGTAAGTATGGGACAG
TTTTTGAGATACTGTTTTTATTATTTTCTTTGTAATTTTTTTTCTCTCCAACTGGTCAAGCAGGTAGCCAGATTTTTTGTTGATTTATTTGTTTGTGG
AATTTTTTGAATTAAGCTATCTGAGGTTTGAAAGTGCCTCATCTTTTACAGAAAAGATTATGCTCGGAGCTCCAGAGAAGGAAGGAAGAGGAGA
GTTTTAAGGAATGAGAACAAGACAGAAGTTCGGCTCATTCAGACTAGAAAGGTTACAGAGGTTTTTGCTTTCACACTGGAGAGCCTGCTCTGAG
GATGCCCCTGAGGATGGTGGCAAGACCTCAGGTTAGAAGACTTCATGACGTCGATATTAAAAGCTGACTGTCCACTTTGCATGGCAGAGAGAT
AGACAAAAGTACAGATCTACATCGATTCATGGGCAAGTGCTATTTGGCTAAGGAGTCGGGGCTTGAAGAAACAGGATTAGGAGATGGGTTAAAA
GGAGGTCTGAGAAAGAGTTATATGGATGGGCCTCTCAGAGAGGACAGAATGAGATTATTTGTGTTCTATATAGAAGCTTACTAAAAGGTATCCA
CTGGAAAAGATTGTCAATAGTCAAGTAAACACTATGACCACTGTGAATGTCACTTAGCTGTCTTCCCCAGCCACCCTGGTGCTTGACAGTGGGC
CTGGCAAGAATGTGGGACTGGGAACATAGGTTCTCTTCACCAAGGCTGATCTGGCTACTTTCCCTGATGAGTGCCTCTCTCATCAACATCAGAT
ATCCACAATGAGCTCCTAAAGTGGCATCAATTCCAGGACTTGCCAGTCACCTGATGGCAGATGGATTACACTGAACCTCTGTCATCATGGGGGA
AAGACACTTAACCTGGATTTGGATTTGCCTTCTTTGCCTATGATGTTTCTGCTAGCACCACTATCCATAGACTTAGGGACTGCTTCATTCACTCTT
CTGGAATCCCATAAAATATTGCTATAGATGAAGGAATTCCATTAAAAGAATAAGCATTGAAGACATGGTTTCATGTCCTTAGGAAGAAGCTGGCT
TGATAGAATGATGGAATGAACTATTGACAACTCACTTATGTGGCCACTTGTGGGACGGGGGAGCTGTCCCACTAGTTATACTGTATTCTCTGAA
CCAGCACCAATACATAATGCTGTTTCTCCCACAGCCAGAACACTTAGATCCAGGAACGAAAATGCTCAAGTGGTGGTGGTCTCTTCATTATAATT
GCTAATGACCTACTCACATACTTTTATTTCTAGCCCCACAATGTTATGCTCTTAGTCCCCAAGGGAGGAACACTTTCTCTAGGAAACACCGGAAG

GGTTCCACTGAATGGAGTGGAGACTGCCAAATCAGCAATGTCAATTCTTCATGCCACTGAACCAGTAAGCAAAGAAGGGACTCACTGTATTGGC
TGGGGCAGTTGATCCTAATTATAAAATAGAAAAGGTCAGTATACACAGCAAAGGGGAGGAGGACTATGTCTGAAAGCCTGGGGATTTTCTGGG
GAACTCTGAGTACTCCCATGTCCATGGTAAAATCTATTGGGAAATTAGAACAACCCAAGCCAAGCAAGAACCACTAATGGTTCAGCTTTTTCAGG
AATGAAAGTTTAACTAATAAATAATCACAACCAGTGAAACTATTATCTGAAGACGCAGGGAGCAAAGGAAGGTTAGTAGAAGAAGTGAGGTGCA
AATACCAACTATGGCCCATAAACCTTGTAGAAATGAGAACTGTAATCATCATGCATAGTTACCGATTTTTAAATTTTCCTTTTCATCCCTGAGGTC
TTCAGGATCTGAATCTAATTATGGAATAGGCCTGGGTCTGGAAGCAATGAGACCCAGTGGGTTTGACATTGAGGAGAGTCCCTATCTCTATTCA
GGAATCTGAATCAGATTCCTCTGATAAGTCAACTGCCTCAGGTTAATATTAAAACAGGTTACCAGGCAGAGAGAGACAAAGATTGTCAGACAGA
GGAGGGAGGTTTGTTTCTATTGACAACTGACTTAGTGGGATTTGGGGATTCAAGATAGCTGGATTCACTGGAACCTATCCAAGTGTTTCCATTCT
CAGGATCCCAGTCTCCTAATCAATGCCTAGAAAGCCAGTGCTGTAATGCATAAAGCCACAGAATCAAATTTAGGTTTGTTTTTTGGGTGCCTCAG
CCCCACTGGTACAAGAAATAAGACATTTTTTTAGGCAGTCAAGGAAATGTTCTGATTCTCCAACAGTTCCCTGAGTTAAACATCTAAATCTCTGG
GTTTGTCATTTTTTTCTGGCAAGCTTTCCAGTGCAGTCAGAACCACCCCACATCCCTTATAATCTCCACCATGATACTTTACTAACAGCCACTACA
TTTCCTAAATTCTTATCATTGAATGGTACCTGATTCCAGAAAACAATGGGATCATTTAAGGAACATTTTGTCACTGCATGGTATGGACTACCAGTG
TCCTGGTTTCCACTGACAGTAAGACCACTACTGATTTCAAATCTAATCTGTGTTTATGCTCATGTTCACTACGTGCTCATATCATATGCTAAGTTG
CTTGGGAGGAAAGGGGATACAAATGAAGGAAACAAATGGGCCCTGACCTCAAGTATGTTGTGATCTTATAATCAGTAGCTAAGAGAGTTCTGCT
GAACTCAGAGAGCAAACAGCATTCTCTGAGCTGCCTCATTCCCTCTTCACTAGAGGACCAGTGGGCACTTCTATTTGTTAAGCTGAGATAGAGA
GGGAAGTAGTAGTTTCTTGGAGTCCCAAGTAGTGACCCCATATTTCTCACAGAAACAGTGTTATAAATGATGGTTACATTTCCACACTAATTCCT
CCAAGCCTATTATAGGCCCCCCAAAGTTACTGAAATACAGTGCTATTTGTATATAAAATACAGTATATTTCTGGCAAAATTATGAATAAGCTTTAA
GTGAATGACCAGGAAACCTAATTACCATCTAGATATTGTTTCTATTGTAAAACCTATTTCAGATCTAATATAAGACATTAAAAATATTCTTCCACTT
TCCCTCCCACCTAAGCACATATACTGTACCCTAGCATCTGTGGCTAAAAGGGAATCCTCTCTTCTTGGTCACCTCTCAACAACACCATGTGGTCT
GGCAGTAAAGGAATCATTCCTGGTGGCTTTGGGAATGTTTGGCAGCAGGGACGGGAGGCAGAAGAGGGTCTTCTCAATGCACAGTGGGCAGG
CTGACCCCAATAGTAGGACACCAGAGACAGCATTCACTCCATCTGTTTCAAGATAGAATCCTCTTCTTAGGGACACCTTTACTTCTTTCAGAATC
AATCCTTTACAAACTGCTAAATAACTTGCCAAAGGCCACTCAGCAAGAAAGTGTCAAAGCTAAGGTTCAAAGTTAGGTCTTTCCTACTCCGAAGC
CCATGTACCTTCCTCTATATTCTTCAGTACTTTGTATAAAAATATAATCACTTTTAAAATAGCCCACAGTGCCATTTCCCACCAGTGTATCCTGATA
AACGAATGTGCCTTACCTCAAGCTTCCTACTTACTTTTGATAGCTATGGTCTCAAACACATTAAAATCACCTCGAAGCTTTAAAATTACCTGAGCT
CTACTCCAGATCAATGAAATTAGACTCTCTGAGGGTAGGGGTTTTTTTTAAGTTCCCCATTTGATTCCAGTGCACGTCCAGGGCTGAGAACCACT
GGTAATCAAATCTAAGACAAAGGCTTGAAGTTGACAGCTATAATATGCTGCCTGTATAAGCAAGGCACTGAGTGTCATATTAAAGAAAATACATA
CAGATTAAAGAGAATGCATCTGAGCATTTCACAGTGTGTCTGGCATATGGTAGAAACAAAATAAATGGTAGCATTAAAACTGGACACGCTATAGT
GAAAGGAAAGTGGGGTTGGAGTTGGATAGACCTGATTTCAGATCCCAGTGTTACTATTTTTAGTCTTTCCTAAGTTAAATATGCTGTGGCTTAGT
TTCCTAGTTTGTACAATAGTTACAACAAATCCTATCTTATAGAATTGCTGTGAGAATTAAATAAAATGTTTGTGAAACACCTGGCACATTCCTTCTT
TTGTTTGTAACCCACCTATGCTAATAAAACAGCCACTGAAAACAGTTACAGAATATTCCTGACATTTAGAAATGTTCTGTTATAGGTGTGGAAATG
TATAAGTTCTGTATTTAGTATAATAAATTCAGATAACAGTGTAGATTCTATTTATTCTATACTATTTCCTACTCTACTGTAGAATAAAGCATCAAGAT
TGGTGACCTGGTATGATAAAGAGGGTTCTTGAAGAGAGGGCTGGTGGGTAATGAGCTGGGACAGAAATGCAGGCATGTGGACTCTTCCATCCA
TGTTATGCTGTTGCCCGGACCTGACCCCAGTGCAGATCAAAGTGGCTCAGAACACAGTGGGAAGCCAGGTTAGACTGTCACCACTGCCTTCAT
GAAACTGAGTCTAGAACAGCCATTAGCCGCTTACGTCTCAGTGCTGTCAGTGCCTGATGTAGGGGATTGCAATCAAGATGGAATGTGTCAGCA
GTGTTCAAACAAACATCACTGCTCCATCTTGGGAGCAAAGCCTATAAATAGTAGATCTGGATATGGCTCACACCAGACCCTTGTGTAATTATCTG
TCTTCACAGGATGTGCCTGGCACAAAGCGCTAAGAAATCTGGAACACTGCCTGACATATACAAGGTACTCAATAAGTGTTCCTTGAATTAAACTG
ACCTGTGTCCTCTGCCTGGAACACTTTCCTGACTGGTACTAGATGTTAACATTTACTGGGTCTTAACGACTGCCTGGCCCTGTACTAGGCACTTT
ATATAGAGATCATCTCATTCATCTGGCAAACTCCTGCCCATCCTTCAAGATCTAGCTCAAATGTCACCTATTCGGTTAAACCTTCTTGACTCCCCA
AGGCAGAGTCCTCCGCGCTTCTGCACTGACAGCTCTTTGCTCAGACTTCAGAAAAGCACTTACCACCTTCTACTTTAACATATCTGTTTACAAGT
CTGTCTCCCCCATTGCACTGTGAGGTCATCAGGGACATCATGTCTTTATTACTTTTTTCTTCATTAGTGGTACTTAATGCAGTATCTGATGAAATG
AATTTTCAAAACTGCTTGCAATTCTGCTGACTAAATGATGTTACACTGAATGCTTTTACTTATAAAACTACATCCTTTTCCACATAGAAGCCAGCTA
ATATTTTTATGTAACTGAGGAAGGACAATTTTTATATAACTGAGGAAGAATGAATCCTCTGTCTCAGAATCTGAACCAGTGAAGAGTGGGATATG
AGCTCTATCTGCACACACCAACTGTGAGAATCAACATCTCCCGGGAAGTGCCTAATACGCTTCCACTAGCACCTACCTTGGGACCAAATTGGTG
TTCTCAGGGTATCTGTAGTTCCTGGTAACTGTCTTCTGGCAGGACCTATGGTCTGCAACAAAGAAAAAGAAACATGAGAGGATTTATACATAAAT
ACAAACACACCGCATTTTTCAATAAAGCATTATCACCTGAAATCTTAAACTCTAATTATAAAATGGAGAAATAATACTTGCCTTATATACTTAGTGA
GGATTAAATGAGATAATGTATATGAAGTGCCTGATATAGTGACTAATTTATTGCAAACTAAAAAAAAAAAAAAATGTGGTAGCTATTTTATTATCCCA
CAACCAGGCAATACTCCACTAAACTTCCAGCTTTCTAACTCCTTTATTTGCAATACATAGTGTGGAGCTGGGAGCTTATTGAGAAAATTTCCTTG
ACTCCCAGGTGTCGATGTGAGCAAGTGGTTGAATAATGGTGCCATTTAGTGAAACGGAGAAGACTGGGTGGGGAGGAGAGGACTGGTGGCAA
GTCAAGAGTTCTGTTTTGGCCATGGTATGTTGGAAATTCGTATTAACTACCCCAAAAAGGATGTAAAGTAGACTGCTGAATACGTCTGGAGCAAA
CAGCAAATATTTAATATTAAAGGTCATAGGACTGGATGAAATATCTCTGGAGAAAGCATACATATAATAAATGTCATTTTTTTTCATAGTTATAAAAC
CTATTCTGTTCTAAAATTTTTTTCTGCTTAGTATATTATAATCATCTTTCTATGTCAGTATATACTTACATTTTGCATGGAGGTAGATAAATAATTGA
TTTAATTTATTTCTAATTGTTGGACAAGTAGGGTATTTGCAATTTTTCAATGTTATAAATTGTATAAATACTTTTGAGACTTTGTGCAAGTTGATCTG
TGGAAAAAATTCTAGAAAATAGAACTTCTGGATCAAAGCGCATAGCATATCTAAAATTTTGAAAGAGAATGCCAAAATGCCCTACAAACTGGATA
AAAATTAAAATCTACACTCCCACCAAGAGGGCAAGAGTGGGCTCTTTTCCAATGTTTATTCTAACACTTGAGTGAGAAATGGTATTTCACTGATA
CTTTAATCTTATTCTTACTTATGAGGGAGGTTGAATATCTTTTTATTTATTGGAAATAGATAAAATTTATTTTTGGTTTATTGGAAATTTGTATTTCTT
TTGTCATTTTTCTACTGGGTTGTTAACTTTTTTCTTATTCTTAAGGGTTCTGGAAGGTAAGACAAATTTAGCCTTTTTCCTGTCATAAGCATTTCAT
TTCCTCATCTTTGTTATGTTTGACTTCCAAGGAGACTTCTACCTCGGGCTACATTTGATTATCTGGTACTGTACCTGCCTTTCTGCTGTAAACGAT
TAGAAAACAGGGTAGAATATGCAAAACAACTGCTTCAGAACTGGACACAGACAGAATAAGACTGTGATCCCTAAGAGAAGGGAAACAAGTCAAT
GAGCCATAGAACTGCCCTAGAGGCACATTTTGGATCACAGTGCAGAGAAGGGAAAGTCAGGCAGAGCACAATAATCTTGCTAAGTTGAGGAAC
CAAAGATCAGAAATCTAGGAGGTCAAGGACACTGGGTATTGTGGGATAGGGTGCTGGAGAGGACGGAGCTATTTGAAGAAAAACTCCAAAAAT

CTGTGCAGGGTTCCCTTGAGTCTGAAATAGGCTGCAAATGAATAGAGTGAAATTTTATGGGCCAGGCAATGAACACCGGGGGAACTGTAAGTG
GAACACTTCTTCAAGGTCACAATGGGCTCCAAGATCTGACCAGCCAGAGTAAAAGACATGTTGAGTATCCAGGGCATTAATTAACCTTAGAAAG
GTCATGTGTTCATAATTAGATTAAACTAGTCCTGGACTAAAGGCTACTGTAGACCCACCCCAGCAAACTTGAAAGGCAGCTTCAAAAACATCAAG
CTGATCTACAAGAAACTATCTGCTTGTCTAAACCAATTTTAATATTCGTTAAAGGAAGACAATGAAATTCAGACACTTTATAACGTAATGTTTATAA
TTCCCTCCACCCAATAAAAAAGTTAGCAGACATATAAATAAACAAGAAAATGTGACTCAAATCAGGGGGAAAAAACAGTCAATGTAAACAGACAC
ACAAAAAGACACTGATGATGGAATTTGCAGGCAAGGATTTTAAAAGTCATTAAAAATATGCTCTAAGCTTTAAAAGAAAACATGAACATAATAAGG
AGAGACATGGAAGATATAAAAGAACCAAATGGATTTTCTAGGGATGAAGTACAATATCTGGGATGAAAATTTCACTTGATGAGATTAAGAGATGA
TTAGATACTGCAGAAAAAAATCAATGAACCAGAAAAATAGGAAATAGCTGTCAAAACTGAAACACACAGAGATAAAGAGAATGAAAAATATTAAT
ACAGCCTCTGTGACCTGTGGGACAACATCAAATTGTCTTATTAATATATGTGCAATTACAGTCCCAGAAAGAGAGGAGGAAGAAAAAATATTTGA
ATAAATCATAGTTAAACTTTTCTCAAAATTGATGAAAACTATAAACCCCCAAATCAAGGGAAGCCAATAAACCACAAGCAGGATTAACACAAAGAA
AACCACACCAAGGCACATCATCTTAAAATTGCTGAGAACCAATGATAAAGTCTTAAAAGTAGTCAAAGAAAGACATACCACACATAAGAACAATG
GTAAGTATTACTGCTAATGTCCAGAACAATGTAATATAGAGGACAATAAAATGATATCTTTAAAATGCTTAAAGGAAAATGATGATAACAGAGAAT
TTTATATCCAGTGAAAATATCCCTCAAAAATGACATACTTCTAAATAATCAATAGGTCAAAGAAATAAGCACAAGAAAAATTAGAAAATATTTTATA
TTGTGCATGACTTTAATGTTTACTTTGCTATGCTGAAATTTGAAAGTTTTATCTGGACATGCTTATCAGGCTACAATAGAAAAGACTGATAAATGT
GCCTAGTTAATTTTAAATTTCATCATGGCAAAGAAAACCTCAAACACATGGACAGACAAGTGCTTAGAATGTATGCTACCCACTCCAAAACTTAC
ATCATGCTATTCAAAACACACTCACATTTCCTTCTCATACTTTTATGCTTTCATTTTTGAGATCTAAATTTTTGATGAATACAAAATTGTCCCTGTTT
TCATGGAGCTTACAGTCTAGTGGCCAAGGCTCCCTGGGCTATTTCTGTTGCAATACCAATACACAAGCCATCTATTTAATGGCTTAACTGAAAAT
GACACTTTTATCACTTATGTGATTTTCTTACATGCTTGTGTCTATTTTGACTCTTCTTTCTGTTTTACTTCTCTTTTTATTATTCTATTAATACCATGC
TATTTTAAACTACTAAGCCTTAAAATGTTTTCACATCCATTAATGCCAGTCCCCTCTCATTCCTCTTCCTTATCAAAATTTACCTACCTATTCTGAA
ATATTTGTTACATCTAAAAGATCGATTGAGGTTTTAATTGGAAATTAGGTTGCATTTATAGACTAAAGAGAATGACATTTATACATCATTCTTTCTA
TTCAAGAAAAGAATATGTCTTTCCATTTATTATTTGAGTTTTAATATTTTCTTCATATGGAGCAGCGGGCTTTTTCCCTGGTTGTACATTACAATCA
CTTAGATGGTTTTTAAAAATACAGATGTTTAGGCCCTGTTACCAAAGGCTTAACTGGTTTGGTGTGGAGCATAGGAGTTGTGTATTTATTAAGAG
CTTAACCCCATTCACAGTTAACAACTACCAGTGCAGGGCCTTGCATACTTATAAGCTGAGTTCTTCATAGCTGTTTTTATTTGCTTTGGCTTAGTT
TTGGCTGTTATTTTGAATGGCATCTTTTTTGTTCATTATATTTTAAAACTGATTACTTTTTACATAAAGAAAACCTATGATTGCAGAGTGCACTAATT
TTGTAACATGTCACCTTACTGAATTTGTTTCATATGTTTTTCAGTCAGTTCTTTATATTTTTATAGGCATTAAATTATACCACCTGCAAAAATGACAA
CTGTTCCTTTTCTTATATTTATGCCTCCTTCCACTATCTTGTTCATATTCCATAAGAATAAAAGTAATAGTTATGGTTGAGTGTATCCTTAACATATT
TCTGATTTTAATGAGAATATGTATAGTGTTTCATCATTAAGCATAATGCTGATTTATCACTTTAAGAAAATATCCATCTACTCATATTTTACTGAAG
GTTGTTTTTTTTTTTTAACAAGGAATGGTCCTAACTAAAGAATTCCTATTTAAATTCTTTCAAGTGTTTTACAGGACTATATTAAGATGATCATGTTT
TCCTCCTCCTCTTACCTATTAATATGTTGAACGAACCATGTTTGTGCTGCTGGAATGACTATGTTTCATTTCAGTATAATGTTCTTTCAATGTACTA
CAGAACTCTACTTCTAATATTTAATTTAAAAATTCAGGTGGGTAGTCTTTTCTTGCTTTTATTGTCATGTGTTAGCATCAGTAGCTCAATTTTTTTTT
GTAAGCTTCCTGTATTAATTTTTATTCTTTAATTTATTAAAATTCTAAGACAGTGGCTTTCAGAGTATTTTTATTGTGACCCAGAGTAAGAAAGGCA
GCTTTACTGGAGACATGGAAGAAGAGTCTGTTTCTTTTCCAGTATCTCTGTGCTTCCTTCCTCCCCGTGGGATGAAGACATAACTCACCTCCCCT
GCCTTTAACATGTCCAACTCATCACAAATCCCCAGTGAGTAGCACCTGAGTTACTCAAGTTTTGCTGTTATACTTTTAGACTTTAAAAAAATTTAG
TTTTCAAAATAGATAATACATTCTCATGGGTCAAAACCAGAACATACAAAAAGTTATATTCACCTAACTCATGTTTCTATTAACCCAGTTCCTCCCC
TCCAGATGTAGCTACTCTTCTTAATTGCTTACATATACTTAGTTCCAGAGTTGTTTAACGCACATACAAGCAAAGTTCAAATATGTGTTCTTATATT
TCTCTCTTTACCAGAGCATAACATAATTTTTTGATCTCTGTGTGTGTGTATTTTTTGTTTTTGTCTTGTTTTGTTTTTAATTAATATGTTTTGGAAACT
TTTCCTTACCTGTTCATAATTTGCTTTCTCATTCTTTTGTACAGTGGCACAGTATTCCAGTATGTGAATGCATCACAGTTTTTACTTAATTAGACCT
TTATTGATAGAAAAAAAGAGAAAGAAATGCAGCTTTAAAAGGAGATCTAGCATAAGTATACGCACTGTAACAAGTTTCACATAATACTTGCTCTAT
GAGTATTCACTCCGTACTATTCTATTTCTTTAAAAAAATGCTCATCTCAAAACACTATTTTGAAAAATACTGTATGTTATTTGAAGATTTGGAAGAA
TTATCTATAAACTGATGTTTTTCAGAAAGTAGTTCTCTAGCAATTATTCCATTTTCTTTCTTGCTTATGTTTTTTCTAGACAATCTATACCATCTTAA
TTTCACACAGGTCTTCAAATTTATTTGCATAATACTTTCGCAGTACACTTTTTAAATTCTTTAATTCTTTCACAGGGTCTGTAAATCGTTTCTCTCTC
TCGTGCCTTCTTTATGTTAGTCAGCTTAGCTAGTATATCGGGATTTTTATTTTCTCCTTAAGTAAAGAAATTAGAGATTTATATTATCAATTCTATTG
TTTCTGATTTCTGATTTTACCTTTTTATTTCTCTTCTGCTTTCTGAGGTTTTTTGTTTGTTTGTTTGTTTTGTTTGTTTTCTTTCTTTTTTTTTTTTCTTT
TTTTTGCTTTCTGAGGTTTAACTTGTCTGTTTTCTACTTTTTTGAGTTAAATAGTATGTTATTTTTCTTCTTTCTTGTACTTGCATTTGAAACCATGA
CTATCTGATGAACATAGCAGTAGCTGTATCCTATAGGTTAGGTGTGTAATGTTTCAAATTACTTTGTACACATTTTGCAACATCAATTTGATTTCCT
CTTTGTAAAAAGGTTGTTTTAAATAGTTTTTATTTTTCCAAGTAGGGTAGATGTTAACTTTTATTTTGGTCATTCATTTTTTTGTTGAATTGCATGGTT
ACCAGAGAATGTGGTCTGGATTATTTCTAATTTTTGGAATTAATTGAGGGATCTTTTGTGTAGGGGGGCTAATAATATAGTCAATTTGGTAAATGT
TTCATGAGCTTTTAAAAATAAGATGATTTTCAGTTAACAAGATACCTATTTCATCTATTTCATCTACCAAGTCTATTTCATTAACCTCATGGACTTCA
TTGTTCAAATATATCCTTACCAACTTTTGATGACTTGATCCATGAAATGAATTAAAGTAGGGTACTACTATTTTGTTTCTAATCATTTTTCTTTGTAG
TTCTGCGTTTTTTCTTTATGTGTTTTATTGTATTATGTAGTGCTTTAAGATTTATGAAAGGTGTATCCTCTCTGTAGATCTTTTACCATCATAAAATT
TCCTCCTTCATCTTGTATTATACTTTTTGCCTTAAATTTCACCCTATCTGAGTGATATCATGACCCTAACTTTCTTTTTGTTCACCTTTGCCTAGCA
TATCTTTTCCCACTTTTTTCTCTCTAGTTTTCTAGTCATTTTGCTTTAGATCTACCTCTGTGTACATCATATATGCCTATCCCTCACTTTACAAAAGT
TAAATTTATAAATTTTCAATTTTGCAAAAAGTTCTAATACAAATTAATATTAGTAATATAAATATTGTTAGGTACTACTGGTACAATTGTTATTAAATA
CTGCCAATGATTTTAACTTTTACCTGGCATCAGGCACTGTTCTTGATGCCTTACATTATTTTCTCATTTAGTCCTCACAACTTCACCAATATTTCCA
TTCTGCAGATGAGGAAATGAACGTTTCCTAATTTGTCCAAAGTCAGGCAGCTCATAGAGAGTGGCTAGCAAAAGAGTTTATTTATTCATGGGTTT
TGGAGAGGGGAGTCTTTATAATTTTGTTTCTAGTCACCCATTTGTTCTCCTCTTTTCACTCGTAAGTCTCCAGTGTACATACCATTTCAGCTACAC
CCAGGAAACTGTTGGTTTGCCAAATAGTCCAACTTCTTTCCATAATTTTGGACTTTCTCAAGTGCTGCTTTAACTATTACCCACTCTTCTCTAGGG
GCAGCACGGTGTTGTATATAAATATACTCAATGCCTACCCACAACCAGTGAGTGTTAAACGAATATTTCATGAAACGAAATTCTAAGTAGAGTCT
TTATCAAATTTTATCAAGTATTTAAATTTCAGCTGTTCTCTCATCAGATTTCTCCTCTGAGTCTAACTGAACATGTAAAATATTAGACTTATTCATAT
TTAAAATTAAGGTAGAAAATTTGATTATATTACAGTGGATTTAAACTTACTGTATCATGCAAAAATGTCTAATATTTAACACATATTTTGAGTCTTAA

TAATAGTAATGCCTTACTTATGGATTTTATTCAATAAATGTATGTTATAAAAAAGAGAGAAGATACATAAAACTAAATTCAAGGTTTAATCTGAAGT
CCTTAAGACCTTAGAAAAATTGGTAGTATATAGGTGATGTAGTTCTTAGAGTTCCCAGCAGGGGCCAGAATGTTCATGTTAAATCTAATTAAATG
AAAATGTTTTTACTACAATTCAAGACTGTACTCATTTTTAAACCAATATGAAAGTCAGATAGAACTCTTTATAATTAAAATTACTAGAAACTTCTGAC
TTATTAATTTAAATTTTCAAGAATGTAAATATATAATTCAAAATATCATAAAATAAGCTTAACAGAAATAGTAATGTTTTGTAACAAAAGGCTGACCC
CCAATATCTGAGCAAATGGGTATCCAAAGTACGTTCAGTCAGTAGCTACTAAAACCCCTACACTGATTGCCAAACTCAGCTCATTGGGTCTTTCA
GTTGCCAAGCCAGACGATGGACTGGGTCCAAGAACAAAGGGCACCACTATGGCATTGAGCCAGGAAATTGAGTACTGACCTTATTTGTTAATTT
TAGAACATTAAAAAAAAAAATTCTTAACTCAATATAAAAGAAGTACTGGTGATTCAAATGTATTTCAATGCAAATTTCCTTTGTAAATCAAATAAACT
GTTTTTAATTTAATTCTAATTTAAGTCACGTAACTTCTCTCTGGGCCTGTTTCTCCATCTGTTCATGAGTGGGTTAGATATGATAATCTTAAAATTC
TGATGTTCCTAAAATTCAATTTTTTTTCCCTTAGGCAAAAAGCATCATTTTTACCCTGTCCCTAGTTCTTGGCATAGTCCCTGTCACAAACTAGGT
GCTTAGTAAGTGTTTAATGAGCAAATAAATGAATAAACAAAACAAATGAATGCATTTTTAGAGAGCTCTTTCTAGGAATTTACAAAAGAATTTGGA
AATCAGATGCCTGTGAAAGCCAGTCTTCTGTGAATTATGAAAAAAAATGCTTTAGGCAGTTCCAAGGAAATAAGAAAATGTTTTCTGAATACTCTT
GGGAAGAACTGAATTCTTCTATGGCTATCACTACTTTCTAGTCAGTAACTGAGTAAAGAGGGAGTTAATGTTTAAAAAGGATTTTATACTTTAAAT
TTAAGATCTAAATCTAAAAAAAAATTTTTTAATTATTTGAAGAAACATCCTCTGTATTGAGTCTATGGTTTCCCAAGCTCCGCCAAAACAAAATCCT
GAAAACTACAAGGTTGTTCTCTTGGGGAGTGGTTGTTTACCAGAAGGCTTAATTCTTAATAATAAGGGTAAAGCTTTCAGAACTAAAGATATGCC
TCAGCCAATTCAATCTACATTGTCATCTGCCCTGGAAACCATGTACTTGTTCACTTGTTCTAAAGTAAATGAAACCATCATGAAGGAAATCAATTA
TCAAAAATCACAACAATGAAACTTCTTATACTCGGTTTCTAATTTGTAAATTTTAAAAATATTACTTCATTAAATCTGGGGGAAAATGTATAACTGC
AGGCATAAGTTTTGTGCCTTCTTAATCCAAGGAGGTGGTCAGTAGTATGGAAGCTTAGACCAAATGTGAGGATGTGGCTGTAGCAAATTTGAGA
GCCTAACTAGAGACTGCTGAAACATAGGTCAGGGAATCTGAAACATCCCCACCTGCTATTACACCACATAAACAAAAAGAAAATTTAAGTTCAGT
TTAAAAAGGGCTAAATTAGGGAATCACAAACTTTTTCCAAACATCAGCCACTGCCTTTGGGCAAAGGATTTAAGTAGCTCTGCTTATTTGCTAGT
AATGGAATAAAGACATTATTTGAGATAGATTCTCAAAACTTGAATAATGTCAAGATTCTTTTCCAAGGAAAAACAATTTTTTGAGAACCACCTTAAA
TAAAAGTCAACCCCTATCACTTTAGATGCCACTTGAAGCCAACTTGATTATAGTAAAAAATTAAACTGTAAACACTGTTTAACAACACTATAGACA
TTATATTTGCAGACACTGTAGAAAATAAAGCATAAAAATTGAAGCATCATTCAGATGTAAGGTGGTTGGTTACCTGGATGCTCCAAAATATGCAT
CAAGTACTAAGTTTTGTGAAATTACTTCTGGAACTCACAGACACCCTTGGGGAGCTAAGGAACTTAGCTGTAGAGAGCATTCTGGGAAAACAGG
TTTACTTCTGGTTGACCTGAGCTGTCTCCTCACAGAGCTATGCCTTGTTTTAAACTGAGGCAATAAACTCCATCCCAGTTTTAGGGGAAAGTGCC
TTCCTGCTGGTATGAGGAGAAAGCAGGGAGCGAAGGAGTACTTTACGAAAAAGAAAATGTAGTAGAAGACTATAACATGTATCATCAGTTCACA
GTTCCACCCACTGGGTCAAGCAAAGAAGACTGTCCAGCCTTGGCGTTGCCTAAACATGCATGCGCGTGCGCGCGCGCGCGCACACACACA
CACAGCCTTTTGAGATTTCTTTTCTCTGGCTACCTTTGACGCTCCTTAAACTCAGTTTGCCTTAAATAGGACTGTGTTAGGTAAGGGCTCTGTAG
TCTTAAACAGTGATGTCACAGCCAGCATGGTCACTCCCTAGCAAGGTCTTGTTAAGAGCCACACAGTAGTCATAGATCATTTAAATGCAAAAGCT
CCACTGGGATGTTCAAGTGCTCAGTGCGCTACCACAGAGCTGTGAAAAGGGGCGAGGACAGAGGAACGTACCAGTACTGGTACTCCATGCCA
GTACAAGTAGGAGGGAATCCAATAGAAATTCTACGAATCATAGTCAGAGCAAACAAAAAACACTACAGAAGCACTTAGAAAGTCTATAAATATAA
ACATAGCAATATAAATACCTACTGAAAAATTTGCAAGTTGAATTTTCTTTTAATTAAAAGATGTTCATTCATTTATTGGTTCAATTATCAGTAAACAC
CCTTTTGGCAACCTAATTATGTGCTAGGTATTAGACTTGAAGCTGAAGAGACAAAAATGAGCAAAATAATTCCGTTTTTAAGGAGCTCGTAGTCT
AATGGGGGAGGGAGGAGTAGAGATGGGAAGATAAGTAAGGACACAAAAGCAAGCATTTGAGAATTATAATAATCTATATAATTTTAACACTAAGT
CTGGATCTAGTATTTTTTTCAGTGGAAAAATGAGGAAGCCAAATTACAACTTAATTTTTATTCTTTTTGTTTCAAATGTATAATGAATAAACTAGAG
TTCCCACTTTGGAAAGGAAGGAGGAGAGAAATCACATTTTGAACTCTTCCTGAATTAATCCTACCTCTGAAGGACAGTGGAATGCAGAAATTTTG
TTAACTGGGACATTATCATAACTCACTGGCATAATATAAATGTTATCATCCCAAGACTGACCTGAAAACTCCCTAGATAAATCCCTCAAGACCAG
ATTTCAGTCTATACCCCAAAACTGACAAATGTTGTATAAATGTACTCTAAATAACCTCCAAATTAGTAGTTGCTGATTATAATACAGTAATGTTTAT
ATTTCTGCTCTTGGCAATAATTATGTATCAGTAGGTAAATAAGACCATATTTGATGTCTCATTAAAACCAGAGGTATTAGGAGGAGGGGACTTCA
AGCTCAAAAAAGTTCAATCGAAGCCGTGTAAAAATTATTACTTTGGGGCACTGTTCTCAACGTGTGATCTGGGGAATCCCTGGAGTGAAGGCCC
TTTCAGTCTGTGGAGTAAAAACTATTTTCAGAGGGGTGGGTACAGCCCAGTGGAGAGAACATGCTTAGCGTGTACAAGCACGCACGAGGTCCT
GAGTTCAATCCCCAGTACCTCCATTAAAAAAAAAAAAAATTAAAAATGAATAAAAATAGAAACTATTTTCATAATAAAGTTAAGATTTTTTTGCTACT
CTCATTCTTTCTTATGCGTATAGTGGGGCTCTCCAGAGGTTCTGGGATATGTGATGACATCATCACTCTGTCAGCCAATAGAATATAGATGTGCA
TATGCCTTATTCTAAAATTTCTCATTGTTAATTTATAATGTGTTAAATATTGATAGATAGATATGACCCACACAAACAAAAGCTTTTGAGGATCCTA
AATAACTTTTAAGCAGAAAGGAAATCCTGAGACCAAAAGGTTTGAGAACTGCTTTAGGGGATACACCTCAAAAGAAAGGGTATACCTCATCAAAT
TTTCTCCTGTTCTACATGAAGAAATTAGACAGTAGGTTTCATGAAGACAGTGGCTGTTTCTGTCTGGCTCATTGTTTTCTCCTCAGAATCTAGCAC
AGTGCCTGGCATGTAATAGGCATTAGAATATTTCATAAATAACAAATGAATTAATACAGTTGACACTCAAAACAGTGCAGAGGTTAGGAGTGCCA
ACTGTCCTTGGAGTCAAAAATCCGTATATAACTTTATAGTTGGCTCTCTGTATCCAAGCTTCTGCATCTGAGGATTTAACCAATAGCAGATGGTG
TCCTACTGTTGTATGCATTTAGTGAAAAAAATGTGTATATACATGGACCCATGCAGGTTAATCCCGTGTTGTCCAAGGGTCCACTGTACATACAG
GAATACATTGCTATATGAAGGCTTTGCTAACAGAATTATTCAAGGTCACATAAAATGTCAGCAACAAACCCAGAACTATATTCCAGATCTCCTGTC
TTCTATTCCTCTACTGTTGCTCCCTGGCTTAAGGATACTTTTGTTTTTGTTACATTTTATTCTGTAACCTATCGAAAGCATGGATAATCGGATGTCA
CTGATGTGCGGTGATGACCTTCTCATGCTGTTATGTGAGTTGAGCACCACACAGTTTTTTTCTGGTCTTGAATGTAATCAGAACAGTGTGATACC
AAAGTGCTGTCATTATAAGGAAATGGCAAGAGTGATCAGATAAAATATAACTTTCTGGTTTTTCCCACAATAGAGTTGAATTTTTTATGATTCATTC
TTATTCTAGTTCCCAGTCTCCTCCTCATTTCTTTGGCCAGATCATTAAACTTCTGGTAAGCTTTTACATTAATGCACTCAGTATTCCCAGACCACA
AAGAGTAAAACCATAAATGTTTTTAGGGAGATATGGTTTATACTCTCAAAGTTGTTTTGGAACCCCTCTGCAACCCAGTATTTGTAAGAGAGATG
AGAGAAATTTGCTTGCTACTTTACTTATGCTTACAGAAATATAAGGCACACCCAGTACAAAGAAAAAAAGGGGGAAAGAAAAATACAAGTCTTGT
GAAGAGCTTCACTGGCCACCTAAATTCTGACTCCCGCGAGAGACTGCTTTCAAAGATGGTAAACCTAAGACAAAGCCAAGTCGGCAGACCATG
CCCTCCCAGCCAACTAGAAGGAATTCTCTACTCACTCATCCTAATGACACAGTCCTGTTTTTAGGAAAGCAGCCAAAGTGAGGCCCCCAGCAGT
CAGAGCACAGTGAAGGTTCCTACTGCTGGTGAACCTTTCTGCTCTGGGGCCTTGGGAAGGTTGCAATCACCAAAACCTGAGAAGGAATATATG
TTCTTGAAACATCTAGTGCACCTCTTAACTCATTCAATACTTACCTCTGGAGAAGTTGATTAGATACAGTTGATTCTCATTATTCACAGTAGTTAG
GTTTTATCAAGTCACTGCCAACACTGAATGAATGATTACTGAACCACTGCTCCTAAGGTATACACAAGGTTAGGTTCCTGTGAGCCTCTGGTTAC

ATTTCATCAACCAATCAATATATTATCTTGCTTTATCTATCTATCTATCTATCTATCTATCTATCTATCTATCTATCTATCTATCTTGTGTTTGTTTTA
AATTATCTTATTTAATATATACTGTTGATGCATTACCATTGGGTTCACAGCCAAAAGCACTGTTAACTCAAACCTAAATGAAGGTTACATAACACG
TATTTGTCTGTAGGGAAAATTTTTTTTTCCTAAGTCATATCAACCTTTCTTTTTACAAATTATGGTAAAATGTACATAACATAAAATTGACCATTTAG
CCATTTTTAACTACAGCTCAGTGGCATTAAGTATGTTTACTTTGTTGTGCAGCCATGATCAACATCCTTCTCCAGAACTATCTGATTTTCCCAAAT
TGAAAACTCTGTATCTCTGAAACAGTAGCTTTCCCCTATCCCACCCTACTCCTCCTCCCAGGACCTGGCAACCACCATTCTACTTTCCATCACAG
CCTTCCTGTGCTTTGGGGCACAGCACCTCAGCACTATTCTTGGGAGCCATTTTAAACAGCAGAATCACTGCCACCACCCCCCACCAACACACA
GAAATGTGAAAAACATGGCACTATATAGACTGCAAAAAGGGCATTCATTTACAGTATGAGAGCTGAAACAAAGCAGAGAATTGCCTTGTTTGACC
TCATTTGAAGTCCTGTGAGTTTGGAAGCCTCAGTTTCCCCTGTTCTGTGCATGTGCATGAATGACTATAAAAGTGAATTGATTCTGGGTTAAAAA
TAAATTTCGGCAAGTATACAAATCTGCAAACATGGAATCCATGAATAATGAGCATCCACCGTATTTGGAAATGATGACCTTGGTTTCCTTTATTCC
AGGTGGTAAGAGTAAAGAGCTGAAAAGTGCCTTGTTAATAGGGCTGCCTTAGAACCTTTTACTTGCCTCAGAGGCCTAGAGAAGTGGCCTATGT
GAATTGATAATGGGCAGCTGCAACCTGAAGCAGTTCTAGTTCACCTGGAGGGTAAATATAAGCATAATCTCAAGCCCTTCTGCATGAAACAAAG
ACCAAGCACTCAAGTACCAGTTATCAATCACTATGTGACAGGCACTATACTCGGCAATTTACATGCTTTATTGAATTACATCGCCCCAAAATGCT
ATAATGAAACTGAAGGTTAGAGAAGTTAAGTATCTCATCCATTATTACATAGTTAGAACTGGCAAAGTTGAGATTTGAACTCAGGTCTGTCTGACT
GCAGAGCCTGAGGTCCCAACTGCTATGCAATTCTAATCAAGTTTAAAAAAAGTTTGATTTACTCAGAAGTGTATAGGAGCATAAGTTATAATTACT
ATAACATTATGAAGATTTACATGTTGAAAAAGAAATTTACCAAACAAATTAAACTTAATAAGCCTGGTCTGATATTGCTCCACAACAAAGAATATCT
GAAATTCTTCAGGGCATCTGGTTTGTGTCTGGTTTTCCTTAATCTTTAATGATGGGCAAATCTAATGCATTATGTAAGGCCATTTTTTCTCAAGAG
ATGTAGATACCTCTTAAGAATTTGATGAAAATGCATTAACTTTTCAGGCTACTGAGTTGCATTTTAGTGCACTGAGGCAGTAAATTAGTGTACAAT
GTGCAAAAGTAGTGACCTAAAAAATAAATATTTGATATGAACCACTGCATTCTCTTGGAAAAAAAAAAGTAATGGGTCAACTCTCTTAGGAGTCCT
TAGCTTCCCAAAAAGGAGTAGGAAGAATAATCTCCTGTGGCCTGGAAACAGCTTCTGTTTCTCGCTGGCTATGTTTGTTTAGCTCTTTAATAGTT
CATTTGATTAGATCTTGTGGCTCCCAAAGCTAAGGTTCAGAGTTTGATCCCTACAGAGGCCACTTAAATTTAGAGAACAAAAAGCTCTATTCTCT
GCTCCCAGACCTCACCCCAAATCCCTGCCAGGTGTCTGCCCTCTGGTCAAATGAGAAGCTGGCAAAGGGGTGCAAACCTATCACAGTGTAGGC
AAACAGAAAAAGGGGCACCCTTCATTATGGTGCTGCTTCCCTTGTATGTGCTTACAATATTTGGATATAACTTACAGAGAATAGAGCCTACATTT
TTAACTCTTACCACTGGAAATCTGAGGCAAACTTCATTACCAGTCATAAAATTCATTGTCTTCTCAGTTATTCTAAGCTTATTCTAAATTCAGGGA
GCTGACATAATCCTCTTGGTAATAAACAATGAAAAACACATCTTCTGAGCAACATTAATCTGCAGCTTTAGGACAGGAAATAACTTAATAGTAATC
AGTCAAAAGTTGAGCACAATCTTCACATAAATAAAAGATATTAAATTATTTAAAAATAATTCTATGTGCAATATAGCATTAGGATCAGTATGATTTT
CATCATGTGCTAAGAATTTAGACAGGCAA**ATG**AGTTTCTCAAATCATAGCTGAAAATATTTTACTAGTATTACAATCTTTTCAAATTCAGGACTTCC
TAATTTAAATCTTTCCTAATTTAAATCTAAATATTTCCCTAATCACACAGAACTAAAAATAATTTAAGACAGCAAATAAAATTCTTTTTACTTCAA**AT**
GTTTGCCTAAATAATATAAAATCATTTTATTTTTGGAGGAAAAAAAAATTTCAACTTTTAAAGTACGAAGTGTAAATTAAGATTTACTTAGATTATAAT
TTTAAAATTTCCCACATAAAGATTAATAAGATTTAA**ATG**TAGTTTATATTAGTGTTAACATAGATTTTAATTTTTCAA**ATG**TCACATAGATATTTCATT
ATTTGTAGATTTATTTCTTTT**ATG**AAGTAGTCAA**ATG**AATCAGCTCACCCTTGACTGTAACAAAATACTGTTTGGTGACTTGTGACAGACAGGGTT
TTAACCTCTGACAGCGAGATTCATTGTGGAGCAAGAGCCAATCATAGATCCTGACGACACTTGTCTCATCAAAGTTGGAATATAAAAAGCCACTT
GGAATACAGTATAAAAGATTCACTGGTGTGGCAAGTTGTCTCTCAGGCTGTGCAGGCATTAAAATTTTGCTTGGCATTACTCAAAAGCAAAAGAA
AAATAAAAGGAAGAAATAAGAACAAGGGAAAAGATTGTCTTGATTTTAAAATC**ATG**CAAAAACTGCAAATCTATGTTTATATTTACCTGTTTATGCT
GATTGTTGCTGGTCCAGTGGATCTGAATGAGAACAACGAACAAAAAGAAAATGTGGAAAAAGAGGGGCTGTGTAATGCATGTATGTGGAGACA
AAACACTAAATCTTCAAGACTAGAAGCTATAAAAATTCAAATCCTCAGTAAACTTCGCCTGGAAACAGCTCCTAACATCAGCAAAGATGCTATAA
GACAACTTTTGCCCAAAGCTCCTCCGCTCCGGGAACTGATTGATCAGTACGATGTCCAGAGAGATGACAGCAGTGATGGCTCCTTGGAAGATG
ATGATTACCACGCTACGACGGAAACAATCATTACCATGCCTACAGAGTGTAAGTAGTCCTATTAGTGTATATCAACAATTCTGCTGACTGTTGTT
CTAGTGTTTATGAGAAACAGATCTATTTTCAGGCTCTTTTAACAGGCTGTTCGTGTGTATGTAAGTAGGAGGGAAAAGAGTTTCATTTTTCAAGAT
TTCATGAGAAATCTAATGAGACTGAGAGCTGCTGCATTATATATTCCCTGAGGGAGCTAAAAAGCTAAAAATAAAAAATAAAATGCTTGCATAGC
ATTAATATTATATAGTTTAGCATGACCAATATGCTTATGTTTTCACAGCTTAATACCACCAAGGCAAGGATTGTAAGATACTGCAAACAATGTGAA
AAACACATGAAATTTGTAACTGCAGTTAGTTATCTGAAATATGCATTTATAACAGCAGACTTTTTGACTAATAACAGAGAAAGGAAGAAGTTTGTA
GATGTTGAAACCTATTTGGGCATTTGCTGACAACCTAGAATGACTTCTGTTATTCAAAACTATTTCTCACAGTGTTTTTATGTTCTTCACAAATTAC
AATATCTAATTTTGAAAGCTATTACACTGGAAAGTATAAAAATATTTTTAAAAAATTTAATGTATTAGTAAGAGCAATGATGAAGTAAACATAGCAT
AATAGAAATCATGAGCTAATGATTAGAAAATGCCAAGAAATAAACATTTTAATCAAATAGGTTATGGCTCACAAAGTCCTACTTATACCTTGGCCA
TGGTGCTATTGTTGAGAATACCCGGTCTGCAGATTTCCAGGCAGGCACACTGCTTAATAACCTTCTAAAATATGATTTTATTATTCATGGAAGGG
AGGACTGTTACTTGCAGTAGCTACATTTCTTCTGAAAGATAATATATTTCATATCTTTCCTGTTGCAGTCAGTTGAAAACTATACTCAAGGAAAGG
GAGACAGGCACCTTAACAGAGAAGGCATGACAAGAAAGATTTTTGTGCCATGTGTCTGTGATCTTGCTTTATCCACTTTAAACTGGACACAAAAC
AGTTTCAAAGTATTGTCTTTCTTATTAAGTAATTAGGTTATAATGCAACAAATAATTTCCTTTAAGACTGTGCTATCAGATAGTCCTGGAGTAGAT
CTGCCTTATTTATAAATAATCATGGAAAACCAAAAGGAAAGAAATTTTTAAGTGTTTCTCCTTACAATGACAGCCTGCCCCTAAAGACAATGTTCT
CTAACTTTTGAGATAGCCTGAATGCGACATTTAAATTTTGGTATTAATTAGCTGCTAGTTTTGTTCCTTTCAAAGGCTATCCCAGCGCCAAAGCAC
AACAGATGTACTATATTTTCTACTAATTCCCGTTAGTTGCTCAGTGTCTTGTCCCCAGGTAATTCAGGCCTGGGGGAAGGGTTCCTTCTTCCAGA
CTGATTGGTACAGCTGCTCAGTAAGTGTAACTACTCAGATTCCCAAAGAATTCTAAGTGGATGTTCCTCCACGGTGTCTCTTGTTCTCTCTAATC
ATCATCATTTTAAAATTTCGTCCACTGTTCATTCCTTCATAGAATTTTCCTTAGTTCACAGTTCTCTGGAAAGGAAGCAGGTTCCTCATAAACAGC
TGAAAAAAAATAGAGCCAAAAATTCTAAACAGCTATAGTAGCTATTTTGTTTGATTTTTTTTTGAGTTATGAATGAAATTCTACATAGTTTTTCACTT
AAGAAGACTAATAAGTATTTATTATTCAATAGAAAAATGCTCACTGATTAATATGGAGGGGTTTGTTCATTTTCATGAAAAATAATCTCAATAACTC
TTTTCTTTTCTTATTCATTTATAAGCTGATCTTCTAATGCAAGTAGAAGGAAAACCCAAATGTTGCTTCTTTAAGTTTAGCTCTAAAATACAATACA
ATAAAGTAGTAAAGGCCCAATTGTGGATCTATCTGAGACCCGTACAGACTCCTACAACAGTGTTTGTGCAAATCCTGAGACTCATCAAACCCAT
GAAAGACGGTACAAGGTATACTGGAATCCGATCTCTGAAACTTGACATGAACCCAGGCACTGGTATTTGGCAGAGCATTGATGTGAAGACAGT
GTTGCAAAATTGGCTCAAACAACCTGAATCCAACTTAGGCATTGAAATCAAAGCTTTAGATGAGAATGGTCATGATCTTGCTGTAACCTTCCCAG
GACCAGGAGAAGATGGTTTGGTAAGTGATAACTGAAAATAACACTCTAAAAACCTTGTCATGTTTTTATTCATAATGTGAATGAATAGTAGTGGAA

AATAACTACCAGTTTCCTATGCTCATAAGCCAGACAAAGATAACTTACCCCAATGGTAGCCCTGTACCCAATAAAAGTAGGTGTTTAGTTTCATAT
CCTATGAAACACCCTCTTGATACTTTTACTTTGCATGAGGATTTAAAAGAAAAAAGTTATAACACAGTCCTTAACTTCTAAGGGAATTTTTTTTGAA
TTGGGAATGAAATATAAAATGCTTTTTCATTGATATGCACTACGGTCATATGAATAAAAACATGAAATCTTCATAGTAGATTCTAGTACATATTCAA
CAAAACATTTTTTCCCTAGAAGAGTATCAAATGTGTTAAAATTTTTTTTGGCTTAATAGGGCAGGAAAAAAACCTCTAAAATTATAATTAAAATAAA
AAACTTTTATTTATAGCAATTAACTATAATATGTTTAGGCTTATATATTATTAAATATATATTTAAGATCTCTCATGATAAATATGTTCATTATTCTGT
AGGGTGTTGATGCAATAATGTATATGTAGATTACTTTCTGAATTACTCCTAATAAAATTAAAATTTTCAGGCTAGTTAACCTGTGCCACCCAGCTT
CTTTCTGAACTGTTTTGTCTTTCCATTCTTTTGCAAGAGTTTACTTAGGTAATGCCAACTAATTTAATATCAGGCCAAACAGATGATAATGCTTTAT
ATTTTATAAAAATTAATAAAAATCATTTTAAAACCTACTATAAATTTAGAGTTACTCTTCTGGCTTACCTGTGCTTGTGTTTATTTCTGGTTTCAAAA
ATTTATTTAATGTGATATTTTTTCCTTCCATTTATTGATAAAATTTACAACAAAAGATTACACTTACAAGCAATAAATGTTATCTTTTTAGTTTTTAAA
TGGTCTTATTTATAAAGATCATATGGTTAGTAAGTCACATCTACTTTAAATGAAAACATATTTTTTAAGAGATTACATAATTTTCCAAGTGAACTATT
TTTCTTTAACTATGCTACAAATGTTATTGACTCCCAAAATGATGTTACTGTTTTTTATAGTCTTAAATAACAATAATTACCAGGTCTATTTTGATTTT
GATATAGGATAAAAAATACTATTATTTATTTAAGAATGTGGTTTTTTTATAGGTAGCATTTTAATCATTAAAGTTGGTGATGTGACAAATTTTAATTA
TTATTAAACAGATGGTTAATTTGATGTATTTCTCAGATTCTTCCATATTCAAGGAAAAAAGTCTCAAATTCATGAAAAGATTGGGGCAGAGGAAGA
ATAAGCAGATTATTGTTTAAATATCTAAATAGAAGACTTTTTCAGTGAAGGAATAAAGGAAATATTATCAGTATCTTCTTCTGAATCTGTCCCTCTC
TTTCTTGGAGTTTGCCTTTCCAACCCAATATACCTACCACCACCTTCATTACCCCACCTTCCTTTTTTCCATTACAGTCCACACAGTGCTGGGAG
GTAACTATTTTGTTTTGGTGTTAGTATCCAAGTTTCCCCAAATAAGACCTAGTGAATGGAAGATGGATGTGTGTACCTGTCCCTCCAGGAGTCAT
CAGACATATTTAGCCACCATATTTAATCAACAAACAGGAAGAGAGGAAGCTAACCTCTCCTCCCTCTTCTTTCCTCCCCCTCCTTCTCTTCCCTC
TCTCCCTTTCTTCCTATAAATATTTTCAGAGCATCTATTATGTGCCAGGCATTCAGATACTCAAACTGGGGAAAACAAGAACAAAAAAAGACACA
GATATGACCACAGGAGAATGTATATTGCTGCTATATTGTTCTGAGCCATAAGGGAAGAATCAAGCCTAGTATAAATTAAAATTCCTTAATGCTGT
GCCTTTTAAAAACAAATGTGGTATAAGCAAAATGATTAGCTTTTGTCTTCAATAATGAGTCCCTGAGGTAGGAAAGTATTTCAGCATCTATTAGTA
TTACTAACTCTTCTTTCCTTTTCATATAGAATCCCTTTTTAGAAGTCAAGGTAACAGACACACCAAAA**AGATCCAGGAGA**GATTTTGGACTTGACT
GTGATGAGCACTCAACAGAATCTCGATGCTGTCGATACCCTCTAACTGTGGATTTTGAAGCTTTTGGATGGGATTGGATTATTGCACCTAAGAG
ATATAAGGCCAATTACTGCTCTGGAGAGTGTGAATTTGTATTTTTACAAAAATATCCTCATACTCACCTTGTGCACCAAGCAAACCCCAGAGGTT
CGGCAGGTCCCTGCTGTACTCCCACAAAGATGTCTCCAATTAATATGCTATATTTTAATGGCAAAGAACAAATAATATATGGGAAAATTCCAGCT
ATGGTAGTAGATCGCTGTGGGTGCTCATGAGGTTTCTATTTGGTTCATAACTTCCTAAAATGTGGAAGGTCTTCCCCTCAACAATTTTGAAACTG
TGAAATTATATACCACAGGCTTTAAGCCTAGAGTATGCTACAGTCACTTAAGCACAAGCTACAGTATATGAACTAAAAGAGAGAATATATGCAAT
GGTTGGCATTTAACCATCAAAACAAATCATACAATAAAAAGTTTTATGATTTCCTAAGTTTTCGAGCTAGGAGATCAAATTCCATTTATGTTCATAT
ATATTACAACATATGCAGGTAAATGAAAGCAATTCTCCTTGTGTTCTGGTGAATTAAAGGAGTTTGCTATGTCTATTTCTTTACAGTTTCATTTAAT
ATTTACAGAAAAATCTATATGTAGTATTGGTAAAATGCAGGATTGTGATATACCATTATTTGAATCATCCTTAAACACTTGAATTTATATTGTATGA
TAGCATATCTGGTAAGATGAGATTCCACAAAAATAGGGATGGTACACCATATGCAAGTTCCCATTCCTATTCTGATTCATACAGTACATTAACAAT
TCATGCCAATGGTGCTAATACAATAGGCTGAATGGCTGATGTTATCAGGCTTAACAAATAAAAAACATACAGTAATGTAATAAGTTTCTCCTTTCT
TTAGGTGCATTTTCATACTCCTCTAAATGGGGAATGGATTTTCTTTAATGAAATAAAAACTTTTTTTTAGAGGTCAGCATTCAATTCTGTAGCATAC
CTGGAGAAACTGCATTTAAAAGGCAGCCAAAAAATATTCATCTTTATCAAAATTTCAAAATTATAGCTTGCCTTTGCAACACTTCAGTTTTTATGAT
AAAATAATGGAAATGACTGATTCTATCAATATTGTATAAAAGACTTTGAAACAATTGCATTTATATAATATGTATACAATATTGTTTTGTAAATAAAT
GTCTCCTTTTTTATTTACTTCGGTATATTTTTACAGTAAGGACATTTCAAATTAAGTATTAAGGCACAGAAACATGTAATGTATGATGGAAAAGCAA
CTGCTTATATTTCGGAGCAAATTAGCAGATTAAATAGTGATCTTAAAACTCCATATGCTAATGGTTAGATGGTTATATTACAATCATTTTATATTTT
TTTACATTATTAACATTCACTTATGGATTCATGATGGCTGTATAATGTGAATGTGTGAAATTTCAATGGTTTACTGTCATTGTATTCAAATCTCAAC
GTTCCATAATTTTAATACTTATAAATATTATTAAGCGTACCAAAATGATTTAACTCTATTATCTGAAATCAGAATAATAAACTGATGCTATGTTAAGA
ATTGTTAATTTTATTTTATAATTCGATAATGAATATATTTCTGCATATATTTACTTCTATTTTGTAAATTAGGATTTTTTAATCAAATACATTGTACTTA
TGACTAAGTGAAATGATTTCTTACATCTAATGTGTAGAAACAACATAAATTATATTAAAGAGTTTTCACCTTTTTTGAAAGACACAACAGTTTTATG
CTATAATGATTAATTCTAGATTTCTGGTTTTCACTTTATTATAAAAGTTAAAAAACTTAGCACAAAAGTTTGGTTTAGAAATTTTAGATCTGCTACTC
TAGTTCTCATGAGTGAAATTCCTGTTAAATTGGTTCTGGTTAAGTTGCTTTAAACATATGAAAGCCAGGACTAGTTATATCTGTTTCATTTCTCTTT
ATCTTGACCTGAAAACTATTTATATGTTTTCATAGGTTCAATTTCCAAATGCATTGCAGTTGGCAAGGGTATATGGTCCTAGAGTTACAAGCTGCC
TTACTGAAGCCACAGGGACACAGGGAAACTGCATCTTTTTCCTAGCACTTAATGATACTGACACCCTTATCTGAGCCTTAGGGGCACCAATTTTC
AAAGTAAATTGAAAAATAATTATAAAGTGCCTAGAAATCCTTGAGTGCAACATTGTACATAAATATTCTCGAGGTGAGCTCCCTTCTCCACCTCTC
ATTAGTTTTGTAACTTCGCTCTAAAGCAGTGACTAAGTGTAATGGGGGAGGTGATTAAAATTCTACCTGGTTCTTAAATAAAAAATTTATAAGTAA
TATAAATGTAATTTTTAACCATGAATGATATTACAGCAATTCATACTCATACAAAGTGCAGTACTATTTCTAATTTGAATACTGACATATTTAATGCT
GACAAGACTGGGGCTGTAAGGGAGTTGCCCCTGTGGGGGAAGGAGCCAGCTGTCATGATAGGGTGAGGCCACTATTCAGTTGCCATCATCTG
AACCTACTTCTTTCAAGACCATGAACATACAGGACCTAAAAGTCTGGGGCAGCAGGCCCATAAACACACCTTAGGCTTTGGAGTACAAATAACC
GGGGACCTTGATCTCTATAGTGTGGACTGTGAGGAAAGAACACCCTCAGTTTCCAAGGACCCAGTCACAGGACTCCCAATCTTTATACTCACCC
TTACCATGAAGCCCCAGGGTCACATTACCAACCTCTCCATTTGGTAGCCCTCTTTGAAACCCATATGACCATGAAAGGAAGCATTTATTTTGGGA
CAAGACCTAGGCATTTCTTTTTCAATTTTGTCTAGGTCCTACAAATGGCAAAGCCACTGCCAGTCTGCCTATGGGCACATCACTTTCCTTTTCAG
CACCCCAAGAATATTCATTTGTAATATGTAATACTACTTTCTGAGATTTTCAGAAAATGCAATACGAAGTTTAATGCAGTGTATCAGTTTTACTATT
CTGAATGTTTTGTACGCCTTCCCATACTTACCGCTAACCTTAAAAAACAACGATAAAAATAAAAACAAAAAAATCCTTTTAAACTCTATGGCTCCTT
TTGTATTCAAGTTGGTTTTTGCTCTTCTGAATAGAACCCTATTTTATTCAAATAAAAAGGCAGCATTTTGACACTGAGAGTAACTAAATTAGAACTC
AATTATTCTCTCGAAAACAAAAACAAATCTATTTCATTTTAAACCAGTCATTTTAGGATATCAGTGGCCCTTTTGGATCTGTTGAAAACTAGTGACA
CTCTCAACAGGAAAAATAAAAATCACAATTTTGCATATAATTTCAGGGAGTTTAGGGCCTTCTGAGAGCTCATTCATAGACTCTGGTCTATAAACC
CTTAAGAAGGATACCAAATTTTCCTTGTTAAGATAAAAATTATTTATGAAGTAATAAAAATATATATATATAAGGGTCTGCCTCCCAGTTAATCAGT
TTTAATCATTAAACGATTTGAATGTTCAAAAAGGAAAATAAACATCAAATCAGCCTGCATGTCTTAACATGGCTAGAACTTTTACTAATTACCAAAT
ATTCTGTGTTTCTTAAAGACTATCCAATCACCAACTGGCACCTCTAATAGATTAAACTAGACAACTAAATACAAAGCACTTCAAATTATGAGTTCA

CATTCTTTTTATGTATCAAAAACAAAATCAAGAAATCATTAGGGCTTCTGATGTCTGGGTGTCAGAATTAGGAGGGCATGAGATGATAGTATTTAC
TGAGGACCAGATATGGACTAGGAACTTTCATGATGTGCTCAGTGTCGGGAGACATTATTGTCCCCAATTTACAGAAGAGAACTTTGAGGACTGA
GACAATAGGAGGTACCTCAGTGGCTGGCCTGGATTTGAGTTAAGAGCAGTTTGACACAATGATCAATTGCCCTGGTTACAACAATTCTTCACCA
AGTCCTAATGAAAGAGTGAAACTATATATTTTAAAATTCATATTTTGTAACATATCTTTATCTAGATTCAGCACTGGTTTCTGCAAAAGGTCTTGTG
GCTGCATTTTAATGAGGGAGGTTAGAGTTGGTATCAGGTAGAAGAAGAGCAGGTGATTAGCAAAGCAGGTAAATGTACACACAGAGATATAAGT
GGGTCAGTGTCATAGGATGCGAGAGCTTGAAAAGACCCTAGAGATAACTAATTTACATATGACAGAATTGAGGTCTAAAGAGAGGGTTGCCCAA
AGTTATAGAGTGAATTGGTTCAAGGACTGAGACAAGAGCTCTGAAATGGTAGAATAGAATAGATGCTGGGTCAGAGTGAGAAAGAACAGTTCCC
AAAGACAAAATACAGGAGAGCTAAAAACAAAACAAAACAAAAGTTCTGACTCATGGTTTGTTAGCTGGGGAATGTCAATATCAGACACTAGATAA
ATGTCCATAGGATTATAACAATTTACTTCTACTTAAACAAATCTTTTAACAACACGGAGATTTTCTTGGACAAATAGACACTAACGGGAAATGTAT
GCTAGCATGTAATTAGATCCACTACTTTATTGCATTATAGTGATACAAATTGAAATAATAATTAATTACTAAATAATAGTTTTCAAAGTACGTTCAC
ATTAATGATACAATTTTATCTTCATAATGCTGAAAATTAAGTATTATTATTATGACCATTTCATAGAGGGAATTGAGGCTAAAATAATCAAACTGCC
TAAGGAAATGTAGCATTTATATTTGTATATTAATTATATAAGAAATAGAAATTTAAATCAAATAAGTTATACTTGTTTCATCATTTATTCCAATAGTA
CTTATTAGAAATGTAGCATGACAAAAATCCATTAAAAATATCAAATGATCACTTTAAAAAATAATAAGACTCATGAATACTACCCGGTAATTTAACT
AAGAAATTAAACCACAGACTAAAATGTGATATGCAAGTCATGACTTGCCTGGCATGTGACTTGTAGTGTTGAAATTTCATTTGCATGGTCATTCA
GTTCCAGTTTACACATTGCAAAACATATCAAAGTAAACTATACTAATTCTGGTCATCCCTGAGTACGATGTCAAGCAATGCAAAATGGTACTAAAT
TTAGTTCATTCATGTCTAGATGGAATTTTGCAGAGGAAGTCTTTGGAAAATTAACACATTTTCATTAGATAGCATTCATTTATAGCAGGATTATCTT
TGTTACCATTTAAAACACTAGACAGAGGGAATATTTACATATATTTTACTAAATTACCTATCAAAAGACCTTAATGAATATCACATCCTGTCAAGAG
CACAAGATGTTCTTGAATGTAAGATTTTACATATGAAGCTGCCTAATTTATCTGACTGGCATAAAATGTATGTATAATAATGATAGACAACTCCCT
AATAAACTGTTATAATGGTTGAATAGTTCACTTCAGCTATGAGTTCACAAAACACTAAATATTCCTAATACAGAAATGCATAACTTGGCAATGAAG
CAAAGACATATATGCTTAGTTTTCATTAATTCCAGTTTTGGATTACTGTAAGATAGAAAAAAATTTTAACAGTATAATATGCTATTATTTCTGCTCCA
TAATAAAGGTGATTATTTATAAAACACACATATATATTTACTTTCTTAAAAAATTGACTTTTATAAAATTTTCTTCTGTTAGTTCTACATACGCACTAT
AGGCAAAACACTATTCAATGGAATTAACTTATAGAGACCTAACATATATTCCCAGATTCTGATCTTAAGAAGATCTATTGGTAAGTCCTCCTGAGG
AAGGCCAACAGATAAATTAGATTCAATTAGTCTTAAAGGTAGTTTCAAACCCAAAATGTTAAGCAATTAACCTACTAATTCATAACTTTAATCTGTT
TTCCTAACCATCCAAGATAACTCTATTAAGAAAACTTTTTTCCTATAAATGTTACGTTCTTTTTTTTTTTCTAACATCTTATGAAGGACCCTATTCAG
AACAAAATTTGATTATGCGGTTTTGGCCATGGATCCTCCACAAGACTTTCCCAGATCCGCTCCCACGGACACTAACTCTGACTAAGAAGCCAAA
CGTCTCTTGGCTCTACTCCCATGGAACTCAGAATTTTGGCACTACCTCTCCACTCACCAAAAACAAAACATGTCAATACAGGAACGTTTGCCTAC
ATCATGACATCAGATCCTCATAATGCTAAGATAGAATTCTGAAAGTGAAAATTAAGCATAAAACACCAATTTACTGAGCACACTGAAGTGACTATG
GGAGAAAACTCAAATTGTCAAATGGAAACAATCAAGCAGTCATTGAGGCAACTGCTTGGCTTGAGCTCCAAGAATATCTTCCTGAAAAGCTGCA
TACTTCTCAGCAACTCAGGAAATGGCATGAGTTATGTGGCTACCGGCTCCTTCCTTCTACCAGGAAACTCAGGTCATCTTGGGCTCTGAAGCAG
GCCAGGTGCCATGGGACAGAAATTATGCCAAGAAAGAGCAGATGAGAGTAGTCTTCTGTACTTATGGTTGCGCCTTGGCAGCAAGTAAAATTGT
CCCAAAGTCTGGAATCTACTGGGACTTATGCCTTATAGTAAACTTAAGTTCCTTACAGCTCCAGAGTCGGACAGAATTACAAGAATTTGCAAAAG
ACTGATATGGGTTCCTGTCTGGGTTCCCATCTACTCATAGGCCAAAATGCATCCCTATGGCTGAAACAAACTGCACCAGGTGGGACTCTCCTGG
GAGAACAGCCCTGCAAGGAAGTGCCAGCAAAGCTCTCTCAGAGGTGGGTAGACAATAGTGAGCCCCACCTTGCTTGACCAGATGTTTGATCAA
AGATTCCTGGTACTGGCCAAAGAGAGCAGTGTCATTTGAGGGCAAAATTTTTCCCAAAGGCTAAAATTTGGCTTTATACCATGTACTGACACACC
ATGTATTGTGTTGACACAACATACCGGTTAGCCAGGTCTCATATATCCTGTTCAGCTTGGCAAGAATAAACTACCCATAGGATCTTGGACAAGAA
AAGCATGCACATTTAGTTACAAAAGAAAAGCACATTATATAGGTATTATTATCTCCATTTTTAAAATGAGGAAGTTGAGGTTCAGAGATGGTTGTG
ACTAATGAGTGACTAAGACAGGTGAATACATAATGGGAGAAATACAAATACAAATGCAGCAGTATAATTTTTCAGCTATTAGTTCAAATGTGAAAA
AAATACAATTTTCTGATTGTTGCAAAACTGATGTCAGCTTTTACCTCAAGCTTCCTGGTGCTATAACACCATTTCTCCTTTATAAAGGCAACACTC
TGATGTCAATGGAAACTGCTCTAATAATGCTTATCATCGCTCTGGACCTCACCGAGAAAATGATACAGAATATTCCCCTGCTCTCACATCTCCAC
ACTTACAGGAGGACTGTAATGTGTCTCTCCTCCTGACATCCAGCTCAGTCTGTGAAGCATGACACCAAAAAGGATCACTGTCCGAAATTTTTCTC
CTCCCATCAAATTCACTCCCATCATAAGCTCATGAGATCTTTATGTGGGGAGATATAGAAATAGGTTCTGGCTTAACTGAATCTATTTACCAACAC
TTCATATTTCATACCTGAACCCTAGACCCTTGAATTTAAAATTCTGCTAAATACTTGCTATCATTTTTTTTTTTCTGATTCTGATCTGTAAAGTACCCC
CTGGAAAAATATAACCTTTTAATGAATCAGTGTCCATTCTAATACTACAAAGGTAAATTAAATTACATATTATCCTTTCATCTACAAATGCACTAAA
TTTTTATGTTACTATCTAGTATTATTAGTAAGGTGATCAGCAATGAACACTTTATACATTGCCTGTAGAAAAGTCCTGATCAGTTGCCTTCCAATAT
TTAACAAGATCCTTAATGTTTCTATACAGTGATTTTACTTCTAAAATTTTATCCAAAGAAAACAAAGATGTAAATATTTATGTTAAATCACAATGTTA
TTTACTGTTCTTTAAAACTAGAAATAATGTATCCAACACTAGGGAATGGTTACATTGATTATGATAGATAATAATAGCATATTAAAATTTAAATACA
ACCTGAGGGCCCAGCAAACACCATGAGGATGAGAGTGAGAGGTAGAAAAAAACTAAGCCTTCACTGATATTATTGAGATACTGATCTTACTGAA
CCTAGAGCTGCCTTACATCTGGACTTGTAGTTATATGAGATACTGTATTTTTCTACTGCTTAAGCTGTATTTGACTTGGGATTTCTGTTATTTACA
GCTGAAATATTCTAAATAAGTAAATATGTGTACAGCATGTATATACATACATATATCAGATATCTATGATTACCTTTAATTTTAACAGAGATGCCAA
TTTGTACTCTGTAGAGGTTTTATAAATTTATATTTCCATTAATAATGTATGAGGTTATCTATTTTCATACACCCTCATCATTTCTATTTCTAGTCTGA
TAGGTGAAAAATGGTATCTGATCATGATTTTAATCATATTTTCTTATTTTGAATGAGGTTATTAACATTTTCATGTTTAAGGATTATCTGTACTTCCT
TTTCTATTAACTGTTTGCTTTTATCCTTTGCTTATTTTTTTCTACTAAGTTGTTGACATTAAAATAAAACCTATATACTTAAGATATTAGCTACAACTA
TTCCCAGATTTTCATTTGTCTTTTGACTTTGTGGTTTCCTGTTTTTTAATGCAGAATTAAAATATTTGTATGATGTTACTTACATATTTTAGTCTTTTT
TATGACTTCAAGTTTTTGTCATACTTAGAGTCTTTTACATATATGCTTATATTTCTAATATATCATATTTTCTTCTAATATTTTTATGGTTTGTTATTTA
CTTTTAAAATCTTTAATCCATGTCAACTCTATTTCATAGTGTGAGGTAGGAACCAATTTACTTTTCTTCAGATGACTATCCATTTGTCACCATTTCT
AATACTATCTTCCCCTTAGTGATTTGAAATGTGGAAGGGTGCTTAGTGGACACATTCTGACCACATGGATCCTTAGATCCAAAAAGTCATGTTCA
TAGAATTTAGCAATAATATGTGATGCCTAGGGTCAAATTAGTTACTCTCTTGAAATCTAACTACAATCAATACTAATGAATATGTAAACAGTAGAAT
GATTGATGATAACAGGGTTCCTGTCCAGTCAGAAATGACCCGATACATTCATGCATATTCATATTCATGCATTTATTCCATCACAAATTTTTAGTC
TACTATTGTTGTATTTTAATTCCTACTTCACTTTAGGTATGGGACATTTTGTATAGGACAGTGTCCCTCACCTCTACTGAAGCCAAGCTCCCTTTG
TGTCACAACTCCGGAGGAGAAAATATTACACATCCCTCCTCCCCTAAAGTCACAAAAACTTCTCAGTCGCATCACTCAATGTATTATTCAGCCTC

CCAAAGGATCAATGTCTTTCTTCCTCTGTTATCAGAAGAAGAATGCAAGAAAATTATTCAGAGGGCCTCTGGTACTTTTCCTCTGAAATAACATAT
GAATTCTGTTCATATTTGTATGCTAGCCTATCAGATTTTACCAATTCATTGAGTCCTACATACCATCGCAGAAACTGTAGAGAGAGAAGAGGAGA
GAAATAAATTACTGTTTTTCTCTTGAGAAGCTCACAGTCTAGTGAGAGCAGAAAAGCACACTGTCAACTTTAATAAAATGTGACAAATGGTTTACA
AGGGGTGCTGTGGGAGAGAAGAGGAAAATTACCTCAGTCATACTAGGAGTTCACTAGAGACTTCAAGGAGAAGGTGATGTTAGCAATGTCTTA
AATAATGAATAAAAGTTTGATGGATGGGTAAGGAGGAAAAATAAATGAGGAAGAAGAAGGACATTCCAAGACAACACCCCCAACCCCCCCCCC
CnACAGACACACAGACATACATACACACACACAGACACACACACCCCAATGCAGGCATGAAAGAGCATGATGCCTTCCAGGTCCTACAAATATT
TCAAAACAGTTGGAGGGACCAACCTTGGACAGTGAGTGACAAGCCATGAGGCTCTAGAGGTAGACTAAATAGACTGAACCAAGACACGCCTTC
TATGTCTTTCTATGGAGTTTATACTTGATGTTGTCAGTAATGAGAGCCAGTGAAACATTTTAAGTAGGGAGCAACATGGTTAGAGACAAATTTTAT
TAAAAAAAAACTCACTTTAAATAAGGTGCTTTAACCTGCTTTGTGCCAAGGCCCTTTCTGATGTCTGATTAAACCTACATATCCCTTCTCAGAATG
CTTTAAATTATTTTAAATTATTTAAATAAATAGTTTAAATTAAAATATATAATTATGTATATTATAATTATAAAATATTTTAAATGTGTCCTATAATAAA
ATCTGATATCACAGAAACTTATTAATGTAAATAACAACATCAAGTAGTGAGTTGAATAACTATCATAATTTAAAAGTGATATGACCAAAAGGGGTT
AATATCCAAAATATATAAACAGCTCATACAACTCAACATCAGAAAAACAAACAACCCAGTAAAAAATGAGCAGAAGACCTGAATAGACATTTTCCA
AAGAAGATACACACATGGCCAATAGGCACATGAAAAGACACTTAACATTGCTAATCATCAGAAAAATCTAAATTAAAACCACAATGAGATATAAC
CTCACACCTGTCAGAATAGCTATCATCAAAAAGATCACAAATAACAAATGTTAGTGAGGATGTGGAGAAAAGAGAACCCTTGTACACTGTTGGTG
GGAATGTAAGTTGATGTAGCCCCTATGGAAAACAGTTGGAAGTTCCTCAAAAAACTAAAAATAGAATCACCATATAACCCAGCAATTCCACTCCT
GGGTATATCTCTAATTAAAATGAAAACACTAATTCAAAGATACATGTATCCAATGTTCATAGCAGCAGGTTATTATTTACAATAACTAAGATATGAA
AGCAACCTAAATGTCCATCAATAGATGATTGGATGAAGAAGATGTGGTATGTGTGTCTACATATACATTCACACAATGGACTAGTACTCAGCCAT
GAAGAAATAAAATTTTGCCATTTGCAACAACATGGATGGGCATTATGCTAAGTGAAATAAGTCAGAGAAAGACAAATACTGTATGTTATTACTTGT
ATGTGGAATATAAAAAATAAAACAAATGAATGAATATAACAAAACAGAAACAGATTCTAGATATAGAGAAAAAGCTAGCGGGTGCCAGAGTGGAG
AGGGAAGCACAGAGGGGCAAGACAGAGGTAGGAGATTAAGAGGTACAAACTACTATGTACCAAATAAATAAGCTACAGGGATATATTATACAGC
ACAGGGAATATAGTCAATATTTCATAACTTTAAATTTGATATTGAACATTTGTGATTTCTGTTGGTGACATGGTCACAGATACTATGAATACTTTTG
TGCTTTCAAGCCTATATTCAGAATGGAAAGAAACACCAAATTAAAATTAAATCTGAGCTAAAGTAAAGATGTAATTTTTCCCATCCAAGTCTTAGA
CCCTCTGAATTCCATTTAACAGTATAAATGACAGAGCCTAGGGATGAAAACAGGGACAGTGAATTCAATTAGAAATCTGTTTTACTAGTCCAGAG
AAGTGATGATGGTCTGAGTGATAGCTAATATTAACTGAACAATTACTGTATTCTGGGCACTGCTCAAAATGCTTTATATAGTTATCCCACCTGCA
GAGTTAGGGCTATTATTAATCTATATTTTACAGGCAAAGAGGCTGAAGCATAGAGTGCTAAGGAACTTCATTCAGTTCACACAGCTAGTGAGTAG
CAGAATCAGGTGTTGAACTGGGAGAATCTGACTCCAGAACTTCACTCTTATCTCTACTGGGACAGTAGGAATCGTGGTTAAAAGTGTATTAGAA
GTATGTGGGAAATTGGGTGAACAGGACACAGAGAAGCAGGGAGAGAAGACTGGAATCTACAGAGAAGCCCAGCTGAATTAGGAATATTGTAAA
ACTATTTGTTACCCAATTTTTTTCCTTCTTGGTAATTCTTCTCTCTCCTCCCCTAGTAGATGTTTGATCATTAAGGGTAAACCATGAGGCTGACGC
TTATTGCCTCTGGAAAAGTAGAGAAAATACTAAGAAACTGGCATATGATACTTTTTTCTGGACCAAAGAAGGAGAGACAAAGGGAGTGAGAAATA
GAGAAATCACATCCAAATATCCTTGCTTCATTTTGGGAGTCAAAACTTGGCTGAACGATGTAAGGAGAGACAAATATGAGAGAATCAAACAACG
CTGAGGCTGGAGCCATGACTCCTATGTAGCACAGAAAGTCAGAAAGCTTTAAGAGAATACCTCTCCTTGATATGGTCCTATATCTAACATGATG
CCAGAGTGGTTGAGTAGCAATGACTAAGAAGGGGTCCAGTCTTAGGCATCCATCCACATTGGATCCTGAACTCTCCAGACAGTTTCCAAGAAAG
TTTCCAAGAGCCACCAGGTCTCCAAGGGAGCTTGTTGGAAGTGCTTCTGTACTGGCTGCTGGGTTTAGATGATTACAAAGTACAGGGACTTCAT
AACTGGAGACAAGAGTGTTGCCATCAATAATAGCAGGAGTCAGCACAATGCTCCAAAAACCAGGTAAAACCAGCAATATATCAGTGGTTATAAG
TACAGGACACTTAGTTACCAGGGAGTCCAGTTAAATTAAAGCAGATACATCAAGAGGAAAGGATGATGCTCTGAGGCCAAATAACATCAAGAAG
GTTGGGAGGGAGGACCAGAAGCCCTTTTGCAATGCCAGGAAGTTGAGTGAGCCTCTTCACTCAGATTCATTTAGAAAAGCACAATGGGGGTGA
GAGAGAACATTTCTGAAGTGAGAAATGTTGAAAAAAATAAGAACCCCACCCAAACCTAAATTTTTTGATGATATCGAATTTATTCTTATTTTTTAAA
TTGTGGTAAAAAACACACATCAGAAAATTTACTATTTTTTCCCATCTTAACATTTTAAAGTCTGCATTTCAGTAAGGTGAAGTGTATTCACATTGTT
GTGCAACTGAGTTCTAGAACATTTTTTATCTCATAAAATTGAAACTATACACATCAAGCAACTCCCTCTCCATTAGCGTCCCCTGTCCCCACCACT
GCCACCTGCAACCCCTGGCCAATGCTGTTCTACTTTCTATGAGTTTGAGTACTTCAGATACCTCATTTAAGTGGAATCATACTTATTTTTAAAAAG
CCTAAAATTTTCCCTGGAAAACAGGTAACTTTGATCGGCAAGCTCAAGCACTTTCCATCATTAGGAGGAGATGAGGTTTAAAAGCACGAGATCA
GTCAAAGAAAATAAAAAGGTTACATCTCTTCAATCTCAGTTTGTGGCCAACACAACTCAACCTTACTAGATGTGGGGTCACACCACAGACCCTGA
GGAAGAAAGTACATTTCTCAGACTCTGGAACTTTGACTCTCAGTCTCCAGAGCAGCAGAATATCAGCTGAGCCCTGTCTCGTGAAGCCAGTATA
GCTCCACTGTGGTTTTCTTAGTTCTTTTAAACTGTTCTTAACTCCCACATTACCAGCTGCAGGTACTCCCTCCACCCTGGCTTTGGAGTTTATCCT
CTAACTACGTCCCAGCTCACTGTATTTAGGCCTTTATTCATCTCTGCATTCCCCTTTTGAAAAGTCTCAATACTTTGAAAATTCAGAGTTCAAGCA
AAGGGACATGCTCTTCAAAATTGTGCATAGCATTTCTTTCCCAAGTTTCTGGCCCTGGCCTCAGACTGACTTGGAATTCTAATCTAAGATTTATAA
TCACTCTATGTTGCAGATGTAGGCTGTGTCTAAAATCTTGGGCATCCTAGACAGGTTCTGAATGACTACAGCATTTGGTGCAAATAAATACTACT
GTGGGACCGTGTCCATCTTCCAGTCTGGGTAGCTATGGCAAAGAATCAGATCAAGTCTGATTATTCTGTGGGTAGACTTCAAGGATGTCTGATC
CCTGAGAGTTGAGTCATTCCAGGTCTCAGTGGAACTTTCAGTTCAAGCAATCATCAACATCATTATCAAGCAGCTATTTGGCCTTCTCTGAAAAT
AAAATGAGATCTCTACCTCTTGGTTGATAATTTATCTGTTGTGTTGTGCTATCAGTTTCAAAGATAGTGATAATATATAAATTCTTTCCTTTACAAA
TTGCCAAAATTTGTTTAATAACATAGAGTAATTCCTAAACATTTACATCACAGATTAGATGCTTGCACCTTGCTAAAGTAGTCTATGTACTTGGTCT
TTTAGGAAGCTATTCCAGCAGTTTTACCTTAATCTGTTATGCTTAGAGGGTACACCGGATAGAGATCTAGTATAGGGCCTCATTGTCTGTGGTAC
TCAATGGTGGTTGAAATGAAATGTTGATAATAATAATGTATTTGAATCTATTCCATACCATACCAAGGTAAACCTAAATAAAGCACATTTAATTTTC
TAATAAAATTGAAAATATGTCATAGTCTTAAATCCATGACCTTTCAAAACTCTGATGGCAAAAAAAAAAAAAATACTGTCAAGGACTTAGAACTCTT
GACTTAAATTATTTCACCAGGTCAAAGGATGATGTACTAGGATGTCACTGTTTATGCTTTCTTTCCAAAATATTCAATGGAAATGACACCATTTTA
ATTTAAAAAGAATTTATTTTAACCCTACAAATAAGGCTCATTTCTTCAGTAGCAGCTTCATTTAACCAGAAAAACAAATCTTCTGGGATCTGTAGTC
TTGTATGATATAGCCTGATTTAAGCCCTAACTTTCCTAAATCCAAACACGTTACAAAGATCAAGTCAGCTGTACTGTGCTGTGGCCCACAGCCAC
ATTGTGATTTTGGGAGAACATAGTTGGCTGGGCTGCACTGTGGTCTCTCCAAGACAACTCTAGTCAGGATCTTGCCAGCAGTAAGCAGCAATAT
GTCTTGATAGTTGCCTCAGTGAATCTCACTGCTTTGAGTAATAAAAATAACCAAGCACTTTAAATCAAAGATTTATTAGTAACAGAAATTTAAAACT
CAAAAAATTGGATAGTTTTCTTAGTTTAAAATTTTGCACTGAATTTTTCACTGCAATAGTGTGCCCACTTTGGAAAACAGTTTGGCCGTTTCTTATC

AAAGTAAACATAGCCATTATATGATCCAGAAATTCCATGCTTAGGTATTTACCCAAGAGAAATACATACAATATTCACAGAAAATCCCAAATGCAA
ACATTCAAACTAGTTTCAGTCATAATAGCCTAAACGGGATAATCTCAATGTCCATCACCCGCTGAATGGATAAACCAATTGTGGTACCTCCATAA
ACCAGAGAACCACCGGACAATTATTGATACATGCAACAACACAGAGGAATCTCAAAAGTCTTATGTTAAATGGAAGGAGCCAGACACAAAAGGC
TCCATACTGTTTGAATCCACTTACATGACATTCCAGACAAAGGCAAACTCTAGGAACAGAAACAAATCAGTGGTTGCTAGAAGATGGGTGTGGG
TAAAGAGACTGACTACAAAGTGGCACAAGGGAAATTTTTGTGGTGATGGAAATATTCTGTATTTTACACATGCACAACTATGTGTGTATGTGTTC
CAGCTCTTTAAAGTGTACACTTTAAAAGGGTACATTATATTTTACATAAAAAACACCTCAATAAATTTGCCTCTAAGAAAATCTCGGGCAGGGACA
GTGAGTCGGGGAATAGATGAAACAAGACTGGCCATGGGTTAAAATGATTAAAGCTGGGTAATAGGTCACTCGAGATTCACTAAACTATTCTCTC
TACTTCTGAATATGTTTAAACTTTTCCATAGTTAACAGTTAAAAATTTTTTTGCACTATAGACTGCTAGGTTTGTGCCATTTAATGTTCTCCAATCAT
CTTGCCCCACTTTTTTCTAGGCCAAAATGTAAAACCTGTGGAACTTCATACAATTCATCAAAGAAAAAAGCCCATTAGCTATTTTTTCAACATACA
AACACACACACACACACACACACACAAATAAACCATCACGAAAACTATCTTGAAAGCCATAGTTAATAAAATTATGGTCATGATTTTTAAAATTAA
GTTCATTAGATGCAAAAATGTTTAAATGTACCTCTATAGCTATTTCCATAGGTCTTTCAGAGTTCATCTGAGACAAGACATTTATTTAAATGCCACA
AAAATAAAAAGAATGAAGCTGTAATTATTCATGCAATCTTTCATTTAATAAACATCTCACATGTCTACTACATGTTAAACACACAATAATCTCGCTT
TCAAGGAGTGCACAAATACAAATACAACCAGCTACAACACAATTGCTGACTGTGTGGAAAGAAAAATTAATGCTGCCTAGAATGGAGAGTAGAT
ATGATTGGGGAGAGATGGTGAGAGGACTAGAAAAGAATCTACAGATACTCTAAGGGTGAATTTGGTGAGGAGAGACAATACATAGACAACCAA
CATTTGAAACTTTGGGTATTTGGGGATAAAGCTACAATAAGTGTATCTTAAAGTTAAAAAAAAAAAAAAAAAGAAACTACAGATACTCTGAATCTA
GAAAGATGAGTTAGAAGTTTACTTGGCCAGGAGTGAGGTGTGGATCGGAGGGCAGGGAAGAAGGGAGAAAGAAAAAGAGCAGATTCTAGGGA
GACATGAAGACAAGTACAAAGCACATTTGGGGACCGCCTGGAGTAATTAACACAGAAGCAGCAAAACAAAACTGAATAATTCATTTATAATCAG
CCCAATCTGTTACTGCAAGTCCATAAACTGTGCAATTTATAAATGCATATTTCTAATGAATTCATGGATTTATTCTACAAATAAATAAGGTGATTTG
GTCCCAAGGTCACAAGTTATATCCACAACTCAGGTCAGCTAGTCTATAAAGGTATGCTGCTAACTTTGAGGCAAGAATCTGGACATACATCTATT
GCCTTTATTGGAAAACCAATTTAAACTTCATACCATTACTATTTTTGCCTATAAATAGATGTGCTTAAGGTTCATTCTTTTTAGCCCTTCCTTAGCA
TTCTGGATGGAGTTGCAGAAGGAGAGAATAATTTGTTAGTTAAATTTAAGAGACTACATAACCTAACCAAGGAGCATTACTAAGTCACTTCCTAA
GCAGACAATCATGAAAGTAGAAGCTTGCAATTAATAAAATTATTAGATTATTTTCAAATCTGCTTCTTTGTTCCACCTGGCTAAAATCTTGTTGTTG
GCATAGCTGAGACTAAATTCATGTATATAGACTTGCATACCATCATGATCGTTTACTGAGGAAAAAGAATTGTTCTCACTATATGATGTCAGTGAG
GATGAATACTTTTCTTTCTCCTCTCCTTCACCCACTACCAACTAGGGAGGGTTGAGTGGGCCACTTTTATTTCTGATCTTTGGTATATAATACAAC
AATGTGTGAATGACACATACTAACTTTTAAATTACAACAAAAACAATTCTTTCAGTAATAATTATGCATGATGTCAATTCTTACACATTTAAGGGTA
TACTGATGCAACGATAAATGCTGGAGAGAGTGTGGAGAAAAGGGAATGAACCCTCCTAGACTGTTGGTGGGGATGTAATTTGGTGCAGCCATT
ATAGAAAAGAGCATGGAAGTTCCTCAAAAGACTAAAAATAAACTTACCATATGATCCAGTAATCCCACTCCTCGGCATATATCCAGAGGGAACCT
TAATTCAGAAAGATACATGTACCCCAATGTTCACAGCAGCACTATTTACAATAGCCAAGACATAGAAACAACCTAAATGTCCATCGACAGTAGAC
TGGATAAAGAAGCTGTGGTATATTTATACAATGGACTACTACTCATAAAAAATAATAAAATAATGCCATTTGCAGCAACATGAATGGAACTGGAGA
ATGTCATTCTTAGTGAAGTAAGCCAGAAAGAGAAAGAAAAATACCATACGATATCACTCATTTGTGGAATCTAAAAAAAAAAAAAAAGACAAACAA
ACTTATTTATAAAACAGGAAACAGAATCACAGACATAGAAAACACACTTATGGTTACCGGGGGGAGGGGAGTAGGAAAGGATAAATTGGGAGTT
TGAGATTCCAGATACTAACTAATATATATAAAATAGATAACAAGTTTTTACTGTATAGCACAGGGAACTGTATTCAGTATCCTGTAGTAACTTACG
GTGAAAAAGAAAATGAAAATGAATATATATTTTGTTCCTATATGACCGAAGGATTATTCTGTACACCAGAAACTGACACAACATTGTAAACTGACT
ATATTTCAATAAAAATATATATACAAAAGAATTAAAGAACAAAAGTGATAATAAAAGAGACATCCAAAAATTAAATTGAATTATTTAAAAAAGCATA
TTGATGCTTCTAAGTTAAATTCCAAGCAGAGAATAAATTTGAAATAAATTAGAAAAAAATAATTCTTGTGCATGATAAGAAGTAGCAACTTAATGCT
TTATATTATTTGATGATTCAAAAGATAGACAAAACACTTCACCAAGAAAAAAACCCTCAGATGTAATAAGAAAGCAATCCACTGAAAAAACACACA
TGGTCAATTTTAGGAGTAAAGTAACCTAGTCAGAGGAGCAGGCCCAAAGACGCTTTTCTACTACACAAGCACACTTGAGCCTATTTAAAAAGGC
ACATTAGTAGGGGTGAGGAGAGTTCAGGCCAGTTAGTCCTTGGCGCCTCAATCAAGATTGCCAGCATAGTTATCAATTAATGTTGCAGTGGTTT
CAGTAATGCATACACGTGTCAAATAGGAACTCTACTGAGTCTGCCACTTCATTTTACACACGATCCTGAACACCTGTCAGGTCGTGATGATTTTC
AGTCACTACTGACCCAGGATAAATTTTAACAGGTGACAGGCTTTGGATCAAATTACCAATTCCCTGAGGCATCCAGTCCTCCCAATCCTAAGTAT
TTTTGGCATGATTTATATGTTCTTTAGTTTCCTTTATAATGTAATTTAGAGTTATGTCAATTCTAGTACTATTCTTGAAATGTGAAAAAAACCTAGTG
TTCAGTTTTTTAAATATCCTTAACAGAAAACTGAACAATAGAACATAGAGGAAAACTAGTGGGTTTTATTATTGTCTTAAAACCTTCTTGTGCTAAA
TAAAAGCAGGCAATTCATGTTGGTCTCATAAATTAGATATGAAATTTTAGCAGCCTATGAATTTAAATGTCTGCAGACTGCTGATTTATAAGGATA
AGGTAAAAACTAATGAGCATGGGATTCAAGACTCTTCACAATCAGGCTTTTGCTTGCTTCCCCAGATTTACTTCTCTATACTGACTTTCCCTGCT
GCACACCTCATACTAGTGAATATTCAAGTATAAACAATTTCCAGGAAACACTGGTCATTTCCATACCTTCATGACTTTGCTAATATTACTCTTTCT
GCCTGAAATATCCATAACCTCTCTGCCTGGCAAAATCCTCAAACTTCTGACTCCTCACTTCTTTTCTTTCTTAGCAGATGATAATAGCCTTCCATT
CATGAGGAAAAGGGTAACTAGTATCCACCAACTGTACATGCTAGAAAACTGAATTATCCTTGGTGGACATTCTCCTTCACGTCTTCAAATCTAGT
CCATCACTAAATTTTGGTCATGTTTCTAAAATCCCAGAACCCATTTACTTTTTTTGATGTCCACTGCAATCTAGTTAGTCAAAGCCACCATCATATC
TTGCCTAGACAACCACAGATAAGATTAACTGGTTTCTTTGCTTGTATTCTTCCCTGTCTCCAAATACCTATCCAGAAGGTACCAAAGTGCCTCTTC
CAAGACTGATATTGGAATCCAAGTTCACAATCCAGGCATATTGTGGTGAGCCAGATGTTAAAGGTGAGCTAGTAAATCTAAACTCGGCACTCCA
GCAGGAGGAATTTGATGGAAAGCTAGAATCTACATGAGAAGCTGAAAGTAGTTATGGGGAACTGGTTATTGTAAGGACGTATGGGTTTGAATAA
ATTCCCAAGACAGAATTGGCCACTTGTCACTTACTTCCAAGAATGTCTGAATGAGTCCTCATGTGAAGGTGTATCTGAACCCACGAGAATGCTAA
AGGAAACGATGTAGTGAGAGATGAGAACAAGGACAGAATCTGGAGGGACACCTATATTAAGGGGATAGGAAGAGAAACCAGAAAGCTTAGTTG
GCAGTGAGGGAAATAGGAGAGCCAAGAAAGTAGTGTGTGTGTATTTTATTTATTTTTATTTAAAAACTAGATTATTTCTGATAGATTCTAAAAAAG
CCCAGCTAATAGAATTCTAGATTTAAAAACTATACTTGGGTAAAGCTGATATTTGTGTACATAACAGGAAGAGGAAGGGCTATAAGAATCAGGGG
CAAGGGTATTATTAAAGAACCAAGTAACAATAATGCAGTCATGAGGTGATTTTGCACCAAACAACATGGTGAACTATATGTAAATATTACTATATT
TGGTATATACATATGTAACATTGAAGATCTTAGAAACAGAACTCTCAGGCATGTTACCCAATGAACTTGTTTTTCTGAAGGATAAACATATGTTCC
TTTAATGAATCAATTTATTTTGATTACTTTTCTAACACCTATAAAATTAAACAATATAGAAAATAAAAAGTAGCAAGTAAAAATCATCTAGAATTCCT
CCTACAGAGCCATACTGTTAACACTTGGGTGAACATTCTTCTAGAAACATATAGTCCTTCTCTCTCTCTCTCcacacacacacacacacacacacacac
acaCACACACACACACACACACACACACACACACACACACACACACACACACACACACACACTCTTACATATATCTTAGGGGTCAGCAAGC

TAAGGTGATTGTCTGTTTCCATAAATAAAGATTTATTGGGGGGAGGGTATAGCTCAAAGTGGTAGAGCACATGCTTAGCATGCATGAGGTCCTG
GATTCAATCTCCAGTACCTCCTCCAAAAAAAAGTAAATAAATAAATAAACCTAATTACCCTCAAAAATCTAAAAAAAAAAAGGTTTATTGGAACATA
GCCTCGCTCACTTGTCTACACATTGTCCATGGCTACTTCTGTATTACAATAGGAGAGTTGAATAGTATGACAGGGACTCTACGACCCACAAAACT
TGAAATATTTACTACCTCGCCTTTTACAGAAGTTTGCCAACCTTGATATGAAGAATCATATCATACATGTTCCATTATAATCTGCTTTTTCATTCAA
CAATATAACATATAATATTTTTACAGATCAATATAGGTCTCTATACTTCAGTAAAAAGAAGGAAAAAACTATATATAGTTTGTAAAAAAAAACAGAT
CATCACTTTAATGGCTGCATGGAATTCTGTTGTATAAAGGAGCCATTGTTTATTTAATAAACTTCTACTGATGGATAGAGTTCTAACTCCTATATTA
TAATCAATGCTGCCACAAACATCCTAACATAGACATCTTACTACACTCAATTACTTTCTTAAGATAAATTCTTAGAATCATGGGGTCAAAAGTTAC
ACATATTATACATTTGATATATATTTATAACCTGCTTTCCAGAATGAAATAATTTTCATTCTTATCAATAAATAATGAAAGTGTACATTTTTTCTATTC
TATCAATCCTGAGGATAAACAAGCATTTTTAATCTTGGTAAAAAAAAAGATCTATCAGTTGTTTCCATTTGTACTGTTTGATTCTTATGAGGTAAAG
TATCTTTTATGATTATTGGTCACTTATGCTTCTTTCCTTGTGAATTAATGGACTGCATTACTTTATATTTACATAAAAATGTACTTCATGTACTTTTC
CAATCTTTCTGTTGGAGCATTTCTTTCTTTTCTCTTTTATTTTTATTGAAGCATAGTTGACTTACAATATTATGTAAGTTACAGGTGTACAACATAGT
GATTCACTATTTTTAAAGGTTATACTCCATTTATATTGGCTGTATTCCCTGTGTTGTACAATATATCCCTGTAGCTTATTTGTTTTATACATAGTAGT
TTGTACCTCTTAATTCCCTATCCCTATCTTGCCTCTTGCTCCTTCCCTCTCCCCACTGGTAACCACTAGTTTGTTCTCTGTATCTGTGAGTCTGAT
TTTCTTTTATTATATATACTAGTTTGTTTTATTATTTTTAGATTTCACTTACAATTAATATTACACAGTATTTGTTTTTCTCTGTCTCACTTATTTCATT
AAGCACAATACCATCTAAGTCCATCTATGTTGTTCCAAATGGCAAAATTTTTATTCTTTTTTATGGCTGAGTAGTATTACACACACACACACACAC
ACACACATATATATATATGTATGTGCATATATATATATATATATATATATAT
